# Transcriptome insights into root adaptation of *Erigeron canadensis* to combined cadmium and lead stress

**DOI:** 10.1093/aobpla/plag053

**Published:** 2026-10-15

**Authors:** Haiyan Zhang, Shanshan Qi, Yajie Shi, Zhicong Dai, Guangqian Ren, Daolin Du, Zhen Ji, Yuying Deng

**Affiliations:** School of Inspection and Testing Certification, Changzhou Vocational Institute of Engineering, 33 Gehu Middle Road, Changzhou 213164, China; School of Agricultural Engineering, Jiangsu University, 301 Xuefu Road, Zhenjiang 212013, China; School of Inspection and Testing Certification, Changzhou Vocational Institute of Engineering, 33 Gehu Middle Road, Changzhou 213164, China; School of the Environment and Safety Engineering, Jiangsu University, 301 Xuefu Road, Zhenjiang 212013, China; School of the Environment and Safety Engineering, Jiangsu University, 301 Xuefu Road, Zhenjiang 212013, China; Jingjiang College, Jiangsu University, 301 Xuefu Road, Zhenjiang 212013, China; School of Inspection and Testing Certification, Changzhou Vocational Institute of Engineering, 33 Gehu Middle Road, Changzhou 213164, China; School of Inspection and Testing Certification, Changzhou Vocational Institute of Engineering, 33 Gehu Middle Road, Changzhou 213164, China

**Keywords:** invasive plant species, heavy metal stress, root development, adaptive strategy, transcriptome

## Abstract

The invasive plant species, *Erigeron canadensis* L., exhibits a propensity to colonize heavy metal-contaminated areas. However, its molecular adaptation mechanisms to heavy metals remain poorly understood. This study explored its adaptive mechanisms under combined cadmium (Cd) and lead (Pb) stress by investigating growth performance, heavy metal accumulation, and root molecular responses. Aboveground growth of *E. canadensis* was unaffected by any treatment, whereas root biomass was reduced only under high-concentration combined Cd and Pb stress. Cd and Pb were predominantly accumulated in roots instead of aboveground tissues. Notably, a translocation factor (TF) > 1 for Cd implied that *E. canadensis* actively accumulated Cd in roots and translocated it to aerial parts. However, a TF < 1 for Pb suggested that this species employed an exclusion strategy to mitigate Pb stress. Differentially expressed genes (DEGs) in *E. canadensis* roots were primarily involved in transport, signal transduction, antioxidant defence, transcription regulation, and ribosome function under heavy metal stress. Zinc transporter *ZntB* was verified to mediate Cd uptake and tolerance, while natural resistance-associated macrophage protein 3 and cyclic nucleotide-gated ion channel 2 were shown to mediate Cd and Pb uptake and tolerance by heterologous expression in yeast. Up-regulated amino acid, sugar, and auxin transporters together with ribosomal proteins may contribute to the maintenance of nutrient supply, auxin homeostasis, and protein synthesis for normal root growth. High-level metal stress triggered more extensive down-regulation of ROS-associated genes in *E. canadensis* roots. These findings provide root-specific molecular insights into the *E. canadensis* adaptation to heavy metal stress.

## Teaser

*Erigeron canadensis* is an invasive plant that thrives in heavy metal-contaminated soils. Our study investigated how its roots adapt to combined cadmium and lead stress. Surprisingly, aboveground growth remained unaffected even at high contamination levels, with only root biomass declining under the highest concentration. The plant accumulated cadmium and lead in its roots. Cadmium was transported from the roots to the shoots, while lead was retained in the roots. Gene expression analysis revealed that the plant activates transport, signalling, antioxidant, and protein synthesis pathways to cope with stress. The roles of three key transporter proteins in heavy metal transport were also confirmed. These findings reveal how *E. canadensis* adapts to heavy metal stress through root-specific responses, offering insights into the use of this plant to clean up contaminated soils.

## Introduction

Preventing and controlling invasive plant species is crucial for maintaining ecological security (de Oliveira *et al*. 2023). Research on invasive plant species and the mechanisms driving their successful invasion has grown increasingly prevalent (Zhang *et al*. 2020, Khan *et al*. 2023). Given the complexity of environmental problems, the interaction between pollution-driven environmental alterations and plant invasion has garnered increased attention, as these environmental shifts are expected to facilitate the spread of invasive plant species (Martin *et al*. 2025, Savoca *et al*. 2026). Environmental stressors such as heavy metal pollution can disrupt ecosystem balance, alter existing biological populations, and provide opportunities for invasive plant species to spread, ultimately leading to the formation of monocultures (Gulezian *et al*. 2012).

Soil heavy metal contamination is a critical global environmental challenge that threatens ecosystems and human health (El-Sharkawy *et al*. 2023, Kama *et al*. 2024). The National Soil Pollution Survey Bulletin of China (MEP of China) reported that 16.1% of soil samples exceeded the Grade II environmental quality standard (GB 15618-1995), with 82.8% of these exceedances attributed to inorganic pollutants, including cadmium (Cd), lead (Pb), nickel (Ni), copper (Cu), arsenic (As), and mercury (Hg). The Ecological and Environmental Status Bulletin of China in 2022 and 2023 further indicated that heavy metals are the primary pollutants affecting agricultural land quality, which has emerged as a pressing ecological issue. Furthermore, soil heavy metal pollution frequently occurs with a dominant heavy metal element alongside the presence of other elements, a phenomenon referred to as co-pollution by multiple heavy metals (January *et al*. 2008). Co-occurrence network analysis has confirmed a positive correlation among several heavy metals (Zhao *et al*. 2021).

Invasive plants have evolved a series of adaptive mechanisms that enable them to tolerate and even thrive under adverse environmental conditions (Uveges *et al*. 2002, Fidalgo *et al*. 2013). Under heavy metal stress, these species have been shown to enhance their stress tolerance through physiological regulation, such as modified root biomass allocation and altered nutrient uptake strategies (Uveges *et al*. 2002, Khan *et al*. 2023). Some invasive plants, such as *Praxelis clematidea* (Griseb.) R.M. King & H. Rob., *Ageratina adenophora* (Spreng.) R.M. King & H. Rob., and *Solidago canadensis* L., have been reported to exhibit high heavy metal tolerance and accumulation capacity while maintaining root growth (Dai *et al*. 2020). However, despite these physiological observations, in-depth studies on the molecular regulatory networks that govern the responses of invasive plants to heavy metal stress remain limited.

*Erigeron canadensis* L., native to North and Central America, can propagate rapidly and establish populations outside its native range, forming monocultures by displacing native species (Weber *et al*. 2008). It reduces biodiversity, destabilizes ecosystems, and causes agricultural losses (Weidenhamer and Callaway 2010), infesting orchards, roadsides, and farmlands globally (Queiroz *et al*. 2012). Previous studies have demonstrated that *E. canadensis* can grow in heavy metal-polluted soil and exhibits tolerance to Cd and Pb (Zhou *et al*. 2015, Li *et al*. 2016, Wright and Steven 2024). However, most existing studies primarily focus on the heavy metal accumulation capacity of this species, with a lack of molecular-level investigations into its underlying adaptive mechanisms.

As global heavy metal pollution intensifies, invasive plant species face pressures that may reshape their adaptations and ecological competitiveness. Therefore, understanding their molecular responses to heavy metals is crucial for invasive plant species management. Cd and Pb are the predominant heavy metal pollutants in Chinese agricultural soils (Yang *et al*. 2018), and *E. canadensis* exhibits strong tolerance to both metals (Zhou *et al*. 2015, Wright and Steven 2024). Given that Cd and Pb commonly co-occur in field-contaminated soil (Zhao *et al*. 2021), and to reflect such field-realistic conditions, this study focused exclusively on combined Cd and Pb treatments to elucidate the underlying adaptive molecular mechanisms of the invasive plant *E. canadensis*. Specifically, we investigated growth performance, heavy metal uptake characteristics, and root transcriptome profiles of *E. canadensis* under combined Cd and Pb stress. Insights into the interactions between invasive plants and heavy metals can provide valuable implications for managing invasive plants in contaminated soils and conserving native plant communities.

## Materials and methods

### Study design

*Erigeron canadensis* seeds were collected in October 2022 from a vegetable field adjacent to Jiangsu University (32°12′17″N, 119°30′17″E), Zhenjiang, China, and stored at 4°C until use. For the pot experiment, seeds were surface sterilized with 5% sodium hypochlorite for 10 min, rinsed 10 times with sterile distilled water, and then sown in seedling trays and cultivated in a greenhouse under natural sunlight. Uniform-sized seedlings were transplanted into pots (23 × 18 × 22 cm), each filled with 4.5 kg of sieved and homogenized yellow-brown soil. The soil was obtained from a relatively unpolluted vegetable field. No prior natural invasion or established population of *E. canadensis* or other invasive plant species has been recorded in this field. The background levels of Cd and Pb in the soil were 0.11 and 27.0 mg/kg, respectively, and its basic physicochemical properties were as follows: pH 6.70, soil organic matter (SOM) 6.13 g/kg, total nitrogen (TN) 0.83 g/kg, total phosphorus (TP) 0.73 g/kg, and total potassium (TK) 16.9 g/kg. After 2 weeks of acclimatization, seedlings were exposed to combined Cd and Pb stress, simulated by adding cadmium acetate [Cd(CH_3_CO_2_)_2_·2H_2_O] and lead acetate [Pb(CH_3_CO_2_)_2_·3H_2_O]. To ensure homogeneous Cd and Pb distribution, air-dried soil was spiked with metal solutions and mechanically stirred for 60 min. Homogeneity was verified by 12 random subsamples with a relative standard deviation (RSD) of less than 10%. The spiked soil was equilibrated for 6 weeks before transplanting and no notable variation in soil pH was observed.

The combined Cd and Pb concentrations were selected to simulate realistic soil contamination levels rather than background values. Based on Yang *et al*. (2018), the 90th percentile values of Cd (∼1 mg/kg) and Pb (∼90 mg/kg) in Chinese agricultural soils were used as the low concentration (LC) group. The moderate (MC) and high concentration (HC) groups were set at two- and four-fold of the LC level, respectively, considering that heavy metal levels in industrial and mining areas are substantially higher than those in agricultural regions (Yang *et al*. 2018). Thus, four groups were established as follows: (i) control group (CK, with background levels of heavy metals and no external addition); (ii) LC group (1 mg/kg Cd + 90 mg/kg Pb); (iii) MC group (2 mg/kg Cd + 180 mg/kg Pb); and (iv) HC group (4 mg/kg Cd + 360 mg/kg Pb). These concentrations represent total soil metal concentrations, including the initial background levels. According to the Soil Environmental Quality—Risk Control Standard for Soil Contamination of Agricultural Land (GB 15618-2018), the risk screening value indicates a potential risk requiring safe utilization, whereas the risk intervention value indicates a high risk requiring strict control. For soils at pH 6.5–7.5, the screening values are 0.3 mg/kg for Cd and 120 mg/kg for Pb, and the intervention values are 3 mg/kg for Cd and 700 mg/kg for Pb. Based on these thresholds, Cd surpassed the screening value in all treatments and exceeded the intervention value only in the HC treatment, whereas Pb exceeded the screening value in the MC and HC treatments and remained below the intervention value in all treatments. Since only combined Cd and Pb treatments were applied in this study, the individual contributions of Cd or Pb to the observed responses cannot be distinguished. Each treatment had three independent biological replicates, with one *E. canadensis* seedling per pot. Native plants were not included as controls, since this study focused on the adaptive mechanisms of *E. canadensis* under combined Cd and Pb stress rather than on comparing invasive and native plant species. All pots were completely randomized in the greenhouse and rotated weekly to minimize microenvironmental variations in light and temperature. Plants were grown in the greenhouse at Changzhou Vocational Institute of Engineering (31°41′07″N, 119°56′40″E) under natural sunlight, 24–28°C, and a 14 h light/10 h dark photoperiod. Purified water was supplied to all plants as required.

### Sampling and assessment

Following 2 weeks of seedling cultivation and another 2 weeks of acclimation, the plants were subjected to 3 months of heavy metal exposure, after which soil and *E. canadensis* tissues (roots, stems, and leaves) were collected from three biological replicates per treatment. Plant tissue biomass was determined to assess the impacts of heavy metals on *E. canadensis* growth. Soil samples were collected from the top 5 cm layer using a 5 cm diameter core from each pot and digested with aqua regia (37% HCl: 70% HNO_3_ = 3:1, v/v) at room temperature for 16 h (Melaku *et al*. 2005). Fresh *E. canadensis* tissues were cut into small pieces, oven-dried at 70°C, and then digested with 70% HNO_3_ at 125°C for 1 h (Baghour *et al*. 2001). Cd and Pb concentrations in all samples were determined by inductively coupled plasma–mass spectrometry (ICP–MS; iCAP RQ, Thermo Fisher, USA).

The bioconcentration factor (BCF) was used to evaluate the transfer and accumulation of heavy metals from soil to plants. It was calculated as the ratio of the heavy metal concentrations in plant tissues to that in the soil within the rooting zone using Equations (1)–(3):


(1) $$BCF_{\mathrm{root}}=\frac{C_{\mathrm{root}}}{C_{\mathrm{soil}}}$$

(2) $$BCF_{\mathrm{stem}}=\frac{C_{\mathrm{stem}}}{C_{\mathrm{soil}}}$$

(3) $$BCF_{\mathrm{leaf}}=\frac{C_{\mathrm{leaf}}}{C_{\mathrm{soil}}}$$

Here, *C*_root_, *C*_stem_, *C*_leaf_, and *C*_soil_ represent heavy metal concentrations in roots, stems, leaves, and soils, respectively. A BCF > 1 suggests a high capacity for accumulating metals from the soil (Ba *et al*. 2024).

The translocation factor (TF) was employed to evaluate the distribution of heavy metals in plant tissues. It was calculated as the ratio of the heavy metal concentrations in the aerial tissues to that in roots following Equation (4).


(4) $$TF=\frac{C_{\mathrm{aerial}}}{C_{\mathrm{root}}}$$

Here, *C*_aerial_ denotes the heavy metal concentrations in aerial tissues (stems and leaves). A TF > 1 indicates efficient root-to-aerial part translocation of heavy metals, highlighting the plant’s capacity to redistribute heavy metals within its tissues (Ba *et al*. 2024).

### RNA extraction and preparation

*Erigeron canadensis* roots from the CK, LC, MC, and HC treatments (three biological replicates per treatment) were collected for RNA extraction after 3 months of growth. Total RNA was extracted using the Total RNA Extractor (Trizol) Kit (Sangon Biotech Co., Ltd., Shanghai, China) according to the manufacturer’s protocol. RNA integrity was evaluated with a 1.0% agarose gel, quality was assessed using a NanoPhotometer spectrophotometer (IMPLEN, Munich, Germany), and quantity was measured by a Qubit 2.0 Fluorometer (Invitrogen, Carlsbad, CA, USA). The total RNA was aliquoted for downstream RNA-seq and RT-qPCR analyses.

For RNA-seq analysis, the aliquoted RNA samples were subsequently sent to Sangon Biotech Co., Ltd. (Shanghai, China) for library preparation and sequencing. For RNA sample preparation, 1 μg RNA per sample served as the input material. Sequencing libraries were generated using the VAHTSTM mRNA-seq V2 Library Prep Kit for Illumina (Vazyme, Nanjing, China), following the manufacturer’s recommendations. cDNA fragments of 150–200 bp in length were purified using the AMPure XP system (Beckman Coulter, Beverly, MA, USA). PCR amplification was carried out using Phusion High–Fidelity DNA polymerase, universal PCR primers and an index (X) primer. The libraries were then quantified, pooled, and paired-end sequenced using NovaSeq sequencers (Illumina, San Diego, CA, USA).

### Transcriptome analysis

Raw reads were quality-checked with FastQC v0.11.2 (Andrews 2010) and filtered using Trimmomatic v0.36 (Bolger *et al*. 2014) according to the following steps: (i) removed adaptor sequences if present in the reads; (ii) trimmed low-quality bases from the 3′ to 5′ end of the reads (*Q* < 20); (iii) trimmed low-quality bases from the 5′ to 3′ end of the reads (*Q* < 20); (iv) employed a sliding window method to eliminate bases with a quality score of less than 20 at the tail end of the reads (with a window size of 5 bp); and (v) discarded reads shorter than 35 nucleotides and their corresponding paired reads.

The remaining clean reads were aligned to the *E. canadensis* (syn. *Conyza canadensis*) strain Cc 75 reference genome (RefSeq: GCF_010389155.1; assembly name: C_canadensis_v1; BioProject: PRJNA586872) (Laforest *et al*. 2020) using HISAT2 v2.0 under default parameters (Kim *et al*. 2015). These default settings comprised a 20 bp seed length (where a seed is a short subsequence of a read used as an anchor for initial alignment) with no mismatches within seeds, a maximum of two mismatches during read extension, a minimum intron length of 20 bp, a maximum intron length of 500 000 bp, and an unstranded spliced alignment mode. In addition, reads with up to five valid alignments were reported by default, and canonical GT/AG splice sites were employed as the default splicing signals. The transcriptome annotation was directly retrieved from the annotation file (GTF/GFF) provided with the reference genome. This annotation retrieval was performed for all transcripts across all samples and was completed prior to differential expression analysis.

Gene expression levels were then calculated using StringTie v1.3.3b (Pertea *et al*. 2015). After quantification, transcript expression levels were reported in transcripts per million (TPM) to standardize measurements across samples, accounting for gene lengths and sequencing depth. Principal component analysis (PCA) was then performed to assess overall similarity and variability among biological samples. Differentially expressed genes (DEGs) between CK and the three treatments were filtered using a *q*-value < 0.05 and |Fold change| ≥ 2 as criteria. The *q*-value was obtained by correcting the *P*-value according to Storey and Tibshirani (2003), and the fold change was calculated as the ratio of expression levels. All statistical analyses were performed using DESeq2 v1.26.0 in R (Love *et al*. 2014).

Predicted protein sequences were extracted from the reference genome and its annotation file using GffRead (Pertea and Pertea 2020) and subjected to functional annotation. Specifically, Gene Ontology (GO) terms were assigned using eggNOG-mapper v2 against the eggNOG database (Cantalapiedra *et al*. 2021). EuKaryotic Orthologous Groups (KOG) classifications were obtained by aligning predicted proteins to the NCBI KOG database with BLASTP v2.10.0 (Camacho *et al*. 2009). Kyoto Encyclopaedia of Genes and Genomes (KEGG) annotations were generated using KOBAS v3.0 against the KEGG GENES database (Bu *et al*. 2021). Functional enrichment analyses, including GO, KOG, and KEGG, were then performed using clusterProfiler v3.5 in R (Yu *et al*. 2012), with a significance threshold of *q*-value < 0.05. These analyses were conducted separately for each comparison (LC versus CK, MC versus CK, and HC versus CK), using all identified DEGs from the respective comparison as the query gene list. The reference background (gene universe) was defined as all genes in the *E. canadensis* reference genome that were annotated in the corresponding database (GO, KOG, or KEGG).

### Real-time quantitative PCR (RT-qPCR)

As DEGs from the LC treatment were not significantly enriched in any KEGG pathway (*q*-value > 0.05), aliquots of the total RNA from the CK, MC, and HC treatments were used to validate transcriptome data by RT-qPCR in three biological replicates. Four DEGs related to membrane transporters, responsive kinases, and ROS, namely ABC transporter C family member 4 (*ABCC4*, LOC122581240), serine/threonine-protein kinase CCR4 (*STK-CCR4*, LOC122607819), CBL-interacting protein kinase 2 (*CIPK2*, LOC122582470), and glutathione S-transferase U19 (*GSTU19*, LOC122592435), were chosen. Primers of the four DEGs are listed in Supplementary Table S1. Ubiquitin-conjugating enzyme E2-2 (UBE22, LOC122603355) was selected as the internal reference gene (Supplementary Table S1). All primers were designed using Primer Premier 5.0. Each RT-qPCR reaction was performed by mixing 10 μl SYBR Green qPCR Master Mix (BBI, Sangon Biotech, Shanghai, China), 0.4 μl of each primer, 2 μl of the synthesized cDNA, and sterilized water for a total volume of 20 μl. The reactions commenced with an initial denaturation step at 95°C for 3 min, followed by 45 cycles of denaturation at 95°C for 15 s, annealing at 60°C for 30 s, and extension at 72°C for 30 s. The relative gene expression levels were calculated using the 2^−ΔΔCT^ method (Rao *et al*. 2013).

### Heterologous expression in yeast

The full-length coding sequences (CDS) of *NRAMP3*, *ZntB*, and *CNGC2* were inserted into the polyclonal site of the pYES2-NTB vector to construct recombinant plasmids driven by the GAL1 promoter. These three plasmids were transferred into the Cd/Pb-sensitive yeast (*Saccharomyces cerevisiae*) mutant Δ*ycf1*, yielding recombinant strains Δ*ycf1* + *NRAMP3*, Δ*ycf1* + *ZntB*, and Δ*ycf1* + *CNGC2*. Wild-type BY4741 and Δ*ycf1* mutant transformed with empty vector (WT + EV and Δ*ycf1* + EV) served as positive and negative controls, respectively. Prior to phenotypic analysis, RT-qPCR was carried out to confirm the successful expression of *NRAMP3*, *ZntB*, and *CNGC2* in the recombinant yeast strains. Recombinant strains were cultured on SD-U medium supplemented with 0 μM, 60 μM Cd(CH_3_CO_2_)_2_·2H_2_O, or 60 μM Pb(CH_3_CO_2_)_2_·3H_2_O, with yeast suspensions serially diluted to 10^0^, 10^–1^, 10^–2^, and 10^–3^. Plates were incubated upside down at 30°C for 3 days. Growth curves of yeast cells expressing *NRAMP3*, *ZntB*, and *CNGC2* in liquid SD-U medium containing 60 μM Cd(CH_3_CO_2_)_2_·2H_2_O or 60 μM Pb(CH_3_CO_2_)_2_·3H_2_O were determined by measuring OD_600_ values at 0, 4, 8, 12, 24, 36, 48, 60, and 72 h. After 72 h incubation, yeast cells were harvested for Cd and Pb quantification by ICP–MS. All experiments were performed in three biological replicates.

### Statistical analyses

Normality and homogeneity of variances were assessed using the Shapiro–Wilk and the Brown–Forsythe tests, respectively. Datasets meeting both assumptions were assessed using one-way analysis of variance followed by Tukey’s post-hoc test. Since stem biomass, Cd accumulation in stems, and BCF_Pb_ in leaves for inter-treatment comparisons, as well as BCF_Cd_ under LC treatment and BCF_Pb_ under HC treatment for inter-organ comparisons, were not normally distributed, the non-parametric Kruskal–Wallis H test followed by multiple comparisons was used. Data were obtained from three independent replicates. The significance level was set at *P* < .05. All statistical analyses were performed using SPSS 22.0 software (IBM, Armonk, NY, USA).

## Results

### Impact of heavy metals on *Erigeron canadensis* biomass

No significant differences were observed in stem biomass (*H* = .292, *P* = .514) and leaf biomass (*F* = 3.950, *P* = .053) of *E. canadensis* across all treatments (Fig. 1). However, root biomass was significantly affected by the treatment (*F* = 12.262, *P* = .002), showing a significant reduction under the HC treatment relative to CK (*P* = .047) (Fig. 1).

**Figure 1:**
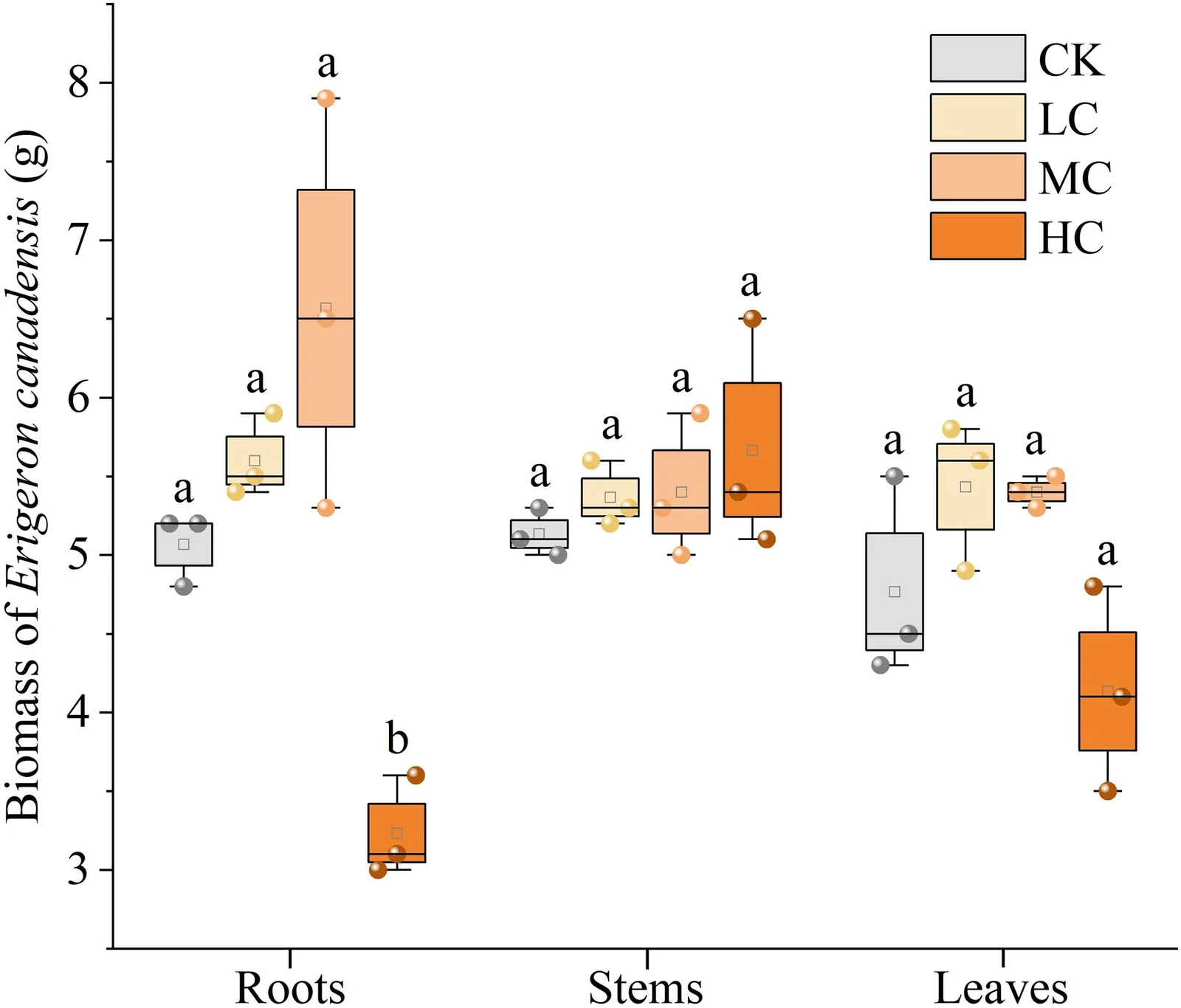
Root, stem, and leaf biomass of *Erigeron canadensis* under combined cd and pb treatments. CK, LC, MC, and HC represent the control (no Cd or Pb addition), low concentration (1 mg/kg Cd and 90 mg/kg Pb), moderate concentration (2 mg/kg Cd and 180 mg/kg Pb), and high concentration (4 mg/kg Cd and 360 mg/kg Pb) treatments, respectively. Data are presented as boxplots with individual data points (*n* = 3). For each organ, multiple comparisons of biomass among treatments were performed. Different lowercase letters above boxplots indicate significant differences among treatments (*P* < .05).

### Heavy metal accumulation, bioconcentration, and translocation in *Erigeron canadensis*

For inter-treatment comparisons within each organ, Cd and Pb accumulation differed significantly among treatments in roots, stems, and leaves (Fig. 2a and b and Table 1). Post-hoc analysis showed that LC treatment increased Cd in roots (*P* = .003) and leaves (*P* = .011) and Pb in roots (*P* = .007) and stems (*P* = .017), whereas both MC and HC treatments significantly elevated Cd and Pb in all three organs (all *P* < .05). BCF values for Cd differed among treatments in all three organs, whereas BCF values for Pb differed significantly in roots and leaves but not in stems (Fig. 2c and d and Table 1). Post-hoc analysis revealed that LC treatment significantly decreased the BCF_stem_ for Cd (*P* = .001) but significantly increased the BCF_root_ for Pb (*P* = .003). Both MC and HC treatments significantly reduced the BCF_root_, BCF_stem,_ and BCF_leaf_ for Cd (all *P* < .05). In addition, the BCF_leaf_ for Pb was also significantly decreased under MC and HC treatments (*P* = .004 and *P* = .003, respectively).

**Figure 2:**
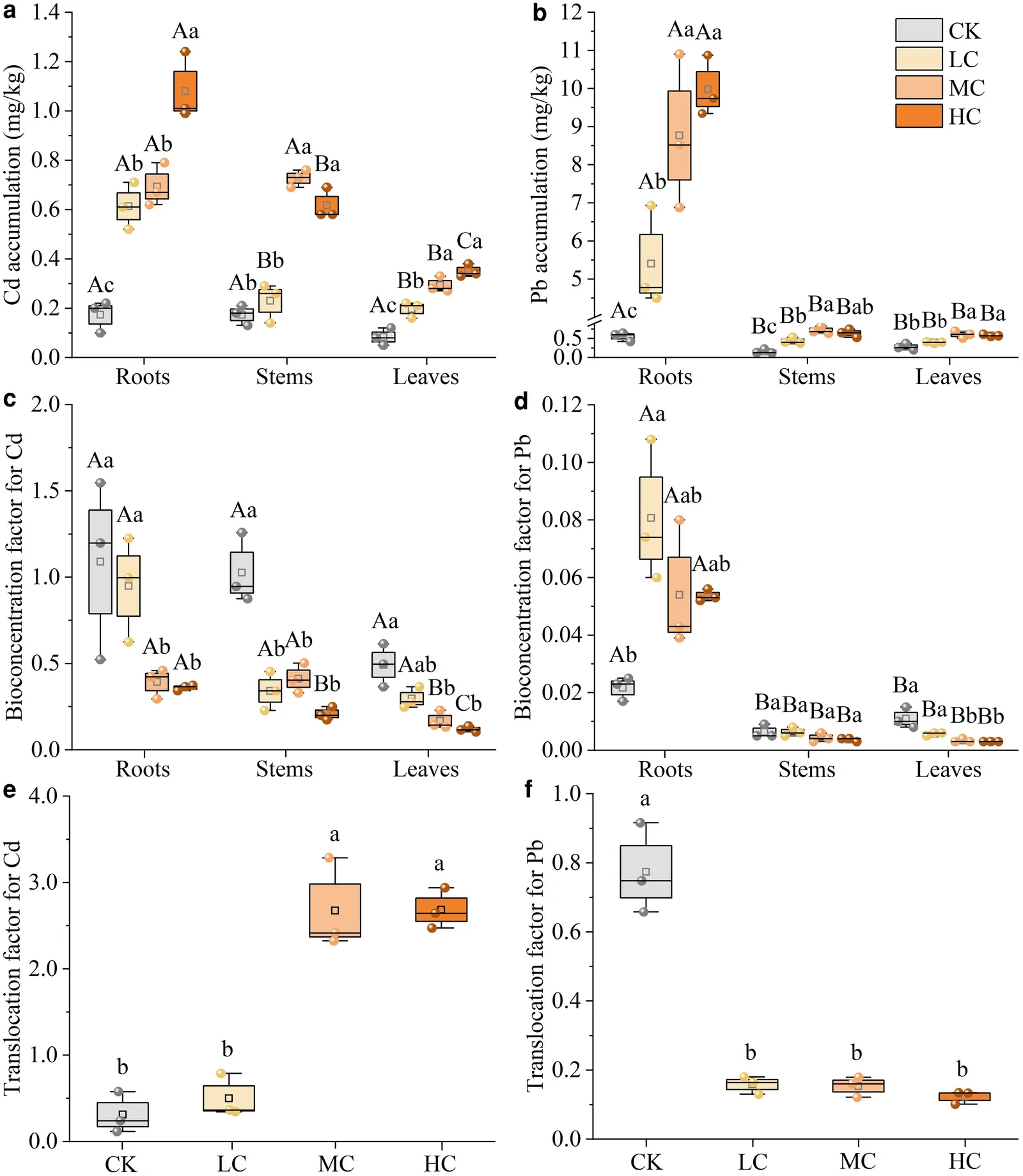
Heavy metal accumulation, bioconcentration, and translocation in *Erigeron canadensis*. CK, LC, MC, and HC represent the control (no Cd or Pb addition), low concentration (1 mg/kg Cd and 90 mg/kg Pb), moderate concentration (2 mg/kg Cd and 180 mg/kg Pb), and high concentration (4 mg/kg Cd and 360 mg/kg Pb) treatments, respectively. (a–b) Cd and Pb accumulation in *E. canadensis* organs, (c–d) bioconcentration factor for Cd and Pb, and (e–f) translocation factor for Cd and Pb. Data are presented as boxplots with individual data points (*n* = 3). Inter-treatment comparisons within each plant organ and inter-organ comparisons within each treatment were both performed for Cd and Pb accumulation and bioconcentration. Different lowercase letters above boxplots indicate significant differences in the inter-treatment comparisons within each organ, and different uppercase letters above boxplots indicate significant differences in the inter-organ comparisons within each treatment (*P* < .05).

**Table 1:** Inter-treatment comparisons of heavy metal accumulation and bioconcentration within each organ.

Traits	Roots		Stems		Leaves	
	*F/H*	*P*	*F/H*	*P*	*F/H*	*P*
Cd accumulation	41.390	.001	9.515^a^	.023	41.031	.001
Pb accumulation	32.820	.001	24.261	<.001	17.052	.001
BCF_Cd_	4.574	.038	25.024	<.001	14.844	.001
BCF_Pb_	6.058	.019	2.461	.137	10.385^a^	.016

*F/H* denote the *F* value from one-way ANOVA or *H* value from the Kruskal–Wallis test; BCF_Cd_ represents the bioconcentration factor for Cd; BCF_Pb_ represents the bioconcentration factor for Pb.

^a^Indicates datasets analysed by the non-parametric Kruskal–Wallis *H* test.

For inter-organ comparisons within each treatment, Cd and Pb accumulations differed significantly among organs under LC, MC, and HC treatments (Fig. 2a and b and Table 2). Subsequent post-hoc comparisons confirmed that roots accumulated significantly higher Cd and Pb concentrations than stems and leaves in almost all treatment groups (all *P* < .05), with the exception that under MC treatment, Cd levels in roots and stems did not differ significantly. BCF values varied among organs under LC, MC, and HC treatments for both Cd and Pb (Fig. 2c and d and Table 2). Post-hoc analysis revealed that BCF_root_ was higher than BCF_stem_ and BCF_leaf_ under MC and HC treatments for Cd and under LC, MC, and HC treatments for Pb (all *P* < .05).

**Table 2.** Inter-organ comparisons of heavy metal accumulation and bioconcentration within each treatment.

Traits	CK		LC		MC		HC	
	*F/H*	*P*	*F/H*	*P*	*F/H*	*P*	*F/H*	*P*
Cd accumulation	3.471	.100	29.481	.001	52.862	<.001	51.097	<.001
Pb accumulation	15.390	.004	42.066	<.001	48.090	<.001	41.093	<.001
BCF_Cd_	2.961	.127	5.422^a^	.066	9.327	.014	65.927	<.001
BCF_Pb_	15.589	.004	27.331	.001	14.703	.005	6.826^a^	.033

*F/H* denote the *F* value from one-way ANOVA or *H* value from the Kruskal−Wallis test; BCF_Cd_ represents the bioconcentration factor for Cd; BCF_Pb_ represents the bioconcentration factor for Pb.

^a^Indicates datasets analysed by the non-parametric Kruskal−Wallis *H* test.

Furthermore, TF values for Cd and Pb differed significantly among treatments (*F* = 45.474, *P* < .001 for Cd; *F* = 62.603, *P* < .001 for Pb) (Fig. 2e and f). LC treatment had no significant effect on TF for Cd (*P* = .902) but significantly decreased TF for Pb (*P* < .001). Both MC and HC treatments significantly increased TFs for Cd (both *P* < .001) and significantly decreased TFs for Pb (both *P* < .001). Notably, TF > 1 for Cd was observed under MC and HC treatments, indicating efficient Cd translocation from roots to aerial tissues in *E. canadensis* (Fig. 2e and f).

### Transcriptome assembly for *Erigeron canadensis* roots

A total of 89.79 Gb of clean data (624 659 034 clean reads) was generated from *E. canadensis* roots across all four treatments (CK, LC, MC, and HC). The average read length ranged from 143 to 145 base pairs (bp), with the longest read length of 150 bp for each sample (Supplementary Table S2). Reads with a base quality score above Q30 accounted for 94.7–97.3%, and the GC content ranged from 42.7% to 49.3% (Supplementary Table S2).

PCA showed tight clustering of biological replicates within each of the four groups, confirming high reproducibility of sample preparation and sequencing (Fig. 3). Based on these reliable transcriptomic data, a total of 635 (649), 2571 (2680), and 2909 (2996) DEGs (transcripts) were identified in the LC, MC, and HC treatments compared with CK, respectively (Fig. 4; Supplementary Tables S3–S5). Specifically, for LC versus CK, MC versus CK, and HC versus CK, the numbers of up-regulated DEGs (transcripts) were 143 (143), 1079 (1116), and 1184 (1201), respectively; down-regulated DEGs (transcripts) were 489 (500), 1477 (1534), and 1710 (1765), respectively; and bidirectional DEGs (transcripts) were three (six), 15 (30), and 15 (30), respectively.

**Figure 3:**
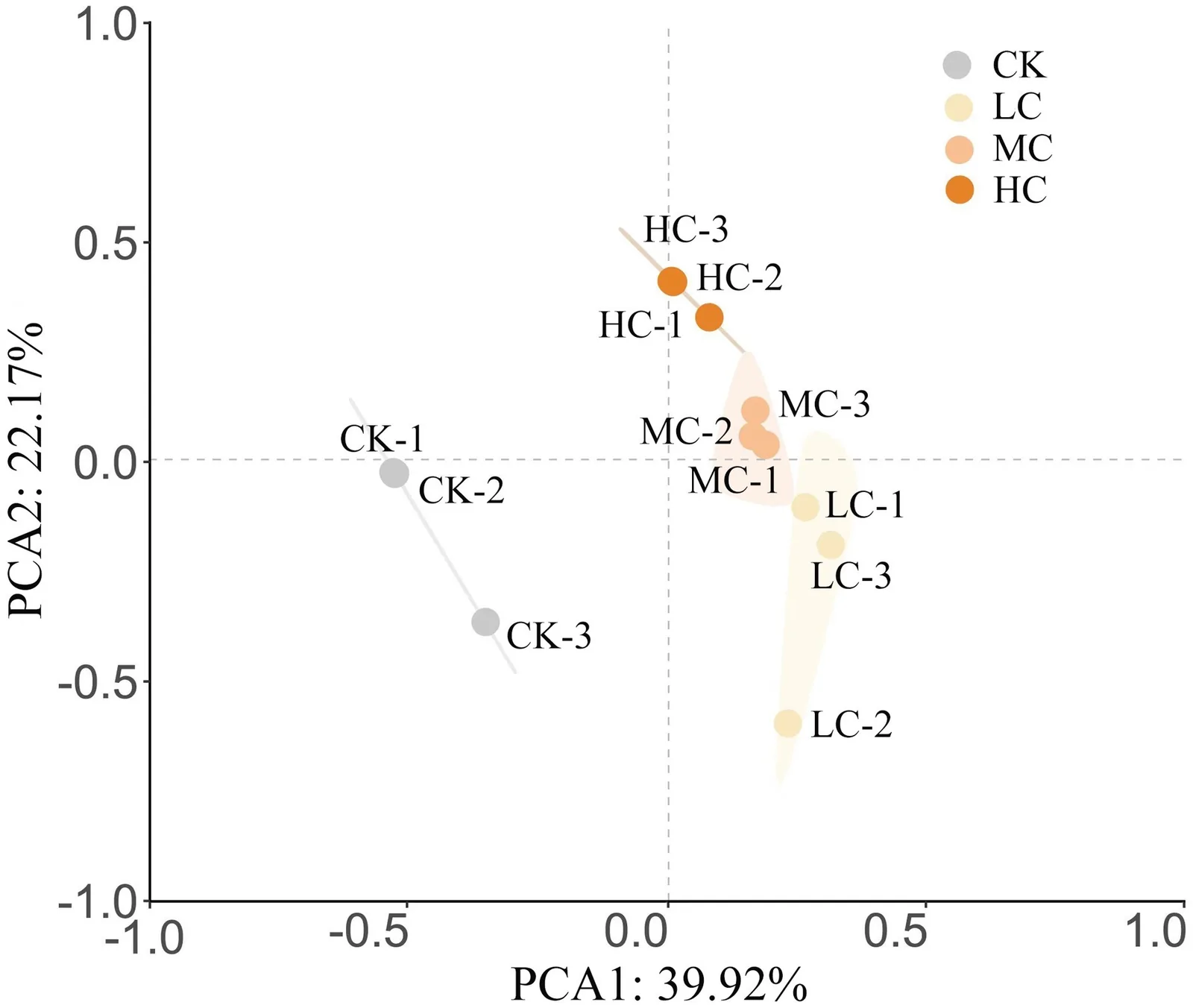
Principal component analysis (PCA) of biological replicates based on normalized expression levels of all expressed genes in *Erigeron canadensis* roots under combined cd and pb treatments. Each point represents one RNA-Seq sample (*n* = 3), and shaded areas represent 95% confidence ellipses. CK, LC, MC, and HC represent the control (no Cd or Pb addition), low concentration (1 mg/kg Cd and 90 mg/kg Pb), moderate concentration (2 mg/kg Cd and 180 mg/kg Pb), and high concentration (4 mg/kg Cd and 360 mg/kg Pb) treatments, respectively. Numbers following CK, LC, MC, and HC indicate the replicate number.

**Figure 4:**
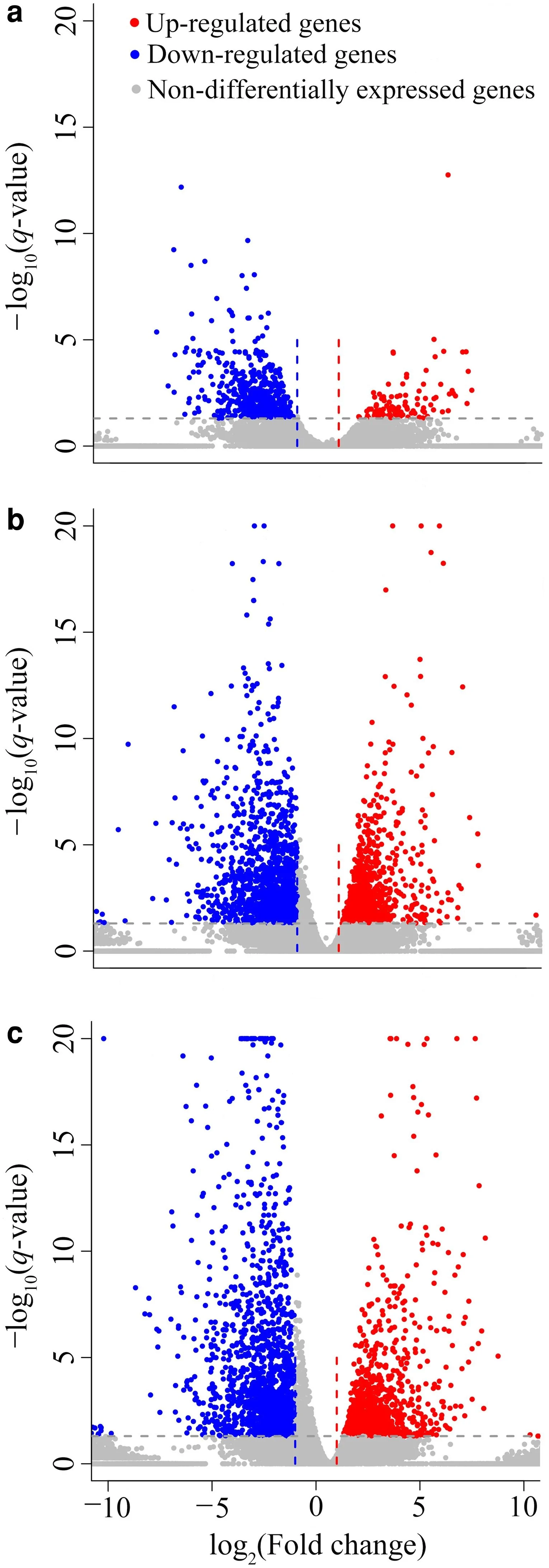
Volcano plots of differentially expressed genes in *Erigeron canadensis* roots (a) LC versus CK; (b) MC versus CK; (c) HC versus CK. Dots above the horizontal grey dashed line represent significantly differentially expressed genes (DEGs, *q*-value < 0.05). Red dots indicate significantly up-regulated genes, blue dots indicate significantly down-regulated genes, and grey dots indicate non-differentially expressed genes. The vertical blue dashed line indicates *FC* = −2, the vertical red dashed line indicates *FC* = 2, and the horizontal grey dashed line indicates the level at which *q*-value = 0.05. Fold change in the expression of each gene was calculated using the mean of three biological replicates (*n* = 3). LC, MC, and HC represent the low concentration (1 mg/kg Cd and 90 mg/kg Pb), moderate concentration (2 mg/kg Cd and 180 mg/kg Pb), and high concentration (4 mg/kg Cd and 360 mg/kg Pb) treatments, respectively.

### Functional annotation and enrichment analysis

GO enrichment analysis revealed 24, 21, and 48 significantly enriched functional terms under LC, MC, and HC treatments, respectively (all *q*-values < 0.05). Specifically, the top three enriched GO terms were ‘response to stimulus’, ‘response to stress’, and ‘response to chemical’ for LC treatment; ‘cytoplasmic part’, ‘response to stimulus’, and ‘response to stress’ for MC treatment; and ‘response to stimulus’, ‘response to chemical’, and ‘response to stress’ for HC treatment (Fig. 5a).

**Figure 5:**
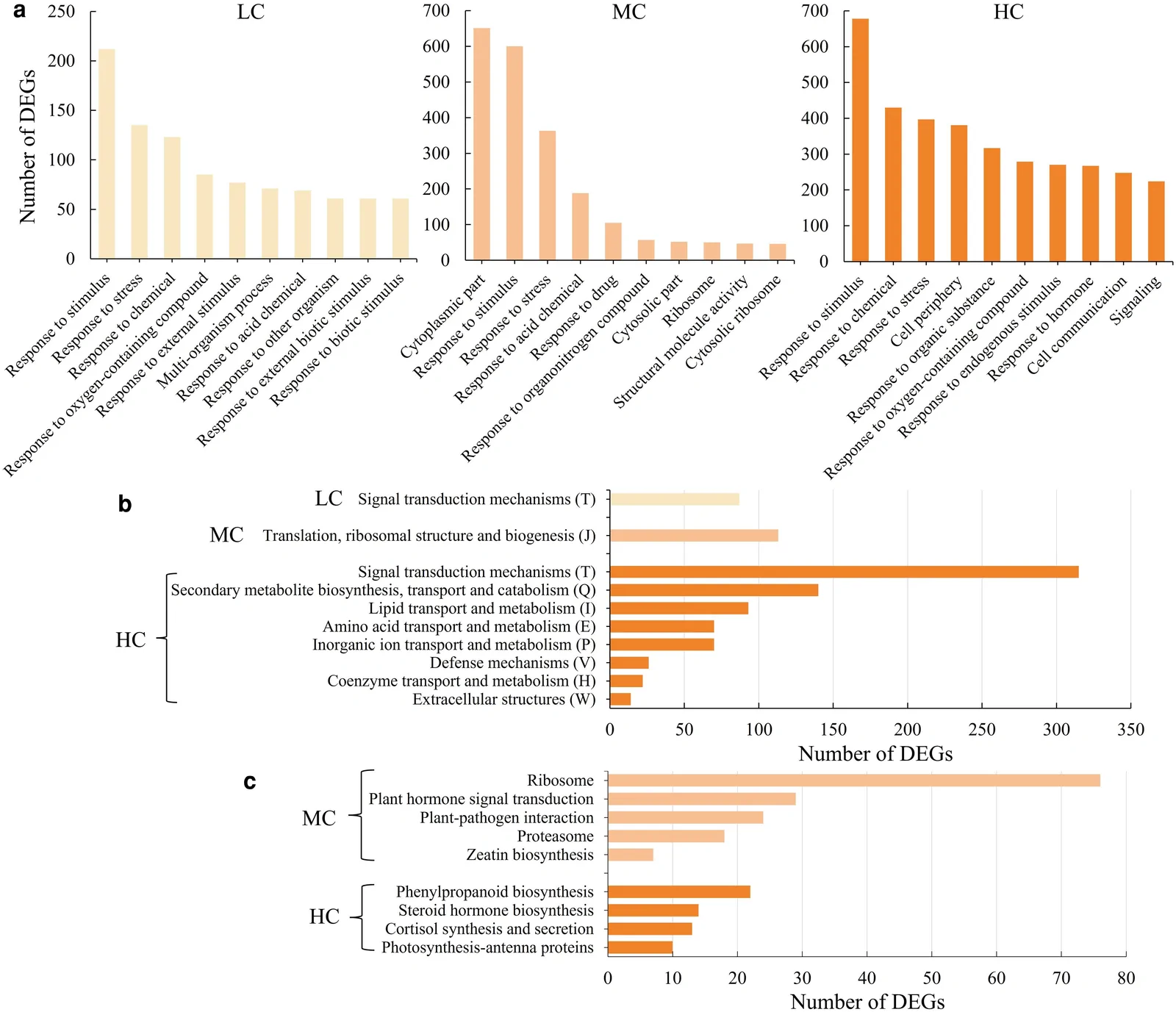
Functional annotation and enrichment analyses in *Erigeron canadensis* roots. LC, MC, and HC represent low concentration (1 mg/kg Cd and 90 mg/kg Pb), moderate concentration (2 mg/kg Cd and 180 mg/kg Pb), and high concentration (4 mg/kg Cd and 360 mg/kg Pb) treatments, respectively. (a) GO enrichment analysis of differentially expressed genes (DEGs) in LC, MC, and HC treatments. The top ten GO terms with the highest number of DEGs are shown for each treatment (*q*-values < 0.05). (b) KOG classification of DEGs in LC, MC, and HC treatments (*q*-values < 0.05). (c) KEGG pathway analysis of DEGs in MC and HC treatments (*q*-values < 0.05).

KOG enrichment analysis revealed that signal transduction mechanisms were the sole significantly enriched category under LC treatment (*q*-value = 0.008). For MC treatment, translation, ribosomal structure and biogenesis were the only significantly enriched category (*q*-value < 0.001); under HC treatment, eight categories were significantly enriched, including signal transduction mechanisms, secondary metabolite biosynthesis, transport and catabolism, inorganic ion transport and metabolism, and amino acid transport and metabolism (all *q*-values < 0.050; Fig. 5b).

KEGG enrichment analysis mapped 213 DEGs (217 transcripts) to nine KEGG pathways. Notably, DEGs from the LC treatment showed no significant enrichment in any KEGG pathway (*q*-value > 0.05). In the MC treatment group, the significantly enriched ribosome pathway accounted for the highest proportion of DEGs (*q*-value < 0.001) (Fig. 5c; Supplementary Figure S1). For the HC treatment, the significantly enriched phenylpropanoid biosynthesis was the most highly represented pathway, involving 22 DEGs (*q*-value = 0.010) (Fig. 5c; Supplementary Figure S2). Based on the KEGG enrichment results, subsequent analyses focused on the functional locations of DEGs from the MC and HC treatment groups.

Collectively, GO, KOG, and KEGG enrichment analyses indicated that the significantly enriched functional terms and pathways were closely associated with substance transport, signal transduction, stress response, transcriptional regulation, and ribosome function.

### Functional locations of DEGs in *Erigeron canadensis* roots after heavy metal exposure

For MC versus CK and HC versus CK, 49 (15 up- and 34 down-regulated) and 48 (16 up- and 32 down-regulated) DEGs were annotated as transport genes, respectively (Supplementary Table S6). Under MC treatment, natural resistance-associated macrophage protein 3 (*NRAMP3*), zinc transporter 29 (*ZTP29*), and cyclic nucleotide-gated ion channel 2 (*CNGC2*) were up-regulated. Conversely, *ZnT1*, *ZnT5*, vacuolar iron transporter 1 (*VIT1*), *VIT4*, and ATP-binding cassette (ABC) transporter family members were down-regulated. Under HC treatment, *ZTP29*, *ZntB*, and *CNGC2* were up-regulated, while *VIT4* and ABC transporters were down-regulated. Nutrient-related transporters were also differentially expressed. Lysine histidine transporter 8 (*LHT8*) was down-regulated, whereas sugar transporters and the majority of amino acid transporters (AATs) were up-regulated under both treatments. Auxin transporter-like protein *AUX2* and IAA-alanine resistance protein *IAR1* were up-regulated under MC treatment, while only *AUX2* was found and was down-regulated under HC treatment. For HC versus MC, 17 DEGs (ten up- and seven down-regulated) were annotated as membrane transport genes. *ZnT1* and *ZnT5* were down-regulated, while *ZntB*, *CNGC2*, and *CNGC14* were up-regulated. Notably, fewer sugar transporters were observed under HC than under MC treatment. Functional enrichment analysis revealed that four transport-related DEGs were enriched in the ‘response to stress’ term under MC treatment, and four DEGs in this term under HC treatment. Among these DEGs, *NRAMP3* and *CNGC2* were assigned to this enriched GO term under MC treatment, whereas *ABCC4* and *CNGC2* were assigned to the same term under HC treatment.

A total of 77 (15 up- and 62 down-regulated) and 103 (29 up- and 74 down-regulated) signalling-related DEGs (mainly protein kinase genes) were identified in *E. canadensis* roots for MC versus CK and HC versus CK, respectively (Supplementary Table S7). The majority of responsive kinases belonged to the STK family. Specifically, DEGs encoding leucine-rich repeat receptor-like serine/threonine-protein kinases (LRR-STKs), leucine-rich repeat receptor-like protein kinases (LRR-RLKs), calcium-dependent protein kinases (CDPKs), CIPKs, mitogen-activated protein kinases (MAPKs), and wall-associated receptor kinases (WAKs) were predominantly down-regulated under both treatments. In contrast, almost all G-type lectin S-receptor-like serine/threonine-protein kinases (GsSRKs) were up-regulated under HC treatment. For HC versus MC, 31 DEGs (22 up- and nine down-regulated) were associated with signal transduction. Functional enrichment analysis revealed that 14 signalling-related DEGs under MC treatment were enriched in the ‘response to stress’ term, while under HC treatment, 24 and 77 DEGs were enriched in the ‘response to stress’ term and the signal transduction mechanisms, respectively.

The involvement of ROS-related genes in stress responses was also studied. A total of 58 (28 up- and 30 down-regulated) and 82 (31 up- and 51 down-regulated) ROS-related DEGs were identified for MC versus CK and HC versus CK, respectively (Supplementary Table S8). These DEGs encoded reductase, oxidoreductase, dioxygenase, thioredoxin (Trx), peroxidase (PER), adenylyl-sulphate reductase, glutathione peroxidase (GSH-Px), GST, methyltransferase, and catalase. Reductase genes accounted for the highest proportion, with over half significantly up-regulated under both treatments. For HC versus MC, 30 ROS-related DEGs (seven up- and 23 down-regulated) were observed. Notably, GSH-Px, GST, and adenylyl-sulphate reductase were up-regulated under MC treatment, with more such genes detected under MC treatment than under HC treatment. Moreover, 16 and 15 ROS-related DEGs were enriched in the ‘response to stress’ term under MC and HC treatments, respectively. Additionally, seven peroxidase genes (EC number 1.11.1.7; Supplementary Figure S2) were involved in the phenylpropanoid biosynthesis pathway under HC treatment.

We identified 174 (32 up- and 142 down-regulated) and 173 (38 up- and 135 down-regulated) DEGs annotated as transcription factors (TFs) for MC versus CK and HC versus CK, respectively (Supplementary Table S9). These genes, implicated in heavy metal stress responses, predominantly belonged to zinc-finger protein (ZFP), APETALA2/ethylene-responsive factor (AP2/ERF), myeloblastosis (MYB), WRKY, auxin response factor (ARF), and basic helix-loop-helix (bHLH) families. ZFP genes accounted for the highest proportion under both treatments. For HC versus MC, 45 DEGs (10 up- and 35 down-regulated) were assigned to transcription factor categories. MYB genes exhibited both up- and down-regulation, with more DEGs observed under MC treatment than under HC treatment, whereas *WRKY75* was uniquely up-regulated under HC treatment. Furthermore, 44 and 47 transcription factor-encoding genes were enriched in the ‘response to stress’ term under MC and HC treatments, respectively.

A total of 73 (72 up- and one down-regulated) and 56 (55 up- and one down-regulated) ribosome-related DEGs were identified for MC versus CK and HC versus CK, respectively (Supplementary Table S10). These DEGs mainly encoded 40S, 50S, and 60S ribosomal proteins, and also included three types of RNA polymerases (polymerases II, III, and IV). For HC versus MC, all ribosomal proteins and RNA polymerases were down-regulated. Under MC treatment, 65 DEGs were enriched in the ribosome pathway (Supplementary Figure S1).

### Gene expression analysis by RT-qPCR

Significant differences were found in *ABCC4* (*F* = 16.748, *P* = .004), *STK-CCR4* (*F* = 7.224, *P* = .025), *CIPK2* (*F* = 10.898, *P* = .010), and *GSTU19* (*F* = 53.703, *P* < .001) (Fig. 6). Under MC and HC treatments, *ABCC4* (*P* = .028 and .003), *STK-CCR4* (*P* = .046 and .033), and *CIPK2* (*P* = .019 and .014) were significantly down-regulated in *E. canadensis* roots, whereas *GSTU19* (*P* < .001 and .019) was up-regulated (Fig. 6). These expression trends matched the transcriptomic data, validating the reliability of the RNA-seq analysis.

**Figure 6:**
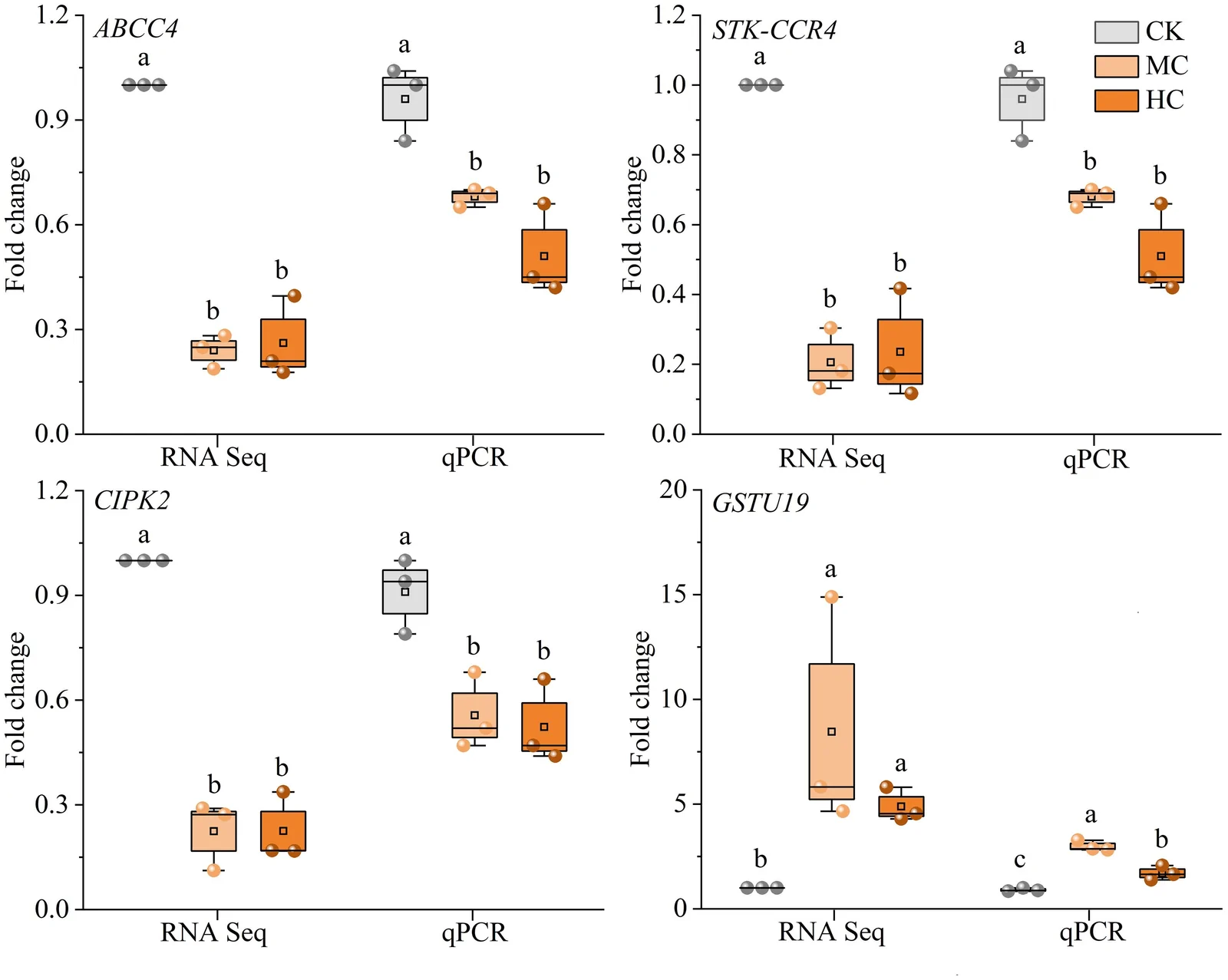
Validation of gene expression by RT-qPCR. CK, MC, and HC represent the control (no Cd or Pb addition), moderate concentration (2 mg/kg Cd and 180 mg/kg Pb), and high concentration (4 mg/kg Cd and 360 mg/kg Pb) treatments, respectively. Fold change in expression was calculated relative to CK. Data are presented as boxplots with individual data points (*n* = 3). *ABCC4*: ABC transporter C family member 4; *STK-CCR4*: serine/threonine-protein kinase protein CCR4; *CIPK2*: CBL-interacting protein kinase 2; *GSTU19*: glutathione S-transferase U19. Different lowercase letters above boxplots indicate a significant difference (*P* < .05).

### Heterologous expression in yeast validated the roles of *NRAMP3*, *ZntB*, and *CNGC2* in Cd and Pb accumulation

WT + EV, Δ*ycf1* + EV, Δ*ycf1* + *NRAMP3*, Δ*ycf1* + *ZntB*, and Δ*ycf1* + *CNGC2* yeast strains exhibited similar growth in media without Cd or Pb. Compared with WT + EV and Δ*ycf1* + EV, heterologous expression of *NRAMP3, ZntB,* and *CNGC2* rescued yeast growth in the medium supplemented with 60 μM Cd(CH_3_CO_2_)_2_·2H_2_O or 60 μM Pb(CH_3_CO_2_)_2_·3H_2_O (Fig. 7a), consistent with the growth curve assays (Fig. 7b). Subsequently, intracellular Cd and Pb concentrations in these strains were quantified. Significant differences were found among the five strains under both Cd (*F* = 1916.359, *P* < .001) and Pb (*F* = 407.555, *P* < .001) treatments. The Cd concentrations in Δ*ycf1* + *NRAMP3*, Δ*ycf1* + *ZntB*, and Δ*ycf1* + *CNGC2* strains were significantly higher than that in Δycf1 + EV mutant (all *P* < .001). Similarly, Pb concentrations in Δ*ycf1* + *NRAMP3* and Δ*ycf1* + *CNGC2* strains were higher than that in Δ*ycf1* + EV mutant (*P* < .001 and *P* = .035). WT + EV strains accumulated significantly more Cd and Pb than all other strains (all *P* < .001; Fig. 7c).

**Figure 7:**
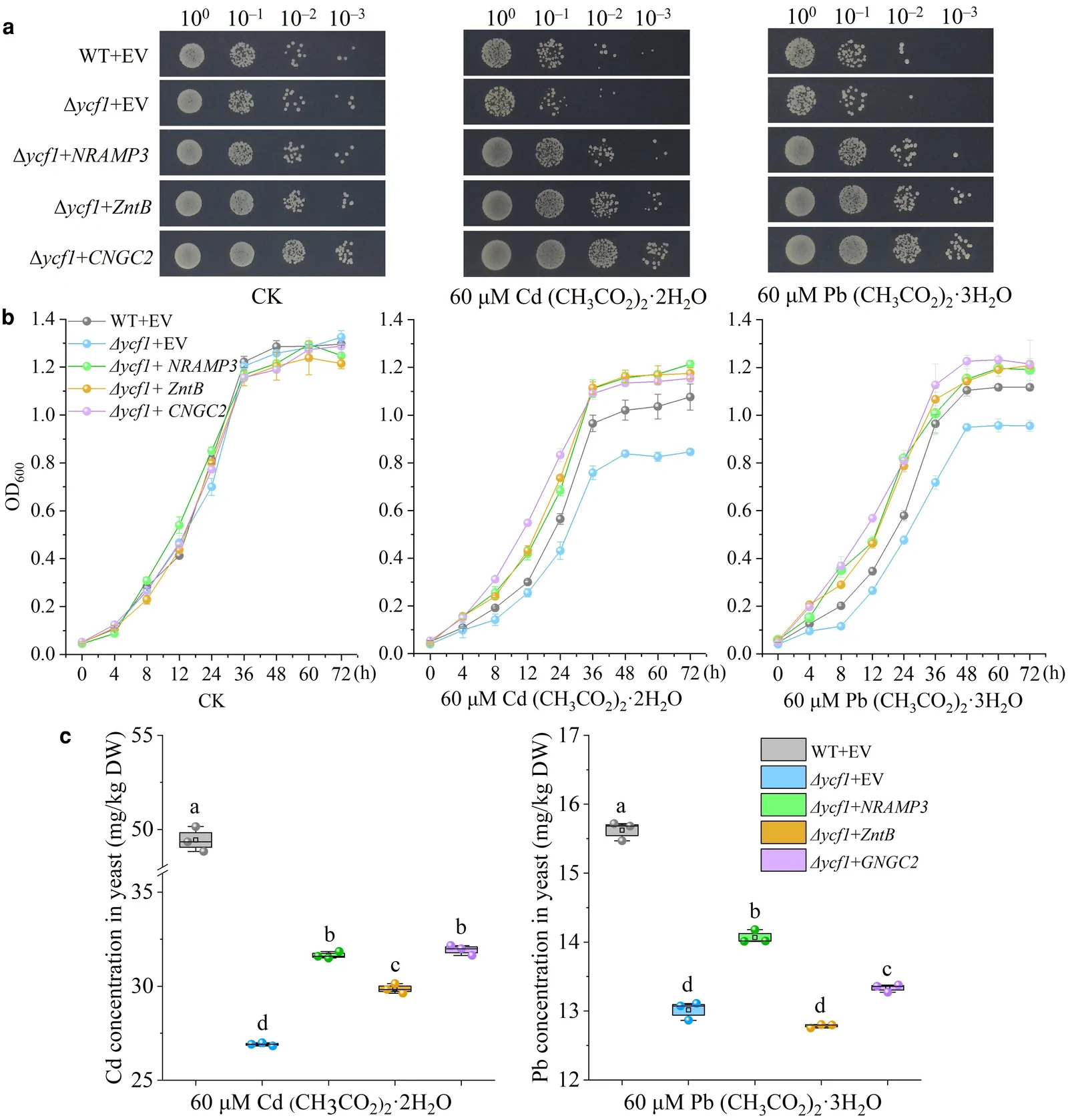
Functional analysis of *NRAMP3*, *ZntB*, and *CNGC2* in yeast strains. (a) Heterologous expression of *NRAMP3*, *ZntB*, and *CNGC2* rescued the growth of *Saccharomyces cerevisiae* Δ*ycf1* mutants. (b) Growth curves of yeast strains under CK, Cd and Pb treatments. (c) Intracellular Cd and Pb concentrations in different yeast strains. WT + EV: wild-type BY4741 transformed with empty vector; Δ*ycf1* + EV: Δ*ycf1* mutant transformed with empty vector; *NRAMP3*: natural resistance-associated macrophage protein 3; *ZntB*: Zinc transport protein *ZntB*; *CNGC2*: cyclic nucleotide-gated ion channel 2. Different lowercase letters above boxplots indicate a significant difference (*P* < .05).

## Discussion

Plants inevitably take up heavy metals via roots due to the non-selective absorption of soil nutrients, which adversely affects growth and development (Checkai *et al*. 1987). While Cu, Zn, Fe, and Ni are essential micronutrients for plants, Cd, Pb, and Hg are non-essential and highly toxic elements (Paulose *et al*. 2009). Once these toxic heavy metals enter plant organs, they trigger various physiological responses, including altered seedling growth, biomass production, photosynthetic pigment synthesis, and nutrient uptake (Shi and Cai 2009). In the present study, growth of *E. canadensis* was not significantly affected under LC and MC treatments, indicating that this species exhibits considerable tolerance to heavy metals, which aligns with findings in other metal-tolerant plant species (Żurek *et al*. 2022, Ariyachandra *et al*. 2023). The significant reduction in root biomass under HC treatment (without a concurrent effect on aerial parts) can be explained by the fact that Cd and Pb predominantly accumulated in roots, with lower concentrations in aerial parts (Fig. 2a and b). Since roots were the primary site of metal uptake and direct exposure, the higher heavy metal levels in roots likely caused local toxicity, inhibiting root growth. In contrast, the lower metal concentrations in aerial parts were probably insufficient to suppress their growth. These findings align with prior studies showing that excessive heavy metal exposure impairs plant growth and development (Zhou *et al*. 2015, Yeboah *et al*. 2020).

A key mechanism underlying plant adaptation to heavy metals is exclusion, which involves sequestering metal ions in specific tissues to mitigate toxic effects (Ye *et al*. 2018). Through this strategy, plants predominantly retain accumulated heavy metals in roots, minimizing excessive translocation to aerial organs (Kushwaha *et al*. 2016). This strategy has been observed in *Brassica juncea* (L.) Czern., *Salix viminalis* L., *Solanum nigrum* L., and *Ricinus communis* L., with higher heavy metal accumulation in roots than in aboveground tissues (Fidalgo *et al*. 2013, Tőzsér *et al*. 2017, Yeboah *et al*. 2020). Notably, the TF for Pb was < 1, which supports the previous report that a significant portion of Pb accumulated in *E. canadensis* roots, with limited translocation to aerial parts (Li *et al*. 2016). In contrast, another adaptive strategy is accumulation and translocation, wherein plant tissues actively accumulate heavy metals and translocate them to aerial organs (Kushwaha *et al*. 2016). The TF for Cd > 1 in the present study implied that *E. canadensis* employed this strategy to actively accumulate Cd in roots and translocate it to stems and leaves in response to combined Cd and Pb stress.

Transmembrane metal transporters play crucial roles in heavy metal absorption, transport, and detoxification (Xu *et al*. 2020). Typically, Cd is taken up, translocated, and accumulated via transporters responsible for essential metal ions such as Zn and Fe (Clemens 2006). Although ZnT genes mainly mediate Zn transport, some ZnTs are involved in Cd transport in *Arabidopsis thaliana* (L.) Heynh. and *Thlaspi caerulescens* J. & C. Presl (Grotz *et al*. 1998). Previous studies have demonstrated that *ZntB* mediates Cd^2+^ efflux (Chaoprasid *et al*. 2015). In the present study, heterologous expression of *ZntB* in yeast verified its role in regulating Cd uptake and tolerance (Fig. 7c). Similarly, NRAMP family transporters facilitate the absorption and translocation of various metal ions including Fe, Cd, Zn, Cu, and Pb in plants (Tian *et al*. 2021, Yuan *et al*. 2025). *NRAMP3* has been shown to transport Cd^2+^ into the cytoplasm under low-metal conditions and facilitate Cd^2+^ efflux from vacuoles during detoxification (Thomine *et al*. 2000, Wei *et al*. 2009). In this study, *NRAMP3* was up-regulated under combined Cd and Pb stress, consistent with observations in *Medicago sativa* L. and *Brassica napus* L. (Zhang *et al*. 2018). In contrast, the mechanisms governing Pb uptake and translocation are not well understood, though CNGCs are implicated in Pb uptake by arabidopsis roots (Sunkar *et al*. 2000, Moon *et al*. 2019). Heterologous overexpression of *NRAMP3* and *CNGC2* in the Δ*ycf1* yeast mutant improved yeast tolerance to Cd and Pb stress, while quantitative analysis of Cd and Pb accumulation in these yeast strains confirmed that the functional characteristics of *NRAMP3* and *CNGC2* are consistent with heavy metal accumulation in *E. canadensis* roots. Notably, WT+EV yeast exhibited the highest intracellular heavy metal accumulation, which can be attributed to the primary function of *YCF1* in vacuolar sequestration rather than extracellular efflux (Li *et al*. 1997). Continuous uptake and vacuolar storage of heavy metals elevate the total intracellular metal burden, thereby triggering cytotoxicity and inhibiting yeast growth.

In this study, transcripts encoding *LHT*s, which participate in amino acid uptake, transport, and utilization, were down-regulated (Tegeder and Ward 2012). However, the up-regulated AAT genes suggested the activation of a compensatory mechanism to alleviate amino acid deficiency, ensuring the supply of carbon and nitrogen nutrients for root cell growth and metabolism under heavy metal stress. In higher plants, sugars act as both essential nutrients and key signalling molecules, and sugar transporters play a pivotal role in mediating sugar distribution across plant organs and allocation between source and sink cells (Afoufa-Bastien *et al*. 2010). Furthermore, *AUX2* is closely associated with the regulation of cellular auxin distribution and maintenance of the root meristem niche, which is critical for sustaining root growth and development (Saito *et al*. 2019). The eukaryotic ribosome, a sophisticated translational machinery responsible for mRNA-mediated protein synthesis, consists of four rRNA types and 79 ribosomal proteins (RPs) (Guo *et al*. 2023). Ribosome biogenesis is an extremely complex process indispensable for cell growth, proliferation, and differentiation (Ahn *et al*. 2016). The synergistic action of these up-regulated genes may contribute to offsetting the mild toxic effects of heavy metal stress from MC treatment on roots, thus maintaining normal root growth of *E. canadensis*.

Exposure to toxic heavy metals triggers changes in gene expression profiles as plants initiate stress responses, including differential regulation of several signalling-related genes. Signalling molecules, particularly protein kinases, play critical roles in transducing stress signals and orchestrating downstream defence pathways essential for plant growth and environmental adaptation (Sessa and Martin 2000). For instance, GsSRK, a well-characterized positive regulator of salt stress tolerance in plants (Sun *et al*. 2013), was also significantly up-regulated in our study, suggesting that GsSRK may act as a conserved stress–responsive regulator that amplifies metal stress signals to induce protective responses under heavy metal exposure.

Cd and Pb exposure can induce oxidative stress in plants, despite their classification as non-redox metals (Thakur *et al*. 2019, Wang *et al*. 2021). Plants have evolved a specialized antioxidant defence system composed of enzymatic and non-enzymatic antioxidants to counteract oxidative damage (Singh *et al*. 2015). In the present study, the transcriptional reprogramming of antioxidant and sulphur metabolism was evident under heavy metal stress (Liu *et al*. 2017). In addition, the expression levels of these antioxidant related genes were closely correlated with plant stress tolerance (Yeboah *et al*. 2020). A larger proportion of ROS-related genes was down-regulated under HC treatment, suggesting that high-level metal stress might impose stronger pressure on *E. canadensis* roots and potentially limit the transcriptional regulation of antioxidant pathways.

The entry of heavy metal ions into plant root cells triggers ROS production, which subsequently activates various regulatory proteins, including transcription factors (Punziano *et al*. 2024). These transcription factors modulate a wide range of physiological processes by modulating the expression of downstream target genes, thereby enhancing heavy metal tolerance in plants (Yoon *et al*. 2020, Zhang *et al*. 2022). In the present study, several transcription factors, such as ZFPs, MYB, and WRKY, were found to be differentially expressed in *E. canadensis* roots under combined Cd and Pb stress. ZFPs are known to play a crucial role in plant growth and development by regulating the auxin signalling pathway (Wang *et al*. 2011). *PsMYB62*, isolated from *Potentilla sericea*, can enhance Cd resistance in arabidopsis by promoting antioxidant enzyme activity and increasing the content of antioxidant metabolites such as GSH (Feng *et al*. 2023). Additionally, *WRKY75* has been reported to enhance Cd accumulation and tolerance in poplar (Wu *et al*. 2022).

Considering its capacity to accumulate Cd and Pb without significant biomass reduction under certain exposure concentrations (Figs. 1 and 2), *E. canadensis* shows considerable potential as a phytoremediation resource for Cd and Pb contaminated soils. However, given the inherent limitations of this short-term pot experiment, field-scale experiments are needed to further assess its performance under natural environmental conditions and monitor heavy metal accumulation in roots and aerial parts across multiple growth cycles. Since heavy metals are primarily sequestered in *E. canadensis* roots, whole plants should be harvested and removed promptly after remediation to prevent secondary soil recontamination.

## Conclusions

*Erigeron canadensis* showed strong tolerance to combined Cd and Pb stress, with aboveground growth maintained across all treatments and root biomass reduced only at the highest concentration. Cd and Pb predominantly accumulated in roots, with Cd showing root-to-shoot translocation (TF > 1) and Pb being retained in roots (TF < 1). Transcriptomic and functional analyses revealed that root adaptation involved coordinated regulation of transporters, signal transduction, antioxidant defence, transcription factors, and ribosomal proteins. Heterologous expression in yeast validated *NRAMP3*, *ZntB*, and *CNGC2* as key mediators of Cd and Pb uptake and tolerance. Moderate stress up-regulated nutrient/auxin transporters and ribosomal proteins to sustain root growth, whereas high-level stress broadly repressed ROS-associated genes. These findings provide molecular insights into root-specific adaptive strategies of *E. canadensis* under combined heavy metal stress and guide its phytoremediation application.

## Supplementary Material

plag053_Supplementary_Data

## Data Availability

The sequence data that support the findings of this study have been deposited in the CNSA (http://db.cngb.org/cnsa) of CNGBdb with accession code CNP0005412.

## References

[plag053-B1] Afoufa-Bastien D, Medici A, Jeauffre J et al The *Vitis vinifera* sugar transporter gene family: phylogenetic overview and macroarray expression profiling. BMC Plant Biol 2010;10:245–66. 10.1186/1471-2229-10-245 PMC3095327 21073695

[plag053-B2] Ahn CS, Cho HK, Lee DH et al Functional characterization of the ribosome biogenesis factors PES, BOP1, and WDR12 (PeBoW), and mechanisms of defective cell growth and proliferation caused by PeBoW deficiency in Arabidopsis. J Exp Bot 2016;67:5217–32. 10.1093/jxb/erw288 PMC5014167 27440937

[plag053-B3] Andrews S . FastQC: a quality control tool for high throughput sequence data. 2010. https://www.bioinformatics.babraham.ac.uk/projects/fastqc (24 April 2010, date last accessed).

[plag053-B4] Ariyachandra SP, Alwis IS, Wimalasiri EM. Phytoremediation potential of heavy metals by *Cyperus rotundus*. Rev Agric Sci 2023;11:20–35. 10.7831/ras.11.0_20

[plag053-B5] Ba VN, Thien BN, Phuong HT et al Bioconcentration and translocation of elements from soil to vegetables and associated health risk. J Food Compos Anal 2024;132:106296. 10.1016/j.jfca.2024.106296

[plag053-B6] Baghour M, Moreno DA, Víllora G et al Phytoextraction of Cd and Pb and physiological effects in potato plants (*Solanum tuberosum* var. Spunta): importance of root temperature. J Agric Food Chem 2001;49:5356–63. 10.1021/jf010428x 11714328

[plag053-B7] Bolger AM, Lohse M, Bjoern U. Trimmomatic: a flexible trimmer for Illumina sequence data. Bioinformatics 2014;30:2114–20. 10.1093/bioinformatics/btu170 PMC4103590 24695404

[plag053-B8] Bu D, Luo H, Huo P et al KOBAS-i: intelligent prioritization and exploratory visualization of biological functions for gene enrichment analysis. Nucleic Acids Res 2021;49:W317–25. 10.1093/nar/gkab447 PMC8265193 34086934

[plag053-B9] Camacho C, Coulouris G, Avagyan V et al BLAST+: architecture and applications. BMC Bioinformatics 2009;10:421. 10.1186/1471-2105-10-421 PMC2803857 20003500

[plag053-B10] Cantalapiedra CP, Hernández-Plaza A, Letunic I et al eggNOG-mapper v2: functional annotation, orthology assignments, and domain prediction at the metagenomic scale. Mol Biol Evol 2021;38:5825–9. 10.1093/molbev/msab293 PMC8662613 34597405

[plag053-B11] Chaoprasid P, Nookabkaew S, Sukchawalit R et al Roles of *Agrobacterium tumefaciens* C58 *ZntA* and *ZntB* and the transcriptional regulator *ZntR* in controlling Cd^2+^/Zn^2+^/Co^2+^ resistance and the peroxide stress response. Microbiology 2015;161:1730–40. 10.1099/mic.0.000135 26296876

[plag053-B12] Checkai RT, Carey RB, Helmke PA. Effects of ionic and complexed metal concentrations on plant uptake of cadmium and micronutrient metals from solution. Plant Soil 1987;99:335–45. 10.1007/BF02370879

[plag053-B13] Clemens S . Toxic metal accumulation, responses to exposure and mechanisms of tolerance in plants. Biochimie 2006;88:1707–19. 10.1016/j.biochi.2006.07.003 16914250

[plag053-B14] Dai ZC, Cai HH, Qi SS et al Cadmium hyperaccumulation as an inexpensive metal armor against disease in crofton weed. Environ Pollut 2020;267:115649. 10.1016/j.envpol.2020.115649 33254657

[plag053-B15] de Oliveira LR, Brito G, Gama M et al Non-native decapods in South America: risk assessment and potential impacts. Diversity (Basel) 2023;15:841–57. 10.3390/d15070841

[plag053-B16] El-Sharkawy M, Li J, Kamal N et al Assessing and predicting soil quality in heavy metal-contaminated soils: statistical and ANN-based techniques. J Soil Sci Plant Nut 2023;23:6510–26. 10.1007/s42729-023-01507-w

[plag053-B17] Feng ZH, Gao B, Gao Y et al Cloning of *PsMYB62* and analysis of cadmium resistant in *Potentilla sericea*. Biocell 2023;47:1571–82. 10.32604/biocell.2023.029106

[plag053-B18] Fidalgo F, Azenha M, Silva AF et al Copper-induced stress in *Solanum nigrum* L. and antioxidant defense system responses. Food Energy Secur 2013;2:70–80. 10.1002/fes3.20

[plag053-B19] GB 15618-2018 . Soil Environmental Quality—Risk Control Standard for Soil Contamination of Agricultural Land. Beijing: Ministry of Ecology and Environment of the People's Republic of China, 2018.

[plag053-B20] Grotz N, Fox T, Connolly E et al Identification of a family of zinc transporter genes from *Arabidopsis* that respond to zinc deficiency. Proc Natl Acad Sci U S A 1998;95:7220–4. 10.1073/pnas.95.12.7220 PMC22785 9618566

[plag053-B21] Gulezian PZ, Jennifer LI, Granberg KJ. Establishment of an invasive plant species (*Conium maculatum*) in contaminated roadside soil in cook county, Illinois. Am Midl Nat 2012;168:375–95. 10.1674/0003-0031-168.2.375

[plag053-B22] Guo L, Guo H, Zhang Y et al Upregulated ribosome pathway plays a key role in *HDAC4*, improving the survival rate and biofunction of chondrocytes. Bone Joint Res 2023;12:433–46. 10.1302/2046-3758.127.Bjr-2022-0279.R2 PMC10325875 37414410

[plag053-B23] January M, Cutright T, Keulen HV et al Hydroponic phytoremediation of Cd, Cr, Ni, As, and Fe: can *Helianthus annuus* hyperaccumulate multiple heavy metals? Chemosphere 2008;70:531–7. 10.1016/j.chemosphere.2007.06.066 17697697

[plag053-B24] Kama R, Liu Y, Aidara M et al Plant–soil feedback combined with straw incorporation under maize/soybean intercropping increases heavy metals migration in soil–plant system and soil HMRG abundance under livestock wastewater irrigation. J Soil Sci Plant Nutr 2024;24:7090–104. 10.1007/s42729-024-02026-y

[plag053-B25] Khan IU, Zhang YF, Shi XN et al Dose dependent effect of nitrogen on the phyto extractability of Cd in metal contaminated soil using *Wedelia trilobata*. Ecotoxicol Environ Saf 2023;264:115419. 10.1016/j.ecoenv.2023.115419 37651793

[plag053-B26] Kim D, Langmead B, Salzberg SL. HISAT: a fast spliced aligner with low memory requirements. Nat Methods 2015;12:357–60. 10.1038/nmeth.3317 PMC4655817 25751142

[plag053-B27] Kushwaha A, Rani R, Kumar S et al Heavy metal detoxification and tolerance mechanisms in plants: implications for phytoremediation. Environ Rev 2016;24:39–51. 10.1139/er-2015-0010

[plag053-B28] Laforest M, Martin SL, Bisaillon K et al A chromosome-scale draft sequence of the Canada fleabane genome. Pest Manage Sci 2020;76:2158–69. 10.1002/ps.5753 31951071

[plag053-B29] Li Y, Zhou CF, Huang MY et al Lead tolerance mechanism in *Conyza canadensis*: subcellular distribution, ultrastructure, antioxidative defense system, and phytochelatins. J Plant Res 2016;129:251–62. 10.1007/s10265-015-0776-x 26733305

[plag053-B30] Li ZS, Lu YP, Zhen RG et al A new pathway for vacuolar cadmium sequestration in *Saccharomyces cerevisiae*: YCF1-catalyzed transport of bis(glutathionato)cadmium. Proc Natl Acad Sci U S A 1997;94:42–7. 10.1073/pnas.94.1.42 PMC19233 8990158

[plag053-B31] Liu Y, Yu X, Feng Y et al Physiological and transcriptome response to cadmium in cosmos (*Cosmos bipinnatus* Cav.) seedlings. Sci Rep-UK 2017;7:14691. 10.1038/s41598-017-14407-8 PMC5665871 29089633

[plag053-B32] Love MI, Huber W, Anders S. Moderated estimation of fold change and dispersion for RNA-Seq data with DESeq2. Genome Biol 2014;15:550–70. 10.1186/s13059-014-0550-8 PMC4302049 25516281

[plag053-B33] Martin N, Blanchet S, Hermet S et al Multiscale physiological responses to organic and inorganic pollution in the invasive mosquitofish *Gambusia holbrooki*. Ecotoxicol Environ Saf 2025;302:118565. 10.1016/j.ecoenv.2025.118565 40580636

[plag053-B34] Melaku S, Dams R, Moens L. Determination of trace elements in agricultural soil samples by inductively coupled plasma-mass spectrometry: microwave acid digestion versus aqua regia extraction. Anal Chim Acta 2005;543:117–23. 10.1016/j.aca.2005.04.055

[plag053-B35] MEP of China . *National Soil Pollution Survey Bulletin*. https://www.mee.gov.cn/gkml/sthjbgw/qt/201404/t20140417_270670.htm (17 April 2014, date last accessed).

[plag053-B36] Moon JY, Belloeil C, Ianna ML et al *Arabidopsis* CNGC family members contribute to heavy metal ion uptake in plants. Int J Mol Sci 2019;20:413. 10.3390/ijms20020413 PMC6358908 30669376

[plag053-B37] Paulose B, Jaiwal PK, Dhankher OP. Heavy metal transporters in plants. In: Jaiwal P, Singh R, Dhanker O (eds.) Plant Membrane and Vacuolar Transporters. Wallingford: CAB International, 2009, 213–38.

[plag053-B38] Pertea G, Pertea M. GFF utilities: GffRead and GffCompare. F1000Res 2020;9:304. 10.12688/f1000research.23297.1 PMC7222033 32489650

[plag053-B39] Pertea M, Pertea GM, Antonescu CM et al The StringTie enables improved reconstruction of a transcriptome from RNA-Seq reads. Nat Biotechnol 2015;33:290–5. 10.1038/nbt.3122 PMC4643835 25690850

[plag053-B40] Punziano C, Trombetti S, Cesaro E et al Antioxidant systems as modulators of ferroptosis: focus on transcription factors. Antioxidants 2024;13:298. 10.3390/antiox13030298 PMC10967371 38539832

[plag053-B41] Queiroz SC, Cantrell CL, Duke SO et al Bioassay-directed isolation and identification of phytotoxic and fungitoxic acetylenes from *Conyza canadensis*. J Agric Food Chem 2012;60:5893–8. 10.1021/jf3010367 22612410

[plag053-B42] Rao X, Huang X, Zhou Z et al An improvement of the 2^(−delta delta CT) method for quantitative real-time polymerase chain reaction data analysis. Biostat Bioinforma Biomath 2013;3:71–85. PMC4280562 25558171

[plag053-B43] Saito T, Opio P, Wang S et al Association of auxin, cytokinin, abscisic acid, and plant peptide response genes during adventitious root formation in Marubakaido apple rootstock (*Malus prunifolia* Borkh. var. ringo Asami). Acta Physiol Plant 2019;41:41–50. 10.1007/s11738-019-2827-8

[plag053-B44] Savoca D, Orecchio S, Maccotta A et al Tracking polycyclic aromatic hydrocarbons (PAHs) across Palermo with the invasive grass *Cenchrus setaceus* (syn. *Pennisetum setaceum*). Environ Pollut 2026;400:128279. 10.1016/j.envpol.2026.128279 42102998

[plag053-B45] Sessa G, Martin GB. Protein kinases in the plant defense response. Adv Bot Res 2000;32:379–404. 10.1016/S0065-2296(00)32030-4

[plag053-B46] Shi G, Cai Q. Cadmium tolerance and accumulation in eight potential energy crops. Biotechnol Adv 2009;27:555–61. 10.1016/j.biotechadv.2009.04.006 19393309

[plag053-B47] Singh S, Parihar P, Singh R et al Heavy metal tolerance in plants: role of transcriptomics, proteomics, metabolomics, and ionomics. Front Plant Sci 2015;6:1143–78. 10.3389/fpls.2015.01143 PMC4744854 26904030

[plag053-B48] Storey JD, Tibshirani R. Statistical significance for genomewide studies. Proc Natl Acad Sci U S A 2003;100:9440–5. 10.1073/pnas.1530509100 PMC170937 12883005

[plag053-B49] Sun XL, Yu QY, Tang LL et al GsSRK, a G-type lectin S-receptor-like serine/threonine protein kinase, is a positive regulator of plant tolerance to salt stress. J Plant Physiol 2013;170:505–15. 10.1016/j.jplph.2012.11.017 23276523

[plag053-B50] Sunkar R, Kaplan B, Bouché N et al Expression of a truncated tobacco *NtCBP4* channel in transgenic plants and disruption of the homologous *Arabidopsis CNGC1* gene confer Pb^2+^ tolerance. Plant J 2000;24:533–42. 10.1111/j.1365-313X.2000.00901.x 11115134

[plag053-B51] Tegeder M, Ward JM. Molecular evolution of plant AAP and LHT amino acid transporters. Front Plant Sci 2012;3:21. 10.3389/fpls.2012.00021 PMC3355764 22645574

[plag053-B52] Thakur S, Choudhary S, Bhardwaj P. Comparative transcriptome profiling under cadmium stress reveals the uptake and tolerance mechanism in *Brassica juncea*. J Plant Growth Regul 2019;38:1141–52. 10.1007/s00344-019-09919-8

[plag053-B53] Thomine S, Wang R, Ward JM et al Cadmium and iron transport by members of a plant metal transporter family in *Arabidopsis* with homology to Nramp genes. Proc Natl Acad Sci U S A 2000;97:4991–6. 10.1073/pnas.97.9.4991 PMC18345 10781110

[plag053-B54] Tian WJ, He GD, Qin LJ et al Genome-wide analysis of the NRAMP gene family in potato (*Solanum tuberosum*): identification, expression analysis and response to five heavy metals stress. Ecotoxicol Environ Saf 2021;208:111661. 10.1016/j.ecoenv.2020.111661 33396171

[plag053-B55] Tőzsér D, Magura T, Simon E. Heavy metal uptake by plant parts of willow species: a meta-analysis. J Hazard Mater 2017;336:101–9. 10.1016/j.jhazmat.2017.03.068 28482187

[plag053-B56] Uveges JL, Corbett AL, Mal TK. Effects of lead contamination on the growth of *Lythrum salicaria* (purple loosestrife). Environ Pollut 2002;120:319–23. 10.1016/S0269-7491(02)00144-6 12395845

[plag053-B57] Wang L, Hua D, He J et al Auxin response factor2 (*ARF2*) and its regulated homeodomain gene *HB33* mediate abscisic acid response in *Arabidopsis*. PLoS Genet 2011;7:e1002172. 10.1371/journal.pgen.1002172 PMC3136439 21779177

[plag053-B58] Wang Y, Meng Y, Mu SJ et al Changes in phenotype and gene expression under lead stress revealed key genetic responses to lead tolerance in *Medicago sativa* L. Gene 2021;791:145714. 10.1016/j.gene.2021.145714 33979680

[plag053-B59] Weber E, Sun SG, Li B. Invasive alien plants in China: diversity and ecological insights. Biol Invasions 2008;10:1411–29. 10.1007/s10530-008-9216-3

[plag053-B60] Wei W, Chai T, Zhang Y et al The *Thlaspi caerulescens* NRAMP homologue *TcNRAMP3* is capable of divalent cation transport. Mol Biotechnol 2009;41:15–21. 10.1007/s12033-008-9088-x 18663607

[plag053-B61] Weidenhamer JD, Callaway RM. Direct and indirect effects of invasive plants on soil chemistry and ecosystem function. J Chem Ecol 2010;36:59–69. 10.1007/s10886-009-9735-0 20077127

[plag053-B62] Wright PB, Steven JC. Accumulation of heavy metals in *Conyza canadensis*. Integr Comp Biol 2024;64:645–54. 10.1093/icb/icae119 39038995

[plag053-B63] Wu X, Chen Q, Chen L et al A WRKY transcription factor, *PyWRKY75*, enhanced cadmium accumulation and tolerance in poplar. Ecotoxicol Environ Saf 2022;239:113630. 10.1016/j.ecoenv.2022.113630 35569299

[plag053-B64] Xu L, Zhang F, Tang MJ et al Melatonin confers cadmium tolerance by modulating critical heavy metal chelators and transporters in radish plants. J Pineal Res 2020;69:e12659. 10.1111/jpi.12659 32323337

[plag053-B65] Yang QQ, Li ZY, Lu XN et al A review of soil heavy metal pollution from industrial and agricultural regions in China: pollution and risk assessment. Sci Total Environ 2018;642:690–700. 10.1016/j.scitotenv.2018.06.068 29909337

[plag053-B66] Ye WL, Guo GF, Wu F et al Absorption, translocation, and detoxification of Cd in two different castor bean (*Ricinus communis* L.) cultivars. Environ Sci Pollut Res Int 2018;25:28899–906. 10.1007/s11356-018-2915-0 30105674

[plag053-B67] Yeboah A, Lu JN, Ting Y et al Assessment of castor plant (*Ricinus communis* L.) tolerance to heavy metal stress—A review. Phyton (B Aires) 2020;89:453–72. 10.32604/phyton.2020.09267

[plag053-B68] Yoon Y, Seo DH, Shin H et al The role of stress–responsive transcription factors in modulating abiotic stress tolerance in plants. Agronomy 2020;10:788–810. 10.3390/agronomy10060788

[plag053-B69] Yu G, Wang L, Han Y et al clusterProfiler: an R package for comparing biological themes among gene clusters. OMICS 2012;16:284–7. 10.1089/omi.2011.0118 PMC3339379 22455463

[plag053-B70] Yuan X, Liang R, Wang G et al Design of rice with low cadmium accumulation in grain using single segment substitution line. New Crops 2025;2:100035. 10.1016/j.ncrops.2024.100035

[plag053-B71] Zhang HY, Goncalves P, Copeland E et al Invasion by the weed *Conyza canadensis* alters soil nutrient supply and shifts microbiota structure. Soil Biol Biochem 2020;143:107739. 10.1016/j.soilbio.2020.107739

[plag053-B72] Zhang HY, Rutherford S, Qi SS et al Transcriptome profiling of *Arabidopsis thaliana* roots in response to allelopathic effects of *Conyza canadensis*. Ecotoxicology 2022;31:53–63. 10.1007/s10646-021-02489-0 34647200

[plag053-B73] Zhang XD, Meng JG, Zhao KX et al Annotation and characterization of Cd-responsive metal transporter genes in rapeseed (*Brassica napus*). BioMetals 2018;31:107–21. 10.1007/s10534-017-0072-4 29250721

[plag053-B74] Zhao Y, Gao L, Zha F et al Research on heavy metal level and co-occurrence network in typical ecological fragile area. J Environ Health Sci 2021;19:531–40. 10.1007/s40201-021-00625-w PMC8172680 34150256

[plag053-B75] Zhou C, Zhang K, Lin J et al Physiological responses and tolerance mechanisms to cadmium in *Conyza canadensis*. Int J Phytoremediation 2015;17:280–9. 10.1080/15226514.2014.898021 25397987

[plag053-B76] Żurek G, Wiewióra B, Krystyna R et al Different response of perennial ryegrass—*Epichloë* endophyte symbiota to the elevated concentration of heavy metals in soil. J Appl Genet 2022;63:47–59. 10.1007/s13353-021-00661-0 PMC8755660 34546560

