# Supplementary material for: Transcriptome insights into root adaptation of Erigeron canadensis to combined cadmium and lead stress
Source: AoB Plants. 2026 Oct 15;18(6):plag053. doi: 10.1093/aobpla/plag053 (PMC13721876; doi:10.1093/aobpla/plag053)
Supplement: Supplementary file 1 [file plag053_supplementary_data.pdf]

Table S1 Primers used for qRT-PCR

Primer	Sequence (5'-3')	Length（bp）	Tm (°C)	Expression level by RNA sequence
UBE22-F	TCC AAA CTC ACC AGC CAA CT	101	57.7	reference gene
UBE22-R	CTG TCC AGC TCT GCT CAA CC	101	57.9	
ABCC4-F	TGA AAG AAG ATG CGG TGG AG	178	58.5	Down -regulated
ABCC4-R	TTG CCC GAA CCT ACT GTG C	178	59.2	
STK-CCR4-F	TGA AGG CGA AAC GAG GTG	126	57.4	Down-regulated
STK-CCR4-R	ACA AAC CAG GCA CCA CAT TAC	126	57.4	
CIPK2-F	GCC TTC GTA TGT TGC TCC TG	109	58.2	Down-regulated
CIPK2-R	AAG AAA GCC AGC CAT AAG CA	109	57.9	
GSTU19-F	CTA TGG GTT TAG CAT GTT CGG T	120	58.8	Up-regulated
GSTU19-R	GGG TTC ATC TCA AGG AGC AGT	120	58.2	

Table S2 Data evaluation statistics

Treatments	Obtained Bases (Gb)	Obtained Reads	The average read length (bp)	The longest read length (bp)	Q30 (%)	GC (%)
CK1	5.63	39,159,658	144	150	94.9	46.1
CK2	5.47	38,048,744	144	150	96.0	45.6
CK3	7.40	50,955,948	145	150	96.2	46.9
LC1	7.78	53,840,984	144	150	96.2	46.9
LC2	6.30	43,668,948	144	150	96.5	42.7
LC3	6.70	46,933,678	143	150	94.5	45.9
MC1	6.99	48,786,732	143	150	96.4	46.8
MC2	7.65	53,425,008	143	150	97.3	47.0
MC3	11.60	80,851,532	144	150	96.1	49.3
HC1	6.51	45,357,942	143	150	95.5	47.2
HC2	8.28	57,929,930	143	150	95.0	47.7
HC3	9.48	65,699,930	144	150	94.7	48.0

CK, LC, MC, and HC represent the control (no Cd or Pb addition), low concentration (1 mg/kg Cd and 90 mg/kg Pb), moderate concentration (2 mg/kg Cd and 180 mg/kg Pb), and high concentration (4 mg/kg Cd and 360 mg/kg Pb) treatments, respectively. Numbers following CK, LC, MC, and HC indicate the replicate number.

Table S3 Differentially expressed genes (DEGs) identified in *Erigeron canadensis* roots under the LC treatment

Transcript id	GeneID	Gene name/function	*P* value	result
XR_006322304.1	LOC122589692	uncharacterized LOC122589692	< 0.0001	up
XM_043775015.1	LOC122602316	flowering-promoting factor 1-like protein 3	< 0.0001	up
XM_043761142.1	LOC122588921	GDSL lipase-like	0.0011	up
XM_043776367.1	LOC122603616	protein GDAP2 homolog	0.0005	up
XM_043758692.1	LOC122586706	probable cadmium/zinc-transporting ATPase HMA1, chloroplastic, transcript variant X4	0.0001	up
XM_043781227.1	LOC122608136	wound-induced basic protein	0.0011	up
XM_043761083.1	LOC122588867	chaperone protein dnaJ 8, chloroplastic	0.0001	up
XR_006323643.1	LOC122598527	uncharacterized LOC122598527	< 0.0001	up
XM_043768098.1	LOC122595678	GATA transcription factor 24-like, transcript variant X1	0.0001	up
XM_043754929.1	LOC122582524	uncharacterized LOC122582524	0.0011	up
XM_043778609.1	LOC122605651	probable serine/threonine-protein kinase PBL3	0.0003	up
XM_043767060.1	LOC122594627	beta-1,3-galactosyltransferase pvg3-like	0.0005	up
XM_043757975.1	LOC122585856	uncharacterized LOC122585856	0.0005	up
XM_043780818.1	LOC122607779	glycine-rich RNA-binding protein 4, mitochondrial-like, transcript variant X1	0.0003	up
XM_043761880.1	LOC122589576	inorganic pyrophosphatase 2-like	< 0.0001	up
XM_043756353.1	LOC122583984	putative receptor-like protein kinase At5g39000, transcript variant X2	0.0013	up
XM_043769326.1	LOC122596706	uncharacterized LOC122596706, transcript variant X1	0.0004	up
XM_043753163.1	LOC122581022	auxin transporter-like protein 2	0.0003	up
XM_043762347.1	LOC122590009	peamaclein	0.0011	up
XM_043750919.1	LOC122578870	isoaspartyl peptidase/L-asparaginase	< 0.0001	up
XM_043758938.1	LOC122586913	amino acid permease 6-like, transcript variant X2	< 0.0001	up
XM_043762910.1	LOC122590735	EG45-like domain containing protein, transcript variant X2	0.0002	up
XM_043781297.1	LOC122608210	glutaredoxin-C11-like	0.0011	up
XM_043770766.1	LOC122598166	protein XRI1-like	< 0.0001	up
XM_043759238.1	LOC122587148	homeobox-leucine zipper protein HAT5-like, transcript variant X2	< 0.0001	up
XM_043753950.1	LOC122581696	homocysteine S-methyltransferase 2-like	< 0.0001	up
XM_043751036.1	LOC122578958	protein transport protein SEC23, transcript variant X1	< 0.0001	up
XM_043753514.1	LOC122581309	uncharacterized LOC122581309, transcript variant X5	0.0004	up
XM_043780158.1	LOC122607222	DNA polymerase zeta processivity subunit, transcript variant X2	< 0.0001	up
XM_043778854.1	LOC122605898	glycerophosphodiester phosphodiesterase GDPD1, chloroplastic	< 0.0001	up
XM_043777930.1	LOC122605056	non-symbiotic hemoglobin 2-like	0.0001	up
XM_043770372.1	LOC122597799	transcription initiation factor TFIID subunit 6-like, transcript variant X1	0.0007	up
XM_043751714.1	LOC122579528	eyes absent homolog	0.0003	up
XM_043781166.1	LOC122608079	protein DELETION OF SUV3 SUPPRESSOR 1(I)-like, transcript variant X2	< 0.0001	up
XR_006321355.1	LOC122583940	uncharacterized LOC122583940	0.0011	up
XR_006322102.1	LOC122587056	serine/arginine-rich splicing factor SC35-like, transcript variant X4	0.0002	up
XM_043755437.1	LOC122583004	transcription factor ORG2-like	< 0.0001	up
XM_043760565.1	LOC122588448	protein SET DOMAIN GROUP 41, transcript variant X2	< 0.0001	up
XM_043764462.1	LOC122592273	stigma-specific STIG1-like protein 1	< 0.0001	up
XR_006321821.1	LOC122585922	uncharacterized LOC122585922	< 0.0001	up
XM_043766707.1	LOC122594236	UPF0481 protein At3g47200-like	0.0013	up
XM_043770037.1	LOC122597444	metacaspase-3-like	< 0.0001	up
XM_043780270.1	LOC122607319	premnaspirodiene oxygenase-like	0.0008	up
XM_043759321.1	LOC122587215	uncharacterized LOC122587215, transcript variant X2	0.0007	up
XM_043771309.1	LOC122598822	uncharacterized LOC122598822	< 0.0001	up
XM_043768727.1	LOC122596194	heparanase-like protein 3	< 0.0001	up
XM_043761156.1	LOC122588936	gibberellin-regulated protein 14	0.0001	up
XM_043782648.1	LOC122609700	uncharacterized LOC122609700	< 0.0001	up
XM_043772164.1	LOC122599626	protein IQ-DOMAIN 5-like, transcript variant X1	< 0.0001	up
XM_043782735.1	LOC122609792	probable xyloglucan endotransglucosylase/hydrolase protein B	0.0007	up
XM_043766325.1	LOC122593871	suppressor protein SRP40-like	0.0002	up
XM_043774722.1	LOC122601990	ATP-dependent DNA helicase Q-like SIM, transcript variant X1	0.0001	up
XM_043763735.1	LOC122591467	EG45-like domain containing protein	< 0.0001	up
XM_043780354.1	LOC122607388	chalcone isomerase-like protein 2	0.0001	up
XM_043756960.1	LOC122584885	uncharacterized LOC122584885	0.0006	up
XM_043765675.1	LOC122593284	uncharacterized LOC122593284	0.0002	up
XM_043753811.1	LOC122581577	pentatricopeptide repeat-containing protein At4g20740, transcript variant X2	< 0.0001	up
XM_043778500.1	LOC122605544	DNA polymerase epsilon subunit 3-like	0.0003	up

Transcript id	GeneID	Gene name/function	*P* value	result
XM_043778576.1	LOC122605615	probable inactive receptor kinase At4g23740	0.0007	up
XM_043771234.1	LOC122598647	uncharacterized LOC122598647	0.0005	up
XR_006323951.1	LOC122599834	uncharacterized LOC122599834, transcript variant X4	0.0002	up
XM_043784330.1	LOC122611320	ACT domain-containing protein ACR6, transcript variant X1	0.0001	up
XM_043765191.1	LOC122592872	plasmodesmata-located protein 2-like	0.0008	up
XM_043769693.1	LOC122597058	putative receptor-like protein kinase At4g00960	0.0012	up
XR_006324199.1	LOC122602286	uncharacterized LOC122602286	0.0002	up
XM_043766446.1	LOC122593971	uncharacterized LOC122593971	0.0003	up
XM_043762361.1	LOC122590024	uncharacterized LOC122590024	0.0007	up
XM_043772820.1	LOC122600153	ABC transporter C family member 3-like	0.0011	up
XM_043762318.1	LOC122589987	probable (S)-N-methylcoclaurine 3'-hydroxylase isozyme 2	0.0008	up
XM_043765566.1	LOC122593194	uncharacterized LOC122593194	0.0002	up
XM_043766643.1	LOC122594176	basic blue protein-like	0.0001	up
XM_043759511.1	LOC122587363	uncharacterized LOC122587363, transcript variant X1	< 0.0001	up
XM_043779660.1	LOC122606805	polygalacturonase inhibitor-like	< 0.0001	up
XM_043779932.1	LOC122607014	histone H2A	0.0010	up
XM_043776690.1	LOC122603860	mediator of RNA polymerase II transcription subunit 11, transcript variant X2	< 0.0001	up
XM_043782887.1	LOC122609934	tubulin-folding cofactor C, transcript variant X2	0.0013	up
XM_043754421.1	LOC122582072	non-specific lipid-transfer protein 1-like	0.0004	up
XM_043753298.1	LOC122581138	uncharacterized LOC122581138	< 0.0001	up
XM_043774706.1	LOC122601973	uncharacterized LOC122601973	0.0002	up
XM_043767247.1	LOC122594967	BTB/POZ domain-containing protein At1g67900, transcript variant X1	< 0.0001	up
XM_043759604.1	LOC122587437	homeobox-leucine zipper protein ATHB-15, transcript variant X1	0.0012	up
XM_043783109.1	LOC122610114	zingipain-2-like	0.0002	up
XR_006324848.1	LOC122606272	protein MARD1-like, transcript variant X2	0.0013	up
XM_043775352.1	LOC122602758	endonuclease MutS2, transcript variant X1	0.0009	up
XM_043780100.1	LOC122607173	60S ribosomal protein L22-3-like	0.0003	up
XM_043767746.1	LOC122595392	putative receptor-like protein kinase At3g47110	0.0012	up
XM_043782024.1	LOC122608949	E3 ubiquitin-protein ligase AIRP2-like, transcript variant X1	0.0008	up
XM_043761794.1	LOC122589499	kinesin-like protein KIN-14C, transcript variant X1	< 0.0001	up
XM_043772650.1	LOC122600020	3-hydroxyisobutyryl-CoA hydrolase-like protein 1, mitochondrial, transcript variant X1	< 0.0001	up
XM_043780035.1	LOC122607118	phosphatidylinositol-3-phosphatase SAC1, transcript variant X1	0.0001	up
XM_043750055.1	LOC122578156	AP-1 complex subunit sigma-2, transcript variant X1	0.0012	up
XM_043762657.1	LOC122590455	glutathione S-transferase U8-like	0.0003	up
XM_043751762.1	LOC122579573	uncharacterized LOC122579573	0.0005	up
XM_043778952.1	LOC122605987	uncharacterized LOC122605987	0.0006	up
XM_043755206.1	LOC122582773	bZIP transcription factor 53-like	< 0.0001	up
XM_043762667.1	LOC122590082	uncharacterized LOC122590082	0.0005	up
XM_043775936.1	LOC122603267	D-2-hydroxyglutarate dehydrogenase, mitochondrial, transcript variant X1	0.0011	up
XM_043764850.1	LOC122592584	MADS-box protein SOC1, transcript variant X4	0.0004	up
XM_043781732.1	LOC122608640	transcription initiation factor TFIID subunit 12b, transcript variant X2	< 0.0001	up
XM_043761120.1	LOC122588900	protein RESPONSE TO LOW SULFUR 3-like	< 0.0001	up
XM_043765659.1	LOC122593271	heterogeneous nuclear ribonucleoprotein 1-like, transcript variant X3	0.0001	up
XM_043775712.1	LOC122603097	probable serine/threonine-protein kinase SIS8, transcript variant X1	< 0.0001	up
XR_006322832.1	LOC122593405	uncharacterized LOC122593405, transcript variant X2	< 0.0001	up
XM_043771971.1	LOC122599456	RGG repeats nuclear RNA binding protein A-like, transcript variant X1	0.0008	up
XM_043756999.1	LOC122584919	uncharacterized LOC122584919, transcript variant X1	0.0004	up
XM_043769319.1	LOC122596698	growth-regulating factor 6, transcript variant X2	< 0.0001	up
XM_043768166.1	LOC122595738	very-long-chain 3-oxoacyl-CoA reductase 1-like	0.0006	up
XM_043755359.1	LOC122582929	probable N-acetyltransferase HLS1-like	0.0009	up
XM_043776870.1	LOC122603993	F-box protein PP2-A15-like, transcript variant X3	< 0.0001	up
XM_043770510.1	LOC122597920	serine carboxypeptidase-like 31	0.0004	up
XM_043780095.1	LOC122607166	mediator of RNA polymerase II transcription subunit 19a-like, transcript variant X1	0.0006	up
XM_043768922.1	LOC122596358	putative receptor-like protein kinase At4g00960	0.0010	up
XR_006321796.1	LOC122585851	uncharacterized LOC122585851	0.0007	up
XM_043757306.1	LOC122585194	21 kDa protein-like	0.0011	up
XR_006325214.1	LOC122608349	uncharacterized LOC122608349	< 0.0001	up
XM_043761026.1	LOC122588818	DNA-repair protein XRCC1, transcript variant X1	0.0009	up

Transcript id	GeneID	Gene name/function	*P* value	result
XM_043769119.1	LOC122596525	protein NUCLEAR FUSION DEFECTIVE 6, mitochondrial-like, transcript variant X2	< 0.0001	up
XM_043771644.1	LOC122599183	pectinesterase 2-like	0.0007	up
XM_043775012.1	LOC122602311	glycosyltransferase BC10-like	0.0008	up
XM_043775470.1	LOC122602863	protein-tyrosine-phosphatase MKP1-like, transcript variant X2	< 0.0001	up
XM_043761660.1	LOC122589371	tyrosine--tRNA ligase 1, cytoplasmic-like, transcript variant X3	0.0012	up
XM_043764667.1	LOC122592441	probable purine permease 11, transcript variant X3	< 0.0001	up
XM_043762751.1	LOC122590552	uncharacterized LOC122590552	0.0008	up
XM_043766069.1	LOC122593640	mitochondrial import inner membrane translocase subunit TIM10-like, transcript variant X1	0.0011	up
XR_006325363.1	LOC122609665	uncharacterized LOC122609665	< 0.0001	up
XR_006321644.1	LOC122585045	uncharacterized LOC122585045	0.0004	up
XM_043761245.1	LOC122589014	uncharacterized LOC122589014, transcript variant X3	0.0001	up
XM_043755250.1	LOC122582813	calcium-binding allergen Ole e 8-like	0.0004	up
XM_043768407.1	LOC122595925	uncharacterized LOC122595925	0.0003	up
XM_043762124.1	LOC122589804	cytochrome P450 81Q32-like	0.0009	up
XM_043758806.1	LOC122586815	nodulation receptor kinase-like, transcript variant X1	0.0003	up
XM_043780387.1	LOC122607418	uncharacterized LOC122607418, transcript variant X1	< 0.0001	up
XM_043752748.1	LOC122580475	glycosyltransferase BC10-like, transcript variant X1	0.0009	up
XM_043753496.1	LOC122581294	PHD finger protein ALFIN-LIKE 2-like, transcript variant X2	< 0.0001	up
XM_043761489.1	LOC122589230	H/ACA ribonucleoprotein complex subunit 2-like protein	0.0003	up
XM_043752635.1	LOC122580361	TLC domain-containing protein At5g14285-like	0.0008	up
XM_043772298.1	LOC122599738	ribosomal RNA small subunit methyltransferase, transcript variant X2	0.0008	up
XM_043757245.1	LOC122585140	expansin-B15-like	0.0004	up
XM_043770419.1	LOC122597847	gibberellin-regulated protein 1-like	0.0003	up
XM_043763711.1	LOC122591443	uncharacterized LOC122591443	0.0008	up
XM_043764556.1	LOC122592353	uncharacterized LOC122592353, transcript variant X2	< 0.0001	up
XM_043783253.1	LOC122610260	F-box protein CPR1-like	0.0003	up
XR_006320704.1	LOC122579814	uncharacterized LOC122579814	0.0009	up
XM_043773770.1	LOC122600991	protein DOG1-like 3	0.0003	down
XM_043755824.1	LOC122583419	uncharacterized LOC122583419	< 0.0001	down
XM_043753886.1	LOC122581640	glycine-rich protein A3-like	< 0.0001	down
XM_043755911.1	LOC122583515	uncharacterized protein At4g26485-like	0.0004	down
XM_043775529.1	LOC122602916	auxin-induced protein PCNT115-like, transcript variant X2	< 0.0001	down
XM_043768584.1	LOC122596068	receptor-like protein EIX1	< 0.0001	down
XM_043768853.1	LOC122596304	cytochrome P450 78A5-like	0.0002	down
XM_043760835.1	LOC122588671	AP2-like ethylene-responsive transcription factor ANT, transcript variant X3	0.0001	down
XM_043765796.1	LOC122593394	3-oxoacyl-[acyl-carrier-protein] reductase FabG-like	< 0.0001	down
XM_043754070.1	LOC122581791	protein TRANSPORT INHIBITOR RESPONSE 1-like	0.0006	down
XM_043769339.1	LOC122596715	two-component response regulator ORR26-like	< 0.0001	down
XM_043775551.1	LOC122602937	copper transport protein CCH-like	< 0.0001	down
XM_043756802.1	LOC122584749	ethylene-responsive transcription factor TINY-like	< 0.0001	down
XM_043758186.1	LOC122586081	acidic endochitinase-like	0.0002	down
XM_043765580.1	LOC122593204	E3 ubiquitin-protein ligase RDUF1-like	< 0.0001	down
XM_043756036.1	LOC122583650	uncharacterized LOC122583650	< 0.0001	down
XM_043752489.1	LOC122580211	alpha,alpha-trehalose-phosphate synthase [UDP-forming] 5	0.0002	down
XM_043775312.1	LOC122602723	wall-associated receptor kinase-like 14, transcript variant X1	0.0002	down
XM_043775689.1	LOC122603077	lanC-like protein GCL1	< 0.0001	down
XM_043755080.1	LOC122582657	uncharacterized LOC122582657	0.0006	down
XM_043761471.1	LOC122589219	cyclin-dependent kinase G-2-like	0.0008	down
XM_043774939.1	LOC122602226	carotenoid cleavage dioxygenase 7, chloroplastic	0.0001	down
XM_043754240.1	LOC122581920	uncharacterized LOC122581920, transcript variant X1	< 0.0001	down
XR_006320916.1	LOC122580896	uncharacterized LOC122580896, transcript variant X2	< 0.0001	down
XM_043772837.1	LOC122600164	phosphoenolpyruvate carboxylase 4-like	0.0003	down
XM_043763752.1	LOC122591485	wall-associated receptor kinase 2-like	< 0.0001	down
XM_043780871.1	LOC122607819	serine/threonine-protein kinase-like protein CCR4	0.0002	down
XM_043770716.1	LOC122598115	membrane protein PM19L-like	0.0006	down
XM_043781858.1	LOC122608768	CRAL-TRIO domain-containing protein YKL091C-like	0.0007	down
XR_006323152.1	LOC122594929	uncharacterized LOC122594929	< 0.0001	down
XM_043766687.1	LOC122594214	polyubiquitin-like	0.0001	down
XM_043769149.1	LOC122596547	protein ALP1-like	0.0009	down
XR_006321208.1	LOC122582648	uncharacterized LOC122582648, transcript variant X1	0.0002	down
XM_043782761.1	LOC122609817	uncharacterized LOC122609817, transcript variant X2	< 0.0001	down

Transcript id	GeneID	Gene name/function	*P* value	result
XM_043751825.1	LOC122579619	calmodulin-binding protein 60 E-like, transcript variant X1	0.0009	down
XM_043776265.1	LOC122603537	hydroquinone glucosyltransferase-like	0.0006	down
XM_043765976.1	LOC122593552	uncharacterized LOC122593552	< 0.0001	down
XM_043775540.1	LOC122602926	putative disease resistance protein At1g59780, transcript variant X1	< 0.0001	down
XM_043751590.1	LOC122579417	putative peptidyl-tRNA hydrolase PTRHD1	0.0012	down
XM_043764023.1	LOC122591784	disease resistance protein RPV1-like	< 0.0001	down
XM_043759302.1	LOC122587200	uncharacterized LOC122587200	0.0005	down
XM_043763058.1	LOC122590877	putative receptor-like protein kinase At5g39000	0.0001	down
XM_043757075.1	LOC122584981	uncharacterized LOC122584981	< 0.0001	down
XM_043781077.1	LOC122607995	acyl-protein thioesterase 2-like	0.0002	down
XM_043772537.1	LOC122599921	vacuolar-processing enzyme-like	0.0001	down
XM_043753373.1	LOC122581203	uncharacterized LOC122581203	0.0013	down
XM_043780793.1	LOC122607761	uncharacterized LOC122607761	0.0011	down
XM_043771649.1	LOC122599187	protein NRT1/ PTR FAMILY 6.2	0.0005	down
XM_043766828.1	LOC122594362	UPF0496 protein At4g34320-like	0.0003	down
XM_043777283.1	LOC122604386	protein SRC2-like	< 0.0001	down
XM_043783468.1	LOC122610481	receptor-like kinase TMK4	< 0.0001	down
XM_043773650.1	LOC122600868	zinc finger CCCH domain-containing protein 29-like	< 0.0001	down
XM_043769751.1	LOC122597113	phosphoinositide phospholipase C 2-like, transcript variant X1	< 0.0001	down
XM_043764454.1	LOC122592264	cysteine-rich receptor-like protein kinase 29	0.0007	down
XM_043757792.1	LOC122585665	probable E3 ubiquitin-protein ligase ZFP1, transcript variant X3	< 0.0001	down
XM_043768411.1	LOC122595933	probable carboxylesterase 6	< 0.0001	down
XM_043779939.1	LOC122607021	cytochrome P450 81Q32-like	< 0.0001	down
XM_043775049.1	LOC122602354	ethylene-responsive transcription factor ERF014-like	< 0.0001	down
XM_043750239.1	LOC122578307	vacuolar-processing enzyme-like	0.0013	down
XM_043755440.1	LOC122583007	agamous-like MADS-box protein AGL62	0.0002	down
XM_043750110.1	LOC122578202	putative glucose-6-phosphate 1-epimerase	0.0007	down
XM_043754618.1	LOC122582251	enolase	0.0011	down
XM_043751908.1	LOC122579686	brassinosteroid-responsive RING protein 1-like	0.0012	down
XM_043761133.1	LOC122588915	probable serine/threonine-protein kinase PBL17	0.0005	down
XM_043767988.1	LOC122595592	germin-like protein 9-3	< 0.0001	down
XM_043776036.1	LOC122603360	8-hydroxygeraniol dehydrogenase-like	< 0.0001	down
XM_043781812.1	LOC122608718	protein NRT1/ PTR FAMILY 1.2-like	0.0010	down
XM_043779690.1	LOC122606823	cell division protein FtsZ homolog 2-1, chloroplastic-like, transcript variant X1	< 0.0001	down
XM_043758814.1	LOC122586820	synaptotagmin-2-like, transcript variant X2	< 0.0001	down
XM_043767175.1	LOC122594874	probable alpha,alpha-trehalose-phosphate synthase [UDP-forming] 7	0.0006	down
XM_043773860.1	LOC122601086	probable CoA ligase CCL5	< 0.0001	down
XM_043756380.1	LOC122584015	probable protein phosphatase 2C 25	0.0005	down
XM_043784039.1	LOC122611077	polyubiquitin 11-like	< 0.0001	down
XM_043768436.1	LOC122595950	probable protein phosphatase 2C 59, transcript variant X3	0.0004	down
XR_006322008.1	LOC122586472	uncharacterized LOC122586472	0.0005	down
XM_043773922.1	LOC122601153	receptor-like protein kinase FERONIA	0.0003	down
XM_043779421.1	LOC122606581	hyoscyamine 6-dioxygenase-like	< 0.0001	down
XM_043761379.1	LOC122589137	transcription factor MYB25-like	< 0.0001	down
XM_043759324.1	LOC122587217	uncharacterized LOC122587217, transcript variant X3	< 0.0001	down
XM_043770729.1	LOC122598126	auxin response factor 18-like	< 0.0001	down
XM_043753753.1	LOC122581524	zinc finger protein ZAT1-like	0.0004	down
XM_043752365.1	LOC122580093	protein SCAI, transcript variant X2	0.0005	down
XM_043780897.1	LOC122607839	sulfate transporter 1.3-like, transcript variant X1	0.0003	down
XM_043750389.1	LOC122578425	RHOMBOID-like protein 2	< 0.0001	down
XR_006320989.1	LOC122581230	micronuclear linker histone polyprotein, transcript variant X3	0.0002	down
XR_006325200.1	LOC122608287	uncharacterized LOC122608287, transcript variant X1	0.0007	down
XM_043781078.1	LOC122607998	acyl-coenzyme A thioesterase 13-like	0.0013	down
XM_043775047.1	LOC122602349	protein DMP3-like	0.0003	down
XM_043754615.1	LOC122582248	WAT1-related protein At5g07050	< 0.0001	down
XM_043776393.1	LOC122603632	myb-related protein 2-like, transcript variant X1	0.0002	down
XM_043776432.1	LOC122603663	probable inactive receptor kinase At5g58300, transcript variant X2	0.0003	down
XM_043770952.1	LOC122598360	CSC1-like protein At3g54510	< 0.0001	down
XM_043759425.1	LOC122587298	nudix hydrolase 17, mitochondrial-like	0.0003	down
XM_043784145.1	LOC122611170	glycerate dehydrogenase	< 0.0001	down
XM_043776739.1	LOC122603893	scarecrow-like protein 8	< 0.0001	down
XM_043779878.1	LOC122606972	protein MICRORCHIDIA 6-like, transcript variant X3	< 0.0001	down
XM_043755138.1	LOC122582715	probable protein phosphatase 2C 25	0.0001	down
XM_043778935.1	LOC122605971	ethylene-responsive transcription factor ERF027-like	< 0.0001	down

Transcript id	GeneID	Gene name/function	*P* value	result
XM_043755005.1	LOC122582591	MACPF domain-containing protein At4g24290-like	0.0008	down
XM_043775323.1	LOC122602734	receptor protein kinase CLAVATA1-like	< 0.0001	down
XM_043767574.1	LOC122595241	C2 domain-containing protein At1g53590-like	< 0.0001	down
XM_043762573.1	LOC122590242	thiol:disulfide interchange protein TxlA homolog	< 0.0001	down
XM_043779728.1	LOC122606851	phospholipase D alpha 1-like	0.0005	down
XM_043759964.1	LOC122587798	U-box domain-containing protein 40	< 0.0001	down
XM_043774482.1	LOC122601742	glycine-rich cell wall structural protein 1.8-like	0.0009	down
XM_043757279.1	LOC122585172	BOI-related E3 ubiquitin-protein ligase 1-like, transcript variant X2	< 0.0001	down
XM_043776045.1	LOC122603367	putative lipid-transfer protein DIR1	< 0.0001	down
XM_043760645.1	LOC122588511	chlorophyllide a oxygenase, chloroplastic-like	0.0003	down
XM_043761886.1	LOC122589581	subtilisin-like protease SBT1.7	< 0.0001	down
XM_043755438.1	LOC122583001	CBL-interacting serine/threonine-protein kinase 7-like	< 0.0001	down
XM_043750685.1	LOC122578668	homeobox-leucine zipper protein ATHB-7-like	0.0006	down
XM_043756248.1	LOC122583874	chaperone protein dnaJ 20, chloroplastic-like	0.0004	down
XM_043773258.1	LOC122600530	L-type lectin-domain containing receptor kinase IX.1-like	0.0003	down
XM_043753432.1	LOC122581249	protein TRANSPARENT TESTA GLABRA 1	< 0.0001	down
XR_006320514.1	LOC122578484	uncharacterized LOC122578484, transcript variant X4	0.0002	down
XM_043766334.1	LOC122593880	heavy metal-associated isoprenylated plant protein 47-like	0.0007	down
XM_043763567.1	LOC122591313	beta-amylase 2, chloroplastic	0.0011	down
XM_043768149.1	LOC122595720	BES1/BZR1 homolog protein 4-like, transcript variant X2	< 0.0001	down
XM_043759220.1	LOC122587132	serine--tRNA ligase, chloroplastic/mitochondrial, transcript variant X1	0.0007	down
XM_043763541.1	LOC122591300	plasma membrane ATPase 4	0.0001	down
XM_043758092.1	LOC122585977	uncharacterized LOC122585977, transcript variant X2	< 0.0001	down
XM_043766138.1	LOC122593693	leucine-rich repeat extensin-like protein 3	0.0001	down
XM_043756732.1	LOC122584685	E3 ubiquitin-protein ligase RHA2A-like	0.0007	down
XM_043766285.1	LOC122593836	alanine--glyoxylate aminotransferase 2 homolog 3, mitochondrial-like	< 0.0001	down
XM_043773347.1	LOC122600598	uncharacterized LOC122600598	0.0003	down
XM_043779905.1	LOC122606989	aldehyde oxidase GLOX	0.0002	down
XM_043784025.1	LOC122611068	RHOMBOID-like protein 2	< 0.0001	down
XM_043765877.1	LOC122593464	xyloglucan endotransglucosylase/hydrolase 2-like	0.0005	down
XM_043780803.1	LOC122607766	transcription factor BIM1-like, transcript variant X1	< 0.0001	down
XM_043770895.1	LOC122598304	probable galacturonosyltransferase-like 9	< 0.0001	down
XR_006321026.1	LOC122581634	uncharacterized LOC122581634, transcript variant X1	0.0006	down
XM_043780942.1	LOC122607872	uncharacterized oxidoreductase At4g09670-like	< 0.0001	down
XM_043775399.1	LOC122602803	cation/H(+) antiporter 20	< 0.0001	down
XM_043752981.1	LOC122580820	ferritin-3, chloroplastic-like	0.0003	down
XR_006320699.1	LOC122579755	uncharacterized LOC122579755, transcript variant X7	0.0007	down
XM_043758226.1	LOC122586117	late embryogenesis abundant protein D-34-like	0.0001	down
XM_043757250.1	LOC122585145	uncharacterized LOC122585145	0.0010	down
XM_043754831.1	LOC122582442	clavaminate synthase-like protein At3g21360	0.0011	down
XM_043776577.1	LOC122603776	xyloglucan galactosyltransferase XLT2	< 0.0001	down
XM_043775154.1	LOC122602542	probable LRR receptor-like serine/threonine-protein kinase At5g63710, transcript variant X1	< 0.0001	down
XM_043770644.1	LOC122598040	lysine histidine transporter-like 6	0.0006	down
XM_043778731.1	LOC122605775	putative lipid-transfer protein DIR1	< 0.0001	down
XM_043768646.1	LOC122596125	LOB domain-containing protein 37-like	0.0012	down
XM_043761104.1	LOC122588884	aspartyl protease family protein At5g10770-like	0.0002	down
XM_043775293.1	LOC122602702	uncharacterized LOC122602702, transcript variant X1	0.0001	down
XM_043776904.1	LOC122604020	type I inositol polyphosphate 5-phosphatase 10	0.0004	down
XR_006321955.1	LOC122586330	uncharacterized LOC122586330	0.0001	down
XM_043783652.1	LOC122610683	putative disease resistance RPP13-like protein 1	0.0004	down
XR_006320814.1	LOC122580529	uncharacterized LOC122580529	0.0002	down
XR_006324602.1	LOC122605362	zinc finger CCCH domain-containing protein 41, transcript variant X3	0.0009	down
XM_043772526.1	LOC122599914	uncharacterized LOC122599914, transcript variant X1	< 0.0001	down
XM_043776694.1	LOC122603861	wound-induced protein 1	< 0.0001	down
XR_006320813.1	LOC122580519	UDP-glycosyltransferase 89A2-like, transcript variant X1	< 0.0001	down
XM_043778816.1	LOC122605859	serine/threonine-protein kinase UCN-like	< 0.0001	down
XM_043776822.1	LOC122603954	zinc finger CCCH domain-containing protein 29-like	0.0002	down
XM_043780001.1	LOC122607082	uncharacterized LOC122607082	< 0.0001	down
XM_043778865.1	LOC122605905	ATP-dependent RNA helicase-like protein DB10	0.0008	down
XM_043761559.1	LOC122589285	zinc finger protein ZAT10-like	< 0.0001	down
XM_043769407.1	LOC122596777	probable indole-3-acetic acid-amido synthetase GH3.1	< 0.0001	down

Transcript id	GeneID	Gene name/function	*P* value	result
XM_043758137.1	LOC122586022	malonyl-coenzyme A:anthocyanin 3-O-glucoside-6"-O-malonyltransferase-like	< 0.0001	down
XM_043761194.1	LOC122588968	cytochrome P450 78A5-like	< 0.0001	down
XM_043776892.1	LOC122604010	histidine kinase 1-like, transcript variant X2	0.0011	down
XM_043752320.1	LOC122580047	uncharacterized LOC122580047, transcript variant X1	0.0006	down
XM_043783963.1	LOC122611012	uncharacterized LOC122611012	0.0008	down
XM_043750014.1	LOC122578126	fructose-bisphosphate aldolase 1, chloroplastic-like	< 0.0001	down
XM_043772811.1	LOC122600143	alpha,alpha-trehalose-phosphate synthase [UDP-forming] 1, transcript variant X1	< 0.0001	down
XM_043783835.1	LOC122610878	uncharacterized LOC122610878	0.0003	down
XM_043760919.1	LOC122588744	receptor-like protein kinase FERONIA, transcript variant X2	< 0.0001	down
XM_043781587.1	LOC122608483	RING-H2 finger protein ATL3-like, transcript variant X2	0.0001	down
XM_043758990.1	LOC122586950	U-box domain-containing protein 4, transcript variant X4	0.0002	down
XM_043780201.1	LOC122607265	adenylate isopentenyltransferase 3, chloroplastic	< 0.0001	down
XM_043765192.1	LOC122592873	ethylene-responsive transcription factor 4-like	< 0.0001	down
XM_043751982.1	LOC122579753	thioredoxin H-type-like	0.0001	down
XM_043758386.1	LOC122586391	dehydration-responsive element-binding protein 1D-like	< 0.0001	down
XM_043758085.1	LOC122585971	proline-rich receptor-like protein kinase PERK1	< 0.0001	down
XM_043767215.1	LOC122594930	calmodulin-binding transcription activator 4-like, transcript variant X1	< 0.0001	down
XM_043758585.1	LOC122586607	protein COBRA-like	0.0006	down
XM_043780826.1	LOC122607783	uncharacterized LOC122607783, transcript variant X2	0.0003	down
XM_043771957.1	LOC122599436	phosphatidylinositol 4-phosphate 5-kinase 9-like, transcript variant X1	0.0013	down
XM_043753421.1	LOC122581240	ABC transporter C family member 4-like	< 0.0001	down
XM_043775083.1	LOC122602392	uncharacterized LOC122602392	0.0005	down
XR_006323941.1	LOC122599733	uncharacterized LOC122599733	0.0005	down
XM_043768355.1	LOC122595883	zinc transporter 5-like	0.0003	down
XM_043769275.1	LOC122596657	uncharacterized LOC122596657, transcript variant X1	0.0002	down
XM_043763568.1	LOC122591314	RNA polymerase II C-terminal domain phosphatase-like 1	0.0003	down
XM_043767900.1	LOC122595529	pectinesterase 1-like	0.0009	down
XM_043783094.1	LOC122610101	probable serine/threonine-protein kinase PBL19	0.0008	down
XM_043753881.1	LOC122581636	NAD-dependent protein deacylase SRT2-like, transcript variant X1	0.0006	down
XM_043770384.1	LOC122597809	U-box domain-containing protein 26-like	< 0.0001	down
XM_043758645.1	LOC122586665	choline/ethanolaminephosphotransferase 1-like, transcript variant X1	0.0012	down
XR_006322586.1	LOC122590718	uncharacterized LOC122590718	0.0010	down
XM_043763476.1	LOC122591242	VAN3-binding protein	0.0002	down
XM_043750947.1	LOC122578898	UDP-glycosyltransferase 72B1-like	< 0.0001	down
XM_043763251.1	LOC122591058	glycerophosphodiester phosphodiesterase GDPDL3-like	< 0.0001	down
XM_043773864.1	LOC122601092	cold-responsive protein kinase 1-like	0.0007	down
XM_043779442.1	LOC122606597	lipoxygenase 1, chloroplastic	< 0.0001	down
XM_043764972.1	LOC122592686	xyloglucan endotransglucosylase/hydrolase 2-like	< 0.0001	down
XM_043778640.1	LOC122605686	beta-fructofuranosidase, soluble isoenzyme I-like	< 0.0001	down
XM_043766204.1	LOC122593758	gamma-glutamyl peptidase 3-like	< 0.0001	down
XM_043763198.1	LOC122591013	alpha-glucan phosphorylase, H isozyme	< 0.0001	down
XM_043765249.1	LOC122592917	transmembrane protein 45A	< 0.0001	down
XM_043752211.1	LOC122579944	anthocyanidin 3-O-glucosyltransferase 5-like	0.0002	down
XM_043779517.1	LOC122606676	ABC transporter C family member 13, transcript variant X1	< 0.0001	down
XM_043763261.1	LOC122591065	COP1-interacting protein 7, transcript variant X1	< 0.0001	down
XM_043759417.1	LOC122587291	F-box protein SKIP2-like	< 0.0001	down
XM_043752474.1	LOC122580198	monosaccharide-sensing protein 2-like	0.0005	down
XM_043752555.1	LOC122580278	F-box protein CPR1-like	0.0008	down
XM_043761081.1	LOC122588866	thioredoxin H2-like, transcript variant X2	0.0006	down
XM_043763230.1	LOC122591041	protein EARLY RESPONSIVE TO DEHYDRATION 15	0.0002	down
XM_043776305.1	LOC122603570	biotin carboxyl carrier protein of acetyl-CoA carboxylase-like, transcript variant X1	< 0.0001	down
XM_043772634.1	LOC122600004	uncharacterized LOC122600004	0.0003	down
XM_043759196.1	LOC122587116	dehydration-responsive element-binding protein 1E-like	< 0.0001	down
XM_043752625.1	LOC122580354	ethylene-responsive transcription factor ERF027-like	< 0.0001	down
XM_043774409.1	LOC122601667	probable leucine-rich repeat receptor-like protein kinase At1g35710	< 0.0001	down
XM_043780011.1	LOC122607091	coiled-coil domain-containing protein SCD2, transcript variant X2	< 0.0001	down
XM_043771792.1	LOC122599298	auxin response factor 9, transcript variant X1	< 0.0001	down
XM_043753433.1	LOC122581250	probable disease resistance protein At5g66900, transcript variant X2	< 0.0001	down
XM_043769428.1	LOC122596789	WAT1-related protein At2g39510-like	< 0.0001	down
XM_043752733.1	LOC122580462	exocyst complex component EXO70B1-like	0.0013	down
XM_043775510.1	LOC122602898	beta-glucosidase BoGH3B-like	< 0.0001	down

Transcript id	GeneID	Gene name/function	*P* value	result
XM_043764258.1	LOC122592046	ethylene-responsive transcription factor ERF017-like	< 0.0001	down
XM_043754657.1	LOC122582288	universal stress protein YxiE-like	< 0.0001	down
XM_043767244.1	LOC122594960	dammarenediol II synthase-like	< 0.0001	down
XM_043781893.1	LOC122608812	probable serine/threonine-protein kinase tsuA	0.0013	down
XM_043753649.1	LOC122581419	receptor like protein kinase S.2-like	< 0.0001	down
XM_043784155.1	LOC122611177	probable E3 ubiquitin-protein ligase RZFP34	0.0002	down
XM_043759287.1	LOC122587187	probable disease resistance protein At5g66900, transcript variant X1	< 0.0001	down
XM_043781173.1	LOC122608087	protein EXORDIUM-like 2	< 0.0001	down
XM_043776835.1	LOC122603965	F-box protein At5g49610-like, transcript variant X2	< 0.0001	down
XM_043776247.1	LOC122603522	nematode resistance protein-like HSPRO2	< 0.0001	down
XM_043750442.1	LOC122578470	U-box domain-containing protein 4-like	0.0006	down
XM_043779893.1	LOC122606979	N-terminal acetyltransferase B complex auxiliary subunit NAA25, transcript variant X1	< 0.0001	down
XM_043774674.1	LOC122601946	uncharacterized LOC122601946	0.0010	down
XM_043770704.1	LOC122598098	cyclin-SDS	< 0.0001	down
XM_043772532.1	LOC122599917	U-box domain-containing protein 33-like, transcript variant X4	< 0.0001	down
XM_043754414.1	LOC122582065	polyamine oxidase 1	< 0.0001	down
XM_043752015.1	LOC122579780	protein PHOSPHATE-INDUCED 1-like	0.0002	down
XM_043782783.1	LOC122609835	serine--tRNA ligase-like, transcript variant X3	0.0011	down
XM_043753931.1	LOC122581680	phosphoinositide phospholipase C 2-like	< 0.0001	down
XM_043767813.1	LOC122595451	probable clathrin assembly protein At4g32285	0.0006	down
XM_043759164.1	LOC122587089	zinc finger CCCH domain-containing protein 29-like	0.0001	down
XM_043769283.1	LOC122596664	B-box zinc finger protein 22-like	0.0013	down
XM_043757870.1	LOC122585741	uncharacterized LOC122585741	0.0013	down
XM_043761567.1	LOC122589291	1-aminocyclopropane-1-carboxylate synthase 9-like	< 0.0001	down
XM_043754207.1	LOC122581892	PRA1 family protein B3-like	0.0001	down
XM_043756840.1	LOC122584781	U-box domain-containing protein 19	0.0002	down
XM_043770245.1	LOC122597672	serine/arginine repetitive matrix protein 1-like	0.0004	down
XM_043758683.1	LOC122586699	uncharacterized LOC122586699	< 0.0001	down
XM_043767260.1	LOC122594977	probable linoleate 9S-lipoxygenase 5	< 0.0001	down
XM_043764599.1	LOC122592386	scarecrow-like transcription factor PAT1	0.0004	down
XM_043760042.1	LOC122587882	ethylene-responsive transcription factor ERF014-like	< 0.0001	down
XM_043769302.1	LOC122596684	kirola-like	0.0007	down
XM_043774070.1	LOC122601301	aluminum-activated malate transporter 10-like, transcript variant X2	0.0008	down
XM_043770858.1	LOC122598256	S-type anion channel SLAH1-like	0.0010	down
XM_043778688.1	LOC122605728	pleiotropic drug resistance protein 1-like, transcript variant X1	< 0.0001	down
XM_043754864.1	LOC122582470	CBL-interacting protein kinase 2-like	0.0006	down
XM_043776258.1	LOC122603532	protein NRT1/ PTR FAMILY 5.8	0.0009	down
XM_043766351.1	LOC122593899	probable hexosyltransferase MUCI70, transcript variant X2	0.0005	down
XM_043773510.1	LOC122600753	uncharacterized LOC122600753	0.0011	down
XM_043776552.1	LOC122603757	syntaxin-61, transcript variant X1	0.0008	down
XM_043751331.1	LOC122579206	probable serine/threonine-protein kinase PBL28, transcript variant X1	< 0.0001	down
XM_043776961.1	LOC122604061	uncharacterized LOC122604061	< 0.0001	down
XM_043781941.1	LOC122608864	probable protein phosphatase 2C 49	0.0003	down
XM_043757440.1	LOC122585312	capsanthin/capsorubin synthase, chromoplastic-like	0.0005	down
XM_043759158.1	LOC122587083	uncharacterized LOC122587083	0.0001	down
XM_043778437.1	LOC122605482	NDR1/HIN1-like protein 6	0.0002	down
XM_043767583.1	LOC122595247	probable ubiquitin-conjugating enzyme E2 24, transcript variant X1	0.0002	down
XM_043760680.1	LOC122588547	serine/threonine-protein kinase STY13-like	0.0003	down
XM_043754663.1	LOC122582292	LRR receptor-like serine/threonine-protein kinase ERL1	0.0005	down
XM_043752559.1	LOC122580282	senescence/dehydration-associated protein At3g51250-like	< 0.0001	down
XM_043755297.1	LOC122582863	uncharacterized LOC122582863	0.0012	down
XM_043759142.1	LOC122587069	dehydration-responsive element-binding protein 1E-like	< 0.0001	down
XM_043761315.1	LOC122589070	histidine-containing phosphotransfer protein 4, transcript variant X3	< 0.0001	down
XM_043766954.1	LOC122594507	glycine-rich protein A3-like	< 0.0001	down
XM_043760878.1	LOC122588708	LRR receptor-like serine/threonine-protein kinase ERL1	0.0001	down
XM_043753569.1	LOC122581351	protein DETOXIFICATION 42-like, transcript variant X1	0.0007	down
XM_043754738.1	LOC122582360	germacrene A hydroxylase-like	< 0.0001	down
XM_043763534.1	LOC122591292	thiamine thiazole synthase, chloroplastic	< 0.0001	down
XR_006321197.1	LOC122582544	uncharacterized LOC122582544	< 0.0001	down
XM_043756209.1	LOC122583829	E3 ubiquitin-protein ligase PUB22-like	0.0001	down
XM_043773482.1	LOC122600728	protein SRC2 homolog	0.0003	down
XM_043766967.1	LOC122594521	vacuolar iron transporter homolog 4-like	< 0.0001	down
XM_043781019.1	LOC122607941	A-kinase anchor protein 7, transcript variant X1	0.0001	down

Transcript id	GeneID	Gene name/function	*P* value	result
XR_006325502.1	LOC122611282	uncharacterized LOC122611282	< 0.0001	down
XM_043783811.1	LOC122610852	ethylene-responsive transcription factor CRF2-like	< 0.0001	down
XM_043768903.1	LOC122596340	vacuolar iron transporter 1	0.0008	down
XM_043763582.1	LOC122591321	protein LONG AFTER FAR-RED 3, transcript variant X1	0.0007	down
XM_043783306.1	LOC122610310	GATA transcription factor 8-like	0.0008	down
XM_043765665.1	LOC122593276	cytochrome P450 710A11-like	0.0008	down
XM_043775722.1	LOC122603105	U-box domain-containing protein 4-like, transcript variant X1	0.0012	down
XM_043779217.1	LOC122606252	uncharacterized LOC122606252	< 0.0001	down
XM_043765081.1	LOC122592772	cytochrome b561 and DOMON domain-containing protein At5g35735-like	< 0.0001	down
XM_043750722.1	LOC122578704	transcription factor TCP15-like	< 0.0001	down
XM_043759461.1	LOC122587327	cyclin-dependent kinase F-4-like	< 0.0001	down
XM_043751533.1	LOC122579368	DNA (cytosine-5)-methyltransferase 1-like, transcript variant X2	< 0.0001	down
XM_043778046.1	LOC122605156	ethylene-responsive transcription factor ERF023-like	< 0.0001	down
XM_043756863.1	LOC122584799	probable E3 ubiquitin-protein ligase RHA4A	0.0004	down
XM_043764902.1	LOC122592622	adenylate isopentenyltransferase 3, chloroplastic-like	0.0003	down
XR_006325194.1	LOC122608261	uncharacterized LOC122608261	< 0.0001	down
XM_043779262.1	LOC122606304	subtilisin-like protease SBT1.3	< 0.0001	down
XM_043782605.1	LOC122609575	protein SABRE, transcript variant X2	< 0.0001	down
XM_043750644.1	LOC122578640	transmembrane protein 53	< 0.0001	down
XM_043776506.1	LOC122603722	receptor-like protein EIX2	< 0.0001	down
XM_043763505.1	LOC122591267	phospholipase A1 PLIP2, chloroplastic-like	0.0003	down
XM_043779190.1	LOC122606227	agamous-like MADS-box protein AGL62	0.0002	down
XM_043771469.1	LOC122599022	ethylene-responsive transcription factor ERF017-like	< 0.0001	down
XM_043759453.1	LOC122587317	EP1-like glycoprotein 3	< 0.0001	down
XM_043782895.1	LOC122609941	cellulose synthase-like protein D2	0.0004	down
XM_043754001.1	LOC122581733	myb-related protein 2, transcript variant X2	< 0.0001	down
XM_043780692.1	LOC122607673	protein OCTOPUS	< 0.0001	down
XM_043761304.1	LOC122589061	lysine-rich arabinogalactan protein 17-like	< 0.0001	down
XM_043778203.1	LOC122605294	ethylene-responsive transcription factor CRF4-like	0.0012	down
XM_043767990.1	LOC122595597	E3 ubiquitin-protein ligase BRE1-like 1, transcript variant X2	< 0.0001	down
XM_043750900.1	LOC122578852	acyl-CoA-binding protein	0.0002	down
XM_043755468.1	LOC122583032	cation/H(+) antiporter 20-like, transcript variant X1	0.0001	down
XM_043772831.1	LOC122600161	G patch domain-containing protein 8, transcript variant X1	< 0.0001	down
XM_043754680.1	LOC122582311	glutaredoxin-C13-like	< 0.0001	down
XM_043758497.1	LOC122586514	NAC domain-containing protein 91-like, transcript variant X1	< 0.0001	down
XM_043782326.1	LOC122609269	probable protein phosphatase 2C 2	0.0003	down
XM_043765116.1	LOC122592802	nuclear cap-binding protein subunit 1, transcript variant X1	0.0007	down
XM_043755170.1	LOC122582742	23 kDa jasmonate-induced protein-like	0.0003	down
XM_043779379.1	LOC122606524	E3 ubiquitin-protein ligase HEL2-like	< 0.0001	down
XM_043762400.1	LOC122590067	classical arabinogalactan protein 2-like	0.0008	down
XM_043773992.1	LOC122601231	adenylate isopentenyltransferase 5, chloroplastic	< 0.0001	down
XM_043759555.1	LOC122587397	transcription factor bHLH110-like, transcript variant X1	0.0003	down
XM_043765911.1	LOC122593493	cytochrome P450 78A9-like	< 0.0001	down
XM_043758786.1	LOC122586800	type IV inositol polyphosphate 5-phosphatase 3-like, transcript variant X1	< 0.0001	down
XM_043767295.1	LOC122595016	beta-xylosidase/alpha-L-arabinofuranosidase 2-like	0.0002	down
XM_043777136.1	LOC122604245	receptor-like protein EIX2	0.0004	down
XM_043767740.1	LOC122595388	disease resistance protein RUN1-like	< 0.0001	down
XM_043778012.1	LOC122605128	E3 ubiquitin-protein ligase UPL6, transcript variant X1	< 0.0001	down
XM_043784083.1	LOC122611115	PH-interacting protein, transcript variant X1	< 0.0001	down
XM_043765157.1	LOC122592839	uracil phosphoribosyltransferase	0.0012	down
XM_043750300.1	LOC122578353	probable amino acid permease 7, transcript variant X2	0.0005	down
XM_043768517.1	LOC122596020	caffeoyl-CoA O-methyltransferase 1	< 0.0001	down
XM_043757904.1	LOC122585781	oleosin L-like	0.0003	down
XM_043765612.1	LOC122593230	cytochrome b561 and DOMON domain-containing protein At3g25290	0.0001	down
XM_043761663.1	LOC122589373	protein BRASSINAZOLE-RESISTANT 1-like	0.0006	down
XM_043780415.1	LOC122607439	uncharacterized LOC122607439, transcript variant X1	< 0.0001	down
XM_043777778.1	LOC122604920	receptor kinase-like protein Xa21	0.0007	down
XM_043770537.1	LOC122597944	probable receptor-like protein kinase At4g10390	< 0.0001	down
XM_043763352.1	LOC122591136	probable pyruvate, phosphate dikinase regulatory protein, chloroplastic, transcript variant X5	< 0.0001	down
XM_043784239.1	LOC122611249	putative disease resistance RPP13-like protein 1	0.0013	down
XM_043757227.1	LOC122585127	leucine-rich repeat protein 1-like	< 0.0001	down

Transcript id	GeneID	Gene name/function	*P* value	result
XM_043762759.1	LOC122590562	tetraspanin-2-like	0.0004	down
XM_043766015.1	LOC122593585	glycine-rich cell wall structural protein 2	< 0.0001	down
XM_043753056.1	LOC122580911	high-affinity nitrate transporter 2.1-like	0.0002	down
XM_043756331.1	LOC122583962	probable LRR receptor-like serine/threonine-protein kinase RFK1, transcript variant X2	0.0002	down
XM_043750748.1	LOC122578724	prolyl endopeptidase-like	< 0.0001	down
XM_043767894.1	LOC122595527	delta(24)-sterol reductase, transcript variant X2	0.0007	down
XM_043778695.1	LOC122605740	probable disease resistance protein At5g66900, transcript variant X1	0.0011	down
XM_043774459.1	LOC122601716	nucleobase-ascorbate transporter 6-like	< 0.0001	down
XM_043768778.1	LOC122596232	lipid phosphate phosphatase 2-like, transcript variant X1	0.0005	down
XM_043763540.1	LOC122591298	phosphoinositide phospholipase C 4-like	0.0007	down
XM_043753688.1	LOC122581455	metallothionein-like protein 1	< 0.0001	down
XM_043779272.1	LOC122606314	probable disease resistance protein At5g66900	0.0006	down
XM_043761241.1	LOC122589012	uncharacterized LOC122589012	< 0.0001	down
XM_043764480.1	LOC122592286	formin-like protein 18, transcript variant X5	0.0007	down
XM_043782841.1	LOC122609887	beta-1,4-mannosyl-glycoprotein 4-beta-N-acetylglucosaminyltransferase-like	< 0.0001	down
XM_043773237.1	LOC122600509	UDP-glucuronate 4-epimerase 1	< 0.0001	down
XM_043780372.1	LOC122607406	cell division cycle 20.2, cofactor of APC complex-like, transcript variant X4	< 0.0001	down
XM_043763075.1	LOC122590897	protein transport protein SEC31 homolog B-like, transcript variant X2	0.0003	down
XM_043772507.1	LOC122599900	MAPK kinase substrate protein At1g80180	0.0002	down
XM_043756200.1	LOC122583820	calcium uniporter protein 6, mitochondrial-like	< 0.0001	down
XM_043773039.1	LOC122600331	probable WRKY transcription factor 53, transcript variant X2	< 0.0001	down
XM_043760908.1	LOC122588735	homeobox-leucine zipper protein HAT5	0.0011	down
XM_043776631.1	LOC122603812	probable amino-acid acetyltransferase NAGS2, chloroplastic	0.0011	down
XM_043776303.1	LOC122603568	lipid phosphate phosphatase delta-like	0.0003	down
XM_043750514.1	LOC122578536	protein NRT1/ PTR FAMILY 7.3	< 0.0001	down
XM_043768917.1	LOC122596352	uncharacterized LOC122596352	< 0.0001	down
XM_043773012.1	LOC122600306	zinc finger protein ZAT10-like	< 0.0001	down
XM_043757294.1	LOC122585185	ethylene-responsive transcription factor ERF025-like	0.0001	down
XM_043775465.1	LOC122602857	phospholipase A1-Ibeta2, chloroplastic-like	< 0.0001	down
XM_043762630.1	LOC122590309	GDSL esterase/lipase At1g71250	0.0001	down
XM_043774828.1	LOC122602099	putative ripening-related protein 1	0.0001	down
XM_043773434.1	LOC122600680	indole-3-acetic acid-amido synthetase GH3.5-like	0.0006	down
XM_043756111.1	LOC122583732	cytochrome P450 94C1	0.0011	down
XR_006321751.1	LOC122585652	uncharacterized LOC122585652, transcript variant X2	0.0006	down
XM_043761043.1	LOC122588832	queuosine salvage protein-like	< 0.0001	down
XM_043767111.1	LOC122594688	protein LURP-one-related 5-like	< 0.0001	down
XM_043759360.1	LOC122587247	F-box protein At1g78280	0.0008	down
XM_043775953.1	LOC122603285	probable LRR receptor-like serine/threonine-protein kinase At1g67720	0.0012	down
XM_043783181.1	LOC122610188	23 kDa jasmonate-induced protein-like	< 0.0001	down
XM_043770684.1	LOC122598073	oxysterol-binding protein-related protein 4C	< 0.0001	down
XM_043766494.1	LOC122594018	short-chain dehydrogenase reductase 3b-like	< 0.0001	down
XM_043780045.1	LOC122607126	extensin-like, transcript variant X1	0.0002	down
XM_043750429.1	LOC122578458	cytochrome P450 78A5-like	< 0.0001	down
XM_043768323.1	LOC122595860	ferredoxin-like	< 0.0001	down
XM_043756017.1	LOC122583633	uncharacterized LOC122583633	0.0009	down
XM_043779784.1	LOC122606897	alanine--glyoxylate aminotransferase 2 homolog 2, mitochondrial-like	< 0.0001	down
XM_043752863.1	LOC122580598	blue copper protein-like	< 0.0001	down
XM_043762042.1	LOC122589717	TOM1-like protein 4	< 0.0001	down
XR_006320492.1	LOC122578247	uncharacterized LOC122578247, transcript variant X1	0.0013	down
XM_043770562.1	LOC122597967	MLO-like protein 14	< 0.0001	down
XM_043784020.1	LOC122611064	transcription factor MYC2-like	< 0.0001	down
XM_043776052.1	LOC122603372	probable aspartyl protease At4g16563	0.0011	down
XM_043763067.1	LOC122590888	molybdenum cofactor sulfurase, transcript variant X3	< 0.0001	down
XM_043767699.1	LOC122595348	splicing factor U2af large subunit A-like, transcript variant X4	< 0.0001	down
XM_043763043.1	LOC122590866	heat shock cognate 70 kDa protein-like	0.0007	down
XM_043779360.1	LOC122606502	G-type lectin S-receptor-like serine/threonine-protein kinase At1g11330, transcript variant X3	< 0.0001	down
XM_043776578.1	LOC122603777	probable glutamate carboxypeptidase AMP1	< 0.0001	down
XM_043750702.1	LOC122578683	probable galacturonosyltransferase-like 3	0.0005	down
XM_043757361.1	LOC122585243	heat stress transcription factor A-3-like	0.0009	down
XM_043753563.1	LOC122581346	COBRA-like protein 7	< 0.0001	down

Transcript id	GeneID	Gene name/function	P value	result
XM_043763504.1	LOC122591266	serine/threonine-protein kinase RIPK-like	< 0.0001	down
XM_043755943.1	LOC122583549	agamous-like MADS-box protein AGL62	0.0008	down
XM_043768262.1	LOC122595813	beta-1,4-mannosyl-glycoprotein 4-beta-N-acetylglucosaminyltransferase-like	< 0.0001	down
XR_006324587.1	LOC122605332	uncharacterized LOC122605332, transcript variant X1	0.0005	down
XM_043770540.1	LOC122597946	ethylene-responsive transcription factor 12-like	< 0.0001	down
XM_043771468.1	LOC122599021	auxin response factor 7-like	< 0.0001	down
XM_043767305.1	LOC122595026	vacuolar iron transporter homolog 4-like	< 0.0001	down
XM_043771466.1	LOC122599020	uncharacterized LOC122599020, transcript variant X1	< 0.0001	down
XM_043767306.1	LOC122595027	vacuolar iron transporter homolog 4-like	< 0.0001	down
XM_043765766.1	LOC122593371	protein TRANSPORT INHIBITOR RESPONSE 1-like	0.0005	down
XM_043777713.1	LOC122604843	E3 ubiquitin-protein ligase UPL7, transcript variant X1	0.0001	down
XM_043775173.1	LOC122602567	uncharacterized LOC122602567, transcript variant X1	0.0005	down
XM_043768579.1	LOC122596066	AP2/ERF and B3 domain-containing transcription factor At1g50680-like, transcript variant X1	0.0004	down
XM_043753872.1	LOC122581630	hsp70 nucleotide exchange factor fes1-like, transcript variant X2	< 0.0001	down
XM_043776373.1	LOC122603620	uncharacterized LOC122603620	< 0.0001	down
XM_043759156.1	LOC122587081	actin-depolymerizing factor 2	0.0004	down
XM_043763549.1	LOC122591304	uncharacterized LOC122591304, transcript variant X11	< 0.0001	down
XM_043752648.1	LOC122580377	BON1-associated protein 2-like	< 0.0001	down
XM_043762364.1	LOC122590027	transcription factor bHLH18-like	< 0.0001	down
XM_043780365.1	LOC122607400	nucleobase-ascorbate transporter 6-like	0.0002	down
XM_043781817.1	LOC122608724	E3 ubiquitin-protein ligase RNF144A, transcript variant X1	< 0.0001	down
XM_043784364.1	LOC122611343	S-adenosylmethionine decarboxylase proenzyme-like	< 0.0001	down
XM_043766001.1	LOC122593574	indole-3-acetic acid-amido synthetase GH3.5-like	0.0006	down
XM_043758384.1	LOC122586390	dehydration-responsive element-binding protein 1E-like	0.0002	down
XM_043767376.1	LOC122594706	copper-transporting ATPase HMA4-like	0.0002	down
XM_043755238.1	LOC122582801	E3 ubiquitin-protein ligase PUB23-like	0.0001	down
XM_043756874.1	LOC122584812	uncharacterized LOC122584812, transcript variant X2	< 0.0001	down
XM_043767638.1	LOC122595296	mitogen-activated protein kinase kinase 5	0.0002	down
XM_043772263.1	LOC122599711	paramyosin, transcript variant X1	0.0002	down
XM_043757379.1	LOC122585261	NAC domain-containing protein 90-like	< 0.0001	down
XM_043755141.1	LOC122582717	cell wall / vacuolar inhibitor of fructosidase 1-like	0.0007	down
XM_043752528.1	LOC122580251	protein DA1-related 3-like, transcript variant X2	< 0.0001	down
XM_043763394.1	LOC122591173	putative F-box protein At1g32420	0.0012	down
XR_006323999.1	LOC122600093	uncharacterized LOC122600093	0.0010	down
XM_043749975.1	LOC122578088	UDP-glucose 6-dehydrogenase 4-like, transcript variant X1	0.0002	down
XM_043778953.1	LOC122605988	pentatricopeptide repeat-containing protein At5g03800, transcript variant X3	0.0009	down
XM_043765626.1	LOC122593241	BTB/POZ domain-containing protein At4g30940	0.0012	down
XM_043783958.1	LOC122611006	glycine-rich protein 5-like	< 0.0001	down
XM_043776825.1	LOC122603957	E3 ubiquitin-protein ligase WAV3	0.0010	down
XM_043767304.1	LOC122595024	vacuolar iron transporter homolog 4-like	< 0.0001	down
XM_043751027.1	LOC122578961	proline dehydrogenase 2, mitochondrial-like	< 0.0001	down
XM_043774699.1	LOC122601966	auxin-responsive protein IAA16-like	< 0.0001	down
XM_043757308.1	LOC122585197	delta(7)-sterol-C5(6)-desaturase-like	0.0008	down
XM_043756713.1	LOC122584668	WAT1-related protein At1g70260	< 0.0001	down
XM_043753351.1	LOC122581184	putative disease resistance RPP13-like protein 1, transcript variant X2	0.0001	down
XM_043779023.1	LOC122606058	inverted formin-2-like	0.0002	down
XM_043751970.1	LOC122579743	uncharacterized LOC122579743	0.0002	down
XM_043771499.1	LOC122599048	probable magnesium transporter NIPA4, transcript variant X1	< 0.0001	down
XM_043782580.1	LOC122609552	LON peptidase N-terminal domain and RING finger protein 1-like, transcript variant X2	< 0.0001	down
XM_043764845.1	LOC122592583	helicase-like transcription factor CHR28	0.0008	down
XM_043760893.1	LOC122588721	protein RADIALIS-like 3	< 0.0001	down
XM_043776319.1	LOC122603581	patatin-like protein 7	0.0002	down
XM_043750267.1	LOC122578329	probable CCR4-associated factor 1 homolog 11	0.0003	down
XM_043753906.1	LOC122581660	F-box/kelch-repeat protein At1g57790-like	0.0009	down
XM_043754323.1	LOC122581987	phosphomethylpyrimidine synthase, chloroplastic	< 0.0001	down
XM_043776275.1	LOC122603549	protein DA1-related 2-like	0.0004	down
XM_043778189.1	LOC122605280	phosphoethanolamine N-methyltransferase 3-like	0.0002	down
XM_043754754.1	LOC122582370	serine/arginine-rich SC35-like splicing factor SCL30A, transcript variant X2	0.0006	down
XM_043765742.1	LOC122593349	protein CYPRO4-like	0.0008	down
XR_006321641.1	LOC122585027	uncharacterized LOC122585027	0.0006	down

Transcript id	GeneID	Gene name/function	*P* value	result
XM_043758903.1	LOC122586886	calcium-transporting ATPase 2, plasma membrane-type-like	0.0010	down
XM_043773226.1	LOC122600499	E3 SUMO-protein ligase SIZ1, transcript variant X3	0.0005	down
XM_043762346.1	LOC122590008	putative UPF0481 protein At3g02645	< 0.0001	down
XM_043762954.1	LOC122590782	uncharacterized LOC122590782, transcript variant X1	0.0007	down
XM_043756849.1	LOC122584790	23 kDa jasmonate-induced protein-like	0.0002	down
XM_043757124.1	LOC122585025	lipid phosphate phosphatase 2-like	< 0.0001	down
XM_043750421.1	LOC122578450	glycine dehydrogenase (decarboxylating) A, mitochondrial	0.0006	down
XM_043756748.1	LOC122584700	dehydration-responsive element-binding protein 1D-like	< 0.0001	down
XM_043784191.1	LOC122611210	protein NRT1/ PTR FAMILY 4.6-like, transcript variant X1	< 0.0001	down
XM_043776827.1	LOC122603960	dehydration-responsive element-binding protein 1D-like	< 0.0001	down
XM_043764914.1	LOC122592632	trifunctional UDP-glucose 4,6-dehydratase/UDP-4-keto-6-deoxy-D-glucose 3,5-epimerase/UDP-4-keto-L-rhamnose-reductase RHM1-like	< 0.0001	down
XM_043770424.1	LOC122597849	probable LRR receptor-like serine/threonine-protein kinase At1g14390, transcript variant X4	0.0008	down
XM_043753597.1	LOC122581373	helicase protein MOM1-like, transcript variant X2	0.0004	down
XM_043774290.1	LOC122601535	probable E3 ubiquitin-protein ligase XERICO	0.0013	down
XM_043781150.1	LOC122608064	ER lumen protein-retaining receptor erd-2.2-like	0.0003	down
XM_043771703.1	**LOC122599226**	elongation factor 1-alpha-like, transcript variant X7	0.0011	down
XM_043771704.1	**LOC122599226**	elongation factor 1-alpha-like, transcript variant X7	< 0.0001	down
XM_043778507.1	**LOC122605549**	uncharacterized LOC122605549, transcript variant X1	0.0007	down
XM_043778508.1	**LOC122605549**	uncharacterized LOC122605549, transcript variant X1	< 0.0001	down
XM_043768751.1	**LOC122596212**	common plant regulatory factor 1-like, transcript variant X2	0.0012	down
XM_043768752.1	**LOC122596212**	common plant regulatory factor 1-like, transcript variant X2	< 0.0001	down
XM_043779126.1	**LOC122606166**	uncharacterized LOC122606166, transcript variant X2	< 0.0001	down
XM_043779129.1	**LOC122606166**	uncharacterized LOC122606166, transcript variant X2	< 0.0001	down
XM_043779125.1	**LOC122606166**	uncharacterized LOC122606166, transcript variant X2	< 0.0001	down
XR_006323120.1	**LOC122594836**	uncharacterized LOC122594836, transcript variant X2	< 0.0001	down
XR_006323117.1	**LOC122594836**	uncharacterized LOC122594836, transcript variant X2	< 0.0001	down
XR_006323401.1	**LOC122596995**	cyclin-dependent kinase G-2-like, transcript variant X6	0.0009	down
XM_043769639.1	**LOC122596995**	cyclin-dependent kinase G-2-like, transcript variant X6	0.0006	down
XM_043768365.1	**LOC122595892**	heptahelical transmembrane protein 1-like, transcript variant X1	0.0002	down
XM_043768364.1	**LOC122595892**	heptahelical transmembrane protein 1-like, transcript variant X1	0.0011	down
XM_043777470.1	**LOC122604587**	monosaccharide-sensing protein 2, transcript variant X2	< 0.0001	down
XM_043777474.1	**LOC122604587**	monosaccharide-sensing protein 2, transcript variant X2	0.0010	down
XM_043781002.1	**LOC122607929**	receptor-like protein kinase, transcript variant X2	0.0010	down
XM_043781003.1	**LOC122607929**	receptor-like protein kinase, transcript variant X2	0.0001	down
XM_043769452.1	**LOC122596808**	histone-lysine N-methyltransferase, H3 lysine-9 specific SUVH4-like, transcript variant X1	0.0002	down
XM_043769453.1	**LOC122596808**	histone-lysine N-methyltransferase, H3 lysine-9 specific SUVH4-like, transcript variant X1	< 0.0001	down
XM_043765644.1	**LOC122593258**	pentatricopeptide repeat-containing protein At5g39350, transcript variant X3	< 0.0001	up
XM_043765643.1	**LOC122593258**	pentatricopeptide repeat-containing protein At5g39350, transcript variant X3	< 0.0001	down
XM_043775549.1	**LOC122602936**	pleiotropic drug resistance protein 2-like, transcript variant X1	< 0.0001	up
XM_043775550.1	**LOC122602936**	pleiotropic drug resistance protein 2-like, transcript variant X1	0.0003	down
XM_043763512.1	**LOC122591271**	putative glucose-6-phosphate 1-epimerase, transcript variant X2	< 0.0001	up
XM_043763511.1	**LOC122591271**	putative glucose-6-phosphate 1-epimerase, transcript variant X2	0.0001	down

LC represents the low concentration treatment (1 mg/kg Cd and 90 mg/kg Pb). Locus IDs shown in bold indicate that more than one transcript is associated with the same gene.

Table S4 Differentially expressed genes (DEGs) identified in *Erigeron canadensis* roots under the MC treatment

Transcript id	GeneID	Gene name/function	*P* value	result
XM_043760959.1	LOC122588766	guanine nucleotide-binding protein subunit gamma 3	< 0.0001	up
XM_043762283.1	LOC122589952	50S ribosomal protein L29, chloroplastic	0.0037	up
XM_043766888.1	LOC122594434	protein disulfide isomerase-like 5-4	0.0006	up
XM_043754281.1	LOC122581947	7-methylguanosine phosphate-specific 5'-nucleotidase A	0.0001	up
XM_043763082.1	LOC122590906	rho GDP-dissociation inhibitor 1-like	0.0003	up
XR_006322304.1	LOC122589692	uncharacterized LOC122589692	0.0006	up
XM_043778095.1	LOC122605195	uridine kinase-like protein 1, chloroplastic	0.0053	up
XM_043756922.1	LOC122584851	protein SAMBA	0.0023	up
XM_043762317.1	LOC122589986	probable (S)-N-methylcoclaurine 3'-hydroxylase isozyme 2	0.0005	up
XM_043753855.1	LOC122581609	probable 6-phosphogluconolactonase 1	< 0.0001	up
XM_043755021.1	LOC122582607	ER lumen protein-retaining receptor-like	< 0.0001	up
XR_006323074.1	LOC122594699	small nucleolar RNA U3	0.0033	up
XM_043771343.1	LOC122598863	uncharacterized LOC122598863	0.0021	up
XM_043767439.1	LOC122595123	trafficking protein particle complex subunit 6B-like	0.0040	up
XM_043767348.1	LOC122595059	uncharacterized LOC122595059, transcript variant X3	0.0006	up
XM_043776279.1	LOC122603552	uncharacterized LOC122603552	0.0024	up
XM_043760253.1	LOC122588118	uncharacterized LOC122588118	< 0.0001	up
XM_043780917.1	LOC122607851	39S ribosomal protein L41-B, mitochondrial-like	< 0.0001	up
XM_043751192.1	LOC122579091	magnesium transporter MRS2-I-like, transcript variant X2	< 0.0001	up
XM_043780159.1	LOC122607223	uncharacterized LOC122607223	< 0.0001	up
XM_043751673.1	LOC122579491	39S ribosomal protein L41-A, mitochondrial-like	0.0014	up
XM_043778719.1	LOC122605763	F-box protein CPR1-like	0.0025	up
XR_006322793.1	LOC122593099	uncharacterized LOC122593099, transcript variant X4	0.0049	up
XM_043754846.1	LOC122582454	mitochondrial import inner membrane translocase subunit Tim9	< 0.0001	up
XM_043768966.1	LOC122596397	60S ribosomal protein L39-1	0.0005	up
XM_043776546.1	LOC122603752	protein NPGR2-like	0.0009	up
XM_043762945.1	LOC122590773	eukaryotic translation initiation factor 1A-like	0.0021	up
XM_043783026.1	LOC122610043	NADH dehydrogenase [ubiquinone] 1 beta subcomplex subunit 10-B	< 0.0001	up
XM_043772740.1	LOC122600082	spore coat polysaccharide biosynthesis protein SpsK	< 0.0001	up
XM_043770555.1	LOC122597960	36.4 kDa proline-rich protein-like	0.0009	up
XM_043775015.1	LOC122602316	flowering-promoting factor 1-like protein 3	< 0.0001	up
XM_043765830.1	LOC122593420	choline transporter protein 1	0.0008	up
XM_043784374.1	LOC122611354	CDPK-related protein kinase-like	0.0017	up
XM_043779385.1	LOC122606535	respirasome Complex Assembly Factor 1	< 0.0001	up
XM_043770688.1	LOC122598077	F-box protein PP2-A13-like	0.0017	up
XM_043776864.1	LOC122603988	cyclin-B1-2-like	< 0.0001	up
XM_043758930.1	LOC122586907	lecithin-cholesterol acyltransferase-like 1	0.0042	up
XM_043750126.1	LOC122578217	O-fucosyltransferase 31-like	0.0002	up
XM_043781075.1	LOC122607993	uncharacterized LOC122607993	0.0004	up
XM_043761142.1	LOC122588921	GDSL lipase-like	0.0041	up
XM_043778447.1	LOC122605493	uncharacterized LOC122605493	0.0002	up
XM_043778374.1	LOC122605423	transcription factor TCP20-like	0.0021	up
XM_043763328.1	LOC122591115	survival of motor neuron-related-splicing factor 30, transcript variant X1	0.0038	up
XM_043765241.1	LOC122592911	calcineurin subunit B-like	< 0.0001	up
XM_043757264.1	LOC122585157	1,4-dihydroxy-2-naphthoyl-CoA synthase, peroxisomal	< 0.0001	up
XM_043761117.1	LOC122588897	uncharacterized LOC122588897	0.0002	up
XM_043754523.1	LOC122582165	uncharacterized protein At4g13200, chloroplastic	0.0017	up
XM_043771758.1	LOC122599273	stress enhanced protein 1, chloroplastic	< 0.0001	up
XM_043765542.1	LOC122593170	uncharacterized LOC122593170	< 0.0001	up
XM_043772384.1	LOC122599799	probable enoyl-CoA hydratase 2, mitochondrial	0.0004	up
XM_043751625.1	LOC122579445	uncharacterized LOC122579445	0.0004	up
XM_043754469.1	LOC122582112	probable small nuclear ribonucleoprotein G	0.0005	up
XR_006321201.1	LOC122582593	uncharacterized LOC122582593, transcript variant X3	< 0.0001	up
XM_043776367.1	LOC122603616	protein GDAP2 homolog	< 0.0001	up
XM_043751443.1	LOC122579300	2,3-bisphosphoglycerate-dependent phosphoglycerate mutase 1-like, transcript variant X2	0.0038	up
XM_043758612.1	LOC122586629	serine/threonine-protein phosphatase PP1 isozyme 3, transcript variant X1	< 0.0001	up
XM_043757174.1	LOC122585077	homeobox protein knotted-1-like 6	0.0007	up
XM_043753776.1	LOC122581546	ATP synthase subunit d, mitochondrial	0.0048	up
XM_043766290.1	LOC122593843	uncharacterized LOC122593843	0.0034	up
XM_043750909.1	LOC122578860	cytochrome P450 93A3-like	0.0004	up
XM_043754436.1	LOC122582083	probable glutathione S-transferase	0.0002	up
XM_043760672.1	LOC122588538	60S ribosomal protein L36-3-like	< 0.0001	up
XM_043780490.1	LOC122607504	uncharacterized LOC122607504	0.0027	up
XM_043765284.1	LOC122592951	serine/threonine-protein phosphatase PP1 isozyme 2-like, transcript variant X2	0.0030	up

Transcript id	GeneID	Gene name/function	*P* value	result
XM_043756605.1	LOC122584442	NADH-ubiquinone oxidoreductase chain 3	0.0021	up
XM_043782199.1	LOC122609138	vesicle-associated protein 1-1-like	0.0002	up
XM_043774450.1	LOC122601707	60S ribosomal protein L30-like	< 0.0001	up
XM_043752327.1	LOC122580053	chaperone protein dnaJ 11, chloroplastic-like	0.0020	up
XM_043781227.1	LOC122608136	wound-induced basic protein	< 0.0001	up
XM_043761535.1	LOC122589267	ATP-dependent Clp protease proteolytic subunit 3, chloroplastic	0.0009	up
XM_043777786.1	LOC122604927	transcription factor MYB73-like	0.0028	up
XM_043761816.1	LOC122589517	heme-binding protein 2-like	0.0043	up
XM_043761083.1	LOC122588867	chaperone protein dnaJ 8, chloroplastic	< 0.0001	up
XM_043782620.1	LOC122609666	uncharacterized protein At5g01610-like	0.0005	up
XM_043751438.1	LOC122579295	NADH dehydrogenase [ubiquinone] iron-sulfur protein 5-B-like	0.0002	up
XM_043783000.1	LOC122610029	uncharacterized LOC122610029	0.0014	up
XM_043750013.1	LOC122578125	probable serine protease EDA2	0.0035	up
XM_043755910.1	LOC122583512	membrane magnesium transporter	< 0.0001	up
XR_006323643.1	LOC122598527	uncharacterized LOC122598527	< 0.0001	up
XM_043765919.1	LOC122593499	style cell-cycle inhibitor 1-A	0.0012	up
XM_043778979.1	LOC122606010	uncharacterized LOC122606010	0.0012	up
XM_043779202.1	LOC122606241	L-type lectin-domain containing receptor kinase IX.1-like	0.0004	up
XM_043776889.1	LOC122604008	probable small nuclear ribonucleoprotein F	0.0039	up
XM_043751302.1	LOC122579188	membrane-anchored ubiquitin-fold protein 5	0.0024	up
XM_043750676.1	LOC122578662	amino acid transporter AVT1I-like, transcript variant X1	0.0025	up
XM_043762379.1	LOC122590041	urease accessory protein G	0.0048	up
XM_043761084.1	LOC122588868	outer envelope pore protein 21B, chloroplastic-like	0.0036	up
XM_043759343.1	LOC122587232	autophagy-related protein 3	0.0014	up
XM_043760800.1	LOC122588644	UDP-N-acetylglucosamine transporter ROCK1	< 0.0001	up
XM_043768098.1	LOC122595678	GATA transcription factor 24-like, transcript variant X1	0.0002	up
XM_043761206.1	LOC122588981	magnesium protoporphyrin IX methyltransferase, chloroplastic-like	0.0023	up
XM_043768384.1	LOC122595905	translation machinery-associated protein 22	0.0001	up
XM_043784211.1	LOC122611223	hydroxyproline O-galactosyltransferase HPGT3-like	0.0010	up
XM_043770975.1	LOC122598382	glucan endo-1,3-beta-glucosidase 11	0.0006	up
XM_043778125.1	LOC122605219	protein REVEILLE 2-like	0.0002	up
XM_043765432.1	LOC122593084	flap endonuclease 1, transcript variant X1	0.0054	up
XM_043751981.1	LOC122579752	cytochrome c-type biogenesis ccda-like chloroplastic protein	0.0007	up
XM_043754929.1	LOC122582524	uncharacterized LOC122582524	< 0.0001	up
XM_043772234.1	LOC122599686	glutathione peroxidase 1	< 0.0001	up
XM_043779681.1	LOC122606816	proteasome subunit alpha type-6	0.0009	up
XM_043767660.1	LOC122595317	early nodulin-93-like	0.0046	up
XM_043775700.1	LOC122603086	protein disulfide isomerase-like 1-4	0.0044	up
XM_043756227.1	LOC122583846	syntaxin-71	0.0003	up
XM_043760641.1	LOC122588509	uncharacterized protein At2g38710	0.0022	up
XR_006324170.1	LOC122602172	uncharacterized LOC122602172	0.0019	up
XM_043767377.1	LOC122595080	2-oxoglutarate-dependent dioxygenase 19-like	< 0.0001	up
XM_043766623.1	LOC122594151	proteinaceous RNase P 1, chloroplastic/mitochondrial-like	< 0.0001	up
XM_043772477.1	LOC122599876	protein SUPPRESSOR OF GENE SILENCING 3-like	< 0.0001	up
XM_043751070.1	LOC122578991	early nodulin-93-like	0.0001	up
XM_043768308.1	LOC122595850	F-actin-capping protein subunit alpha	< 0.0001	up
XM_043752598.1	LOC122580322	histone acetyltransferase HAC1, transcript variant X1	0.0024	up
XM_043772261.1	LOC122599710	serine/threonine-protein kinase D6PKL2	< 0.0001	up
XM_043772814.1	LOC122600148	glucose-6-phosphate/phosphate translocator 2, chloroplastic-like	0.0023	up
XR_006322292.1	LOC122589613	uncharacterized LOC122589613	0.0047	up
XM_043782701.1	LOC122609759	serine/threonine-protein kinase SAPK3-like	0.0001	up
XM_043776958.1	LOC122604058	glycine-rich RNA-binding protein 4, mitochondrial	0.0001	up
XM_043778609.1	LOC122605651	probable serine/threonine-protein kinase PBL3	< 0.0001	up
XM_043777108.1	LOC122604212	GDT1-like protein 4	< 0.0001	up
XM_043758081.1	LOC122585966	probable calcium-binding protein CML13	< 0.0001	up
XM_043761410.1	LOC122589161	protein SYS1 homolog	0.0020	up
XM_043750503.1	LOC122578527	alcohol dehydrogenase 1	0.0013	up
XM_043766799.1	LOC122594321	40S ribosomal protein S15a-like	0.0024	up
XM_043776917.1	LOC122604029	metal transporter Nramp3-like	0.0045	up
XM_043756232.1	LOC122583858	protein FRA10AC1	0.0022	up
XM_043754816.1	LOC122582428	uncharacterized LOC122582428, transcript variant X2	0.0010	up
XM_043772137.1	LOC122599605	probable sphingolipid transporter spinster homolog 2	0.0010	up
XM_043751188.1	LOC122579090	protein IQ-DOMAIN 13-like, transcript variant X2	0.0055	up
XM_043766456.1	LOC122593983	vacuolar protein sorting-associated protein 2 homolog 3	0.0017	up
XM_043780332.1	LOC122607367	endoribonuclease YBEY, chloroplastic	0.0040	up
XM_043780602.1	LOC122607594	60S ribosomal protein L14-2-like	0.0017	up
XM_043765059.1	LOC122592761	uncharacterized LOC122592761	0.0018	up
XM_043753161.1	LOC122581017	uncharacterized LOC122581017, transcript variant X1	0.0002	up

Transcript id	GeneID	Gene name/function	P value	result
XM_043778462.1	LOC122605510	uncharacterized LOC122605510, transcript variant X2	0.0005	up
XM_043778523.1	LOC122605564	small RNA-binding protein 11, chloroplastic-like	0.0037	up
XM_043754988.1	LOC122582577	2-alkenal reductase (NADP(+)-dependent)-like	0.0009	up
XM_043779999.1	LOC122607079	60S ribosomal protein L21-1	0.0006	up
XM_043751205.1	LOC122579105	protein OVEREXPRESSOR OF CATIONIC PEROXIDASE 3, transcript variant X2	0.0035	up
XM_043776893.1	LOC122604011	ubiquitin-like protein 5	< 0.0001	up
XM_043764966.1	LOC122592680	peptidyl-prolyl cis-trans isomerase CYP21-4-like, transcript variant X1	0.0026	up
XM_043779286.1	LOC122606331	protein trichome birefringence-like 39	< 0.0001	up
XM_043767734.1	LOC122595382	subtilisin-like protease SBT2.6, transcript variant X1	0.0020	up
XR_006322898.1	LOC122593893	uncharacterized LOC122593893, transcript variant X2	< 0.0001	up
XM_043760754.1	LOC122588605	aspartic proteinase-like protein 1	0.0011	up
XM_043767060.1	LOC122594627	beta-1,3-galactosyltransferase pvg3-like	0.0003	up
XM_043766550.1	LOC122594076	uncharacterized LOC122594076	0.0004	up
XM_043773000.1	LOC122600294	60S ribosomal protein L37a	< 0.0001	up
XM_043780302.1	LOC122607346	serine/threonine-protein kinase 16-like	0.0024	up
XM_043769041.1	LOC122596460	protein trichome birefringence-like 5	0.0015	up
XM_043751595.1	LOC122579422	protein NUCLEAR FUSION DEFECTIVE 6, mitochondrial-like	0.0039	up
XM_043781783.1	LOC122608689	14-3-3-like protein GF14 iota	0.0003	up
XM_043781096.1	LOC122608015	uncharacterized LOC122608015	< 0.0001	up
XM_043750577.1	LOC122578592	uncharacterized LOC122578592	0.0004	up
XM_043776887.1	LOC122604007	40S ribosomal protein S23	< 0.0001	up
XM_043775764.1	LOC122603142	polyadenylate-binding protein-interacting protein 11-like	0.0004	up
XM_043755112.1	LOC122582686	protein FAM136A-like	< 0.0001	up
XM_043778682.1	LOC122605723	uncharacterized LOC122605723	< 0.0001	up
XM_043754826.1	LOC122582437	glycosyltransferase-like KOBITO 1	0.0002	up
XM_043772416.1	LOC122599828	ras-related protein RABB1c-like	0.0003	up
XR_006321353.1	LOC122583937	uncharacterized LOC122583937	0.0001	up
XM_043769208.1	LOC122596597	monothiol glutaredoxin-S15, mitochondrial-like	0.0003	up
XM_043760882.1	LOC122588712	protein mago nashi homolog	0.0001	up
XM_043780136.1	LOC122607207	RWD domain-containing protein 1, transcript variant X1	0.0029	up
XM_043773536.1	LOC122600777	2-C-methyl-D-erythritol 2,4-cyclodiphosphate synthase, chloroplastic	0.0054	up
XR_006323735.1	LOC122598810	uncharacterized LOC122598810	0.0017	up
XM_043762338.1	LOC122590002	amino acid transporter AVT1I-like	0.0056	up
XM_043761113.1	LOC122588893	ACT domain-containing protein ACR6-like	0.0047	up
XM_043778679.1	LOC122605721	50S ribosomal protein HLP, mitochondrial	0.0012	up
XM_043782963.1	LOC122609995	cytokinin riboside 5'-monophosphate phosphoribohydrolase LOG8	< 0.0001	up
XM_043779916.1	LOC122606999	pyruvate decarboxylase 2	0.0001	up
XM_043776239.1	LOC122603514	probable methionine--tRNA ligase, transcript variant X2	0.0002	up
XM_043759550.1	LOC122587395	GPCR-type G protein 1-like	< 0.0001	up
XM_043751262.1	LOC122579150	uncharacterized LOC122579150	< 0.0001	up
XM_043761880.1	LOC122589576	inorganic pyrophosphatase 2-like	< 0.0001	up
XM_043767819.1	LOC122595456	beta-galactosidase 1-like	0.0004	up
XM_043765551.1	LOC122593182	FCS-Like Zinc finger 8-like	< 0.0001	up
XM_043777027.1	LOC122604124	uncharacterized membrane protein At4g09580-like	0.0033	up
XM_043765785.1	LOC122593386	50S ribosomal protein L9-like	0.0014	up
XM_043762522.1	LOC122590190	urease accessory protein D, transcript variant X2	0.0041	up
XM_043769754.1	LOC122597122	uncharacterized LOC122597122, transcript variant X2	0.0001	up
XM_043770654.1	LOC122598047	ubiquitin-40S ribosomal protein S27a-like	0.0023	up
XM_043776652.1	LOC122603829	esterase AGAP003155-like	0.0027	up
XM_043779721.1	LOC122606843	putative uncharacterized protein DDB_G0286901	0.0034	up
XM_043764784.1	LOC122592532	COP9 signalosome complex subunit 7-like, transcript variant X2	0.0012	up
XM_043753163.1	LOC122581022	auxin transporter-like protein 2	0.0048	up
XR_006324727.1	LOC122605831	uncharacterized LOC122605831	0.0028	up
XM_043772135.1	LOC122599603	proteasome subunit beta type-1-like	< 0.0001	up
XM_043769805.1	LOC122597176	uncharacterized LOC122597176	0.0030	up
XM_043772491.1	LOC122599887	chloroplast processing peptidase, transcript variant X2	0.0016	up
XM_043765946.1	LOC122593525	50S ribosomal protein L18	0.0016	up
XM_043782628.1	LOC122609676	sugar transporter ERD6-like 16	< 0.0001	up
XM_043783311.1	LOC122610315	cytochrome b5	0.0037	up
XM_043774956.1	LOC122602244	organic cation/carnitine transporter 4-like	0.0023	up
XM_043768739.1	LOC122596204	uncharacterized LOC122596204	< 0.0001	up
XM_043762629.1	LOC122590306	epsin-3-like	0.0032	up
XM_043750919.1	LOC122578870	isoaspartyl peptidase/L-asparaginase	< 0.0001	up
XM_043767784.1	LOC122595427	glucan endo-1,3-beta-glucosidase 12	0.0058	up
XM_043779081.1	LOC122606116	cyclin-dependent kinase F-4-like	< 0.0001	up
XM_043779719.1	LOC122606839	N-terminal acetyltransferase A complex catalytic subunit NAA10	0.0002	up
XM_043784340.1	LOC122611326	endoplasmic reticulum-Golgi intermediate compartment protein 3	0.0033	up

Transcript id	GeneID	Gene name/function	P value	result
XM_043764982.1	LOC122592694	transmembrane protein 184C-like, transcript variant X1	0.0058	up
XM_043770382.1	LOC122597807	uncharacterized LOC122597807	0.0053	up
XM_043781297.1	LOC122608210	glutaredoxin-C11-like	< 0.0001	up
XM_043771691.1	LOC122599223	WD-40 repeat-containing protein MSI1-like, transcript variant X1	0.0006	up
XM_043772178.1	LOC122599640	60S ribosomal protein L32-1-like	0.0009	up
XM_043768909.1	LOC122596346	tubulin-folding cofactor A, transcript variant X1	< 0.0001	up
XM_043768609.1	LOC122596091	uncharacterized LOC122596091, transcript variant X2	0.0011	up
XM_043780505.1	LOC122607516	FK506-binding protein 2-like	< 0.0001	up
XM_043754914.1	LOC122582510	uncharacterized LOC122582510	0.0022	up
XM_043754165.1	LOC122581856	probable peroxygenase 3	0.0042	up
XM_043754889.1	LOC122582490	small EDRK-rich factor 2-like	< 0.0001	up
XM_043753950.1	LOC122581696	homocysteine S-methyltransferase 2-like	< 0.0001	up
XM_043778848.1	LOC122605891	acyl-CoA C20 Delta5-desaturase-like	< 0.0001	up
XM_043752008.1	LOC122579776	protein kish	0.0004	up
XR_006324696.1	LOC122605739	uncharacterized LOC122605739	< 0.0001	up
XM_043767960.1	LOC122595574	dolichyl-diphosphooligosaccharide--protein glycosyltransferase subunit DAD1-like	< 0.0001	up
XR_006323972.1	LOC122599969	uncharacterized LOC122599969	< 0.0001	up
XM_043765532.1	LOC122593163	NADH dehydrogenase [ubiquinone] 1 alpha subcomplex subunit 1	0.0051	up
XM_043750628.1	LOC122578630	peptidyl-prolyl cis-trans isomerase CYP38, chloroplastic	0.0005	up
XM_043780487.1	LOC122607501	60S ribosomal protein L23	0.0021	up
XM_043768295.1	LOC122595839	probable polygalacturonase At1g80170	0.0037	up
XM_043770401.1	LOC122597827	putative ribosome biogenesis protein slx9-like	0.0016	up
XM_043776121.1	LOC122603424	thiol-disulfide oxidoreductase LTO1	0.0051	up
XM_043768475.1	LOC122595983	protein Iojap-related, mitochondrial	0.0037	up
XM_043765289.1	LOC122592954	40S ribosomal protein S24-1	< 0.0001	up
XM_043753307.1	LOC122581143	2-isopropylmalate synthase A-like	0.0056	up
XM_043781107.1	LOC122608025	MICOS complex subunit Mic10-like	< 0.0001	up
XM_043784316.1	LOC122611305	calmodulin	0.0016	up
XM_043770440.1	LOC122597859	30S ribosomal protein S31, mitochondrial	< 0.0001	up
XM_043780212.1	LOC122607276	co-chaperone protein p23-1-like	0.0032	up
XM_043761583.1	LOC122589308	serine-threonine kinase receptor-associated protein-like	0.0002	up
XM_043773073.1	LOC122600365	enhanced ethylene response protein 5	0.0039	up
XM_043752083.1	LOC122579836	sugar transporter ERD6-like 5, transcript variant X1	0.0006	up
XM_043772434.1	LOC122599846	ferric reduction oxidase 7, chloroplastic-like, transcript variant X1	< 0.0001	up
XM_043781405.1	LOC122608312	DNA-directed RNA polymerase III subunit RPC8	0.0002	up
XM_043781788.1	LOC122608696	dnaJ homolog subfamily B member 13-like	< 0.0001	up
XM_043769251.1	LOC122596638	RNA exonuclease 4	0.0005	up
XM_043767208.1	LOC122594924	putative transferase At4g12130, mitochondrial, transcript variant X4	< 0.0001	up
XM_043778918.1	LOC122605950	kinesin-like protein KIN-14J, transcript variant X2	0.0001	up
XM_043769974.1	LOC122597375	uncharacterized LOC122597375	0.0010	up
XM_043777031.1	LOC122604132	thaumatin-like protein 1	0.0008	up
XM_043779884.1	LOC122606974	eukaryotic translation initiation factor 2 subunit beta	0.0050	up
XM_043751311.1	LOC122579198	lichenase-like	< 0.0001	up
XR_006325495.1	LOC122611253	protein GDAP2 homolog, transcript variant X4	< 0.0001	up
XM_043778854.1	LOC122605898	glycerophosphodiester phosphodiesterase GDPD1, chloroplastic	< 0.0001	up
XM_043784387.1	LOC122611364	ubiquitin-conjugating enzyme E2-23 kDa-like	0.0008	up
XM_043776759.1	LOC122603906	ethylene-responsive transcription factor ERF104-like	0.0009	up
XM_043775041.1	LOC122602344	casein kinase II subunit beta-1-like	0.0005	up
XM_043776565.1	LOC122603766	uncharacterized LOC122603766	0.0001	up
XM_043762958.1	LOC122590785	cytochrome b5	0.0029	up
XM_043766048.1	LOC122593623	uncharacterized LOC122593623	< 0.0001	up
XM_043762023.1	LOC122589702	HIPL1 protein-like	0.0013	up
XM_043763298.1	LOC122591095	26S proteasome regulatory subunit 6A homolog	0.0058	up
XM_043765339.1	LOC122593005	protein RER1A-like	0.0006	up
XM_043752490.1	LOC122580214	malate synthase, glyoxysomal	0.0002	up
XM_043751852.1	LOC122579641	electron transfer flavoprotein subunit beta, mitochondrial, transcript variant X1	0.0024	up
XM_043751746.1	LOC122579557	serine carboxypeptidase-like 42	< 0.0001	up
XR_006322826.1	LOC122593352	uncharacterized LOC122593352	< 0.0001	up
XM_043780623.1	LOC122607609	uncharacterized LOC122607609	0.0008	up
XM_043774962.1	LOC122602255	uncharacterized LOC122602255	0.0006	up
XM_043753714.1	LOC122581483	60S ribosomal protein L19-1-like	< 0.0001	up
XM_043773667.1	LOC122600889	metal tolerance protein 1-like	0.0034	up
XM_043775511.1	LOC122602899	organelle RRM domain-containing protein 2, mitochondrial	0.0004	up
XM_043781084.1	LOC122608004	uncharacterized LOC122608004	0.0052	up
XM_043777930.1	LOC122605056	non-symbiotic hemoglobin 2-like	< 0.0001	up
XM_043756277.1	LOC122583905	50S ribosomal protein L18-like	0.0021	up

Transcript id	GeneID	Gene name/function	P value	result
XM_043759577.1	LOC122587415	ribosomal protein S7, mitochondrial	< 0.0001	up
XM_043773429.1	LOC122600675	60S ribosomal protein L39-1-like	0.0019	up
XM_043760856.1	LOC122588687	uncharacterized LOC122588687	0.0035	up
XM_043759493.1	LOC122587354	probable enoyl-CoA hydratase 1, peroxisomal, transcript variant X1	0.0003	up
XM_043750830.1	LOC122578792	protein KRTCAP2 homolog	< 0.0001	up
XM_043758476.1	LOC122586493	protein SUPPRESSOR OF QUENCHING 1, chloroplastic	< 0.0001	up
XM_043769340.1	LOC122596716	50S ribosomal protein L18-like	< 0.0001	up
XM_043779606.1	LOC122606747	60S ribosomal protein L32-1-like	< 0.0001	up
XM_043770289.1	LOC122597724	DNA-directed RNA polymerases II and IV subunit 5A-like	0.0011	up
XM_043778375.1	LOC122605424	RING-H2 finger protein ATL8-like	0.0004	up
XM_043763193.1	LOC122591008	polyadenylate-binding protein RBP47-like, transcript variant X3	0.0031	up
XM_043778301.1	LOC122605369	uncharacterized LOC122605369	0.0001	up
XM_043779157.1	LOC122606197	NADH dehydrogenase [ubiquinone] 1 alpha subcomplex subunit 13-A	0.0056	up
XM_043765735.1	LOC122593341	uncharacterized LOC122593341	0.0002	up
XM_043780944.1	LOC122607874	uncharacterized LOC122607874	< 0.0001	up
XM_043773732.1	LOC122600955	uncharacterized LOC122600955	0.0002	up
XM_043781624.1	LOC122608519	uncharacterized LOC122608519	< 0.0001	up
XM_043750536.1	LOC122578556	PLASMODESMATA CALLOSE-BINDING PROTEIN 4-like	0.0002	up
XM_043765755.1	LOC122593361	uncharacterized LOC122593361	0.0008	up
XM_043771853.1	LOC122599349	UDP-galactose/UDP-glucose transporter 7	0.0010	up
XM_043753359.1	LOC122581189	putative GTP diphosphokinase RSH1, chloroplastic, transcript variant	< 0.0001	up
XM_043777934.1	LOC122605060	protein PLASTID REDOX INSENSITIVE 2, chloroplastic-like	< 0.0001	up
XM_043752924.1	LOC122580653	N-alpha-acetyltransferase 50	0.0002	up
XM_043777909.1	LOC122605039	chaperone protein dnaJ 11, chloroplastic-like	0.0006	up
XM_043770393.1	LOC122597817	prohibitin-1, mitochondrial-like	< 0.0001	up
XM_043772852.1	LOC122600182	phosphopantothenoylcysteine decarboxylase subunit VHS3-like	< 0.0001	up
XM_043762159.1	LOC122589839	guanosine deaminase-like	< 0.0001	up
XM_043770266.1	LOC122597696	palmitoyl-monogalactosyldiacylglycerol delta-7 desaturase, chloroplastic-like	0.0037	up
XM_043771283.1	LOC122598760	calcium-binding protein 39	< 0.0001	up
XM_043755213.1	LOC122582778	oleoyl-acyl carrier protein thioesterase, chloroplastic-like	0.0003	up
XM_043755234.1	LOC122582797	gamma-interferon-responsive lysosomal thiol protein-like	< 0.0001	up
XM_043749943.1	LOC122578075	ras-related protein RABH1b-like	0.0055	up
XM_043767853.1	LOC122595487	tropinone reductase homolog At5g06060-like	0.0033	up
XM_043751714.1	LOC122579528	eyes absent homolog	< 0.0001	up
XM_043759618.1	LOC122587153	GMP synthase [glutamine-hydrolyzing]-like, transcript variant X1	0.0002	up
XM_043773164.1	LOC122600452	U-box domain-containing protein 12-like	0.0005	up
XM_043781996.1	LOC122608926	14-3-3 protein 10	0.0002	up
XM_043751085.1	LOC122579006	peptidyl-prolyl cis-trans isomerase CYP21-1-like	0.0002	up
XM_043762982.1	LOC122590813	60S ribosomal protein L11	0.0007	up
XM_043751536.1	LOC122579370	50S ribosomal protein L20	0.0002	up
XM_043771141.1	LOC122598548	(3aS,4S,5R,7aS)-5-hydroxy-7a-methyl-1-oxo-octahydro-1H-indene-4-carboxyl-CoA dehydrogenase	0.0005	up
XM_043778079.1	LOC122605182	40S ribosomal protein S6-like	0.0018	up
XM_043761778.1	LOC122589484	endoglucanase 2-like	< 0.0001	up
XM_043771521.1	LOC122599070	inactive beta-amylase 9	0.0005	up
XM_043754707.1	LOC122582338	cytochrome b-c1 complex subunit 8-2, mitochondrial-like	< 0.0001	up
XM_043759427.1	LOC122587301	40S ribosomal protein S19-3	0.0055	up
XM_043775616.1	LOC122603003	uncharacterized LOC122603003	0.0002	up
XR_006321603.1	LOC122584830	uncharacterized LOC122584830	0.0015	up
XR_006320558.1	LOC122578878	uncharacterized LOC122578878	0.0009	up
XM_043751948.1	LOC122579724	LMBR1 domain-containing protein 2 homolog	0.0003	up
XM_043756753.1	LOC122584706	uncharacterized LOC122584706	0.0010	up
XM_043759467.1	LOC122587333	peptidyl-prolyl cis-trans isomerase FKBP53	0.0003	up
XM_043763980.1	LOC122591733	pelargonidin 3-O-(6-caffeoylglucoside) 5-O-(6-O-malonylglucoside) 4'''-malonyltransferase-like	0.0056	up
XR_006321121.1	LOC122582147	uncharacterized LOC122582147	< 0.0001	up
XM_043750142.1	LOC122578232	protein GrpE-like	0.0004	up
XM_043759248.1	LOC122587158	probable galacturonosyltransferase 9	0.0013	up
XM_043779532.1	LOC122606686	beta-glucosidase 13-like	< 0.0001	up
XM_043769247.1	LOC122596636	heat shock cognate 70 kDa protein	0.0007	up
XM_043781304.1	LOC122608217	mitochondrial uncoupling protein 2-like	< 0.0001	up
XM_043755153.1	LOC122582731	60S ribosomal protein L23	0.0017	up
XM_043765672.1	LOC122593282	40S ribosomal protein S15a-5-like	0.0020	up
XM_043771117.1	LOC122598524	transmembrane emp24 domain-containing protein p24delta9-like	0.0011	up
XM_043757724.1	LOC122585602	60S acidic ribosomal protein P2-like	0.0010	up
XM_043763034.1	LOC122590857	codeine O-demethylase-like	0.0001	up
XM_043774646.1	LOC122601914	upstream activation factor subunit UAF30	0.0027	up

Transcript id	GeneID	Gene name/function	P value	result
XM_043772627.1	LOC122599999	myosin-4-like	0.0012	up
XM_043775500.1	LOC122602890	uncharacterized LOC122602890, transcript variant X1	< 0.0001	up
XM_043752509.1	LOC122580231	flowering-promoting factor 1-like protein 3	0.0002	up
XM_043758996.1	LOC122586954	eukaryotic translation initiation factor 3 subunit E	0.0050	up
XM_043764836.1	LOC122592577	uncharacterized LOC122592577, transcript variant X3	0.0006	up
XM_043767700.1	LOC122595350	protein DETOXIFICATION 29-like	< 0.0001	up
XM_043754581.1	LOC122582220	serine/threonine-protein kinase 19, transcript variant X3	< 0.0001	up
XM_043757068.1	LOC122584975	putative TrmH family tRNA/rRNA methyltransferase	< 0.0001	up
XM_043767788.1	LOC122595431	ER lumen protein-retaining receptor erd-2.2	< 0.0001	up
XM_043772476.1	LOC122599875	pre-mRNA cleavage factor Im 25 kDa subunit 2	0.0001	up
XM_043753937.1	LOC122581686	proteasome subunit alpha type-3	< 0.0001	up
XM_043767314.1	LOC122595033	protein Iojap, chloroplastic	0.0002	up
XM_043778058.1	LOC122605165	glutaredoxin-C3	0.0055	up
XM_043769277.1	LOC122596659	60S ribosomal protein L7-2	< 0.0001	up
XM_043780961.1	LOC122607887	zinc transporter ZTP29	0.0053	up
XM_043770348.1	LOC122597780	cysteine-rich PDZ-binding protein	< 0.0001	up
XM_043756701.1	LOC122584657	uncharacterized LOC122584657	0.0016	up
XM_043777973.1	LOC122605097	ER lumen protein-retaining receptor	< 0.0001	up
XM_043774856.1	LOC122602130	1-aminocyclopropane-1-carboxylate oxidase 1	< 0.0001	up
XM_043778795.1	LOC122605839	exonuclease V, chloroplastic	0.0012	up
XM_043754852.1	LOC122582462	ras-related protein RABA5a, transcript variant X1	0.0024	up
XM_043755162.1	LOC122582739	uncharacterized LOC122582739	0.0001	up
XM_043753823.1	LOC122581586	sorting nexin 1, transcript variant X2	0.0022	up
XM_043781830.1	LOC122608735	LOB domain-containing protein 14-like	0.0003	up
XM_043751071.1	LOC122578994	V-type proton ATPase subunit e1, transcript variant X2	0.0056	up
XM_043776959.1	LOC122604059	reactive oxygen species modulator 1	< 0.0001	up
XM_043764531.1	LOC122592330	putative vacuolar protein sorting-associated protein 13D	< 0.0001	up
XM_043781845.1	LOC122608754	RNA-directed DNA methylation 4, transcript variant X1	0.0004	up
XM_043781048.1	LOC122607964	protein Asterix	< 0.0001	up
XM_043780651.1	LOC122607635	mitochondrial import inner membrane translocase subunit TIM10-like	0.0018	up
XM_043779376.1	LOC122606517	two-component response regulator-like APRR1	0.0019	up
XM_043770726.1	LOC122598127	protein translation factor SUI1 homolog	0.0005	up
XM_043776363.1	LOC122603611	uncharacterized protein At3g27210-like	0.0028	up
XM_043765507.1	LOC122593144	uncharacterized LOC122593144	< 0.0001	up
XM_043755012.1	LOC122582600	DNA gyrase subunit B, chloroplastic/mitochondrial-like	0.0003	up
XM_043768913.1	LOC122596350	protein transport protein SFT2, transcript variant X1	0.0016	up
XM_043756855.1	LOC122584794	mitochondrial import inner membrane translocase subunit PAM16 like 2-like	0.0003	up
XM_043766638.1	LOC122594167	B-box zinc finger protein 20-like	0.0019	up
XM_043773610.1	LOC122600832	eukaryotic translation initiation factor 5A	< 0.0001	up
XM_043772869.1	LOC122600194	poly(rC)-binding protein 4-like	< 0.0001	up
XM_043767851.1	LOC122595485	protein MANNAN SYNTHESIS-RELATED 2	0.0008	up
XM_043755128.1	LOC122582702	calcium-dependent protein kinase SK5-like	0.0002	up
XM_043780493.1	LOC122607506	mitochondrial import receptor subunit TOM40-1-like	0.0041	up
XM_043751663.1	LOC122579481	protein S-acyltransferase 8-like	0.0007	up
XM_043775072.1	LOC122602380	glucan endo-1,3-beta-glucosidase 5	0.0010	up
XM_043752440.1	LOC122580169	peptidyl-prolyl cis-trans isomerase Pin1	0.0001	up
XM_043780362.1	LOC122607396	zinc finger protein CONSTANS-LIKE 13	0.0032	up
XM_043768536.1	LOC122596036	ATP synthase subunit epsilon, mitochondrial	0.0004	up
XM_043778179.1	LOC122605270	uncharacterized LOC122605270	0.0047	up
XM_043752942.1	LOC122580675	heat shock 70 kDa protein II-like, transcript variant X1	< 0.0001	up
XM_043751867.1	LOC122579654	uncharacterized LOC122579654, transcript variant X1	0.0038	up
XM_043759607.1	LOC122587439	aluminum-activated malate transporter 4-like, transcript variant X2	0.0017	up
XM_043770718.1	LOC122598117	glutathione hydrolase 3-like	0.0040	up
XM_043768817.1	LOC122596265	50S ribosomal protein L7/L12	0.0004	up
XR_006322163.1	LOC122588461	uncharacterized LOC122588461	0.0034	up
XM_043775387.1	LOC122602790	calcium load-activated calcium channel-like	0.0053	up
XM_043779179.1	LOC122606219	coiled-coil domain-containing protein 12-like, transcript variant X4	0.0006	up
XM_043776726.1	LOC122603884	SWI/SNF complex component SNF12 homolog	0.0024	up
XM_043752238.1	LOC122579972	mitochondrial import receptor subunit TOM20-like	< 0.0001	up
XR_006320841.1	LOC122580693	U5 spliceosomal RNA	< 0.0001	up
XM_043764511.1	LOC122592308	G-type lectin S-receptor-like serine/threonine-protein kinase At4g27290	< 0.0001	up
XM_043779520.1	LOC122606675	glucan endo-1,3-beta-glucosidase 4	< 0.0001	up
XM_043770909.1	LOC122598316	60S ribosomal protein L11	0.0002	up
XM_043755225.1	LOC122582787	electron transfer flavoprotein-ubiquinone oxidoreductase, mitochondrial	< 0.0001	up
XM_043765304.1	LOC122592971	U1 small nuclear ribonucleoprotein A	0.0003	up
XM_043760502.1	LOC122588398	mitochondrial import inner membrane translocase subunit TIM8	0.0040	up
XM_043754211.1	LOC122581894	BTB/POZ domain-containing protein At4g08455-like	0.0010	up

Transcript id	GeneID	Gene name/function	*P* value	result
XM_043760614.1	LOC122588486	histidine biosynthesis bifunctional protein hisIE, chloroplastic, transcript variant X2	0.0054	up
XM_043750041.1	LOC122578146	uncharacterized LOC122578146	0.0002	up
XM_043771780.1	LOC122599290	cyclic nucleotide-gated ion channel 2	0.0048	up
XM_043779828.1	LOC122606933	40S ribosomal protein S26-1-like	0.0016	up
XM_043749991.1	LOC122578109	40S ribosomal protein S3a	0.0012	up
XM_043765521.1	LOC122593156	protein starmaker	0.0022	up
XM_043751417.1	LOC122579282	ATP-dependent Clp protease proteolytic subunit 2, mitochondrial	0.0009	up
XM_043761322.1	LOC122589078	uncharacterized LOC122589078	0.0017	up
XM_043765040.1	LOC122592746	haloacid dehalogenase-like hydrolase domain-containing protein 3, transcript variant X1	0.0004	up
XM_043765183.1	LOC122592863	uncharacterized LOC122592863	0.0058	up
XM_043781558.1	LOC122608449	40S ribosomal protein S24-1-like	< 0.0001	up
XR_006323461.1	LOC122597803	PI-PLC X domain-containing protein At5g67130-like, transcript variant X2	< 0.0001	up
XR_006324066.1	LOC122600580	MDIS1-interacting receptor like kinase 2-like, transcript variant X4	0.0028	up
XM_043768512.1	LOC122596016	probable protein disulfide-isomerase A6	0.0046	up
XM_043776530.1	LOC122603742	30S ribosomal protein S21, chloroplastic	0.0032	up
XM_043779300.1	LOC122606381	protein ENHANCED DISEASE RESISTANCE 2-like, transcript variant X2	< 0.0001	up
XM_043770376.1	LOC122597801	serine--tRNA ligase	0.0011	up
XM_043765617.1	LOC122593235	cytochrome B5-like protein	< 0.0001	up
XM_043779420.1	LOC122606579	calmodulin-7	< 0.0001	up
XM_043773019.1	LOC122600311	NHP2-like protein 1, transcript variant X2	< 0.0001	up
XM_043757194.1	LOC122585099	probable pectinesterase 68	0.0034	up
XM_043757241.1	LOC122585136	uncharacterized LOC122585136	0.0011	up
XM_043764462.1	LOC122592273	stigma-specific STIG1-like protein 1	< 0.0001	up
XR_006321821.1	LOC122585922	uncharacterized LOC122585922	< 0.0001	up
XM_043777200.1	LOC122604311	dolichyl-diphosphooligosaccharide--protein glycosyltransferase subunit 4A-like	0.0054	up
XM_043780526.1	LOC122607536	uncharacterized LOC122607536, transcript variant X2	< 0.0001	up
XR_006322473.1	LOC122590333	U5 spliceosomal RNA	0.0032	up
XM_043750475.1	LOC122578502	phosphatidylinositol 4-phosphate 5-kinase 2-like	0.0002	up
XM_043769135.1	LOC122596537	transmembrane protein 208 homolog	< 0.0001	up
XM_043751640.1	LOC122579457	CRM-domain containing factor CFM9, mitochondrial	0.0025	up
XM_043750754.1	LOC122578729	WUSCHEL-related homeobox 8-like, transcript variant X2	0.0020	up
XM_043768872.1	LOC122596320	ATP-citrate synthase alpha chain protein 2	0.0025	up
XM_043770037.1	LOC122597444	metacaspase-3-like	< 0.0001	up
XM_043756814.1	LOC122584759	mitoferrin, transcript variant X2	0.0010	up
XM_043753827.1	LOC122581589	DNA-binding protein DDB_G0278111, transcript variant X3	< 0.0001	up
XM_043762703.1	LOC122590502	protein STRICTOSIDINE SYNTHASE-LIKE 4-like	0.0003	up
XM_043754651.1	LOC122582281	uncharacterized LOC122582281	0.0055	up
XM_043782669.1	LOC122609720	uncharacterized LOC122609720	0.0023	up
XM_043768944.1	LOC122596379	mitochondrial import receptor subunit TOM20-like	< 0.0001	up
XM_043779815.1	LOC122606922	COP9 signalosome complex subunit 4	< 0.0001	up
XM_043766244.1	LOC122593794	heparanase-like protein 3	0.0037	up
XM_043779948.1	LOC122607028	NAP1-related protein 1-like, transcript variant X2	0.0003	up
XM_043751704.1	LOC122579518	50S ribosomal protein L27-like	0.0001	up
XM_043768530.1	LOC122596030	beta-glucuronosyltransferase GlcAT14A	0.0055	up
XM_043772683.1	LOC122600039	protein OS-9 homolog	< 0.0001	up
XM_043754557.1	LOC122582195	small nuclear ribonucleoprotein SmD3b	0.0002	up
XM_043758392.1	LOC122586400	uncharacterized protein At1g01500-like, transcript variant X3	0.0006	up
XM_043753019.1	LOC122580871	50S ribosomal protein L15	< 0.0001	up
XM_043755658.1	LOC122583233	uncharacterized LOC122583233	0.0011	up
XR_006323327.1	LOC122596476	uncharacterized LOC122596476	0.0015	up
XR_006325509.1	LOC122611377	uncharacterized LOC122611377, transcript variant X3	0.0023	up
XM_043781442.1	LOC122608347	probable E3 ubiquitin-protein ligase BAH1-like 1	0.0032	up
XM_043772803.1	LOC122600139	V-type proton ATPase subunit F, transcript variant X2	0.0046	up
XM_043783585.1	LOC122610610	6,7-dimethyl-8-ribityllumazine synthase, chloroplastic	0.0029	up
XM_043764683.1	LOC122592450	uncharacterized protein YnbB-like, transcript variant X2	< 0.0001	up
XM_043757049.1	LOC122584957	probable signal peptidase complex subunit 1, transcript variant X1	0.0037	up
XM_043750881.1	LOC122578833	expansin-like B1	< 0.0001	up
XM_043766110.1	LOC122593671	uncharacterized LOC122593671, transcript variant X2	0.0047	up
XM_043774424.1	LOC122601683	uncharacterized LOC122601683	0.0006	up
XM_043779348.1	LOC122606491	ATP-dependent zinc metalloprotease FTSH 3, mitochondrial-like, transcript variant X1	0.0004	up
XM_043773354.1	LOC122600608	peptidyl-prolyl cis-trans isomerase CYP18-1	0.0025	up
XM_043776778.1	LOC122603922	protein transport protein Sec61 subunit gamma-like	< 0.0001	up
XM_043771309.1	LOC122598822	uncharacterized LOC122598822	< 0.0001	up

Transcript id	GeneID	Gene name/function	*P* value	result
XM_043764659.1	LOC122592435	glutathione S-transferase U19-like	0.0001	up
XM_043765842.1	LOC122593431	mediator of RNA polymerase II transcription subunit 28	0.0008	up
XM_043751335.1	LOC122579216	peptidyl-prolyl cis-trans isomerase CYP65	0.0013	up
XR_006320691.1	LOC122579747	signal peptide peptidase-like 2, transcript variant X2	0.0007	up
XM_043752163.1	LOC122579905	ubiquitin-conjugating enzyme E2 22-like, transcript variant X1	< 0.0001	up
XM_043750825.1	LOC122578787	cytochrome c oxidase subunit 5C-1	0.0034	up
XM_043767392.1	LOC122595089	methionine aminopeptidase 1B, chloroplastic	< 0.0001	up
XM_043758912.1	LOC122586893	protein SUPPRESSOR OF FRI 4, transcript variant X2	0.0026	up
XM_043754533.1	LOC122582176	uncharacterized LOC122582176, transcript variant X1	< 0.0001	up
XM_043761529.1	LOC122589261	ADP-ribosylation factor-like protein 2, transcript variant X2	< 0.0001	up
XM_043768727.1	LOC122596194	heparanase-like protein 3	< 0.0001	up
XM_043772559.1	LOC122599942	gamma-tubulin complex component 5-like, transcript variant X1	0.0007	up
XM_043775297.1	LOC122602705	cytochrome P450 CYP72A219-like	0.0018	up
XM_043761156.1	LOC122588936	gibberellin-regulated protein 14	< 0.0001	up
XM_043782648.1	LOC122609700	uncharacterized LOC122609700	< 0.0001	up
XM_043772164.1	LOC122599626	protein IQ-DOMAIN 5-like, transcript variant X1	< 0.0001	up
XM_043756940.1	LOC122584866	organelle RRM domain-containing protein 1, chloroplastic	0.0008	up
XM_043751169.1	LOC122579077	single-stranded DNA-binding protein, mitochondrial	0.0036	up
XM_043754454.1	LOC122582096	uncharacterized LOC122582096, transcript variant X2	0.0031	up
XM_043753079.1	LOC122580933	AP-2 complex subunit sigma-like	< 0.0001	up
XM_043769250.1	LOC122596637	universal stress protein YxiE, transcript variant X2	0.0032	up
XM_043779589.1	LOC122606731	amino acid transporter AVT1A-like	< 0.0001	up
XM_043768827.1	LOC122596276	50S ribosomal protein L3, chloroplastic	0.0007	up
XM_043776110.1	LOC122603417	ethylene-responsive transcription factor 5-like	< 0.0001	up
XM_043769317.1	LOC122596697	spastin	0.0004	up
XM_043783697.1	LOC122610734	putative BPI/LBP family protein At1g04970	0.0019	up
XM_043760538.1	LOC122588428	60S ribosomal protein L24-like	< 0.0001	up
XM_043773124.1	LOC122600412	probable phospholipid hydroperoxide glutathione peroxidase	0.0051	up
XM_043780621.1	LOC122607607	uncharacterized LOC122607607	0.0008	up
XM_043763498.1	LOC122591262	putative methyltransferase C9orf114 homolog	0.0017	up
XM_043775818.1	LOC122603178	putative protein TPRXL	0.0001	up
XM_043766028.1	LOC122593598	sm-like protein LSM3B	0.0004	up
XM_043769793.1	LOC122597163	uncharacterized LOC122597163	< 0.0001	up
XM_043759023.1	LOC122586973	uncharacterized protein At2g23090	0.0006	up
XM_043766325.1	LOC122593871	suppressor protein SRP40-like	< 0.0001	up
XM_043756641.1	LOC122584497	myb-like protein P	0.0039	up
XM_043763123.1	LOC122590949	F-box protein PP2-A13-like	< 0.0001	up
XM_043756428.1	LOC122584195	uncharacterized LOC122584195, transcript variant X1	< 0.0001	up
XM_043763735.1	LOC122591467	EG45-like domain containing protein	< 0.0001	up
XM_043751239.1	LOC122579132	WAT1-related protein At3g28050-like	0.0014	up
XM_043766246.1	LOC122593795	CASP-like protein 5B2, transcript variant X1	< 0.0001	up
XR_006323192.1	LOC122595117	phospholipid:diacylglycerol acyltransferase 1-like, transcript variant X4	0.0004	up
XM_043752604.1	LOC122580332	60S ribosomal protein L34	0.0025	up
XR_006322975.1	LOC122594361	uncharacterized LOC122594361	0.0011	up
XM_043757102.1	LOC122585008	peroxisome biogenesis protein 22, transcript variant X1	< 0.0001	up
XM_043761958.1	LOC122589641	auxin response factor 5-like	0.0002	up
XM_043754612.1	LOC122582245	EKC/KEOPS complex subunit TP53RK	< 0.0001	up
XM_043760713.1	LOC122588576	SAGA-associated factor 29 homolog A, transcript variant X1	0.0013	up
XM_043768189.1	LOC122595756	5'-deoxynucleotidase HDDC2-like	0.0050	up
XM_043776421.1	LOC122603654	60S ribosomal protein L27-like	< 0.0001	up
XM_043753901.1	LOC122581656	cyanate hydratase	0.0011	up
XM_043751116.1	LOC122579032	uncharacterized LOC122579032	0.0012	up
XM_043768782.1	LOC122596235	potassium transporter 7-like	< 0.0001	up
XM_043768819.1	LOC122596268	non-specific lipid transfer protein GPI-anchored 1	0.0004	up
XM_043780034.1	LOC122607118	phosphatidylinositol-3-phosphatase SAC1, transcript variant X1	0.0025	up
XM_043765675.1	LOC122593284	uncharacterized LOC122593284	< 0.0001	up
XM_043755308.1	LOC122582877	uncharacterized LOC122582877	0.0001	up
XM_043774822.1	LOC122602086	peptidyl-prolyl cis-trans isomerase CYP20-1-like	< 0.0001	up
XM_043773130.1	LOC122600419	60S ribosomal protein L38, transcript variant X2	0.0019	up
XM_043768198.1	LOC122595762	50S ribosomal protein L18, chloroplastic	< 0.0001	up
XM_043770736.1	LOC122598134	ADP-ribosylation factor 2, transcript variant X1	0.0058	up
XM_043776617.1	LOC122603804	5'-adenylylsulfate reductase-like 5	0.0015	up
XM_043776774.1	LOC122603919	dolichyl-phosphate beta-glucosyltransferase	0.0033	up
XM_043779639.1	LOC122606785	probable acyl-activating enzyme 17, peroxisomal	0.0002	up
XM_043752130.1	LOC122579868	probable methyltransferase PMT5	0.0004	up
XM_043768110.1	LOC122595687	biotin carboxyl carrier protein of acetyl-CoA carboxylase-like	0.0003	up
XM_043762465.1	LOC122590129	stemmadenine O-acetyltransferase-like	< 0.0001	up
XM_043761373.1	LOC122589130	uncharacterized LOC122589130	0.0041	up

Transcript id	GeneID	Gene name/function	*P* value	result
XM_043768206.1	LOC122595769	proteasome subunit beta type-1	< 0.0001	up
XM_043751660.1	LOC122579475	uncharacterized LOC122579475, transcript variant X2	0.0004	up
XM_043750105.1	LOC122578198	thiosulfate sulfurtransferase 16, chloroplastic-like	< 0.0001	up
XM_043764643.1	LOC122592425	proteasome subunit alpha type-5	0.0022	up
XM_043751104.1	LOC122579024	60S ribosomal protein L9-like	0.0002	up
XM_043765331.1	LOC122592999	protein trichome birefringence-like 35, transcript variant X1	< 0.0001	up
XM_043773383.1	LOC122600636	transcription factor MYB14-like	< 0.0001	up
XM_043768296.1	LOC122595840	putative uncharacterized protein DDB_G0270496, transcript variant X3	0.0003	up
XM_043776080.1	LOC122603393	mitochondrial dicarboxylate/tricarboxylate transporter DTC	0.0012	up
XM_043752624.1	LOC122580350	chitinase CLP-like	0.0012	up
XM_043778500.1	LOC122605544	DNA polymerase epsilon subunit 3-like	< 0.0001	up
XM_043775880.1	LOC122603228	fe-S cluster assembly factor HCF101, chloroplastic, transcript variant	0.0036	up
XM_043775672.1	LOC122603058	F-box protein At2g32560-like	0.0002	up
XM_043751500.1	LOC122579342	40S ribosomal protein S28-2-like	< 0.0001	up
XM_043766566.1	LOC122594089	uncharacterized LOC122594089	0.0015	up
XM_043773280.1	LOC122600548	MOB kinase activator-like 1A	0.0023	up
XM_043781839.1	LOC122608746	acid phosphatase 1	0.0001	up
XM_043769320.1	LOC122596699	phospholipase D zeta 1-like	0.0003	up
XM_043771234.1	LOC122598647	uncharacterized LOC122598647	0.0002	up
XM_043760737.1	LOC122588594	exosome complex component CSL4	< 0.0001	up
XM_043776769.1	LOC122603916	uncharacterized LOC122603916	0.0055	up
XM_043755182.1	LOC122582754	serine carboxypeptidase-like 45	< 0.0001	up
XM_043772341.1	LOC122599773	purple acid phosphatase 2-like	0.0042	up
XM_043766387.1	LOC122593926	ribose-phosphate pyrophosphokinase 1-like	< 0.0001	up
XM_043754665.1	LOC122582294	heat shock factor-binding protein-like	0.0001	up
XM_043754671.1	LOC122582301	peptide chain release factor APG3, chloroplastic	0.0002	up
XM_043768851.1	LOC122596302	sm-like protein LSM8	0.0008	up
XM_043761015.1	LOC122588809	mannose-1-phosphate guanylyltransferase 1-like	< 0.0001	up
XM_043781826.1	LOC122608732	probable carbohydrate esterase At4g34215	0.0003	up
XR_006325361.1	LOC122609611	U1 spliceosomal RNA	0.0054	up
XM_043778236.1	LOC122605316	coiled-coil domain-containing protein 12-like, transcript variant X1	< 0.0001	up
XM_043760784.1	LOC122588629	protein BOLA4, chloroplastic/mitochondrial	< 0.0001	up
XM_043758000.1	LOC122585885	transcription factor MYB62-like	< 0.0001	up
XM_043766930.1	LOC122594482	RNA pseudouridine synthase 1, transcript variant X1	0.0006	up
XM_043753557.1	LOC122581340	glyoxylate/succinic semialdehyde reductase 1	0.0003	up
XM_043780537.1	LOC122607544	serine/threonine-protein kinase BSK5-like, transcript variant X1	0.0024	up
XM_043765191.1	LOC122592872	plasmodesmata-located protein 2-like	< 0.0001	up
XM_043773145.1	LOC122600433	mitochondrial import receptor subunit TOM6 homolog, transcript variant X2	0.0015	up
XM_043784051.1	LOC122611090	14-3-3 protein 2	< 0.0001	up
XM_043753913.1	LOC122581664	uncharacterized protein YnbD-like	0.0002	up
XM_043782919.1	LOC122609958	calcineurin B-like protein 2, transcript variant X2	0.0054	up
XM_043763070.1	LOC122590890	topless-related protein 3-like, transcript variant X1	< 0.0001	up
XM_043757694.1	LOC122585563	uncharacterized LOC122585563, transcript variant X4	0.0034	up
XR_006323694.1	LOC122598690	uncharacterized LOC122598690	0.0030	up
XM_043767568.1	LOC122595231	purple acid phosphatase 2-like	0.0014	up
XM_043754735.1	LOC122582357	Golgi SNAP receptor complex member 1-1	0.0055	up
XM_043754385.1	LOC122582040	putative GPI-anchor transamidase	0.0016	up
XM_043760553.1	LOC122588438	zinc finger CCCH domain-containing protein 11-like	0.0003	up
XR_006322773.1	LOC122592883	uncharacterized LOC122592883	0.0039	up
XM_043776362.1	LOC122603610	uridine nucleosidase 1	0.0023	up
XM_043765009.1	LOC122592719	non-specific lipid transfer protein GPI-anchored 31-like	0.0026	up
XM_043760991.1	LOC122588791	dolichyl-diphosphooligosaccharide--protein glycosyltransferase 48 kDa subunit	0.0012	up
XM_043761045.1	LOC122588834	DNA-(apurinic or apyrimidinic site) endonuclease	0.0032	up
XM_043756731.1	LOC122584684	40S ribosomal protein S10-1-like	0.0006	up
XM_043771874.1	LOC122599368	uncharacterized LOC122599368	0.0015	up
XM_043766446.1	LOC122593971	uncharacterized LOC122593971	< 0.0001	up
XM_043769411.1	LOC122596780	rRNA-processing protein FCF1 homolog	< 0.0001	up
XR_006323494.1	LOC122597927	uncharacterized LOC122597927	0.0016	up
XM_043758959.1	LOC122586930	galactose mutarotase-like	< 0.0001	up
XM_043756156.1	LOC122583781	uncharacterized LOC122583781	0.0046	up
XM_043766018.1	LOC122593589	60S ribosomal protein L39-1	< 0.0001	up
XM_043767840.1	LOC122595473	protein arginine N-methyltransferase 1.1	0.0057	up
XM_043763636.1	LOC122591368	PHD finger protein Alfin1-like, transcript variant X2	0.0007	up
XM_043773163.1	LOC122600451	basic leucine zipper 9	0.0012	up
XM_043770565.1	LOC122597968	CMP-sialic acid transporter 1, transcript variant X5	0.0011	up
XM_043752441.1	LOC122580166	defensin SD2-like	< 0.0001	up

Transcript id	GeneID	Gene name/function	*P* value	result
XM_043762318.1	LOC122589987	probable (S)-N-methylcoclaurine 3'-hydroxylase isozyme 2	0.0040	up
XM_043775449.1	LOC122602844	formate--tetrahydrofolate ligase	0.0038	up
XM_043765566.1	LOC122593194	uncharacterized LOC122593194	0.0003	up
XM_043766643.1	LOC122594176	basic blue protein-like	< 0.0001	up
XM_043772694.1	LOC122600047	dolichyl-diphosphooligosaccharide--protein glycosyltransferase subunit DAD1	0.0003	up
XM_043753184.1	LOC122581046	probable serine/threonine-protein kinase PBL9, transcript variant X1	0.0009	up
XM_043772139.1	LOC122599607	leucine carboxyl methyltransferase 1 homolog	0.0012	up
XR_006323761.1	LOC122598855	uncharacterized LOC122598855	0.0047	up
XR_006324320.1	LOC122602566	uncharacterized LOC122602566	0.0007	up
XM_043757503.1	LOC122585370	methylsterol monooxygenase 1-1-like	0.0028	up
XM_043769285.1	LOC122596666	RNA pseudouridine synthase 3, mitochondrial-like	0.0009	up
XM_043779660.1	LOC122606805	polygalacturonase inhibitor-like	< 0.0001	up
XM_043752201.1	LOC122579934	root allergen protein-like	< 0.0001	up
XM_043756944.1	LOC122584872	MYG1 exonuclease-like	0.0039	up
XR_006320840.1	LOC122580689	U2 spliceosomal RNA	0.0042	up
XR_006324077.1	LOC122600616	uncharacterized LOC122600616	0.0014	up
XM_043753108.1	LOC122580958	autophagy-related protein 27, transcript variant X1	0.0009	up
XM_043756253.1	LOC122583880	NADH dehydrogenase [ubiquinone] 1 alpha subcomplex subunit 6	0.0021	up
XM_043777890.1	LOC122605023	ras-related protein RABE1c	< 0.0001	up
XM_043775258.1	LOC122602664	protein translocase subunit SecA 1	0.0008	up
XM_043778742.1	LOC122605787	zinc finger CCCH domain-containing protein 3	0.0022	up
XM_043767776.1	LOC122595423	peroxisomal (S)-2-hydroxy-acid oxidase GLO4-like	0.0050	up
XM_043778361.1	LOC122605413	transcription elongation factor TFIIS-like	0.0016	up
XM_043751652.1	LOC122579470	V-type proton ATPase subunit C-like	0.0004	up
XM_043751565.1	LOC122579392	ceramide synthase 1 LOH3-like	0.0013	up
XM_043778020.1	LOC122605140	aquaporin SIP1-2-like	0.0024	up
XM_043772183.1	LOC122599642	triphosphate tunnel metalloenzyme 3, transcript variant X2	0.0004	up
XM_043784287.1	LOC122611285	probable polyamine transporter At1g31830, transcript variant X1	0.0022	up
XM_043760679.1	LOC122588546	succinate dehydrogenase subunit 7B, mitochondrial-like	0.0020	up
XM_043763195.1	LOC122591011	CBL-interacting serine/threonine-protein kinase 23	< 0.0001	up
XM_043750938.1	LOC122578889	RHOMBOID-like protein 12, mitochondrial	< 0.0001	up
XM_043776690.1	LOC122603860	mediator of RNA polymerase II transcription subunit 11, transcript variant X2	0.0005	up
XM_043782887.1	LOC122609934	tubulin-folding cofactor C, transcript variant X2	< 0.0001	up
XM_043772538.1	LOC122599923	afadin- and alpha-actinin-binding protein-like	< 0.0001	up
XM_043752531.1	LOC122580252	novel plant SNARE 13	0.0006	up
XM_043766853.1	LOC122594388	zinc finger SWIM domain-containing protein 7	0.0020	up
XM_043765616.1	LOC122593234	pentatricopeptide repeat-containing protein At1g55890, mitochondrial-like	0.0004	up
XM_043765775.1	LOC122593379	EG45-like domain containing protein	0.0013	up
XM_043773304.1	LOC122600564	GABA transporter 1	< 0.0001	up
XM_043768828.1	LOC122596277	probable sodium/metabolite cotransporter BASS1, chloroplastic	< 0.0001	up
XM_043754192.1	LOC122581877	elongation factor P	0.0003	up
XM_043756796.1	LOC122584742	G-patch domain-containing protein 1-like	0.0029	up
XM_043757449.1	LOC122585321	LOB domain-containing protein 20-like	0.0011	up
XM_043754543.1	LOC122582184	protein tas-like	0.0002	up
XM_043770928.1	LOC122598334	LEAF RUST 10 DISEASE-RESISTANCE LOCUS RECEPTOR-LIKE PROTEIN KINASE-like 1.5	< 0.0001	up
XM_043753486.1	LOC122581285	60S ribosomal protein L10a-1	0.0055	up
XM_043782253.1	LOC122609192	calmodulin-7	0.0003	up
XM_043769097.1	LOC122596509	uncharacterized LOC122596509	0.0005	up
XM_043758790.1	LOC122586802	protease Do-like 7, transcript variant X2	0.0012	up
XM_043754270.1	LOC122581942	proliferating cell nuclear antigen	0.0032	up
XM_043768873.1	LOC122596321	acyl carrier protein 1, mitochondrial	0.0011	up
XM_043773515.1	LOC122600758	4-hydroxy-3-methylbut-2-enyl diphosphate reductase, chloroplastic-like	< 0.0001	up
XR_006320503.1	LOC122578042	uncharacterized LOC122578042	0.0032	up
XM_043775808.1	LOC122603174	glutamine synthetase	0.0058	up
XM_043754017.1	LOC122581745	AUGMIN subunit 7	0.0033	up
XM_043770669.1	LOC122598057	signaling peptide TAXIMIN 1	0.0004	up
XM_043775656.1	LOC122603039	DEAD-box ATP-dependent RNA helicase 8-like	0.0020	up
XM_043772001.1	LOC122599481	peptidyl-prolyl cis-trans isomerase CYP59	0.0013	up
XM_043774706.1	LOC122601973	uncharacterized LOC122601973	< 0.0001	up
XM_043778254.1	LOC122605327	mediator of RNA polymerase II transcription subunit 22a-like	0.0020	up
XM_043783096.1	LOC122610099	auxin response factor 4-like	< 0.0001	up
XM_043765114.1	LOC122592800	uncharacterized protein At5g48480-like	0.0022	up
XM_043771686.1	LOC122599220	calmodulin-binding receptor-like cytoplasmic kinase 3, transcript variant X1	0.0054	up

Transcript id	GeneID	Gene name/function	*P* value	result
XM_043762974.1	LOC122590806	tobamovirus multiplication protein 1	< 0.0001	up
XM_043755249.1	LOC122582816	protein RALF-like 24	0.0004	up
XM_043761856.1	LOC122589556	uncharacterized LOC122589556	< 0.0001	up
XM_043781025.1	LOC122607943	pollen-specific protein C13-like	0.0003	up
XM_043767094.1	LOC122594667	cold-inducible RNA-binding protein-like	0.0001	up
XM_043780508.1	LOC122607520	uncharacterized LOC122607520	0.0034	up
XM_043783109.1	LOC122610114	zingipain-2-like	0.0001	up
XM_043752166.1	LOC122579906	cytochrome c oxidase assembly factor 5	0.0002	up
XR_006325290.1	LOC122608698	protein PEP-RELATED DEVELOPMENT ARRESTED 1, chloroplastic, transcript variant X2	0.0017	up
XM_043755488.1	LOC122583044	norbelladine synthase-like	0.0051	up
XM_043753982.1	LOC122581718	SEC23-interacting protein-like, transcript variant X2	< 0.0001	up
XM_043773210.1	LOC122600488	J domain-containing protein spf31	0.0007	up
XM_043766715.1	LOC122594243	acanthoscurrin-2-like	< 0.0001	up
XM_043769130.1	LOC122596532	50S ribosomal protein L21, mitochondrial	0.0005	up
XM_043753678.1	LOC122581442	uncharacterized LOC122581442	< 0.0001	up
XM_043759391.1	LOC122587269	WUSCHEL-related homeobox 8-like, transcript variant X3	< 0.0001	up
XR_006324848.1	LOC122606272	protein MARD1-like, transcript variant X2	0.0018	up
XM_043779054.1	LOC122606091	altered inheritance rate of mitochondria protein 25	< 0.0001	up
XM_043769034.1	LOC122596454	60S ribosomal protein L37-2-like	< 0.0001	up
XM_043778134.1	LOC122605226	thaumatin-like protein 1	0.0005	up
XM_043752653.1	LOC122580383	peroxidase 10-like	0.0001	up
XM_043756836.1	LOC122584778	putative ALA-interacting subunit 2, transcript variant X1	0.0001	up
XM_043761750.1	LOC122589458	uncharacterized LOC122589458	< 0.0001	up
XM_043763144.1	LOC122590968	probable 3-beta-hydroxysteroid-Delta(8),Delta(7)-isomerase	0.0014	up
XM_043752162.1	LOC122579904	uncharacterized LOC122579904	0.0035	up
XM_043754391.1	LOC122582044	pectin acetylesterase 5-like	< 0.0001	up
XM_043775478.1	LOC122602869	60S ribosomal protein L23A, transcript variant X1	0.0002	up
XM_043750290.1	LOC122578345	cellulose synthase-like protein G3	0.0007	up
XM_043765318.1	LOC122592987	protein trichome birefringence-like 38	0.0047	up
XM_043752928.1	LOC122580658	ganglioside-induced differentiation-associated protein 2-like	< 0.0001	up
XM_043760874.1	LOC122588705	mitochondrial import receptor subunit TOM9-2	0.0002	up
XR_006321561.1	LOC122584580	28S ribosomal RNA	0.0056	up
XM_043766956.1	LOC122594511	BURP domain protein USPL1	< 0.0001	up
XM_043774650.1	LOC122601916	uncharacterized LOC122601916, transcript variant X2	0.0049	up
XM_043779906.1	LOC122606990	26S proteasome regulatory subunit 4 homolog A-like	< 0.0001	up
XM_043758767.1	LOC122586782	prostamide/prostaglandin F synthase, transcript variant X2	0.0001	up
XR_006324777.1	LOC122605984	uncharacterized LOC122605984	0.0015	up
XM_043751997.1	LOC122579770	eukaryotic translation initiation factor 3 subunit I-like	0.0048	up
XM_043750367.1	LOC122578411	AUGMIN subunit 5	0.0004	up
XM_043777965.1	LOC122605087	probable prolyl 4-hydroxylase 3, transcript variant X2	0.0002	up
XM_043774190.1	LOC122601430	protein NRT1/ PTR FAMILY 3.1-like	0.0051	up
XM_043764537.1	LOC122592334	telomere repeat-binding protein 5, transcript variant X1	0.0011	up
XM_043775874.1	LOC122603224	dolichyl-diphosphooligosaccharide--protein glycosyltransferase subunit STT3B, transcript variant X1	0.0028	up
XM_043775352.1	LOC122602758	endonuclease MutS2, transcript variant X1	0.0024	up
XM_043772346.1	LOC122599776	ATP-dependent RNA helicase DHX8, transcript variant X3	0.0013	up
XM_043770068.1	LOC122597478	putative lipid-transfer protein DIR1	0.0005	up
XM_043772644.1	LOC122600014	prolyl 4-hydroxylase 1	0.0017	up
XM_043757242.1	LOC122585137	expansin-like B1	< 0.0001	up
XM_043769243.1	LOC122596630	rRNA N6-adenosine-methyltransferase METTL5	0.0011	up
XM_043779134.1	LOC122606171	imidazoleglycerol-phosphate dehydratase 1, chloroplastic-like	0.0007	up
XM_043780529.1	LOC122607538	succinate dehydrogenase assembly factor 2, mitochondrial	< 0.0001	up
XM_043780100.1	LOC122607173	60S ribosomal protein L22-3-like	< 0.0001	up
XM_043773593.1	LOC122600822	uncharacterized LOC122600822	< 0.0001	up
XM_043750042.1	LOC122578147	40S ribosomal protein S12-like	0.0030	up
XR_006323775.1	LOC122598907	uncharacterized LOC122598907	0.0010	up
XM_043756172.1	LOC122583797	uncharacterized LOC122583797	0.0019	up
XM_043778104.1	LOC122605203	30S ribosomal protein S17-like, transcript variant X2	< 0.0001	up
XM_043755452.1	LOC122583013	protein FAR1-RELATED SEQUENCE 4, transcript variant X1	0.0003	up
XM_043772898.1	LOC122600218	pre-mRNA-splicing factor SPF27 homolog	0.0048	up
XM_043764604.1	LOC122592391	apoptosis inhibitor 5-like protein API5	0.0052	up
XM_043774562.1	LOC122601791	26S proteasome non-ATPase regulatory subunit 4 homolog, transcript variant X2	0.0006	up
XR_006323103.1	LOC122594747	small nucleolar RNA Z112	0.0027	up
XM_043762451.1	LOC122590117	mitochondrial outer membrane import complex protein METAXIN	0.0003	up
XM_043773415.1	LOC122600663	transcription factor LHW-like	0.0001	up

Transcript id	GeneID	Gene name/function	P value	result
XM_043768409.1	LOC122595931	U11/U12 small nuclear ribonucleoprotein 59 kDa protein, transcript variant X1	0.0035	up
XM_043764771.1	LOC122592521	ruvB-like protein 1	0.0027	up
XM_043781137.1	LOC122608054	uncharacterized LOC122608054	< 0.0001	up
XM_043754059.1	LOC122581780	root allergen protein-like	0.0002	up
XM_043780563.1	LOC122607559	uncharacterized protein At2g39795, mitochondrial-like, transcript variant X1	0.0020	up
XM_043769999.1	LOC122597400	uncharacterized LOC122597400	0.0057	up
XM_043782151.1	LOC122609090	uncharacterized LOC122609090	0.0059	up
XM_043780765.1	LOC122607736	26S proteasome regulatory subunit 8 homolog A-like	0.0002	up
XM_043751521.1	LOC122579357	single-stranded DNA-binding protein WHY1, chloroplastic-like	< 0.0001	up
XM_043773878.1	LOC122601104	delta-1-pyrroline-5-carboxylate synthase, transcript variant X1	0.0011	up
XM_043779790.1	LOC122606903	40S ribosomal protein S18	< 0.0001	up
XM_043778017.1	LOC122605138	probable U3 small nucleolar RNA-associated protein 11	0.0006	up
XM_043783365.1	LOC122610371	WD repeat-containing protein WRAP73	0.0048	up
XM_043782016.1	LOC122608948	uncharacterized LOC122608948	< 0.0001	up
XM_043752539.1	LOC122580260	probable sugar phosphate/phosphate translocator At3g17430	0.0006	up
XM_043772650.1	LOC122600020	3-hydroxyisobutyryl-CoA hydrolase-like protein 1, mitochondrial, transcript variant X1	0.0011	up
XM_043750443.1	LOC122578471	probable prolyl 4-hydroxylase 10	< 0.0001	up
XM_043750998.1	LOC122578939	50S ribosomal protein L17-like	0.0003	up
XM_043750055.1	LOC122578156	AP-1 complex subunit sigma-2, transcript variant X1	< 0.0001	up
XM_043765083.1	LOC122592774	metal tolerance protein B-like	0.0005	up
XM_043764701.1	LOC122592464	COP9 signalosome complex subunit 2	0.0056	up
XM_043765654.1	LOC122593266	malignant T-cell-amplified sequence 1-A	< 0.0001	up
XM_043772981.1	LOC122600278	uncharacterized LOC122600278	< 0.0001	up
XM_043776004.1	LOC122603327	phosphoenolpyruvate carboxykinase (ATP) 1-like	0.0022	up
XM_043763241.1	LOC122591052	pectin acetylesterase 10-like	< 0.0001	up
XM_043765846.1	LOC122593435	costars family protein-like	< 0.0001	up
XM_043773212.1	LOC122600490	cytochrome c oxidase subunit 5C-2	0.0033	up
XM_043767525.1	LOC122595194	protein RICE SALT SENSITIVE 3-like, transcript variant X2	< 0.0001	up
XM_043775237.1	LOC122602638	arogenate dehydrogenase 2, chloroplastic-like	< 0.0001	up
XM_043782036.1	LOC122608970	pentatricopeptide repeat-containing protein At1g60770	0.0046	up
XM_043780139.1	LOC122607209	ribonuclease 2-like	< 0.0001	up
XM_043762657.1	LOC122590455	glutathione S-transferase U8-like	< 0.0001	up
XM_043751762.1	LOC122579573	uncharacterized LOC122579573	< 0.0001	up
XM_043757337.1	LOC122585222	leucine aminopeptidase	0.0032	up
XM_043770841.1	LOC122598237	uncharacterized metal-dependent hydrolase YabD-like, transcript variant X2	0.0003	up
XM_043750261.1	LOC122578324	50S ribosomal protein L7/L12	0.0039	up
XM_043778952.1	LOC122605987	uncharacterized LOC122605987	< 0.0001	up
XM_043762698.1	LOC122590497	uncharacterized LOC122590497	0.0048	up
XM_043782445.1	LOC122609397	non-specific lipid transfer protein GPI-anchored 5-like	< 0.0001	up
XM_043751989.1	LOC122579761	uncharacterized LOC122579761	0.0007	up
XM_043753382.1	LOC122581210	nascent polypeptide-associated complex subunit alpha-like protein 1	< 0.0001	up
XR_006321548.1	LOC122584540	28S ribosomal RNA	0.0027	up
XM_043768142.1	LOC122595717	COP9 signalosome complex subunit 7-like, transcript variant X1	0.0005	up
XM_043767006.1	LOC122594568	ADP-ribosylation factor 1	0.0007	up
XM_043754787.1	LOC122582399	putative disease resistance protein RGA3	0.0040	up
XM_043755206.1	LOC122582773	bZIP transcription factor 53-like	< 0.0001	up
XM_043762667.1	LOC122590082	uncharacterized LOC122590082	0.0002	up
XM_043780395.1	LOC122607423	bZIP transcription factor 11-like	< 0.0001	up
XR_006325360.1	LOC122609610	small nucleolar RNA snoR122	0.0025	up
XM_043778581.1	LOC122605623	protein PLASTID TRANSCRIPTIONALLY ACTIVE 7, transcript variant X2	0.0012	up
XM_043766921.1	LOC122594470	proteasome subunit beta type-2-A-like	0.0003	up
XM_043752432.1	LOC122580160	uncharacterized protein slr1417	0.0002	up
XM_043763458.1	LOC122591227	epidermis-specific secreted glycoprotein EP1-like	< 0.0001	up
XM_043781732.1	LOC122608640	transcription initiation factor TFIID subunit 12b, transcript variant X2	0.0024	up
XM_043773179.1	LOC122600462	probable signal peptidase complex subunit 1, transcript variant X1	0.0048	up
XM_043754066.1	LOC122581786	uncharacterized LOC122581786	0.0015	up
XM_043761120.1	LOC122588900	protein RESPONSE TO LOW SULFUR 3-like	< 0.0001	up
XM_043768448.1	LOC122595962	IAA-alanine resistance protein 1	0.0016	up
XM_043780230.1	LOC122607291	uncharacterized LOC122607291	0.0047	up
XM_043753361.1	LOC122581192	sulfite exporter TauE/SafE family protein 3-like	< 0.0001	up
XM_043754113.1	LOC122581820	probable pyridoxal 5'-phosphate synthase subunit PDX2	0.0008	up
XM_043750057.1	LOC122578158	UDP-galactose transporter 1	< 0.0001	up
XM_043767981.1	LOC122595590	pyruvate decarboxylase 1, transcript variant X1	< 0.0001	up

Transcript id	GeneID	Gene name/function	*P* value	result
XM_043777537.1	LOC122604662	50S ribosomal protein L9, chloroplastic	< 0.0001	up
XM_043780269.1	LOC122607318	stromal cell-derived factor 2-like protein	0.0029	up
XM_043754691.1	LOC122582320	NADH dehydrogenase [ubiquinone] 1 beta subcomplex subunit 7	< 0.0001	up
XM_043780600.1	LOC122607592	patellin-6	< 0.0001	up
XR_006324892.1	LOC122606380	small nucleolar RNA U3	0.0021	up
XM_043758140.1	LOC122586024	isoprenylcysteine alpha-carbonyl methylesterase ICME-like	< 0.0001	up
XM_043761336.1	LOC122589092	dolichyl-phosphate beta-glucosyltransferase-like	0.0040	up
XM_043783377.1	LOC122610382	amino acid transporter AVT6A-like	0.0015	up
XM_043753568.1	LOC122581350	26S proteasome regulatory subunit 4 homolog A	0.0010	up
XM_043776005.1	LOC122603328	WD repeat-containing protein DWA2-like	0.0055	up
XM_043778003.1	LOC122605122	heavy metal-associated isoprenylated plant protein 21-like	0.0043	up
XM_043771959.1	LOC122599443	GTP-binding protein YPTM2	0.0059	up
XR_006324137.1	LOC122601995	uncharacterized LOC122601995	< 0.0001	up
XM_043780851.1	LOC122607802	carboxypeptidase SOL1	0.0011	up
XM_043780594.1	LOC122607587	cysteine-rich repeat secretory protein 55	< 0.0001	up
XM_043753936.1	LOC122581683	5'-deoxynucleotidase HDDC2	0.0028	up
XM_043763300.1	LOC122591097	uncharacterized LOC122591097	0.0036	up
XM_043761228.1	LOC122588999	protein DELETION OF SUV3 SUPPRESSOR 1(I)-like	0.0013	up
XM_043783559.1	LOC122610582	putative receptor protein kinase ZmPK1	0.0034	up
XM_043781147.1	LOC122608061	transmembrane protein 87A	0.0003	up
XM_043778977.1	LOC122606007	probable carboxylesterase 17	0.0041	up
XM_043755879.1	LOC122583480	LRR receptor-like serine/threonine-protein kinase RGI1	0.0007	up
XM_043773846.1	LOC122601068	60S ribosomal protein L35a-1-like	0.0035	up
XM_043771971.1	LOC122599456	RGG repeats nuclear RNA binding protein A-like, transcript variant X1	0.0013	up
XM_043756999.1	LOC122584919	uncharacterized LOC122584919, transcript variant X1	< 0.0001	up
XM_043781198.1	LOC122608109	sodium/hydrogen exchanger 6	0.0019	up
XR_006323048.1	LOC122594613	uncharacterized LOC122594613	< 0.0001	up
XM_043769319.1	LOC122596698	growth-regulating factor 6, transcript variant X2	< 0.0001	up
XM_043761245.1	LOC122589014	uncharacterized LOC122589014, transcript variant X3	< 0.0001	up
XM_043750461.1	LOC122578487	phosphoenolpyruvate carboxykinase (ATP) 1-like	< 0.0001	up
XM_043755250.1	LOC122582813	calcium-binding allergen Ole e 8-like	< 0.0001	up
XM_043768973.1	LOC122596402	60S acidic ribosomal protein P1-2-like	< 0.0001	up
XR_006324038.1	LOC122600350	uncharacterized LOC122600350	0.0020	up
XR_006321573.1	LOC122584665	uncharacterized LOC122584665, transcript variant X1	0.0048	up
XM_043768140.1	LOC122595714	elongation of fatty acids protein 3-like	0.0005	up
XM_043776846.1	LOC122603973	putative glutamine amidotransferase GAT1_2.1	0.0003	up
XM_043771829.1	LOC122599332	phosphatidylinositol 4-phosphate 5-kinase 7-like	< 0.0001	up
XM_043775319.1	LOC122602731	60S ribosomal protein L35a-1	0.0004	up
XM_043754739.1	LOC122582361	cationic peroxidase 1-like	0.0014	up
XM_043764595.1	LOC122592381	alpha-soluble NSF attachment protein-like	0.0008	up
XM_043769207.1	LOC122596596	uncharacterized LOC122596596	0.0002	up
XM_043762124.1	LOC122589804	cytochrome P450 81Q32-like	< 0.0001	up
XM_043778517.1	LOC122605560	uncharacterized LOC122605560	0.0031	up
XM_043773319.1	LOC122600579	protein cornichon homolog 4-like, transcript variant X1	0.0006	up
XR_006325466.1	LOC122611098	uncharacterized LOC122611098, transcript variant X3	0.0008	up
XM_043770293.1	LOC122597728	sugar transport protein 14	0.0022	up
XM_043765760.1	LOC122593365	magnesium-dependent phosphatase 1	0.0002	up
XM_043751180.1	LOC122579083	histone-lysine N-methyltransferase ASHH1	0.0009	up
XM_043757912.1	LOC122585787	60S ribosomal protein L6, mitochondrial-like	0.0047	up
XM_043780635.1	LOC122607619	uncharacterized LOC122607619	0.0003	up
XM_043754461.1	LOC122582101	trafficking protein particle complex subunit 2-like protein	< 0.0001	up
XM_043781453.1	LOC122608358	uncharacterized LOC122608358, transcript variant X2	0.0001	up
XM_043757521.1	LOC122585387	plasmodesmata-located protein 2-like	0.0013	up
XM_043753828.1	LOC122581590	BAG-associated GRAM protein 1, transcript variant X1	< 0.0001	up
XM_043768852.1	LOC122596303	protein gamma response 1	0.0013	up
XM_043769057.1	LOC122596477	F-box/kelch-repeat protein SKIP4	0.0042	up
XM_043767440.1	LOC122595124	plasma membrane ATPase 4	0.0015	up
XM_043761489.1	LOC122589230	H/ACA ribonucleoprotein complex subunit 2-like protein	< 0.0001	up
XM_043764485.1	LOC122592290	chaperone protein dnaJ 20, chloroplastic-like	< 0.0001	up
XM_043780367.1	LOC122607401	protein LIKE COV 2	0.0055	up
XM_043767843.1	LOC122595477	guanine nucleotide-binding protein alpha-1 subunit, transcript variant X1	0.0033	up
XM_043781554.1	LOC122608444	pathogen-related protein-like	0.0001	up
XR_006320669.1	LOC122579592	uncharacterized LOC122579592, transcript variant X2	0.0024	up
XM_043765882.1	LOC122593468	protein BOLA1, chloroplastic	< 0.0001	up
XM_043763609.1	LOC122591345	uncharacterized LOC122591345	0.0008	up
XM_043773598.1	LOC122600827	CRM-domain containing factor CFM3A, chloroplastic/mitochondrial-	0.0045	up
XM_043772306.1	LOC122599745	protein WVD2-like 3	< 0.0001	up

Transcript id	GeneID	Gene name/function	P value	result
XM_043782600.1	LOC122609572	magnesium-protoporphyrin IX monomethyl ester [oxidative] cyclase, chloroplastic	< 0.0001	up
XM_043765950.1	LOC122593530	probable tyrosine-protein phosphatase DSP4	< 0.0001	up
XM_043780926.1	LOC122607858	50S ribosomal protein L18-like, transcript variant X1	< 0.0001	up
XM_043781276.1	LOC122608186	uncharacterized LOC122608186	0.0051	up
XM_043768562.1	LOC122596056	N-acetyltransferase 9-like protein, transcript variant X1	0.0051	up
XM_043754563.1	LOC122582203	selenoprotein F	0.0003	up
XM_043776817.1	LOC122603950	protein EARLY RESPONSIVE TO DEHYDRATION 15-like	0.0002	up
XM_043755471.1	LOC122583035	uncharacterized LOC122583035, transcript variant X2	0.0019	up
XM_043769354.1	LOC122596729	apoptosis inhibitor 5-like protein API5	0.0002	up
XM_043755024.1	LOC122582610	mitochondrial Rho GTPase 1-like, transcript variant X2	0.0018	up
XM_043759234.1	LOC122587144	protein disulfide isomerase-like 1-6	0.0006	up
XM_043750698.1	LOC122578679	galactoside 2-alpha-L-fucosyltransferase-like	< 0.0001	up
XM_043773339.1	LOC122600594	WD repeat-containing protein 43, transcript variant X3	0.0029	up
XM_043767558.1	LOC122595222	DUF21 domain-containing protein At4g33700-like	< 0.0001	up
XM_043759172.1	LOC122587097	probable NADH dehydrogenase [ubiquinone] 1 alpha subcomplex subunit 5, mitochondrial, transcript variant X2	0.0024	up
XM_043751937.1	LOC122579714	uncharacterized LOC122579714	0.0005	up
XM_043764740.1	LOC122592497	protein PLASTID TRANSCRIPTIONALLY ACTIVE 14	0.0005	up
XM_043773106.1	LOC122600396	chromatin assembly factor 1 subunit A	0.0051	up
XR_006324716.1	LOC122605781	uncharacterized LOC122605781, transcript variant X1	0.0053	up
XM_043770419.1	LOC122597847	gibberellin-regulated protein 1-like	< 0.0001	up
XM_043753963.1	LOC122581706	ran-binding protein 1 homolog b-like	0.0010	up
XM_043777079.1	LOC122604180	traB domain-containing protein, transcript variant X1	0.0001	up
XM_043772385.1	LOC122599800	light-harvesting complex-like protein 3 isotype 2, chloroplastic	0.0001	up
XM_043769196.1	LOC122596587	proteasome subunit beta type-4-like	< 0.0001	up
XM_043775128.1	LOC122602514	uncharacterized LOC122602514, transcript variant X1	0.0050	up
XM_043760781.1	LOC122588625	cycloeucalenol cycloisomerase	< 0.0001	up
XM_043773436.1	LOC122600683	TSET complex member tstD-like	0.0045	up
XM_043751282.1	LOC122579169	protein PAM68, chloroplastic	0.0011	up
XM_043764861.1	LOC122592592	transmembrane protein 205	0.0009	up
XM_043778566.1	LOC122605606	uncharacterized LOC122605606	< 0.0001	up
XR_006325064.1	LOC122607550	uncharacterized LOC122607550, transcript variant X2	< 0.0001	up
XM_043759442.1	LOC122587312	protein RTE1-HOMOLOG-like, transcript variant X2	< 0.0001	up
XM_043757108.1	LOC122585014	bax inhibitor 1-like	0.0002	up
XM_043761669.1	LOC122589379	uncharacterized LOC122589379	< 0.0001	up
XM_043780991.1	LOC122607917	B3 domain-containing protein Os01g0723500-like	0.0015	up
XM_043771641.1	LOC122599179	putative germin-like protein 2-1	0.0045	up
XM_043762229.1	LOC122589900	NADPH:quinone oxidoreductase-like	0.0012	up
XM_043755194.1	LOC122582763	magnesium/proton exchanger	0.0001	up
XM_043781072.1	LOC122607986	GDSL esterase/lipase At3g26430-like	< 0.0001	up
XR_006322437.1	LOC122590283	serine palmitoyltransferase small subunit A, transcript variant X3	< 0.0001	up
XM_043777870.1	LOC122605009	uncharacterized LOC122605009, transcript variant X1	0.0012	up
XM_043750399.1	LOC122578436	stress-associated endoplasmic reticulum protein 2-like	< 0.0001	up
XM_043767771.1	LOC122595417	bifunctional purple acid phosphatase 26-like	0.0022	up
XM_043779901.1	LOC122606987	tropinone reductase homolog At2g29260, chloroplastic-like, transcript variant X2	0.0054	up
XM_043760683.1	LOC122588549	pyrroline-5-carboxylate reductase	0.0010	up
XM_043781841.1	LOC122608750	ubiquitin-NEDD8-like protein RUB2	< 0.0001	up
XM_043756307.1	LOC122583931	basic transcription factor 3-like	< 0.0001	up
XM_043766473.1	LOC122594002	glycogen phosphorylase 1-like, transcript variant X1	0.0006	up
XM_043781796.1	LOC122608702	ubiquitin-60S ribosomal protein L40	< 0.0001	up
XM_043780503.1	LOC122607514	uncharacterized protein At2g34160-like	< 0.0001	up
XM_043768941.1	LOC122596376	lon protease homolog 1, mitochondrial-like, transcript variant X2	< 0.0001	up
XM_043773262.1	LOC122600533	cytochrome c oxidase subunit 6b-3	0.0011	up
XM_043755133.1	LOC122582706	uncharacterized protein At2g27730, mitochondrial-like	0.0003	up
XM_043754669.1	LOC122582298	60S ribosomal protein L14-2-like	0.0004	up
XM_043753801.1	LOC122581567	protein REDUCED WALL ACETYLATION 3-like	< 0.0001	up
XM_043759284.1	LOC122587185	2-(3-amino-3-carboxypropyl)histidine synthase subunit 2	0.0003	up
XM_043770558.1	LOC122597964	60S ribosomal protein L37a	< 0.0001	up
XM_043750581.1	LOC122578586	F-box/FBD/LRR-repeat protein At1g13570-like, transcript variant X4	< 0.0001	up
XM_043759854.1	LOC122587688	uncharacterized LOC122587688	0.0013	up
XM_043766879.1	LOC122594416	pathogen-related protein	0.0003	up
XM_043756401.1	LOC122584050	uncharacterized protein At5g08430, transcript variant X1	0.0003	up
XM_043778019.1	LOC122605139	monodehydroascorbate reductase 4, peroxisomal	0.0032	up
XM_043751712.1	LOC122579529	protein DELETION OF SUV3 SUPPRESSOR 1(I)-like	0.0007	up
XM_043761401.1	LOC122589154	F-box/LRR-repeat/kelch-repeat protein At2g27520-like, transcript variant X1	0.0042	up

Transcript id	GeneID	Gene name/function	*P* value	result
XR_006323698.1	LOC122598706	U2 spliceosomal RNA	0.0006	up
XM_043753289.1	LOC122581133	nodal modulator 1	0.0007	up
XM_043782964.1	LOC122609996	transmembrane emp24 domain-containing protein p24beta2-like, transcript variant X2	0.0003	up
XM_043765392.1	LOC122593051	60S ribosomal protein L32-1, transcript variant X1	< 0.0001	up
XR_006320585.1	LOC122579021	general transcription and DNA repair factor IIH subunit TFB4, transcript variant X2	0.0003	up
XM_043769295.1	LOC122596676	uncharacterized LOC122596676	0.0005	up
XM_043783705.1	LOC122610743	uncharacterized LOC122610743	0.0003	up
XR_006323473.1	LOC122597825	uncharacterized LOC122597825, transcript variant X2	0.0009	up
XM_043768166.1	LOC122595738	very-long-chain 3-oxoacyl-CoA reductase 1-like	< 0.0001	up
XM_043754034.1	LOC122581760	aquaporin SIP1-2-like	0.0041	up
XM_043750363.1	LOC122578409	aspartate aminotransferase, cytoplasmic	< 0.0001	up
XM_043764349.1	LOC122592142	60S ribosomal protein L31-like	0.0036	up
XM_043776273.1	LOC122603546	G patch domain and ankyrin repeat-containing protein 1 homolog	0.0057	up
XM_043767378.1	LOC122595081	2-oxoglutarate-dependent dioxygenase 19-like	0.0024	up
XM_043776990.1	LOC122604090	probable pectate lyase 5	0.0011	up
XM_043773719.1	LOC122600940	uncharacterized LOC122600940	0.0017	up
XM_043782398.1	LOC122609343	ER membrane protein complex subunit 7 homolog	0.0020	up
XM_043771503.1	LOC122599054	3-hydroxyisobutyryl-CoA hydrolase-like protein 2, mitochondrial	< 0.0001	up
XM_043755359.1	LOC122582929	probable N-acetyltransferase HLS1-like	< 0.0001	up
XM_043782777.1	LOC122609830	kinesin-like protein KIN-14B, transcript variant X1	0.0009	up
XM_043770998.1	LOC122598399	serine carboxypeptidase-like 10, transcript variant X1	0.0018	up
XM_043766928.1	LOC122594481	peptide chain release factor PrfB2, chloroplastic	0.0004	up
XM_043772136.1	LOC122599604	soluble inorganic pyrophosphatase 6, chloroplastic-like	< 0.0001	up
XM_043752241.1	LOC122579977	replication protein A 70 kDa DNA-binding subunit A-like	0.0023	up
XM_043769144.1	LOC122596545	AUGMIN subunit 2	< 0.0001	up
XM_043760877.1	LOC122588707	probable arabinosyltransferase ARAD1	0.0022	up
XM_043776686.1	LOC122603856	cytochrome c oxidase subunit 6a, mitochondrial-like	< 0.0001	up
XM_043752361.1	LOC122580090	thioredoxin-like 1-2, chloroplastic	0.0037	up
XM_043776870.1	LOC122603993	F-box protein PP2-A15-like, transcript variant X3	0.0007	up
XM_043778556.1	LOC122605596	16 kDa phloem protein 1	0.0026	up
XM_043770510.1	LOC122597920	serine carboxypeptidase-like 31	< 0.0001	up
XM_043771944.1	LOC122599433	40S ribosomal protein S8	0.0032	up
XM_043778051.1	LOC122605160	plant cysteine oxidase 2	< 0.0001	up
XM_043776115.1	LOC122603406	protein LTO1 homolog, transcript variant X3	< 0.0001	up
XM_043769448.1	LOC122596804	uncharacterized LOC122596804, transcript variant X1	0.0003	up
XM_043771555.1	LOC122599094	14-3-3 protein 9	0.0031	up
XM_043771549.1	LOC122599090	uncharacterized membrane protein At4g09580-like	0.0021	up
XM_043762447.1	LOC122590112	glycosylphosphatidylinositol anchor biosynthesis protein 11	< 0.0001	up
XM_043779429.1	LOC122606587	sec14 cytosolic factor-like	0.0029	up
XM_043770609.1	LOC122598001	heavy metal-associated isoprenylated plant protein 33-like	0.0004	up
XM_043754513.1	LOC122582157	60S ribosomal protein L29-2-like	< 0.0001	up
XM_043768528.1	LOC122596028	homeobox-leucine zipper protein ATHB-15-like	< 0.0001	up
XM_043750492.1	LOC122578517	NAP1-related protein 2	< 0.0001	up
XM_043770513.1	LOC122597924	uncharacterized LOC122597924	0.0043	up
XM_043772873.1	LOC122600197	mediator of RNA polymerase II transcription subunit 7a	0.0004	up
XM_043761984.1	LOC122589668	uncharacterized LOC122589668	< 0.0001	up
XM_043762917.1	LOC122590742	ubiquitin-60S ribosomal protein L40, transcript variant X2	0.0004	up
XM_043780095.1	LOC122607166	mediator of RNA polymerase II transcription subunit 19a-like, transcript variant X1	0.0022	up
XM_043772647.1	LOC122600019	uncharacterized LOC122600019	0.0017	up
XM_043754185.1	LOC122581870	leucine-rich repeat receptor-like serine/threonine-protein kinase SKM1	0.0002	up
XM_043755247.1	LOC122582812	uncharacterized LOC122582812, transcript variant X2	0.0043	up
XM_043768918.1	LOC122596353	SKP1-like protein 1B	0.0011	up
XM_043750000.1	LOC122578115	26S proteasome non-ATPase regulatory subunit 7 homolog A	0.0028	up
XM_043762252.1	LOC122589920	uncharacterized LOC122589920	0.0003	up
XM_043757306.1	LOC122585194	21 kDa protein-like	< 0.0001	up
XM_043768182.1	LOC122595750	tetratricopeptide repeat protein 1	0.0001	up
XM_043768612.1	LOC122596092	omega-6 fatty acid desaturase, chloroplastic-like, transcript variant X1	0.0026	up
XM_043752882.1	LOC122580615	uncharacterized protein DDB_G0275933	0.0010	up
XM_043760747.1	LOC122588600	uncharacterized LOC122588600	0.0004	up
XR_006325214.1	LOC122608349	uncharacterized LOC122608349	< 0.0001	up
XR_006321547.1	LOC122584539	28S ribosomal RNA	0.0059	up
XM_043783157.1	LOC122610164	glycine-rich protein 5	0.0045	up
XM_043765907.1	LOC122593489	DDRGK domain-containing protein 1, transcript variant X1	< 0.0001	up
XM_043761579.1	LOC122589302	mitochondrial inner membrane protease ATP23	0.0036	up
XM_043764661.1	LOC122592436	katanin p60 ATPase-containing subunit A-like 2, transcript variant X1	0.0002	up

Transcript id	GeneID	Gene name/function	*P* value	result
XM_043781733.1	LOC122608638	photosynthetic NDH subunit of lumenal location 2, chloroplastic	0.0015	up
XM_043757889.1	LOC122585766	pantothenate kinase 1	0.0015	up
XR_006321928.1	LOC122586223	U1 spliceosomal RNA	0.0001	up
XM_043771644.1	LOC122599183	pectinesterase 2-like	< 0.0001	up
XR_006323188.1	LOC122595078	uncharacterized LOC122595078	0.0002	up
XM_043778326.1	LOC122605385	uncharacterized LOC122605385	0.0008	up
XM_043766303.1	LOC122593853	cytochrome b-c1 complex subunit 6-1, mitochondrial-like	0.0003	up
XM_043779380.1	LOC122606526	expansin-A4-like	0.0009	up
XM_043766000.1	LOC122593573	aspartate carbamoyltransferase, chloroplastic-like	0.0015	up
XM_043754577.1	LOC122582216	NADPH-dependent pterin aldehyde reductase, transcript variant X1	0.0033	up
XM_043761596.1	LOC122589314	myosin-1	0.0005	up
XR_006324177.1	LOC122602198	uncharacterized LOC122602198, transcript variant X2	0.0010	up
XM_043775012.1	LOC122602311	glycosyltransferase BC10-like	< 0.0001	up
XM_043776140.1	LOC122603440	actin-related protein 2	0.0055	up
XM_043770356.1	LOC122597787	mitochondrial inner membrane protease subunit 1	0.0040	up
XM_043778694.1	LOC122605738	uncharacterized LOC122605738	0.0049	up
XM_043751743.1	LOC122579553	3-epi-6-deoxocathasterone 23-monooxygenase CYP90C1	< 0.0001	up
XM_043773753.1	LOC122600972	probable glutathione S-transferase parC	< 0.0001	up
XM_043761337.1	LOC122589093	F-box protein At5g67140	0.0037	up
XM_043764289.1	LOC122592082	probable inorganic phosphate transporter 1-7	< 0.0001	up
XM_043768269.1	LOC122595821	rRNA (cytosine-C(5))-methyltransferase NOP2C, transcript variant X1	< 0.0001	up
XM_043772134.1	LOC122599602	grpE protein homolog 2, mitochondrial	0.0001	up
XM_043769067.1	LOC122596481	purple acid phosphatase 15	0.0031	up
XM_043778661.1	LOC122605700	30S ribosomal protein 3-1, chloroplastic-like	< 0.0001	up
XM_043771786.1	LOC122599292	beta-glucosidase 44-like	0.0003	up
XM_043764566.1	LOC122592358	phosphomannomutase	< 0.0001	up
XM_043764617.1	LOC122592400	protein SCAR1-like, transcript variant X2	< 0.0001	up
XM_043768487.1	LOC122595993	nucleolar and coiled-body phosphoprotein 1	0.0023	up
XM_043780703.1	LOC122607683	exosome complex component RRP4 homolog, transcript variant X1	0.0006	up
XM_043775163.1	LOC122602561	probable inactive receptor kinase At5g10020	0.0006	up
XM_043761660.1	LOC122589371	tyrosine--tRNA ligase 1, cytoplasmic-like, transcript variant X3	0.0021	up
XM_043751979.1	LOC122579750	peptidyl-prolyl cis-trans isomerase E	0.0002	up
XM_043753932.1	LOC122581681	uncharacterized LOC122581681	0.0007	up
XM_043750211.1	LOC122578286	stromal cell-derived factor 2-like protein	0.0057	up
XM_043769347.1	LOC122596722	U4/U6 small nuclear ribonucleoprotein PRP4-like protein	< 0.0001	up
XM_043754076.1	LOC122581796	40S ribosomal protein S14-3	< 0.0001	up
XM_043761685.1	LOC122589391	proteasome subunit alpha type-7	< 0.0001	up
XM_043753290.1	LOC122581134	sugar transporter ERD6-like 7, transcript variant X2	0.0006	up
XM_043754922.1	LOC122582517	cytochrome c oxidase-assembly factor COX23, mitochondrial	0.0003	up
XM_043766435.1	LOC122593959	proteasome subunit alpha type-4	0.0008	up
XR_006323697.1	LOC122598705	U1 spliceosomal RNA	0.0054	up
XM_043776880.1	LOC122604000	signal peptidase complex subunit 3A-like	0.0010	up
XM_043764953.1	LOC122592669	eukaryotic translation initiation factor 1A	0.0016	up
XM_043752094.1	LOC122579844	NAC domain-containing protein 21/22-like	0.0034	up
XM_043764667.1	LOC122592441	probable purine permease 11, transcript variant X3	< 0.0001	up
XM_043783080.1	LOC122610090	glycosyl hydrolase 5 family protein-like	0.0048	up
XM_043776433.1	LOC122603664	60S acidic ribosomal protein P2-like	0.0002	up
XM_043754492.1	LOC122582134	P-loop NTPase domain-containing protein LPA1 homolog 2	0.0001	up
XM_043781347.1	LOC122608263	mitochondrial import receptor subunit TOM5 homolog	< 0.0001	up
XM_043753344.1	LOC122581175	transcription initiation factor TFIID subunit 13, transcript variant X1	< 0.0001	up
XM_043780840.1	LOC122607792	protein EMBRYO DEFECTIVE 514	0.0020	up
XM_043776496.1	LOC122603714	acyl-coenzyme A thioesterase 13	0.0028	up
XM_043765442.1	LOC122593091	glyoxylate/hydroxypyruvate reductase HPR3-like	0.0021	up
XM_043760809.1	LOC122588653	F-box protein At1g55000	0.0039	up
XM_043772327.1	LOC122599762	protein argonaute 16-like	0.0014	up
XM_043762751.1	LOC122590552	uncharacterized LOC122590552	< 0.0001	up
XM_043765989.1	LOC122593563	N-terminal acetyltransferase B complex catalytic subunit NAA20	0.0005	up
XM_043751623.1	LOC122579443	uncharacterized LOC122579443	< 0.0001	up
XM_043766775.1	LOC122594298	uncharacterized LOC122594298	0.0058	up
XR_006324039.1	LOC122600362	uncharacterized LOC122600362	< 0.0001	up
XM_043763559.1	LOC122591306	protein SCAR2	0.0008	up
XM_043751582.1	LOC122579409	60S ribosomal protein L38-like	< 0.0001	up
XM_043757027.1	LOC122584939	uncharacterized LOC122584939, transcript variant X2	0.0029	up
XR_006325363.1	LOC122609665	uncharacterized LOC122609665	< 0.0001	up
XM_043764923.1	LOC122592640	protein SEMI-ROLLED LEAF 2	0.0036	up
XR_006321644.1	LOC122585045	uncharacterized LOC122585045	0.0026	up
XM_043751384.1	**LOC122579255**	defensin SD2-like, transcript variant X2	0.0002	up
XM_043751393.1	**LOC122579255**	defensin SD2-like, transcript variant X2	< 0.0001	up

Transcript id	GeneID	Gene name/function	*P* value	result
XM_043751578.1	**LOC122579406**	protein NUCLEAR FUSION DEFECTIVE 6, mitochondrial-like, transcript variant X2	0.0021	up
XM_043751577.1	**LOC122579406**	protein NUCLEAR FUSION DEFECTIVE 6, mitochondrial-like, transcript variant X2	< 0.0001	up
XM_043753006.1	**LOC122580854**	uncharacterized LOC122580854, transcript variant X1	0.0005	up
XM_043753007.1	**LOC122580854**	uncharacterized LOC122580854, transcript variant X1	0.0022	up
XM_043758187.1	**LOC122586082**	deoxyhypusine synthase, transcript variant X3	0.0003	up
XM_043758189.1	**LOC122586082**	deoxyhypusine synthase, transcript variant X3	0.0048	up
XM_043758401.1	**LOC122586406**	serine carboxypeptidase-like 12, transcript variant X2	< 0.0001	up
XM_043758402.1	**LOC122586406**	serine carboxypeptidase-like 12, transcript variant X2	< 0.0001	up
XM_043759383.1	**LOC122587267**	60S ribosomal protein L35a-1-like, transcript variant X2	< 0.0001	up
XM_043759385.1	**LOC122587267**	60S ribosomal protein L35a-1-like, transcript variant X2	< 0.0001	up
XM_043759382.1	**LOC122587267**	60S ribosomal protein L35a-1-like, transcript variant X2	< 0.0001	up
XM_043759414.1	**LOC122587288**	inositol-phosphate phosphatase-like, transcript variant X2	0.0011	up
XM_043759413.1	**LOC122587288**	inositol-phosphate phosphatase-like, transcript variant X2	0.0003	up
XM_043759411.1	**LOC122587288**	inositol-phosphate phosphatase-like, transcript variant X2	0.0043	up
XM_043760567.1	**LOC122588448**	protein SET DOMAIN GROUP 41, transcript variant X2	0.0014	up
XM_043760565.1	**LOC122588448**	protein SET DOMAIN GROUP 41, transcript variant X2	0.0035	up
XR_006322237.1	**LOC122589186**	uncharacterized LOC122589186, transcript variant X2	0.0047	up
XM_043761436.1	**LOC122589186**	uncharacterized LOC122589186, transcript variant X2	< 0.0001	up
XM_043761544.1	**LOC122589270**	transport protein particle 20 kDa subunit, transcript variant X2	< 0.0001	up
XM_043761543.1	**LOC122589270**	transport protein particle 20 kDa subunit, transcript variant X2	0.0001	up
XM_043761721.1	**LOC122589431**	vesicle transport protein SFT2B, transcript variant X1	0.0006	up
XM_043761723.1	**LOC122589431**	vesicle transport protein SFT2B, transcript variant X1	0.0003	up
XM_043762910.1	**LOC122590735**	EG45-like domain containing protein, transcript variant X2	0.0004	up
XM_043762912.1	**LOC122590735**	EG45-like domain containing protein, transcript variant X2	< 0.0001	up
XM_043764875.1	**LOC122592602**	probable pyridoxal 5'-phosphate synthase subunit PDX1, transcript variant X2	< 0.0001	up
XM_043764877.1	**LOC122592602**	probable pyridoxal 5'-phosphate synthase subunit PDX1, transcript variant X2	0.0013	up
XM_043765513.1	**LOC122593149**	zinc finger protein CONSTANS-LIKE 9-like, transcript variant X1	< 0.0001	up
XM_043765514.1	**LOC122593149**	zinc finger protein CONSTANS-LIKE 9-like, transcript variant X1	< 0.0001	up
XM_043766069.1	**LOC122593640**	mitochondrial import inner membrane translocase subunit TIM10-like, transcript variant X1	< 0.0001	up
XM_043766068.1	**LOC122593640**	mitochondrial import inner membrane translocase subunit TIM10-like, transcript variant X1	< 0.0001	up
XM_043767973.1	**LOC122595586**	uncharacterized LOC122595586, transcript variant X1	0.0006	up
XM_043767974.1	**LOC122595586**	uncharacterized LOC122595586, transcript variant X1	< 0.0001	up
XM_043768418.1	**LOC122595939**	mini-ribonuclease 3, transcript variant X2	< 0.0001	up
XM_043768417.1	**LOC122595939**	mini-ribonuclease 3, transcript variant X2	< 0.0001	up
XM_043768997.1	**LOC122596422**	2-hydroxy-palmitic acid dioxygenase MPO1-like, transcript variant X2	0.0001	up
XM_043768996.1	**LOC122596422**	2-hydroxy-palmitic acid dioxygenase MPO1-like, transcript variant X2	< 0.0001	up
XM_043769062.1	**LOC122596480**	DCN1-like protein 4, transcript variant X1	0.0017	up
XM_043769061.1	**LOC122596480**	DCN1-like protein 4, transcript variant X1	< 0.0001	up
XM_043769122.1	**LOC122596525**	protein NUCLEAR FUSION DEFECTIVE 6, mitochondrial-like, transcript variant X2	0.0011	up
XM_043769121.1	**LOC122596525**	protein NUCLEAR FUSION DEFECTIVE 6, mitochondrial-like, transcript variant X2	< 0.0001	up
XM_043769119.1	**LOC122596525**	protein NUCLEAR FUSION DEFECTIVE 6, mitochondrial-like, transcript variant X2	< 0.0001	up
XM_043769271.1	**LOC122596654**	lysine-specific demethylase JMJ30, transcript variant X1	0.0043	up
XM_043769279.1	**LOC122596654**	lysine-specific demethylase JMJ30, transcript variant X1	< 0.0001	up
XM_043770662.1	**LOC122598053**	protein NONRESPONDING TO OXYLIPINS 2, mitochondrial, transcript variant X2	0.0013	up
XM_043770663.1	**LOC122598053**	protein NONRESPONDING TO OXYLIPINS 2, mitochondrial, transcript variant X2	0.0009	up
XM_043772628.1	**LOC122600000**	uncharacterized protein At2g34160-like, transcript variant X2	< 0.0001	up
XM_043772629.1	**LOC122600000**	uncharacterized protein At2g34160-like, transcript variant X2	< 0.0001	up
XM_043772665.1	**LOC122600030**	protein NONRESPONDING TO OXYLIPINS 2, mitochondrial-like, transcript variant X6	0.0038	up
XM_043772669.1	**LOC122600030**	protein NONRESPONDING TO OXYLIPINS 2, mitochondrial-like, transcript variant X6	0.0020	up
XM_043772666.1	**LOC122600030**	protein NONRESPONDING TO OXYLIPINS 2, mitochondrial-like, transcript variant X6	0.0051	up
XM_043773082.1	**LOC122600374**	RGG repeats nuclear RNA binding protein A-like, transcript variant X2	0.0024	up
XM_043773083.1	**LOC122600374**	RGG repeats nuclear RNA binding protein A-like, transcript variant X2	0.0008	up
XM_043773105.1	**LOC122600395**	uncharacterized LOC122600395, transcript variant X2	< 0.0001	up
XM_043773103.1	**LOC122600395**	uncharacterized LOC122600395, transcript variant X2	0.0011	up
XM_043775585.1	**LOC122602958**	secretory carrier-associated membrane protein 1-like, transcript variant X1	0.0011	up

Transcript id	GeneID	Gene name/function	P value	result
XM_043775577.1	**LOC122602958**	secretory carrier-associated membrane protein 1-like, transcript variant X1	< 0.0001	up
XM_043776746.1	**LOC122603900**	50S ribosomal protein L7/L12, transcript variant X1	0.0002	up
XM_043776745.1	**LOC122603900**	50S ribosomal protein L7/L12, transcript variant X1	0.0011	up
XM_043779175.1	**LOC122606217**	coiled-coil domain-containing protein 12-like, transcript variant X4	0.0023	up
XM_043779176.1	**LOC122606217**	coiled-coil domain-containing protein 12-like, transcript variant X4	0.0041	up
XM_043781164.1	**LOC122608079**	protein DELETION OF SUV3 SUPPRESSOR 1(I)-like, transcript variant X2	< 0.0001	up
XM_043781166.1	**LOC122608079**	protein DELETION OF SUV3 SUPPRESSOR 1(I)-like, transcript variant X2	< 0.0001	up
XR_006325189.1	**LOC122608243**	uncharacterized LOC122608243, transcript variant X2	0.0056	up
XR_006325190.1	**LOC122608243**	uncharacterized LOC122608243, transcript variant X2	0.0020	up
XM_043783634.1	**LOC122610653**	60S ribosomal protein L29-1-like, transcript variant X1	0.0013	up
XM_043783626.1	**LOC122610653**	60S ribosomal protein L29-1-like, transcript variant X1	< 0.0001	up
XM_043784094.1	**LOC122611124**	ethylene-responsive transcription factor RAP2-3, transcript variant X1	0.0025	up
XM_043784095.1	**LOC122611124**	ethylene-responsive transcription factor RAP2-3, transcript variant X1	0.0012	up
XM_043751535.1	LOC122579369	cytokinin dehydrogenase 5-like	< 0.0001	down
XM_043773770.1	LOC122600991	protein DOG1-like 3	0.0021	down
XR_006324608.1	LOC122605442	uncharacterized LOC122605442, transcript variant X4	0.0007	down
XM_043776781.1	LOC122603925	ethylene-responsive transcription factor RAP2-7-like	< 0.0001	down
XM_043763044.1	LOC122590867	protein PELPK1-like	< 0.0001	down
XM_043755824.1	LOC122583419	uncharacterized LOC122583419	< 0.0001	down
XM_043753886.1	LOC122581640	glycine-rich protein A3-like	< 0.0001	down
XM_043772519.1	LOC122599910	probable protein kinase At2g41970, transcript variant X1	0.0013	down
XM_043760718.1	LOC122588579	E3 ubiquitin-protein ligase MBR2-like	0.0021	down
XM_043771646.1	LOC122599185	AAA-ATPase At3g28580-like	0.0032	down
XM_043781225.1	LOC122608134	patatin-like protein 2	0.0002	down
XM_043755911.1	LOC122583515	uncharacterized protein At4g26485-like	0.0020	down
XM_043769823.1	LOC122597198	uncharacterized LOC122597198	< 0.0001	down
XM_043756901.1	LOC122584838	uncharacterized LOC122584838	< 0.0001	down
XM_043758817.1	LOC122586823	uncharacterized LOC122586823	< 0.0001	down
XM_043766305.1	LOC122593856	CCR4-NOT transcription complex subunit 1-like	< 0.0001	down
XM_043778061.1	LOC122605167	2-methylpropanoate--CoA ligase CCL4-like	< 0.0001	down
XM_043784317.1	LOC122611307	protein WALLS ARE THIN 1-like	0.0031	down
XM_043751202.1	LOC122579101	elongation of fatty acids protein 3-like	0.0029	down
XM_043775529.1	LOC122602916	auxin-induced protein PCNT115-like, transcript variant X2	< 0.0001	down
XM_043768584.1	LOC122596068	receptor-like protein EIX1	< 0.0001	down
XR_006322896.1	LOC122593892	uncharacterized LOC122593892, transcript variant X2	0.0013	down
XM_043768504.1	LOC122596008	cucumber peeling cupredoxin-like	0.0049	down
XM_043771740.1	LOC122599259	probable 1-deoxy-D-xylulose-5-phosphate synthase 2, chloroplastic	< 0.0001	down
XM_043753870.1	LOC122581628	F-box/kelch-repeat protein At3g24760	< 0.0001	down
XM_043768853.1	LOC122596304	cytochrome P450 78A5-like	< 0.0001	down
XM_043777185.1	LOC122604298	uncharacterized LOC122604298	0.0005	down
XM_043754752.1	LOC122582369	heterogeneous nuclear ribonucleoprotein U-like protein 1, transcript variant X2	0.0006	down
XM_043780454.1	LOC122607476	mitochondrial uncoupling protein 1, transcript variant X1	< 0.0001	down
XM_043760835.1	LOC122588671	AP2-like ethylene-responsive transcription factor ANT, transcript variant X3	0.0004	down
XM_043780145.1	LOC122607206	protein trichome birefringence-like 16, transcript variant X2	0.0044	down
XM_043773400.1	LOC122600650	tubby-like F-box protein 8, transcript variant X3	< 0.0001	down
XM_043765796.1	LOC122593394	3-oxoacyl-[acyl-carrier-protein] reductase FabG-like	< 0.0001	down
XM_043754070.1	LOC122581791	protein TRANSPORT INHIBITOR RESPONSE 1-like	0.0006	down
XM_043759653.1	LOC122587470	ruBisCO large subunit-binding protein subunit beta, chloroplastic-like, transcript variant X2	0.0020	down
XM_043763162.1	LOC122590985	shaggy-related protein kinase eta-like, transcript variant X1	< 0.0001	down
XM_043776645.1	LOC122603822	putative oxidoreductase TDA3	< 0.0001	down
XM_043762651.1	LOC122590447	auxin-induced protein 15A-like	0.0001	down
XM_043776651.1	LOC122603826	probable glucuronoxylan glucuronosyltransferase IRX7, transcript variant X2	0.0032	down
XM_043759351.1	LOC122587242	protein NRT1/ PTR FAMILY 4.6-like	0.0010	down
XM_043775551.1	LOC122602937	copper transport protein CCH-like	0.0024	down
XM_043763511.1	LOC122591271	putative glucose-6-phosphate 1-epimerase, transcript variant X2	0.0003	down
XM_043749946.1	LOC122578077	trans-cinnamate:CoA ligase, peroxisomal-like	0.0001	down
XM_043761570.1	LOC122589293	bZIP transcription factor 27-like, transcript variant X2	0.0015	down
XM_043755547.1	LOC122583110	protein SCARECROW-like	0.0041	down
XM_043773557.1	LOC122600794	transcription factor MYB17-like	0.0025	down
XM_043758569.1	LOC122586584	PI-PLC X domain-containing protein At5g67130-like	0.0014	down
XM_043750121.1	LOC122578211	splicing factor-like protein 1, transcript variant X3	< 0.0001	down
XM_043756802.1	LOC122584749	ethylene-responsive transcription factor TINY-like	< 0.0001	down

Transcript id	GeneID	Gene name/function	*P* value	result
XM_043756065.1	LOC122583681	uncharacterized LOC122583681	< 0.0001	down
XM_043757797.1	LOC122585668	uncharacterized LOC122585668	0.0019	down
XM_043758186.1	LOC122586081	acidic endochitinase-like	0.0012	down
XM_043757153.1	LOC122585054	uncharacterized LOC122585054	0.0049	down
XM_043771051.1	LOC122598441	ubiquitin fusion degradation protein 1 homolog, transcript variant X2	< 0.0001	down
XM_043783708.1	LOC122610747	uncharacterized LOC122610747	0.0004	down
XM_043772445.1	LOC122599856	phosphatidylinositol 4-phosphate 5-kinase 8-like, transcript variant X1	0.0058	down
XM_043779861.1	LOC122606958	mitochondrial amidoxime reducing component 2-like, transcript variant X1	< 0.0001	down
XM_043782575.1	LOC122609541	protein LURP-one-related 15-like	0.0021	down
XM_043757064.1	LOC122584971	uncharacterized LOC122584971	0.0031	down
XM_043771545.1	LOC122599088	beta-amyrin synthase 1-like, transcript variant X1	< 0.0001	down
XM_043757586.1	LOC122585457	plastocyanin-like	0.0034	down
XM_043752489.1	LOC122580211	alpha,alpha-trehalose-phosphate synthase [UDP-forming] 5	0.0044	down
XM_043772387.1	LOC122599802	E3 ubiquitin ligase PARAQUAT TOLERANCE 3-like, transcript variant X2	< 0.0001	down
XM_043756289.1	LOC122583917	ankyrin repeat-containing protein At5g02620-like	0.0037	down
XM_043765887.1	LOC122593473	putative UPF0481 protein At3g02645, transcript variant X2	0.0024	down
XM_043775689.1	LOC122603077	lanC-like protein GCL1	< 0.0001	down
XM_043774689.1	LOC122601924	disease resistance protein RUN1-like	0.0030	down
XM_043781503.1	LOC122608402	uncharacterized LOC122608402, transcript variant X1	0.0023	down
XM_043753099.1	LOC122580951	thioredoxin H-type-like	0.0022	down
XM_043761471.1	LOC122589219	cyclin-dependent kinase G-2-like	0.0022	down
XM_043761875.1	LOC122589572	mitogen-activated protein kinase kinase kinase 20-like	0.0001	down
XM_043774939.1	LOC122602226	carotenoid cleavage dioxygenase 7, chloroplastic	0.0018	down
XM_043754240.1	LOC122581920	uncharacterized LOC122581920, transcript variant X1	0.0003	down
XM_043751791.1	LOC122579596	uncharacterized LOC122579596, transcript variant X3	< 0.0001	down
XR_006320986.1	LOC122581230	micronuclear linker histone polyprotein, transcript variant X3	0.0009	down
XM_043772837.1	LOC122600164	phosphoenolpyruvate carboxylase 4-like	< 0.0001	down
XM_043775334.1	LOC122602745	uncharacterized LOC122602745, transcript variant X1	< 0.0001	down
XM_043779597.1	LOC122606739	1-acyl-sn-glycerol-3-phosphate acyltransferase 2-like	0.0003	down
XM_043780165.1	LOC122607227	NAC transcription factor 32-like	< 0.0001	down
XM_043769171.1	LOC122596561	dirigent protein 18-like	0.0003	down
XM_043775838.1	LOC122603196	BAG family molecular chaperone regulator 3-like	< 0.0001	down
XM_043776236.1	LOC122603511	splicing factor U2af small subunit B-like, transcript variant X2	0.0002	down
XM_043762712.1	LOC122590514	disease resistance protein RGA5-like	0.0019	down
XM_043780871.1	LOC122607819	serine/threonine-protein kinase-like protein CCR4	0.0028	down
XM_043770716.1	LOC122598115	membrane protein PM19L-like	0.0002	down
XM_043760973.1	LOC122588777	post-GPI attachment to proteins factor 3-like	< 0.0001	down
XM_043781693.1	LOC122608596	UDP-glycosyltransferase 75C1-like	0.0019	down
XM_043783841.1	LOC122610885	transcription factor WER-like	0.0007	down
XR_006324821.1	LOC122606139	B-box zinc finger protein 19-like, transcript variant X3	< 0.0001	down
XM_043761603.1	LOC122589318	L-ascorbate oxidase	0.0020	down
XR_006322277.1	LOC122589531	uncharacterized LOC122589531	< 0.0001	down
XM_043757747.1	LOC122585622	pyruvate kinase 1, cytosolic-like	0.0001	down
XM_043757899.1	LOC122585775	dehydration-responsive element-binding protein 1D-like	0.0002	down
XR_006322330.1	LOC122589763	uncharacterized LOC122589763, transcript variant X3	< 0.0001	down
XM_043781743.1	LOC122608648	casparian strip membrane protein 1	0.0003	down
XM_043783532.1	LOC122610553	glutamate receptor 2.8-like	0.0004	down
XM_043751695.1	LOC122579512	uncharacterized LOC122579512	< 0.0001	down
XM_043766687.1	LOC122594214	polyubiquitin-like	< 0.0001	down
XM_043781293.1	LOC122608206	protein LIFEGUARD 2-like	0.0043	down
XM_043757222.1	LOC122585123	dof zinc finger protein DOF2.1	< 0.0001	down
XM_043769857.1	LOC122597239	calmodulin-binding receptor-like cytoplasmic kinase 2	0.0003	down
XM_043766024.1	LOC122593594	dof zinc finger protein DOF3.2-like	0.0006	down
XM_043766599.1	LOC122594121	septum-promoting GTP-binding protein 1	< 0.0001	down
XM_043773447.1	LOC122600694	uncharacterized LOC122600694	0.0003	down
XM_043782761.1	LOC122609817	uncharacterized LOC122609817, transcript variant X2	< 0.0001	down
XM_043755177.1	LOC122582750	probable protein phosphatase 2C 4	0.0043	down
XM_043772176.1	LOC122599637	protein ENHANCED DISEASE RESISTANCE 2-like	< 0.0001	down
XM_043782987.1	LOC122610017	serine protease SPPA, chloroplastic-like, transcript variant X2	0.0038	down
XM_043768327.1	LOC122595864	potassium channel AKT1	0.0005	down
XM_043784057.1	LOC122611093	putative potassium transporter 12, transcript variant X1	0.0004	down
XM_043760255.1	LOC122588116	uncharacterized LOC122588116	0.0003	down
XM_043776265.1	LOC122603537	hydroquinone glucosyltransferase-like	0.0010	down
XM_043752583.1	LOC122580307	protein CHROMATIN REMODELING 19	0.0017	down
XM_043765976.1	LOC122593552	uncharacterized LOC122593552	< 0.0001	down
XM_043775540.1	LOC122602926	putative disease resistance protein At1g59780, transcript variant X1	0.0001	down

Transcript id	GeneID	Gene name/function	*P* value	result
XM_043772809.1	LOC122600142	bHLH transcription factor RHL1-like	< 0.0001	down
XM_043767221.1	LOC122594934	protein SPA1-RELATED 3-like, transcript variant X4	0.0025	down
XM_043762078.1	LOC122589749	omega-hydroxypalmitate O-feruloyl transferase-like	0.0017	down
XM_043779850.1	LOC122606950	SNAP25 homologous protein SNAP33-like	< 0.0001	down
XM_043779161.1	LOC122606202	em-like protein GEA6	0.0001	down
XM_043755036.1	LOC122582618	uncharacterized LOC122582618	0.0027	down
XM_043751590.1	LOC122579417	putative peptidyl-tRNA hydrolase PTRHD1	< 0.0001	down
XM_043771664.1	LOC122599199	aquaporin PIP2-7	< 0.0001	down
XM_043755265.1	LOC122582829	eukaryotic translation initiation factor 5B-like	0.0020	down
XM_043761496.1	LOC122589237	uncharacterized LOC122589237	0.0009	down
XM_043776442.1	LOC122603671	uncharacterized LOC122603671, transcript variant X1	0.0003	down
XM_043774998.1	LOC122602295	protein PELPK2-like	0.0029	down
XM_043752352.1	LOC122580082	peroxidase 9-like	< 0.0001	down
XM_043760313.1	LOC122588188	rust resistance kinase Lr10-like	< 0.0001	down
XM_043759302.1	LOC122587200	uncharacterized LOC122587200	< 0.0001	down
XM_043763058.1	LOC122590877	putative receptor-like protein kinase At5g39000	< 0.0001	down
XM_043757069.1	LOC122584976	receptor-like kinase TMK4	< 0.0001	down
XM_043758219.1	LOC122586110	uncharacterized LOC122586110	0.0010	down
XM_043757075.1	LOC122584981	uncharacterized LOC122584981	0.0005	down
XM_043762685.1	LOC122590482	protein SRG1-like	< 0.0001	down
XM_043754564.1	LOC122582204	uncharacterized LOC122582204	0.0021	down
XR_006320717.1	LOC122579958	uncharacterized LOC122579958	0.0003	down
XM_043781368.1	LOC122608280	high mobility group B protein 1, transcript variant X2	0.0027	down
XM_043781077.1	LOC122607995	acyl-protein thioesterase 2-like	< 0.0001	down
XM_043778378.1	LOC122605418	U-box domain-containing protein 45-like, transcript variant X1	0.0058	down
XM_043761554.1	LOC122589279	receptor protein-tyrosine kinase CEPR1-like	0.0002	down
XM_043780960.1	LOC122607889	serine/threonine protein phosphatase 2A 55 kDa regulatory subunit B beta isoform-like, transcript variant X3	0.0007	down
XM_043756714.1	LOC122584670	receptor-like kinase TMK4	0.0001	down
XM_043765352.1	LOC122593015	probable protein phosphatase 2C 23	0.0002	down
XM_043772537.1	LOC122599921	vacuolar-processing enzyme-like	0.0002	down
XM_043753373.1	LOC122581203	uncharacterized LOC122581203	0.0017	down
XM_043778922.1	LOC122605958	UDP-glycosyltransferase 91D1-like	0.0004	down
XM_043777021.1	LOC122604120	uncharacterized LOC122604120	< 0.0001	down
XM_043780793.1	LOC122607761	uncharacterized LOC122607761	0.0006	down
XM_043754531.1	LOC122582172	protein NAR1, transcript variant X2	< 0.0001	down
XM_043764918.1	LOC122592635	probable glycerol-3-phosphate acyltransferase 8	< 0.0001	down
XM_043769229.1	LOC122596617	2-hydroxyisoflavanone dehydratase-like	0.0003	down
XM_043778817.1	LOC122605860	uncharacterized LOC122605860	< 0.0001	down
XM_043776455.1	LOC122603682	probable glycosyltransferase At5g25310	< 0.0001	down
XM_043757498.1	LOC122585366	uncharacterized LOC122585366	< 0.0001	down
XM_043768000.1	LOC122595601	peroxidase 60-like	< 0.0001	down
XM_043766828.1	LOC122594362	UPF0496 protein At4g34320-like	< 0.0001	down
XM_043777283.1	LOC122604386	protein SRC2-like	< 0.0001	down
XM_043780650.1	LOC122607634	protein REVEILLE 1-like	< 0.0001	down
XM_043783468.1	LOC122610481	receptor-like kinase TMK4	0.0008	down
XM_043776233.1	LOC122603510	mechanosensitive ion channel protein 6-like	< 0.0001	down
XM_043768266.1	LOC122595817	scarecrow-like protein 13	< 0.0001	down
XM_043763401.1	LOC122591181	callose synthase 2-like, transcript variant X1	0.0014	down
XM_043759911.1	LOC122587748	methylthioribose kinase-like, transcript variant X1	0.0041	down
XM_043769191.1	LOC122596583	protein TIFY 10A-like	< 0.0001	down
XM_043774982.1	LOC122602273	AAA-ATPase ASD, mitochondrial-like	0.0046	down
XM_043782792.1	LOC122609845	aluminum-activated malate transporter 10	0.0011	down
XM_043764454.1	LOC122592264	cysteine-rich receptor-like protein kinase 29	< 0.0001	down
XM_043762359.1	LOC122590022	F-box protein SKIP2-like	0.0047	down
XM_043777877.1	LOC122605013	protein CHUP1, chloroplastic	0.0048	down
XM_043779174.1	LOC122606216	uncharacterized LOC122606216	0.0003	down
XM_043761044.1	LOC122588833	cellulose synthase-like protein D3	0.0025	down
XM_043775515.1	LOC122602903	uncharacterized LOC122602903	< 0.0001	down
XM_043763468.1	LOC122591235	ARF guanine-nucleotide exchange factor GNOM-like, transcript variant X2	< 0.0001	down
XM_043768411.1	LOC122595933	probable carboxylesterase 6	< 0.0001	down
XM_043767907.1	LOC122595532	probable F-box protein At2g36090	< 0.0001	down
XM_043779939.1	LOC122607021	cytochrome P450 81Q32-like	< 0.0001	down
XM_043762566.1	LOC122590235	deoxypodophyllotoxin synthase-like	< 0.0001	down
XM_043759243.1	LOC122587149	probable LRR receptor-like serine/threonine-protein kinase At3g47570	0.0001	down
XM_043780492.1	LOC122607505	probable carboxylesterase 2	0.0002	down
XM_043750239.1	LOC122578307	vacuolar-processing enzyme-like	0.0002	down

Transcript id	GeneID	Gene name/function	P value	result
XM_043757494.1	LOC122585363	disease resistance protein Roq1-like	< 0.0001	down
XM_043779026.1	LOC122606063	ethylene-responsive transcription factor 1B-like	0.0005	down
XM_043783555.1	LOC122610575	potassium transporter 6-like	< 0.0001	down
XM_043759570.1	LOC122587408	transcription factor AS1-like	< 0.0001	down
XM_043760638.1	LOC122588506	probable protein phosphatase 2C 55	0.0008	down
XM_043755408.1	LOC122582974	glutamate receptor 1.3-like	< 0.0001	down
XM_043767513.1	LOC122595182	putative UPF0481 protein At3g02645	< 0.0001	down
XM_043778903.1	LOC122605938	germin-like protein subfamily 3 member 2	< 0.0001	down
XM_043780191.1	LOC122607254	protein SENESCENCE-ASSOCIATED GENE 21, mitochondrial-like	0.0006	down
XM_043772412.1	LOC122599825	xyloglucan 6-xylosyltransferase 2-like	< 0.0001	down
XM_043766399.1	LOC122593933	BURP domain-containing protein 3-like	0.0031	down
XM_043764031.1	LOC122591790	uncharacterized LOC122591790	0.0024	down
XM_043776381.1	LOC122603627	putative HVA22-like protein g, transcript variant X1	< 0.0001	down
XM_043766859.1	LOC122594394	MACPF domain-containing protein NSL1	< 0.0001	down
XM_043771475.1	LOC122599030	cyclin-T1-4-like, transcript variant X2	0.0039	down
XM_043754150.1	LOC122581843	benzyl alcohol O-benzoyltransferase-like	< 0.0001	down
XM_043776294.1	LOC122603562	exocyst complex component EXO70H1-like	< 0.0001	down
XM_043761446.1	LOC122589197	probable cinnamyl alcohol dehydrogenase 6	0.0057	down
XR_006324539.1	LOC122605083	uncharacterized LOC122605083, transcript variant X4	0.0001	down
XM_043763519.1	LOC122591280	glutamate receptor 3.4-like	< 0.0001	down
XM_043767892.1	LOC122595525	protein LNK1	< 0.0001	down
XM_043767648.1	LOC122595307	potassium channel AKT2/3	0.0039	down
XM_043754618.1	LOC122582251	enolase	0.0022	down
XM_043768092.1	LOC122595672	nuclear transcription factor Y subunit B-3-like	0.0053	down
XM_043775411.1	LOC122602813	phenylalanine ammonia-lyase-like	< 0.0001	down
XM_043781292.1	LOC122608205	uncharacterized LOC122608205	0.0006	down
XM_043767988.1	LOC122595592	germin-like protein 9-3	0.0004	down
XM_043764155.1	LOC122591928	G-type lectin S-receptor-like serine/threonine-protein kinase LECRK1	< 0.0001	down
XM_043776036.1	LOC122603360	8-hydroxygeraniol dehydrogenase-like	0.0036	down
XM_043757551.1	LOC122585417	NAC domain-containing protein 87-like	0.0007	down
XM_043768234.1	LOC122595790	uncharacterized LOC122595790, transcript variant X2	0.0005	down
XM_043783284.1	LOC122610290	auxin response factor 18-like, transcript variant X2	0.0005	down
XM_043774887.1	LOC122602162	3-hydroxy-3-methylglutaryl coenzyme A reductase 1-like	0.0006	down
XM_043776542.1	LOC122603750	uncharacterized LOC122603750, transcript variant X1	0.0055	down
XM_043760802.1	LOC122588646	probable adenylate kinase 7, mitochondrial	< 0.0001	down
XM_043779445.1	LOC122606603	UDP-glycosyltransferase 75C1-like	0.0002	down
XM_043776801.1	LOC122603940	chitinase-like protein 2, transcript variant X2	0.0002	down
XM_043770383.1	LOC122597808	peroxidase N1-like	< 0.0001	down
XM_043759054.1	LOC122586998	lysine histidine transporter 1-like	< 0.0001	down
XM_043750131.1	LOC122578221	photosystem II core complex proteins psbY, chloroplastic	0.0009	down
XM_043767175.1	LOC122594874	probable alpha,alpha-trehalose-phosphate synthase [UDP-forming] 7	0.0012	down
XM_043773860.1	LOC122601086	probable CoA ligase CCL5	< 0.0001	down
XM_043762219.1	LOC122589889	uncharacterized LOC122589889	0.0013	down
XM_043777860.1	LOC122604999	protein FREE1-like	0.0047	down
XM_043779951.1	LOC122607032	probable glycerol-3-phosphate acyltransferase 3	< 0.0001	down
XM_043766070.1	LOC122593641	transcription initiation factor TFIID subunit 11-like	< 0.0001	down
XM_043784039.1	LOC122611077	polyubiquitin 11-like	< 0.0001	down
XM_043777921.1	LOC122605048	thaumatin-like protein 1b	0.0026	down
XM_043783651.1	LOC122610682	3-beta-hydroxylase-like	0.0029	down
XM_043768436.1	LOC122595950	probable protein phosphatase 2C 59, transcript variant X3	< 0.0001	down
XM_043782121.1	LOC122609059	probable calcium-binding protein CML31	0.0025	down
XM_043767361.1	LOC122595068	protein SUPPRESSOR OF MAX2 1	< 0.0001	down
XM_043760564.1	LOC122588447	uncharacterized LOC122588447	0.0002	down
XR_006322008.1	LOC122586472	uncharacterized LOC122586472	< 0.0001	down
XM_043773922.1	LOC122601153	receptor-like protein kinase FERONIA	0.0006	down
XM_043758598.1	LOC122586620	pentatricopeptide repeat-containing protein At5g01110, transcript variant X3	0.0020	down
XM_043772535.1	LOC122599919	protein LNK1-like	< 0.0001	down
XM_043779421.1	LOC122606581	hyoscyamine 6-dioxygenase-like	0.0008	down
XM_043779503.1	LOC122606664	receptor-like protein kinase FERONIA	< 0.0001	down
XM_043767456.1	LOC122595140	ferredoxin-like	0.0017	down
XR_006324666.1	LOC122605649	uncharacterized LOC122605649	0.0006	down
XM_043770729.1	LOC122598126	auxin response factor 18-like	< 0.0001	down
XM_043776221.1	LOC122603503	uncharacterized LOC122603503, transcript variant X2	0.0001	down
XM_043782427.1	LOC122609380	probable inorganic phosphate transporter 1-7	< 0.0001	down
XM_043750944.1	LOC122578892	probable E3 ubiquitin-protein ligase XERICO	0.0029	down
XM_043770981.1	LOC122598386	lysine histidine transporter-like 8	0.0047	down
XM_043765782.1	LOC122593385	uncharacterized LOC122593385, transcript variant X1	0.0045	down

Transcript id	GeneID	Gene name/function	*P* value	result
XM_043750466.1	LOC122578493	F-box protein AUF1	< 0.0001	down
XM_043750763.1	LOC122578734	uncharacterized LOC122578734, transcript variant X5	0.0044	down
XM_043766935.1	LOC122594489	cold-responsive protein kinase 1-like	0.0057	down
XM_043757808.1	LOC122585686	23 kDa jasmonate-induced protein-like	0.0010	down
XM_043761962.1	LOC122589645	protein NRT1/ PTR FAMILY 4.4	0.0002	down
XM_043749956.1	LOC122578085	DEAD-box ATP-dependent RNA helicase 21-like, transcript variant X2	< 0.0001	down
XM_043774799.1	LOC122602068	G-type lectin S-receptor-like serine/threonine-protein kinase At1g34300	< 0.0001	down
XR_006320611.1	LOC122579212	uncharacterized LOC122579212, transcript variant X6	0.0008	down
XM_043762092.1	LOC122589769	polygalacturonase QRT3-like	0.0003	down
XM_043751978.1	LOC122579749	7-ethoxycoumarin O-deethylase-like	0.0004	down
XM_043768392.1	LOC122595914	metalloendoproteinase 5-MMP-like	0.0017	down
XM_043768100.1	LOC122595679	probable protein phosphatase 2C 63	0.0010	down
XM_043759495.1	LOC122587355	uncharacterized LOC122587355	0.0002	down
XM_043781764.1	LOC122608669	uncharacterized LOC122608669	0.0001	down
XM_043750389.1	LOC122578425	RHOMBOID-like protein 2	< 0.0001	down
XM_043759481.1	LOC122587344	BTB/POZ and TAZ domain-containing protein 4-like, transcript variant X2	0.0005	down
XM_043777023.1	LOC122604122	F-box protein CPR1-like	0.0018	down
XM_043752646.1	LOC122580374	WAT1-related protein At1g43650	< 0.0001	down
XM_043775250.1	LOC122602653	phosphoinositide phospholipase C 6-like	< 0.0001	down
XM_043781078.1	LOC122607998	acyl-coenzyme A thioesterase 13-like	0.0003	down
XM_043775047.1	LOC122602349	protein DMP3-like	0.0006	down
XR_006323120.1	LOC122594836	uncharacterized LOC122594836, transcript variant X2	0.0059	down
XM_043752435.1	LOC122580163	protein EDS1-like	< 0.0001	down
XM_043752333.1	LOC122580062	extensin-3-like	< 0.0001	down
XM_043754615.1	LOC122582248	WAT1-related protein At5g07050	< 0.0001	down
XM_043758823.1	LOC122586828	serine/threonine-protein kinase AFC2, transcript variant X1	< 0.0001	down
XM_043767080.1	LOC122594650	uncharacterized LOC122594650	< 0.0001	down
XM_043770952.1	LOC122598360	CSC1-like protein At3g54510	< 0.0001	down
XM_043767890.1	LOC122595522	protein CELLULOSE SYNTHASE INTERACTIVE 1	< 0.0001	down
XM_043759425.1	LOC122587298	nudix hydrolase 17, mitochondrial-like	0.0037	down
XM_043770305.1	LOC122597737	peroxidase N1-like	< 0.0001	down
XM_043769740.1	LOC122597104	AP2/ERF domain-containing protein PFD0985w-like	0.0027	down
XM_043768615.1	LOC122596097	probable leucine-rich repeat receptor-like protein kinase At1g68400	0.0004	down
XM_043751238.1	LOC122579131	NDR1/HIN1-like protein 6	< 0.0001	down
XM_043784145.1	LOC122611170	glycerate dehydrogenase	< 0.0001	down
XM_043776739.1	LOC122603893	scarecrow-like protein 8	< 0.0001	down
XM_043781455.1	LOC122608360	protein NRT1/ PTR FAMILY 5.2-like	0.0006	down
XM_043778750.1	LOC122605796	S-adenosylmethionine decarboxylase proenzyme-like	0.0033	down
XM_043762332.1	LOC122589999	RING-H2 finger protein ATL8-like	0.0010	down
XM_043779878.1	LOC122606972	protein MICRORCHIDIA 6-like, transcript variant X3	< 0.0001	down
XM_043749941.1	LOC122578071	transcription factor BIM1	0.0015	down
XM_043750713.1	LOC122578694	uncharacterized LOC122578694	< 0.0001	down
XM_043758276.1	LOC122586168	DEAD-box ATP-dependent RNA helicase 42-like	0.0017	down
XM_043755138.1	LOC122582715	probable protein phosphatase 2C 25	0.0019	down
XM_043753709.1	LOC122581479	protein SMG7-like	0.0003	down
XM_043778935.1	LOC122605971	ethylene-responsive transcription factor ERF027-like	< 0.0001	down
XM_043755005.1	LOC122582591	MACPF domain-containing protein At4g24290-like	< 0.0001	down
XM_043750408.1	LOC122578441	uncharacterized LOC122578441, transcript variant X2	0.0001	down
XM_043761370.1	LOC122589125	agamous-like MADS-box protein AGL12	0.0005	down
XM_043767574.1	LOC122595241	C2 domain-containing protein At1g53590-like	< 0.0001	down
XM_043762573.1	LOC122590242	thiol:disulfide interchange protein TxlA homolog	< 0.0001	down
XM_043768201.1	LOC122595765	protein MLN51 homolog, transcript variant X1	0.0015	down
XM_043779728.1	LOC122606851	phospholipase D alpha 1-like	< 0.0001	down
XM_043770972.1	LOC122598379	probable carboxylesterase 7	< 0.0001	down
XM_043762830.1	LOC122590652	calmodulin-binding protein 60 B-like, transcript variant X2	0.0038	down
XM_043784189.1	LOC122611206	glycine cleavage system H protein, mitochondrial	0.0015	down
XM_043759421.1	LOC122587295	palmitoyl-monogalactosyldiacylglycerol delta-7 desaturase, chloroplastic-like	< 0.0001	down
XM_043756900.1	LOC122584836	S-adenosylmethionine decarboxylase proenzyme-like	< 0.0001	down
XM_043753780.1	LOC122581549	methylsterol monooxygenase 2-2, transcript variant X1	0.0007	down
XM_043778014.1	LOC122605135	protein ROOT HAIR DEFECTIVE 3-like	< 0.0001	down
XM_043770468.1	LOC122597886	uncharacterized LOC122597886, transcript variant X3	0.0059	down
XM_043774673.1	LOC122601945	uncharacterized LOC122601945	< 0.0001	down
XM_043759964.1	LOC122587798	U-box domain-containing protein 40	< 0.0001	down
XM_043756404.1	LOC122584092	aquaporin PIP2-1-like	0.0006	down
XM_043778507.1	LOC122605549	uncharacterized LOC122605549, transcript variant X1	0.0020	down
XM_043784037.1	LOC122611075	floral homeotic protein APETALA 2-like, transcript variant X3	0.0013	down

Transcript id	GeneID	Gene name/function	*P* value	result
XM_043779238.1	LOC122606277	protein SIEVE ELEMENT OCCLUSION B-like	0.0008	down
XM_043765822.1	LOC122593413	auxin-responsive protein IAA11-like	0.0004	down
XM_043776045.1	LOC122603367	putative lipid-transfer protein DIR1	< 0.0001	down
XM_043760645.1	LOC122588511	chlorophyllide a oxygenase, chloroplastic-like	0.0018	down
XM_043761886.1	LOC122589581	subtilisin-like protease SBT1.7	< 0.0001	down
XM_043755438.1	LOC122583001	CBL-interacting serine/threonine-protein kinase 7-like	< 0.0001	down
XM_043774160.1	LOC122601397	protein PELPK1-like	0.0012	down
XR_006320697.1	LOC122579755	uncharacterized LOC122579755, transcript variant X7	0.0002	down
XM_043781411.1	LOC122608318	protein NPGR2-like, transcript variant X3	< 0.0001	down
XM_043750704.1	LOC122578686	alcohol dehydrogenase-like 3	< 0.0001	down
XM_043756248.1	LOC122583874	chaperone protein dnaJ 20, chloroplastic-like	< 0.0001	down
XM_043779727.1	LOC122606850	phospholipase D alpha 1	0.0004	down
XM_043773258.1	LOC122600530	L-type lectin-domain containing receptor kinase IX.1-like	0.0008	down
XM_043779341.1	LOC122606487	uncharacterized LOC122606487, transcript variant X4	0.0016	down
XM_043771243.1	LOC122598654	probable E3 ubiquitin-protein ligase LUL3	< 0.0001	down
XM_043775120.1	LOC122602438	uncharacterized LOC122602438, transcript variant X1	0.0008	down
XM_043754969.1	LOC122582562	signal recognition particle receptor FtsY-like	0.0005	down
XM_043761310.1	LOC122589067	protein Dr1 homolog, transcript variant X1	0.0039	down
XM_043753432.1	LOC122581249	protein TRANSPARENT TESTA GLABRA 1	< 0.0001	down
XM_043780550.1	LOC122607554	uncharacterized LOC122607554, transcript variant X1	0.0018	down
XM_043752511.1	LOC122580233	probable methyltransferase TCM_000336	0.0002	down
XM_043757697.1	LOC122585568	repetitive proline-rich cell wall protein-like	< 0.0001	down
XM_043779390.1	LOC122606543	(S)-8-oxocitronellyl enol synthase CYC2	0.0006	down
XM_043763567.1	LOC122591313	beta-amylase 2, chloroplastic	< 0.0001	down
XM_043780451.1	LOC122607474	molybdopterin biosynthesis protein CNX1	0.0037	down
XM_043781714.1	LOC122608620	transcription factor GTE10-like	< 0.0001	down
XM_043754646.1	LOC122582273	uncharacterized LOC122582273	< 0.0001	down
XM_043772035.1	LOC122599507	rust resistance kinase Lr10-like	0.0011	down
XM_043761811.1	LOC122589511	protein TAPETUM DETERMINANT 1-like	< 0.0001	down
XM_043757411.1	LOC122585290	U-box domain-containing protein 21-like	0.0017	down
XM_043767364.1	LOC122595071	ribulose bisphosphate carboxylase small subunit, chloroplastic 2	0.0007	down
XM_043781002.1	LOC122607929	receptor-like protein kinase, transcript variant X2	< 0.0001	down
XM_043773301.1	LOC122600565	uncharacterized LOC122600565	< 0.0001	down
XM_043758654.1	LOC122586672	low affinity sulfate transporter 3-like, transcript variant X2	0.0049	down
XM_043771031.1	LOC122598439	cytochrome P450 72A397-like	0.0005	down
XM_043759354.1	LOC122587244	uncharacterized LOC122587244, transcript variant X6	< 0.0001	down
XM_043765728.1	LOC122593333	WRKY transcription factor WRKY51-like	0.0003	down
XR_006322727.1	LOC122592535	uncharacterized LOC122592535, transcript variant X6	0.0003	down
XM_043760142.1	LOC122587996	amino acid transporter AVT6C-like	0.0001	down
XM_043755346.1	LOC122582916	serine/threonine-protein kinase PCRK1-like	< 0.0001	down
XM_043758092.1	LOC122585977	uncharacterized LOC122585977, transcript variant X2	< 0.0001	down
XM_043767357.1	LOC122595066	protein DETOXIFICATION 24-like, transcript variant X2	< 0.0001	down
XM_043760546.1	LOC122588436	uncharacterized LOC122588436	0.0012	down
XM_043775416.1	LOC122602819	uncharacterized LOC122602819, transcript variant X1	0.0002	down
XM_043763587.1	LOC122591327	ultraviolet-B receptor UVR8	0.0048	down
XM_043753706.1	LOC122581475	NAD(H) kinase 1	< 0.0001	down
XM_043770062.1	LOC122597472	probable carboxylesterase 5	0.0028	down
XM_043755337.1	LOC122582908	adenylate isopentenyltransferase 3, chloroplastic-like	< 0.0001	down
XM_043753604.1	LOC122581377	ACT domain-containing protein ACR8-like	< 0.0001	down
XM_043769164.1	LOC122596558	NDR1/HIN1-like protein 6	< 0.0001	down
XR_006325384.1	LOC122609749	uncharacterized LOC122609749, transcript variant X5	0.0033	down
XR_006321322.1	LOC122583751	uncharacterized LOC122583751, transcript variant X2	< 0.0001	down
XM_043778701.1	LOC122605737	annexin D5-like, transcript variant X1	0.0013	down
XM_043770719.1	LOC122598114	calcium-dependent protein kinase 26-like	0.0001	down
XM_043777868.1	LOC122605006	calcium-dependent protein kinase 14	0.0001	down
XR_006322380.1	LOC122589970	uncharacterized LOC122589970	0.0016	down
XM_043776994.1	LOC122604084	nuclear pore complex protein NUP1, transcript variant X2	0.0030	down
XM_043783797.1	LOC122610838	putative receptor-like protein kinase At4g00960, transcript variant X1	< 0.0001	down
XM_043773426.1	LOC122600674	metacaspase-1-like, transcript variant X1	0.0033	down
XM_043777679.1	LOC122604812	G-type lectin S-receptor-like serine/threonine-protein kinase SD2-5, transcript variant X2	< 0.0001	down
XM_043779905.1	LOC122606989	aldehyde oxidase GLOX	0.0011	down
XM_043767904.1	LOC122595531	calcium homeostasis endoplasmic reticulum protein, transcript variant	< 0.0001	down
XM_043762559.1	LOC122590224	uncharacterized LOC122590224	0.0028	down
XR_006323844.1	LOC122599023	uncharacterized LOC122599023	< 0.0001	down
XM_043758570.1	LOC122586585	mitogen-activated protein kinase kinase kinase 20-like	< 0.0001	down
XM_043784025.1	LOC122611068	RHOMBOID-like protein 2	< 0.0001	down

Transcript id	GeneID	Gene name/function	*P* value	result
XM_043773513.1	LOC122600757	cytochrome b561 and DOMON domain-containing protein At5g35735-like	0.0010	down
XM_043750614.1	LOC122578618	NAC domain-containing protein 83-like	< 0.0001	down
XM_043783735.1	LOC122610777	basic leucine zipper 43-like	0.0003	down
XR_006324430.1	LOC122602678	uncharacterized LOC122602678, transcript variant X1	0.0021	down
XM_043755813.1	LOC122583407	AT-hook motif nuclear-localized protein 23-like	< 0.0001	down
XM_043765877.1	LOC122593464	xyloglucan endotransglucosylase/hydrolase 2-like	0.0052	down
XM_043763242.1	LOC122591053	NADP-dependent malic enzyme-like	< 0.0001	down
XM_043770889.1	LOC122598296	heat stress transcription factor C-1-like	< 0.0001	down
XM_043781742.1	LOC122608651	uncharacterized LOC122608651	0.0011	down
XM_043770895.1	LOC122598304	probable galacturonosyltransferase-like 9	< 0.0001	down
XM_043771347.1	LOC122598859	serine/threonine-protein kinase prpf4B-like, transcript variant X8	0.0015	down
XM_043750826.1	LOC122578788	xyloglucan glycosyltransferase 4-like	0.0003	down
XM_043774665.1	LOC122601934	protein TsetseEP-like	0.0003	down
XM_043757443.1	LOC122585316	E3 ubiquitin-protein ligase RDUF2-like	< 0.0001	down
XM_043769148.1	LOC122596546	transcription factor VOZ1, transcript variant X1	0.0005	down
XM_043750521.1	LOC122578544	galactan beta-1,4-galactosyltransferase GALS3-like	0.0028	down
XM_043771305.1	LOC122598818	probable WRKY transcription factor 53	< 0.0001	down
XM_043769282.1	LOC122596663	ethylene-responsive transcription factor 3-like	0.0042	down
XM_043757261.1	LOC122585152	ruBisCO large subunit-binding protein subunit beta, chloroplastic-like	0.0038	down
XM_043772169.1	LOC122599632	uncharacterized protein At1g76660-like	< 0.0001	down
XR_006321026.1	LOC122581634	uncharacterized LOC122581634, transcript variant X1	0.0015	down
XR_006323765.1	LOC122598873	uncharacterized LOC122598873, transcript variant X1	0.0014	down
XM_043780942.1	LOC122607872	uncharacterized oxidoreductase At4g09670-like	0.0004	down
XM_043775399.1	LOC122602803	cation/H(+) antiporter 20	< 0.0001	down
XM_043769052.1	LOC122596473	calcium-transporting ATPase 10, plasma membrane-type-like, transcript variant X2	0.0009	down
XM_043753847.1	LOC122581604	formin-like protein 1	0.0046	down
XM_043752981.1	LOC122580820	ferritin-3, chloroplastic-like	< 0.0001	down
XR_006323351.1	LOC122596633	uncharacterized LOC122596633, transcript variant X2	< 0.0001	down
XM_043773140.1	LOC122600429	endoplasmic reticulum oxidoreductin-1-like	< 0.0001	down
XM_043761069.1	LOC122588853	triacylglycerol lipase OBL1-like	< 0.0001	down
XM_043777097.1	LOC122604202	uncharacterized LOC122604202	< 0.0001	down
XM_043779710.1	LOC122606835	E3 ubiquitin-protein ligase SIS3-like, transcript variant X3	0.0054	down
XM_043753268.1	LOC122581117	carbon catabolite repressor protein 4 homolog 6, transcript variant X1	0.0015	down
XM_043762506.1	LOC122590173	UDP-glycosyltransferase 85C2-like	0.0005	down
XM_043750774.1	LOC122578747	phosphoglycerate mutase-like protein AT74H	< 0.0001	down
XM_043760603.1	LOC122588476	GATA transcription factor 8-like, transcript variant X1	< 0.0001	down
XM_043750706.1	LOC122578684	GDSL esterase/lipase At4g10955-like	0.0012	down
XM_043757250.1	LOC122585145	uncharacterized LOC122585145	0.0002	down
XM_043754831.1	LOC122582442	clavaminate synthase-like protein At3g21360	< 0.0001	down
XM_043773111.1	LOC122600401	SNF1-related protein kinase regulatory subunit beta-2-like	< 0.0001	down
XM_043776577.1	LOC122603776	xyloglucan galactosyltransferase XLT2	< 0.0001	down
XM_043755804.1	LOC122583395	carnosic acid synthase-like	0.0013	down
XM_043775154.1	LOC122602542	probable LRR receptor-like serine/threonine-protein kinase At5g63710, transcript variant X1	< 0.0001	down
XM_043756145.1	LOC122583773	probable LRR receptor-like serine/threonine-protein kinase At3g47570	0.0011	down
XM_043776503.1	LOC122603718	uncharacterized LOC122603718	0.0022	down
XR_006325374.1	LOC122609722	uncharacterized LOC122609722, transcript variant X3	0.0059	down
XM_043753312.1	LOC122581145	lysine histidine transporter-like 8	< 0.0001	down
XM_043770644.1	LOC122598040	lysine histidine transporter-like 6	< 0.0001	down
XM_043778731.1	LOC122605775	putative lipid-transfer protein DIR1	< 0.0001	down
XM_043758037.1	LOC122585920	protein ALWAYS EARLY 3, transcript variant X1	< 0.0001	down
XM_043766931.1	LOC122594484	U-box domain-containing protein 13-like	< 0.0001	down
XM_043761104.1	LOC122588884	aspartyl protease family protein At5g10770-like	< 0.0001	down
XM_043775293.1	LOC122602702	uncharacterized LOC122602702, transcript variant X1	< 0.0001	down
XM_043761556.1	LOC122589282	uncharacterized LOC122589282, transcript variant X2	0.0038	down
XM_043763324.1	LOC122591113	beta-amylase 7, transcript variant X2	< 0.0001	down
XM_043776904.1	LOC122604020	type I inositol polyphosphate 5-phosphatase 10	0.0019	down
XR_006322100.1	LOC122587043	uncharacterized LOC122587043	0.0013	down
XM_043760607.1	LOC122588480	amino acid transporter AVT6A-like	< 0.0001	down
XR_006321955.1	LOC122586330	uncharacterized LOC122586330	0.0026	down
XM_043761179.1	LOC122588958	probable inactive receptor kinase At5g58300, transcript variant X2	0.0032	down
XM_043783652.1	LOC122610683	putative disease resistance RPP13-like protein 1	0.0017	down
XR_006320814.1	LOC122580529	uncharacterized LOC122580529	< 0.0001	down
XM_043758828.1	LOC122586831	low affinity sulfate transporter 3-like, transcript variant X1	< 0.0001	down
XR_006324602.1	LOC122605362	zinc finger CCCH domain-containing protein 41, transcript variant X3	0.0016	down
XM_043753737.1	LOC122581506	putative disease resistance protein At3g14460	0.0030	down

Transcript id	GeneID	Gene name/function	P value	result
XM_043782878.1	LOC122609931	type I inositol polyphosphate 5-phosphatase 2, transcript variant X1	0.0002	down
XM_043772526.1	LOC122599914	uncharacterized LOC122599914, transcript variant X1	< 0.0001	down
XM_043776694.1	LOC122603861	wound-induced protein 1	< 0.0001	down
XM_043760354.1	LOC122588232	vegetative cell wall protein gp1-like	< 0.0001	down
XM_043780084.1	LOC122607159	GEM-like protein 1	< 0.0001	down
XM_043778816.1	LOC122605859	serine/threonine-protein kinase UCN-like	< 0.0001	down
XR_006321839.1	LOC122585969	uncharacterized LOC122585969	0.0012	down
XM_043755326.1	LOC122582898	zeatin O-xylosyltransferase-like	0.0015	down
XM_043776822.1	LOC122603954	zinc finger CCCH domain-containing protein 29-like	0.0022	down
XM_043778300.1	LOC122605367	uncharacterized LOC122605367	0.0016	down
XM_043771908.1	LOC122599400	probable carotenoid cleavage dioxygenase 4, chloroplastic	< 0.0001	down
XM_043780001.1	LOC122607082	uncharacterized LOC122607082	< 0.0001	down
XM_043781744.1	LOC122608652	protein MIZU-KUSSEI 1-like	0.0047	down
XM_043754547.1	LOC122582187	sucrose synthase 2-like	0.0011	down
XM_043773062.1	LOC122600353	phospholipid-transporting ATPase 3-like, transcript variant X1	< 0.0001	down
XM_043768870.1	LOC122596318	WAT1-related protein At1g21890-like	< 0.0001	down
XM_043756091.1	LOC122583711	WAT1-related protein At2g39510-like	0.0032	down
XM_043778865.1	LOC122605905	ATP-dependent RNA helicase-like protein DB10	< 0.0001	down
XM_043753180.1	LOC122581038	peptidyl-prolyl cis-trans isomerase CYP95, transcript variant X2	0.0017	down
XM_043755673.1	LOC122583248	non-specific lipid transfer protein GPI-anchored 5-like	< 0.0001	down
XM_043753266.1	LOC122581115	zeatin O-glucosyltransferase-like	< 0.0001	down
XM_043776608.1	LOC122603800	benzaldehyde dehydrogenase, mitochondrial-like, transcript variant X2	0.0024	down
XM_043780919.1	LOC122607853	ethylene-responsive transcription factor CRF1-like	0.0002	down
XM_043762701.1	LOC122590500	protein PELPK1-like	< 0.0001	down
XM_043783746.1	LOC122610787	cysteine protease ATG4-like	< 0.0001	down
XM_043769407.1	LOC122596777	probable indole-3-acetic acid-amido synthetase GH3.1	< 0.0001	down
XM_043771376.1	LOC122598893	TNF receptor-associated factor homolog 1a-like, transcript variant X1	0.0001	down
XM_043758137.1	LOC122586022	malonyl-coenzyme A:anthocyanin 3-O-glucoside-6"-O-malonyltransferase-like	< 0.0001	down
XM_043775243.1	LOC122602644	germin-like protein 9-3	0.0001	down
XM_043779155.1	LOC122606195	universal stress protein PHOS32	0.0056	down
XM_043751530.1	LOC122579365	aspartyl protease family protein 2-like	0.0004	down
XM_043757480.1	LOC122585353	TMV resistance protein N-like	0.0002	down
XM_043765701.1	LOC122593308	zinc finger protein ZAT10-like	0.0001	down
XM_043782052.1	LOC122608987	receptor-like protein kinase ANXUR1	0.0049	down
XM_043783411.1	LOC122610418	23 kDa jasmonate-induced protein-like	< 0.0001	down
XM_043772310.1	LOC122599749	putative gamma-glutamylcyclotransferase At3g02910	0.0009	down
XM_043776892.1	LOC122604010	histidine kinase 1-like, transcript variant X2	0.0001	down
XM_043752320.1	LOC122580047	uncharacterized LOC122580047, transcript variant X1	< 0.0001	down
XM_043783439.1	LOC122610448	uncharacterized LOC122610448	0.0026	down
XM_043755924.1	LOC122583530	putative disease resistance RPP13-like protein 1	< 0.0001	down
XM_043781567.1	LOC122608460	transcription factor DIVARICATA-like	0.0055	down
XM_043761131.1	LOC122588913	probable fructokinase-5	0.0005	down
XM_043783963.1	LOC122611012	uncharacterized LOC122611012	0.0006	down
XM_043778436.1	LOC122605481	chaperone protein dnaJ 20, chloroplastic-like	0.0013	down
XM_043757811.1	LOC122585689	heavy metal-associated isoprenylated plant protein 20-like	< 0.0001	down
XM_043759159.1	LOC122587085	monocopper oxidase-like protein SKS1	< 0.0001	down
XM_043784312.1	LOC122611304	probable galactinol--sucrose galactosyltransferase 1, transcript variant	0.0048	down
XM_043778503.1	LOC122605546	uncharacterized LOC122605546	0.0050	down
XM_043773947.1	LOC122601180	putative phospholipid-transporting ATPase 9	< 0.0001	down
XM_043768786.1	LOC122596239	uncharacterized LOC122596239	0.0009	down
XM_043750014.1	LOC122578126	fructose-bisphosphate aldolase 1, chloroplastic-like	0.0002	down
XM_043779661.1	LOC122606806	mitogen-activated protein kinase homolog NTF6-like	< 0.0001	down
XM_043775389.1	LOC122602792	squalene epoxidase 3-like, transcript variant X2	0.0006	down
XM_043761398.1	LOC122589152	glucosamine inositolphosphorylceramide transferase 1-like	0.0009	down
XM_043750549.1	LOC122578566	probable protein phosphatase 2C 10, transcript variant X1	< 0.0001	down
XM_043762587.1	LOC122590256	ethylene-responsive transcription factor ERF019	0.0006	down
XM_043758107.1	LOC122585991	probable LRR receptor-like serine/threonine-protein kinase At1g56130	0.0027	down
XM_043752413.1	LOC122580140	magnesium transporter MRS2-1-like, transcript variant X1	0.0019	down
XM_043763025.1	LOC122590849	mitogen-activated protein kinase kinase kinase 18-like	< 0.0001	down
XM_043783835.1	LOC122610878	uncharacterized LOC122610878	0.0003	down
XM_043759136.1	LOC122587064	IAA-amino acid hydrolase ILR1-like 6	< 0.0001	down
XM_043766624.1	LOC122594152	uncharacterized LOC122594152	< 0.0001	down
XM_043767563.1	LOC122595227	3-ketoacyl-CoA synthase 10	0.0011	down
XM_043756150.1	LOC122583747	ACT domain-containing protein ACR10-like	< 0.0001	down
XM_043750374.1	LOC122578418	F-box/kelch-repeat protein At5g15710-like, transcript variant X3	0.0017	down
XM_043770971.1	LOC122598378	probable carboxylesterase 7	0.0007	down
XM_043758734.1	LOC122586744	cyclic dof factor 1-like	0.0013	down

Transcript id	GeneID	Gene name/function	*P* value	result
XM_043781587.1	LOC122608483	RING-H2 finger protein ATL3-like, transcript variant X2	0.0051	down
XM_043752379.1	LOC122580105	adagio protein 3	0.0002	down
XM_043766039.1	LOC122593613	uncharacterized LOC122593613	0.0030	down
XM_043776480.1	LOC122603705	patatin-like protein 6, transcript variant X3	< 0.0001	down
XM_043753414.1	LOC122581237	syntaxin-121-like	0.0049	down
XR_006322923.1	LOC122594075	uncharacterized LOC122594075	0.0004	down
XM_043763309.1	LOC122591104	UDP-glucuronic acid decarboxylase 2-like	0.0003	down
XR_006324174.1	LOC122602182	uncharacterized LOC122602182	0.0006	down
XM_043780201.1	LOC122607265	adenylate isopentenyltransferase 3, chloroplastic	< 0.0001	down
XM_043765192.1	LOC122592873	ethylene-responsive transcription factor 4-like	< 0.0001	down
XR_006322884.1	LOC122593829	uncharacterized LOC122593829, transcript variant X2	< 0.0001	down
XM_043770619.1	LOC122598015	cucumber peeling cupredoxin-like	0.0004	down
XM_043751982.1	LOC122579753	thioredoxin H-type-like	0.0002	down
XM_043758386.1	LOC122586391	dehydration-responsive element-binding protein 1D-like	< 0.0001	down
XM_043762291.1	LOC122589962	exocyst complex component EXO70B1	< 0.0001	down
XM_043758085.1	LOC122585971	proline-rich receptor-like protein kinase PERK1	< 0.0001	down
XM_043777622.1	LOC122604750	S-adenosylmethionine synthase 3-like	0.0004	down
XM_043767215.1	LOC122594930	calmodulin-binding transcription activator 4-like, transcript variant X1	0.0002	down
XM_043779737.1	LOC122606857	respiratory burst oxidase homolog protein C-like	0.0036	down
XM_043772936.1	LOC122600249	AAA-ATPase ASD, mitochondrial-like	< 0.0001	down
XM_043758585.1	LOC122586607	protein COBRA-like	0.0024	down
XM_043755186.1	LOC122582756	remorin 4.1-like	< 0.0001	down
XM_043754230.1	LOC122581910	receptor-like protein kinase FERONIA	0.0003	down
XM_043759405.1	LOC122587283	putative UPF0481 protein At3g02645	< 0.0001	down
XM_043778099.1	LOC122605198	AAA-ATPase At3g50940-like	< 0.0001	down
XM_043767469.1	LOC122595149	probable galacturonosyltransferase 4, transcript variant X1	0.0003	down
XM_043780826.1	LOC122607783	uncharacterized LOC122607783, transcript variant X2	< 0.0001	down
XM_043773257.1	LOC122600529	FCS-Like Zinc finger 1-like	< 0.0001	down
XM_043771328.1	LOC122598843	NF-X1-type zinc finger protein NFXL1, transcript variant X2	0.0006	down
XM_043763593.1	LOC122591333	delta(8)-fatty-acid desaturase-like	< 0.0001	down
XM_043782418.1	LOC122609369	wall-associated receptor kinase-like 9	0.0030	down
XM_043757168.1	LOC122585070	(-)-beta-pinene synthase, chloroplastic-like	0.0031	down
XM_043771957.1	LOC122599436	phosphatidylinositol 4-phosphate 5-kinase 9-like, transcript variant X1	0.0021	down
XM_043753421.1	LOC122581240	ABC transporter C family member 4-like	< 0.0001	down
XM_043782892.1	LOC122609939	HEAT repeat-containing protein 6, transcript variant X1	0.0012	down
XM_043774658.1	LOC122601928	glutamyl-tRNA reductase 2-like	< 0.0001	down
XM_043750427.1	LOC122578457	putative transferase At4g12130, mitochondrial	< 0.0001	down
XR_006321069.1	LOC122582025	uncharacterized LOC122582025, transcript variant X6	0.0023	down
XR_006323941.1	LOC122599733	uncharacterized LOC122599733	0.0002	down
XM_043775564.1	LOC122602951	uncharacterized LOC122602951	0.0052	down
XM_043763443.1	LOC122591217	probable carboxylesterase 12	0.0022	down
XM_043763568.1	LOC122591314	RNA polymerase II C-terminal domain phosphatase-like 1	< 0.0001	down
XM_043775665.1	LOC122603052	calcium-dependent protein kinase 18-like, transcript variant X2	0.0024	down
XM_043767900.1	LOC122595529	pectinesterase 1-like	< 0.0001	down
XM_043770668.1	LOC122598056	metacaspase-1-like	0.0002	down
XM_043766346.1	LOC122593895	pentatricopeptide repeat-containing protein At1g11710, mitochondrial	0.0008	down
XM_043778907.1	LOC122605941	putative HVA22-like protein g	< 0.0001	down
XM_043764392.1	LOC122592193	glycine-rich cell wall structural protein 1.8-like	0.0008	down
XM_043768533.1	LOC122596034	fasciclin-like arabinogalactan protein 7	0.0046	down
XM_043751671.1	LOC122579489	probable sucrose-phosphate synthase 4	0.0002	down
XM_043776476.1	LOC122603702	ultraviolet-B receptor UVR8-like, transcript variant X2	< 0.0001	down
XM_043755042.1	LOC122582625	probable WRKY transcription factor 26	0.0047	down
XM_043763180.1	LOC122591002	protein NRT1/ PTR FAMILY 6.4-like	0.0020	down
XM_043780003.1	LOC122607084	uncharacterized LOC122607084	0.0015	down
XM_043782354.1	LOC122609302	probable disease resistance protein At4g27220	0.0014	down
XM_043779069.1	LOC122606105	probable endo-1,3(4)-beta-glucanase ARB_01444	< 0.0001	down
XM_043753881.1	LOC122581636	NAD-dependent protein deacylase SRT2-like, transcript variant X1	0.0014	down
XM_043758732.1	LOC122586743	malate dehydrogenase, chloroplastic, transcript variant X1	< 0.0001	down
XR_006320706.1	LOC122579839	uncharacterized LOC122579839	< 0.0001	down
XM_043772311.1	LOC122599751	uncharacterized LOC122599751	0.0005	down
XM_043753719.1	LOC122581489	cyclin-dependent kinase F-4-like	0.0010	down
XM_043770759.1	LOC122598158	disease resistance protein RUN1-like	< 0.0001	down
XM_043782750.1	LOC122609809	putative receptor-like protein kinase At5g39000	0.0012	down
XM_043765317.1	LOC122592986	uncharacterized LOC122592986	< 0.0001	down
XM_043752911.1	LOC122580644	lysine-specific demethylase JMJ706	0.0006	down
XM_043770384.1	LOC122597809	U-box domain-containing protein 26-like	< 0.0001	down
XM_043779891.1	LOC122606978	uncharacterized LOC122606978, transcript variant X1	0.0004	down
XM_043751483.1	LOC122579330	transcription factor TGA3-like	< 0.0001	down

Transcript id	GeneID	Gene name/function	*P* value	result
XM_043767576.1	LOC122595242	putative callose synthase 8	0.0007	down
XR_006322586.1	LOC122590718	uncharacterized LOC122590718	< 0.0001	down
XM_043772727.1	LOC122600072	alpha-1,3-arabinosyltransferase XAT2-like	0.0028	down
XM_043779332.1	LOC122606483	probably inactive leucine-rich repeat receptor-like protein kinase At5g48380	0.0050	down
XM_043768124.1	LOC122595699	artemisinic aldehyde Delta(11(13)) reductase	0.0030	down
XM_043775550.1	LOC122602936	pleiotropic drug resistance protein 2-like, transcript variant X1	< 0.0001	down
XM_043756383.1	LOC122584022	dirigent protein 18-like	< 0.0001	down
XM_043774995.1	LOC122602290	non-specific lipid transfer protein GPI-anchored 20	0.0010	down
XM_043781653.1	LOC122608555	uncharacterized LOC122608555	0.0008	down
XM_043751761.1	LOC122579572	probable protein phosphatase 2C 12	< 0.0001	down
XM_043750947.1	LOC122578898	UDP-glycosyltransferase 72B1-like	0.0001	down
XM_043763251.1	LOC122591058	glycerophosphodiester phosphodiesterase GDPDL3-like	< 0.0001	down
XM_043773864.1	LOC122601092	cold-responsive protein kinase 1-like	0.0046	down
XM_043751476.1	LOC122579323	transcription factor TCP7-like	< 0.0001	down
XM_043770961.1	LOC122598368	probable serine/threonine-protein kinase PBL1	0.0043	down
XM_043779442.1	LOC122606597	lipoxygenase 1, chloroplastic	< 0.0001	down
XM_043759420.1	LOC122587294	rop guanine nucleotide exchange factor 5-like	< 0.0001	down
XM_043781215.1	LOC122608122	calcium-transporting ATPase 12, plasma membrane-type-like	< 0.0001	down
XM_043778640.1	LOC122605686	beta-fructofuranosidase, soluble isoenzyme I-like	< 0.0001	down
XM_043770896.1	LOC122598305	calmodulin-like	0.0002	down
XM_043777441.1	LOC122604557	putative WEB family protein At1g65010, chloroplastic	< 0.0001	down
XM_043780197.1	LOC122607259	uncharacterized LOC122607259	< 0.0001	down
XM_043765388.1	LOC122593046	AAA-ATPase At2g46620	< 0.0001	down
XM_043779185.1	LOC122606223	uncharacterized LOC122606223	0.0024	down
XM_043773952.1	LOC122600898	casparian strip membrane protein 1-like	0.0008	down
XM_043766204.1	LOC122593758	gamma-glutamyl peptidase 3-like	< 0.0001	down
XM_043763198.1	LOC122591013	alpha-glucan phosphorylase, H isozyme	< 0.0001	down
XM_043761440.1	LOC122589189	cell division cycle 20.2, cofactor of APC complex-like	0.0002	down
XR_006322176.1	LOC122588688	uncharacterized LOC122588688	< 0.0001	down
XM_043750911.1	LOC122578863	GEM-like protein 5	< 0.0001	down
XM_043752211.1	LOC122579944	anthocyanidin 3-O-glucosyltransferase 5-like	0.0022	down
XM_043770637.1	LOC122598029	alkaline/neutral invertase A, mitochondrial-like	< 0.0001	down
XM_043750098.1	LOC122578192	pimeloyl-[acyl-carrier protein] methyl ester esterase-like	0.0053	down
XM_043773157.1	LOC122600442	3-ketoacyl-CoA synthase 11-like	0.0013	down
XR_006321854.1	LOC122586035	DEAD-box ATP-dependent RNA helicase 37-like, transcript variant X2	0.0051	down
XM_043763261.1	LOC122591065	COP1-interacting protein 7, transcript variant X1	< 0.0001	down
XM_043770410.1	LOC122597839	transcription factor MYB1R1	< 0.0001	down
XM_043753376.1	LOC122581204	uncharacterized protein At3g49140, transcript variant X1	0.0003	down
XM_043771965.1	LOC122599451	gamma-glutamylcyclotransferase 2-1-like	0.0025	down
XM_043768107.1	LOC122595684	gallate 1-beta-glucosyltransferase 84A23-like	0.0058	down
XM_043759417.1	LOC122587291	F-box protein SKIP2-like	0.0001	down
XM_043762537.1	LOC122590202	granule-bound starch synthase 2, chloroplastic/amyloplastic-like, transcript variant X2	0.0010	down
XM_043752555.1	LOC122580278	F-box protein CPR1-like	0.0027	down
XM_043755871.1	LOC122583471	cell number regulator 7-like	0.0008	down
XM_043759327.1	LOC122587218	uncharacterized LOC122587218, transcript variant X1	< 0.0001	down
XM_043784319.1	LOC122611309	uncharacterized LOC122611309	0.0042	down
XM_043759196.1	LOC122587116	dehydration-responsive element-binding protein 1E-like	< 0.0001	down
XM_043752625.1	LOC122580354	ethylene-responsive transcription factor ERF027-like	< 0.0001	down
XM_043774918.1	LOC122602205	GATA zinc finger domain-containing protein 4-like	0.0005	down
XM_043758511.1	LOC122586524	ethylene-responsive transcription factor 2-like	0.0027	down
XM_043771792.1	LOC122599298	auxin response factor 9, transcript variant X1	< 0.0001	down
XM_043753433.1	LOC122581250	probable disease resistance protein At5g66900, transcript variant X2	0.0020	down
XM_043778415.1	LOC122605461	uncharacterized LOC122605461, transcript variant X1	< 0.0001	down
XM_043767437.1	LOC122595122	B3 domain-containing protein Os06g0194400-like, transcript variant X2	0.0016	down
XM_043762352.1	LOC122590014	uncharacterized LOC122590014	0.0003	down
XM_043783863.1	LOC122610909	leucine-rich repeat extensin-like protein 4	< 0.0001	down
XM_043769428.1	LOC122596789	WAT1-related protein At2g39510-like	< 0.0001	down
XM_043754982.1	LOC122582570	uncharacterized LOC122582570, transcript variant X2	0.0006	down
XM_043759787.1	LOC122587612	cucumisin-like	0.0006	down
XM_043755938.1	LOC122583544	galactolipase DONGLE, chloroplastic-like	0.0056	down
XM_043752733.1	LOC122580462	exocyst complex component EXO70B1-like	< 0.0001	down
XM_043775510.1	LOC122602898	beta-glucosidase BoGH3B-like	0.0031	down
XM_043782842.1	LOC122609891	uncharacterized LOC122609891	0.0007	down
XR_006323456.1	LOC122597765	uncharacterized LOC122597765	0.0028	down
XM_043764258.1	LOC122592046	ethylene-responsive transcription factor ERF017-like	0.0001	down
XM_043761890.1	LOC122589585	U-box domain-containing protein 4-like	< 0.0001	down

Transcript id	GeneID	Gene name/function	P value	result
XM_043775663.1	LOC122603049	uncharacterized LOC122603049	0.0008	down
XM_043778915.1	LOC122605947	wall-associated receptor kinase-like 20	0.0028	down
XM_043763185.1	LOC122591005	UDP-glucuronic acid decarboxylase 5, transcript variant X1	0.0002	down
XM_043754657.1	LOC122582288	universal stress protein YxiE-like	0.0001	down
XM_043760286.1	LOC122588152	uncharacterized LOC122588152	0.0048	down
XM_043750251.1	LOC122578318	uncharacterized LOC122578318, transcript variant X2	0.0033	down
XM_043778071.1	LOC122605176	zinc finger A20 and AN1 domain-containing stress-associated protein 6-like	< 0.0001	down
XM_043778390.1	LOC122605437	early nodulin-20-like	< 0.0001	down
XM_043765705.1	LOC122593312	cold-responsive protein kinase 1	0.0034	down
XM_043764585.1	LOC122592375	probable xyloglucan endotransglucosylase/hydrolase protein 23	0.0056	down
XM_043767244.1	LOC122594960	dammarenediol II synthase-like	0.0028	down
XM_043767272.1	LOC122594994	uncharacterized LOC122594994, transcript variant X1	0.0016	down
XM_043753649.1	LOC122581419	receptor like protein kinase S.2-like	< 0.0001	down
XM_043773189.1	LOC122600468	tubulin beta chain-like	< 0.0001	down
XM_043750870.1	LOC122578824	replication protein A 70 kDa DNA-binding subunit A	< 0.0001	down
XM_043754624.1	LOC122582257	E3 ubiquitin-protein ligase ATL42-like	0.0020	down
XM_043761623.1	LOC122589335	uncharacterized LOC122589335	< 0.0001	down
XM_043784155.1	LOC122611177	probable E3 ubiquitin-protein ligase RZFP34	< 0.0001	down
XM_043759287.1	LOC122587187	probable disease resistance protein At5g66900, transcript variant X1	0.0019	down
XM_043763648.1	LOC122591377	scarecrow-like protein 14	0.0017	down
XM_043764816.1	LOC122592556	mucin-5AC	0.0012	down
XM_043781010.1	LOC122607933	probable serine/threonine-protein kinase PBL10, transcript variant X4	< 0.0001	down
XM_043781173.1	LOC122608087	protein EXORDIUM-like 2	< 0.0001	down
XM_043776835.1	LOC122603965	F-box protein At5g49610-like, transcript variant X2	< 0.0001	down
XM_043756816.1	LOC122584760	heavy metal-associated isoprenylated plant protein 26-like	< 0.0001	down
XM_043754169.1	LOC122581860	ATP-dependent zinc metalloprotease FTSH 8, mitochondrial-like, transcript variant X3	0.0002	down
XM_043776247.1	LOC122603522	nematode resistance protein-like HSPRO2	< 0.0001	down
XM_043771427.1	LOC122598974	putative late blight resistance protein homolog R1C-3, transcript variant X3	0.0033	down
XM_043750442.1	LOC122578470	U-box domain-containing protein 4-like	< 0.0001	down
XM_043774818.1	LOC122602085	protein DETOXIFICATION 51-like	0.0036	down
XM_043760243.1	LOC122588103	serine/threonine-protein kinase STY46-like	0.0007	down
XM_043779893.1	LOC122606979	N-terminal acetyltransferase B complex auxiliary subunit NAA25, transcript variant X1	< 0.0001	down
XM_043765910.1	LOC122593492	putative UPF0481 protein At3g02645	< 0.0001	down
XM_043770704.1	LOC122598098	cyclin-SDS	< 0.0001	down
XM_043775366.1	LOC122602772	scarecrow-like protein 15	< 0.0001	down
XM_043784167.1	LOC122611187	amino acid permease 3-like	0.0002	down
XM_043771893.1	LOC122599387	protein ENHANCED DISEASE RESISTANCE 2-like, transcript variant X2	< 0.0001	down
XM_043754414.1	LOC122582065	polyamine oxidase 1	< 0.0001	down
XM_043753455.1	LOC122581265	phototropin-1	< 0.0001	down
XM_043778135.1	LOC122605227	NDR1/HIN1-like protein 3	0.0005	down
XM_043778699.1	LOC122605743	NDR1/HIN1-like protein 13	0.0029	down
XM_043758571.1	LOC122586587	NDR1/HIN1-like protein 1	< 0.0001	down
XM_043781204.1	LOC122608115	transcription factor TGA4-like, transcript variant X1	< 0.0001	down
XM_043772983.1	LOC122600280	BOI-related E3 ubiquitin-protein ligase 1-like	0.0002	down
XM_043775188.1	LOC122602590	uncharacterized LOC122602590	0.0010	down
XM_043775236.1	LOC122602635	1-aminocyclopropane-1-carboxylate synthase-like	< 0.0001	down
XM_043751877.1	LOC122579662	F-box/kelch-repeat protein At3g06240-like	0.0036	down
XM_043782783.1	LOC122609835	serine--tRNA ligase-like, transcript variant X3	0.0024	down
XM_043781568.1	LOC122608461	protein PHLOEM PROTEIN 2-LIKE A1-like	< 0.0001	down
XM_043765690.1	LOC122593298	WRKY transcription factor 22-like	< 0.0001	down
XM_043764893.1	LOC122592618	uncharacterized LOC122592618	< 0.0001	down
XM_043778000.1	LOC122605119	LRR receptor-like serine/threonine-protein kinase FLS2	0.0001	down
XM_043752782.1	LOC122580506	GDSL esterase/lipase At1g74460	0.0040	down
XM_043782041.1	LOC122608973	double-stranded RNA-binding protein 2	0.0013	down
XM_043765385.1	LOC122593043	pathogenesis-related genes transcriptional activator PTI6-like	< 0.0001	down
XM_043757978.1	LOC122585863	phospholipase A1-Igamma3, chloroplastic	0.0053	down
XM_043752016.1	LOC122579781	probable WRKY transcription factor 27	< 0.0001	down
XM_043759426.1	LOC122587300	exocyst complex component SEC3A-like	0.0008	down
XR_006323317.1	LOC122596391	uncharacterized LOC122596391, transcript variant X2	0.0017	down
XM_043775906.1	LOC122603247	plant intracellular Ras-group-related LRR protein 4-like, transcript variant X1	0.0005	down
XM_043781469.1	LOC122608374	tRNA-uridine aminocarboxypropyltransferase 2, transcript variant X2	0.0033	down
XM_043761820.1	LOC122589521	peroxidase 9-like	0.0004	down
XM_043767813.1	LOC122595451	probable clathrin assembly protein At4g32285	0.0039	down

Transcript id	GeneID	Gene name/function	*P* value	result
XM_043756635.1	LOC122584478	F-box/kelch-repeat protein At3g06240-like	0.0045	down
XM_043769283.1	LOC122596664	B-box zinc finger protein 22-like	< 0.0001	down
XM_043780778.1	LOC122607746	nuclear transcription factor Y subunit A-3-like, transcript variant X2	0.0049	down
XM_043778897.1	LOC122605932	uncharacterized LOC122605932	0.0044	down
XM_043755227.1	LOC122582789	GTP cyclohydrolase 1	< 0.0001	down
XM_043775901.1	LOC122603243	protein trichome birefringence-like 19	0.0012	down
XM_043772166.1	LOC122599628	homeobox-leucine zipper protein HAT4	< 0.0001	down
XM_043756752.1	LOC122584704	MACPF domain-containing protein At1g14780	< 0.0001	down
XM_043768433.1	LOC122595949	beta-glucosidase 47-like, transcript variant X1	< 0.0001	down
XM_043765255.1	LOC122592922	SPX domain-containing membrane protein At4g22990-like	0.0011	down
XM_043762872.1	LOC122590691	uncharacterized LOC122590691	0.0059	down
XM_043753608.1	LOC122581384	CBL-interacting serine/threonine-protein kinase 11	< 0.0001	down
XM_043758885.1	LOC122586872	tubulin beta-1 chain	0.0030	down
XM_043750696.1	LOC122578677	zinc finger CCCH domain-containing protein 18-like, transcript variant X1	0.0055	down
XM_043754207.1	LOC122581892	PRA1 family protein B3-like	< 0.0001	down
XM_043774710.1	LOC122601978	ethylene-responsive transcription factor ERF109-like	< 0.0001	down
XM_043757358.1	LOC122585239	molybdenum cofactor sulfurase	0.0022	down
XM_043756616.1	LOC122584454	zinc finger protein CONSTANS-like	0.0002	down
XM_043782794.1	LOC122609847	butanoate--CoA ligase AAE1-like	0.0036	down
XM_043760417.1	LOC122588303	uncharacterized LOC122588303	0.0010	down
XM_043754945.1	LOC122582537	fasciclin-like arabinogalactan protein 11	< 0.0001	down
XM_043775792.1	LOC122603163	choline-phosphate cytidylyltransferase 2-like	< 0.0001	down
XM_043779572.1	LOC122606714	probable serine/threonine-protein kinase PIX13, transcript variant X1	< 0.0001	down
XM_043758723.1	LOC122586734	MDIS1-interacting receptor like kinase 2-like	0.0024	down
XR_006322708.1	LOC122592408	uncharacterized LOC122592408	0.0053	down
XM_043751798.1	LOC122579601	uncharacterized LOC122579601	< 0.0001	down
XM_043772563.1	LOC122599943	sodium transporter HKT1-like	< 0.0001	down
XM_043784052.1	LOC122611091	thiamine thiazole synthase, chloroplastic-like	0.0009	down
XM_043756361.1	LOC122583990	aluminum-activated malate transporter 13-like	< 0.0001	down
XM_043778993.1	LOC122606023	uncharacterized LOC122606023	0.0038	down
XR_006322656.1	LOC122590996	DEAD-box ATP-dependent RNA helicase 42, transcript variant X1	< 0.0001	down
XM_043754038.1	LOC122581763	malonyl-coenzyme A:anthocyanin 3-O-glucoside-6"-O-malonyltransferase-like	0.0007	down
XM_043758928.1	LOC122586905	uncharacterized LOC122586905	< 0.0001	down
XM_043763459.1	LOC122591228	pleiotropic drug resistance protein 3-like	0.0010	down
XM_043758683.1	LOC122586699	uncharacterized LOC122586699	0.0019	down
XM_043758433.1	LOC122586444	uncharacterized LOC122586444, transcript variant X1	< 0.0001	down
XM_043751635.1	LOC122579451	probable L-type lectin-domain containing receptor kinase S.5	< 0.0001	down
XM_043767260.1	LOC122594977	probable linoleate 9S-lipoxygenase 5	< 0.0001	down
XM_043773453.1	LOC122600699	AAA-ATPase At3g28580-like	< 0.0001	down
XM_043758718.1	LOC122586729	ubiquitin-conjugating enzyme E2 28-like	0.0052	down
XR_006324573.1	LOC122605256	uncharacterized LOC122605256, transcript variant X1	0.0045	down
XM_043757095.1	LOC122585001	receptor-like protein 11	0.0003	down
XM_043752698.1	LOC122580428	epoxide hydrolase A-like, transcript variant X1	0.0007	down
XM_043763539.1	LOC122591297	protein TIFY 10A-like	< 0.0001	down
XM_043755163.1	LOC122582740	lachrymatory-factor synthase	0.0011	down
XM_043764599.1	LOC122592386	scarecrow-like transcription factor PAT1	0.0038	down
XM_043765779.1	LOC122593382	shikimate O-hydroxycinnamoyltransferase-like	< 0.0001	down
XM_043769302.1	LOC122596684	kirola-like	< 0.0001	down
XM_043783372.1	LOC122610377	probable polygalacturonase	< 0.0001	down
XM_043775863.1	LOC122603216	fasciclin-like arabinogalactan protein 16	< 0.0001	down
XM_043769746.1	LOC122597113	phosphoinositide phospholipase C 2-like, transcript variant X1	< 0.0001	down
XM_043776423.1	LOC122603657	probable serine/threonine-protein kinase PIX13, transcript variant X1	< 0.0001	down
XM_043762783.1	LOC122590593	2-oxoglutarate dehydrogenase, mitochondrial-like	0.0023	down
XM_043758802.1	LOC122586812	probable RNA-dependent RNA polymerase 1, transcript variant X2	0.0014	down
XM_043776989.1	LOC122604089	protein phosphatase 2C 37-like	0.0053	down
XM_043760027.1	LOC122587865	mitochondrial uncoupling protein 5-like	0.0020	down
XM_043766810.1	LOC122594336	glutaredoxin-C9-like	0.0022	down
XM_043764361.1	LOC122592156	gibberellin 20-oxidase-like protein	0.0010	down
XM_043769884.1	LOC122597270	inositol-tetrakisphosphate 1-kinase 1-like	0.0002	down
XR_006322179.1	LOC122588724	O-fucosyltransferase 14-like, transcript variant X2	0.0002	down
XM_043769931.1	LOC122597325	F-box/FBD/LRR-repeat protein At1g13570-like	0.0002	down
XM_043762397.1	LOC122590063	probable receptor-like protein kinase At2g39360	0.0002	down
XM_043768924.1	LOC122596360	glycosyltransferase BC10-like	0.0003	down
XM_043754864.1	LOC122582470	CBL-interacting protein kinase 2-like	< 0.0001	down
XM_043767650.1	LOC122595305	L-type lectin-domain containing receptor kinase VIII.2-like	0.0016	down
XM_043762601.1	LOC122590270	clavaminate synthase-like protein At3g21360, transcript variant X1	0.0025	down

Transcript id	GeneID	Gene name/function	*P* value	result
XM_043774043.1	LOC122601274	protein NLP7-like, transcript variant X1	0.0004	down
XM_043776258.1	LOC122603532	protein NRT1/ PTR FAMILY 5.8	0.0022	down
XM_043770677.1	LOC122598062	protein NLP4-like, transcript variant X2	0.0030	down
XM_043750975.1	LOC122578924	protein NEN1-like	< 0.0001	down
XM_043779451.1	LOC122606607	probable methyltransferase PMT24	< 0.0001	down
XM_043769416.1	LOC122596784	protein LSD1-like, transcript variant X1	0.0039	down
XM_043773510.1	LOC122600753	uncharacterized LOC122600753	0.0013	down
XR_006322223.1	LOC122589066	uncharacterized LOC122589066	< 0.0001	down
XM_043775073.1	LOC122602383	pelargonidin 3-O-(6-caffeoylglucoside) 5-O-(6-O-malonylglucoside) 4'''-malonyltransferase-like	0.0010	down
XM_043766682.1	LOC122594211	uncharacterized LOC122594211	0.0036	down
XM_043767286.1	LOC122595008	protein MEI2-like 4, transcript variant X3	0.0012	down
XM_043766874.1	LOC122594413	uncharacterized LOC122594413	0.0057	down
XM_043759605.1	LOC122587437	homeobox-leucine zipper protein ATHB-15, transcript variant X1	0.0009	down
XM_043760168.1	LOC122588021	ferric reduction oxidase 2-like	0.0017	down
XM_043782508.1	LOC122609462	protein NIM1-INTERACTING 1-like	0.0005	down
XM_043767610.1	LOC122595271	transcription factor bHLH35-like	< 0.0001	down
XM_043768244.1	LOC122595798	eukaryotic translation initiation factor-like, transcript variant X1	0.0005	down
XM_043776961.1	LOC122604061	uncharacterized LOC122604061	< 0.0001	down
XM_043782951.1	LOC122609986	2-alkenal reductase (NADP(+)-dependent)-like	0.0050	down
XM_043768276.1	LOC122595825	probable disease resistance protein At4g27220, transcript variant X1	< 0.0001	down
XM_043758381.1	LOC122586387	uncharacterized methyltransferase At1g78140, chloroplastic-like, transcript variant X2	0.0044	down
XM_043781941.1	LOC122608864	probable protein phosphatase 2C 49	< 0.0001	down
XM_043781265.1	LOC122608175	NAC domain-containing protein 83-like	< 0.0001	down
XM_043757440.1	LOC122585312	capsanthin/capsorubin synthase, chromoplastic-like	0.0022	down
XM_043750328.1	LOC122578380	transcription factor bHLH162-like	< 0.0001	down
XM_043759158.1	LOC122587083	uncharacterized LOC122587083	0.0026	down
XM_043778976.1	LOC122606006	uncharacterized LOC122606006	< 0.0001	down
XM_043754443.1	LOC122582090	scarecrow-like protein 9	0.0004	down
XM_043751131.1	LOC122579043	protein SRG1-like	< 0.0001	down
XM_043779653.1	LOC122606797	cytosolic sulfotransferase 15-like	0.0008	down
XM_043770004.1	LOC122597404	ABC transporter E family member 2-like	0.0034	down
XM_043778790.1	LOC122605835	putative F-box/kelch-repeat protein At1g12170	< 0.0001	down
XM_043772810.1	LOC122600143	alpha,alpha-trehalose-phosphate synthase [UDP-forming] 1, transcript variant X1	0.0030	down
XM_043772350.1	LOC122599777	probable pectin methylesterase CGR3, transcript variant X1	< 0.0001	down
XM_043773286.1	LOC122600552	inositol transporter 4-like, transcript variant X2	0.0018	down
XM_043757072.1	LOC122584978	uncharacterized LOC122584978	< 0.0001	down
XM_043783287.1	LOC122610289	IAA-amino acid hydrolase ILR1-like 4	0.0049	down
XM_043765150.1	LOC122592832	60S ribosomal protein L3-like	0.0005	down
XM_043780285.1	LOC122607330	bidirectional sugar transporter SWEET9-like	0.0027	down
XM_043754238.1	LOC122581922	glycine-rich protein A3-like	0.0004	down
XM_043782057.1	LOC122608992	dehydrin ERD14-like	0.0008	down
XM_043766720.1	LOC122594250	heat stress transcription factor A-2b-like, transcript variant X1	0.0017	down
XM_043760680.1	LOC122588547	serine/threonine-protein kinase STY13-like	0.0018	down
XM_043769890.1	LOC122597276	FBD-associated F-box protein At5g56370-like	0.0009	down
XM_043773713.1	LOC122600934	cucumber peeling cupredoxin-like	< 0.0001	down
XM_043772633.1	LOC122600003	patatin-like protein 2	0.0036	down
XM_043754663.1	LOC122582292	LRR receptor-like serine/threonine-protein kinase ERL1	0.0027	down
XM_043777195.1	LOC122604304	transcription factor bHLH62-like, transcript variant X1	0.0004	down
XM_043750567.1	LOC122578583	uncharacterized LOC122578583	0.0020	down
XM_043752559.1	LOC122580282	senescence/dehydration-associated protein At3g51250-like	< 0.0001	down
XM_043752402.1	LOC122580132	cytochrome P450 81Q32-like	0.0015	down
XM_043755297.1	LOC122582863	uncharacterized LOC122582863	< 0.0001	down
XM_043771769.1	LOC122599281	ubiquitin-conjugating enzyme E2 28-like, transcript variant X2	< 0.0001	down
XM_043766757.1	LOC122594285	ABC transporter B family member 6-like	0.0008	down
XM_043759142.1	LOC122587069	dehydration-responsive element-binding protein 1E-like	< 0.0001	down
XM_043774356.1	LOC122601604	homeobox-leucine zipper protein ANTHOCYANINLESS 2	0.0014	down
XR_006324754.1	LOC122605918	uncharacterized LOC122605918, transcript variant X2	0.0043	down
XM_043777075.1	LOC122604178	putative F-box protein At1g47765	< 0.0001	down
XM_043783006.1	LOC122610033	auxin response factor 8-like, transcript variant X2	0.0002	down
XM_043761315.1	LOC122589070	histidine-containing phosphotransfer protein 4, transcript variant X3	0.0004	down
XM_043760194.1	LOC122588050	probable pectinesterase/pectinesterase inhibitor 59	0.0038	down
XM_043766954.1	LOC122594507	glycine-rich protein A3-like	< 0.0001	down
XM_043760878.1	LOC122588708	LRR receptor-like serine/threonine-protein kinase ERL1	< 0.0001	down
XR_006325236.1	LOC122608513	uncharacterized LOC122608513	< 0.0001	down
XM_043773147.1	LOC122600437	4-coumarate--CoA ligase-like 6	0.0035	down

Transcript id	GeneID	Gene name/function	*P* value	result
XM_043769730.1	LOC122597094	probable inactive poly [ADP-ribose] polymerase SRO2	< 0.0001	down
XM_043768073.1	LOC122595659	cell division control protein 2 homolog, transcript variant X1	0.0046	down
XM_043762454.1	LOC122590118	ADP-ribosylation factor-like, transcript variant X2	< 0.0001	down
XM_043763534.1	LOC122591292	thiamine thiazole synthase, chloroplastic	< 0.0001	down
XM_043761651.1	LOC122589364	hippocampus abundant transcript-like protein 1	0.0053	down
XM_043751720.1	LOC122579535	uncharacterized protein At5g23160	0.0032	down
XR_006321197.1	LOC122582544	uncharacterized LOC122582544	< 0.0001	down
XM_043756209.1	LOC122583829	E3 ubiquitin-protein ligase PUB22-like	< 0.0001	down
XM_043776290.1	LOC122603560	probable glycosyltransferase At5g03795, transcript variant X1	0.0002	down
XM_043773482.1	LOC122600728	protein SRC2 homolog	0.0010	down
XM_043772765.1	LOC122600105	U-box domain-containing protein 44-like	0.0057	down
XM_043754048.1	LOC122581772	B-box zinc finger protein 24-like	< 0.0001	down
XM_043766967.1	LOC122594521	vacuolar iron transporter homolog 4-like	< 0.0001	down
XM_043780096.1	LOC122607167	purine permease 1-like	0.0056	down
XM_043783493.1	LOC122610508	putative disease resistance RPP13-like protein 1	0.0040	down
XM_043777974.1	LOC122605098	protein ROOT HAIR DEFECTIVE 3-like	0.0003	down
XM_043776072.1	LOC122603387	probable E3 ubiquitin-protein ligase RHC2A	0.0013	down
XM_043771859.1	LOC122599355	disease resistance protein RPV1-like, transcript variant X9	< 0.0001	down
XM_043783811.1	LOC122610852	ethylene-responsive transcription factor CRF2-like	< 0.0001	down
XM_043761433.1	LOC122589183	protein NUCLEAR FUSION DEFECTIVE 4-like	< 0.0001	down
XM_043768903.1	LOC122596340	vacuolar iron transporter 1	< 0.0001	down
XM_043757913.1	LOC122585789	E3 ubiquitin-protein ligase PUB23-like	< 0.0001	down
XM_043765007.1	LOC122592717	zinc finger protein CONSTANS-LIKE 5-like	< 0.0001	down
XR_006320795.1	LOC122580451	uncharacterized LOC122580451	0.0002	down
XM_043755551.1	LOC122583117	uncharacterized LOC122583117	< 0.0001	down
XR_006325364.1	LOC122609669	probable carboxylesterase 11, transcript variant X2	0.0018	down
XM_043783306.1	LOC122610310	GATA transcription factor 8-like	< 0.0001	down
XM_043781775.1	LOC122608684	probable WRKY transcription factor 70	0.0049	down
XM_043765665.1	LOC122593276	cytochrome P450 710A11-like	< 0.0001	down
XM_043771923.1	LOC122599415	cyclin-dependent kinase F-4-like, transcript variant X2	0.0003	down
XM_043774205.1	LOC122601445	ACD11 homolog protein-like	0.0020	down
XM_043764764.1	LOC122592515	protein HEAT-STRESS-ASSOCIATED 32	< 0.0001	down
XM_043752410.1	LOC122580139	ELMO domain-containing protein A, transcript variant X2	< 0.0001	down
XM_043763116.1	LOC122590941	187-kDa microtubule-associated protein AIR9, transcript variant X2	< 0.0001	down
XM_043767652.1	LOC122595310	shikimate O-hydroxycinnamoyltransferase-like	0.0024	down
XM_043765081.1	LOC122592772	cytochrome b561 and DOMON domain-containing protein At5g35735-like	< 0.0001	down
XM_043781314.1	LOC122608226	F-box protein CPR1-like, transcript variant X1	0.0028	down
XM_043750722.1	LOC122578704	transcription factor TCP15-like	< 0.0001	down
XM_043757544.1	LOC122585410	ABC transporter G family member 6-like	0.0012	down
XM_043755716.1	LOC122583290	probable WRKY transcription factor 57, transcript variant X2	0.0002	down
XM_043754639.1	LOC122582268	unknown protein 1	< 0.0001	down
XM_043762857.1	LOC122590676	ABC transporter C family member 3-like, transcript variant X7	< 0.0001	down
XM_043780002.1	LOC122607083	uncharacterized LOC122607083	0.0043	down
XR_006324057.1	LOC122600499	E3 SUMO-protein ligase SIZ1, transcript variant X3	0.0005	down
XM_043759280.1	LOC122587183	lysine--tRNA ligase-like, transcript variant X3	< 0.0001	down
XM_043776191.1	LOC122603479	glutathione synthetase, chloroplastic-like, transcript variant X1	< 0.0001	down
XM_043750490.1	LOC122578515	transcription factor MYB10-like	0.0015	down
XM_043771085.1	LOC122598491	uncharacterized LOC122598491	0.0003	down
XM_043778046.1	LOC122605156	ethylene-responsive transcription factor ERF023-like	< 0.0001	down
XM_043770459.1	LOC122597881	WRKY transcription factor WRKY51-like	< 0.0001	down
XM_043762199.1	LOC122589872	allene oxide cyclase, chloroplastic-like	< 0.0001	down
XM_043765747.1	LOC122593354	auxin response factor 1-like, transcript variant X2	0.0006	down
XM_043756891.1	LOC122584827	transcription factor ILR3-like	< 0.0001	down
XM_043779523.1	LOC122606679	long chain acyl-CoA synthetase 1-like	0.0030	down
XM_043769033.1	LOC122596451	golgin subfamily A member 6-like protein 22	0.0030	down
XM_043756863.1	LOC122584799	probable E3 ubiquitin-protein ligase RHA4A	0.0012	down
XM_043782991.1	LOC122610019	protein TOPLESS-like	< 0.0001	down
XM_043766226.1	LOC122593777	protein TIFY 5A	< 0.0001	down
XM_043764902.1	LOC122592622	adenylate isopentenyltransferase 3, chloroplastic-like	0.0010	down
XR_006325194.1	LOC122608261	uncharacterized LOC122608261	0.0002	down
XM_043759659.1	LOC122587474	NDR1/HIN1-like protein 13	< 0.0001	down
XM_043779262.1	LOC122606304	subtilisin-like protease SBT1.3	< 0.0001	down
XM_043776991.1	LOC122604091	probable disease resistance protein At4g27220	< 0.0001	down
XM_043775014.1	LOC122602314	protein PELPK1-like	0.0016	down
XM_043769909.1	LOC122597297	cytochrome P450 CYP749A22-like	0.0003	down
XM_043754975.1	LOC122582566	integrator complex subunit 9, transcript variant X3	0.0039	down
XM_043758562.1	LOC122586578	receptor-like protein EIX2	0.0023	down

Transcript id	GeneID	Gene name/function	*P* value	result
XM_043750644.1	LOC122578640	transmembrane protein 53	< 0.0001	down
XM_043778711.1	LOC122605756	mitogen-activated protein kinase kinase kinase 20-like	0.0036	down
XM_043756520.1	LOC122584307	sarcoplasmic reticulum histidine-rich calcium-binding protein, transcript variant X2	< 0.0001	down
XM_043752745.1	LOC122580474	uncharacterized LOC122580474	< 0.0001	down
XM_043763250.1	LOC122591057	zinc finger CCCH domain-containing protein 20	< 0.0001	down
XM_043763505.1	LOC122591267	phospholipase A1 PLIP2, chloroplastic-like	< 0.0001	down
XM_043776964.1	LOC122604063	NDR1/HIN1-like protein 3	0.0041	down
XM_043776226.1	LOC122603492	zinc finger protein CONSTANS-LIKE 15-like, transcript variant X1	< 0.0001	down
XM_043756371.1	LOC122584003	endoribonuclease Dicer homolog 1	0.0002	down
XM_043768273.1	LOC122595823	DELLA protein GAI-like	< 0.0001	down
XM_043778232.1	LOC122605313	protein ESKIMO 1	0.0029	down
XM_043753207.1	LOC122581066	CBL-interacting protein kinase 18-like, transcript variant X1	0.0021	down
XM_043772746.1	LOC122600086	splicing factor 3B subunit 1, transcript variant X1	< 0.0001	down
XM_043771469.1	LOC122599022	ethylene-responsive transcription factor ERF017-like	< 0.0001	down
XM_043763085.1	LOC122590909	protein SPA1-RELATED 4-like, transcript variant X1	< 0.0001	down
XM_043763398.1	LOC122591179	non-specific lipid transfer protein GPI-anchored 5-like	0.0032	down
XM_043780967.1	LOC122607895	allene oxide synthase	0.0002	down
XM_043757564.1	LOC122585431	WRKY transcription factor 22-like	< 0.0001	down
XM_043759453.1	LOC122587317	EP1-like glycoprotein 3	< 0.0001	down
XM_043784061.1	LOC122611096	probable trehalose-phosphate phosphatase D	< 0.0001	down
XM_043775912.1	LOC122603252	probable serine/threonine-protein kinase PIX13	< 0.0001	down
XM_043773180.1	LOC122600463	L-type lectin-domain containing receptor kinase IX.1-like	0.0034	down
XM_043780329.1	LOC122607368	dimethylnonatriene synthase-like	< 0.0001	down
XM_043782895.1	LOC122609941	cellulose synthase-like protein D2	< 0.0001	down
XM_043782649.1	LOC122609701	uncharacterized LOC122609701	< 0.0001	down
XM_043781017.1	LOC122607939	protein LIGHT-DEPENDENT SHORT HYPOCOTYLS 5-like	0.0002	down
XM_043770315.1	LOC122597746	zinc finger A20 and AN1 domain-containing stress-associated protein 5-like	0.0002	down
XM_043780692.1	LOC122607673	protein OCTOPUS	0.0010	down
XR_006325455.1	LOC122610309	uncharacterized LOC122610309	0.0021	down
XM_043767551.1	LOC122595214	protein unc-13 homolog	< 0.0001	down
XM_043751759.1	LOC122579560	probable protein phosphatase 2C 35, transcript variant X2	0.0014	down
XM_043779061.1	LOC122606096	uncharacterized LOC122606096	< 0.0001	down
XR_006323437.1	LOC122597473	ABC transporter F family member 4-like, transcript variant X7	< 0.0001	down
XM_043769233.1	LOC122596621	probable 2-oxoglutarate-dependent dioxygenase AOP1	< 0.0001	down
XM_043775982.1	LOC122603308	uncharacterized LOC122603308	0.0009	down
XM_043778983.1	LOC122606012	histidinol dehydrogenase, chloroplastic, transcript variant X3	0.0002	down
XM_043776097.1	LOC122603405	staphylococcal-like nuclease CAN1	< 0.0001	down
XM_043766062.1	LOC122593635	subtilisin-like protease SBT5.6	0.0004	down
XM_043777808.1	LOC122604952	blue copper protein-like	0.0009	down
XM_043762484.1	LOC122590147	uncharacterized LOC122590147	0.0005	down
XM_043756484.1	LOC122584271	pyruvate kinase 2, cytosolic-like	0.0021	down
XM_043761285.1	LOC122589046	uncharacterized LOC122589046	< 0.0001	down
XM_043761304.1	LOC122589061	lysine-rich arabinogalactan protein 17-like	< 0.0001	down
XM_043778203.1	LOC122605294	ethylene-responsive transcription factor CRF4-like	< 0.0001	down
XM_043760797.1	LOC122588641	CRAL-TRIO domain-containing protein YKL091C-like	0.0013	down
XM_043756195.1	LOC122583817	non-specific lipid-transfer protein-like, transcript variant X2	< 0.0001	down
XM_043758003.1	LOC122585887	protein NLP6-like, transcript variant X1	0.0001	down
XM_043777177.1	LOC122604290	wall-associated receptor kinase 5-like	0.0016	down
XM_043755468.1	LOC122583032	cation/H(+) antiporter 20-like, transcript variant X1	0.0003	down
XM_043772831.1	LOC122600161	G patch domain-containing protein 8, transcript variant X1	< 0.0001	down
XM_043766537.1	LOC122594058	E3 ubiquitin-protein ligase RMA1H1-like	0.0003	down
XM_043775787.1	LOC122603158	zinc finger protein CONSTANS-LIKE 2	0.0014	down
XM_043778279.1	LOC122605354	laccase-12-like	< 0.0001	down
XM_043764259.1	LOC122592047	ethylene-responsive transcription factor ERF018-like	< 0.0001	down
XM_043767493.1	LOC122595167	thioredoxin H2-like, transcript variant X1	0.0028	down
XM_043761429.1	LOC122589178	cytochrome P450 736A117-like	0.0006	down
XM_043754656.1	LOC122582287	uncharacterized LOC122582287	0.0010	down
XM_043777458.1	LOC122604575	peroxidase 17	0.0002	down
XM_043761351.1	LOC122589108	uncharacterized LOC122589108	0.0014	down
XM_043749954.1	LOC122578084	DEAD-box ATP-dependent RNA helicase 21-like, transcript variant X2	0.0002	down
XM_043765118.1	LOC122592803	U-box domain-containing protein 26-like	< 0.0001	down
XM_043768752.1	LOC122596212	common plant regulatory factor 1-like, transcript variant X2	< 0.0001	down
XM_043782326.1	LOC122609269	probable protein phosphatase 2C 2	0.0029	down
XM_043759285.1	LOC122587186	caffeic acid 3-O-methyltransferase-like, transcript variant X2	0.0024	down
XM_043765116.1	LOC122592802	nuclear cap-binding protein subunit 1, transcript variant X1	< 0.0001	down

Transcript id	GeneID	Gene name/function	*P* value	result
XM_043756285.1	LOC122583911	polycomb group protein EMBRYONIC FLOWER 2, transcript variant X2	0.0024	down
XM_043774058.1	LOC122601293	early nodulin-like protein 1	< 0.0001	down
XR_006323565.1	LOC122598254	uncharacterized LOC122598254, transcript variant X2	0.0037	down
XM_043766300.1	LOC122593851	vegetative cell wall protein gp1	0.0005	down
XM_043755170.1	LOC122582742	23 kDa jasmonate-induced protein-like	0.0010	down
XM_043779379.1	LOC122606524	E3 ubiquitin-protein ligase HEL2-like	< 0.0001	down
XM_043756399.1	LOC122584042	casparian strip membrane protein 1	< 0.0001	down
XM_043767509.1	LOC122595178	DELLA protein GAI-like	0.0002	down
XM_043749911.1	LOC122578046	probable isoprenylcysteine alpha-carbonyl methylesterase ICMEL2, transcript variant X2	< 0.0001	down
XM_043782460.1	LOC122609416	protein VAPYRIN-LIKE-like	0.0056	down
XM_043781534.1	LOC122608426	UDP-arabinopyranose mutase 1-like	< 0.0001	down
XM_043773992.1	LOC122601231	adenylate isopentenyltransferase 5, chloroplastic	< 0.0001	down
XM_043768783.1	LOC122596236	serine/threonine-protein phosphatase 6 regulatory subunit 3-like	0.0046	down
XM_043771674.1	LOC122599210	CBL-interacting serine/threonine-protein kinase 6	0.0007	down
XM_043753573.1	LOC122581354	cinnamoyl-CoA reductase 1	< 0.0001	down
XM_043754882.1	LOC122582485	scarecrow-like protein 14	< 0.0001	down
XM_043771867.1	LOC122599057	dirigent protein 9-like	< 0.0001	down
XM_043765911.1	LOC122593493	cytochrome P450 78A9-like	< 0.0001	down
XM_043767816.1	LOC122595453	protein LURP-one-related 5	0.0012	down
XM_043762372.1	LOC122590033	centromere-associated protein E, transcript variant X3	< 0.0001	down
XM_043768858.1	LOC122596309	uncharacterized LOC122596309	0.0022	down
XM_043750322.1	LOC122578373	mitochondrial uncoupling protein 5-like	< 0.0001	down
XM_043756875.1	LOC122584813	aquaporin TIP4-1	0.0003	down
XM_043758786.1	LOC122586800	type IV inositol polyphosphate 5-phosphatase 3-like, transcript variant X1	0.0010	down
XM_043777364.1	LOC122604467	protein CNGC15b-like	0.0034	down
XM_043755674.1	LOC122583250	ABC transporter G family member 29-like	0.0009	down
XM_043778713.1	LOC122605758	beta-amyrin 28-monooxygenase-like	0.0001	down
XM_043750711.1	LOC122578692	uncharacterized LOC122578692	< 0.0001	down
XM_043761646.1	LOC122589358	HVA22-like protein e	0.0057	down
XM_043751479.1	LOC122579325	transcription factor bHLH123-like	0.0033	down
XM_043767295.1	LOC122595016	beta-xylosidase/alpha-L-arabinofuranosidase 2-like	0.0009	down
XM_043782847.1	LOC122609896	respiratory burst oxidase homolog protein C-like	0.0002	down
XM_043783479.1	LOC122610494	receptor-like protein kinase 7	0.0007	down
XM_043774671.1	LOC122601943	swi5-dependent recombination DNA repair protein 1 homolog	0.0030	down
XM_043758731.1	LOC122586742	probable xyloglucan 6-xylosyltransferase 5	0.0013	down
XM_043773225.1	LOC122600501	hydroxymethylglutaryl-CoA lyase, mitochondrial-like, transcript variant X1	0.0008	down
XM_043778895.1	LOC122605930	probable receptor-like protein kinase At5g24010, transcript variant X1	< 0.0001	down
XM_043762953.1	LOC122590781	chorion class high-cysteine HCB protein 13-like	0.0009	down
XR_006320828.1	LOC122580590	uncharacterized LOC122580590	0.0007	down
XM_043781543.1	LOC122608435	probable protein phosphatase 2C 2	0.0019	down
XM_043778298.1	LOC122605366	ribulose bisphosphate carboxylase/oxygenase activase, chloroplastic-like	0.0055	down
XM_043778565.1	LOC122605604	putative protein phosphatase 2C 53	0.0001	down
XM_043765094.1	LOC122592782	chitin-inducible gibberellin-responsive protein 1-like, transcript variant X1	< 0.0001	down
XM_043755351.1	LOC122582922	histidine protein methyltransferase 1 homolog	< 0.0001	down
XM_043774547.1	LOC122601812	subtilisin-like protease	0.0005	down
XM_043750781.1	LOC122578753	chlorophyll a-b binding protein P4, chloroplastic	0.0002	down
XM_043759624.1	LOC122587450	probable serine/threonine-protein kinase DDB_G0281745	< 0.0001	down
XR_006321363.1	LOC122583965	uncharacterized LOC122583965	0.0011	down
XM_043767387.1	LOC122595087	SNW/SKI-interacting protein A-like, transcript variant X3	0.0033	down
XM_043781243.1	LOC122608151	probable WRKY transcription factor 70, transcript variant X1	0.0017	down
XM_043768517.1	LOC122596020	caffeoyl-CoA O-methyltransferase 1	< 0.0001	down
XM_043753200.1	LOC122581058	uncharacterized LOC122581058, transcript variant X2	0.0002	down
XM_043757904.1	LOC122585781	oleosin L-like	0.0004	down
XM_043775596.1	LOC122602982	UDP-glucuronate 4-epimerase 6-like	< 0.0001	down
XM_043782079.1	LOC122609016	scarecrow-like protein 8	< 0.0001	down
XM_043753532.1	LOC122581320	nuclear pore complex protein NUP214, transcript variant X2	0.0046	down
XM_043765612.1	LOC122593230	cytochrome b561 and DOMON domain-containing protein At3g25290	0.0004	down
XM_043765555.1	LOC122593186	cytochrome b561 and DOMON domain-containing protein At5g47530-like	< 0.0001	down
XM_043762118.1	LOC122589795	uncharacterized LOC122589795	0.0019	down
XM_043760381.1	LOC122588263	oleosin-like	0.0021	down
XM_043761663.1	LOC122589373	protein BRASSINAZOLE-RESISTANT 1-like	0.0010	down
XM_043753174.1	LOC122581030	nucleolar protein 58-like	0.0015	down

Transcript id	GeneID	Gene name/function	*P* value	result
XM_043771518.1	LOC122599068	probable serine/threonine-protein kinase At1g54610, transcript variant X1	< 0.0001	down
XM_043752202.1	LOC122579935	FCS-Like Zinc finger 2-like	0.0007	down
XM_043755266.1	LOC122582830	glucan endo-1,3-beta-glucosidase-like	0.0012	down
XM_043770819.1	LOC122598216	probable serine/threonine-protein kinase PBL26, transcript variant X1	0.0004	down
XM_043775385.1	LOC122602788	probable cyclic nucleotide-gated ion channel 20, chloroplastic	0.0040	down
XM_043779109.1	LOC122606148	tRNA N(3)-methylcytidine methyltransferase METTL6, transcript variant X1	0.0001	down
XM_043769042.1	LOC122596462	zinc finger protein CONSTANS-LIKE 5-like	< 0.0001	down
XM_043759663.1	LOC122587477	probable protein phosphatase 2C 12, transcript variant X1	0.0004	down
XM_043773150.1	LOC122600439	transcription factor MYC2-like, transcript variant X2	0.0013	down
XM_043779213.1	LOC122606250	cytochrome P450 711A1-like	< 0.0001	down
XM_043762351.1	LOC122590013	uncharacterized LOC122590013	0.0051	down
XM_043783545.1	LOC122610567	uncharacterized LOC122610567	0.0007	down
XM_043775454.1	LOC122602850	glutamate dehydrogenase B, transcript variant X2	0.0053	down
XM_043777778.1	LOC122604920	receptor kinase-like protein Xa21	0.0005	down
XM_043763876.1	LOC122591614	flotillin-like protein 4	< 0.0001	down
XM_043761230.1	LOC122589002	caffeoylshikimate esterase-like	0.0003	down
XM_043770537.1	LOC122597944	probable receptor-like protein kinase At4g10390	< 0.0001	down
XM_043760969.1	LOC122588774	ACT domain-containing protein ACR12	0.0002	down
XM_043784239.1	LOC122611249	putative disease resistance RPP13-like protein 1	0.0034	down
XM_043778519.1	LOC122605558	syntaxin-121	0.0037	down
XM_043769981.1	LOC122597381	uncharacterized LOC122597381	0.0006	down
XM_043757080.1	LOC122584984	BTB/POZ domain-containing protein At5g41330-like	0.0016	down
XM_043766374.1	LOC122593915	disease resistance protein RGA5-like	0.0011	down
XM_043757227.1	LOC122585127	leucine-rich repeat protein 1-like	< 0.0001	down
XM_043749909.1	LOC122578045	auxin-responsive protein IAA29, transcript variant X1	0.0005	down
XM_043782339.1	LOC122609285	putative F-box protein At2g02030	< 0.0001	down
XM_043770901.1	LOC122598309	ethylene-responsive transcription factor ERF109	< 0.0001	down
XM_043753056.1	LOC122580911	high-affinity nitrate transporter 2.1-like	< 0.0001	down
XM_043757476.1	LOC122585348	uncharacterized LOC122585348	0.0024	down
XM_043783447.1	LOC122610459	keratin, type II cytoskeletal 1-like	0.0036	down
XM_043769376.1	LOC122596747	probable inactive 2-oxoglutarate-dependent dioxygenase AOP2	0.0002	down
XM_043753349.1	LOC122581181	protein FLUORESCENT IN BLUE LIGHT, chloroplastic-like	< 0.0001	down
XM_043751091.1	LOC122579011	protein TIFY 9-like	< 0.0001	down
XM_043750748.1	LOC122578724	prolyl endopeptidase-like	< 0.0001	down
XM_043772221.1	LOC122599676	early nodulin-like protein 2	< 0.0001	down
XM_043753964.1	LOC122581707	pheophytinase, chloroplastic, transcript variant X2	< 0.0001	down
XM_043770321.1	LOC122597752	WRKY transcription factor 22-like	0.0032	down
XM_043778695.1	LOC122605740	probable disease resistance protein At5g66900, transcript variant X1	0.0005	down
XM_043765743.1	LOC122593351	calcium-dependent protein kinase 19-like	< 0.0001	down
XM_043753221.1	LOC122581077	ABC transporter G family member 32-like, transcript variant X1	0.0023	down
XM_043765227.1	LOC122592902	E3 ubiquitin-protein ligase MBR2, transcript variant X3	< 0.0001	down
XM_043751195.1	LOC122579095	F-box protein SKIP14-like	< 0.0001	down
XM_043763634.1	LOC122591366	probable xyloglucan endotransglucosylase/hydrolase protein 23	0.0002	down
XM_043779204.1	LOC122606244	GDSL esterase/lipase At2g23540	0.0047	down
XM_043767446.1	LOC122595132	caffeoyl-CoA O-methyltransferase-like	0.0019	down
XM_043763540.1	LOC122591298	phosphoinositide phospholipase C 4-like	< 0.0001	down
XM_043753688.1	LOC122581455	metallothionein-like protein 1	< 0.0001	down
XM_043779272.1	LOC122606314	probable disease resistance protein At5g66900	0.0024	down
XM_043776263.1	LOC122603539	E3 ubiquitin-protein ligase RING1-like	0.0037	down
XR_006322222.1	LOC122589065	uncharacterized LOC122589065	0.0027	down
XM_043775746.1	LOC122603126	pectinesterase 2-like	0.0003	down
XM_043757887.1	LOC122585764	aquaporin TIP1-3-like	< 0.0001	down
XM_043782768.1	LOC122609827	uncharacterized LOC122609827	< 0.0001	down
XM_043751308.1	LOC122579193	inactive LRR receptor-like serine/threonine-protein kinase BIR2	< 0.0001	down
XM_043761241.1	LOC122589012	uncharacterized LOC122589012	< 0.0001	down
XM_043751945.1	LOC122579721	probable polyol transporter 4	< 0.0001	down
XM_043782841.1	LOC122609887	beta-1,4-mannosyl-glycoprotein 4-beta-N-acetylglucosaminyltransferase-like	< 0.0001	down
XM_043773237.1	LOC122600509	UDP-glucuronate 4-epimerase 1	0.0026	down
XM_043779237.1	LOC122606274	probable cytokinin riboside 5'-monophosphate phosphoribohydrolase LOGL10	0.0047	down
XM_043768291.1	LOC122595835	methylmalonate-semialdehyde dehydrogenase [acylating], mitochondrial-like, transcript variant X1	< 0.0001	down
XM_043763075.1	LOC122590897	protein transport protein SEC31 homolog B-like, transcript variant X2	< 0.0001	down
XM_043780037.1	LOC122607110	catalase, transcript variant X1	0.0043	down
XM_043755811.1	LOC122583406	probable disease resistance protein At5g66900	0.0040	down

Transcript id	GeneID	Gene name/function	*P* value	result
XM_043771056.1	LOC122598460	lysine histidine transporter-like 8	0.0026	down
XM_043781270.1	LOC122608180	V-type proton ATPase subunit a3-like	0.0014	down
XM_043781822.1	LOC122608729	VQ motif-containing protein 19-like	0.0003	down
XM_043772507.1	LOC122599900	MAPK kinase substrate protein At1g80180	0.0013	down
XM_043777259.1	LOC122604366	7-deoxyloganetic acid glucosyl transferase-like, transcript variant X3	0.0021	down
XM_043756200.1	LOC122583820	calcium uniporter protein 6, mitochondrial-like	0.0002	down
XM_043773668.1	LOC122600890	L-type lectin-domain containing receptor kinase IX.1-like	< 0.0001	down
XM_043772191.1	LOC122599650	heat shock factor protein HSF30, transcript variant X2	0.0008	down
XM_043760908.1	LOC122588735	homeobox-leucine zipper protein HAT5	0.0009	down
XM_043772054.1	LOC122599528	umecyanin-like	0.0001	down
XM_043776631.1	LOC122603812	probable amino-acid acetyltransferase NAGS2, chloroplastic	0.0028	down
XM_043761830.1	LOC122589530	ubiquitin-like protein ATG12, transcript variant X2	0.0034	down
XM_043776303.1	LOC122603568	lipid phosphate phosphatase delta-like	< 0.0001	down
XR_006324619.1	LOC122605448	uncharacterized LOC122605448	0.0015	down
XM_043765559.1	LOC122593188	protein DETOXIFICATION 17-like, transcript variant X2	0.0017	down
XM_043750514.1	LOC122578536	protein NRT1/ PTR FAMILY 7.3	0.0003	down
XM_043768917.1	LOC122596352	uncharacterized LOC122596352	< 0.0001	down
XM_043772383.1	LOC122599798	elongation factor 1-alpha-like	< 0.0001	down
XM_043767393.1	LOC122595088	UDP-glycosyltransferase 73C4-like	< 0.0001	down
XM_043764459.1	LOC122592270	probable F-box protein At4g22030	0.0041	down
XM_043757294.1	LOC122585185	ethylene-responsive transcription factor ERF025-like	< 0.0001	down
XM_043754097.1	LOC122581809	histone deacetylase HDT1, transcript variant X1	< 0.0001	down
XM_043784284.1	LOC122611284	DNA polymerase I B, chloroplastic/mitochondrial, transcript variant X2	< 0.0001	down
XM_043775465.1	LOC122602857	phospholipase A1-Ibeta2, chloroplastic-like	< 0.0001	down
XM_043762630.1	LOC122590309	GDSL esterase/lipase At1g71250	0.0012	down
XM_043761293.1	LOC122589051	phosphate transporter PHO1 homolog 9-like	< 0.0001	down
XM_043775671.1	LOC122603057	uncharacterized LOC122603057	< 0.0001	down
XM_043774828.1	LOC122602099	putative ripening-related protein 1	0.0002	down
XM_043757673.1	LOC122585545	uncharacterized LOC122585545	0.0003	down
XM_043779583.1	LOC122606723	phosphoinositide phosphatase SAC2-like, transcript variant X2	< 0.0001	down
XM_043757404.1	LOC122585282	transmembrane 9 superfamily member 3-like, transcript variant X1	< 0.0001	down
XM_043765129.1	LOC122592815	protein SSUH2 homolog	< 0.0001	down
XM_043751963.1	LOC122579738	probable galactinol--sucrose galactosyltransferase 5	0.0018	down
XM_043761235.1	LOC122589007	protein CELLULOSE SYNTHASE INTERACTIVE 1, transcript variant X2	< 0.0001	down
XM_043771835.1	LOC122599338	mitogen-activated protein kinase 15-like	< 0.0001	down
XR_006324416.1	LOC122602884	scarecrow-like protein 9, transcript variant X3	0.0011	down
XM_043756111.1	LOC122583732	cytochrome P450 94C1	0.0023	down
XM_043755559.1	LOC122583126	non-specific lipid transfer protein GPI-anchored 16-like	0.0025	down
XR_006321751.1	LOC122585652	uncharacterized LOC122585652, transcript variant X2	0.0014	down
XM_043761043.1	LOC122588832	queuosine salvage protein-like	< 0.0001	down
XM_043767111.1	LOC122594688	protein LURP-one-related 5-like	0.0041	down
XM_043756459.1	LOC122584246	protein MKS1-like	< 0.0001	down
XM_043759360.1	LOC122587247	F-box protein At1g78280	< 0.0001	down
XM_043783181.1	LOC122610188	23 kDa jasmonate-induced protein-like	< 0.0001	down
XM_043783071.1	LOC122610085	serine/threonine-protein kinase AFC2-like, transcript variant X2	0.0011	down
XM_043775867.1	LOC122603219	myb-related protein 308-like, transcript variant X1	0.0015	down
XM_043759677.1	LOC122587491	protein phosphatase 2C 37-like	0.0033	down
XM_043763249.1	LOC122591056	vesicle-associated protein 4-1-like	0.0029	down
XM_043770684.1	LOC122598073	oxysterol-binding protein-related protein 4C	< 0.0001	down
XM_043781125.1	LOC122608043	probable WRKY transcription factor 70	< 0.0001	down
XM_043766376.1	LOC122593914	BURP domain protein RD22-like	0.0013	down
XM_043753537.1	LOC122581323	calmodulin-binding transcription activator 6-like, transcript variant X2	< 0.0001	down
XM_043754386.1	LOC122582034	ranBP2-type zinc finger protein At1g67325, transcript variant X3	0.0003	down
XM_043765635.1	LOC122593249	phosphatidylinositol:ceramide inositolphosphotransferase 1-like	0.0012	down
XM_043758027.1	LOC122585909	protein HAIKU1-like	< 0.0001	down
XM_043750546.1	LOC122578561	UDP-glucuronate 4-epimerase 6-like	< 0.0001	down
XM_043767353.1	LOC122595063	FT-interacting protein 7-like	< 0.0001	down
XM_043759017.1	LOC122586966	uncharacterized acetyltransferase At3g50280-like, transcript variant X3	0.0002	down
XM_043775898.1	LOC122603238	protein NLP4-like	0.0054	down
XM_043766494.1	LOC122594018	short-chain dehydrogenase reductase 3b-like	0.0001	down
XM_043776147.1	LOC122603444	transcription factor TGA2.3-like, transcript variant X2	0.0004	down
XM_043755455.1	LOC122583017	probable serine/threonine-protein kinase PBL7	0.0001	down
XM_043750429.1	LOC122578458	cytochrome P450 78A5-like	< 0.0001	down
XM_043770698.1	LOC122598091	probable LRR receptor-like serine/threonine-protein kinase At1g67720	0.0031	down
XM_043752827.1	LOC122580556	cytosolic sulfotransferase 12-like	0.0025	down
XM_043776771.1	LOC122603917	mannitol dehydrogenase-like	0.0007	down
XM_043765686.1	LOC122593294	U-box domain-containing protein 17	< 0.0001	down

Transcript id	GeneID	Gene name/function	*P* value	result
XM_043776612.1	LOC122603802	protein PSK SIMULATOR 1-like, transcript variant X2	0.0047	down
XM_043751777.1	LOC122579590	uncharacterized LOC122579590, transcript variant X2	0.0001	down
XM_043768323.1	LOC122595860	ferredoxin-like	< 0.0001	down
XM_043752445.1	LOC122580174	putative disease resistance RPP13-like protein 1, transcript variant X3	0.0012	down
XM_043766368.1	LOC122593911	common plant regulatory factor 1-like, transcript variant X1	< 0.0001	down
XM_043781315.1	LOC122608227	uncharacterized LOC122608227	0.0028	down
XM_043752863.1	LOC122580598	blue copper protein-like	< 0.0001	down
XM_043762042.1	LOC122589717	TOM1-like protein 4	< 0.0001	down
XR_006320492.1	LOC122578247	uncharacterized LOC122578247, transcript variant X1	0.0041	down
XM_043768950.1	LOC122596384	uncharacterized LOC122596384, transcript variant X2	< 0.0001	down
XM_043783721.1	LOC122610762	uncharacterized LOC122610762	0.0005	down
XM_043751947.1	LOC122579723	aldehyde oxidase GLOX-like	0.0003	down
XM_043770562.1	LOC122597967	MLO-like protein 14	< 0.0001	down
XM_043755248.1	LOC122582815	transcription repressor OFP16-like	0.0013	down
XM_043751772.1	LOC122579583	polyamine oxidase 2-like	< 0.0001	down
XM_043763287.1	LOC122591086	dirigent protein 10-like	< 0.0001	down
XM_043763043.1	LOC122590866	heat shock cognate 70 kDa protein-like	0.0019	down
XR_006322144.1	LOC122587425	uncharacterized LOC122587425, transcript variant X2	< 0.0001	down
XM_043753192.1	LOC122581054	leucine-rich repeat receptor-like protein kinase PXC1	0.0004	down
XM_043767792.1	LOC122595433	uncharacterized LOC122595433, transcript variant X3	< 0.0001	down
XM_043752615.1	LOC122580343	geranylgeranyl pyrophosphate synthase 7, chloroplastic-like	< 0.0001	down
XM_043753614.1	LOC122581389	OVARIAN TUMOR DOMAIN-containing deubiquitinating enzyme 12-like, transcript variant X1	0.0010	down
XM_043756759.1	LOC122584712	beta-1,3-galactosyltransferase GALT1	< 0.0001	down
XM_043782560.1	LOC122609524	probable LRR receptor-like serine/threonine-protein kinase IRK	< 0.0001	down
XM_043750791.1	LOC122578759	metal transporter Nramp5-like, transcript variant X1	0.0030	down
XM_043769957.1	LOC122597353	probable polygalacturonase	< 0.0001	down
XM_043752543.1	LOC122580265	zinc finger CCCH domain-containing protein 44-like	0.0045	down
XM_043775313.1	LOC122602723	wall-associated receptor kinase-like 14, transcript variant X1	0.0012	down
XM_043764882.1	LOC122592607	transcription factor MYB102	< 0.0001	down
XM_043765855.1	LOC122593444	cysteine-rich and transmembrane domain-containing protein WIH2-like	< 0.0001	down
XM_043768138.1	LOC122595712	transcription factor MTB1-like	0.0002	down
XM_043767878.1	LOC122595510	protein BIG GRAIN 1-like B	0.0003	down
XM_043784379.1	LOC122611359	protein DA1, transcript variant X1	0.0005	down
XM_043765640.1	LOC122593255	nuclear pore complex protein NUP98A-like	0.0059	down
XR_006322061.1	LOC122586765	uncharacterized LOC122586765, transcript variant X3	< 0.0001	down
XM_043776578.1	LOC122603777	probable glutamate carboxypeptidase AMP1	< 0.0001	down
XM_043751319.1	LOC122579204	uncharacterized LOC122579204	0.0008	down
XM_043769422.1	LOC122596788	putative disease resistance protein At3g14460, transcript variant X1	0.0019	down
XM_043750702.1	LOC122578683	probable galacturonosyltransferase-like 3	< 0.0001	down
XM_043757361.1	LOC122585243	heat stress transcription factor A-3-like	< 0.0001	down
XM_043753563.1	LOC122581346	COBRA-like protein 7	< 0.0001	down
XM_043784164.1	LOC122611185	nucleolin 1, transcript variant X1	0.0006	down
XM_043773480.1	LOC122600727	U-box domain-containing protein 6-like	0.0026	down
XM_043774230.1	LOC122601472	EG45-like domain containing protein	0.0021	down
XM_043778647.1	LOC122605692	protein DETOXIFICATION 49-like	< 0.0001	down
XM_043773250.1	LOC122600519	probable WRKY transcription factor 11	< 0.0001	down
XM_043762950.1	LOC122590778	glycine-rich protein A3-like, transcript variant X2	< 0.0001	down
XM_043752032.1	LOC122579798	putative clathrin assembly protein At4g40080	< 0.0001	down
XM_043763504.1	LOC122591266	serine/threonine-protein kinase RIPK-like	< 0.0001	down
XM_043776237.1	LOC122603513	1-aminocyclopropane-1-carboxylate synthase 3-like	0.0022	down
XM_043775914.1	LOC122603253	probable serine/threonine-protein kinase CST, transcript variant X2	< 0.0001	down
XM_043763290.1	LOC122591091	delta-1-pyrroline-5-carboxylate synthase-like	0.0018	down
XM_043773654.1	LOC122600876	berberine bridge enzyme-like 8	0.0034	down
XM_043760875.1	LOC122588706	mitogen-activated protein kinase kinase kinase 1-like	0.0040	down
XM_043762713.1	LOC122590515	mavicyanin-like	0.0006	down
XM_043770540.1	LOC122597946	ethylene-responsive transcription factor 12-like	< 0.0001	down
XM_043750319.1	LOC122578371	uncharacterized LOC122578371	< 0.0001	down
XM_043771468.1	LOC122599021	auxin response factor 7-like	< 0.0001	down
XM_043769380.1	LOC122596751	pollen-specific leucine-rich repeat extensin-like protein 2	0.0040	down
XM_043767305.1	LOC122595026	vacuolar iron transporter homolog 4-like	< 0.0001	down
XM_043772933.1	LOC122600246	BES1/BZR1 homolog protein 4-like	0.0048	down
XM_043764390.1	LOC122592190	E3 ubiquitin-protein ligase RNF126-like	0.0051	down
XM_043768155.1	LOC122595726	UPF0496 protein 4-like	< 0.0001	down
XM_043779412.1	LOC122606571	farnesyl pyrophosphate synthase-like, transcript variant X2	< 0.0001	down
XM_043761819.1	LOC122589520	uncharacterized protein At5g65660-like	< 0.0001	down
XM_043783378.1	LOC122610386	DNA topoisomerase 6 subunit A3-like	0.0032	down
XM_043760474.1	LOC122588369	IQ domain-containing protein IQM1-like	< 0.0001	down

Transcript id	GeneID	Gene name/function	*P* value	result
XM_043767306.1	LOC122595027	vacuolar iron transporter homolog 4-like	< 0.0001	down
XM_043765766.1	LOC122593371	protein TRANSPORT INHIBITOR RESPONSE 1-like	< 0.0001	down
XM_043777713.1	LOC122604843	E3 ubiquitin-protein ligase UPL7, transcript variant X1	0.0002	down
XM_043776217.1	LOC122603501	UDP-glucose 6-dehydrogenase 3-like	< 0.0001	down
XM_043778920.1	LOC122605955	probable WRKY transcription factor 27	0.0016	down
XM_043773658.1	LOC122600878	omega-3 fatty acid desaturase, chloroplastic-like	0.0019	down
XM_043768579.1	LOC122596066	AP2/ERF and B3 domain-containing transcription factor At1g50680-like, transcript variant X1	0.0010	down
XM_043757051.1	LOC122584958	uncharacterized LOC122584958	0.0002	down
XM_043753872.1	LOC122581630	hsp70 nucleotide exchange factor fes1-like, transcript variant X2	< 0.0001	down
XM_043765918.1	LOC122593498	DExH-box ATP-dependent RNA helicase DExH8	0.0013	down
XM_043782018.1	LOC122608952	protein SHI RELATED SEQUENCE 5-like	0.0019	down
XM_043776373.1	LOC122603620	uncharacterized LOC122603620	0.0025	down
XM_043759156.1	LOC122587081	actin-depolymerizing factor 2	0.0004	down
XM_043772422.1	LOC122599835	zinc finger CCCH domain-containing protein 18	0.0003	down
XM_043776926.1	LOC122604033	probable RNA-dependent RNA polymerase 1	< 0.0001	down
XM_043750968.1	LOC122578915	5'-nucleotidase domain-containing protein 4-like, transcript variant X1	0.0023	down
XM_043752849.1	LOC122580579	auxin response factor 2A-like	< 0.0001	down
XM_043759120.1	LOC122587050	cytochrome P450 81Q32-like	0.0018	down
XM_043783155.1	LOC122610161	uncharacterized LOC122610161	0.0003	down
XM_043764064.1	LOC122591830	CASP-like protein 4D1	0.0014	down
XM_043751882.1	LOC122579667	protein ZW2-like	< 0.0001	down
XM_043764767.1	LOC122592519	protein NRT1/ PTR FAMILY 5.6-like	< 0.0001	down
XM_043779160.1	LOC122606198	two-on-two hemoglobin-3	< 0.0001	down
XR_006322930.1	LOC122594099	chaperone protein dnaJ 72, transcript variant X4	0.0051	down
XM_043766709.1	LOC122594238	membrane protein PM19L-like	< 0.0001	down
XM_043762364.1	LOC122590027	transcription factor bHLH18-like	< 0.0001	down
XM_043780365.1	LOC122607400	nucleobase-ascorbate transporter 6-like	0.0005	down
XM_043770672.1	LOC122598061	cytochrome P450 76T24-like	< 0.0001	down
XM_043763020.1	LOC122590844	mitogen-activated protein kinase kinase kinase 18-like	< 0.0001	down
XM_043763018.1	LOC122590842	tubulin alpha chain	< 0.0001	down
XM_043784364.1	LOC122611343	S-adenosylmethionine decarboxylase proenzyme-like	< 0.0001	down
XM_043765119.1	LOC122592804	RHOMBOID-like protein 1	0.0005	down
XM_043766001.1	LOC122593574	indole-3-acetic acid-amido synthetase GH3.5-like	< 0.0001	down
XM_043778664.1	LOC122605706	type I inositol polyphosphate 5-phosphatase 8	< 0.0001	down
XM_043776088.1	LOC122603400	uncharacterized protein At2g39920-like, transcript variant X4	0.0010	down
XM_043780392.1	LOC122607421	protein TIFY 10B-like	< 0.0001	down
XM_043757112.1	LOC122585016	probable N-acetylglucosaminyl-phosphatidylinositol de-N-acetylase, transcript variant X4	< 0.0001	down
XM_043774682.1	LOC122601950	mitogen-activated protein kinase kinase kinase 3	0.0033	down
XR_006325411.1	LOC122609873	uncharacterized LOC122609873	0.0036	down
XM_043769108.1	LOC122596521	DEAD-box ATP-dependent RNA helicase 52-like	0.0002	down
XM_043775923.1	LOC122603260	probable magnesium transporter NIPA8	0.0015	down
XM_043758384.1	LOC122586390	dehydration-responsive element-binding protein 1E-like	< 0.0001	down
XM_043766177.1	LOC122593732	kinesin-related protein 10-like	0.0020	down
XM_043772043.1	LOC122599516	endochitinase EP3-like	0.0012	down
XM_043773826.1	LOC122601049	putative leucine-rich repeat receptor-like serine/threonine-protein kinase At2g24130	< 0.0001	down
XM_043755238.1	LOC122582801	E3 ubiquitin-protein ligase PUB23-like	< 0.0001	down
XM_043752419.1	LOC122580144	IQ domain-containing protein IQM2-like	< 0.0001	down
XM_043769296.1	LOC122596677	cyclin-U4-1-like	0.0055	down
XM_043773452.1	LOC122600698	ethylene-responsive transcription factor RAP2-3-like	< 0.0001	down
XM_043759566.1	LOC122587405	exportin-T, transcript variant X1	0.0058	down
XM_043776142.1	LOC122603443	serine/threonine-protein kinase/endoribonuclease IRE1b-like	0.0012	down
XM_043756896.1	LOC122584832	rhodanese-like domain-containing protein 14, chloroplastic	0.0032	down
XM_043767829.1	LOC122595464	7-dehydrocholesterol reductase	0.0046	down
XM_043755432.1	LOC122582998	uncharacterized LOC122582998	0.0055	down
XM_043771306.1	LOC122598819	protein LATE ELONGATED HYPOCOTYL-like, transcript variant X1	0.0004	down
XM_043767638.1	LOC122595296	mitogen-activated protein kinase kinase 5	< 0.0001	down
XM_043753209.1	LOC122581067	3-ketoacyl CoA thiolase 1, peroxisomal-like	< 0.0001	down
XM_043782561.1	LOC122609525	O-fucosyltransferase 19-like	0.0002	down
XM_043777091.1	LOC122604196	probable disease resistance protein At4g27220	0.0009	down
XM_043770978.1	LOC122598383	putative pentatricopeptide repeat-containing protein At3g15930, transcript variant X2	0.0036	down
XM_043757379.1	LOC122585261	NAC domain-containing protein 90-like	< 0.0001	down
XM_043755141.1	LOC122582717	cell wall / vacuolar inhibitor of fructosidase 1-like	0.0033	down
XR_006323282.1	LOC122595972	uncharacterized LOC122595972, transcript variant X7	< 0.0001	down
XM_043775301.1	LOC122602710	MLO protein homolog 1-like	0.0014	down

Transcript id	GeneID	Gene name/function	P value	result
XR_006321656.1	LOC122585084	uncharacterized LOC122585084, transcript variant X4	< 0.0001	down
XM_043773252.1	LOC122600524	glucan endo-1,3-beta-glucosidase 12-like	0.0001	down
XM_043769356.1	LOC122596731	cytochrome P450 76T24-like	< 0.0001	down
XM_043774115.1	LOC122601347	uncharacterized LOC122601347	< 0.0001	down
XM_043769176.1	LOC122596568	anaphase-promoting complex subunit 4, transcript variant X1	0.0052	down
XM_043763394.1	LOC122591173	putative F-box protein At1g32420	< 0.0001	down
XM_043760799.1	LOC122588643	dehydration-responsive element-binding protein 1E-like	0.0005	down
XM_043761442.1	LOC122589191	short-chain dehydrogenase TIC 32 B, chloroplastic	0.0031	down
XR_006323999.1	LOC122600093	uncharacterized LOC122600093	0.0021	down
XM_043778943.1	LOC122605978	uncharacterized protein At3g28850-like	< 0.0001	down
XM_043765626.1	LOC122593241	BTB/POZ domain-containing protein At4g30940	< 0.0001	down
XM_043756162.1	LOC122583790	non-specific lipid transfer protein GPI-anchored 5-like	0.0003	down
XM_043750390.1	LOC122578429	U-box domain-containing protein 45-like	0.0003	down
XM_043754920.1	LOC122582515	alpha carbonic anhydrase 7-like	0.0017	down
XR_006323956.1	LOC122599845	uncharacterized LOC122599845, transcript variant X6	0.0004	down
XM_043751078.1	LOC122579000	probable receptor-like protein kinase At1g80640, transcript variant X1	0.0008	down
XM_043775289.1	LOC122602701	F-box/kelch-repeat protein At3g23880-like, transcript variant X5	< 0.0001	down
XM_043766437.1	LOC122593961	mitogen-activated protein kinase kinase kinase 18-like	0.0005	down
XM_043771653.1	LOC122599182	inositol transporter 1-like, transcript variant X1	< 0.0001	down
XM_043776825.1	LOC122603957	E3 ubiquitin-protein ligase WAV3	< 0.0001	down
XM_043767304.1	LOC122595024	vacuolar iron transporter homolog 4-like	< 0.0001	down
XM_043757919.1	LOC122585794	protein EARLY-RESPONSIVE TO DEHYDRATION 7, chloroplastic-like	0.0010	down
XM_043764030.1	LOC122591793	disease resistance protein RUN1-like	0.0029	down
XM_043756299.1	LOC122583924	carotenoid cleavage dioxygenase 8 homolog B, chloroplastic-like	0.0023	down
XM_043751027.1	LOC122578961	proline dehydrogenase 2, mitochondrial-like	0.0018	down
XM_043774699.1	LOC122601966	auxin-responsive protein IAA16-like	< 0.0001	down
XM_043782633.1	LOC122609685	uncharacterized LOC122609685	< 0.0001	down
XM_043773561.1	LOC122600798	probable protein phosphatase 2C 72, transcript variant X2	0.0044	down
XM_043776215.1	LOC122603499	UDP-glycosyltransferase 88B1-like	0.0004	down
XM_043753134.1	LOC122580990	zinc finger protein 10	< 0.0001	down
XM_043756713.1	LOC122584668	WAT1-related protein At1g70260	< 0.0001	down
XM_043766551.1	LOC122594078	uncharacterized protein C24B11.05-like	0.0002	down
XM_043751674.1	LOC122579495	uncharacterized LOC122579495	< 0.0001	down
XM_043768719.1	LOC122596187	small polypeptide DEVIL 4-like	< 0.0001	down
XM_043751745.1	LOC122579556	phosphatidylinositol 4-kinase gamma 5-like	< 0.0001	down
XM_043772795.1	LOC122600131	tubulin alpha-2 chain-like	< 0.0001	down
XM_043780902.1	LOC122607841	CBL-interacting protein kinase 2-like, transcript variant X1	0.0010	down
XM_043779023.1	LOC122606058	inverted formin-2-like	0.0018	down
XM_043751970.1	LOC122579743	uncharacterized LOC122579743	< 0.0001	down
XM_043773423.1	LOC122600670	myb family transcription factor EFM	0.0031	down
XM_043750665.1	LOC122578659	B3 domain-containing protein Os03g0120900-like, transcript variant X2	0.0010	down
XM_043751644.1	LOC122579460	probable receptor-like protein kinase At1g11050	0.0014	down
XM_043766736.1	LOC122594262	protein NRT1/ PTR FAMILY 4.5-like	0.0028	down
XM_043756178.1	LOC122583803	protein unc-13 homolog	< 0.0001	down
XM_043782580.1	LOC122609552	LON peptidase N-terminal domain and RING finger protein 1-like, transcript variant X2	0.0054	down
XM_043779991.1	LOC122607070	dirigent protein 22-like	< 0.0001	down
XM_043759367.1	LOC122587253	ribulose bisphosphate carboxylase/oxygenase activase, chloroplastic	0.0029	down
XM_043765072.1	LOC122592766	non-lysosomal glucosylceramidase-like, transcript variant X7	0.0024	down
XM_043751539.1	LOC122579374	probable methyltransferase At1g27930	0.0018	down
XM_043776138.1	LOC122603438	alcohol acyltransferase 9-like	0.0009	down
XM_043764845.1	LOC122592583	helicase-like transcription factor CHR28	< 0.0001	down
XM_043760893.1	LOC122588721	protein RADIALIS-like 3	< 0.0001	down
XM_043758576.1	LOC122586594	aquaporin TIP2-1	0.0040	down
XM_043754409.1	LOC122582061	dirigent protein 21-like	< 0.0001	down
XM_043777782.1	LOC122604923	protein FAR1-RELATED SEQUENCE 11-like	0.0021	down
XM_043776319.1	LOC122603581	patatin-like protein 7	0.0029	down
XM_043757474.1	LOC122585345	ethylene-responsive transcription factor ERF061-like	0.0012	down
XM_043763336.1	LOC122591121	fimbrin-1-like	< 0.0001	down
XM_043778758.1	LOC122605802	protein SINE1-like	0.0010	down
XM_043758118.1	LOC122586003	uncharacterized LOC122586003	0.0010	down
XM_043759179.1	LOC122587101	metacaspase-1-like	0.0003	down
XM_043781262.1	LOC122608172	dof zinc finger protein DOF3.1-like	0.0047	down
XM_043754323.1	LOC122581987	phosphomethylpyrimidine synthase, chloroplastic	< 0.0001	down
XM_043776275.1	LOC122603549	protein DA1-related 2-like	0.0005	down
XM_043778189.1	LOC122605280	phosphoethanolamine N-methyltransferase 3-like	< 0.0001	down

Transcript id	GeneID	Gene name/function	*P* value	result
XM_043754754.1	LOC122582370	serine/arginine-rich SC35-like splicing factor SCL30A, transcript variant X2	< 0.0001	down
XM_043765742.1	LOC122593349	protein CYPRO4-like	< 0.0001	down
XM_043751929.1	LOC122579706	zinc finger CCCH domain-containing protein 34-like	< 0.0001	down
XR_006321641.1	LOC122585027	uncharacterized LOC122585027	< 0.0001	down
XM_043754072.1	LOC122581793	TOM1-like protein 9	0.0004	down
XM_043784236.1	LOC122611248	metal-nicotianamine transporter YSL1-like, transcript variant X2	< 0.0001	down
XM_043752995.1	LOC122580839	serine/threonine-protein phosphatase BSL1-like, transcript variant X1	< 0.0001	down
XM_043767339.1	LOC122595054	probable serine/threonine-protein kinase PBL19, transcript variant X1	0.0006	down
XM_043764478.1	LOC122592286	formin-like protein 18, transcript variant X5	< 0.0001	down
XM_043753677.1	LOC122581445	uridine 5'-monophosphate synthase-like	0.0002	down
XM_043776446.1	LOC122603676	S-type anion channel SLAH2-like, transcript variant X1	< 0.0001	down
XM_043772717.1	LOC122600063	F-box protein At2g02240-like, transcript variant X1	0.0001	down
XM_043768003.1	LOC122595606	U-box domain-containing protein 4	< 0.0001	down
XM_043779716.1	LOC122606838	probable pre-mRNA-splicing factor ATP-dependent RNA helicase DEAH2, transcript variant X1	0.0029	down
XM_043754227.1	LOC122581911	mediator of RNA polymerase II transcription subunit 7a-like, transcript variant X1	< 0.0001	down
XM_043758903.1	LOC122586886	calcium-transporting ATPase 2, plasma membrane-type-like	0.0013	down
XM_043784082.1	LOC122611114	uncharacterized LOC122611114	< 0.0001	down
XM_043770549.1	LOC122597954	ACD11 homolog protein	0.0010	down
XM_043758412.1	LOC122586419	expansin-A1-like	0.0002	down
XM_043772296.1	LOC122599737	glutaredoxin-C6-like	0.0026	down
XM_043756410.1	LOC122584166	classical arabinogalactan protein 1-like	0.0046	down
XM_043762060.1	LOC122589734	UDP-glycosyltransferase 85C2-like	0.0040	down
XM_043780638.1	LOC122607624	uncharacterized LOC122607624	< 0.0001	down
XM_043753909.1	LOC122581663	E3 ubiquitin-protein ligase DIS1-like, transcript variant X1	< 0.0001	down
XM_043758312.1	LOC122586310	probable WRKY transcription factor 70	0.0002	down
XM_043762346.1	LOC122590008	putative UPF0481 protein At3g02645	< 0.0001	down
XM_043757338.1	LOC122585223	uncharacterized LOC122585223	0.0051	down
XM_043757176.1	LOC122585080	monothiol glutaredoxin-S2-like	0.0051	down
XR_006321691.1	LOC122585333	uncharacterized LOC122585333	< 0.0001	down
XM_043783762.1	LOC122610808	LEAF RUST 10 DISEASE-RESISTANCE LOCUS RECEPTOR-LIKE PROTEIN KINASE-like 2.1	< 0.0001	down
XM_043756644.1	LOC122584545	mitogen-activated protein kinase 9-like	< 0.0001	down
XR_006324830.1	LOC122606191	uncharacterized LOC122606191	< 0.0001	down
XM_043771731.1	LOC122599251	protein ANTAGONIST OF LIKE HETEROCHROMATIN PROTEIN 1-like	< 0.0001	down
XM_043758863.1	LOC122586860	aminopeptidase M1-like, transcript variant X3	0.0005	down
XM_043767301.1	LOC122595021	vacuolar iron transporter homolog 4-like	0.0004	down
XM_043754896.1	LOC122582494	uncharacterized LOC122582494, transcript variant X2	0.0019	down
XM_043756445.1	LOC122584228	uncharacterized LOC122584228, transcript variant X1	< 0.0001	down
XM_043753046.1	LOC122580901	myb family transcription factor PHL7-like	0.0014	down
XM_043781398.1	LOC122608307	ABC transporter A family member 2-like, transcript variant X1	0.0025	down
XM_043777843.1	LOC122604987	late embryogenesis abundant protein At1g64065-like	< 0.0001	down
XM_043757124.1	LOC122585025	lipid phosphate phosphatase 2-like	0.0002	down
XM_043756748.1	LOC122584700	dehydration-responsive element-binding protein 1D-like	< 0.0001	down
XM_043776105.1	LOC122603413	jasmonate-induced oxygenase 2-like	< 0.0001	down
XM_043778376.1	LOC122605425	uncharacterized LOC122605425	< 0.0001	down
XM_043784191.1	LOC122611210	protein NRT1/ PTR FAMILY 4.6-like, transcript variant X1	0.0007	down
XM_043772523.1	LOC122599913	3-ketoacyl-CoA synthase 9-like	< 0.0001	down
XM_043767838.1	LOC122595471	RNA demethylase ALKBH9B-like	< 0.0001	down
XM_043752308.1	LOC122580038	probable aspartyl protease At4g16563	0.0006	down
XR_006322255.1	LOC122589377	uncharacterized LOC122589377	0.0001	down
XM_043778926.1	LOC122605962	transcription factor LHW, transcript variant X2	0.0008	down
XM_043752332.1	LOC122580061	class V chitinase CHIT5-like	< 0.0001	down
XM_043776827.1	LOC122603960	dehydration-responsive element-binding protein 1D-like	0.0018	down
XM_043764914.1	LOC122592632	trifunctional UDP-glucose 4,6-dehydratase/UDP-4-keto-6-deoxy-D-glucose 3,5-epimerase/UDP-4-keto-L-rhamnose-reductase RHM1-like	< 0.0001	down
XM_043760402.1	LOC122588288	protein JINGUBANG-like	0.0018	down
XM_043772944.1	LOC122600254	transcription factor GTE3, chloroplastic-like, transcript variant X3	< 0.0001	down
XM_043773206.1	LOC122600486	G2/mitotic-specific cyclin S13-7-like, transcript variant X2	0.0003	down
XM_043756735.1	LOC122584688	nudix hydrolase 17, mitochondrial-like	0.0059	down
XM_043755333.1	LOC122582904	protein MALE DISCOVERER 2-like, transcript variant X4	0.0038	down
XM_043775140.1	LOC122602533	putative clathrin assembly protein At2g25430	0.0007	down
XM_043765657.1	LOC122593270	purple acid phosphatase 2-like	< 0.0001	down
XM_043765544.1	LOC122593174	9-cis-epoxycarotenoid dioxygenase NCED1, chloroplastic-like	0.0022	down
XM_043758702.1	LOC122586712	laccase-12-like	0.0037	down
XM_043751895.1	LOC122579673	uncharacterized LOC122579673, transcript variant X1	< 0.0001	down

Transcript id	GeneID	Gene name/function	*P* value	result
XM_043784048.1	LOC122611086	probable 2-oxoglutarate-dependent dioxygenase AOP1	0.0037	down
XM_043779370.1	LOC122606501	TMV resistance protein N-like, transcript variant X2	0.0001	down
XM_043753774.1	LOC122581545	thaumatin-like protein	0.0026	down
XM_043781150.1	LOC122608064	ER lumen protein-retaining receptor erd-2.2-like	0.0009	down
XM_043769436.1	LOC122596792	trimethyltridecatetraene synthase-like	< 0.0001	down
XM_043779031.1	LOC122606069	protein DWARF 53-like	< 0.0001	down
XM_043778543.1	LOC122605580	uncharacterized LOC122605580	< 0.0001	down
XM_043751325.1	**LOC122579206**	probable serine/threonine-protein kinase PBL28, transcript variant X1	< 0.0001	down
XM_043751331.1	**LOC122579206**	probable serine/threonine-protein kinase PBL28, transcript variant X1	< 0.0001	down
XM_043752181.1	**LOC122579917**	nuclear transport factor 2-like, transcript variant X3	0.0014	down
XM_043752179.1	**LOC122579917**	nuclear transport factor 2-like, transcript variant X3	0.0017	down
XM_043752460.1	**LOC122580176**	uncharacterized protein At1g76660-like, transcript variant X1	< 0.0001	down
XM_043752452.1	**LOC122580176**	uncharacterized protein At1g76660-like, transcript variant X1	0.0033	down
XM_043752896.1	**LOC122580601**	nuclear transcription factor Y subunit B-1-like, transcript variant X6	0.0010	down
XM_043752874.1	**LOC122580601**	nuclear transcription factor Y subunit B-1-like, transcript variant X6	< 0.0001	down
XM_043754001.1	**LOC122581733**	myb-related protein 2, transcript variant X2	< 0.0001	down
XM_043754000.1	**LOC122581733**	myb-related protein 2, transcript variant X2	0.0023	down
XM_043755623.1	**LOC122583188**	synaptotagmin-2-like, transcript variant X1	< 0.0001	down
XM_043755627.1	**LOC122583188**	synaptotagmin-2-like, transcript variant X1	< 0.0001	down
XM_043756331.1	**LOC122583962**	probable LRR receptor-like serine/threonine-protein kinase RFK1, transcript variant X2	0.0006	down
XM_043756332.1	**LOC122583962**	probable LRR receptor-like serine/threonine-protein kinase RFK1, transcript variant X2	0.0011	down
XM_043757772.1	**LOC122585644**	uncharacterized LOC122585644, transcript variant X1	< 0.0001	down
XM_043757774.1	**LOC122585644**	uncharacterized LOC122585644, transcript variant X1	< 0.0001	down
XM_043757770.1	**LOC122585644**	uncharacterized LOC122585644, transcript variant X1	< 0.0001	down
XM_043757775.1	**LOC122585644**	uncharacterized LOC122585644, transcript variant X1	< 0.0001	down
XM_043758215.1	**LOC122586108**	berberine bridge enzyme-like 21, transcript variant X1	< 0.0001	down
XM_043758214.1	**LOC122586108**	berberine bridge enzyme-like 21, transcript variant X1	0.0009	down
XM_043758498.1	**LOC122586514**	NAC domain-containing protein 91-like, transcript variant X1	0.0016	down
XM_043758497.1	**LOC122586514**	NAC domain-containing protein 91-like, transcript variant X1	0.0018	down
XM_043758647.1	**LOC122586665**	choline/ethanolaminephosphotransferase 1-like, transcript variant X1	0.0013	down
XM_043758645.1	**LOC122586665**	choline/ethanolaminephosphotransferase 1-like, transcript variant X1	< 0.0001	down
XM_043758687.1	**LOC122586705**	truncated transcription factor CAULIFLOWER D-like, transcript variant X1	< 0.0001	down
XM_043758688.1	**LOC122586705**	truncated transcription factor CAULIFLOWER D-like, transcript variant X1	< 0.0001	down
XM_043759141.1	**LOC122587068**	uncharacterized LOC122587068, transcript variant X3	< 0.0001	down
XR_006322111.1	**LOC122587068**	uncharacterized LOC122587068, transcript variant X3	0.0046	down
XM_043759170.1	**LOC122587096**	probable WRKY transcription factor 40, transcript variant X2	< 0.0001	down
XM_043759171.1	**LOC122587096**	probable WRKY transcription factor 40, transcript variant X2	0.0003	down
XM_043759322.1	**LOC122587217**	uncharacterized LOC122587217, transcript variant X3	< 0.0001	down
XM_043759324.1	**LOC122587217**	uncharacterized LOC122587217, transcript variant X3	0.0045	down
XM_043760610.1	**LOC122588483**	nuclear poly(A) polymerase 4-like, transcript variant X3	0.0002	down
XM_043760612.1	**LOC122588483**	nuclear poly(A) polymerase 4-like, transcript variant X3	0.0024	down
XM_043761081.1	**LOC122588866**	thioredoxin H2-like, transcript variant X2	< 0.0001	down
XM_043761082.1	**LOC122588866**	thioredoxin H2-like, transcript variant X2	0.0006	down
XM_043761807.1	**LOC122589507**	calcium-dependent mitochondrial ATP-magnesium/phosphate carrier protein 2-like, transcript variant X2	0.0031	down
XM_043761806.1	**LOC122589507**	calcium-dependent mitochondrial ATP-magnesium/phosphate carrier protein 2-like, transcript variant X2	0.0006	down
XM_043762181.1	**LOC122589860**	uroporphyrinogen-III synthase, chloroplastic, transcript variant X1	0.0002	down
XM_043762180.1	**LOC122589860**	uroporphyrinogen-III synthase, chloroplastic, transcript variant X1	0.0003	down
XM_043762182.1	**LOC122589860**	uroporphyrinogen-III synthase, chloroplastic, transcript variant X1	< 0.0001	down
XM_043762930.1	**LOC122590754**	probable protein phosphatase 2C 34, transcript variant X1	< 0.0001	down
XM_043762928.1	**LOC122590754**	probable protein phosphatase 2C 34, transcript variant X1	0.0013	down
XM_043763009.1	**LOC122590838**	receptor-like protein EIX2, transcript variant X1	< 0.0001	down
XM_043763013.1	**LOC122590838**	receptor-like protein EIX2, transcript variant X1	0.0024	down
XM_043763010.1	**LOC122590838**	receptor-like protein EIX2, transcript variant X1	< 0.0001	down
XM_043763369.1	**LOC122591151**	probable protein phosphatase 2C 49, transcript variant X1	0.0020	down
XM_043763368.1	**LOC122591151**	probable protein phosphatase 2C 49, transcript variant X1	< 0.0001	down
XM_043763549.1	**LOC122591304**	uncharacterized LOC122591304, transcript variant X11	< 0.0001	down
XM_043763555.1	**LOC122591304**	uncharacterized LOC122591304, transcript variant X11	0.0029	down
XM_043764519.1	**LOC122592316**	G-type lectin S-receptor-like serine/threonine-protein kinase SD1-1, transcript variant X1	0.0001	down
XM_043764520.1	**LOC122592316**	G-type lectin S-receptor-like serine/threonine-protein kinase SD1-1, transcript variant X1	0.0045	down
XM_043766154.1	**LOC122593706**	metacaspase-1-like, transcript variant X1	0.0029	down
XM_043766153.1	**LOC122593706**	metacaspase-1-like, transcript variant X1	0.0026	down

Transcript id	GeneID	Gene name/function	*P* value	result
XM_043766431.1	**LOC122593957**	probable F-box protein At2g36090, transcript variant X1	0.0010	down
XM_043766432.1	**LOC122593957**	probable F-box protein At2g36090, transcript variant X1	< 0.0001	down
XM_043767531.1	**LOC122595198**	nuclear pore complex protein NUP205, transcript variant X4	0.0037	down
XM_043767530.1	**LOC122595198**	nuclear pore complex protein NUP205, transcript variant X4	0.0041	down
XM_043768148.1	**LOC122595720**	BES1/BZR1 homolog protein 4-like, transcript variant X2	< 0.0001	down
XM_043768149.1	**LOC122595720**	BES1/BZR1 homolog protein 4-like, transcript variant X2	< 0.0001	down
XR_006323251.1	**LOC122595734**	serine/arginine-rich splicing factor SR30-like, transcript variant X2	0.0003	down
XR_006323252.1	**LOC122595734**	serine/arginine-rich splicing factor SR30-like, transcript variant X2	0.0012	down
XR_006323253.1	**LOC122595734**	serine/arginine-rich splicing factor SR30-like, transcript variant X2	0.0016	down
XM_043768365.1	**LOC122595892**	heptahelical transmembrane protein 1-like, transcript variant X1	0.0009	down
XM_043768364.1	**LOC122595892**	heptahelical transmembrane protein 1-like, transcript variant X1	0.0026	down
XM_043768897.1	**LOC122596335**	magnesium transporter MRS2-3-like, transcript variant X4	0.0034	down
XM_043768895.1	**LOC122596335**	magnesium transporter MRS2-3-like, transcript variant X4	0.0037	down
XM_043768893.1	**LOC122596335**	magnesium transporter MRS2-3-like, transcript variant X4	< 0.0001	down
XM_043769452.1	**LOC122596808**	histone-lysine N-methyltransferase, H3 lysine-9 specific SUVH4-like, transcript variant X1	0.0005	down
XM_043769453.1	**LOC122596808**	histone-lysine N-methyltransferase, H3 lysine-9 specific SUVH4-like, transcript variant X1	< 0.0001	down
XR_006323398.1	**LOC122596995**	cyclin-dependent kinase G-2-like, transcript variant X6	0.0034	down
XR_006323401.1	**LOC122596995**	cyclin-dependent kinase G-2-like, transcript variant X6	0.0001	down
XM_043769639.1	**LOC122596995**	cyclin-dependent kinase G-2-like, transcript variant X6	< 0.0001	down
XM_043769653.1	**LOC122596995**	cyclin-dependent kinase G-2-like, transcript variant X6	< 0.0001	down
XM_043773038.1	**LOC122600331**	probable WRKY transcription factor 53, transcript variant X2	0.0034	down
XM_043773039.1	**LOC122600331**	probable WRKY transcription factor 53, transcript variant X2	0.0009	down
XM_043775121.1	**LOC122602434**	two-component response regulator ARR17, transcript variant X2	0.0003	down
XM_043775118.1	**LOC122602434**	two-component response regulator ARR17, transcript variant X2	< 0.0001	down
XM_043775798.1	**LOC122603165**	exocyst complex component SEC8, transcript variant X1	< 0.0001	down
XM_043775796.1	**LOC122603165**	exocyst complex component SEC8, transcript variant X1	0.0004	down
XM_043776700.1	**LOC122603868**	protein REVEILLE 8, transcript variant X3	0.0006	down
XM_043776702.1	**LOC122603868**	protein REVEILLE 8, transcript variant X3	< 0.0001	down
XM_043777474.1	**LOC122604587**	monosaccharide-sensing protein 2, transcript variant X2	0.0009	down
XM_043777470.1	**LOC122604587**	monosaccharide-sensing protein 2, transcript variant X2	< 0.0001	down
XM_043777829.1	**LOC122604965**	TNF receptor-associated factor homolog 1a-like, transcript variant X6	< 0.0001	down
XM_043777853.1	**LOC122604965**	TNF receptor-associated factor homolog 1a-like, transcript variant X6	0.0042	down
XM_043777844.1	**LOC122604965**	TNF receptor-associated factor homolog 1a-like, transcript variant X6	0.0019	down
XM_043778687.1	**LOC122605728**	pleiotropic drug resistance protein 1-like, transcript variant X1	0.0019	down
XM_043778688.1	**LOC122605728**	pleiotropic drug resistance protein 1-like, transcript variant X1	< 0.0001	down
XM_043779668.1	**LOC122606810**	cysteine-tryptophan domain-containing zinc finger protein 5-like, transcript variant X1	< 0.0001	down
XM_043779669.1	**LOC122606810**	cysteine-tryptophan domain-containing zinc finger protein 5-like, transcript variant X1	0.0005	down
XM_043780045.1	**LOC122607126**	extensin-like, transcript variant X1	0.0002	down
XM_043780046.1	**LOC122607126**	extensin-like, transcript variant X1	0.0008	down
XM_043780170.1	**LOC122607234**	septin and tuftelin-interacting protein 1 homolog 1-like, transcript variant X2	< 0.0001	down
XM_043780169.1	**LOC122607234**	septin and tuftelin-interacting protein 1 homolog 1-like, transcript variant X2	0.0046	down
XM_043780802.1	**LOC122607766**	transcription factor BIM1-like, transcript variant X1	0.0012	down
XM_043780803.1	**LOC122607766**	transcription factor BIM1-like, transcript variant X1	0.0001	down
XR_006325439.1	**LOC122610000**	uncharacterized LOC122610000, transcript variant X1	< 0.0001	down
XR_006325440.1	**LOC122610000**	uncharacterized LOC122610000, transcript variant X1	0.0034	down
XM_043784240.1	**LOC122611240**	protein TIFY 6B-like, transcript variant X5	0.0004	down
XM_043784232.1	**LOC122611240**	protein TIFY 6B-like, transcript variant X5	0.0010	down
XM_043784247.1	**LOC122611240**	protein TIFY 6B-like, transcript variant X5	0.0004	down
XM_043784397.1	**LOC122611374**	putative late blight resistance protein homolog R1A-10, transcript variant X1	0.0048	down
XM_043784398.1	**LOC122611374**	putative late blight resistance protein homolog R1A-10, transcript variant X1	< 0.0001	down
XM_043750191.1	**LOC122578271**	probable receptor-like protein kinase At5g18500, transcript variant X2	< 0.0001	up
XM_043750192.1	**LOC122578271**	probable receptor-like protein kinase At5g18500, transcript variant X2	< 0.0001	down
XM_043750778.1	**LOC122578752**	agamous-like MADS-box protein AGL65, transcript variant X2	0.0012	up
XM_043750779.1	**LOC122578752**	agamous-like MADS-box protein AGL65, transcript variant X2	0.0005	down
XM_043761794.1	**LOC122589499**	kinesin-like protein KIN-14C, transcript variant X1	< 0.0001	up
XM_043761795.1	**LOC122589499**	kinesin-like protein KIN-14C, transcript variant X1	0.0015	down
XM_043765644.1	**LOC122593258**	pentatricopeptide repeat-containing protein At5g39350, transcript variant X3	< 0.0001	up
XM_043765643.1	**LOC122593258**	pentatricopeptide repeat-containing protein At5g39350, transcript variant X3	< 0.0001	down
XM_043765803.1	**LOC122593399**	uncharacterized LOC122593399, transcript variant X2	0.0002	up

Transcript id	GeneID	Gene name/function	*P* value	result
XM_043765804.1	**LOC122593399**	uncharacterized LOC122593399, transcript variant X2	0.0002	down
XM_043767247.1	**LOC122594967**	BTB/POZ domain-containing protein At1g67900, transcript variant X1	< 0.0001	up
XM_043767248.1	**LOC122594967**	BTB/POZ domain-containing protein At1g67900, transcript variant X1	< 0.0001	down
XM_043771467.1	**LOC122599020**	uncharacterized LOC122599020, transcript variant X1	0.0002	up
XM_043771466.1	**LOC122599020**	uncharacterized LOC122599020, transcript variant X1	< 0.0001	down
XR_006323901.1	**LOC122599322**	uncharacterized LOC122599322, transcript variant X7	0.0002	up
XR_006323900.1	**LOC122599322**	uncharacterized LOC122599322, transcript variant X7	0.0033	down
XM_043773061.1	**LOC122600356**	uncharacterized LOC122600356, transcript variant X2	0.0026	up
XM_043773063.1	**LOC122600356**	uncharacterized LOC122600356, transcript variant X2	< 0.0001	down
XM_043774722.1	**LOC122601990**	ATP-dependent DNA helicase Q-like SIM, transcript variant X1	0.0003	up
XM_043774721.1	**LOC122601990**	ATP-dependent DNA helicase Q-like SIM, transcript variant X1	< 0.0001	down
XM_043777980.1	**LOC122605102**	glycosyltransferase-like protein gnt14, transcript variant X1	< 0.0001	up
XM_043777977.1	**LOC122605102**	glycosyltransferase-like protein gnt14, transcript variant X1	0.0016	down
XM_043780176.1	**LOC122607238**	ubiquitin carboxyl-terminal hydrolase 24, transcript variant X1	0.0055	up
XM_043780183.1	**LOC122607238**	ubiquitin carboxyl-terminal hydrolase 24, transcript variant X1	0.0005	down
XM_043781869.1	**LOC122608773**	myosin-1-like, transcript variant X2	< 0.0001	up
XM_043781863.1	**LOC122608773**	myosin-1-like, transcript variant X2	< 0.0001	down
XM_043782503.1	**LOC122609423**	U11/U12 small nuclear ribonucleoprotein 65 kDa protein, transcript variant X7	0.0001	up
XM_043782507.1	**LOC122609423**	U11/U12 small nuclear ribonucleoprotein 65 kDa protein, transcript variant X7	0.0008	down
XM_043782823.1	**LOC122609878**	probable protein phosphatase 2C 60, transcript variant X1	< 0.0001	up
XM_043782822.1	**LOC122609878**	probable protein phosphatase 2C 60, transcript variant X1	0.0041	down

MC represents the moderate concentration treatment (2 mg/kg Cd and 180 mg/kg Pb). Locus IDs shown in bold indicate that more than one transcript is associated with the same gene.

Table S5 Differentially expressed genes (DEGs) identified in *Erigeron canadensis* roots under the HC treatment

Transcript id	GeneID	Gene name/function	*P* Value	result
XM_043772114.1	LOC122599585	uncharacterized LOC122599585	0.0013	up
XM_043762283.1	LOC122589952	50S ribosomal protein L29, chloroplastic	< 0.0001	up
XM_043762088.1	LOC122589765	WD repeat and HMG-box DNA-binding protein 1	< 0.0001	up
XM_043765637.1	LOC122593251	39S ribosomal protein L46, mitochondrial	0.0023	up
XM_043763461.1	LOC122591230	(S)-2-hydroxy-acid oxidase GLO1	0.0059	up
XM_043762003.1	LOC122589680	BEL1-like homeodomain protein 3, transcript variant X2	< 0.0001	up
XM_043766888.1	LOC122594434	protein disulfide isomerase-like 5-4	0.0010	up
XM_043768039.1	LOC122595633	uncharacterized LOC122595633, transcript variant X2	0.0011	up
XM_043753855.1	LOC122581609	probable 6-phosphogluconolactonase 1	< 0.0001	up
XM_043755209.1	LOC122582772	ABC transporter B family member 26, chloroplastic	< 0.0001	up
XM_043765455.1	LOC122593100	probable NAD(P)H dehydrogenase (quinone) FQR1-like 3	0.0047	up
XM_043771343.1	LOC122598863	uncharacterized LOC122598863	0.0057	up
XM_043767439.1	LOC122595123	trafficking protein particle complex subunit 6B-like	0.0023	up
XM_043776279.1	LOC122603552	uncharacterized LOC122603552	0.0056	up
XM_043782725.1	LOC122609783	transcriptional elongation regulator MINIYO	0.0008	up
XM_043760253.1	LOC122588118	uncharacterized LOC122588118	0.0032	up
XR_006322388.1	LOC122590029	uncharacterized LOC122590029	0.0020	up
XM_043772712.1	LOC122600061	arginine--tRNA ligase, chloroplastic/mitochondrial-like, transcript variant X1	0.0015	up
XM_043756898.1	LOC122584834	uncharacterized LOC122584834	0.0013	up
XM_043751192.1	LOC122579091	magnesium transporter MRS2-I-like, transcript variant X2	0.0062	up
XM_043780159.1	LOC122607223	uncharacterized LOC122607223	0.0002	up
XM_043768152.1	LOC122595723	uncharacterized LOC122595723	0.0017	up
XM_043769056.1	LOC122596475	homeobox protein knotted-1-like 6	0.0010	up
XM_043775858.1	LOC122603214	pentatricopeptide repeat-containing protein At5g21222-like, transcript variant X3	0.0010	up
XM_043763617.1	LOC122591353	lipoyl synthase, chloroplastic, transcript variant X2	< 0.0001	up
XM_043753671.1	LOC122581438	L-galactono-1,4-lactone dehydrogenase, mitochondrial	0.0003	up
XM_043776546.1	LOC122603752	protein NPGR2-like	< 0.0001	up
XM_043755481.1	LOC122583039	plant cysteine oxidase 2-like, transcript variant X1	0.0004	up
XM_043752001.1	LOC122579772	tetraketide alpha-pyrone reductase 1, transcript variant X2	0.0002	up
XM_043770555.1	LOC122597960	36.4 kDa proline-rich protein-like	0.0002	up
XM_043765830.1	LOC122593420	choline transporter protein 1	0.0005	up
XR_006324688.1	LOC122605701	uncharacterized LOC122605701, transcript variant X4	0.0034	up
XM_043778890.1	LOC122605927	phosphopantetheine adenylyltransferase-like, transcript variant X1	0.0003	up
XR_006323070.1	LOC122594695	small nucleolar RNA snoR2/U65	0.0057	up
XM_043772628.1	LOC122600000	uncharacterized protein At2g34160-like, transcript variant X2	0.0034	up
XM_043770688.1	LOC122598077	F-box protein PP2-A13-like	0.0032	up
XR_006324967.1	LOC122606629	pathogen-related protein-like, transcript variant X2	< 0.0001	up
XM_043752371.1	LOC122580097	F-box/kelch-repeat protein At3g06240-like	0.0006	up
XM_043772579.1	LOC122599957	THO complex subunit 7A-like	0.0042	up
XM_043761142.1	LOC122588921	GDSL lipase-like	< 0.0001	up
XM_043778449.1	LOC122605495	F-box/kelch-repeat protein At3g06240-like, transcript variant X2	0.0004	up
XM_043767365.1	LOC122595073	sister chromatid cohesion protein PDS5 homolog B-like, transcript variant X1	0.0009	up
XM_043779706.1	LOC122606833	peroxisome biogenesis protein 1, transcript variant X1	0.0008	up
XM_043778374.1	LOC122605423	transcription factor TCP20-like	0.0005	up
XM_043765729.1	LOC122593334	protein SUPPRESSOR OF GENE SILENCING 3-like	< 0.0001	up
XM_043757264.1	LOC122585157	1,4-dihydroxy-2-naphthoyl-CoA synthase, peroxisomal	< 0.0001	up
XM_043752680.1	LOC122580414	cytochrome P450 CYP82D47-like	0.0026	up
XM_043771758.1	LOC122599273	stress enhanced protein 1, chloroplastic	< 0.0001	up
XM_043765631.1	LOC122593245	charged multivesicular body protein 7, transcript variant X1	0.0018	up
XM_043772384.1	LOC122599799	probable enoyl-CoA hydratase 2, mitochondrial	0.0042	up
XM_043753759.1	LOC122581530	uncharacterized CRM domain-containing protein At3g25440, chloroplastic, transcript variant X1	0.0012	up
XM_043767053.1	LOC122594617	uncharacterized LOC122594617	< 0.0001	up
XM_043768660.1	LOC122596136	protein SUPPRESSOR OF GENE SILENCING 3-like	0.0015	up
XR_006323375.1	LOC122596764	SKP1-like protein 12, transcript variant X1	< 0.0001	up
XM_043773251.1	LOC122600520	(S)-coclaurine N-methyltransferase-like, transcript variant X2	0.0003	up
XM_043779546.1	LOC122606695	non-specific phospholipase C1, transcript variant X2	< 0.0001	up
XR_006321333.1	LOC122583843	uncharacterized LOC122583843	0.0026	up
XR_006321201.1	LOC122582593	uncharacterized LOC122582593, transcript variant X3	0.0004	up
XM_043778866.1	LOC122605907	phosphoenolpyruvate/phosphate translocator 2, chloroplastic-like	< 0.0001	up
XM_043776367.1	LOC122603616	protein GDAP2 homolog	< 0.0001	up

Transcript id	GeneID	Gene name/function	P Value	result
XM_043758612.1	LOC122586629	serine/threonine-protein phosphatase PP1 isozyme 3, transcript variant X1	0.0003	up
XM_043757174.1	LOC122585077	homeobox protein knotted-1-like 6	< 0.0001	up
XM_043768582.1	LOC122596070	uncharacterized LOC122596070	0.0002	up
XM_043759559.1	LOC122587400	cytochrome P450 CYP82D47-like	0.0038	up
XM_043750909.1	LOC122578860	cytochrome P450 93A3-like	< 0.0001	up
XR_006324596.1	LOC122605348	uncharacterized LOC122605348, transcript variant X3	< 0.0001	up
XM_043778157.1	LOC122605246	uncharacterized LOC122605246	0.0027	up
XM_043760963.1	LOC122588769	ABC transporter F family member 5-like, transcript variant X2	< 0.0001	up
XM_043779628.1	LOC122606771	bZIP transcription factor 23-like, transcript variant X1	0.0013	up
XM_043767336.1	LOC122595053	pentatricopeptide repeat-containing protein At1g63330-like, transcript variant X1	0.0013	up
XM_043764633.1	LOC122592415	DNA repair protein recA homolog 1, chloroplastic	0.0044	up
XM_043765284.1	LOC122592951	serine/threonine-protein phosphatase PP1 isozyme 2-like, transcript variant X2	< 0.0001	up
XM_043762081.1	LOC122589754	protein JOKA2-like	0.0028	up
XM_043777566.1	LOC122604689	acylamino-acid-releasing enzyme, transcript variant X3	0.0011	up
XM_043762014.1	LOC122589691	uncharacterized LOC122589691	0.0045	up
XM_043752854.1	LOC122580586	ferredoxin--NADP reductase, leaf-type isozyme, chloroplastic	< 0.0001	up
XM_043777786.1	LOC122604927	transcription factor MYB73-like	< 0.0001	up
XM_043761816.1	LOC122589517	heme-binding protein 2-like	< 0.0001	up
XM_043779976.1	LOC122607053	uncharacterized LOC122607053	< 0.0001	up
XM_043755207.1	LOC122582774	protein SRG1-like	0.0050	up
XM_043758437.1	LOC122586448	flavin mononucleotide hydrolase 1, chloroplatic, transcript variant X3	0.0051	up
XM_043782620.1	LOC122609666	uncharacterized protein At5g01610-like	< 0.0001	up
XM_043761083.1	LOC122588867	chaperone protein dnaJ 8, chloroplastic	< 0.0001	up
XM_043783000.1	LOC122610029	uncharacterized LOC122610029	0.0024	up
XM_043773659.1	LOC122600882	FAD-linked sulfhydryl oxidase ERV1	0.0027	up
XR_006324495.1	LOC122603735	uncharacterized LOC122603735, transcript variant X4	0.0058	up
XM_043750013.1	LOC122578125	probable serine protease EDA2	< 0.0001	up
XM_043755329.1	LOC122582901	putative pre-16S rRNA nuclease, transcript variant X1	0.0036	up
XM_043757815.1	LOC122585692	abscisic-aldehyde oxidase-like, transcript variant X3	0.0003	up
XR_006323643.1	LOC122598527	uncharacterized LOC122598527	0.0039	up
XM_043765919.1	LOC122593499	style cell-cycle inhibitor 1-A	0.0001	up
XM_043756591.1	LOC122584410	RNA polymerase sigma factor sigC	0.0001	up
XR_006325261.1	LOC122608573	uncharacterized LOC122608573	0.0008	up
XM_043755300.1	LOC122582866	calcium-transporting ATPase 12, plasma membrane-type-like	0.0003	up
XR_006320774.1	LOC122580341	uncharacterized LOC122580341, transcript variant X3	< 0.0001	up
XM_043759135.1	LOC122587063	uncharacterized LOC122587063	0.0005	up
XM_043774709.1	LOC122601976	mediator of RNA polymerase II transcription subunit 15a	0.0064	up
XM_043777664.1	LOC122604789	probable DNA double-strand break repair Rad50 ATPase, transcript variant X1	< 0.0001	up
XM_043762379.1	LOC122590041	urease accessory protein G	0.0003	up
XM_043752606.1	LOC122580335	pectate lyase-like	0.0011	up
XM_043760506.1	LOC122588400	uncharacterized LOC122588400, transcript variant X2	0.0039	up
XM_043761039.1	LOC122588828	alpha-1,3/1,6-mannosyltransferase ALG2-like	0.0005	up
XM_043770559.1	LOC122597965	UDP-glycosyltransferase 91C1	0.0003	up
XM_043767377.1	LOC122595080	2-oxoglutarate-dependent dioxygenase 19-like	< 0.0001	up
XM_043772052.1	LOC122599526	uncharacterized LOC122599526	0.0009	up
XM_043778125.1	LOC122605219	protein REVEILLE 2-like	< 0.0001	up
XR_006322140.1	LOC122587425	uncharacterized LOC122587425, transcript variant X2	0.0034	up
XM_043783039.1	LOC122610056	RNA cytosine-C(5)-methyltransferase NSUN2-like	0.0003	up
XM_043772286.1	LOC122599729	protein CHLOROPLAST IMPORT APPARATUS 2, transcript variant X2	0.0043	up
XM_043761206.1	LOC122588981	magnesium protoporphyrin IX methyltransferase, chloroplastic-like	< 0.0001	up
XM_043755324.1	LOC122582895	pentatricopeptide repeat-containing protein At3g59040-like	< 0.0001	up
XM_043775292.1	LOC122602702	uncharacterized LOC122602702, transcript variant X1	< 0.0001	up
XR_006321111.1	LOC122582141	uncharacterized LOC122582141, transcript variant X1	0.0007	up
XM_043779609.1	LOC122606751	heat shock 70 kDa protein 1-like	0.0055	up
XM_043751675.1	LOC122579496	zinc finger protein VAR3, chloroplastic-like	< 0.0001	up
XM_043768834.1	LOC122596286	50S ribosomal protein L6, chloroplastic	0.0022	up
XM_043768308.1	LOC122595850	F-actin-capping protein subunit alpha	< 0.0001	up
XM_043773718.1	LOC122600939	growth-regulating factor 6-like	< 0.0001	up
XM_043752776.1	LOC122580497	demethylepipodophyllotoxin synthase-like	< 0.0001	up
XM_043772261.1	LOC122599710	serine/threonine-protein kinase D6PKL2	< 0.0001	up
XR_006323718.1	LOC122598729	U1 spliceosomal RNA	0.0002	up

Transcript id	GeneID	Gene name/function	*P* Value	result
XM_043762429.1	LOC122590097	probable endo-1,3(4)-beta-glucanase ARB_01444	< 0.0001	up
XM_043778609.1	LOC122605651	probable serine/threonine-protein kinase PBL3	< 0.0001	up
XM_043766544.1	LOC122594068	probable dolichyl pyrophosphate Glc1Man9GlcNAc2 alpha-1,3-glucosyltransferase	0.0016	up
XM_043752219.1	LOC122579951	proline transporter 1-like	0.0036	up
XR_006322292.1	LOC122589613	uncharacterized LOC122589613	0.0004	up
XM_043772477.1	LOC122599876	protein SUPPRESSOR OF GENE SILENCING 3-like	0.0034	up
XM_043781366.1	LOC122608279	conserved oligomeric Golgi complex subunit 3, transcript variant X1	0.0032	up
XM_043780389.1	LOC122607387	TMV resistance protein N-like	0.0011	up
XM_043774145.1	LOC122601374	aminoacylase-1, transcript variant X1	0.0011	up
XM_043761863.1	LOC122589562	pentatricopeptide repeat-containing protein At3g09040, mitochondrial, transcript variant X2	0.0007	up
XM_043757625.1	LOC122585497	norbelladine synthase-like	< 0.0001	up
XM_043771604.1	LOC122599147	cyclase-like protein 2	< 0.0001	up
XM_043752423.1	LOC122580153	putative uncharacterized protein DDB_G0282129	0.0012	up
XM_043756232.1	LOC122583858	protein FRA10AC1	0.0001	up
XM_043750766.1	LOC122578740	cytoplasmic tRNA 2-thiolation protein 1	< 0.0001	up
XM_043775460.1	LOC122602853	protein CHROMATIN REMODELING 4-like, transcript variant X3	0.0002	up
XM_043780332.1	LOC122607367	endoribonuclease YBEY, chloroplastic	0.0001	up
XM_043753722.1	LOC122581491	DNA-3-methyladenine glycosylase-like	0.0026	up
XR_006323748.1	LOC122598821	uncharacterized LOC122598821, transcript variant X8	0.0027	up
XM_043774441.1	LOC122601697	uncharacterized LOC122601697	0.0003	up
XM_043758531.1	LOC122586542	probable LRR receptor-like serine/threonine-protein kinase At3g47570	0.0019	up
XM_043753161.1	LOC122581017	uncharacterized LOC122581017, transcript variant X1	0.0049	up
XM_043767302.1	LOC122595022	chlorophyll a-b binding protein CP24 10B, chloroplastic-like	< 0.0001	up
XM_043778155.1	LOC122605245	probable inactive serine/threonine-protein kinase bub1	0.0006	up
XM_043776106.1	LOC122603414	ATP-dependent DNA helicase At3g02060, chloroplastic	0.0043	up
XM_043779812.1	LOC122606920	transcription termination factor MTERF4, chloroplastic, transcript variant X1	0.0020	up
XM_043779286.1	LOC122606331	protein trichome birefringence-like 39	< 0.0001	up
XM_043772971.1	LOC122600271	protein KAKU4-like, transcript variant X2	0.0002	up
XM_043781372.1	LOC122608283	cytochrome P450 93A3-like	< 0.0001	up
XR_006325460.1	LOC122610870	uncharacterized LOC122610870, transcript variant X2	0.0003	up
XM_043751279.1	LOC122579167	triadin-like	0.0011	up
XM_043776883.1	LOC122604003	cold-responsive protein kinase 1-like, transcript variant X2	0.0031	up
XM_043751205.1	LOC122579105	protein OVEREXPRESSOR OF CATIONIC PEROXIDASE 3, transcript variant X2	< 0.0001	up
XM_043778462.1	LOC122605510	uncharacterized LOC122605510, transcript variant X2	< 0.0001	up
XM_043774666.1	LOC122601937	probable protein phosphatase 2C 21	< 0.0001	up
XM_043767734.1	LOC122595382	subtilisin-like protease SBT2.6, transcript variant X1	< 0.0001	up
XM_043760754.1	LOC122588605	aspartic proteinase-like protein 1	0.0021	up
XM_043769544.1	LOC122596900	G-type lectin S-receptor-like serine/threonine-protein kinase At4g27290	0.0017	up
XM_043752304.1	LOC122580036	probable GTP-binding protein OBGC2, transcript variant X2	0.0027	up
XM_043754157.1	LOC122581850	trihelix transcription factor ASIL2-like	0.0003	up
XM_043753535.1	LOC122581322	F-box/kelch-repeat protein At2g44130-like	< 0.0001	up
XM_043750577.1	LOC122578592	uncharacterized LOC122578592	0.0003	up
XM_043769041.1	LOC122596460	protein trichome birefringence-like 5	0.0003	up
XM_043755246.1	LOC122582811	kinesin-like protein KIN-5D	0.0003	up
XM_043765661.1	LOC122593273	guanine nucleotide-binding protein subunit beta-like, transcript variant X6	< 0.0001	up
XM_043766511.1	LOC122594033	scopoletin 8-hydroxylase, transcript variant X1	0.0044	up
XM_043751336.1	LOC122579217	uncharacterized LOC122579217	< 0.0001	up
XM_043765906.1	LOC122593488	COBRA-like protein 10	0.0001	up
XM_043778612.1	LOC122605657	putative receptor-like protein kinase At3g47110	< 0.0001	up
XM_043760315.1	LOC122588191	uncharacterized LOC122588191	0.0008	up
XM_043772798.1	LOC122600133	cytochrome P450 CYP72A219-like	0.0008	up
XM_043761386.1	LOC122589144	uncharacterized LOC122589144, transcript variant X3	0.0036	up
XM_043782963.1	LOC122609995	cytokinin riboside 5'-monophosphate phosphoribohydrolase LOG8	0.0005	up
XM_043773648.1	LOC122600870	putative pre-16S rRNA nuclease	0.0026	up
XM_043780224.1	LOC122607284	WD repeat-containing protein 43	0.0044	up
XM_043784322.1	LOC122611313	serine/threonine-protein kinase SAPK2	< 0.0001	up
XM_043761047.1	LOC122588837	F-box/kelch-repeat protein At3g06240-like	0.0021	up
XM_043776239.1	LOC122603514	probable methionine--tRNA ligase, transcript variant X2	< 0.0001	up
XM_043759423.1	LOC122587297	protein TSS, transcript variant X2	0.0056	up

Transcript id	GeneID	Gene name/function	*P* Value	result
XM_043773536.1	LOC122600777	2-C-methyl-D-erythritol 2,4-cyclodiphosphate synthase, chloroplastic	0.0006	up
XM_043762338.1	LOC122590002	amino acid transporter AVT1I-like	0.0038	up
XM_043756537.1	LOC122584335	uncharacterized LOC122584335	0.0052	up
XM_043761113.1	LOC122588893	ACT domain-containing protein ACR6-like	< 0.0001	up
XM_043775795.1	LOC122603164	protein PAM71-homolog, chloroplastic, transcript variant X1	0.0047	up
XM_043754826.1	LOC122582437	glycosyltransferase-like KOBITO 1	0.0007	up
XM_043764896.1	LOC122592620	leucine-rich repeat protein 2-like	< 0.0001	up
XR_006322175.1	LOC122588679	uncharacterized LOC122588679	0.0020	up
XM_043767819.1	LOC122595456	beta-galactosidase 1-like	< 0.0001	up
XM_043750170.1	LOC122578254	WD repeat-containing protein DDB_G0290555	0.0027	up
XM_043770046.1	LOC122597455	uncharacterized LOC122597455	< 0.0001	up
XR_006322790.1	LOC122593060	stromal 70 kDa heat shock-related protein, chloroplastic-like, transcript variant X4	0.0044	up
XM_043765551.1	LOC122593182	FCS-Like Zinc finger 8-like	< 0.0001	up
XM_043756353.1	LOC122583984	putative receptor-like protein kinase At5g39000, transcript variant X2	< 0.0001	up
XM_043763717.1	LOC122591448	protein DETOXIFICATION 17-like	0.0058	up
XM_043776012.1	LOC122603338	acyl-CoA-binding domain-containing protein 4-like	0.0033	up
XM_043773288.1	LOC122600553	FBD-associated F-box protein At4g10400-like	0.0023	up
XM_043776457.1	LOC122603684	germacrene A synthase short form-like	< 0.0001	up
XR_006323573.1	LOC122598276	uncharacterized LOC122598276, transcript variant X5	< 0.0001	up
XM_043779721.1	LOC122606843	putative uncharacterized protein DDB_G0286901	0.0021	up
XM_043763233.1	LOC122591043	ferritin, chloroplastic-like	< 0.0001	up
XM_043753761.1	LOC122581531	peroxisomal nicotinamide adenine dinucleotide carrier-like	0.0015	up
XM_043763483.1	LOC122591249	putative beta-glucosidase 41	< 0.0001	up
XM_043751740.1	LOC122579551	protein NRT1/ PTR FAMILY 8.3-like	0.0023	up
XR_006324970.1	LOC122606661	uncharacterized LOC122606661	0.0017	up
XM_043775507.1	LOC122602896	cytochrome P450 CYP72A219-like	0.0013	up
XM_043776794.1	LOC122603935	DNA excision repair protein ERCC-1, transcript variant X2	0.0006	up
XM_043750919.1	LOC122578870	isoaspartyl peptidase/L-asparaginase	0.0006	up
XM_043767784.1	LOC122595427	glucan endo-1,3-beta-glucosidase 12	0.0006	up
XM_043759241.1	LOC122587152	transcription termination factor MTERF5, chloroplastic	0.0059	up
XM_043779081.1	LOC122606116	cyclin-dependent kinase F-4-like	< 0.0001	up
XM_043768831.1	LOC122596282	ferredoxin-thioredoxin reductase, variable chain-like	0.0012	up
XM_043760140.1	LOC122587992	putative UPF0481 protein At3g02645	0.0018	up
XM_043767863.1	LOC122595496	multiple organellar RNA editing factor 8, chloroplastic/mitochondrial-like	0.0008	up
XM_043772206.1	LOC122599661	homogentisate 1,2-dioxygenase	0.0063	up
XM_043772105.1	LOC122599577	beta carbonic anhydrase 5, chloroplastic, transcript variant X2	0.0015	up
XM_043781297.1	LOC122608210	glutaredoxin-C11-like	< 0.0001	up
XM_043770766.1	LOC122598166	protein XRI1-like	0.0052	up
XM_043750530.1	LOC122578550	adenylate kinase 4-like, transcript variant X2	0.0032	up
XM_043760808.1	LOC122588652	J domain-containing protein required for chloroplast accumulation response 1	0.0013	up
XM_043755115.1	LOC122582689	protein PLASTID TRANSCRIPTIONALLY ACTIVE 16, chloroplastic	0.0006	up
XM_043772802.1	LOC122600138	trigger factor-like protein TIG, Chloroplastic	< 0.0001	up
XM_043768909.1	LOC122596346	tubulin-folding cofactor A, transcript variant X1	0.0004	up
XM_043768814.1	LOC122596260	receptor-like protein 46	0.0041	up
XM_043768609.1	LOC122596091	uncharacterized LOC122596091, transcript variant X2	< 0.0001	up
XM_043780505.1	LOC122607516	FK506-binding protein 2-like	< 0.0001	up
XM_043752227.1	LOC122579960	root allergen protein-like	< 0.0001	up
XM_043753950.1	LOC122581696	homocysteine S-methyltransferase 2-like	0.0043	up
XM_043778090.1	LOC122605190	glutamate decarboxylase 5-like	0.0026	up
XM_043778848.1	LOC122605891	acyl-CoA C20 Delta5-desaturase-like	< 0.0001	up
XM_043773642.1	LOC122600864	fructose-1,6-bisphosphatase class 1	0.0006	up
XM_043761549.1	LOC122589276	pyridoxal kinase	0.0004	up
XR_006324696.1	LOC122605739	uncharacterized LOC122605739	0.0008	up
XR_006325362.1	LOC122609662	uncharacterized LOC122609662	< 0.0001	up
XM_043763746.1	LOC122591481	protein TSS-like	< 0.0001	up
XM_043771480.1	LOC122599035	probable plastid-lipid-associated protein 4, chloroplastic	0.0019	up
XR_006323972.1	LOC122599969	uncharacterized LOC122599969	0.0022	up
XM_043750628.1	LOC122578630	peptidyl-prolyl cis-trans isomerase CYP38, chloroplastic	< 0.0001	up
XM_043763244.1	LOC122591054	2-oxoglutarate-Fe(II) type oxidoreductase hxnY-like, transcript variant X2	0.0002	up
XM_043762808.1	LOC122590626	uncharacterized LOC122590626	0.0048	up
XM_043760629.1	LOC122588497	RNA pseudouridine synthase 2, chloroplastic	0.0019	up

Transcript id	GeneID	Gene name/function	*P* Value	result
XM_043779443.1	LOC122606600	uncharacterized LOC122606600, transcript variant X1	0.0041	up
XM_043763348.1	LOC122591134	callose synthase 11-like	0.0019	up
XM_043781238.1	LOC122608146	uncharacterized LOC122608146	0.0014	up
XM_043767198.1	LOC122594911	(+)-neomenthol dehydrogenase-like	0.0014	up
XM_043780212.1	LOC122607276	co-chaperone protein p23-1-like	0.0003	up
XM_043753514.1	LOC122581309	uncharacterized LOC122581309, transcript variant X5	< 0.0001	up
XR_006323462.1	LOC122597806	uncharacterized LOC122597806	0.0004	up
XM_043758268.1	LOC122586158	formin-like protein 3, transcript variant X2	0.0028	up
XM_043772165.1	LOC122599627	uncharacterized LOC122599627	0.0011	up
XM_043781063.1	LOC122607981	protein translocase subunit SECA1, chloroplastic	< 0.0001	up
XM_043772434.1	LOC122599846	ferric reduction oxidase 7, chloroplastic-like, transcript variant X1	0.0041	up
XM_043776286.1	LOC122603558	scarecrow-like protein 3	0.0057	up
XM_043770040.1	LOC122597448	uncharacterized LOC122597448	0.0020	up
XM_043777099.1	LOC122604205	uncharacterized LOC122604205	0.0003	up
XM_043770738.1	LOC122598136	heme-binding protein 2	< 0.0001	up
XM_043767208.1	LOC122594924	putative transferase At4g12130, mitochondrial, transcript variant X4	< 0.0001	up
XM_043754233.1	LOC122581915	serine/threonine-protein kinase rio2	< 0.0001	up
XM_043767945.1	LOC122595563	uncharacterized LOC122595563	0.0030	up
XM_043769974.1	LOC122597375	uncharacterized LOC122597375	< 0.0001	up
XM_043777031.1	LOC122604132	thaumatin-like protein 1	0.0003	up
XM_043751311.1	LOC122579198	lichenase-like	< 0.0001	up
XR_006325495.1	LOC122611253	protein GDAP2 homolog, transcript variant X4	< 0.0001	up
XM_043778854.1	LOC122605898	glycerophosphodiester phosphodiesterase GDPD1, chloroplastic	0.0019	up
XM_043761522.1	LOC122589255	uncharacterized protein DDB_G0283357	0.0038	up
XM_043762575.1	LOC122590245	LOB domain-containing protein 39-like	0.0035	up
XM_043761002.1	LOC122588796	40S ribosomal protein S25	0.0009	up
XM_043776759.1	LOC122603906	ethylene-responsive transcription factor ERF104-like	< 0.0001	up
XM_043780553.1	LOC122607554	uncharacterized LOC122607554, transcript variant X1	0.0032	up
XM_043761994.1	LOC122589676	E3 ubiquitin-protein ligase SDIR1, transcript variant X7	0.0001	up
XM_043767168.1	LOC122594867	replication factor C subunit 1	0.0016	up
XM_043776565.1	LOC122603766	uncharacterized LOC122603766	0.0003	up
XR_006324961.1	LOC122606614	uncharacterized LOC122606614	0.0012	up
XM_043766048.1	LOC122593623	uncharacterized LOC122593623	0.0017	up
XM_043762023.1	LOC122589702	HIPL1 protein-like	0.0012	up
XM_043772789.1	LOC122600124	uncharacterized LOC122600124	0.0028	up
XM_043771586.1	LOC122599129	uncharacterized LOC122599129, transcript variant X2	< 0.0001	up
XM_043759199.1	LOC122587118	arabinosyltransferase RRA3-like	0.0046	up
XM_043751746.1	LOC122579557	serine carboxypeptidase-like 42	< 0.0001	up
XM_043764565.1	LOC122592357	transcription factor bHLH112-like, transcript variant X2	< 0.0001	up
XM_043775094.1	LOC122602405	elongation factor G, chloroplastic	< 0.0001	up
XM_043779199.1	LOC122606234	uncharacterized LOC122606234	< 0.0001	up
XM_043781084.1	LOC122608004	uncharacterized LOC122608004	< 0.0001	up
XM_043777930.1	LOC122605056	non-symbiotic hemoglobin 2-like	< 0.0001	up
XM_043756277.1	LOC122583905	50S ribosomal protein L18-like	< 0.0001	up
XM_043768174.1	LOC122595744	BOI-related E3 ubiquitin-protein ligase 1-like	0.0001	up
XM_043759493.1	LOC122587354	probable enoyl-CoA hydratase 1, peroxisomal, transcript variant X1	0.0057	up
XM_043770613.1	LOC122598008	uncharacterized LOC122598008	0.0031	up
XM_043758476.1	LOC122586493	protein SUPPRESSOR OF QUENCHING 1, chloroplastic	< 0.0001	up
XM_043769340.1	LOC122596716	50S ribosomal protein L18-like	< 0.0001	up
XM_043779606.1	LOC122606747	60S ribosomal protein L32-1-like	0.0047	up
XM_043770246.1	LOC122597673	DNA-directed RNA polymerase I subunit rpa49-like	0.0006	up
XM_043756557.1	LOC122584369	photosystem I assembly protein Ycf4	0.0034	up
XM_043759598.1	LOC122587432	inositol oxygenase 1-like	< 0.0001	up
XM_043770289.1	LOC122597724	DNA-directed RNA polymerases II and IV subunit 5A-like	< 0.0001	up
XM_043761709.1	LOC122589415	mitochondrial arginine transporter BAC2	0.0001	up
XM_043751160.1	LOC122579068	mechanosensitive ion channel protein 6-like	< 0.0001	up
XM_043763193.1	LOC122591008	polyadenylate-binding protein RBP47-like, transcript variant X3	0.0030	up
XM_043751148.1	LOC122579060	uncharacterized LOC122579060	0.0056	up
XR_006325115.1	LOC122607840	uncharacterized LOC122607840, transcript variant X3	0.0003	up
XM_043756961.1	LOC122584886	cytochrome P450 76C1-like	< 0.0001	up
XM_043760530.1	LOC122588420	nephrocystin-3	0.0022	up
XM_043759083.1	LOC122587024	uncharacterized LOC122587024	0.0042	up
XM_043781624.1	LOC122608519	uncharacterized LOC122608519	< 0.0001	up
XM_043750536.1	LOC122578556	PLASMODESMATA CALLOSE-BINDING PROTEIN 4-like	0.0014	up

Transcript id	GeneID	Gene name/function	*P* Value	result
XM_043753359.1	LOC122581189	putative GTP diphosphokinase RSH1, chloroplastic, transcript variant X1	< 0.0001	up
XM_043774481.1	LOC122601741	acyltransferase-like protein At1g54570, chloroplastic	0.0023	up
XM_043756493.1	LOC122584281	photosystem II protein D1	< 0.0001	up
XM_043777909.1	LOC122605039	chaperone protein dnaJ 11, chloroplastic-like	0.0009	up
XM_043750558.1	LOC122578574	thaumatin-like protein	< 0.0001	up
XM_043772852.1	LOC122600182	phosphopantothenoylcysteine decarboxylase subunit VHS3-like	< 0.0001	up
XM_043783055.1	LOC122610073	protein NSP-INTERACTING KINASE 3-like, transcript variant X1	< 0.0001	up
XM_043762159.1	LOC122589839	guanosine deaminase-like	< 0.0001	up
XM_043771283.1	LOC122598760	calcium-binding protein 39	0.0033	up
XM_043757827.1	LOC122585703	protein EMBRYONIC FLOWER 1, transcript variant X2	0.0004	up
XM_043755213.1	LOC122582778	oleoyl-acyl carrier protein thioesterase, chloroplastic-like	< 0.0001	up
XM_043755234.1	LOC122582797	gamma-interferon-responsive lysosomal thiol protein-like	< 0.0001	up
XM_043768583.1	LOC122596071	DExH-box ATP-dependent RNA helicase DExH16, mitochondrial, transcript variant X2	0.0017	up
XM_043750072.1	LOC122578169	ATP-citrate synthase alpha chain protein 1-like	< 0.0001	up
XM_043750895.1	LOC122578846	uncharacterized LOC122578846, transcript variant X1	0.0005	up
XM_043767853.1	LOC122595487	tropinone reductase homolog At5g06060-like	0.0056	up
XM_043751085.1	LOC122579006	peptidyl-prolyl cis-trans isomerase CYP21-1-like	0.0018	up
XM_043769977.1	LOC122597377	MYB-like transcription factor EOBII	0.0020	up
XM_043755634.1	LOC122583208	chitin elicitor receptor kinase 1-like	< 0.0001	up
XM_043763344.1	LOC122591129	probable LRR receptor-like serine/threonine-protein kinase RFK1, transcript variant X1	0.0041	up
XM_043771521.1	LOC122599070	inactive beta-amylase 9	< 0.0001	up
XR_006324084.1	LOC122600711	uncharacterized LOC122600711, transcript variant X2	0.0003	up
XM_043762000.1	LOC122589677	U-box domain-containing protein 33-like, transcript variant X1	< 0.0001	up
XM_043753541.1	LOC122581328	2-alkenal reductase (NADP(+)-dependent)-like	< 0.0001	up
XM_043762175.1	LOC122589856	PHD finger protein EHD3	0.0014	up
XM_043758406.1	LOC122586410	uncharacterized protein At4g06744-like	< 0.0001	up
XR_006320558.1	LOC122578878	uncharacterized LOC122578878	0.0014	up
XM_043751948.1	LOC122579724	LMBR1 domain-containing protein 2 homolog	< 0.0001	up
XM_043767727.1	LOC122595376	uncharacterized LOC122595376	< 0.0001	up
XM_043767147.1	LOC122594840	uncharacterized LOC122594840, transcript variant X2	0.0005	up
XM_043774901.1	LOC122602185	peroxisomal (S)-2-hydroxy-acid oxidase-like	< 0.0001	up
XR_006321252.1	LOC122582842	uncharacterized LOC122582842	0.0009	up
XM_043751907.1	LOC122579684	DEAD-box ATP-dependent RNA helicase 32-like	0.0004	up
XM_043751816.1	LOC122579613	uncharacterized LOC122579613, transcript variant X1	< 0.0001	up
XR_006321121.1	LOC122582147	uncharacterized LOC122582147	0.0004	up
XM_043762794.1	LOC122590607	E3 ubiquitin-protein ligase RBBP6	0.0017	up
XM_043759589.1	LOC122587426	uncharacterized membrane protein At4g09580-like	0.0057	up
XM_043779532.1	LOC122606686	beta-glucosidase 13-like	< 0.0001	up
XM_043770830.1	LOC122598226	heavy metal-associated isoprenylated plant protein 45-like, transcript variant X1	0.0006	up
XM_043777700.1	LOC122604825	alpha-mannosidase I MNS4, transcript variant X1	< 0.0001	up
XM_043753073.1	LOC122580929	ubiquitin C-terminal hydrolase 12-like, transcript variant X2	0.0022	up
XM_043751393.1	LOC122579255	defensin SD2-like, transcript variant X2	0.0059	up
XR_006322262.1	LOC122589411	uncharacterized LOC122589411	0.0051	up
XM_043750053.1	LOC122578155	erlin-2-B	0.0011	up
XM_043762550.1	LOC122590215	probable dolichyl pyrophosphate Man9GlcNAc2 alpha-1,3-glucosyltransferase, transcript variant X1	0.0059	up
XR_006320886.1	LOC122580823	uncharacterized LOC122580823, transcript variant X2	0.0007	up
XM_043751170.1	LOC122579079	chlorophyll a-b binding protein 13, chloroplastic-like	< 0.0001	up
XM_043784131.1	LOC122611155	carotenoid 9,10(9',10')-cleavage dioxygenase 1-like	0.0039	up
XM_043776316.1	LOC122603578	uncharacterized LOC122603578, transcript variant X1	0.0020	up
XM_043763361.1	LOC122591145	uncharacterized protein At1g10890, transcript variant X1	0.0003	up
XM_043775500.1	LOC122602890	uncharacterized LOC122602890, transcript variant X1	< 0.0001	up
XM_043756235.1	LOC122583857	protein LIGHT-DEPENDENT SHORT HYPOCOTYLS 1-like	0.0033	up
XM_043752509.1	LOC122580231	flowering-promoting factor 1-like protein 3	0.0006	up
XM_043752168.1	LOC122579908	malate dehydrogenase, glyoxysomal-like	0.0011	up
XM_043760941.1	LOC122588753	PITH domain-containing protein At3g04780	0.0033	up
XM_043759668.1	LOC122587482	60S ribosomal protein L7-1	0.0037	up
XR_006323166.1	LOC122594965	uncharacterized LOC122594965, transcript variant X2	0.0003	up
XM_043775262.1	LOC122602665	cytochrome P450 CYP72A219-like	0.0012	up
XM_043768421.1	LOC122595940	beta-galactosidase	0.0055	up
XM_043767314.1	LOC122595033	protein Iojap, chloroplastic	0.0002	up
XM_043766964.1	LOC122594517	probable helicase MAGATAMA 3	0.0006	up

Transcript id	GeneID	Gene name/function	P Value	result
XM_043755057.1	LOC122582637	succinate dehydrogenase assembly factor 1, mitochondrial	0.0030	up
XM_043780961.1	LOC122607887	zinc transporter ZTP29	0.0026	up
XM_043776336.1	LOC122603590	protein IQ-DOMAIN 31-like, transcript variant X1	0.0004	up
XM_043756680.1	LOC122584629	30S ribosomal protein S4, chloroplastic	0.0044	up
XM_043774856.1	LOC122602130	1-aminocyclopropane-1-carboxylate oxidase 1	0.0048	up
XM_043755162.1	LOC122582739	uncharacterized LOC122582739	< 0.0001	up
XM_043764531.1	LOC122592330	putative vacuolar protein sorting-associated protein 13D	< 0.0001	up
XM_043759290.1	LOC122587189	phosphoglycolate phosphatase 1A, chloroplastic-like	0.0004	up
XM_043753118.1	LOC122580973	glycosyl hydrolase 5 family protein-like	0.0002	up
XR_006322471.1	LOC122590331	U12 minor spliceosomal RNA	< 0.0001	up
XM_043767995.1	LOC122595600	uncharacterized LOC122595600, transcript variant X3	< 0.0001	up
XM_043756224.1	LOC122583847	serine carboxypeptidase-like 45	0.0049	up
XM_043779376.1	LOC122606517	two-component response regulator-like APRR1	< 0.0001	up
XM_043772583.1	LOC122599962	probable inactive purple acid phosphatase 28	0.0008	up
XM_043755759.1	LOC122583339	protein NRT1/ PTR FAMILY 6.3-like	0.0012	up
XM_043776363.1	LOC122603611	uncharacterized protein At3g27210-like	< 0.0001	up
XM_043773123.1	LOC122600411	rop guanine nucleotide exchange factor 14	0.0028	up
XM_043770951.1	LOC122598358	protein STICHEL-like 4	< 0.0001	up
XM_043779551.1	LOC122606697	peptide chain release factor PrfB1, chloroplastic-like	0.0029	up
XM_043773605.1	LOC122600831	mannosyltransferase APTG1, transcript variant X2	0.0050	up
XM_043755850.1	LOC122583448	toll/interleukin-1 receptor-like protein	< 0.0001	up
XM_043766488.1	LOC122594013	disease resistance protein At4g27190-like	< 0.0001	up
XM_043781166.1	LOC122608079	protein DELETION OF SUV3 SUPPRESSOR 1(I)-like, transcript variant X2	< 0.0001	up
XM_043762567.1	LOC122590236	oxygen-evolving enhancer protein 1, chloroplastic	< 0.0001	up
XM_043750622.1	LOC122578625	uncharacterized LOC122578625	0.0057	up
XM_043758127.1	LOC122586011	uncharacterized protein At1g28695-like	0.0009	up
XM_043750897.1	LOC122578849	protein RGF1 INDUCIBLE TRANSCRIPTION FACTOR 1-like	0.0049	up
XM_043777889.1	LOC122605022	ribulose-1,5 bisphosphate carboxylase/oxygenase large subunit N-methyltransferase, chloroplastic	0.0002	up
XM_043783389.1	LOC122610397	uncharacterized LOC122610397	< 0.0001	up
XM_043768211.1	LOC122595775	proline iminopeptidase, transcript variant X1	0.0005	up
XM_043755128.1	LOC122582702	calcium-dependent protein kinase SK5-like	0.0001	up
XM_043750112.1	LOC122578204	phosphatase IMPL1, chloroplastic	0.0038	up
XM_043780362.1	LOC122607396	zinc finger protein CONSTANS-LIKE 13	0.0008	up
XM_043778419.1	LOC122605463	plastidial pyruvate kinase 4, chloroplastic	0.0015	up
XR_006324309.1	LOC122602548	uncharacterized LOC122602548, transcript variant X1	0.0051	up
XM_043770663.1	LOC122598053	protein NONRESPONDING TO OXYLIPINS 2, mitochondrial, transcript variant X2	0.0060	up
XM_043765332.1	LOC122593000	protein TSS	< 0.0001	up
XM_043752942.1	LOC122580675	heat shock 70 kDa protein II-like, transcript variant X1	< 0.0001	up
XM_043775933.1	LOC122603266	phosphoglucan phosphatase LSF1, chloroplastic	0.0003	up
XR_006322906.1	LOC122593970	uncharacterized LOC122593970	0.0002	up
XM_043766942.1	LOC122594493	ethylene-responsive transcription factor 13-like	< 0.0001	up
XM_043754145.1	LOC122581837	tRNA dimethylallyltransferase 2	< 0.0001	up
XR_006323329.1	LOC122596486	uncharacterized LOC122596486, transcript variant X5	0.0002	up
XM_043776417.1	LOC122603648	pentatricopeptide repeat-containing protein At4g01400, mitochondrial-like	0.0004	up
XM_043766489.1	LOC122594014	probable disease resistance protein At4g27220	< 0.0001	up
XM_043781167.1	LOC122608080	phosphatidylinositol transfer protein 3	0.0032	up
XM_043774913.1	LOC122602200	glu S.griseus protease inhibitor-like	0.0003	up
XM_043770941.1	LOC122598012	coniferyl alcohol acyltransferase-like	0.0008	up
XM_043762779.1	LOC122590590	photosystem I chlorophyll a/b-binding protein 3-1, chloroplastic-like	0.0025	up
XM_043770718.1	LOC122598117	glutathione hydrolase 3-like	< 0.0001	up
XM_043766055.1	LOC122593626	lysine-specific demethylase JMJ25-like	0.0039	up
XM_043779845.1	LOC122606945	probable transmembrane GTPase FZO-like, chloroplastic	< 0.0001	up
XM_043765086.1	LOC122592776	uncharacterized LOC122592776, transcript variant X2	0.0011	up
XM_043767926.1	LOC122595547	alpha-N-acetylglucosaminidase, transcript variant X1	0.0009	up
XM_043756945.1	LOC122584873	lysM domain-containing GPI-anchored protein 2-like	0.0019	up
XM_043773297.1	LOC122600560	(E)-beta-farnesene synthase-like	< 0.0001	up
XM_043779179.1	LOC122606219	coiled-coil domain-containing protein 12-like, transcript variant X4	0.0013	up
XM_043781519.1	LOC122608413	uncharacterized LOC122608413	0.0055	up
XM_043752238.1	LOC122579972	mitochondrial import receptor subunit TOM20-like	0.0042	up
XR_006320841.1	LOC122580693	U5 spliceosomal RNA	< 0.0001	up
XM_043764511.1	LOC122592308	G-type lectin S-receptor-like serine/threonine-protein kinase At4g27290	< 0.0001	up

Transcript id	GeneID	Gene name/function	*P* Value	result
XM_043765867.1	LOC122593455	protein KINESIN LIGHT CHAIN-RELATED 3, transcript variant X2	< 0.0001	up
XM_043782545.1	LOC122609505	receptor-like protein 34	0.0051	up
XM_043755225.1	LOC122582787	electron transfer flavoprotein-ubiquinone oxidoreductase, mitochondrial	< 0.0001	up
XM_043772721.1	LOC122600065	peptide-N4-(N-acetyl-beta-glucosaminyl)asparagine amidase A	0.0050	up
XM_043760614.1	LOC122588486	histidine biosynthesis bifunctional protein hisIE, chloroplastic, transcript variant X2	0.0001	up
XM_043771780.1	LOC122599290	cyclic nucleotide-gated ion channel 2	< 0.0001	up
XM_043766491.1	LOC122594016	uncharacterized LOC122594016, transcript variant X1	< 0.0001	up
XM_043753986.1	LOC122581722	1-(5-phosphoribosyl)-5-[(5-phosphoribosylamino)methylideneamino] imidazole-4-carboxamide isomerase, chloroplastic	< 0.0001	up
XM_043784158.1	LOC122611181	uncharacterized LOC122611181	0.0003	up
XM_043771362.1	LOC122598882	repetitive proline-rich cell wall protein 3-like	< 0.0001	up
XM_043758514.1	LOC122586530	serine hydroxymethyltransferase 1, mitochondrial	0.0002	up
XM_043761322.1	LOC122589078	uncharacterized LOC122589078	0.0016	up
XM_043767410.1	LOC122595100	sec14 cytosolic factor-like	< 0.0001	up
XM_043753903.1	LOC122581657	uncharacterized protein At5g03900, chloroplastic-like	0.0029	up
XM_043767405.1	LOC122595098	alpha-glucan water dikinase 1, chloroplastic-like, transcript variant X6	< 0.0001	up
XM_043765040.1	LOC122592746	haloacid dehalogenase-like hydrolase domain-containing protein 3, transcript variant X1	0.0003	up
XR_006322822.1	LOC122593321	uncharacterized LOC122593321, transcript variant X1	< 0.0001	up
XM_043784323.1	LOC122611314	vacuolar sorting protein 18	0.0022	up
XM_043781558.1	LOC122608449	40S ribosomal protein S24-1-like	0.0002	up
XR_006323461.1	LOC122597803	PI-PLC X domain-containing protein At5g67130-like, transcript variant X2	0.0047	up
XR_006322905.1	LOC122593964	E3 ubiquitin-protein ligase AIRP2-like, transcript variant X2	0.0008	up
XM_043764878.1	LOC122592603	protein unc-13 homolog	0.0011	up
XM_043761218.1	LOC122588991	beta-glucosidase 42	0.0013	up
XM_043781607.1	LOC122608501	gibberellin 20 oxidase 2-like	0.0006	up
XM_043760565.1	LOC122588448	protein SET DOMAIN GROUP 41, transcript variant X2	0.0046	up
XM_043770376.1	LOC122597801	serine--tRNA ligase	< 0.0001	up
XM_043780963.1	LOC122607891	phosphatidate phosphatase PAH2-like	0.0009	up
XM_043758968.1	LOC122586935	dihydropyrimidine dehydrogenase (NADP(+)), chloroplastic	0.0029	up
XM_043750291.1	LOC122578346	probable inactive ATP-dependent zinc metalloprotease FTSHI 5, chloroplastic	0.0017	up
XM_043763736.1	LOC122591468	uncharacterized LOC122591468	0.0045	up
XM_043755366.1	LOC122582935	uncharacterized protein At2g34460, chloroplastic, transcript variant X1	0.0002	up
XM_043779420.1	LOC122606579	calmodulin-7	0.0018	up
XM_043773019.1	LOC122600311	NHP2-like protein 1, transcript variant X2	0.0059	up
XM_043756442.1	LOC122584224	uncharacterized LOC122584224	0.0063	up
XR_006322473.1	LOC122590333	U5 spliceosomal RNA	0.0048	up
XM_043767588.1	LOC122595252	pentatricopeptide repeat-containing protein At1g02150	0.0001	up
XM_043758837.1	LOC122586839	putative receptor-like protein kinase At3g47110	0.0008	up
XM_043780849.1	LOC122607800	uncharacterized LOC122607800	0.0064	up
XM_043756258.1	LOC122583886	putative disease resistance RPP13-like protein 1	0.0047	up
XM_043750475.1	LOC122578502	phosphatidylinositol 4-phosphate 5-kinase 2-like	0.0025	up
XM_043773340.1	LOC122600594	WD repeat-containing protein 43, transcript variant X3	0.0034	up
XM_043769135.1	LOC122596537	transmembrane protein 208 homolog	< 0.0001	up
XM_043773690.1	LOC122600913	root allergen protein-like	0.0013	up
XM_043751640.1	LOC122579457	CRM-domain containing factor CFM9, mitochondrial	0.0036	up
XM_043750649.1	LOC122578643	uncharacterized LOC122578643	0.0028	up
XM_043775835.1	LOC122603194	BTB/POZ domain-containing protein NPY4-like, transcript variant X1	< 0.0001	up
XM_043754654.1	LOC122582284	uncharacterized LOC122582284	0.0004	up
XM_043761696.1	LOC122589403	zinc finger protein ZAT9-like	0.0002	up
XM_043756814.1	LOC122584759	mitoferrin, transcript variant X2	0.0003	up
XM_043775203.1	LOC122602604	probable E3 ubiquitin-protein ligase RHG1A, transcript variant X1	< 0.0001	up
XM_043762703.1	LOC122590502	protein STRICTOSIDINE SYNTHASE-LIKE 4-like	0.0006	up
XM_043753721.1	LOC122581490	putative disease resistance RPP13-like protein 1	0.0003	up
XM_043773612.1	LOC122600837	KRR1 small subunit processome component	0.0012	up
XM_043756310.1	LOC122583934	delta(14)-sterol reductase	< 0.0001	up
XM_043773943.1	LOC122601174	UDP-glycosyltransferase 73C5-like	0.0063	up
XM_043772036.1	LOC122599508	LRR receptor-like serine/threonine-protein kinase GSO2	0.0032	up
XM_043773416.1	LOC122600664	kinesin-like protein KIN-12F	0.0005	up

Transcript id	GeneID	Gene name/function	*P* Value	result
XM_043758542.1	LOC122586560	boron transporter 4-like	0.0002	up
XM_043778870.1	LOC122605910	membrane steroid-binding protein 1-like	0.0002	up
XR_006323684.1	LOC122598662	uncharacterized LOC122598662, transcript variant X2	0.0049	up
XM_043755380.1	LOC122582949	uncharacterized LOC122582949	0.0026	up
XM_043772683.1	LOC122600039	protein OS-9 homolog	< 0.0001	up
XR_006320594.1	LOC122579075	uncharacterized LOC122579075, transcript variant X5	0.0013	up
XM_043768187.1	LOC122595754	sec14 cytosolic factor-like	0.0030	up
XM_043758402.1	LOC122586406	serine carboxypeptidase-like 12, transcript variant X2	0.0025	up
XM_043755658.1	LOC122583233	uncharacterized LOC122583233	0.0009	up
XR_006325509.1	LOC122611377	uncharacterized LOC122611377, transcript variant X3	0.0004	up
XM_043761849.1	LOC122589552	uncharacterized protein At5g50100, chloroplastic, transcript variant X4	0.0028	up
XR_006322013.1	LOC122586458	uncharacterized LOC122586458	0.0043	up
XM_043777941.1	LOC122605067	uncharacterized LOC122605067	0.0043	up
XM_043761888.1	LOC122589583	receptor-like protein EIX2	0.0005	up
XM_043773291.1	LOC122600556	growth-regulating factor 1, transcript variant X2	0.0005	up
XM_043752876.1	LOC122580611	chlorophyll a-b binding protein, chloroplastic	0.0026	up
XM_043750881.1	LOC122578833	expansin-like B1	< 0.0001	up
XM_043761411.1	LOC122589162	DNA-binding protein RHL1	0.0056	up
XM_043774424.1	LOC122601683	uncharacterized LOC122601683	0.0043	up
XM_043779263.1	LOC122606305	uncharacterized LOC122606305	0.0054	up
XM_043775026.1	LOC122602329	phosphatidylinositol/phosphatidylcholine transfer protein SFH8-like, transcript variant X2	0.0004	up
XM_043776175.1	LOC122603464	tRNA-dihydrouridine(20) synthase [NAD(P)+]-like	0.0056	up
XM_043751916.1	LOC122579695	transcription factor EGL1, transcript variant X1	0.0012	up
XM_043771309.1	LOC122598822	uncharacterized LOC122598822	< 0.0001	up
XM_043764659.1	LOC122592435	glutathione S-transferase U19-like	0.0003	up
XM_043765842.1	LOC122593431	mediator of RNA polymerase II transcription subunit 28	< 0.0001	up
XM_043766748.1	LOC122594275	leucine-rich repeat receptor protein kinase HPCA1-like	0.0003	up
XM_043780644.1	LOC122607627	uncharacterized RNA methyltransferase CT0009, transcript variant X1	0.0010	up
XM_043754699.1	LOC122582329	uncharacterized isomerase BH0283, transcript variant X2	0.0004	up
XM_043761775.1	LOC122589481	vegetative cell wall protein gp1	0.0016	up
XM_043780032.1	LOC122607116	cytochrome P450 736A117-like	< 0.0001	up
XM_043771954.1	LOC122599441	uncharacterized LOC122599441, transcript variant X1	0.0051	up
XM_043751229.1	LOC122579122	uncharacterized LOC122579122	0.0003	up
XM_043760588.1	LOC122588466	uncharacterized protein slr1919	0.0011	up
XM_043767199.1	LOC122594912	seipin-2-like	0.0050	up
XM_043761529.1	LOC122589261	ADP-ribosylation factor-like protein 2, transcript variant X2	0.0005	up
XM_043775297.1	LOC122602705	cytochrome P450 CYP72A219-like	< 0.0001	up
XM_043761156.1	LOC122588936	gibberellin-regulated protein 14	0.0005	up
XR_006323463.1	LOC122597812	protein CHROMATIN REMODELING 35-like, transcript variant X2	0.0002	up
XM_043782648.1	LOC122609700	uncharacterized LOC122609700	< 0.0001	up
XM_043772164.1	LOC122599626	protein IQ-DOMAIN 5-like, transcript variant X1	0.0035	up
XM_043759388.1	LOC122587269	WUSCHEL-related homeobox 8-like, transcript variant X3	0.0010	up
XR_006321194.1	LOC122582529	uncharacterized LOC122582529	< 0.0001	up
XM_043750485.1	LOC122578510	probable feruloyl esterase A	0.0014	up
XM_043754454.1	LOC122582096	uncharacterized LOC122582096, transcript variant X2	0.0003	up
XM_043773464.1	LOC122600712	uncharacterized LOC122600712	0.0061	up
XM_043779589.1	LOC122606731	amino acid transporter AVT1A-like	< 0.0001	up
XM_043765609.1	LOC122593227	probable plastid-lipid-associated protein 13, chloroplastic	0.0003	up
XM_043768827.1	LOC122596276	50S ribosomal protein L3, chloroplastic	0.0042	up
XM_043769975.1	LOC122597372	extensin-like	< 0.0001	up
XM_043779048.1	LOC122606088	protein VAPYRIN-like	0.0063	up
XM_043776110.1	LOC122603417	ethylene-responsive transcription factor 5-like	< 0.0001	up
XM_043783697.1	LOC122610734	putative BPI/LBP family protein At1g04970	0.0018	up
XM_043753028.1	LOC122580878	uncharacterized LOC122580878, transcript variant X1	< 0.0001	up
XM_043758288.1	LOC122586181	DEAD-box ATP-dependent RNA helicase 7-like, transcript variant X1	0.0002	up
XM_043780621.1	LOC122607607	uncharacterized LOC122607607	0.0053	up
XM_043775818.1	LOC122603178	putative protein TPRXL	< 0.0001	up
XM_043781421.1	LOC122608317	spermine synthase, transcript variant X1	< 0.0001	up
XM_043752396.1	LOC122580128	cleavage stimulation factor subunit 50, transcript variant X1	0.0009	up
XM_043769793.1	LOC122597163	uncharacterized LOC122597163	< 0.0001	up
XM_043778559.1	LOC122605599	protein STRICTOSIDINE SYNTHASE-LIKE 5-like	0.0052	up

Transcript id	GeneID	Gene name/function	*P* Value	result
XM_043766325.1	LOC122593871	suppressor protein SRP40-like	< 0.0001	up
XM_043761459.1	LOC122589208	uncharacterized LOC122589208, transcript variant X4	0.0028	up
XM_043763123.1	LOC122590949	F-box protein PP2-A13-like	< 0.0001	up
XM_043775108.1	LOC122602426	uncharacterized LOC122602426	< 0.0001	up
XM_043756428.1	LOC122584195	uncharacterized LOC122584195, transcript variant X1	< 0.0001	up
XM_043763735.1	LOC122591467	EG45-like domain containing protein	< 0.0001	up
XM_043768521.1	LOC122596024	glyoxylate/hydroxypyruvate/pyruvate reductase 2KGR-like	0.0015	up
XM_043772034.1	LOC122599505	G-type lectin S-receptor-like serine/threonine-protein kinase At4g27290	0.0006	up
XM_043781112.1	LOC122608026	protein seele	0.0023	up
XM_043771997.1	LOC122599477	G-type lectin S-receptor-like serine/threonine-protein kinase At4g27290	0.0002	up
XM_043757793.1	LOC122585665	probable E3 ubiquitin-protein ligase ZFP1, transcript variant X3	< 0.0001	up
XM_043754118.1	LOC122581824	protein WVD2-like 4, transcript variant X1	0.0004	up
XM_043756546.1	LOC122584345	uncharacterized LOC122584345	0.0008	up
XM_043765034.1	LOC122592740	3-oxoacyl-[acyl-carrier-protein] synthase, mitochondrial	0.0035	up
XM_043751239.1	LOC122579132	WAT1-related protein At3g28050-like	0.0052	up
XM_043766246.1	LOC122593795	CASP-like protein 5B2, transcript variant X1	< 0.0001	up
XM_043751520.1	LOC122579356	translation initiation factor eIF-2B subunit epsilon	0.0062	up
XM_043775955.1	LOC122603287	pre-rRNA-processing protein las1, transcript variant X1	0.0010	up
XM_043760713.1	LOC122588576	SAGA-associated factor 29 homolog A, transcript variant X1	0.0062	up
XM_043778130.1	LOC122605221	uncharacterized LOC122605221, transcript variant X3	< 0.0001	up
XM_043767284.1	LOC122595004	myosin-1, transcript variant X1	0.0029	up
XR_006322775.1	LOC122592938	uncharacterized LOC122592938	0.0026	up
XM_043751116.1	LOC122579032	uncharacterized LOC122579032	0.0012	up
XM_043772881.1	LOC122600203	uncharacterized LOC122600203	0.0011	up
XM_043771427.1	LOC122598974	putative late blight resistance protein homolog R1C-3, transcript variant X3	0.0010	up
XM_043772801.1	LOC122600136	leucine aminopeptidase 1-like	0.0013	up
XM_043760936.1	LOC122588750	uncharacterized protein At2g33490-like, transcript variant X2	0.0001	up
XM_043750543.1	LOC122578562	uncharacterized LOC122578562	< 0.0001	up
XM_043753971.1	LOC122581711	cytochrome P450 Tp9025-like, transcript variant X2	< 0.0001	up
XR_006324277.1	LOC122602460	U2 spliceosomal RNA	< 0.0001	up
XM_043763548.1	LOC122591304	uncharacterized LOC122591304, transcript variant X11	0.0007	up
XM_043773351.1	LOC122600604	cytochrome P450 93A2-like	< 0.0001	up
XM_043774822.1	LOC122602086	peptidyl-prolyl cis-trans isomerase CYP20-1-like	< 0.0001	up
XM_043768198.1	LOC122595762	50S ribosomal protein L18, chloroplastic	< 0.0001	up
XM_043752567.1	LOC122580290	zinc finger protein CONSTANS-LIKE 6	< 0.0001	up
XM_043778876.1	LOC122605915	uncharacterized LOC122605915, transcript variant X1	< 0.0001	up
XM_043779639.1	LOC122606785	probable acyl-activating enzyme 17, peroxisomal	0.0001	up
XM_043758797.1	LOC122586807	plastocyanin-like	< 0.0001	up
XM_043752130.1	LOC122579868	probable methyltransferase PMT5	0.0013	up
XM_043759382.1	LOC122587267	60S ribosomal protein L35a-1-like, transcript variant X2	0.0028	up
XM_043762465.1	LOC122590129	stemmadenine O-acetyltransferase-like	< 0.0001	up
XM_043768231.1	LOC122595788	pentatricopeptide repeat-containing protein At1g30610, chloroplastic	< 0.0001	up
XM_043773512.1	LOC122600756	ATP-dependent DNA helicase Q-like 2	0.0062	up
XM_043778470.1	LOC122605515	pentatricopeptide repeat-containing protein At2g27800, mitochondrial-like	0.0001	up
XM_043757894.1	LOC122585771	uncharacterized LOC122585771	0.0024	up
XR_006324425.1	LOC122602977	uncharacterized LOC122602977, transcript variant X2	< 0.0001	up
XM_043750105.1	LOC122578198	thiosulfate sulfurtransferase 16, chloroplastic-like	< 0.0001	up
XM_043765955.1	LOC122593533	U-box domain-containing protein 10-like	0.0033	up
XM_043761777.1	LOC122589483	growth-regulating factor 1-like	0.0019	up
XM_043765331.1	LOC122592999	protein trichome birefringence-like 35, transcript variant X1	0.0015	up
XM_043750829.1	LOC122578791	tetratricopeptide repeat protein 5-like	< 0.0001	up
XM_043773383.1	LOC122600636	transcription factor MYB14-like	< 0.0001	up
XM_043768296.1	LOC122595840	putative uncharacterized protein DDB_G0270496, transcript variant X3	< 0.0001	up
XM_043761332.1	LOC122589090	plant intracellular Ras-group-related LRR protein 7-like	0.0004	up
XM_043783059.1	LOC122610076	chaperone protein dnaJ C76, chloroplastic-like	0.0013	up
XM_043756409.1	LOC122584150	protein CASP	0.0049	up
XM_043753811.1	LOC122581577	pentatricopeptide repeat-containing protein At4g20740, transcript variant X2	0.0041	up
XM_043784298.1	LOC122611294	AAA-ATPase At2g18193-like	0.0022	up
XM_043762329.1	LOC122589996	pentatricopeptide repeat-containing protein At5g50280, chloroplastic	0.0062	up

Transcript id	GeneID	Gene name/function	*P* Value	result
XM_043775880.1	LOC122603228	fe-S cluster assembly factor HCF101, chloroplastic, transcript variant X2	< 0.0001	up
XM_043752644.1	LOC122580372	protein yippee-like At4g27740	< 0.0001	up
XM_043775672.1	LOC122603058	F-box protein At2g32560-like	0.0003	up
XM_043768080.1	LOC122595663	membrane-bound O-acyltransferase gup1, transcript variant X1	0.0027	up
XM_043759019.1	LOC122586969	cytochrome P450 90A1-like	0.0015	up
XM_043755495.1	LOC122583056	uncharacterized LOC122583056, transcript variant X2	0.0002	up
XM_043761833.1	LOC122589534	uncharacterized LOC122589534	0.0002	up
XM_043767948.1	LOC122595566	alpha-L-fucosidase 2-like	0.0005	up
XM_043750064.1	LOC122578164	serine/threonine-protein kinase BLUS1-like, transcript variant X3	0.0010	up
XM_043755182.1	LOC122582754	serine carboxypeptidase-like 45	< 0.0001	up
XM_043760613.1	LOC122588485	uncharacterized LOC122588485	0.0016	up
XM_043754671.1	LOC122582301	peptide chain release factor APG3, chloroplastic	0.0004	up
XM_043757725.1	LOC122585603	DNA polymerase epsilon catalytic subunit A-like	0.0007	up
XM_043761015.1	LOC122588809	mannose-1-phosphate guanylyltransferase 1-like	< 0.0001	up
XM_043749982.1	LOC122578102	transcription factor HY5-like	0.0029	up
XM_043761421.1	LOC122589170	CASP-like protein 5C3, transcript variant X2	0.0003	up
XR_006325361.1	LOC122609611	U1 spliceosomal RNA	0.0002	up
XM_043779465.1	LOC122606626	G-type lectin S-receptor-like serine/threonine-protein kinase At1g67520	0.0019	up
XM_043762413.1	LOC122590077	probable pectate lyase 18	0.0011	up
XM_043758000.1	LOC122585885	transcription factor MYB62-like	< 0.0001	up
XM_043760852.1	LOC122588685	protein TAB2 homolog, chloroplastic	< 0.0001	up
XM_043764810.1	LOC122592551	ribulose-1,5 bisphosphate carboxylase/oxygenase large subunit N-methyltransferase, chloroplastic, transcript variant X2	0.0006	up
XM_043751422.1	LOC122579285	uncharacterized LOC122579285, transcript variant X2	0.0048	up
XM_043784330.1	LOC122611320	ACT domain-containing protein ACR6, transcript variant X1	0.0003	up
XM_043768345.1	LOC122595876	uncharacterized protein C630.12, transcript variant X1	0.0006	up
XM_043768065.1	LOC122595653	mitotic spindle checkpoint protein MAD1	< 0.0001	up
XM_043757701.1	LOC122585574	heavy metal-associated isoprenylated plant protein 3-like	0.0005	up
XM_043771831.1	LOC122599334	shikimate O-hydroxycinnamoyltransferase-like, transcript variant X1	0.0020	up
XM_043767552.1	LOC122595216	magnesium-chelatase subunit ChlI, chloroplastic	0.0018	up
XM_043764989.1	LOC122592698	coiled-coil domain-containing protein 22 homolog, transcript variant X1	0.0048	up
XM_043760553.1	LOC122588438	zinc finger CCCH domain-containing protein 11-like	< 0.0001	up
XM_043754545.1	LOC122582186	caffeoylshikimate esterase-like	< 0.0001	up
XM_043763217.1	LOC122591030	chlorophyll a-b binding protein of LHCII type 1-like	< 0.0001	up
XM_043783118.1	LOC122610123	formin-like protein 6	0.0006	up
XM_043765009.1	LOC122592719	non-specific lipid transfer protein GPI-anchored 31-like	0.0006	up
XM_043760991.1	LOC122588791	dolichyl-diphosphooligosaccharide--protein glycosyltransferase 48 kDa subunit	< 0.0001	up
XM_043772887.1	LOC122600209	DExH-box ATP-dependent RNA helicase DExH7, chloroplastic	0.0001	up
XM_043765752.1	LOC122593359	F-box/FBD/LRR-repeat protein At1g13570-like, transcript variant X3	0.0010	up
XM_043766446.1	LOC122593971	uncharacterized LOC122593971	< 0.0001	up
XM_043753160.1	LOC122581020	UBX domain-containing protein 1	< 0.0001	up
XR_006323494.1	LOC122597927	uncharacterized LOC122597927	< 0.0001	up
XM_043776607.1	LOC122603800	benzaldehyde dehydrogenase, mitochondrial-like, transcript variant X2	0.0005	up
XM_043758959.1	LOC122586930	galactose mutarotase-like	< 0.0001	up
XM_043758781.1	LOC122586794	chlorophyll a-b binding protein CP26, chloroplastic-like	< 0.0001	up
XM_043768358.1	LOC122595886	germacrene A synthase short form-like	< 0.0001	up
XM_043763933.1	LOC122591681	wall-associated receptor kinase 2-like	0.0005	up
XM_043781230.1	LOC122608141	mRNA-capping enzyme-like, transcript variant X2	0.0035	up
XM_043758141.1	LOC122586025	labd-13Z-ene-9,15,16-triol synthase, chloroplastic-like	< 0.0001	up
XR_006324552.1	LOC122605102	glycosyltransferase-like protein gnt14, transcript variant X1	0.0047	up
XM_043752532.1	LOC122580253	probable WRKY transcription factor 75	0.0017	up
XM_043752441.1	LOC122580166	defensin SD2-like	< 0.0001	up
XM_043775449.1	LOC122602844	formate--tetrahydrofolate ligase	0.0040	up
XM_043766643.1	LOC122594176	basic blue protein-like	< 0.0001	up
XR_006320523.1	LOC122578568	uncharacterized LOC122578568	0.0016	up
XR_006323761.1	LOC122598855	uncharacterized LOC122598855	< 0.0001	up
XM_043780546.1	LOC122607552	dehydrogenase/reductase SDR family member 12, transcript variant X1	0.0012	up
XM_043759511.1	LOC122587363	uncharacterized LOC122587363, transcript variant X1	< 0.0001	up
XM_043770566.1	LOC122597971	enoyl-[acyl-carrier-protein] reductase [NADH] 1, chloroplastic-like	< 0.0001	up

Transcript id	GeneID	Gene name/function	P Value	result
XM_043767720.1	LOC122595368	ABC transporter G family member 7, transcript variant X2	0.0010	up
XM_043776343.1	LOC122603594	metalloendoproteinase 5-MMP-like	0.0054	up
XM_043773608.1	LOC122600833	zinc finger CCCH domain-containing protein 22	< 0.0001	up
XM_043765829.1	LOC122593419	tRNA:m(4)X modification enzyme TRM13	0.0029	up
XM_043779660.1	LOC122606805	polygalacturonase inhibitor-like	< 0.0001	up
XM_043784046.1	LOC122611084	glutamyl-tRNA reductase 1, chloroplastic-like	0.0031	up
XM_043752201.1	LOC122579934	root allergen protein-like	< 0.0001	up
XR_006320840.1	LOC122580689	U2 spliceosomal RNA	0.0007	up
XM_043778746.1	LOC122605790	probable acetyltransferase NATA1-like, transcript variant X2	< 0.0001	up
XM_043763330.1	LOC122591115	survival of motor neuron-related-splicing factor 30, transcript variant X1	< 0.0001	up
XM_043768500.1	LOC122596005	endonuclease MutS2	0.0039	up
XM_043775556.1	LOC122602944	uncharacterized LOC122602944	< 0.0001	up
XM_043771973.1	LOC122599458	eukaryotic translation initiation factor 3 subunit A-like	0.0005	up
XM_043780995.1	LOC122607920	auxin-induced in root cultures protein 12-like	0.0003	up
XM_043753108.1	LOC122580958	autophagy-related protein 27, transcript variant X1	0.0010	up
XM_043753561.1	LOC122581343	protein EDS1L-like	< 0.0001	up
XM_043764333.1	LOC122592124	long chain acyl-CoA synthetase 1-like	< 0.0001	up
XR_006321167.1	LOC122582372	uncharacterized LOC122582372, transcript variant X5	0.0060	up
XM_043756834.1	LOC122584773	uncharacterized LOC122584773	0.0021	up
XM_043775670.1	LOC122603056	dolichyl-diphosphooligosaccharide--protein glycosyltransferase subunit 1B	0.0056	up
XM_043770949.1	LOC122598357	pentatricopeptide repeat-containing protein At1g22960, mitochondrial-like, transcript variant X4	< 0.0001	up
XM_043767776.1	LOC122595423	peroxisomal (S)-2-hydroxy-acid oxidase GLO4-like	< 0.0001	up
XM_043779106.1	LOC122606147	alpha-galactosidase-like	0.0021	up
XM_043764560.1	LOC122592355	probable ethanolamine kinase, transcript variant X2	0.0004	up
XR_006321032.1	LOC122581721	uncharacterized LOC122581721, transcript variant X3	< 0.0001	up
XM_043765347.1	LOC122593012	beta-amyrin 28-monooxygenase-like, transcript variant X2	< 0.0001	up
XM_043752064.1	LOC122579828	uncharacterized LOC122579828	< 0.0001	up
XM_043772199.1	LOC122599653	phosphatidylinositol glycan anchor biosynthesis class U protein-like, transcript variant X1	0.0003	up
XM_043776690.1	LOC122603860	mediator of RNA polymerase II transcription subunit 11, transcript variant X2	< 0.0001	up
XM_043768426.1	LOC122595943	geraniol 8-hydroxylase-like	< 0.0001	up
XM_043772246.1	LOC122599695	uncharacterized LOC122599695	0.0004	up
XM_043772538.1	LOC122599923	afadin- and alpha-actinin-binding protein-like	0.0038	up
XM_043781484.1	LOC122608384	cytochrome P450 93A3-like	0.0061	up
XR_006324120.1	LOC122601234	uncharacterized LOC122601234, transcript variant X2	0.0006	up
XM_043771656.1	LOC122599194	probable 1-deoxy-D-xylulose-5-phosphate synthase 2, chloroplastic	< 0.0001	up
XM_043756706.1	LOC122584661	acyltransferase Pun1-like	< 0.0001	up
XR_006321010.1	LOC122581411	uncharacterized LOC122581411, transcript variant X4	0.0009	up
XM_043765775.1	LOC122593379	EG45-like domain containing protein	< 0.0001	up
XM_043751080.1	LOC122579002	aarF domain-containing protein kinase 1	< 0.0001	up
XM_043773304.1	LOC122600564	GABA transporter 1	< 0.0001	up
XM_043768828.1	LOC122596277	probable sodium/metabolite cotransporter BASS1, chloroplastic	0.0005	up
XR_006322666.1	LOC122591049	uncharacterized LOC122591049, transcript variant X5	< 0.0001	up
XM_043769304.1	LOC122596686	alpha-1,3-mannosyl-glycoprotein 2-beta-N-acetylglucosaminyltransferase-like, transcript variant X3	< 0.0001	up
XR_006323817.1	LOC122598989	uncharacterized LOC122598989	< 0.0001	up
XM_043756796.1	LOC122584742	G-patch domain-containing protein 1-like	< 0.0001	up
XM_043754543.1	LOC122582184	protein tas-like	< 0.0001	up
XM_043751942.1	LOC122579719	uncharacterized LOC122579719	0.0038	up
XM_043770928.1	LOC122598334	LEAF RUST 10 DISEASE-RESISTANCE LOCUS RECEPTOR-LIKE PROTEIN KINASE-like 1.5	< 0.0001	up
XM_043780614.1	LOC122607600	STE20/SPS1-related proline-alanine-rich protein kinase-like	0.0005	up
XM_043753835.1	LOC122581592	TGACG-sequence-specific DNA-binding protein TGA-2.1-like, transcript variant X3	0.0001	up
XM_043750547.1	LOC122578565	CRM-domain containing factor CFM2, chloroplastic	< 0.0001	up
XM_043771907.1	LOC122599403	phosphomevalonate kinase, peroxisomal	0.0010	up
XM_043773569.1	LOC122600802	cytochrome P450 93A2-like	< 0.0001	up
XM_043778867.1	LOC122605908	2-oxoglutarate-dependent dioxygenase DAO-like	0.0063	up
XM_043767803.1	LOC122595443	serine/threonine-protein kinase PBL34-like, transcript variant X2	< 0.0001	up
XM_043773515.1	LOC122600758	4-hydroxy-3-methylbut-2-enyl diphosphate reductase, chloroplastic-like	< 0.0001	up
XR_006320503.1	LOC122578042	uncharacterized LOC122578042	0.0024	up

Transcript id	GeneID	Gene name/function	*P* Value	result
XM_043775711.1	LOC122603097	probable serine/threonine-protein kinase SIS8, transcript variant X1	0.0043	up
XR_006322724.1	LOC122592535	uncharacterized LOC122592535, transcript variant X6	0.0046	up
XM_043765871.1	LOC122593458	abscisic acid receptor PYL9	0.0045	up
XM_043761748.1	LOC122589453	LRR receptor-like serine/threonine-protein kinase FEI 2	0.0013	up
XM_043754017.1	LOC122581745	AUGMIN subunit 7	0.0023	up
XM_043772001.1	LOC122599481	peptidyl-prolyl cis-trans isomerase CYP59	0.0033	up
XM_043758120.1	LOC122586005	BTB/POZ domain-containing protein FBL11	0.0036	up
XM_043781461.1	LOC122608366	very-long-chain aldehyde decarbonylase CER3-like	0.0001	up
XM_043756533.1	LOC122584330	30S ribosomal protein S3, chloroplastic	0.0047	up
XM_043774706.1	LOC122601973	uncharacterized LOC122601973	< 0.0001	up
XM_043778807.1	LOC122605850	receptor-like protein EIX2	0.0047	up
XM_043751045.1	LOC122578973	uncharacterized LOC122578973	0.0010	up
XM_043752401.1	LOC122580131	3-hydroxy-3-methylglutaryl coenzyme A reductase 2-B-like	0.0004	up
XM_043753248.1	LOC122581100	MLO-like protein 12, transcript variant X2	0.0018	up
XR_006323901.1	LOC122599322	uncharacterized LOC122599322, transcript variant X7	< 0.0001	up
XM_043773599.1	LOC122600828	trihelix transcription factor ASR3-like, transcript variant X2	0.0044	up
XM_043775211.1	LOC122602607	uncharacterized LOC122602607	< 0.0001	up
XM_043761856.1	LOC122589556	uncharacterized LOC122589556	< 0.0001	up
XM_043758403.1	LOC122586407	serine carboxypeptidase-like 7, transcript variant X3	0.0011	up
XM_043780508.1	LOC122607520	uncharacterized LOC122607520	0.0017	up
XM_043772044.1	LOC122599517	chitinase 6-like	0.0004	up
XM_043756758.1	LOC122584711	probable WRKY transcription factor 56	< 0.0001	up
XM_043778453.1	LOC122605499	Fanconi anemia group D2 protein homolog	0.0046	up
XR_006325290.1	LOC122608698	protein PEP-RELATED DEVELOPMENT ARRESTED 1, chloroplastic, transcript variant X2	< 0.0001	up
XM_043768047.1	LOC122595640	nucleolar complex protein 2 homolog, transcript variant X1	0.0004	up
XM_043754930.1	LOC122582525	serine carboxypeptidase-like 13	0.0008	up
XR_006322597.1	LOC122590764	uncharacterized LOC122590764, transcript variant X3	0.0018	up
XM_043781052.1	LOC122607969	ribosome-recycling factor, chloroplastic	0.0027	up
XM_043755488.1	LOC122583044	norbelladine synthase-like	0.0009	up
XM_043773210.1	LOC122600488	J domain-containing protein spf31	< 0.0001	up
XM_043766715.1	LOC122594243	acanthoscurrin-2-like	< 0.0001	up
XM_043782619.1	LOC122609661	signal peptidase complex catalytic subunit SEC11A-like	0.0038	up
XR_006325330.1	LOC122609544	uncharacterized LOC122609544	0.0002	up
XM_043782790.1	LOC122609844	cytochrome P450 93A3-like	< 0.0001	up
XM_043779054.1	LOC122606091	altered inheritance rate of mitochondria protein 25	0.0052	up
XM_043778134.1	LOC122605226	thaumatin-like protein 1	0.0004	up
XM_043752653.1	LOC122580383	peroxidase 10-like	< 0.0001	up
XM_043766203.1	LOC122593756	uncharacterized LOC122593756	0.0009	up
XM_043773082.1	LOC122600374	RGG repeats nuclear RNA binding protein A-like, transcript variant X2	0.0001	up
XM_043761750.1	LOC122589458	uncharacterized LOC122589458	< 0.0001	up
XR_006322432.1	LOC122590272	uncharacterized LOC122590272	0.0001	up
XM_043752023.1	LOC122579788	uncharacterized LOC122579788	0.0012	up
XM_043754391.1	LOC122582044	pectin acetylesterase 5-like	< 0.0001	up
XM_043764618.1	LOC122592401	gamma-glutamyl hydrolase 2-like	0.0016	up
XM_043750290.1	LOC122578345	cellulose synthase-like protein G3	< 0.0001	up
XM_043765318.1	LOC122592987	protein trichome birefringence-like 38	< 0.0001	up
XM_043750800.1	LOC122578765	E3 ubiquitin-protein ligase BRE1-like 2	0.0006	up
XM_043777142.1	LOC122604253	uncharacterized LOC122604253	0.0020	up
XM_043749972.1	LOC122578095	ATP-dependent 6-phosphofructokinase 5, chloroplastic	0.0010	up
XM_043754106.1	LOC122581814	ubinuclein-2-like	0.0041	up
XM_043756561.1	LOC122584372	30S ribosomal protein S4, chloroplastic	0.0013	up
XM_043758767.1	LOC122586782	prostamide/prostaglandin F synthase, transcript variant X2	< 0.0001	up
XR_006324463.1	LOC122603477	histone-lysine N-methyltransferase ATXR2, transcript variant X2	0.0050	up
XM_043766066.1	LOC122593636	uncharacterized LOC122593636	0.0053	up
XM_043777320.1	LOC122604427	receptor-like protein EIX2	0.0040	up
XM_043763199.1	LOC122591014	zinc finger BED domain-containing protein DAYSLEEPER-like, transcript variant X1	0.0059	up
XM_043777965.1	LOC122605087	probable prolyl 4-hydroxylase 3, transcript variant X2	0.0019	up
XM_043751999.1	LOC122579771	alpha-1,3-mannosyl-glycoprotein 2-beta-N-acetylglucosaminyltransferase, transcript variant X3	0.0003	up
XM_043768424.1	LOC122595941	zinc transport protein ZntB-like, transcript variant X2	0.0002	up
XM_043775352.1	LOC122602758	endonuclease MutS2, transcript variant X1	0.0016	up
XM_043771855.1	LOC122599352	photosystem I reaction center subunit II, chloroplastic-like	0.0001	up

Transcript id	GeneID	Gene name/function	*P* Value	result
XM_043767307.1	LOC122595030	putative 1-phosphatidylinositol-3-phosphate 5-kinase FAB1C	< 0.0001	up
XM_043772346.1	LOC122599776	ATP-dependent RNA helicase DHX8, transcript variant X3	0.0027	up
XM_043757242.1	LOC122585137	expansin-like B1	< 0.0001	up
XM_043749930.1	LOC122578063	serine protease SPPA, chloroplastic	0.0014	up
XM_043771327.1	LOC122598847	tRNA A64-2'-O-ribosylphosphate transferase, transcript variant X1	0.0042	up
XM_043773090.1	LOC122600381	ribosomal RNA processing protein 36 homolog	0.0026	up
XM_043773593.1	LOC122600822	uncharacterized LOC122600822	0.0050	up
XM_043780434.1	LOC122607457	uncharacterized LOC122607457	0.0057	up
XM_043761299.1	LOC122589056	splicing factor U2AF-associated protein 2	0.0057	up
XM_043765021.1	LOC122592726	enoyl-[acyl-carrier-protein] reductase, mitochondrial	0.0025	up
XM_043772992.1	LOC122600289	protein PGR	0.0063	up
XM_043779819.1	LOC122606925	uncharacterized LOC122606925	< 0.0001	up
XM_043765838.1	LOC122593428	transcription factor TCP4-like, transcript variant X1	0.0003	up
XM_043780352.1	LOC122607390	cytochrome P450 93A3-like	0.0004	up
XM_043770432.1	LOC122597854	tRNA-specific adenosine deaminase TAD2-like, transcript variant X2	0.0024	up
XM_043774875.1	LOC122602152	uncharacterized LOC122602152, transcript variant X1	0.0063	up
XM_043766323.1	LOC122593869	uncharacterized LOC122593869	0.0027	up
XM_043768409.1	LOC122595931	U11/U12 small nuclear ribonucleoprotein 59 kDa protein, transcript variant X1	0.0045	up
XM_043784227.1	LOC122611238	protein PLASTID TRANSCRIPTIONALLY ACTIVE 10	0.0045	up
XR_006324447.1	LOC122603336	uncharacterized LOC122603336, transcript variant X2	0.0040	up
XM_043769999.1	LOC122597400	uncharacterized LOC122597400	0.0002	up
XM_043776041.1	LOC122603364	vacuolar-sorting receptor 6-like	< 0.0001	up
XM_043782151.1	LOC122609090	uncharacterized LOC122609090	< 0.0001	up
XM_043773878.1	LOC122601104	delta-1-pyrroline-5-carboxylate synthase, transcript variant X1	< 0.0001	up
XM_043750279.1	LOC122578336	uncharacterized protein PFB0145c-like	0.0028	up
XM_043751108.1	LOC122579027	uncharacterized LOC122579027	0.0009	up
XM_043772220.1	LOC122599675	G-type lectin S-receptor-like serine/threonine-protein kinase At1g67520	0.0027	up
XM_043780236.1	LOC122607296	mannose-P-dolichol utilization defect 1 protein homolog 2	< 0.0001	up
XM_043782024.1	LOC122608949	E3 ubiquitin-protein ligase AIRP2-like, transcript variant X1	0.0050	up
XM_043761794.1	LOC122589499	kinesin-like protein KIN-14C, transcript variant X1	< 0.0001	up
XM_043782016.1	LOC122608948	uncharacterized LOC122608948	< 0.0001	up
XM_043781853.1	LOC122608762	uncharacterized LOC122608762	0.0003	up
XM_043765060.1	LOC122592762	serine/threonine-protein kinase 24-like	< 0.0001	up
XM_043774137.1	LOC122601375	uncharacterized LOC122601375	0.0017	up
XM_043772650.1	LOC122600020	3-hydroxyisobutyryl-CoA hydrolase-like protein 1, mitochondrial, transcript variant X1	< 0.0001	up
XM_043750055.1	LOC122578156	AP-1 complex subunit sigma-2, transcript variant X1	0.0046	up
XM_043772981.1	LOC122600278	uncharacterized LOC122600278	0.0026	up
XM_043767565.1	LOC122595229	DUF21 domain-containing protein At4g14240-like	0.0052	up
XM_043750439.1	LOC122578468	transcription factor MYB62-like	0.0009	up
XM_043763767.1	LOC122591502	preprotein translocase subunit SCY1, chloroplastic	< 0.0001	up
XM_043776004.1	LOC122603327	phosphoenolpyruvate carboxykinase (ATP) 1-like	0.0049	up
XR_006322347.1	LOC122589800	uncharacterized LOC122589800, transcript variant X9	0.0062	up
XM_043752568.1	LOC122580291	uncharacterized LOC122580291	< 0.0001	up
XM_043763241.1	LOC122591052	pectin acetylesterase 10-like	0.0012	up
XM_043781050.1	LOC122607966	G-type lectin S-receptor-like serine/threonine-protein kinase SD1-1, transcript variant X1	0.0028	up
XM_043765846.1	LOC122593435	costars family protein-like	0.0010	up
XM_043776618.1	LOC122603805	alpha-crystallin domain-containing protein 22.3, transcript variant X2	0.0004	up
XM_043758894.1	LOC122586879	interaptin	0.0019	up
XM_043767525.1	LOC122595194	protein RICE SALT SENSITIVE 3-like, transcript variant X2	0.0006	up
XM_043775237.1	LOC122602638	arogenate dehydrogenase 2, chloroplastic-like	< 0.0001	up
XM_043774280.1	LOC122601527	uncharacterized LOC122601527	< 0.0001	up
XM_043751451.1	LOC122579296	synaptotagmin-3-like, transcript variant X1	0.0006	up
XR_006321046.1	LOC122581789	uncharacterized LOC122581789, transcript variant X3	0.0004	up
XM_043780139.1	LOC122607209	ribonuclease 2-like	0.0005	up
XM_043751762.1	LOC122579573	uncharacterized LOC122579573	< 0.0001	up
XR_006321223.1	LOC122582678	uncharacterized LOC122582678	< 0.0001	up
XM_043763684.1	LOC122591413	DNA glycosylase/AP lyase ROS1-like	0.0007	up
XM_043753187.1	LOC122581049	phosphoinositide phospholipase C 2-like	< 0.0001	up
XM_043778952.1	LOC122605987	uncharacterized LOC122605987	< 0.0001	up
XM_043758779.1	LOC122586792	chlorophyll a-b binding protein CP26, chloroplastic-like	0.0023	up
XR_006322871.1	LOC122593808	protein FLX-like 1, transcript variant X3	0.0005	up

Transcript id	GeneID	Gene name/function	*P* Value	result
XM_043782445.1	LOC122609397	non-specific lipid transfer protein GPI-anchored 5-like	< 0.0001	up
XR_006322243.1	LOC122589258	uncharacterized LOC122589258, transcript variant X2	0.0001	up
XM_043756205.1	LOC122583824	scarecrow-like protein 3	0.0039	up
XM_043754787.1	LOC122582399	putative disease resistance protein RGA3	< 0.0001	up
XM_043752097.1	LOC122579835	lipid droplet-associated hydrolase, transcript variant X1	0.0032	up
XM_043766380.1	LOC122593918	uncharacterized LOC122593918, transcript variant X1	0.0002	up
XM_043779296.1	LOC122606355	uncharacterized LOC122606355, transcript variant X2	0.0003	up
XM_043755206.1	LOC122582773	bZIP transcription factor 53-like	< 0.0001	up
XM_043773899.1	LOC122601131	uncharacterized LOC122601131	0.0038	up
XM_043755411.1	LOC122582978	F-box protein At2g02240-like	0.0002	up
XM_043757371.1	LOC122585251	probable serine/threonine-protein kinase PBL3	0.0037	up
XM_043764552.1	LOC122592349	temperature-induced lipocalin-1-like	< 0.0001	up
XM_043775801.1	LOC122603168	systemin receptor SR160	< 0.0001	up
XM_043755266.1	LOC122582830	glucan endo-1,3-beta-glucosidase-like	0.0029	up
XM_043759475.1	LOC122587341	uncharacterized LOC122587341, transcript variant X2	0.0047	up
XM_043762614.1	LOC122590287	uncharacterized LOC122590287	0.0017	up
XM_043759469.1	LOC122587335	ycf3-interacting protein 1, chloroplastic	0.0002	up
XM_043778581.1	LOC122605623	protein PLASTID TRANSCRIPTIONALLY ACTIVE 7, transcript variant X2	0.0060	up
XM_043778201.1	LOC122605291	high mobility group B protein 15-like	< 0.0001	up
XM_043760853.1	LOC122588686	acyltransferase-like protein At3g26840, chloroplastic, transcript variant X1	0.0028	up
XM_043757291.1	LOC122585181	cysteine-rich receptor-like protein kinase 2	0.0043	up
XM_043776199.1	LOC122603486	pentatricopeptide repeat-containing protein At5g02830, chloroplastic	0.0022	up
XM_043750652.1	LOC122578647	11-beta-hydroxysteroid dehydrogenase-like 4A, transcript variant X3	0.0005	up
XM_043783844.1	LOC122610886	ras-related protein RABA6a-like	< 0.0001	up
XM_043771498.1	LOC122599048	probable magnesium transporter NIPA4, transcript variant X1	0.0002	up
XM_043751419.1	LOC122579283	nucleolar protein 56-like	0.0020	up
XM_043782825.1	LOC122609879	chaperone protein dnaJ 8, chloroplastic-like	0.0038	up
XM_043761679.1	LOC122589386	uncharacterized LOC122589386, transcript variant X1	0.0041	up
XM_043764850.1	LOC122592584	MADS-box protein SOC1, transcript variant X4	< 0.0001	up
XM_043754501.1	LOC122581841	pentatricopeptide repeat-containing protein At1g69290, transcript variant X3	0.0057	up
XR_006323567.1	LOC122598262	uncharacterized LOC122598262	< 0.0001	up
XM_043771423.1	LOC122598968	beta-1,3-galactosyltransferase 7-like	0.0059	up
XM_043751233.1	LOC122579128	protein LAZ1 homolog 1	0.0023	up
XM_043779782.1	LOC122606895	GDSL esterase/lipase At5g62930	< 0.0001	up
XM_043778834.1	LOC122605873	uncharacterized LOC122605873, transcript variant X1	0.0007	up
XM_043753564.1	LOC122581347	peptidyl-prolyl cis-trans isomerase FKBP42	0.0007	up
XM_043779528.1	LOC122606682	uncharacterized LOC122606682, transcript variant X2	0.0029	up
XM_043762370.1	LOC122590033	centromere-associated protein E, transcript variant X3	< 0.0001	up
XM_043754113.1	LOC122581820	probable pyridoxal 5'-phosphate synthase subunit PDX2	0.0003	up
XM_043768060.1	LOC122595647	putative lipid-binding protein At4g00165	0.0062	up
XM_043777537.1	LOC122604662	50S ribosomal protein L9, chloroplastic	< 0.0001	up
XR_006322178.1	LOC122588694	uncharacterized LOC122588694, transcript variant X1	0.0013	up
XM_043750746.1	LOC122578726	CRS2-associated factor 2, chloroplastic	0.0012	up
XM_043773407.1	LOC122600655	SOSS complex subunit B homolog	0.0021	up
XR_006323084.1	LOC122594722	U1 spliceosomal RNA	0.0056	up
XM_043757710.1	LOC122585586	pyruvate kinase, cytosolic isozyme-like	0.0017	up
XM_043754014.1	LOC122581743	DNA repair protein RAD51 homolog 4, transcript variant X2	0.0041	up
XM_043764483.1	LOC122592288	glucose-6-phosphate 1-dehydrogenase, chloroplastic, transcript variant X1	0.0004	up
XM_043754691.1	LOC122582320	NADH dehydrogenase [ubiquinone] 1 beta subcomplex subunit 7	0.0025	up
XM_043758780.1	LOC122586793	chlorophyll a-b binding protein CP26, chloroplastic-like	0.0002	up
XM_043780600.1	LOC122607592	patellin-6	< 0.0001	up
XR_006324892.1	LOC122606380	small nucleolar RNA U3	0.0009	up
XM_043768547.1	LOC122596044	F-box protein At4g00893-like	< 0.0001	up
XM_043758140.1	LOC122586024	isoprenylcysteine alpha-carbonyl methylesterase ICME-like	< 0.0001	up
XM_043772980.1	LOC122600277	26S proteasome non-ATPase regulatory subunit 12 homolog A-like	0.0014	up
XM_043783860.1	LOC122610906	uncharacterized LOC122610906	0.0047	up
XM_043776963.1	LOC122604062	uncharacterized LOC122604062	0.0039	up
XR_006322258.1	LOC122589398	uncharacterized LOC122589398, transcript variant X2	< 0.0001	up
XM_043783377.1	LOC122610382	amino acid transporter AVT6A-like	0.0003	up
XM_043779050.1	LOC122606090	miraculin-like	< 0.0001	up
XM_043776005.1	LOC122603328	WD repeat-containing protein DWA2-like	0.0004	up

Transcript id	GeneID	Gene name/function	*P* Value	result
XM_043778648.1	LOC122605693	RING-H2 finger protein ATL46-like	0.0020	up
XM_043779499.1	LOC122606658	plasma membrane ATPase 1-like, transcript variant X2	0.0062	up
XM_043775062.1	LOC122602363	early endosome antigen 1-like	0.0036	up
XR_006324137.1	LOC122601995	uncharacterized LOC122601995	< 0.0001	up
XM_043780851.1	LOC122607802	carboxypeptidase SOL1	0.0001	up
XM_043782177.1	LOC122609116	probable aspartic proteinase GIP2	< 0.0001	up
XM_043784002.1	LOC122611049	trihelix transcription factor DF1	< 0.0001	up
XM_043780594.1	LOC122607587	cysteine-rich repeat secretory protein 55	< 0.0001	up
XR_006322832.1	LOC122593405	uncharacterized LOC122593405, transcript variant X2	< 0.0001	up
XM_043763300.1	LOC122591097	uncharacterized LOC122591097	0.0045	up
XM_043759533.1	LOC122587384	protein IN CHLOROPLAST ATPASE BIOGENESIS, chloroplastic-like, transcript variant X1	0.0015	up
XM_043761200.1	LOC122588974	boron transporter 4-like	< 0.0001	up
XM_043777604.1	LOC122604735	probable mitochondrial saccharopine dehydrogenase-like oxidoreductase At5g39410	0.0040	up
XM_043756999.1	LOC122584919	uncharacterized LOC122584919, transcript variant X1	< 0.0001	up
XM_043781198.1	LOC122608109	sodium/hydrogen exchanger 6	0.0012	up
XM_043757352.1	LOC122585232	signal peptidase complex catalytic subunit SEC11A-like	0.0024	up
XM_043769319.1	LOC122596698	growth-regulating factor 6, transcript variant X2	< 0.0001	up
XM_043763080.1	LOC122590905	phospholipid--sterol O-acyltransferase, transcript variant X2	0.0045	up
XM_043781665.1	LOC122608571	heat shock cognate 70 kDa protein-like	0.0008	up
XM_043761245.1	LOC122589014	uncharacterized LOC122589014, transcript variant X3	0.0004	up
XM_043773566.1	LOC122600800	protein MAIN-LIKE 2, transcript variant X3	0.0002	up
XM_043763525.1	LOC122591285	putative DUF21 domain-containing protein At3g13070, chloroplastic	0.0021	up
XR_006324863.1	LOC122606346	U2 spliceosomal RNA	0.0031	up
XM_043768997.1	LOC122596422	2-hydroxy-palmitic acid dioxygenase MPO1-like, transcript variant X2	0.0017	up
XM_043750461.1	LOC122578487	phosphoenolpyruvate carboxykinase (ATP) 1-like	< 0.0001	up
XM_043761359.1	LOC122589116	zinc finger CCCH domain-containing protein 15 homolog	0.0052	up
XM_043751932.1	LOC122579711	2-oxoisovalerate dehydrogenase subunit alpha 1, mitochondrial-like	< 0.0001	up
XM_043768257.1	LOC122595808	uncharacterized LOC122595808, transcript variant X2	< 0.0001	up
XR_006322081.1	LOC122586840	uncharacterized LOC122586840, transcript variant X3	0.0062	up
XM_043781571.1	LOC122608463	inactive protein kinase SELMODRAFT_444075-like	0.0051	up
XM_043751040.1	LOC122578968	vacuolar protein sorting-associated protein 52 A, transcript variant X1	0.0015	up
XM_043762526.1	LOC122590191	renalase, transcript variant X1	0.0041	up
XM_043776674.1	LOC122603847	disease resistance protein Roq1-like	0.0015	up
XM_043757177.1	LOC122585082	uncharacterized LOC122585082	0.0039	up
XM_043758924.1	LOC122586903	uncharacterized LOC122586903	< 0.0001	up
XM_043781705.1	LOC122608611	homogentisate phytyltransferase 1, chloroplastic-like	0.0039	up
XM_043768140.1	LOC122595714	elongation of fatty acids protein 3-like	0.0010	up
XM_043771829.1	LOC122599332	phosphatidylinositol 4-phosphate 5-kinase 7-like	< 0.0001	up
XM_043781684.1	LOC122608588	putative F-box/LRR-repeat protein At5g02930	0.0034	up
XM_043764911.1	LOC122592628	photosystem I subunit O	< 0.0001	up
XM_043777541.1	LOC122604668	DNA-directed RNA polymerase III subunit RPC3	< 0.0001	up
XM_043781706.1	LOC122608603	probable E3 ubiquitin-protein ligase MARCHF10, transcript variant X2	< 0.0001	up
XM_043771337.1	LOC122598860	uncharacterized LOC122598860, transcript variant X1	< 0.0001	up
XM_043778517.1	LOC122605560	uncharacterized LOC122605560	0.0002	up
XM_043780387.1	LOC122607418	uncharacterized LOC122607418, transcript variant X1	< 0.0001	up
XM_043770293.1	LOC122597728	sugar transport protein 14	< 0.0001	up
XM_043765760.1	LOC122593365	magnesium-dependent phosphatase 1	0.0019	up
XM_043755174.1	LOC122582748	protein BPS1, chloroplastic-like	0.0009	up
XM_043751842.1	LOC122579636	uncharacterized LOC122579636	0.0019	up
XM_043757173.1	LOC122585041	E3 ubiquitin-protein ligase MBR1-like, transcript variant X1	< 0.0001	up
XM_043779979.1	LOC122607059	villin-5-like	0.0059	up
XM_043780635.1	LOC122607619	uncharacterized LOC122607619	0.0019	up
XM_043761136.1	LOC122588919	coiled-coil domain-containing protein 115, transcript variant X2	0.0045	up
XM_043771863.1	LOC122599357	uncharacterized LOC122599357	0.0015	up
XM_043768852.1	LOC122596303	protein gamma response 1	0.0033	up
XM_043767983.1	LOC122595591	protein LAZ1-like, transcript variant X2	0.0008	up
XM_043752748.1	LOC122580475	glycosyltransferase BC10-like, transcript variant X1	< 0.0001	up
XM_043758487.1	LOC122586503	cysteine synthase-like	0.0004	up
XM_043777948.1	LOC122605076	uncharacterized LOC122605076	0.0042	up
XM_043783456.1	LOC122610469	uncharacterized LOC122610469	< 0.0001	up

Transcript id	GeneID	Gene name/function	*P* Value	result
XM_043771667.1	LOC122599203	isoleucine--tRNA ligase, chloroplastic/mitochondrial, transcript variant X2	0.0061	up
XM_043779537.1	LOC122606689	putative cysteine-rich receptor-like protein kinase 23, transcript variant X2	0.0039	up
XM_043764485.1	LOC122592290	chaperone protein dnaJ 20, chloroplastic-like	< 0.0001	up
XM_043780377.1	LOC122607407	brassinosteroid-responsive RING protein 1-like	< 0.0001	up
XM_043777747.1	LOC122604883	spermidine synthase	< 0.0001	up
XM_043769109.1	LOC122596522	uncharacterized LOC122596522	0.0059	up
XM_043762944.1	LOC122590771	protein CURVATURE THYLAKOID 1A, chloroplastic-like	0.0002	up
XM_043767843.1	LOC122595477	guanine nucleotide-binding protein alpha-1 subunit, transcript variant X1	< 0.0001	up
XM_043767521.1	LOC122595191	uncharacterized LOC122595191	< 0.0001	up
XM_043784094.1	LOC122611124	ethylene-responsive transcription factor RAP2-3, transcript variant X1	0.0056	up
XM_043771622.1	LOC122599166	PGR5-like protein 1A, chloroplastic	< 0.0001	up
XM_043763609.1	LOC122591345	uncharacterized LOC122591345	< 0.0001	up
XM_043773598.1	LOC122600827	CRM-domain containing factor CFM3A, chloroplastic/mitochondrial-like	0.0015	up
XM_043782600.1	LOC122609572	magnesium-protoporphyrin IX monomethyl ester [oxidative] cyclase, chloroplastic	< 0.0001	up
XM_043780926.1	LOC122607858	50S ribosomal protein L18-like, transcript variant X1	0.0001	up
XM_043783976.1	LOC122611025	uncharacterized LOC122611025	< 0.0001	up
XM_043776817.1	LOC122603950	protein EARLY RESPONSIVE TO DEHYDRATION 15-like	< 0.0001	up
XM_043778448.1	LOC122605494	presequence protease 1, chloroplastic/mitochondrial-like	0.0039	up
XM_043770523.1	LOC122597930	uncharacterized LOC122597930	< 0.0001	up
XM_043759234.1	LOC122587144	protein disulfide isomerase-like 1-6	< 0.0001	up
XM_043772230.1	LOC122599683	beta-glucosidase 18-like	0.0023	up
XR_006323046.1	LOC122594603	uncharacterized LOC122594603	0.0008	up
XM_043767558.1	LOC122595222	DUF21 domain-containing protein At4g33700-like	< 0.0001	up
XM_043757875.1	LOC122585746	labd-13Z-ene-9,15,16-triol synthase, chloroplastic-like	< 0.0001	up
XM_043751937.1	LOC122579714	uncharacterized LOC122579714	0.0007	up
XM_043772813.1	LOC122600147	tryptophan synthase beta chain 1-like	0.0013	up
XM_043764740.1	LOC122592497	protein PLASTID TRANSCRIPTIONALLY ACTIVE 14	0.0028	up
XM_043753566.1	LOC122581345	two-component response regulator-like APRR2, transcript variant X1	0.0003	up
XM_043776623.1	LOC122603806	uncharacterized LOC122603806, transcript variant X1	0.0017	up
XM_043777079.1	LOC122604180	traB domain-containing protein, transcript variant X1	0.0040	up
XM_043778230.1	LOC122605311	myosin-binding protein 3-like	< 0.0001	up
XM_043754521.1	LOC122582163	homeobox-leucine zipper protein HOX11-like	< 0.0001	up
XM_043763453.1	LOC122591224	4-alpha-glucanotransferase DPE2, transcript variant X1	0.0035	up
XM_043751282.1	LOC122579169	protein PAM68, chloroplastic	< 0.0001	up
XM_043764861.1	LOC122592592	transmembrane protein 205	< 0.0001	up
XM_043778039.1	LOC122605152	uncharacterized protein At5g08430-like, transcript variant X3	0.0045	up
XM_043782914.1	LOC122609952	photosystem I reaction center subunit II, chloroplastic-like	< 0.0001	up
XM_043756988.1	LOC122584912	cytochrome P450 76C1-like	< 0.0001	up
XM_043762714.1	LOC122590516	acetyl-CoA acetyltransferase, cytosolic 1-like	0.0015	up
XM_043773736.1	LOC122600958	MATH domain and coiled-coil domain-containing protein At3g44800-like, transcript variant X1	< 0.0001	up
XM_043771282.1	LOC122598734	ornithine transcarbamylase, chloroplastic, transcript variant X1	0.0055	up
XM_043754932.1	LOC122582528	putative disease resistance RPP13-like protein 1	< 0.0001	up
XM_043775875.1	LOC122603225	protein GRIP	0.0003	up
XM_043771655.1	LOC122599193	probable 1-deoxy-D-xylulose-5-phosphate synthase 2, chloroplastic	< 0.0001	up
XR_006322331.1	LOC122589767	uncharacterized LOC122589767	0.0009	up
XM_043771641.1	LOC122599179	putative germin-like protein 2-1	< 0.0001	up
XR_006325021.1	LOC122607263	uncharacterized LOC122607263, transcript variant X2	0.0022	up
XM_043781008.1	LOC122607933	probable serine/threonine-protein kinase PBL10, transcript variant X4	0.0002	up
XM_043762515.1	LOC122590182	B3 domain-containing protein Os01g0234100-like, transcript variant X1	0.0003	up
XM_043753871.1	LOC122581629	putative uridine kinase C227.14	0.0044	up
XM_043778762.1	LOC122605806	granule-bound starch synthase 2, chloroplastic/amyloplastic-like	< 0.0001	up
XM_043775983.1	LOC122603309	ATP-dependent zinc metalloprotease FTSH 11, chloroplastic/mitochondrial	0.0025	up
XM_043773265.1	LOC122600535	calcineurin B-like protein 10	0.0013	up
XM_043772731.1	LOC122600075	uncharacterized LOC122600075	0.0004	up
XR_006323768.1	LOC122598878	uncharacterized LOC122598878	< 0.0001	up
XM_043757282.1	LOC122585173	RNA polymerase sigma factor sigF, chloroplastic, transcript variant X3	< 0.0001	up

Transcript id	GeneID	Gene name/function	*P* Value	result
XM_043777915.1	LOC122605045	protein INVOLVED IN DE NOVO 2	0.0004	up
XM_043784091.1	LOC122611122	protochlorophyllide reductase, chloroplastic-like	< 0.0001	up
XM_043751585.1	LOC122579413	uncharacterized LOC122579413	0.0057	up
XM_043763999.1	LOC122591755	15-cis-phytoene desaturase, chloroplastic/chromoplastic	0.0023	up
XM_043765513.1	LOC122593149	zinc finger protein CONSTANS-LIKE 9-like, transcript variant X1	< 0.0001	up
XM_043779754.1	LOC122606869	THO complex subunit 1	0.0001	up
XM_043754669.1	LOC122582298	60S ribosomal protein L14-2-like	0.0014	up
XM_043767966.1	LOC122595581	chlorophyll a-b binding protein of LHCII type 1	0.0005	up
XM_043753801.1	LOC122581567	protein REDUCED WALL ACETYLATION 3-like	0.0008	up
XM_043759284.1	LOC122587185	2-(3-amino-3-carboxypropyl)histidine synthase subunit 2	< 0.0001	up
XM_043778616.1	LOC122605661	nudix hydrolase 13, mitochondrial, transcript variant X2	0.0011	up
XM_043750581.1	LOC122578586	F-box/FBD/LRR-repeat protein At1g13570-like, transcript variant X4	0.0037	up
XM_043771630.1	LOC122599172	myb-like protein X	0.0006	up
XM_043778019.1	LOC122605139	monodehydroascorbate reductase 4, peroxisomal	0.0034	up
XM_043751712.1	LOC122579529	protein DELETION OF SUV3 SUPPRESSOR 1(I)-like	< 0.0001	up
XR_006323698.1	LOC122598706	U2 spliceosomal RNA	< 0.0001	up
XM_043784367.1	LOC122611348	30S ribosomal protein S9, chloroplastic	0.0006	up
XM_043753289.1	LOC122581133	nodal modulator 1	< 0.0001	up
XM_043751220.1	LOC122579113	polyprenol reductase 2-like	0.0023	up
XM_043769295.1	LOC122596676	uncharacterized LOC122596676	< 0.0001	up
XM_043763424.1	LOC122591200	succinate-semialdehyde dehydrogenase, mitochondrial	< 0.0001	up
XR_006323473.1	LOC122597825	uncharacterized LOC122597825, transcript variant X2	0.0011	up
XM_043777922.1	LOC122605049	28 kDa ribonucleoprotein, chloroplastic	0.0040	up
XM_043766140.1	LOC122593695	G-type lectin S-receptor-like serine/threonine-protein kinase At1g34300	0.0040	up
XR_006324070.1	LOC122600603	uncharacterized LOC122600603, transcript variant X3	< 0.0001	up
XM_043750363.1	LOC122578409	aspartate aminotransferase, cytoplasmic	0.0027	up
XM_043751509.1	LOC122579349	mechanosensitive ion channel protein 10-like, transcript variant X2	0.0022	up
XM_043767378.1	LOC122595081	2-oxoglutarate-dependent dioxygenase 19-like	0.0052	up
XM_043753233.1	LOC122581087	zinc finger CCCH domain-containing protein 53, transcript variant X2	0.0056	up
XM_043775036.1	LOC122602339	bifunctional aspartokinase/homoserine dehydrogenase 1, transcript variant X1	0.0004	up
XM_043784180.1	LOC122611196	uncharacterized LOC122611196, transcript variant X1	0.0052	up
XM_043768936.1	LOC122596370	UBP1-associated proteins 1C	< 0.0001	up
XM_043756874.1	LOC122584812	uncharacterized LOC122584812, transcript variant X2	0.0029	up
XM_043773023.1	LOC122600315	pentatricopeptide repeat-containing protein At1g03100, mitochondrial, transcript variant X2	0.0029	up
XM_043755359.1	LOC122582929	probable N-acetyltransferase HLS1-like	< 0.0001	up
XM_043760522.1	LOC122588413	tetratricopeptide repeat protein SKI3, transcript variant X2	0.0025	up
XM_043752037.1	LOC122579802	uncharacterized LOC122579802	0.0003	up
XM_043767182.1	LOC122594883	uncharacterized LOC122594883, transcript variant X1	< 0.0001	up
XM_043756519.1	LOC122584314	cytochrome f-like	0.0033	up
XM_043766197.1	LOC122593752	CRM-domain containing factor CFM3, chloroplastic/mitochondrial	0.0003	up
XM_043772136.1	LOC122599604	soluble inorganic pyrophosphatase 6, chloroplastic-like	0.0012	up
XM_043784233.1	LOC122611244	growth-regulating factor 8-like	0.0003	up
XM_043781869.1	LOC122608773	myosin-1-like, transcript variant X2	< 0.0001	up
XM_043754494.1	LOC122582137	glucan endo-1,3-beta-glucosidase-like	0.0063	up
XM_043752241.1	LOC122579977	replication protein A 70 kDa DNA-binding subunit A-like	< 0.0001	up
XM_043755369.1	LOC122582939	uncharacterized LOC122582939, transcript variant X2	0.0029	up
XM_043760877.1	LOC122588707	probable arabinosyltransferase ARAD1	0.0007	up
XM_043754776.1	LOC122582390	uncharacterized LOC122582390	< 0.0001	up
XR_006320649.1	LOC122579461	uncharacterized LOC122579461, transcript variant X8	0.0009	up
XM_043778498.1	LOC122605542	AP2-like ethylene-responsive transcription factor AIL7	0.0060	up
XM_043752361.1	LOC122580090	thioredoxin-like 1-2, chloroplastic	0.0011	up
XM_043758258.1	LOC122586152	serine/threonine-protein kinase SRPK-like, transcript variant X1	0.0001	up
XM_043768688.1	LOC122596158	uncharacterized LOC122596158, transcript variant X2	0.0005	up
XM_043770510.1	LOC122597920	serine carboxypeptidase-like 31	< 0.0001	up
XM_043768696.1	LOC122596165	GDSL esterase/lipase At3g26430-like	< 0.0001	up
XM_043779702.1	LOC122606829	ubiquitin-conjugating enzyme E2 4-like, transcript variant X1	0.0043	up
XM_043778051.1	LOC122605160	plant cysteine oxidase 2	< 0.0001	up
XM_043776685.1	LOC122603855	cationic amino acid transporter 5	0.0005	up
XM_043776115.1	LOC122603406	protein LTO1 homolog, transcript variant X3	< 0.0001	up
XM_043769448.1	LOC122596804	uncharacterized LOC122596804, transcript variant X1	0.0005	up
XM_043753883.1	LOC122581638	transcription termination factor MTERF2, chloroplastic	0.0059	up

Transcript id	GeneID	Gene name/function	*P* Value	result
XM_043762447.1	LOC122590112	glycosylphosphatidylinositol anchor biosynthesis protein 11	0.0006	up
XM_043752499.1	LOC122580223	demethylepipodophyllotoxin synthase-like	< 0.0001	up
XM_043754513.1	LOC122582157	60S ribosomal protein L29-2-like	< 0.0001	up
XR_006324459.1	LOC122603462	uncharacterized LOC122603462, transcript variant X4	0.0024	up
XM_043768528.1	LOC122596028	homeobox-leucine zipper protein ATHB-15-like	0.0003	up
XM_043750492.1	LOC122578517	NAP1-related protein 2	0.0015	up
XM_043771846.1	LOC122599343	putative inactive cadmium/zinc-transporting ATPase HMA3	0.0056	up
XM_043761984.1	LOC122589668	uncharacterized LOC122589668	0.0007	up
XM_043779600.1	LOC122606743	photosystem I reaction center subunit IV A, chloroplastic-like	0.0042	up
XM_043754199.1	LOC122581884	protein FAR1-RELATED SEQUENCE 5	0.0018	up
XM_043754185.1	LOC122581870	leucine-rich repeat receptor-like serine/threonine-protein kinase SKM1	< 0.0001	up
XM_043756737.1	LOC122584690	cysteine-rich receptor-like protein kinase 2	< 0.0001	up
XM_043758198.1	LOC122586089	uncharacterized LOC122586089	0.0022	up
XM_043765645.1	LOC122593258	pentatricopeptide repeat-containing protein At5g39350, transcript variant X3	0.0021	up
XM_043779947.1	LOC122607028	NAP1-related protein 1-like, transcript variant X2	0.0031	up
XM_043768612.1	LOC122596092	omega-6 fatty acid desaturase, chloroplastic-like, transcript variant X1	< 0.0001	up
XR_006322465.1	LOC122590325	U2 spliceosomal RNA	< 0.0001	up
XM_043752882.1	LOC122580615	uncharacterized protein DDB_G0275933	0.0006	up
XM_043767862.1	LOC122595494	DNA-binding protein SMUBP-2	0.0055	up
XM_043775575.1	LOC122602959	dihydrofolate synthetase-like, transcript variant X2	0.0035	up
XM_043779935.1	LOC122607017	probable RNA-binding protein EIF1AD	0.0039	up
XM_043765907.1	LOC122593489	DDRGK domain-containing protein 1, transcript variant X1	0.0007	up
XM_043761485.1	LOC122589226	F-box protein SKIP24	0.0012	up
XM_043751360.1	LOC122579237	probable carboxylesterase 18	0.0041	up
XM_043763522.1	LOC122591279	epidermis-specific secreted glycoprotein EP1-like	0.0058	up
XM_043769119.1	LOC122596525	protein NUCLEAR FUSION DEFECTIVE 6, mitochondrial-like, transcript variant X2	< 0.0001	up
XR_006321928.1	LOC122586223	U1 spliceosomal RNA	0.0060	up
XR_006324474.1	LOC122603607	uncharacterized LOC122603607, transcript variant X3	< 0.0001	up
XR_006323188.1	LOC122595078	uncharacterized LOC122595078	0.0002	up
XM_043772953.1	LOC122600259	chromatin modification-related protein MEAF6-like	0.0023	up
XM_043766303.1	LOC122593853	cytochrome b-c1 complex subunit 6-1, mitochondrial-like	0.0005	up
XM_043779380.1	LOC122606526	expansin-A4-like	< 0.0001	up
XM_043774775.1	LOC122602043	zinc finger protein CONSTANS-LIKE 9-like, transcript variant X1	0.0011	up
XM_043766000.1	LOC122593573	aspartate carbamoyltransferase, chloroplastic-like	0.0063	up
XM_043765498.1	LOC122593138	F-box/FBD/LRR-repeat protein At4g00160-like	0.0025	up
XM_043761596.1	LOC122589314	myosin-1	0.0003	up
XM_043766634.1	LOC122594162	uncharacterized LOC122594162	< 0.0001	up
XM_043772437.1	LOC122599848	double-strand break repair protein MRE11	0.0031	up
XM_043775012.1	LOC122602311	glycosyltransferase BC10-like	< 0.0001	up
XM_043757255.1	LOC122585147	actin-histidine N-methyltransferase, transcript variant X2	0.0054	up
XM_043774022.1	LOC122601259	uncharacterized LOC122601259	0.0003	up
XM_043776140.1	LOC122603440	actin-related protein 2	0.0007	up
XM_043764875.1	LOC122592602	probable pyridoxal 5'-phosphate synthase subunit PDX1, transcript variant X2	0.0023	up
XM_043758494.1	LOC122586511	FBD-associated F-box protein At2g26860-like, transcript variant X2	0.0027	up
XM_043780157.1	LOC122607222	DNA polymerase zeta processivity subunit, transcript variant X2	0.0006	up
XM_043751743.1	LOC122579553	3-epi-6-deoxocathasterone 23-monooxygenase CYP90C1	0.0002	up
XM_043758977.1	LOC122586944	ABC transporter C family member 8-like	0.0052	up
XM_043770641.1	LOC122598035	bZIP transcription factor 11-like	< 0.0001	up
XM_043753422.1	LOC122581241	methylcrotonoyl-CoA carboxylase subunit alpha, mitochondrial, transcript variant X1	< 0.0001	up
XM_043780670.1	LOC122607652	lactosylceramide 4-alpha-galactosyltransferase-like	< 0.0001	up
XM_043763297.1	LOC122591094	ABSCISIC ACID-INSENSITIVE 5-like protein 7, transcript variant X1	0.0004	up
XM_043766286.1	LOC122593838	cytochrome P450 Tp4149-like	< 0.0001	up
XM_043751309.1	LOC122579194	histidine kinase 5-like	0.0008	up
XM_043755143.1	LOC122582720	D-aminoacyl-tRNA deacylase, transcript variant X2	0.0002	up
XM_043751950.1	LOC122579726	cysteine--tRNA ligase, chloroplastic/mitochondrial, transcript variant X1	0.0040	up
XM_043778661.1	LOC122605700	30S ribosomal protein 3-1, chloroplastic-like	< 0.0001	up
XM_043781752.1	LOC122608663	uncharacterized LOC122608663	0.0048	up
XM_043767450.1	LOC122595135	aldehyde dehydrogenase family 7 member B4, transcript variant X3	0.0048	up

Transcript id	GeneID	Gene name/function	*P* Value	result
XM_043771786.1	LOC122599292	beta-glucosidase 44-like	< 0.0001	up
XM_043764786.1	LOC122592533	cytochrome P450 Tp4149-like	< 0.0001	up
XM_043784300.1	LOC122611296	la protein 1	0.0009	up
XM_043752780.1	LOC122580502	uncharacterized LOC122580502, transcript variant X2	0.0003	up
XM_043781462.1	LOC122608367	subtilisin-like protease SBT3	0.0004	up
XM_043770999.1	LOC122598399	serine carboxypeptidase-like 10, transcript variant X1	0.0042	up
XM_043775508.1	LOC122602897	magnesium-chelatase subunit ChlD, chloroplastic	< 0.0001	up
XM_043749977.1	LOC122578099	pentatricopeptide repeat-containing protein At3g02330, mitochondrial, transcript variant X2	0.0043	up
XM_043779175.1	LOC122606217	coiled-coil domain-containing protein 12-like, transcript variant X4	< 0.0001	up
XM_043766894.1	LOC122594443	DNA-directed RNA polymerase III subunit 2	0.0051	up
XM_043750124.1	LOC122578215	berberine bridge enzyme-like 27	0.0023	up
XM_043754076.1	LOC122581796	40S ribosomal protein S14-3	0.0005	up
XM_043754922.1	LOC122582517	cytochrome c oxidase-assembly factor COX23, mitochondrial	< 0.0001	up
XM_043751089.1	LOC122579010	protein COFACTOR ASSEMBLY OF COMPLEX C SUBUNIT B CCB3, chloroplastic	0.0060	up
XM_043751934.1	LOC122579712	putative F-box/FBD/LRR-repeat protein At4g03220	0.0007	up
XM_043757866.1	LOC122585740	uncharacterized LOC122585740, transcript variant X1	< 0.0001	up
XM_043752094.1	LOC122579844	NAC domain-containing protein 21/22-like	< 0.0001	up
XM_043783080.1	LOC122610090	glycosyl hydrolase 5 family protein-like	0.0003	up
XM_043751623.1	LOC122579443	uncharacterized LOC122579443	0.0017	up
XM_043766021.1	LOC122593591	anaphase-promoting complex subunit 6	0.0004	up
XM_043754617.1	LOC122582250	54S ribosomal protein L24, mitochondrial-like	0.0005	up
XM_043770681.1	LOC122598070	protein PFC0760c-like, transcript variant X1	0.0017	up
XR_006324039.1	LOC122600362	uncharacterized LOC122600362	0.0007	up
XM_043763559.1	LOC122591306	protein SCAR2	< 0.0001	up
XM_043776740.1	LOC122603894	uncharacterized LOC122603894	0.0045	up
XR_006323555.1	LOC122598194	uncharacterized LOC122598194	0.0015	up
XM_043765089.1	LOC122592779	poly(A)-specific ribonuclease PARN, transcript variant X2	0.0056	up
XM_043750180.1	LOC122578263	uncharacterized LOC122578263	< 0.0001	up
XM_043778934.1	LOC122605968	protein RGF1 INDUCIBLE TRANSCRIPTION FACTOR 1	0.0030	up
XM_043764923.1	LOC122592640	protein SEMI-ROLLED LEAF 2	0.0007	up
XM_043775362.1	**LOC122602768**	O-fucosyltransferase 39-like, transcript variant X1	< 0.0001	up
XM_043775363.1	**LOC122602768**	O-fucosyltransferase 39-like, transcript variant X1	0.0001	up
XM_043782883.1	**LOC122609933**	cytochrome P450 CYP82D47-like, transcript variant X2	< 0.0001	up
XM_043782884.1	**LOC122609933**	cytochrome P450 CYP82D47-like, transcript variant X2	0.0009	up
XM_043768418.1	**LOC122595939**	mini-ribonuclease 3, transcript variant X2	< 0.0001	up
XM_043768417.1	**LOC122595939**	mini-ribonuclease 3, transcript variant X2	< 0.0001	up
XR_006323193.1	**LOC122595117**	phospholipid:diacylglycerol acyltransferase 1-like, transcript variant X4	0.0003	up
XR_006323192.1	**LOC122595117**	phospholipid:diacylglycerol acyltransferase 1-like, transcript variant X4	< 0.0001	up
XR_006325123.1	**LOC122607945**	pheromone-processing carboxypeptidase KEX1-like, transcript variant X7	0.0042	up
XR_006325124.1	**LOC122607945**	pheromone-processing carboxypeptidase KEX1-like, transcript variant X7	0.0010	up
XM_043778236.1	**LOC122605316**	coiled-coil domain-containing protein 12-like, transcript variant X1	< 0.0001	up
XM_043778234.1	**LOC122605316**	coiled-coil domain-containing protein 12-like, transcript variant X1	< 0.0001	up
XM_043770288.1	**LOC122597722**	protein GAMETE CELL DEFECTIVE 1, mitochondrial, transcript variant X4	0.0007	up
XR_006323449.1	**LOC122597722**	protein GAMETE CELL DEFECTIVE 1, mitochondrial, transcript variant X4	0.0002	up
XR_006320930.1	**LOC122580953**	uncharacterized LOC122580953, transcript variant X5	< 0.0001	up
XR_006320926.1	**LOC122580953**	uncharacterized LOC122580953, transcript variant X5	0.0004	up
XM_043775132.1	**LOC122602505**	calcineurin B-like protein 4, transcript variant X2	0.0038	up
XM_043775130.1	**LOC122602505**	calcineurin B-like protein 4, transcript variant X2	< 0.0001	up
XM_043767974.1	**LOC122595586**	uncharacterized LOC122595586, transcript variant X1	< 0.0001	up
XM_043767973.1	**LOC122595586**	uncharacterized LOC122595586, transcript variant X1	0.0034	up
XR_006324176.1	**LOC122602198**	uncharacterized LOC122602198, transcript variant X2	< 0.0001	up
XR_006324177.1	**LOC122602198**	uncharacterized LOC122602198, transcript variant X2	< 0.0001	up
XM_043776813.1	**LOC122603947**	probable ubiquitin-conjugating enzyme E2 16, transcript variant X1	0.0005	up
XM_043776814.1	**LOC122603947**	probable ubiquitin-conjugating enzyme E2 16, transcript variant X1	0.0006	up
XM_043779447.1	**LOC122606604**	peptidyl-prolyl cis-trans isomerase CYP19-4, transcript variant X1	0.0020	up
XM_043779448.1	**LOC122606604**	peptidyl-prolyl cis-trans isomerase CYP19-4, transcript variant X1	0.0048	up
XM_043769279.1	**LOC122596654**	lysine-specific demethylase JMJ30, transcript variant X1	< 0.0001	up
XM_043769271.1	**LOC122596654**	lysine-specific demethylase JMJ30, transcript variant X1	< 0.0001	up

Transcript id	GeneID	Gene name/function	P Value	result
XM_043753291.1	**LOC122581134**	sugar transporter ERD6-like 7, transcript variant X2	0.0002	up
XM_043753290.1	**LOC122581134**	sugar transporter ERD6-like 7, transcript variant X2	0.0015	up
XM_043750021.1	**LOC122578131**	protein LOL1, transcript variant X1	< 0.0001	up
XM_043750022.1	**LOC122578131**	protein LOL1, transcript variant X1	< 0.0001	up
XM_043773223.1	**LOC122600500**	dihydropyrimidinase, transcript variant X1	< 0.0001	up
XM_043773224.1	**LOC122600500**	dihydropyrimidinase, transcript variant X1	< 0.0001	up
XM_043751535.1	LOC122579369	cytokinin dehydrogenase 5-like	< 0.0001	down
XM_043773770.1	LOC122600991	protein DOG1-like 3	< 0.0001	down
XM_043771231.1	LOC122598630	chromatin structure-remodeling complex protein SYD, transcript variant X4	< 0.0001	down
XM_043755824.1	LOC122583419	uncharacterized LOC122583419	< 0.0001	down
XM_043753886.1	LOC122581640	glycine-rich protein A3-like	< 0.0001	down
XM_043760718.1	LOC122588579	E3 ubiquitin-protein ligase MBR2-like	0.0001	down
XM_043778838.1	LOC122605880	probable N-acetyltransferase HLS1	< 0.0001	down
XM_043754538.1	LOC122582180	uncharacterized protein At5g39865	< 0.0001	down
XM_043758089.1	LOC122585976	E3 ubiquitin-protein ligase ATL23	0.0041	down
XM_043769823.1	LOC122597198	uncharacterized LOC122597198	< 0.0001	down
XM_043770237.1	LOC122597664	uncharacterized LOC122597664	0.0036	down
XM_043771344.1	LOC122598864	uncharacterized LOC122598864, transcript variant X1	0.0018	down
XM_043778061.1	LOC122605167	2-methylpropanoate--CoA ligase CCL4-like	< 0.0001	down
XM_043784317.1	LOC122611307	protein WALLS ARE THIN 1-like	< 0.0001	down
XM_043751202.1	LOC122579101	elongation of fatty acids protein 3-like	0.0003	down
XM_043775529.1	LOC122602916	auxin-induced protein PCNT115-like, transcript variant X2	< 0.0001	down
XM_043768584.1	LOC122596068	receptor-like protein EIX1	< 0.0001	down
XM_043757278.1	LOC122585171	zinc finger protein ZAT11-like	0.0008	down
XM_043768504.1	LOC122596008	cucumber peeling cupredoxin-like	0.0002	down
XM_043763109.1	LOC122590934	serine/threonine-protein kinase PBL35-like	< 0.0001	down
XM_043771740.1	LOC122599259	probable 1-deoxy-D-xylulose-5-phosphate synthase 2, chloroplastic	0.0004	down
XM_043768853.1	LOC122596304	cytochrome P450 78A5-like	< 0.0001	down
XM_043777185.1	LOC122604298	uncharacterized LOC122604298	0.0005	down
XM_043780454.1	LOC122607476	mitochondrial uncoupling protein 1, transcript variant X1	< 0.0001	down
XM_043753540.1	LOC122581327	metacaspase-1	< 0.0001	down
XM_043757007.1	LOC122584925	WAT1-related protein At2g39510-like	0.0004	down
XM_043757017.1	LOC122584934	probable inorganic phosphate transporter 1-9	0.0025	down
XM_043765687.1	LOC122593295	PXMP2/4 family protein 4-like	0.0023	down
XM_043765796.1	LOC122593394	3-oxoacyl-[acyl-carrier-protein] reductase FabG-like	< 0.0001	down
XM_043754070.1	LOC122581791	protein TRANSPORT INHIBITOR RESPONSE 1-like	0.0005	down
XM_043769339.1	LOC122596715	two-component response regulator ORR26-like	0.0004	down
XM_043766741.1	LOC122594266	flavin-containing monooxygenase FMO GS-OX5-like	< 0.0001	down
XM_043759653.1	LOC122587470	ruBisCO large subunit-binding protein subunit beta, chloroplastic-like, transcript variant X2	< 0.0001	down
XM_043779495.1	LOC122606653	caffeic acid 3-O-methyltransferase-like	0.0011	down
XM_043775551.1	LOC122602937	copper transport protein CCH-like	< 0.0001	down
XM_043763511.1	LOC122591271	putative glucose-6-phosphate 1-epimerase, transcript variant X2	0.0003	down
XM_043749946.1	LOC122578077	trans-cinnamate:CoA ligase, peroxisomal-like	0.0022	down
XM_043754099.1	LOC122581810	anthocyanin 5-aromatic acyltransferase-like	< 0.0001	down
XM_043763378.1	LOC122591160	uncharacterized LOC122591160, transcript variant X2	0.0059	down
XM_043778708.1	LOC122605754	uncharacterized LOC122605754, transcript variant X2	0.0058	down
XM_043758569.1	LOC122586584	PI-PLC X domain-containing protein At5g67130-like	0.0037	down
XM_043784213.1	LOC122611226	dof zinc finger protein DOF2.5-like, transcript variant X2	0.0025	down
XM_043751656.1	LOC122579474	U-box domain-containing protein 28	0.0002	down
XM_043756263.1	LOC122583890	SPX domain-containing protein 2-like	0.0032	down
XM_043756802.1	LOC122584749	ethylene-responsive transcription factor TINY-like	< 0.0001	down
XM_043756065.1	LOC122583681	uncharacterized LOC122583681	< 0.0001	down
XM_043757797.1	LOC122585668	uncharacterized LOC122585668	0.0027	down
XM_043751255.1	LOC122579143	uncharacterized LOC122579143	< 0.0001	down
XM_043774848.1	LOC122602121	serine/threonine-protein kinase-like protein ACR4	0.0006	down
XM_043779230.1	LOC122606268	parthenolide synthase-like	0.0052	down
XM_043755306.1	LOC122582875	major allergen Pru ar 1-like	0.0025	down
XM_043767615.1	LOC122595276	putative pentatricopeptide repeat-containing protein At1g10330	0.0062	down
XM_043777157.1	LOC122604270	uncharacterized LOC122604270	0.0001	down
XM_043781463.1	LOC122608368	probable inactive receptor kinase At5g58300	< 0.0001	down
XM_043781522.1	LOC122608417	uncharacterized protein At3g28850	0.0027	down
XM_043772479.1	LOC122599877	ethylene-responsive transcription factor 1A-like	< 0.0001	down

Transcript id	GeneID	Gene name/function	*P* Value	result
XM_043772445.1	LOC122599856	phosphatidylinositol 4-phosphate 5-kinase 8-like, transcript variant X1	< 0.0001	down
XM_043779861.1	LOC122606958	mitochondrial amidoxime reducing component 2-like, transcript variant X1	0.0003	down
XM_043782575.1	LOC122609541	protein LURP-one-related 15-like	0.0001	down
XM_043757064.1	LOC122584971	uncharacterized LOC122584971	< 0.0001	down
XR_006323047.1	LOC122594606	uncharacterized LOC122594606	0.0050	down
XM_043756036.1	LOC122583650	uncharacterized LOC122583650	< 0.0001	down
XM_043781258.1	LOC122608164	xyloglucan galactosyltransferase MUR3-like	< 0.0001	down
XM_043766881.1	LOC122594425	UDP-glycosyltransferase 83A1-like	< 0.0001	down
XM_043752489.1	LOC122580211	alpha,alpha-trehalose-phosphate synthase [UDP-forming] 5	0.0011	down
XM_043756289.1	LOC122583917	ankyrin repeat-containing protein At5g02620-like	0.0004	down
XM_043775689.1	LOC122603077	lanC-like protein GCL1	< 0.0001	down
XM_043759269.1	LOC122587176	protein CLP1 homolog, transcript variant X4	< 0.0001	down
XM_043755080.1	LOC122582657	uncharacterized LOC122582657	0.0007	down
XM_043775000.1	LOC122602298	puromycin-sensitive aminopeptidase, transcript variant X1	< 0.0001	down
XM_043753099.1	LOC122580951	thioredoxin H-type-like	0.0009	down
XM_043761875.1	LOC122589572	mitogen-activated protein kinase kinase kinase 20-like	0.0002	down
XM_043779731.1	LOC122606853	probable sugar phosphate/phosphate translocator At3g11320	< 0.0001	down
XM_043753771.1	LOC122581541	uncharacterized LOC122581541	0.0020	down
XM_043762063.1	LOC122589737	ABC transporter B family member 19	0.0020	down
XM_043772837.1	LOC122600164	phosphoenolpyruvate carboxylase 4-like	0.0021	down
XM_043775334.1	LOC122602745	uncharacterized LOC122602745, transcript variant X1	< 0.0001	down
XM_043780165.1	LOC122607227	NAC transcription factor 32-like	< 0.0001	down
XM_043766767.1	LOC122594289	spermatogenesis-associated protein 20, transcript variant X2	0.0026	down
XM_043766194.1	LOC122593749	remorin, transcript variant X1	0.0020	down
XM_043763752.1	LOC122591485	wall-associated receptor kinase 2-like	0.0006	down
XM_043754176.1	LOC122581865	sterol 3-beta-glucosyltransferase UGT80A2-like, transcript variant X1	0.0055	down
XM_043764783.1	LOC122592531	suppressor of disruption of TFIIS	< 0.0001	down
XM_043775838.1	LOC122603196	BAG family molecular chaperone regulator 3-like	< 0.0001	down
XM_043751443.1	LOC122579300	2,3-bisphosphoglycerate-dependent phosphoglycerate mutase 1-like, transcript variant X2	< 0.0001	down
XM_043780346.1	LOC122607382	heterogeneous nuclear ribonucleoprotein 1-like, transcript variant X4	< 0.0001	down
XM_043766056.1	LOC122593630	AT-hook motif nuclear-localized protein 23-like	< 0.0001	down
XM_043749952.1	LOC122578082	ATP-dependent DNA helicase 2 subunit KU70, transcript variant X2	< 0.0001	down
XM_043780871.1	LOC122607819	serine/threonine-protein kinase-like protein CCR4	0.0029	down
XM_043783796.1	LOC122610840	probable glutathione S-transferase	< 0.0001	down
XM_043770716.1	LOC122598115	membrane protein PM19L-like	0.0006	down
XM_043778940.1	LOC122605975	phosphomethylethanolamine N-methyltransferase-like	0.0015	down
XM_043768621.1	LOC122596101	E3 ubiquitin-protein ligase RGLG2-like	0.0001	down
XM_043781693.1	LOC122608596	UDP-glycosyltransferase 75C1-like	0.0002	down
XM_043783841.1	LOC122610885	transcription factor WER-like	< 0.0001	down
XM_043772403.1	LOC122599819	nuclear pore complex protein NUP133, transcript variant X1	0.0014	down
XM_043779800.1	LOC122606912	WAT1-related protein At5g40240-like	0.0024	down
XM_043761603.1	LOC122589318	L-ascorbate oxidase	0.0021	down
XM_043767023.1	LOC122594584	shewanella-like protein phosphatase 2	0.0008	down
XM_043757899.1	LOC122585775	dehydration-responsive element-binding protein 1D-like	0.0001	down
XM_043749933.1	LOC122578062	monocopper oxidase-like protein SKU5	< 0.0001	down
XR_006325012.1	LOC122607034	probable galacturonosyltransferase-like 7, transcript variant X2	< 0.0001	down
XM_043770500.1	LOC122597911	lignin-forming anionic peroxidase-like	< 0.0001	down
XR_006320978.1	LOC122581131	transcription initiation factor TFIID subunit 15b, transcript variant X3	0.0052	down
XM_043780187.1	LOC122607249	argininosuccinate synthase, chloroplastic-like, transcript variant X1	< 0.0001	down
XM_043751695.1	LOC122579512	uncharacterized LOC122579512	0.0001	down
XM_043773229.1	LOC122600504	protein LATERAL ROOT PRIMORDIUM 1-like	< 0.0001	down
XM_043766687.1	LOC122594214	polyubiquitin-like	< 0.0001	down
XM_043753778.1	LOC122581549	methylsterol monooxygenase 2-2, transcript variant X1	0.0008	down
XM_043781293.1	LOC122608206	protein LIFEGUARD 2-like	0.0003	down
XM_043757222.1	LOC122585123	dof zinc finger protein DOF2.1	< 0.0001	down
XM_043751175.1	LOC122579082	transcription factor bHLH47, transcript variant X1	< 0.0001	down
XM_043773572.1	LOC122600807	NAD(P)H dehydrogenase (quinone) FQR1-like, transcript variant X1	0.0002	down
XM_043769857.1	LOC122597239	calmodulin-binding receptor-like cytoplasmic kinase 2	< 0.0001	down
XM_043765561.1	LOC122593189	serine/threonine-protein kinase ATG1c-like, transcript variant X2	0.0004	down
XM_043766024.1	LOC122593594	dof zinc finger protein DOF3.2-like	< 0.0001	down
XM_043766599.1	LOC122594121	septum-promoting GTP-binding protein 1	0.0028	down

Transcript id	GeneID	Gene name/function	*P* Value	result
XM_043770381.1	LOC122597804	glutamate receptor 2.7-like, transcript variant X2	< 0.0001	down
XM_043755177.1	LOC122582750	probable protein phosphatase 2C 4	0.0003	down
XM_043772176.1	LOC122599637	protein ENHANCED DISEASE RESISTANCE 2-like	< 0.0001	down
XM_043784209.1	LOC122611224	serine/threonine-protein phosphatase 5, transcript variant X2	0.0037	down
XM_043768327.1	LOC122595864	potassium channel AKT1	< 0.0001	down
XM_043758335.1	LOC122586341	serine/threonine-protein phosphatase PP1 isozyme 3-like	0.0030	down
XM_043776265.1	LOC122603537	hydroquinone glucosyltransferase-like	0.0003	down
XM_043765976.1	LOC122593552	uncharacterized LOC122593552	< 0.0001	down
XM_043758215.1	LOC122586108	berberine bridge enzyme-like 21, transcript variant X1	< 0.0001	down
XM_043772809.1	LOC122600142	bHLH transcription factor RHL1-like	0.0019	down
XM_043770584.1	LOC122597981	protein ANTI-SILENCING 1-like	0.0009	down
XM_043762078.1	LOC122589749	omega-hydroxypalmitate O-feruloyl transferase-like	< 0.0001	down
XM_043779850.1	LOC122606950	SNAP25 homologous protein SNAP33-like	< 0.0001	down
XM_043757314.1	LOC122585204	putative phytosulfokines 6	< 0.0001	down
XM_043771664.1	LOC122599199	aquaporin PIP2-7	< 0.0001	down
XM_043772473.1	LOC122599873	BRASSINOSTEROID INSENSITIVE 1-associated receptor kinase 1-like, transcript variant X1	0.0028	down
XM_043778843.1	LOC122605887	uncharacterized LOC122605887	0.0004	down
XM_043761496.1	LOC122589237	uncharacterized LOC122589237	< 0.0001	down
XM_043764023.1	LOC122591784	disease resistance protein RPV1-like	0.0006	down
XM_043779334.1	LOC122606485	hyoscyamine 6-dioxygenase-like	0.0027	down
XM_043760313.1	LOC122588188	rust resistance kinase Lr10-like	0.0002	down
XM_043759188.1	LOC122587110	macoilin, transcript variant X1	0.0009	down
XM_043752261.1	LOC122579993	uncharacterized LOC122579993, transcript variant X1	0.0026	down
XM_043768647.1	LOC122596126	nucleobase-ascorbate transporter 4	0.0045	down
XM_043772017.1	LOC122599492	probable xyloglucan endotransglucosylase/hydrolase protein 23	< 0.0001	down
XM_043780608.1	LOC122607595	laccase-7-like, transcript variant X1	0.0063	down
XM_043779569.1	LOC122606713	protein HUA2-LIKE 2-like	0.0060	down
XM_043784376.1	LOC122611356	ethylene-responsive transcription factor RAP2-4-like	0.0053	down
XM_043759302.1	LOC122587200	uncharacterized LOC122587200	< 0.0001	down
XM_043763425.1	LOC122591201	gibberellin 2-beta-dioxygenase 1-like	0.0003	down
XM_043763058.1	LOC122590877	putative receptor-like protein kinase At5g39000	< 0.0001	down
XM_043754594.1	LOC122582228	probable glycosyltransferase At5g03795	0.0018	down
XM_043757069.1	LOC122584976	receptor-like kinase TMK4	< 0.0001	down
XM_043757075.1	LOC122584981	uncharacterized LOC122584981	< 0.0001	down
XM_043763339.1	LOC122591120	receptor protein kinase CLAVATA1	0.0032	down
XM_043762685.1	LOC122590482	protein SRG1-like	< 0.0001	down
XM_043757328.1	LOC122585216	uncharacterized LOC122585216	0.0005	down
XM_043768602.1	LOC122596085	phosphate transporter PHO1 homolog 1	0.0025	down
XM_043761178.1	LOC122588954	histidine-containing phosphotransfer protein 1-like, transcript variant X4	< 0.0001	down
XM_043781077.1	LOC122607995	acyl-protein thioesterase 2-like	0.0008	down
XM_043781275.1	LOC122608185	nuclear transcription factor Y subunit B-5-like	< 0.0001	down
XM_043758086.1	LOC122585972	F-box/kelch-repeat protein At3g06240-like, transcript variant X2	0.0047	down
XM_043750023.1	LOC122578132	histone H2AX	< 0.0001	down
XM_043766413.1	LOC122593941	BURP domain protein RD22-like	0.0011	down
XM_043761554.1	LOC122589279	receptor protein-tyrosine kinase CEPR1-like	< 0.0001	down
XM_043756714.1	LOC122584670	receptor-like kinase TMK4	< 0.0001	down
XM_043765352.1	LOC122593015	probable protein phosphatase 2C 23	0.0050	down
XM_043772537.1	LOC122599921	vacuolar-processing enzyme-like	0.0002	down
XM_043753373.1	LOC122581203	uncharacterized LOC122581203	0.0002	down
XM_043778922.1	LOC122605958	UDP-glycosyltransferase 91D1-like	< 0.0001	down
XM_043756211.1	LOC122583830	calcium-binding protein CBP-like	0.0039	down
XM_043780793.1	LOC122607761	uncharacterized LOC122607761	< 0.0001	down
XM_043764918.1	LOC122592635	probable glycerol-3-phosphate acyltransferase 8	< 0.0001	down
XM_043752285.1	LOC122580017	putative 12-oxophytodienoate reductase 11, transcript variant X2	0.0046	down
XM_043769229.1	LOC122596617	2-hydroxyisoflavanone dehydratase-like	0.0003	down
XM_043771512.1	LOC122599066	disease resistance protein RPV1-like, transcript variant X1	< 0.0001	down
XM_043750232.1	LOC122578300	F-box only protein 6-like	0.0046	down
XM_043757498.1	LOC122585366	uncharacterized LOC122585366	0.0007	down
XM_043768000.1	LOC122595601	peroxidase 60-like	< 0.0001	down
XM_043766828.1	LOC122594362	UPF0496 protein At4g34320-like	< 0.0001	down
XM_043777283.1	LOC122604386	protein SRC2-like	< 0.0001	down
XM_043780650.1	LOC122607634	protein REVEILLE 1-like	0.0003	down
XM_043783468.1	LOC122610481	receptor-like kinase TMK4	0.0003	down

Transcript id	GeneID	Gene name/function	*P* Value	result
XM_043777292.1	LOC122604393	type I inositol polyphosphate 5-phosphatase 2-like	0.0031	down
XM_043756800.1	LOC122584747	extradiol ring-cleavage dioxygenase	0.0007	down
XM_043776233.1	LOC122603510	mechanosensitive ion channel protein 6-like	< 0.0001	down
XM_043768266.1	LOC122595817	scarecrow-like protein 13	< 0.0001	down
XM_043750030.1	LOC122578136	uncharacterized LOC122578136, transcript variant X1	0.0024	down
XM_043763401.1	LOC122591181	callose synthase 2-like, transcript variant X1	< 0.0001	down
XM_043766196.1	LOC122593751	transcription factor MYB17-like	< 0.0001	down
XM_043753788.1	LOC122581556	calcium-binding protein CAST-like	< 0.0001	down
XM_043769191.1	LOC122596583	protein TIFY 10A-like	0.0004	down
XM_043774982.1	LOC122602273	AAA-ATPase ASD, mitochondrial-like	0.0006	down
XM_043782792.1	LOC122609845	aluminum-activated malate transporter 10	< 0.0001	down
XM_043756074.1	LOC122583696	uncharacterized LOC122583696	< 0.0001	down
XM_043764454.1	LOC122592264	cysteine-rich receptor-like protein kinase 29	< 0.0001	down
XM_043752054.1	LOC122579819	peroxidase 12-like	0.0009	down
XM_043776230.1	LOC122603509	UBP1-associated protein 2B-like, transcript variant X1	< 0.0001	down
XM_043757948.1	LOC122585824	gibberellin 2-beta-dioxygenase 8-like	0.0031	down
XM_043775515.1	LOC122602903	uncharacterized LOC122602903	< 0.0001	down
XM_043761169.1	LOC122588950	receptor-like protein kinase FERONIA	< 0.0001	down
XM_043768411.1	LOC122595933	probable carboxylesterase 6	< 0.0001	down
XM_043767907.1	LOC122595532	probable F-box protein At2g36090	< 0.0001	down
XM_043776861.1	LOC122603985	E3 ubiquitin-protein ligase parkin, transcript variant X2	< 0.0001	down
XM_043779939.1	LOC122607021	cytochrome P450 81Q32-like	0.0003	down
XM_043762566.1	LOC122590235	deoxypodophyllotoxin synthase-like	< 0.0001	down
XM_043759243.1	LOC122587149	probable LRR receptor-like serine/threonine-protein kinase At3g47570	< 0.0001	down
XM_043767269.1	LOC122594987	pectinesterase-like	0.0056	down
XM_043780492.1	LOC122607505	probable carboxylesterase 2	0.0009	down
XM_043772339.1	LOC122599771	LRR receptor-like serine/threonine-protein kinase FEI 2, transcript variant X2	< 0.0001	down
XM_043757494.1	LOC122585363	disease resistance protein Roq1-like	0.0019	down
XM_043780955.1	LOC122607884	calcium-transporting ATPase 12, plasma membrane-type-like	< 0.0001	down
XM_043783555.1	LOC122610575	potassium transporter 6-like	< 0.0001	down
XM_043759570.1	LOC122587408	transcription factor AS1-like	< 0.0001	down
XM_043767513.1	LOC122595182	putative UPF0481 protein At3g02645	< 0.0001	down
XM_043778903.1	LOC122605938	germin-like protein subfamily 3 member 2	< 0.0001	down
XM_043752321.1	LOC122580048	glucan endo-1,3-beta-glucosidase 13-like	0.0005	down
XM_043772412.1	LOC122599825	xyloglucan 6-xylosyltransferase 2-like	< 0.0001	down
XM_043766399.1	LOC122593933	BURP domain-containing protein 3-like	< 0.0001	down
XM_043755440.1	LOC122583007	agamous-like MADS-box protein AGL62	0.0024	down
XM_043766859.1	LOC122594394	MACPF domain-containing protein NSL1	< 0.0001	down
XM_043771475.1	LOC122599030	cyclin-T1-4-like, transcript variant X2	0.0026	down
XM_043754150.1	LOC122581843	benzyl alcohol O-benzoyltransferase-like	< 0.0001	down
XM_043776294.1	LOC122603562	exocyst complex component EXO70H1-like	< 0.0001	down
XM_043767892.1	LOC122595525	protein LNK1	< 0.0001	down
XM_043753276.1	LOC122581123	chromatin modification-related protein EAF1 B-like, transcript variant X1	< 0.0001	down
XR_006323725.1	LOC122598793	uncharacterized LOC122598793	0.0001	down
XM_043751908.1	LOC122579686	brassinosteroid-responsive RING protein 1-like	0.0033	down
XM_043763119.1	LOC122590945	laccase-7-like	< 0.0001	down
XM_043775411.1	LOC122602813	phenylalanine ammonia-lyase-like	< 0.0001	down
XM_043752400.1	LOC122580130	shikimate O-hydroxycinnamoyltransferase-like	0.0053	down
XM_043782536.1	LOC122609492	protein SCAR2-like	0.0028	down
XM_043780725.1	LOC122607701	protein SOSEKI 5-like	0.0035	down
XM_043762735.1	LOC122590539	FT-interacting protein 1	< 0.0001	down
XM_043763768.1	LOC122591500	wall-associated receptor kinase 2-like	< 0.0001	down
XM_043781313.1	LOC122608226	F-box protein CPR1-like, transcript variant X1	0.0016	down
XM_043776036.1	LOC122603360	8-hydroxygeraniol dehydrogenase-like	0.0001	down
XM_043752482.1	LOC122580202	probable LRR receptor-like serine/threonine-protein kinase RPK1	0.0039	down
XM_043781812.1	LOC122608718	protein NRT1/ PTR FAMILY 1.2-like	< 0.0001	down
XM_043779163.1	LOC122606204	uncharacterized LOC122606204	0.0032	down
XR_006324852.1	LOC122606293	uncharacterized LOC122606293	0.0028	down
XM_043774887.1	LOC122602162	3-hydroxy-3-methylglutaryl coenzyme A reductase 1-like	0.0011	down
XM_043760802.1	LOC122588646	probable adenylate kinase 7, mitochondrial	< 0.0001	down
XM_043760149.1	LOC122587972	ankyrin repeat-containing protein NPR4-like, transcript variant X1	0.0001	down
XM_043780473.1	LOC122607493	protein PHOSPHATE-INDUCED 1-like	< 0.0001	down

Transcript id	GeneID	Gene name/function	*P* Value	result
XM_043768116.1	LOC122595692	uncharacterized LOC122595692	0.0002	down
XM_043757643.1	LOC122585517	cationic peroxidase 1-like	0.0004	down
XM_043770383.1	LOC122597808	peroxidase N1-like	< 0.0001	down
XM_043752754.1	LOC122580480	probable serine/threonine-protein kinase At1g54610	0.0026	down
XM_043767175.1	LOC122594874	probable alpha,alpha-trehalose-phosphate synthase [UDP-forming] 7	0.0013	down
XM_043773860.1	LOC122601086	probable CoA ligase CCL5	< 0.0001	down
XM_043762219.1	LOC122589889	uncharacterized LOC122589889	0.0003	down
XM_043756380.1	LOC122584015	probable protein phosphatase 2C 25	< 0.0001	down
XM_043779951.1	LOC122607032	probable glycerol-3-phosphate acyltransferase 3	< 0.0001	down
XM_043766070.1	LOC122593641	transcription initiation factor TFIID subunit 11-like	< 0.0001	down
XM_043784039.1	LOC122611077	polyubiquitin 11-like	< 0.0001	down
XM_043784398.1	LOC122611374	putative late blight resistance protein homolog R1A-10, transcript variant X1	< 0.0001	down
XM_043758217.1	LOC122586109	uncharacterized LOC122586109, transcript variant X1	0.0004	down
XM_043777921.1	LOC122605048	thaumatin-like protein 1b	0.0001	down
XM_043768436.1	LOC122595950	probable protein phosphatase 2C 59, transcript variant X3	< 0.0001	down
XM_043782121.1	LOC122609059	probable calcium-binding protein CML31	< 0.0001	down
XM_043755541.1	LOC122583105	MDIS1-interacting receptor like kinase 2-like	0.0006	down
XM_043767361.1	LOC122595068	protein SUPPRESSOR OF MAX2 1	< 0.0001	down
XM_043760564.1	LOC122588447	uncharacterized LOC122588447	0.0012	down
XR_006322008.1	LOC122586472	uncharacterized LOC122586472	< 0.0001	down
XM_043770357.1	LOC122597788	F-box/kelch-repeat protein At3g23880-like, transcript variant X1	< 0.0001	down
XM_043773922.1	LOC122601153	receptor-like protein kinase FERONIA	0.0047	down
XM_043772535.1	LOC122599919	protein LNK1-like	< 0.0001	down
XM_043766805.1	LOC122594326	serine/threonine-protein kinase-like protein ACR4	< 0.0001	down
XM_043779421.1	LOC122606581	hyoscyamine 6-dioxygenase-like	< 0.0001	down
XM_043779053.1	LOC122606094	SKP1-like protein 21, transcript variant X2	< 0.0001	down
XM_043762189.1	LOC122589867	4-coumarate--CoA ligase 2-like	0.0002	down
XM_043761379.1	LOC122589137	transcription factor MYB25-like	0.0011	down
XM_043769310.1	LOC122596690	subtilisin-like protease SBT6.1, transcript variant X3	0.0013	down
XM_043755528.1	LOC122583090	phospholipase A1-IIgamma	0.0004	down
XM_043779503.1	LOC122606664	receptor-like protein kinase FERONIA	< 0.0001	down
XR_006322646.1	LOC122590931	uncharacterized LOC122590931, transcript variant X1	0.0063	down
XR_006324666.1	LOC122605649	uncharacterized LOC122605649	< 0.0001	down
XM_043784302.1	LOC122611298	metal tolerance protein 1-like, transcript variant X1	0.0004	down
XM_043782427.1	LOC122609380	probable inorganic phosphate transporter 1-7	< 0.0001	down
XM_043750944.1	LOC122578892	probable E3 ubiquitin-protein ligase XERICO	< 0.0001	down
XM_043770981.1	LOC122598386	lysine histidine transporter-like 8	< 0.0001	down
XM_043761467.1	LOC122589216	protein RALF-like 34	0.0007	down
XM_043757685.1	LOC122585555	AT-hook motif nuclear-localized protein 24	< 0.0001	down
XM_043752513.1	LOC122580237	cytochrome P450 87A3-like	0.0012	down
XM_043757832.1	LOC122585708	uncharacterized LOC122585708	< 0.0001	down
XM_043754804.1	LOC122582410	AT-hook motif nuclear-localized protein 10-like	0.0060	down
XM_043761962.1	LOC122589645	protein NRT1/ PTR FAMILY 4.4	< 0.0001	down
XM_043774799.1	LOC122602068	G-type lectin S-receptor-like serine/threonine-protein kinase At1g34300	0.0009	down
XM_043762092.1	LOC122589769	polygalacturonase QRT3-like	0.0003	down
XM_043751978.1	LOC122579749	7-ethoxycoumarin O-deethylase-like	< 0.0001	down
XM_043768392.1	LOC122595914	metalloendoproteinase 5-MMP-like	0.0015	down
XM_043768100.1	LOC122595679	probable protein phosphatase 2C 63	0.0002	down
XM_043759495.1	LOC122587355	uncharacterized LOC122587355	< 0.0001	down
XM_043750389.1	LOC122578425	RHOMBOID-like protein 2	< 0.0001	down
XM_043759481.1	LOC122587344	BTB/POZ and TAZ domain-containing protein 4-like, transcript variant X2	0.0009	down
XM_043777023.1	LOC122604122	F-box protein CPR1-like	0.0016	down
XM_043752646.1	LOC122580374	WAT1-related protein At1g43650	0.0017	down
XM_043775250.1	LOC122602653	phosphoinositide phospholipase C 6-like	< 0.0001	down
XM_043772900.1	LOC122600220	uncharacterized LOC122600220, transcript variant X1	< 0.0001	down
XM_043781078.1	LOC122607998	acyl-coenzyme A thioesterase 13-like	< 0.0001	down
XM_043775047.1	LOC122602349	protein DMP3-like	< 0.0001	down
XM_043752435.1	LOC122580163	protein EDS1-like	0.0001	down
XM_043778068.1	LOC122605173	E3 ubiquitin-protein ligase PUB23-like	< 0.0001	down
XM_043779028.1	LOC122606067	probable indole-3-acetic acid-amido synthetase GH3.1	< 0.0001	down
XM_043752333.1	LOC122580062	extensin-3-like	< 0.0001	down
XM_043754615.1	LOC122582248	WAT1-related protein At5g07050	< 0.0001	down

Transcript id	GeneID	Gene name/function	*P* Value	result
XM_043758823.1	LOC122586828	serine/threonine-protein kinase AFC2, transcript variant X1	< 0.0001	down
XM_043765030.1	LOC122592736	REF/SRPP-like protein At3g05500	< 0.0001	down
XM_043767080.1	LOC122594650	uncharacterized LOC122594650	< 0.0001	down
XM_043770952.1	LOC122598360	CSC1-like protein At3g54510	< 0.0001	down
XM_043767890.1	LOC122595522	protein CELLULOSE SYNTHASE INTERACTIVE 1	< 0.0001	down
XM_043759425.1	LOC122587298	nudix hydrolase 17, mitochondrial-like	0.0006	down
XM_043770305.1	LOC122597737	peroxidase N1-like	< 0.0001	down
XM_043777043.1	LOC122604145	receptor protein kinase CLAVATA1-like	0.0015	down
XM_043769740.1	LOC122597104	AP2/ERF domain-containing protein PFD0985w-like	0.0042	down
XM_043751238.1	LOC122579131	NDR1/HIN1-like protein 6	0.0046	down
XM_043784145.1	LOC122611170	glycerate dehydrogenase	< 0.0001	down
XM_043779166.1	LOC122606207	subtilisin-like protease SBT1.7	< 0.0001	down
XM_043776739.1	LOC122603893	scarecrow-like protein 8	< 0.0001	down
XM_043778750.1	LOC122605796	S-adenosylmethionine decarboxylase proenzyme-like	0.0021	down
XM_043762332.1	LOC122589999	RING-H2 finger protein ATL8-like	0.0002	down
XM_043777411.1	LOC122604524	uncharacterized LOC122604524	< 0.0001	down
XM_043775158.1	LOC122602556	peroxidase N-like	< 0.0001	down
XM_043776783.1	LOC122603926	transcription factor MYB73-like	< 0.0001	down
XM_043769882.1	LOC122597267	serine carboxypeptidase-like 40	0.0002	down
XM_043750713.1	LOC122578694	uncharacterized LOC122578694	< 0.0001	down
XM_043757756.1	LOC122585629	laccase-7-like	< 0.0001	down
XM_043755138.1	LOC122582715	probable protein phosphatase 2C 25	< 0.0001	down
XM_043778935.1	LOC122605971	ethylene-responsive transcription factor ERF027-like	< 0.0001	down
XM_043766503.1	LOC122594027	protein GLUTAMINE DUMPER 2-like	< 0.0001	down
XM_043759125.1	LOC122587054	LEAF RUST 10 DISEASE-RESISTANCE LOCUS RECEPTOR-LIKE PROTEIN KINASE-like 1.4, transcript variant X2	0.0024	down
XM_043762157.1	LOC122589836	E3 ubiquitin-protein ligase WAV3-like	0.0015	down
XM_043755005.1	LOC122582591	MACPF domain-containing protein At4g24290-like	< 0.0001	down
XM_043750408.1	LOC122578441	uncharacterized LOC122578441, transcript variant X2	< 0.0001	down
XM_043755019.1	LOC122582605	probable boron transporter 6	< 0.0001	down
XM_043761370.1	LOC122589125	agamous-like MADS-box protein AGL12	< 0.0001	down
XM_043767574.1	LOC122595241	C2 domain-containing protein At1g53590-like	< 0.0001	down
XM_043762573.1	LOC122590242	thiol:disulfide interchange protein TxlA homolog	< 0.0001	down
XM_043770972.1	LOC122598379	probable carboxylesterase 7	< 0.0001	down
XM_043784189.1	LOC122611206	glycine cleavage system H protein, mitochondrial	< 0.0001	down
XM_043772269.1	LOC122599716	probable polyamine oxidase 5	0.0027	down
XM_043764991.1	LOC122592701	pleiotropic drug resistance protein 3-like, transcript variant X1	0.0008	down
XM_043759421.1	LOC122587295	palmitoyl-monogalactosyldiacylglycerol delta-7 desaturase, chloroplastic-like	< 0.0001	down
XM_043759958.1	LOC122587792	uncharacterized LOC122587792	0.0030	down
XM_043756900.1	LOC122584836	S-adenosylmethionine decarboxylase proenzyme-like	< 0.0001	down
XM_043781651.1	LOC122608552	molybdate transporter 1	0.0012	down
XM_043778014.1	LOC122605135	protein ROOT HAIR DEFECTIVE 3-like	< 0.0001	down
XM_043762217.1	LOC122589878	protein ANTI-SILENCING 1, transcript variant X2	0.0008	down
XM_043757463.1	LOC122585335	ras-related protein RABC2a-like	0.0004	down
XM_043759964.1	LOC122587798	U-box domain-containing protein 40	< 0.0001	down
XM_043759760.1	LOC122587580	inositol hexakisphosphate and diphosphoinositol-pentakisphosphate kinase VIP2-like	0.0017	down
XM_043775124.1	LOC122602489	3-ketoacyl-CoA thiolase 2, peroxisomal-like	0.0011	down
XM_043784037.1	LOC122611075	floral homeotic protein APETALA 2-like, transcript variant X3	0.0038	down
XM_043779238.1	LOC122606277	protein SIEVE ELEMENT OCCLUSION B-like	0.0007	down
XM_043779008.1	LOC122606044	caffeoyl-CoA O-methyltransferase-like	< 0.0001	down
XM_043765822.1	LOC122593413	auxin-responsive protein IAA11-like	< 0.0001	down
XM_043764293.1	LOC122592086	cytochrome P450 94A2-like	0.0019	down
XM_043757279.1	LOC122585172	BOI-related E3 ubiquitin-protein ligase 1-like, transcript variant X2	< 0.0001	down
XM_043776045.1	LOC122603367	putative lipid-transfer protein DIR1	< 0.0001	down
XM_043761886.1	LOC122589581	subtilisin-like protease SBT1.7	< 0.0001	down
XM_043783213.1	LOC122610219	histidine kinase 1-like	0.0003	down
XM_043758389.1	LOC122586396	histone chaperone RTT106-like	0.0052	down
XM_043755438.1	LOC122583001	CBL-interacting serine/threonine-protein kinase 7-like	< 0.0001	down
XM_043765821.1	LOC122593411	cation/calcium exchanger 4-like	0.0036	down
XM_043773734.1	LOC122600957	zinc transporter 1	< 0.0001	down
XM_043760342.1	LOC122588218	UPF0481 protein At3g47200-like	0.0002	down
XM_043781604.1	LOC122608502	uncharacterized LOC122608502	< 0.0001	down
XM_043758220.1	LOC122586112	transcription repressor OFP6-like	0.0006	down

Transcript id	GeneID	Gene name/function	P Value	result
XM_043750704.1	LOC122578686	alcohol dehydrogenase-like 3	< 0.0001	down
XM_043756248.1	LOC122583874	chaperone protein dnaJ 20, chloroplastic-like	< 0.0001	down
XM_043755020.1	LOC122582606	protein NRT1/ PTR FAMILY 7.2-like	0.0008	down
XM_043765259.1	LOC122592926	probable carboxylesterase 12	< 0.0001	down
XM_043775494.1	LOC122602884	scarecrow-like protein 9, transcript variant X3	0.0047	down
XM_043779727.1	LOC122606850	phospholipase D alpha 1	0.0027	down
XM_043773258.1	LOC122600530	L-type lectin-domain containing receptor kinase IX.1-like	0.0026	down
XM_043779472.1	LOC122606633	uncharacterized LOC122606633	< 0.0001	down
XM_043754969.1	LOC122582562	signal recognition particle receptor FtsY-like	0.0031	down
XM_043750412.1	LOC122578443	photosynthetic NDH subunit of lumenal location 3, chloroplastic	0.0004	down
XM_043753432.1	LOC122581249	protein TRANSPARENT TESTA GLABRA 1	< 0.0001	down
XM_043776866.1	LOC122603990	endochitinase EP3-like	0.0012	down
XM_043772493.1	LOC122599888	abscisic acid 8'-hydroxylase CYP707A2-like	0.0031	down
XM_043772094.1	LOC122599572	myb family transcription factor APL	< 0.0001	down
XM_043779390.1	LOC122606543	(S)-8-oxocitronellyl enol synthase CYC2	0.0003	down
XM_043774954.1	LOC122602243	UDP-glycosyltransferase 74G1-like	0.0001	down
XM_043782843.1	LOC122609892	uncharacterized LOC122609892	< 0.0001	down
XM_043763567.1	LOC122591313	beta-amylase 2, chloroplastic	0.0029	down
XM_043777470.1	LOC122604587	monosaccharide-sensing protein 2, transcript variant X2	< 0.0001	down
XM_043762094.1	LOC122589772	cryptochrome-1, transcript variant X1	0.0013	down
XM_043755317.1	LOC122582887	benzyl alcohol O-benzoyltransferase-like	< 0.0001	down
XM_043781714.1	LOC122608620	transcription factor GTE10-like	< 0.0001	down
XM_043761811.1	LOC122589511	protein TAPETUM DETERMINANT 1-like	< 0.0001	down
XM_043772478.1	LOC122599869	anthranilate synthase beta subunit 2, chloroplastic-like, transcript variant X2	0.0031	down
XM_043767826.1	LOC122595463	ELMO domain-containing protein C-like, transcript variant X1	< 0.0001	down
XM_043771196.1	LOC122598610	1-aminocyclopropane-1-carboxylate oxidase homolog 3-like	0.0034	down
XM_043773301.1	LOC122600565	uncharacterized LOC122600565	< 0.0001	down
XM_043758654.1	LOC122586672	low affinity sulfate transporter 3-like, transcript variant X2	< 0.0001	down
XM_043771031.1	LOC122598439	cytochrome P450 72A397-like	< 0.0001	down
XM_043763541.1	LOC122591300	plasma membrane ATPase 4	< 0.0001	down
XM_043751386.1	LOC122579259	protein KINESIN LIGHT CHAIN-RELATED 2-like, transcript variant X1	< 0.0001	down
XM_043755619.1	LOC122583195	desmethyl-deoxy-podophyllotoxin synthase-like	< 0.0001	down
XM_043765728.1	LOC122593333	WRKY transcription factor WRKY51-like	< 0.0001	down
XM_043760142.1	LOC122587996	amino acid transporter AVT6C-like	< 0.0001	down
XM_043766557.1	LOC122594081	zinc finger protein ZAT11-like	0.0025	down
XM_043755346.1	LOC122582916	serine/threonine-protein kinase PCRK1-like	< 0.0001	down
XM_043762414.1	LOC122590081	protein GLUTAMINE DUMPER 6-like	0.0057	down
XM_043762111.1	LOC122589787	protein IQ-DOMAIN 8-like	< 0.0001	down
XM_043776420.1	LOC122603653	18.1 kDa class I heat shock protein-like	< 0.0001	down
XM_043781502.1	LOC122608402	uncharacterized LOC122608402, transcript variant X1	< 0.0001	down
XM_043761102.1	LOC122588883	solute carrier family 25 member 44	< 0.0001	down
XM_043760546.1	LOC122588436	uncharacterized LOC122588436	0.0001	down
XM_043775416.1	LOC122602819	uncharacterized LOC122602819, transcript variant X1	< 0.0001	down
XM_043766138.1	LOC122593693	leucine-rich repeat extensin-like protein 3	0.0013	down
XM_043781172.1	LOC122608085	glucuronoxylan 4-O-methyltransferase 3-like	0.0047	down
XM_043763587.1	LOC122591327	ultraviolet-B receptor UVR8	< 0.0001	down
XM_043753706.1	LOC122581475	NAD(H) kinase 1	< 0.0001	down
XM_043770062.1	LOC122597472	probable carboxylesterase 5	0.0002	down
XM_043751197.1	LOC122579097	uncharacterized LOC122579097	< 0.0001	down
XM_043770313.1	LOC122597743	glutamate receptor 2.7-like	0.0002	down
XM_043753205.1	LOC122581064	probable serine/threonine-protein kinase WNK4, transcript variant X1	< 0.0001	down
XM_043767933.1	LOC122595552	nodulin-related protein 1-like	0.0003	down
XM_043757754.1	LOC122585627	pre-mRNA splicing factor SR-like 1, transcript variant X4	0.0004	down
XM_043755337.1	LOC122582908	adenylate isopentenyltransferase 3, chloroplastic-like	< 0.0001	down
XM_043774361.1	LOC122601612	TMV resistance protein N-like	0.0033	down
XM_043753604.1	LOC122581377	ACT domain-containing protein ACR8-like	< 0.0001	down
XM_043769164.1	LOC122596558	NDR1/HIN1-like protein 6	< 0.0001	down
XM_043777273.1	LOC122604378	beta-fructofuranosidase, soluble isoenzyme I-like, transcript variant X1	0.0030	down
XM_043755202.1	LOC122582768	uncharacterized LOC122582768, transcript variant X3	0.0007	down
XR_006321322.1	LOC122583751	uncharacterized LOC122583751, transcript variant X2	< 0.0001	down
XM_043778701.1	LOC122605737	annexin D5-like, transcript variant X1	< 0.0001	down

Transcript id	GeneID	Gene name/function	P Value	result
XM_043775134.1	LOC122602529	zinc finger protein BALDIBIS-like	0.0021	down
XM_043770719.1	LOC122598114	calcium-dependent protein kinase 26-like	< 0.0001	down
XM_043777868.1	LOC122605006	calcium-dependent protein kinase 14	0.0004	down
XM_043777417.1	LOC122604531	receptor-like protein EIX2	0.0026	down
XM_043779905.1	LOC122606989	aldehyde oxidase GLOX	< 0.0001	down
XM_043767904.1	LOC122595531	calcium homeostasis endoplasmic reticulum protein, transcript variant X2	0.0014	down
XM_043762559.1	LOC122590224	uncharacterized LOC122590224	0.0016	down
XR_006323844.1	LOC122599023	uncharacterized LOC122599023	< 0.0001	down
XM_043757555.1	LOC122585422	cationic peroxidase 1-like	0.0018	down
XM_043758570.1	LOC122586585	mitogen-activated protein kinase kinase kinase 20-like	< 0.0001	down
XM_043784025.1	LOC122611068	RHOMBOID-like protein 2	< 0.0001	down
XM_043773513.1	LOC122600757	cytochrome b561 and DOMON domain-containing protein At5g35735-like	< 0.0001	down
XM_043751042.1	LOC122578969	lectin-like, transcript variant X2	< 0.0001	down
XM_043783735.1	LOC122610777	basic leucine zipper 43-like	< 0.0001	down
XR_006324430.1	LOC122602678	uncharacterized LOC122602678, transcript variant X1	< 0.0001	down
XM_043755813.1	LOC122583407	AT-hook motif nuclear-localized protein 23-like	< 0.0001	down
XM_043757493.1	LOC122585362	putative F-box protein PP2-B12	0.0004	down
XM_043765877.1	LOC122593464	xyloglucan endotransglucosylase/hydrolase 2-like	0.0014	down
XM_043761409.1	LOC122589160	probable sulfate transporter 3.4	< 0.0001	down
XM_043751268.1	LOC122579157	eukaryotic peptide chain release factor subunit 1-3, transcript variant X1	< 0.0001	down
XM_043780488.1	LOC122607502	protein LURP-one-related 15-like	< 0.0001	down
XM_043759437.1	LOC122587307	mannose-6-phosphate isomerase 1-like	0.0058	down
XM_043780803.1	LOC122607766	transcription factor BIM1-like, transcript variant X1	0.0001	down
XM_043767854.1	LOC122595484	protein GL2-INTERACTING REPRESSOR 1-like	0.0054	down
XM_043770889.1	LOC122598296	heat stress transcription factor C-1-like	0.0009	down
XM_043781742.1	LOC122608651	uncharacterized LOC122608651	< 0.0001	down
XM_043770895.1	LOC122598304	probable galacturonosyltransferase-like 9	< 0.0001	down
XM_043750826.1	LOC122578788	xyloglucan glycosyltransferase 4-like	< 0.0001	down
XM_043782332.1	LOC122609277	laccase-13-like	0.0017	down
XM_043757443.1	LOC122585316	E3 ubiquitin-protein ligase RDUF2-like	< 0.0001	down
XM_043750521.1	LOC122578544	galactan beta-1,4-galactosyltransferase GALS3-like	0.0001	down
XM_043771305.1	LOC122598818	probable WRKY transcription factor 53	< 0.0001	down
XM_043769282.1	LOC122596663	ethylene-responsive transcription factor 3-like	0.0001	down
XM_043774784.1	LOC122602051	calcium-binding protein CML42-like	0.0002	down
XM_043763728.1	LOC122591460	protein GLUTAMINE DUMPER 3-like	0.0028	down
XM_043772169.1	LOC122599632	uncharacterized protein At1g76660-like	< 0.0001	down
XM_043768930.1	LOC122596364	nicotianamine synthase-like	< 0.0001	down
XR_006323765.1	LOC122598873	uncharacterized LOC122598873, transcript variant X1	0.0015	down
XM_043752901.1	LOC122580633	homeobox-leucine zipper protein HDG11-like	0.0054	down
XM_043756239.1	LOC122583864	uncharacterized LOC122583864	0.0029	down
XM_043762906.1	LOC122590726	chaperone protein dnaJ 11, chloroplastic-like	< 0.0001	down
XM_043775399.1	LOC122602803	cation/H(+) antiporter 20	< 0.0001	down
XM_043769052.1	LOC122596473	calcium-transporting ATPase 10, plasma membrane-type-like, transcript variant X2	0.0001	down
XM_043762182.1	LOC122589860	uroporphyrinogen-III synthase, chloroplastic, transcript variant X1	0.0022	down
XM_043750358.1	LOC122578402	plasmodesmata-located protein 6-like, transcript variant X1	< 0.0001	down
XR_006320699.1	LOC122579755	uncharacterized LOC122579755, transcript variant X7	0.0017	down
XM_043766631.1	LOC122594159	protein NRT1/ PTR FAMILY 5.5-like	0.0004	down
XM_043752415.1	LOC122580141	protein MIZU-KUSSEI 1-like	0.0037	down
XM_043773140.1	LOC122600429	endoplasmic reticulum oxidoreductin-1-like	< 0.0001	down
XM_043761069.1	LOC122588853	triacylglycerol lipase OBL1-like	< 0.0001	down
XM_043777097.1	LOC122604202	uncharacterized LOC122604202	< 0.0001	down
XM_043761576.1	LOC122589299	dof zinc finger protein DOF1.7-like	< 0.0001	down
XM_043762506.1	LOC122590173	UDP-glycosyltransferase 85C2-like	0.0005	down
XM_043750774.1	LOC122578747	phosphoglycerate mutase-like protein AT74H	< 0.0001	down
XM_043750796.1	LOC122578764	delta(12) fatty acid desaturase DES8.11-like	0.0031	down
XM_043750706.1	LOC122578684	GDSL esterase/lipase At4g10955-like	< 0.0001	down
XM_043760612.1	LOC122588483	nuclear poly(A) polymerase 4-like, transcript variant X3	0.0003	down
XM_043754831.1	LOC122582442	clavaminate synthase-like protein At3g21360	0.0031	down
XM_043774985.1	LOC122602276	uncharacterized LOC122602276	0.0030	down
XM_043768744.1	LOC122596209	protein TIFY 7-like	0.0040	down
XM_043751327.1	LOC122579210	NAC domain-containing protein 83-like	< 0.0001	down

Transcript id	GeneID	Gene name/function	P Value	result
XM_043773111.1	LOC122600401	SNF1-related protein kinase regulatory subunit beta-2-like	0.0002	down
XM_043776577.1	LOC122603776	xyloglucan galactosyltransferase XLT2	0.0004	down
XM_043782429.1	LOC122609382	probable indole-3-pyruvate monooxygenase YUCCA10	< 0.0001	down
XM_043765448.1	LOC122593096	heptahelical transmembrane protein 1-like	< 0.0001	down
XM_043770739.1	LOC122598137	F-box protein SNE-like	0.0012	down
XM_043771504.1	LOC122599055	1-aminocyclopropane-1-carboxylate oxidase homolog 1-like	< 0.0001	down
XM_043753312.1	LOC122581145	lysine histidine transporter-like 8	< 0.0001	down
XM_043770644.1	LOC122598040	lysine histidine transporter-like 6	< 0.0001	down
XM_043778731.1	LOC122605775	putative lipid-transfer protein DIR1	< 0.0001	down
XM_043760197.1	LOC122588053	F-box protein At5g07610-like	0.0008	down
XM_043758037.1	LOC122585920	protein ALWAYS EARLY 3, transcript variant X1	< 0.0001	down
XM_043766931.1	LOC122594484	U-box domain-containing protein 13-like	< 0.0001	down
XM_043761104.1	LOC122588884	aspartyl protease family protein At5g10770-like	0.0011	down
XM_043762098.1	LOC122589775	cytochrome P450 81Q32-like	< 0.0001	down
XM_043754039.1	LOC122581764	zinc finger A20 and AN1 domain-containing stress-associated protein 5	0.0036	down
XM_043782745.1	LOC122609806	(-)-isopiperitenol/(-)-carveol dehydrogenase, mitochondrial-like	< 0.0001	down
XM_043763324.1	LOC122591113	beta-amylase 7, transcript variant X2	< 0.0001	down
XM_043776904.1	LOC122604020	type I inositol polyphosphate 5-phosphatase 10	0.0056	down
XR_006322100.1	LOC122587043	uncharacterized LOC122587043	< 0.0001	down
XM_043760607.1	LOC122588480	amino acid transporter AVT6A-like	0.0001	down
XR_006321955.1	LOC122586330	uncharacterized LOC122586330	< 0.0001	down
XM_043761179.1	LOC122588958	probable inactive receptor kinase At5g58300, transcript variant X2	0.0001	down
XM_043774712.1	LOC122601981	protein DOG1-like 3, transcript variant X2	0.0010	down
XR_006320814.1	LOC122580529	uncharacterized LOC122580529	< 0.0001	down
XM_043771806.1	LOC122599310	ABC transporter C family member 3-like	0.0034	down
XM_043771315.1	LOC122598829	ankyrin repeat-containing protein NPR4-like	0.0022	down
XM_043758828.1	LOC122586831	low affinity sulfate transporter 3-like, transcript variant X1	< 0.0001	down
XM_043768835.1	LOC122596283	F-box/kelch-repeat protein At3g06240-like	0.0021	down
XM_043782878.1	LOC122609931	type I inositol polyphosphate 5-phosphatase 2, transcript variant X1	< 0.0001	down
XM_043751362.1	LOC122579241	chaperone protein dnaJ 10	0.0004	down
XM_043776694.1	LOC122603861	wound-induced protein 1	< 0.0001	down
XM_043779044.1	LOC122606086	E3 ubiquitin-protein ligase RNFT1-like, transcript variant X1	0.0002	down
XM_043760354.1	LOC122588232	vegetative cell wall protein gp1-like	< 0.0001	down
XM_043765987.1	LOC122593561	macrophage migration inhibitory factor homolog	< 0.0001	down
XM_043782789.1	LOC122609842	fasciclin-like arabinogalactan protein 21	0.0025	down
XM_043780084.1	LOC122607159	GEM-like protein 1	< 0.0001	down
XM_043757751.1	LOC122585626	protein downstream neighbor of Son-like	0.0036	down
XM_043778816.1	LOC122605859	serine/threonine-protein kinase UCN-like	< 0.0001	down
XM_043755326.1	LOC122582898	zeatin O-xylosyltransferase-like	< 0.0001	down
XM_043775076.1	LOC122602387	flowering-promoting factor 1-like protein 3	< 0.0001	down
XM_043784100.1	LOC122611128	protein TIFY 7-like, transcript variant X2	< 0.0001	down
XM_043771908.1	LOC122599400	probable carotenoid cleavage dioxygenase 4, chloroplastic	< 0.0001	down
XM_043780001.1	LOC122607082	uncharacterized LOC122607082	< 0.0001	down
XM_043781744.1	LOC122608652	protein MIZU-KUSSEI 1-like	0.0050	down
XM_043773062.1	LOC122600353	phospholipid-transporting ATPase 3-like, transcript variant X1	0.0006	down
XM_043768870.1	LOC122596318	WAT1-related protein At1g21890-like	< 0.0001	down
XM_043754175.1	LOC122581864	dirigent protein 21-like	0.0002	down
XM_043756091.1	LOC122583711	WAT1-related protein At2g39510-like	0.0040	down
XM_043755673.1	LOC122583248	non-specific lipid transfer protein GPI-anchored 5-like	0.0002	down
XM_043753266.1	LOC122581115	zeatin O-glucosyltransferase-like	0.0002	down
XM_043762284.1	LOC122589953	cytokinin hydroxylase	< 0.0001	down
XM_043780919.1	LOC122607853	ethylene-responsive transcription factor CRF1-like	0.0001	down
XM_043761559.1	LOC122589285	zinc finger protein ZAT10-like	0.0003	down
XM_043783746.1	LOC122610787	cysteine protease ATG4-like	< 0.0001	down
XM_043752677.1	LOC122580411	uncharacterized LOC122580411	< 0.0001	down
XM_043759486.1	LOC122587349	cytochrome b5-like	0.0041	down
XM_043769407.1	LOC122596777	probable indole-3-acetic acid-amido synthetase GH3.1	< 0.0001	down
XM_043761194.1	LOC122588968	cytochrome P450 78A5-like	< 0.0001	down
XM_043775243.1	LOC122602644	germin-like protein 9-3	< 0.0001	down
XM_043750887.1	LOC122578840	probable boron transporter 6	0.0001	down
XM_043753834.1	LOC122581591	GDSL esterase/lipase At5g03610-like	< 0.0001	down
XM_043751741.1	LOC122579552	beta-galactosidase 3-like	0.0055	down
XM_043761080.1	LOC122588864	inorganic pyrophosphatase 2-like	0.0007	down
XM_043751530.1	LOC122579365	aspartyl protease family protein 2-like	< 0.0001	down

Transcript id	GeneID	Gene name/function	P Value	result
XM_043765701.1	LOC122593308	zinc finger protein ZAT10-like	< 0.0001	down
XM_043773096.1	LOC122600386	berberine bridge enzyme-like 15	0.0058	down
XM_043780223.1	LOC122607283	probable galacturonosyltransferase-like 1	0.0046	down
XM_043783411.1	LOC122610418	23 kDa jasmonate-induced protein-like	< 0.0001	down
XM_043772310.1	LOC122599749	putative gamma-glutamylcyclotransferase At3g02910	< 0.0001	down
XM_043780768.1	LOC122607739	transmembrane protein DDB_G0273707/DDB_G0273361-like	0.0054	down
XM_043783439.1	LOC122610448	uncharacterized LOC122610448	< 0.0001	down
XM_043755924.1	LOC122583530	putative disease resistance RPP13-like protein 1	< 0.0001	down
XM_043761131.1	LOC122588913	probable fructokinase-5	0.0022	down
XM_043783963.1	LOC122611012	uncharacterized LOC122611012	0.0014	down
XM_043778436.1	LOC122605481	chaperone protein dnaJ 20, chloroplastic-like	0.0026	down
XM_043757811.1	LOC122585689	heavy metal-associated isoprenylated plant protein 20-like	< 0.0001	down
XM_043759159.1	LOC122587085	monocopper oxidase-like protein SKS1	< 0.0001	down
XM_043773947.1	LOC122601180	putative phospholipid-transporting ATPase 9	< 0.0001	down
XM_043768786.1	LOC122596239	uncharacterized LOC122596239	< 0.0001	down
XM_043775389.1	LOC122602792	squalene epoxidase 3-like, transcript variant X2	< 0.0001	down
XM_043755686.1	LOC122583262	uncharacterized LOC122583262	< 0.0001	down
XM_043754623.1	LOC122582256	ankyrin repeat-containing protein At5g02620-like	0.0002	down
XM_043763927.1	LOC122591676	putative UPF0481 protein At3g02645	0.0002	down
XM_043775897.1	LOC122603241	xanthoxin dehydrogenase-like, transcript variant X2	0.0017	down
XM_043776939.1	LOC122604045	putative kinase-like protein TMKL1	0.0052	down
XM_043750549.1	LOC122578566	probable protein phosphatase 2C 10, transcript variant X1	0.0037	down
XM_043774312.1	LOC122601559	sodium/calcium exchanger NCL-like	0.0052	down
XM_043762587.1	LOC122590256	ethylene-responsive transcription factor ERF019	0.0002	down
XM_043763025.1	LOC122590849	mitogen-activated protein kinase kinase kinase 18-like	< 0.0001	down
XR_006320710.1	LOC122579897	trihelix transcription factor GT-4, transcript variant X4	0.0057	down
XM_043783835.1	LOC122610878	uncharacterized LOC122610878	< 0.0001	down
XM_043757146.1	LOC122585048	ABC transporter G family member 14-like	< 0.0001	down
XM_043771105.1	LOC122598510	chaperone protein dnaJ 49-like	0.0003	down
XM_043759136.1	LOC122587064	IAA-amino acid hydrolase ILR1-like 6	< 0.0001	down
XM_043758100.1	LOC122585986	(S)-8-oxocitronellyl enol synthase CYC2-like	0.0003	down
XM_043767563.1	LOC122595227	3-ketoacyl-CoA synthase 10	0.0016	down
XM_043755325.1	LOC122582896	probable glucuronoxylan glucuronosyltransferase IRX7	< 0.0001	down
XM_043756150.1	LOC122583747	ACT domain-containing protein ACR10-like	< 0.0001	down
XM_043753414.1	LOC122581237	syntaxin-121-like	0.0045	down
XM_043758029.1	LOC122585911	FT-interacting protein 7	< 0.0001	down
XM_043763309.1	LOC122591104	UDP-glucuronic acid decarboxylase 2-like	< 0.0001	down
XM_043778021.1	LOC122605141	guanosine deaminase	< 0.0001	down
XM_043775057.1	LOC122602358	dirigent protein 6-like	0.0017	down
XM_043758990.1	LOC122586950	U-box domain-containing protein 4, transcript variant X4	< 0.0001	down
XM_043765409.1	LOC122593066	copper transporter 5-like	0.0023	down
XM_043757204.1	LOC122585106	uncharacterized LOC122585106, transcript variant X2	0.0004	down
XM_043751726.1	LOC122579538	protein GIGANTEA, transcript variant X2	0.0033	down
XM_043780201.1	LOC122607265	adenylate isopentenyltransferase 3, chloroplastic	< 0.0001	down
XM_043765192.1	LOC122592873	ethylene-responsive transcription factor 4-like	< 0.0001	down
XR_006322884.1	LOC122593829	uncharacterized LOC122593829, transcript variant X2	< 0.0001	down
XM_043770619.1	LOC122598015	cucumber peeling cupredoxin-like	< 0.0001	down
XM_043751982.1	LOC122579753	thioredoxin H-type-like	< 0.0001	down
XM_043762197.1	LOC122589871	allene oxide cyclase, chloroplastic-like, transcript variant X4	0.0038	down
XM_043777892.1	LOC122605025	ras-related protein RHN1, transcript variant X2	0.0002	down
XM_043758386.1	LOC122586391	dehydration-responsive element-binding protein 1D-like	< 0.0001	down
XM_043756389.1	LOC122584027	F-box protein FBW2-like	0.0008	down
XM_043776877.1	LOC122603995	serine/threonine-protein kinase PCRK1-like	< 0.0001	down
XM_043762291.1	LOC122589962	exocyst complex component EXO70B1	< 0.0001	down
XM_043758085.1	LOC122585971	proline-rich receptor-like protein kinase PERK1	< 0.0001	down
XM_043777622.1	LOC122604750	S-adenosylmethionine synthase 3-like	< 0.0001	down
XM_043756128.1	LOC122583754	uncharacterized LOC122583754	0.0003	down
XM_043779737.1	LOC122606857	respiratory burst oxidase homolog protein C-like	0.0005	down
XM_043755186.1	LOC122582756	remorin 4.1-like	< 0.0001	down
XM_043776073.1	LOC122603388	aspartic proteinase 36-like, transcript variant X1	< 0.0001	down
XM_043780780.1	LOC122607749	wound-induced protein 1-like	< 0.0001	down
XM_043759405.1	LOC122587283	putative UPF0481 protein At3g02645	< 0.0001	down
XM_043780826.1	LOC122607783	uncharacterized LOC122607783, transcript variant X2	< 0.0001	down
XM_043781178.1	LOC122608092	protein SENESCENCE-ASSOCIATED GENE 21, mitochondrial-like	< 0.0001	down

Transcript id	GeneID	Gene name/function	*P* Value	result
XM_043776424.1	LOC122603657	probable serine/threonine-protein kinase PIX13, transcript variant X1	0.0003	down
XR_006322328.1	LOC122589763	uncharacterized LOC122589763, transcript variant X3	0.0027	down
XM_043784106.1	LOC122611133	probable phospholipid hydroperoxide glutathione peroxidase	< 0.0001	down
XM_043773257.1	LOC122600529	FCS-Like Zinc finger 1-like	0.0031	down
XM_043771328.1	LOC122598843	NF-X1-type zinc finger protein NFXL1, transcript variant X2	0.0002	down
XM_043763593.1	LOC122591333	delta(8)-fatty-acid desaturase-like	< 0.0001	down
XM_043755313.1	LOC122582881	expansin-A4-like	< 0.0001	down
XM_043753465.1	LOC122581271	uncharacterized LOC122581271, transcript variant X9	< 0.0001	down
XM_043757168.1	LOC122585070	(-)-beta-pinene synthase, chloroplastic-like	< 0.0001	down
XM_043777055.1	LOC122604157	F-box/kelch-repeat protein At3g23880-like	0.0029	down
XM_043753421.1	LOC122581240	ABC transporter C family member 4-like	0.0006	down
XM_043762380.1	LOC122590044	uncharacterized LOC122590044	0.0010	down
XM_043759502.1	LOC122587358	uncharacterized LOC122587358, transcript variant X1	0.0007	down
XM_043774658.1	LOC122601928	glutamyl-tRNA reductase 2-like	< 0.0001	down
XM_043750427.1	LOC122578457	putative transferase At4g12130, mitochondrial	0.0011	down
XR_006323941.1	LOC122599733	uncharacterized LOC122599733	< 0.0001	down
XM_043759153.1	LOC122587078	tropinone reductase homolog At5g06060-like	0.0027	down
XM_043769077.1	LOC122596492	histone-lysine N-methyltransferase ATXR6-like, transcript variant X1	< 0.0001	down
XM_043756767.1	LOC122584718	probable serine/threonine-protein kinase PIX13	0.0024	down
XM_043768355.1	LOC122595883	zinc transporter 5-like	< 0.0001	down
XM_043762628.1	LOC122590304	high-affinity nitrate transporter 3.1-like	0.0043	down
XM_043763443.1	LOC122591217	probable carboxylesterase 12	< 0.0001	down
XM_043763568.1	LOC122591314	RNA polymerase II C-terminal domain phosphatase-like 1	< 0.0001	down
XM_043767900.1	LOC122595529	pectinesterase 1-like	< 0.0001	down
XM_043770668.1	LOC122598056	metacaspase-1-like	< 0.0001	down
XM_043766346.1	LOC122593895	pentatricopeptide repeat-containing protein At1g11710, mitochondrial	0.0008	down
XM_043778907.1	LOC122605941	putative HVA22-like protein g	< 0.0001	down
XM_043764392.1	LOC122592193	glycine-rich cell wall structural protein 1.8-like	< 0.0001	down
XM_043776476.1	LOC122603702	ultraviolet-B receptor UVR8-like, transcript variant X2	< 0.0001	down
XM_043770255.1	LOC122597682	dehydration-responsive element-binding protein 3-like	0.0005	down
XM_043760036.1	LOC122587877	WAT1-related protein At5g64700	< 0.0001	down
XR_006324669.1	LOC122605665	uncharacterized LOC122605665, transcript variant X3	0.0010	down
XM_043763180.1	LOC122591002	protein NRT1/ PTR FAMILY 6.4-like	< 0.0001	down
XM_043765769.1	LOC122593370	enoyl-CoA delta isomerase 2, peroxisomal-like	0.0013	down
XM_043780003.1	LOC122607084	uncharacterized LOC122607084	< 0.0001	down
XM_043782354.1	LOC122609302	probable disease resistance protein At4g27220	< 0.0001	down
XM_043779069.1	LOC122606105	probable endo-1,3(4)-beta-glucanase ARB_01444	< 0.0001	down
XM_043753881.1	LOC122581636	NAD-dependent protein deacylase SRT2-like, transcript variant X1	0.0015	down
XM_043754599.1	LOC122582233	polyubiquitin-like	< 0.0001	down
XM_043761445.1	LOC122589195	uncharacterized LOC122589195	0.0061	down
XR_006320706.1	LOC122579839	uncharacterized LOC122579839	< 0.0001	down
XM_043772311.1	LOC122599751	uncharacterized LOC122599751	< 0.0001	down
XM_043753719.1	LOC122581489	cyclin-dependent kinase F-4-like	0.0023	down
XM_043770759.1	LOC122598158	disease resistance protein RUN1-like	< 0.0001	down
XM_043782750.1	LOC122609809	putative receptor-like protein kinase At5g39000	< 0.0001	down
XM_043765317.1	LOC122592986	uncharacterized LOC122592986	< 0.0001	down
XM_043752911.1	LOC122580644	lysine-specific demethylase JMJ706	< 0.0001	down
XM_043770384.1	LOC122597809	U-box domain-containing protein 26-like	< 0.0001	down
XM_043779891.1	LOC122606978	uncharacterized LOC122606978, transcript variant X1	0.0029	down
XM_043751483.1	LOC122579330	transcription factor TGA3-like	< 0.0001	down
XM_043774629.1	LOC122601896	pathogenesis-related thaumatin-like protein 3.5	0.0055	down
XM_043758645.1	LOC122586665	choline/ethanolaminephosphotransferase 1-like, transcript variant X1	< 0.0001	down
XR_006322586.1	LOC122590718	uncharacterized LOC122590718	< 0.0001	down
XM_043772727.1	LOC122600072	alpha-1,3-arabinosyltransferase XAT2-like	0.0001	down
XM_043782559.1	LOC122609523	receptor-like protein 33	0.0046	down
XM_043775886.1	LOC122603230	SNF1-related protein kinase catalytic subunit alpha KIN10, transcript variant X3	< 0.0001	down
XM_043773442.1	LOC122600688	trihelix transcription factor ASIL1, transcript variant X3	0.0016	down
XM_043779332.1	LOC122606483	probably inactive leucine-rich repeat receptor-like protein kinase At5g48380	0.0028	down
XM_043773197.1	LOC122600480	AT-hook motif nuclear-localized protein 22-like	0.0001	down
XM_043767971.1	LOC122595585	F-box protein At4g00755-like, transcript variant X1	< 0.0001	down
XM_043756383.1	LOC122584022	dirigent protein 18-like	0.0063	down
XM_043781653.1	LOC122608555	uncharacterized LOC122608555	< 0.0001	down

Transcript id	GeneID	Gene name/function	*P* Value	result
XM_043757101.1	LOC122585008	peroxisome biogenesis protein 22, transcript variant X1	0.0024	down
XM_043751761.1	LOC122579572	probable protein phosphatase 2C 12	< 0.0001	down
XM_043770961.1	LOC122598368	probable serine/threonine-protein kinase PBL1	< 0.0001	down
XM_043750947.1	LOC122578898	UDP-glycosyltransferase 72B1-like	< 0.0001	down
XM_043773864.1	LOC122601092	cold-responsive protein kinase 1-like	0.0032	down
XM_043763251.1	LOC122591058	glycerophosphodiester phosphodiesterase GDPDL3-like	< 0.0001	down
XM_043779442.1	LOC122606597	lipoxygenase 1, chloroplastic	< 0.0001	down
XM_043752022.1	LOC122579787	AT-hook motif nuclear-localized protein 23-like	< 0.0001	down
XM_043781215.1	LOC122608122	calcium-transporting ATPase 12, plasma membrane-type-like	< 0.0001	down
XM_043778640.1	LOC122605686	beta-fructofuranosidase, soluble isoenzyme I-like	< 0.0001	down
XM_043752935.1	LOC122580662	pentatricopeptide repeat-containing protein At2g02980, chloroplastic	0.0012	down
XM_043761330.1	LOC122589088	protein SMAX1-LIKE 3-like	0.0004	down
XM_043781650.1	LOC122608551	probable acyl-activating enzyme 17, peroxisomal	0.0015	down
XM_043751000.1	LOC122578941	peroxidase 63-like	< 0.0001	down
XM_043780197.1	LOC122607259	uncharacterized LOC122607259	< 0.0001	down
XM_043765388.1	LOC122593046	AAA-ATPase At2g46620	< 0.0001	down
XM_043767724.1	LOC122595372	uncharacterized LOC122595372	0.0006	down
XM_043756574.1	LOC122584392	ATP synthase subunit 9, mitochondrial-like	0.0022	down
XM_043779185.1	LOC122606223	uncharacterized LOC122606223	< 0.0001	down
XM_043784335.1	LOC122611322	bZIP transcription factor 68-like, transcript variant X1	0.0034	down
XM_043766204.1	LOC122593758	gamma-glutamyl peptidase 3-like	< 0.0001	down
XM_043763198.1	LOC122591013	alpha-glucan phosphorylase, H isozyme	< 0.0001	down
XR_006321021.1	LOC122581618	uncharacterized LOC122581618, transcript variant X2	< 0.0001	down
XR_006322176.1	LOC122588688	uncharacterized LOC122588688	< 0.0001	down
XM_043765249.1	LOC122592917	transmembrane protein 45A	0.0009	down
XM_043750911.1	LOC122578863	GEM-like protein 5	< 0.0001	down
XM_043752211.1	LOC122579944	anthocyanidin 3-O-glucosyltransferase 5-like	< 0.0001	down
XM_043770637.1	LOC122598029	alkaline/neutral invertase A, mitochondrial-like	< 0.0001	down
XM_043781837.1	LOC122608744	short-chain dehydrogenase reductase 2a	< 0.0001	down
XM_043754938.1	LOC122582535	protein SCO1 homolog 2, mitochondrial, transcript variant X1	0.0059	down
XM_043776396.1	LOC122603634	non-specific lipid transfer protein GPI-anchored 6-like	0.0014	down
XM_043775702.1	LOC122603089	xyloglucan endotransglucosylase/hydrolase 2-like	0.0001	down
XM_043773157.1	LOC122600442	3-ketoacyl-CoA synthase 11-like	< 0.0001	down
XM_043759793.1	LOC122587618	F-box protein At2g27310-like	0.0007	down
XM_043763261.1	LOC122591065	COP1-interacting protein 7, transcript variant X1	< 0.0001	down
XM_043781327.1	LOC122608238	protein JINGUBANG-like	0.0037	down
XM_043777084.1	LOC122604187	disease resistance protein Roq1-like	< 0.0001	down
XM_043763275.1	LOC122591079	farnesyl pyrophosphate synthase-like	0.0046	down
XM_043750330.1	LOC122578378	protein RESPONSE TO LOW SULFUR 3-like	0.0002	down
XM_043753094.1	LOC122580945	putative calcium-transporting ATPase 13, plasma membrane-type	< 0.0001	down
XM_043770410.1	LOC122597839	transcription factor MYB1R1	< 0.0001	down
XM_043759417.1	LOC122587291	F-box protein SKIP2-like	0.0003	down
XM_043771965.1	LOC122599451	gamma-glutamylcyclotransferase 2-1-like	0.0005	down
XM_043780012.1	LOC122607092	uncharacterized LOC122607092	0.0063	down
XM_043776450.1	LOC122603675	glutathione S-transferase DHAR2-like	< 0.0001	down
XM_043752555.1	LOC122580278	F-box protein CPR1-like	0.0007	down
XM_043755871.1	LOC122583471	cell number regulator 7-like	0.0008	down
XM_043763230.1	LOC122591041	protein EARLY RESPONSIVE TO DEHYDRATION 15	0.0028	down
XM_043759196.1	LOC122587116	dehydration-responsive element-binding protein 1E-like	< 0.0001	down
XM_043752625.1	LOC122580354	ethylene-responsive transcription factor ERF027-like	< 0.0001	down
XM_043758356.1	LOC122586366	probable CoA ligase CCL9	0.0029	down
XM_043780011.1	LOC122607091	coiled-coil domain-containing protein SCD2, transcript variant X2	< 0.0001	down
XM_043771792.1	LOC122599298	auxin response factor 9, transcript variant X1	< 0.0001	down
XM_043759198.1	LOC122587117	dehydration-responsive element-binding protein 1E-like	0.0004	down
XM_043761305.1	LOC122589062	senescence-associated carboxylesterase 101-like	0.0008	down
XM_043767437.1	LOC122595122	B3 domain-containing protein Os06g0194400-like, transcript variant X2	0.0002	down
XM_043762352.1	LOC122590014	uncharacterized LOC122590014	< 0.0001	down
XR_006320527.1	LOC122578578	uncharacterized LOC122578578, transcript variant X2	< 0.0001	down
XM_043751345.1	LOC122579224	diacylglycerol kinase 5-like, transcript variant X2	< 0.0001	down
XM_043783863.1	LOC122610909	leucine-rich repeat extensin-like protein 4	< 0.0001	down
XM_043769428.1	LOC122596789	WAT1-related protein At2g39510-like	< 0.0001	down
XM_043754982.1	LOC122582570	uncharacterized LOC122582570, transcript variant X2	0.0005	down
XM_043759787.1	LOC122587612	cucumisin-like	< 0.0001	down
XM_043755938.1	LOC122583544	galactolipase DONGLE, chloroplastic-like	0.0060	down

Transcript id	GeneID	Gene name/function	*P* Value	result
XM_043752733.1	LOC122580462	exocyst complex component EXO70B1-like	< 0.0001	down
XM_043768357.1	LOC122595885	spindle and kinetochore-associated protein 1 homolog	0.0018	down
XR_006323456.1	LOC122597765	uncharacterized LOC122597765	0.0040	down
XM_043764258.1	LOC122592046	ethylene-responsive transcription factor ERF017-like	0.0001	down
XM_043761890.1	LOC122589585	U-box domain-containing protein 4-like	< 0.0001	down
XM_043754657.1	LOC122582288	universal stress protein YxiE-like	0.0004	down
XM_043761350.1	LOC122589106	probable boron transporter 2	0.0039	down
XM_043774705.1	LOC122601972	beta-xylosidase/alpha-L-arabinofuranosidase 1-like	0.0011	down
XM_043749984.1	LOC122578105	E3 ubiquitin-protein ligase RGLG2-like, transcript variant X1	0.0058	down
XM_043778071.1	LOC122605176	zinc finger A20 and AN1 domain-containing stress-associated protein 6-like	< 0.0001	down
XM_043778390.1	LOC122605437	early nodulin-20-like	< 0.0001	down
XM_043765705.1	LOC122593312	cold-responsive protein kinase 1	0.0042	down
XM_043764585.1	LOC122592375	probable xyloglucan endotransglucosylase/hydrolase protein 23	< 0.0001	down
XM_043767244.1	LOC122594960	dammarenediol II synthase-like	< 0.0001	down
XM_043783433.1	LOC122610441	ankyrin repeat-containing protein NPR4-like	0.0033	down
XM_043775384.1	LOC122602787	probable cyclic nucleotide-gated ion channel 20, chloroplastic, transcript variant X1	0.0011	down
XM_043756693.1	LOC122584650	3-ketoacyl-CoA synthase 12-like	0.0002	down
XM_043753649.1	LOC122581419	receptor like protein kinase S.2-like	< 0.0001	down
XM_043750870.1	LOC122578824	replication protein A 70 kDa DNA-binding subunit A	< 0.0001	down
XM_043754624.1	LOC122582257	E3 ubiquitin-protein ligase ATL42-like	< 0.0001	down
XM_043761623.1	LOC122589335	uncharacterized LOC122589335	< 0.0001	down
XM_043759287.1	LOC122587187	probable disease resistance protein At5g66900, transcript variant X1	0.0025	down
XM_043763648.1	LOC122591377	scarecrow-like protein 14	0.0001	down
XR_006325035.1	LOC122607408	uncharacterized LOC122607408	< 0.0001	down
XM_043781173.1	LOC122608087	protein EXORDIUM-like 2	< 0.0001	down
XM_043776835.1	LOC122603965	F-box protein At5g49610-like, transcript variant X2	< 0.0001	down
XM_043756816.1	LOC122584760	heavy metal-associated isoprenylated plant protein 26-like	< 0.0001	down
XM_043776247.1	LOC122603522	nematode resistance protein-like HSPRO2	< 0.0001	down
XM_043750442.1	LOC122578470	U-box domain-containing protein 4-like	< 0.0001	down
XM_043765768.1	LOC122593373	histone H2A.Z-specific chaperone CHZ1-like	< 0.0001	down
XM_043774818.1	LOC122602085	protein DETOXIFICATION 51-like	0.0002	down
XM_043772218.1	LOC122599673	AT-hook motif nuclear-localized protein 8-like	< 0.0001	down
XM_043772829.1	LOC122600160	uncharacterized LOC122600160	< 0.0001	down
XM_043773579.1	LOC122600812	uncharacterized LOC122600812	0.0003	down
XM_043760243.1	LOC122588103	serine/threonine-protein kinase STY46-like	< 0.0001	down
XM_043779893.1	LOC122606979	N-terminal acetyltransferase B complex auxiliary subunit NAA25, transcript variant X1	< 0.0001	down
XM_043754548.1	LOC122582188	probable pectinesterase/pectinesterase inhibitor 41	< 0.0001	down
XM_043765655.1	LOC122593267	dof zinc finger protein DOF2.4-like	< 0.0001	down
XM_043765910.1	LOC122593492	putative UPF0481 protein At3g02645	< 0.0001	down
XM_043770704.1	LOC122598098	cyclin-SDS	0.0009	down
XM_043775366.1	LOC122602772	scarecrow-like protein 15	< 0.0001	down
XM_043784167.1	LOC122611187	amino acid permease 3-like	< 0.0001	down
XM_043768479.1	LOC122595987	50S ribosomal protein L22, chloroplastic-like, transcript variant X2	0.0006	down
XM_043754414.1	LOC122582065	polyamine oxidase 1	< 0.0001	down
XM_043753455.1	LOC122581265	phototropin-1	0.0025	down
XM_043752015.1	LOC122579780	protein PHOSPHATE-INDUCED 1-like	0.0008	down
XM_043778135.1	LOC122605227	NDR1/HIN1-like protein 3	< 0.0001	down
XM_043778699.1	LOC122605743	NDR1/HIN1-like protein 13	< 0.0001	down
XM_043761934.1	LOC122589625	uncharacterized LOC122589625, transcript variant X1	< 0.0001	down
XM_043758571.1	LOC122586587	NDR1/HIN1-like protein 1	< 0.0001	down
XM_043781204.1	LOC122608115	transcription factor TGA4-like, transcript variant X1	0.0004	down
XM_043762570.1	LOC122590240	probable 2-oxoglutarate-dependent dioxygenase AOP1	0.0001	down
XM_043772983.1	LOC122600280	BOI-related E3 ubiquitin-protein ligase 1-like	< 0.0001	down
XM_043775236.1	LOC122602635	1-aminocyclopropane-1-carboxylate synthase-like	< 0.0001	down
XM_043751877.1	LOC122579662	F-box/kelch-repeat protein At3g06240-like	< 0.0001	down
XM_043774258.1	LOC122601502	S-type anion channel SLAH1-like	< 0.0001	down
XM_043781568.1	LOC122608461	protein PHLOEM PROTEIN 2-LIKE A1-like	0.0020	down
XM_043765690.1	LOC122593298	WRKY transcription factor 22-like	< 0.0001	down
XM_043764893.1	LOC122592618	uncharacterized LOC122592618	< 0.0001	down
XM_043782041.1	LOC122608973	double-stranded RNA-binding protein 2	< 0.0001	down
XM_043765385.1	LOC122593043	pathogenesis-related genes transcriptional activator PTI6-like	0.0003	down
XM_043757978.1	LOC122585863	phospholipase A1-Igamma3, chloroplastic	0.0007	down

Transcript id	GeneID	Gene name/function	P Value	result
XM_043779695.1	LOC122606825	probable serine/threonine-protein kinase DDB_G0271402, transcript variant X6	< 0.0001	down
XM_043752016.1	LOC122579781	probable WRKY transcription factor 27	< 0.0001	down
XM_043784023.1	LOC122611066	imidazole glycerol phosphate synthase hisHF, chloroplastic, transcript variant X1	0.0056	down
XM_043784033.1	LOC122611074	transcription initiation factor TFIID subunit 2, transcript variant X3	0.0044	down
XM_043753931.1	LOC122581680	phosphoinositide phospholipase C 2-like	< 0.0001	down
XM_043759426.1	LOC122587300	exocyst complex component SEC3A-like	0.0051	down
XM_043771354.1	LOC122598859	serine/threonine-protein kinase prpf4B-like, transcript variant X8	< 0.0001	down
XR_006323317.1	LOC122596391	uncharacterized LOC122596391, transcript variant X2	0.0019	down
XM_043770894.1	LOC122598303	ATPase WRNIP1	0.0011	down
XM_043781469.1	LOC122608374	tRNA-uridine aminocarboxypropyltransferase 2, transcript variant X2	< 0.0001	down
XM_043767813.1	LOC122595451	probable clathrin assembly protein At4g32285	< 0.0001	down
XM_043759164.1	LOC122587089	zinc finger CCCH domain-containing protein 29-like	0.0033	down
XR_006322870.1	LOC122593804	F-box protein AUF1-like, transcript variant X1	0.0059	down
XM_043778346.1	LOC122605399	aspartic proteinase nepenthesin-1	0.0012	down
XM_043769283.1	LOC122596664	B-box zinc finger protein 22-like	< 0.0001	down
XM_043755227.1	LOC122582789	GTP cyclohydrolase 1	< 0.0001	down
XM_043758350.1	LOC122586355	uncharacterized LOC122586355	0.0002	down
XM_043762433.1	LOC122590102	pleiotropic drug resistance protein 1-like, transcript variant X2	0.0062	down
XM_043772166.1	LOC122599628	homeobox-leucine zipper protein HAT4	< 0.0001	down
XM_043756752.1	LOC122584704	MACPF domain-containing protein At1g14780	< 0.0001	down
XM_043765255.1	LOC122592922	SPX domain-containing membrane protein At4g22990-like	0.0026	down
XM_043762872.1	LOC122590691	uncharacterized LOC122590691	0.0001	down
XM_043753608.1	LOC122581384	CBL-interacting serine/threonine-protein kinase 11	< 0.0001	down
XM_043757870.1	LOC122585741	uncharacterized LOC122585741	0.0038	down
XM_043772722.1	LOC122600066	MYB-like transcription factor ODO1	0.0022	down
XM_043782271.1	LOC122609211	protein MEI2-like 2, transcript variant X3	< 0.0001	down
XM_043750513.1	LOC122578533	S-adenosylmethionine synthase 5	< 0.0001	down
XM_043761567.1	LOC122589291	1-aminocyclopropane-1-carboxylate synthase 9-like	0.0021	down
XM_043775554.1	LOC122602941	protein ENHANCED PSEUDOMONAS SUSCEPTIBILITY 1	< 0.0001	down
XM_043777729.1	LOC122604858	phenylalanine ammonia-lyase, transcript variant X2	< 0.0001	down
XM_043756251.1	LOC122583878	protein FAR1-RELATED SEQUENCE 5-like, transcript variant X2	0.0062	down
XM_043760634.1	LOC122588502	probable trehalose-phosphate phosphatase J	< 0.0001	down
XM_043775210.1	LOC122602610	putative F-box protein At1g47790	0.0030	down
XM_043754207.1	LOC122581892	PRA1 family protein B3-like	< 0.0001	down
XM_043774710.1	LOC122601978	ethylene-responsive transcription factor ERF109-like	< 0.0001	down
XM_043750052.1	LOC122578154	probable serine/threonine-protein kinase PIX13, transcript variant X1	< 0.0001	down
XM_043757358.1	LOC122585239	molybdenum cofactor sulfurase	0.0016	down
XM_043756616.1	LOC122584454	zinc finger protein CONSTANS-like	< 0.0001	down
XM_043782794.1	LOC122609847	butanoate--CoA ligase AAE1-like	< 0.0001	down
XM_043757934.1	LOC122585811	cyclic dof factor 3-like	0.0002	down
XM_043751067.1	LOC122578993	BOI-related E3 ubiquitin-protein ligase 1-like, transcript variant X2	0.0005	down
XM_043775792.1	LOC122603163	choline-phosphate cytidylyltransferase 2-like	< 0.0001	down
XM_043769220.1	LOC122596609	CASP-like protein 2C1	< 0.0001	down
XM_043762272.1	LOC122589940	F-box protein At5g07610-like	0.0017	down
XM_043779572.1	LOC122606714	probable serine/threonine-protein kinase PIX13, transcript variant X1	0.0063	down
XM_043763360.1	LOC122591144	auxin response factor 3, transcript variant X1	< 0.0001	down
XM_043752390.1	LOC122580118	ABC transporter G family member 14-like	0.0003	down
XM_043772563.1	LOC122599943	sodium transporter HKT1-like	0.0039	down
XM_043784052.1	LOC122611091	thiamine thiazole synthase, chloroplastic-like	< 0.0001	down
XM_043756361.1	LOC122583990	aluminum-activated malate transporter 13-like	< 0.0001	down
XM_043778993.1	LOC122606023	uncharacterized LOC122606023	0.0012	down
XR_006322656.1	LOC122590996	DEAD-box ATP-dependent RNA helicase 42, transcript variant X1	< 0.0001	down
XM_043754038.1	LOC122581763	malonyl-coenzyme A:anthocyanin 3-O-glucoside-6"-O-malonyltransferase-like	0.0003	down
XM_043758928.1	LOC122586905	uncharacterized LOC122586905	< 0.0001	down
XM_043774866.1	LOC122602139	probable glutathione S-transferase	0.0001	down
XM_043759399.1	LOC122587275	protein PHOSPHATE-INDUCED 1-like	0.0018	down
XM_043770245.1	LOC122597672	serine/arginine repetitive matrix protein 1-like	0.0040	down
XM_043758683.1	LOC122586699	uncharacterized LOC122586699	< 0.0001	down
XM_043758433.1	LOC122586444	uncharacterized LOC122586444, transcript variant X1	< 0.0001	down
XM_043772775.1	LOC122600112	protein RALF-like 33	< 0.0001	down
XM_043767260.1	LOC122594977	probable linoleate 9S-lipoxygenase 5	< 0.0001	down
XM_043773453.1	LOC122600699	AAA-ATPase At3g28580-like	< 0.0001	down

Transcript id	GeneID	Gene name/function	*P* Value	result
XM_043778518.1	LOC122605561	peamaclein-like	< 0.0001	down
XM_043758718.1	LOC122586729	ubiquitin-conjugating enzyme E2 28-like	< 0.0001	down
XM_043763539.1	LOC122591297	protein TIFY 10A-like	< 0.0001	down
XM_043755163.1	LOC122582740	lachrymatory-factor synthase	< 0.0001	down
XM_043764599.1	LOC122592386	scarecrow-like transcription factor PAT1	0.0015	down
XM_043765779.1	LOC122593382	shikimate O-hydroxycinnamoyltransferase-like	< 0.0001	down
XM_043760042.1	LOC122587882	ethylene-responsive transcription factor ERF014-like	0.0002	down
XM_043754600.1	LOC122582231	bidirectional sugar transporter SWEET10-like	0.0042	down
XM_043769302.1	LOC122596684	kirola-like	< 0.0001	down
XM_043760701.1	LOC122588564	protein EXORDIUM-like 2	0.0008	down
XM_043783372.1	LOC122610377	probable polygalacturonase	< 0.0001	down
XM_043775863.1	LOC122603216	fasciclin-like arabinogalactan protein 16	< 0.0001	down
XM_043768208.1	LOC122595771	aspartic proteinase-like	< 0.0001	down
XM_043761264.1	LOC122589029	BAG family molecular chaperone regulator 2-like	< 0.0001	down
XM_043766208.1	LOC122593763	calcium-binding protein PBP1-like	0.0042	down
XM_043770209.1	LOC122597636	uncharacterized LOC122597636	0.0014	down
XM_043761173.1	LOC122588953	germin-like protein subfamily 3 member 4	< 0.0001	down
XM_043776823.1	LOC122603955	transcription factor TCP19	< 0.0001	down
XM_043760027.1	LOC122587865	mitochondrial uncoupling protein 5-like	< 0.0001	down
XM_043766810.1	LOC122594336	glutaredoxin-C9-like	< 0.0001	down
XM_043770858.1	LOC122598256	S-type anion channel SLAH1-like	0.0012	down
XM_043769884.1	LOC122597270	inositol-tetrakisphosphate 1-kinase 1-like	0.0001	down
XM_043769931.1	LOC122597325	F-box/FBD/LRR-repeat protein At1g13570-like	0.0002	down
XM_043762397.1	LOC122590063	probable receptor-like protein kinase At2g39360	< 0.0001	down
XM_043757043.1	LOC122584951	AT-hook motif nuclear-localized protein 23-like	0.0010	down
XM_043768924.1	LOC122596360	glycosyltransferase BC10-like	< 0.0001	down
XM_043754864.1	LOC122582470	CBL-interacting protein kinase 2-like	< 0.0001	down
XM_043751296.1	LOC122579182	protein PHOX1-like	0.0001	down
XM_043763635.1	LOC122591367	probable xyloglucan endotransglucosylase/hydrolase protein 23	0.0055	down
XM_043767650.1	LOC122595305	L-type lectin-domain containing receptor kinase VIII.2-like	0.0034	down
XM_043776258.1	LOC122603532	protein NRT1/ PTR FAMILY 5.8	0.0002	down
XM_043753453.1	LOC122581264	protein transport protein Sec24-like At3g07100, transcript variant X3	0.0012	down
XM_043752328.1	LOC122580057	uncharacterized LOC122580057	0.0002	down
XM_043750975.1	LOC122578924	protein NEN1-like	< 0.0001	down
XM_043779451.1	LOC122606607	probable methyltransferase PMT24	< 0.0001	down
XM_043775219.1	LOC122602624	17.8 kDa class I heat shock protein-like	< 0.0001	down
XM_043773510.1	LOC122600753	uncharacterized LOC122600753	< 0.0001	down
XM_043766912.1	LOC122594461	protein GLUTAMINE DUMPER 3-like	0.0052	down
XR_006322223.1	LOC122589066	uncharacterized LOC122589066	< 0.0001	down
XM_043775073.1	LOC122602383	pelargonidin 3-O-(6-caffeoylglucoside) 5-O-(6-O-malonylglucoside) 4'''-malonyltransferase-like	0.0040	down
XM_043766682.1	LOC122594211	uncharacterized LOC122594211	0.0039	down
XM_043766874.1	LOC122594413	uncharacterized LOC122594413	0.0015	down
XM_043760168.1	LOC122588021	ferric reduction oxidase 2-like	< 0.0001	down
XM_043771382.1	LOC122598902	probable serine/threonine-protein kinase PBL28	0.0055	down
XM_043783462.1	LOC122610474	putative leucine-rich repeat receptor-like protein kinase At2g19210	0.0061	down
XM_043782508.1	LOC122609462	protein NIM1-INTERACTING 1-like	0.0002	down
XM_043761935.1	LOC122589627	interferon-related developmental regulator 1-like	< 0.0001	down
XM_043761346.1	LOC122589102	clavaminate synthase-like protein At3g21360	< 0.0001	down
XM_043763315.1	LOC122591109	putative esterase YitV, transcript variant X4	< 0.0001	down
XM_043767610.1	LOC122595271	transcription factor bHLH35-like	< 0.0001	down
XM_043782951.1	LOC122609986	2-alkenal reductase (NADP(+)-dependent)-like	0.0048	down
XM_043781566.1	LOC122608459	cytochrome P450 Tp4149-like	0.0059	down
XM_043781941.1	LOC122608864	probable protein phosphatase 2C 49	< 0.0001	down
XM_043781265.1	LOC122608175	NAC domain-containing protein 83-like	< 0.0001	down
XM_043762144.1	LOC122589823	uncharacterized LOC122589823	< 0.0001	down
XM_043750328.1	LOC122578380	transcription factor bHLH162-like	< 0.0001	down
XM_043779653.1	LOC122606797	cytosolic sulfotransferase 15-like	0.0008	down
XM_043755378.1	LOC122582947	uncharacterized protein C24B11.05	< 0.0001	down
XM_043778437.1	LOC122605482	NDR1/HIN1-like protein 6	0.0002	down
XM_043773286.1	LOC122600552	inositol transporter 4-like, transcript variant X2	0.0019	down
XM_043757072.1	LOC122584978	uncharacterized LOC122584978	< 0.0001	down
XM_043775221.1	LOC122602626	putative wall-associated receptor kinase-like 16	0.0054	down
XM_043775171.1	LOC122602569	putative lipase ROG1, transcript variant X3	< 0.0001	down
XM_043765150.1	LOC122592832	60S ribosomal protein L3-like	0.0021	down

Transcript id	GeneID	Gene name/function	*P* Value	result
XM_043780285.1	LOC122607330	bidirectional sugar transporter SWEET9-like	0.0005	down
XM_043782057.1	LOC122608992	dehydrin ERD14-like	< 0.0001	down
XR_006323273.1	LOC122595961	uncharacterized LOC122595961	< 0.0001	down
XM_043760680.1	LOC122588547	serine/threonine-protein kinase STY13-like	0.0009	down
XM_043752107.1	LOC122579855	1-aminocyclopropane-1-carboxylate synthase-like	0.0006	down
XM_043772633.1	LOC122600003	patatin-like protein 2	< 0.0001	down
XM_043754663.1	LOC122582292	LRR receptor-like serine/threonine-protein kinase ERL1	0.0032	down
XR_006323854.1	LOC122599059	uncharacterized LOC122599059	0.0003	down
XM_043753441.1	LOC122581256	protein SULFUR DEFICIENCY-INDUCED 2, transcript variant X1	< 0.0001	down
XM_043766579.1	LOC122594107	strigolactone esterase RMS3	< 0.0001	down
XM_043752559.1	LOC122580282	senescence/dehydration-associated protein At3g51250-like	< 0.0001	down
XM_043752402.1	LOC122580132	cytochrome P450 81Q32-like	< 0.0001	down
XM_043755297.1	LOC122582863	uncharacterized LOC122582863	< 0.0001	down
XM_043771769.1	LOC122599281	ubiquitin-conjugating enzyme E2 28-like, transcript variant X2	< 0.0001	down
XM_043766757.1	LOC122594285	ABC transporter B family member 6-like	0.0007	down
XM_043759142.1	LOC122587069	dehydration-responsive element-binding protein 1E-like	< 0.0001	down
XM_043762274.1	LOC122589942	heat stress transcription factor B-4-like	0.0002	down
XM_043776768.1	LOC122603915	uncharacterized LOC122603915	0.0010	down
XR_006324754.1	LOC122605918	uncharacterized LOC122605918, transcript variant X2	0.0037	down
XM_043755779.1	LOC122583365	ankyrin repeat-containing protein NPR4-like	0.0002	down
XM_043776754.1	LOC122603905	uncharacterized LOC122603905, transcript variant X4	< 0.0001	down
XM_043761950.1	LOC122589636	protein C2-DOMAIN ABA-RELATED 4-like	0.0062	down
XM_043783006.1	LOC122610033	auxin response factor 8-like, transcript variant X2	0.0049	down
XM_043753117.1	LOC122580972	probably inactive leucine-rich repeat receptor-like protein kinase At3g28040	< 0.0001	down
XM_043761315.1	LOC122589070	histidine-containing phosphotransfer protein 4, transcript variant X3	0.0027	down
XM_043760782.1	LOC122588627	phosphatidylcholine:diacylglycerol cholinephosphotransferase 1-like	< 0.0001	down
XM_043766954.1	LOC122594507	glycine-rich protein A3-like	< 0.0001	down
XR_006325236.1	LOC122608513	uncharacterized LOC122608513	< 0.0001	down
XM_043775091.1	LOC122602402	13S globulin seed storage protein 2-like	0.0007	down
XM_043750651.1	LOC122578646	probable carboxylesterase 18	0.0059	down
XM_043751896.1	LOC122579677	delta(7)-sterol-C5(6)-desaturase-like	< 0.0001	down
XM_043773147.1	LOC122600437	4-coumarate--CoA ligase-like 6	< 0.0001	down
XM_043753569.1	LOC122581351	protein DETOXIFICATION 42-like, transcript variant X1	< 0.0001	down
XM_043769730.1	LOC122597094	probable inactive poly [ADP-ribose] polymerase SRO2	< 0.0001	down
XM_043754738.1	LOC122582360	germacrene A hydroxylase-like	< 0.0001	down
XM_043762454.1	LOC122590118	ADP-ribosylation factor-like, transcript variant X2	< 0.0001	down
XM_043763534.1	LOC122591292	thiamine thiazole synthase, chloroplastic	0.0010	down
XM_043754643.1	LOC122582271	protein DETOXIFICATION 43-like, transcript variant X1	0.0007	down
XM_043761651.1	LOC122589364	hippocampus abundant transcript-like protein 1	0.0061	down
XM_043752701.1	LOC122580430	protein NLP7-like, transcript variant X3	0.0001	down
XR_006321197.1	LOC122582544	uncharacterized LOC122582544	< 0.0001	down
XM_043756209.1	LOC122583829	E3 ubiquitin-protein ligase PUB22-like	< 0.0001	down
XM_043776290.1	LOC122603560	probable glycosyltransferase At5g03795, transcript variant X1	0.0002	down
XM_043781570.1	LOC122608462	cytochrome P450 94B3-like	< 0.0001	down
XM_043773482.1	LOC122600728	protein SRC2 homolog	< 0.0001	down
XM_043754048.1	LOC122581772	B-box zinc finger protein 24-like	< 0.0001	down
XM_043766967.1	LOC122594521	vacuolar iron transporter homolog 4-like	0.0002	down
XM_043780833.1	LOC122607785	vesicle-associated membrane protein 721-like	< 0.0001	down
XM_043758550.1	LOC122586567	loricrin-like	0.0023	down
XM_043783493.1	LOC122610508	putative disease resistance RPP13-like protein 1	0.0032	down
XM_043781352.1	LOC122608257	uncharacterized LOC122608257, transcript variant X2	< 0.0001	down
XR_006325502.1	LOC122611282	uncharacterized LOC122611282	< 0.0001	down
XM_043783916.1	LOC122610965	protein CUP-SHAPED COTYLEDON 2	< 0.0001	down
XM_043777974.1	LOC122605098	protein ROOT HAIR DEFECTIVE 3-like	0.0003	down
XM_043776072.1	LOC122603387	probable E3 ubiquitin-protein ligase RHC2A	0.0001	down
XM_043783811.1	LOC122610852	ethylene-responsive transcription factor CRF2-like	< 0.0001	down
XM_043759404.1	LOC122587282	glucan endo-1,3-beta-glucosidase 7	< 0.0001	down
XM_043768903.1	LOC122596340	vacuolar iron transporter 1	< 0.0001	down
XM_043762292.1	LOC122589964	calcium-binding protein CML38-like	0.0011	down
XM_043754674.1	LOC122582303	casparian strip membrane protein 1-like	0.0055	down
XR_006324375.1	LOC122602715	uncharacterized LOC122602715, transcript variant X2	0.0007	down
XM_043772158.1	LOC122599622	pectin acetylesterase 8-like	< 0.0001	down
XM_043757913.1	LOC122585789	E3 ubiquitin-protein ligase PUB23-like	< 0.0001	down
XM_043765007.1	LOC122592717	zinc finger protein CONSTANS-LIKE 5-like	< 0.0001	down

Transcript id	GeneID	Gene name/function	*P* Value	result
XM_043763582.1	LOC122591321	protein LONG AFTER FAR-RED 3, transcript variant X1	0.0037	down
XM_043765458.1	LOC122593103	PI-PLC X domain-containing protein At5g67130-like	0.0004	down
XM_043783472.1	LOC122610486	putative pectinesterase/pectinesterase inhibitor 26	0.0007	down
XR_006320795.1	LOC122580451	uncharacterized LOC122580451	< 0.0001	down
XM_043752381.1	LOC122580107	receptor-like protein kinase ANXUR1	< 0.0001	down
XM_043783306.1	LOC122610310	GATA transcription factor 8-like	< 0.0001	down
XM_043751247.1	LOC122579137	zinc finger CCCH domain-containing protein 38-like, transcript variant X1	0.0040	down
XM_043765665.1	LOC122593276	cytochrome P450 710A11-like	< 0.0001	down
XR_006322116.1	LOC122587146	uncharacterized LOC122587146, transcript variant X2	0.0005	down
XM_043775722.1	LOC122603105	U-box domain-containing protein 4-like, transcript variant X1	0.0010	down
XM_043770036.1	LOC122597443	peroxidase N1-like	0.0059	down
XM_043783874.1	LOC122610920	zinc finger protein ZAT11-like	0.0007	down
XM_043774205.1	LOC122601445	ACD11 homolog protein-like	0.0009	down
XM_043766531.1	LOC122594050	NDR1/HIN1-like protein 2	< 0.0001	down
XM_043767652.1	LOC122595310	shikimate O-hydroxycinnamoyltransferase-like	0.0008	down
XM_043750985.1	LOC122578934	protein SPIRAL1-like 2	< 0.0001	down
XM_043765081.1	LOC122592772	cytochrome b561 and DOMON domain-containing protein At5g35735-like	< 0.0001	down
XM_043752908.1	LOC122580640	uncharacterized LOC122580640	0.0013	down
XM_043766439.1	LOC122593963	nudix hydrolase 10-like	< 0.0001	down
XM_043762475.1	LOC122590138	transcription factor ILI6-like	0.0013	down
XM_043778173.1	LOC122605265	arogenate dehydratase 3-like	< 0.0001	down
XM_043750722.1	LOC122578704	transcription factor TCP15-like	< 0.0001	down
XM_043763775.1	LOC122591506	wall-associated receptor kinase 2-like	< 0.0001	down
XM_043757544.1	LOC122585410	ABC transporter G family member 6-like	< 0.0001	down
XM_043760154.1	LOC122588006	putative receptor-like protein kinase At5g39000	0.0027	down
XM_043755716.1	LOC122583290	probable WRKY transcription factor 57, transcript variant X2	< 0.0001	down
XM_043780361.1	LOC122607395	zinc finger protein ZAT10	0.0006	down
XM_043772307.1	LOC122599746	zerumbone synthase-like	< 0.0001	down
XM_043754639.1	LOC122582268	unknown protein 1	< 0.0001	down
XM_043762847.1	LOC122590674	E3 ubiquitin-protein ligase PRT6, transcript variant X4	< 0.0001	down
XM_043759461.1	LOC122587327	cyclin-dependent kinase F-4-like	< 0.0001	down
XM_043758168.1	LOC122586057	tetrahydrocannabinolic acid synthase-like	< 0.0001	down
XM_043780002.1	LOC122607083	uncharacterized LOC122607083	< 0.0001	down
XM_043762165.1	LOC122589845	probable calcium-binding protein CML41	0.0002	down
XM_043753243.1	LOC122581094	3-dehydrosphinganine reductase TSC10A-like	< 0.0001	down
XM_043750490.1	LOC122578515	transcription factor MYB10-like	< 0.0001	down
XM_043771085.1	LOC122598491	uncharacterized LOC122598491	0.0005	down
XM_043772078.1	LOC122599555	GTP-binding nuclear protein Ran1A-like	0.0013	down
XM_043761275.1	LOC122589038	ubiquitin-conjugating enzyme E2 7-like	0.0048	down
XM_043762928.1	LOC122590754	probable protein phosphatase 2C 34, transcript variant X1	< 0.0001	down
XM_043760129.1	LOC122587981	probable transcriptional regulator RABBIT EARS	0.0018	down
XM_043775117.1	LOC122602437	arginine decarboxylase-like	0.0002	down
XM_043778046.1	LOC122605156	ethylene-responsive transcription factor ERF023-like	< 0.0001	down
XM_043781538.1	LOC122608429	purple acid phosphatase 3-like	0.0052	down
XM_043765306.1	LOC122592973	uncharacterized protein C24B11.05-like	< 0.0001	down
XM_043770459.1	LOC122597881	WRKY transcription factor WRKY51-like	< 0.0001	down
XM_043762199.1	LOC122589872	allene oxide cyclase, chloroplastic-like	< 0.0001	down
XM_043774831.1	LOC122602101	uncharacterized LOC122602101	0.0007	down
XM_043779873.1	LOC122606967	probable beta-D-xylosidase 7	0.0009	down
XM_043757070.1	LOC122584977	soluble inorganic pyrophosphatase 1	0.0050	down
XM_043778744.1	LOC122605791	tetraspanin-6-like	< 0.0001	down
XM_043757842.1	LOC122585719	chaperone protein dnaJ 20, chloroplastic-like	0.0008	down
XM_043756891.1	LOC122584827	transcription factor ILR3-like	< 0.0001	down
XM_043756863.1	LOC122584799	probable E3 ubiquitin-protein ligase RHA4A	< 0.0001	down
XM_043782991.1	LOC122610019	protein TOPLESS-like	< 0.0001	down
XM_043770725.1	LOC122598124	AT-hook motif nuclear-localized protein 22-like	< 0.0001	down
XM_043766226.1	LOC122593777	protein TIFY 5A	< 0.0001	down
XM_043764902.1	LOC122592622	adenylate isopentenyltransferase 3, chloroplastic-like	0.0011	down
XR_006325194.1	LOC122608261	uncharacterized LOC122608261	0.0002	down
XM_043759659.1	LOC122587474	NDR1/HIN1-like protein 13	0.0012	down
XM_043782605.1	LOC122609575	protein SABRE, transcript variant X2	< 0.0001	down
XM_043769909.1	LOC122597297	cytochrome P450 CYP749A22-like	0.0002	down
XM_043756267.1	LOC122583894	egg cell-secreted protein 1.1-like	0.0001	down

Transcript id	GeneID	Gene name/function	*P* Value	result
XM_043750644.1	LOC122578640	transmembrane protein 53	< 0.0001	down
XM_043778711.1	LOC122605756	mitogen-activated protein kinase kinase kinase 20-like	< 0.0001	down
XM_043752745.1	LOC122580474	uncharacterized LOC122580474	< 0.0001	down
XM_043762990.1	LOC122590821	LRR receptor-like serine/threonine-protein kinase EFR	< 0.0001	down
XM_043763250.1	LOC122591057	zinc finger CCCH domain-containing protein 20	0.0014	down
XM_043766608.1	LOC122594135	uncharacterized protein C24B11.05-like	0.0016	down
XM_043763505.1	LOC122591267	phospholipase A1 PLIP2, chloroplastic-like	< 0.0001	down
XM_043776964.1	LOC122604063	NDR1/HIN1-like protein 3	0.0046	down
XM_043776912.1	LOC122604026	protein SODIUM POTASSIUM ROOT DEFECTIVE 2	0.0023	down
XM_043765100.1	LOC122592788	lateral signaling target protein 2 homolog	0.0005	down
XM_043768695.1	LOC122596164	homocysteine S-methyltransferase 1	0.0022	down
XM_043779190.1	LOC122606227	agamous-like MADS-box protein AGL62	< 0.0001	down
XM_043761157.1	LOC122588937	ABC transporter G family member 25	0.0019	down
XM_043771469.1	LOC122599022	ethylene-responsive transcription factor ERF017-like	< 0.0001	down
XM_043763398.1	LOC122591179	non-specific lipid transfer protein GPI-anchored 5-like	0.0057	down
XM_043780967.1	LOC122607895	allene oxide synthase	0.0004	down
XM_043757564.1	LOC122585431	WRKY transcription factor 22-like	< 0.0001	down
XM_043759453.1	LOC122587317	EP1-like glycoprotein 3	< 0.0001	down
XM_043775759.1	LOC122603138	cucumber peeling cupredoxin	0.0012	down
XM_043784061.1	LOC122611096	probable trehalose-phosphate phosphatase D	< 0.0001	down
XM_043775912.1	LOC122603252	probable serine/threonine-protein kinase PIX13	0.0005	down
XM_043780329.1	LOC122607368	dimethylnonatriene synthase-like	0.0004	down
XM_043782895.1	LOC122609941	cellulose synthase-like protein D2	< 0.0001	down
XM_043783752.1	LOC122610796	uncharacterized LOC122610796	0.0055	down
XM_043782649.1	LOC122609701	uncharacterized LOC122609701	< 0.0001	down
XM_043758427.1	LOC122586437	putative pentatricopeptide repeat-containing protein At5g52630, transcript variant X2	0.0003	down
XM_043781017.1	LOC122607939	protein LIGHT-DEPENDENT SHORT HYPOCOTYLS 5-like	0.0016	down
XM_043754001.1	LOC122581733	myb-related protein 2, transcript variant X2	< 0.0001	down
XM_043761806.1	LOC122589507	calcium-dependent mitochondrial ATP-magnesium/phosphate carrier protein 2-like, transcript variant X2	< 0.0001	down
XM_043770315.1	LOC122597746	zinc finger A20 and AN1 domain-containing stress-associated protein 5-like	< 0.0001	down
XM_043760161.1	LOC122588014	uncharacterized LOC122588014	0.0010	down
XM_043750003.1	LOC122578104	squamosa promoter-binding-like protein 7, transcript variant X1	0.0002	down
XM_043780692.1	LOC122607673	protein OCTOPUS	< 0.0001	down
XR_006325455.1	LOC122610309	uncharacterized LOC122610309	0.0016	down
XM_043771790.1	LOC122599297	phosphatidylinositol 3,4,5-trisphosphate 3-phosphatase and protein-tyrosine-phosphatase PTEN2A-like, transcript variant X3	< 0.0001	down
XM_043781491.1	LOC122608392	uncharacterized LOC122608392	0.0003	down
XM_043767551.1	LOC122595214	protein unc-13 homolog	< 0.0001	down
XR_006323437.1	LOC122597473	ABC transporter F family member 4-like, transcript variant X7	< 0.0001	down
XM_043781758.1	LOC122608666	wound-induced protein 1-like	0.0011	down
XM_043769233.1	LOC122596621	probable 2-oxoglutarate-dependent dioxygenase AOP1	< 0.0001	down
XM_043771317.1	LOC122598832	putative ABC transporter B family member 8	< 0.0001	down
XM_043779240.1	LOC122606276	acetylserotonin O-methyltransferase-like	< 0.0001	down
XM_043768649.1	LOC122596128	probable serine incorporator	< 0.0001	down
XM_043766062.1	LOC122593635	subtilisin-like protease SBT5.6	< 0.0001	down
XM_043762484.1	LOC122590147	uncharacterized LOC122590147	0.0008	down
XM_043761285.1	LOC122589046	uncharacterized LOC122589046	0.0002	down
XM_043761304.1	LOC122589061	lysine-rich arabinogalactan protein 17-like	< 0.0001	down
XM_043778203.1	LOC122605294	ethylene-responsive transcription factor CRF4-like	< 0.0001	down
XM_043756195.1	LOC122583817	non-specific lipid-transfer protein-like, transcript variant X2	< 0.0001	down
XM_043765536.1	LOC122593165	glutathione S-transferase APIC-like	< 0.0001	down
XM_043766905.1	LOC122594455	ethylene-responsive transcription factor 12-like	< 0.0001	down
XM_043755468.1	LOC122583032	cation/H(+) antiporter 20-like, transcript variant X1	< 0.0001	down
XM_043770491.1	LOC122597904	uncharacterized LOC122597904	< 0.0001	down
XM_043769074.1	LOC122596491	uncharacterized LOC122596491, transcript variant X1	0.0004	down
XM_043778279.1	LOC122605354	laccase-12-like	< 0.0001	down
XM_043764624.1	LOC122592406	RING-H2 finger protein ATL74-like	< 0.0001	down
XM_043754680.1	LOC122582311	glutaredoxin-C13-like	< 0.0001	down
XM_043779201.1	LOC122606240	uncharacterized LOC122606240	< 0.0001	down
XM_043767493.1	LOC122595167	thioredoxin H2-like, transcript variant X1	< 0.0001	down
XM_043761429.1	LOC122589178	cytochrome P450 736A117-like	< 0.0001	down
XM_043772457.1	LOC122599862	probable ribose-5-phosphate isomerase 3, chloroplastic	< 0.0001	down

Transcript id	GeneID	Gene name/function	*P* Value	result
XM_043754656.1	LOC122582287	uncharacterized LOC122582287	< 0.0001	down
XM_043761351.1	LOC122589108	uncharacterized LOC122589108	0.0015	down
XM_043765118.1	LOC122592803	U-box domain-containing protein 26-like	< 0.0001	down
XM_043768752.1	LOC122596212	common plant regulatory factor 1-like, transcript variant X2	0.0013	down
XM_043782326.1	LOC122609269	probable protein phosphatase 2C 2	0.0001	down
XM_043753138.1	LOC122580994	ABC transporter B family member 21-like, transcript variant X2	< 0.0001	down
XM_043765116.1	LOC122592802	nuclear cap-binding protein subunit 1, transcript variant X1	< 0.0001	down
XM_043756285.1	LOC122583911	polycomb group protein EMBRYONIC FLOWER 2, transcript variant X2	0.0010	down
XM_043774058.1	LOC122601293	early nodulin-like protein 1	0.0041	down
XM_043757103.1	LOC122585009	uncharacterized LOC122585009	0.0002	down
XM_043755170.1	LOC122582742	23 kDa jasmonate-induced protein-like	< 0.0001	down
XM_043780313.1	LOC122607356	serine/threonine-protein kinase CTR1	0.0008	down
XM_043779379.1	LOC122606524	E3 ubiquitin-protein ligase HEL2-like	< 0.0001	down
XM_043767509.1	LOC122595178	DELLA protein GAI-like	< 0.0001	down
XR_006322302.1	LOC122589669	protein SKIP34, transcript variant X2	0.0012	down
XM_043782460.1	LOC122609416	protein VAPYRIN-LIKE-like	< 0.0001	down
XM_043776834.1	LOC122603964	uncharacterized LOC122603964, transcript variant X1	0.0005	down
XM_043781534.1	LOC122608426	UDP-arabinopyranose mutase 1-like	< 0.0001	down
XM_043760992.1	LOC122588792	zinc finger protein BRUTUS-like At1g74770, transcript variant X2	0.0026	down
XM_043773992.1	LOC122601231	adenylate isopentenyltransferase 5, chloroplastic	< 0.0001	down
XM_043754882.1	LOC122582485	scarecrow-like protein 14	< 0.0001	down
XM_043771867.1	LOC122599057	dirigent protein 9-like	0.0008	down
XM_043777760.1	LOC122604902	UDP-glycosyltransferase 76G1-like	< 0.0001	down
XM_043759555.1	LOC122587397	transcription factor bHLH110-like, transcript variant X1	0.0003	down
XM_043765911.1	LOC122593493	cytochrome P450 78A9-like	< 0.0001	down
XM_043767816.1	LOC122595453	protein LURP-one-related 5	< 0.0001	down
XM_043750322.1	LOC122578373	mitochondrial uncoupling protein 5-like	< 0.0001	down
XM_043757855.1	LOC122585730	auxin-responsive protein SAUR15	0.0009	down
XR_006325192.1	LOC122608252	DNA repair protein XRCC2 homolog, transcript variant X3	0.0010	down
XM_043758786.1	LOC122586800	type IV inositol polyphosphate 5-phosphatase 3-like, transcript variant X1	0.0005	down
XM_043756350.1	LOC122583982	protein TIFY 4B-like, transcript variant X1	0.0005	down
XM_043758946.1	LOC122586920	CBS domain-containing protein CBSCBSPB1-like	0.0048	down
XM_043777364.1	LOC122604467	protein CNGC15b-like	< 0.0001	down
XM_043775653.1	LOC122603040	ruBisCO large subunit-binding protein subunit alpha	0.0017	down
XM_043756300.1	LOC122583925	UDP-glycosyltransferase 76H1-like	0.0003	down
XM_043767530.1	LOC122595198	nuclear pore complex protein NUP205, transcript variant X4	0.0001	down
XM_043750711.1	LOC122578692	uncharacterized LOC122578692	< 0.0001	down
XM_043761646.1	LOC122589358	HVA22-like protein e	0.0009	down
XM_043767295.1	LOC122595016	beta-xylosidase/alpha-L-arabinofuranosidase 2-like	0.0002	down
XM_043765427.1	LOC122593082	putative protein FAR1-RELATED SEQUENCE 10, transcript variant X1	0.0041	down
XM_043758731.1	LOC122586742	probable xyloglucan 6-xylosyltransferase 5	0.0001	down
XM_043773225.1	LOC122600501	hydroxymethylglutaryl-CoA lyase, mitochondrial-like, transcript variant X1	< 0.0001	down
XM_043762337.1	LOC122590001	DDB1- and CUL4-associated factor 4-like protein 1, transcript variant X1	0.0033	down
XM_043762953.1	LOC122590781	chorion class high-cysteine HCB protein 13-like	0.0006	down
XM_043761926.1	LOC122589619	uncharacterized LOC122589619	< 0.0001	down
XM_043780031.1	LOC122607115	uncharacterized LOC122607115	0.0016	down
XR_006320828.1	LOC122580590	uncharacterized LOC122580590	0.0025	down
XM_043760319.1	LOC122588194	transmembrane protein 53-A-like	< 0.0001	down
XM_043781543.1	LOC122608435	probable protein phosphatase 2C 2	0.0001	down
XM_043776021.1	LOC122603347	protein ASPARTIC PROTEASE IN GUARD CELL 1-like	0.0059	down
XM_043778565.1	LOC122605604	putative protein phosphatase 2C 53	0.0001	down
XM_043757566.1	LOC122585433	actin-97	0.0028	down
XM_043765094.1	LOC122592782	chitin-inducible gibberellin-responsive protein 1-like, transcript variant X1	< 0.0001	down
XM_043755351.1	LOC122582922	histidine protein methyltransferase 1 homolog	< 0.0001	down
XM_043774547.1	LOC122601812	subtilisin-like protease	0.0003	down
XM_043771209.1	LOC122598622	type IV inositol polyphosphate 5-phosphatase 9	0.0004	down
XR_006321363.1	LOC122583965	uncharacterized LOC122583965	0.0011	down
XM_043783356.1	LOC122610362	uncharacterized LOC122610362	0.0027	down
XM_043756116.1	LOC122583740	gibberellin 3-beta-dioxygenase 1-like	0.0001	down
XM_043759139.1	LOC122587067	probable protein phosphatase 2C 48	< 0.0001	down

Transcript id	GeneID	Gene name/function	*P* Value	result
XM_043781243.1	LOC122608151	probable WRKY transcription factor 70, transcript variant X1	0.0018	down
XM_043768517.1	LOC122596020	caffeoyl-CoA O-methyltransferase 1	< 0.0001	down
XM_043753200.1	LOC122581058	uncharacterized LOC122581058, transcript variant X2	0.0002	down
XM_043775596.1	LOC122602982	UDP-glucuronate 4-epimerase 6-like	< 0.0001	down
XM_043782079.1	LOC122609016	scarecrow-like protein 8	< 0.0001	down
XM_043765612.1	LOC122593230	cytochrome b561 and DOMON domain-containing protein At3g25290	0.0061	down
XM_043780843.1	LOC122607795	uncharacterized LOC122607795, transcript variant X3	0.0042	down
XM_043765555.1	LOC122593186	cytochrome b561 and DOMON domain-containing protein At5g47530-like	0.0010	down
XM_043762118.1	LOC122589795	uncharacterized LOC122589795	< 0.0001	down
XM_043752257.1	LOC122579990	wall-associated receptor kinase-like 15	0.0016	down
XM_043753174.1	LOC122581030	nucleolar protein 58-like	0.0012	down
XM_043753093.1	LOC122580944	polyubiquitin, transcript variant X2	0.0012	down
XM_043766085.1	LOC122593653	probable receptor-like protein kinase At1g49730, transcript variant X2	0.0003	down
XM_043752202.1	LOC122579935	FCS-Like Zinc finger 2-like	< 0.0001	down
XM_043779464.1	LOC122606623	RAN GTPase-activating protein 2-like	0.0027	down
XM_043758864.1	LOC122586860	aminopeptidase M1-like, transcript variant X3	0.0006	down
XM_043753571.1	LOC122581352	tubulin beta-2 chain-like	0.0003	down
XM_043757406.1	LOC122585284	probable methyltransferase At1g27930	< 0.0001	down
XM_043780415.1	LOC122607439	uncharacterized LOC122607439, transcript variant X1	< 0.0001	down
XM_043777883.1	LOC122605019	serine/threonine-protein phosphatase PP1-like, transcript variant X3	0.0001	down
XM_043779109.1	LOC122606148	tRNA N(3)-methylcytidine methyltransferase METTL6, transcript variant X1	0.0001	down
XM_043769042.1	LOC122596462	zinc finger protein CONSTANS-LIKE 5-like	< 0.0001	down
XM_043759310.1	LOC122587207	uncharacterized LOC122587207, transcript variant X2	< 0.0001	down
XM_043764924.1	LOC122592641	shikimate O-hydroxycinnamoyltransferase-like	0.0043	down
XM_043759663.1	LOC122587477	probable protein phosphatase 2C 12, transcript variant X1	0.0026	down
XM_043774879.1	LOC122602155	protein phosphatase 2C 56-like	0.0013	down
XM_043783545.1	LOC122610567	uncharacterized LOC122610567	0.0059	down
XM_043775454.1	LOC122602850	glutamate dehydrogenase B, transcript variant X2	0.0060	down
XM_043777778.1	LOC122604920	receptor kinase-like protein Xa21	0.0013	down
XR_006320636.1	LOC122579450	uncharacterized LOC122579450, transcript variant X6	0.0020	down
XM_043752067.1	LOC122579830	glycosylphosphatidylinositol anchor attachment 1 protein, transcript variant X1	0.0060	down
XM_043782906.1	LOC122609944	protein NLP6-like	0.0013	down
XM_043763876.1	LOC122591614	flotillin-like protein 4	0.0005	down
XM_043775755.1	LOC122603133	general negative regulator of transcription subunit 3, transcript variant X1	0.0003	down
XM_043761230.1	LOC122589002	caffeoylshikimate esterase-like	0.0007	down
XM_043770537.1	LOC122597944	probable receptor-like protein kinase At4g10390	< 0.0001	down
XM_043756033.1	LOC122583647	uncharacterized LOC122583647	0.0001	down
XM_043766016.1	LOC122593587	dof zinc finger protein DOF1.4-like	< 0.0001	down
XM_043766548.1	LOC122594072	solute carrier family 25 member 44-like	0.0054	down
XM_043784239.1	LOC122611249	putative disease resistance RPP13-like protein 1	< 0.0001	down
XM_043778519.1	LOC122605558	syntaxin-121	0.0034	down
XM_043778337.1	LOC122605393	calmodulin-7-like, transcript variant X1	< 0.0001	down
XM_043769981.1	LOC122597381	uncharacterized LOC122597381	< 0.0001	down
XM_043757227.1	LOC122585127	leucine-rich repeat protein 1-like	< 0.0001	down
XM_043775183.1	LOC122602567	uncharacterized LOC122602567, transcript variant X1	< 0.0001	down
XM_043762759.1	LOC122590562	tetraspanin-2-like	< 0.0001	down
XM_043749909.1	LOC122578045	auxin-responsive protein IAA29, transcript variant X1	0.0004	down
XR_006322613.1	LOC122590803	uncharacterized LOC122590803, transcript variant X2	< 0.0001	down
XM_043766015.1	LOC122593585	glycine-rich cell wall structural protein 2	< 0.0001	down
XM_043759059.1	LOC122587002	60S ribosomal export protein NMD3-like, transcript variant X1	0.0024	down
XM_043777906.1	LOC122605036	aspartic proteinase PCS1-like	0.0014	down
XM_043770901.1	LOC122598309	ethylene-responsive transcription factor ERF109	< 0.0001	down
XM_043757476.1	LOC122585348	uncharacterized LOC122585348	< 0.0001	down
XM_043783447.1	LOC122610459	keratin, type II cytoskeletal 1-like	< 0.0001	down
XM_043766811.1	LOC122594337	protein GLUTAMINE DUMPER 5-like	0.0039	down
XM_043769376.1	LOC122596747	probable inactive 2-oxoglutarate-dependent dioxygenase AOP2	0.0002	down
XM_043756331.1	LOC122583962	probable LRR receptor-like serine/threonine-protein kinase RFK1, transcript variant X2	0.0006	down
XM_043751091.1	LOC122579011	protein TIFY 9-like	< 0.0001	down
XM_043750748.1	LOC122578724	prolyl endopeptidase-like	< 0.0001	down

Transcript id	GeneID	Gene name/function	*P* Value	result
XM_043769885.1	LOC122597272	inositol-tetrakisphosphate 1-kinase 5-like	< 0.0001	down
XM_043772221.1	LOC122599676	early nodulin-like protein 2	< 0.0001	down
XM_043770321.1	LOC122597752	WRKY transcription factor 22-like	0.0023	down
XM_043778695.1	LOC122605740	probable disease resistance protein At5g66900, transcript variant X1	0.0027	down
XM_043765743.1	LOC122593351	calcium-dependent protein kinase 19-like	< 0.0001	down
XM_043775344.1	LOC122602753	protein LEAD-SENSITIVE 1-like	< 0.0001	down
XM_043774459.1	LOC122601716	nucleobase-ascorbate transporter 6-like	< 0.0001	down
XM_043751195.1	LOC122579095	F-box protein SKIP14-like	< 0.0001	down
XM_043768778.1	LOC122596232	lipid phosphate phosphatase 2-like, transcript variant X1	0.0003	down
XM_043755364.1	LOC122582934	nuclear transport factor 2B-like	0.0015	down
XM_043763634.1	LOC122591366	probable xyloglucan endotransglucosylase/hydrolase protein 23	< 0.0001	down
XM_043768951.1	LOC122596384	uncharacterized LOC122596384, transcript variant X2	0.0023	down
XM_043775111.1	LOC122602429	EG45-like domain containing protein 2	0.0053	down
XM_043767446.1	LOC122595132	caffeoyl-CoA O-methyltransferase-like	0.0037	down
XM_043763540.1	LOC122591298	phosphoinositide phospholipase C 4-like	< 0.0001	down
XM_043753688.1	LOC122581455	metallothionein-like protein 1	< 0.0001	down
XM_043779272.1	LOC122606314	probable disease resistance protein At5g66900	0.0002	down
XM_043762468.1	LOC122590131	probable carboxylesterase 120	< 0.0001	down
XM_043771748.1	LOC122599266	protein LSD1-like, transcript variant X2	0.0011	down
XM_043775746.1	LOC122603126	pectinesterase 2-like	< 0.0001	down
XM_043782768.1	LOC122609827	uncharacterized LOC122609827	< 0.0001	down
XM_043751308.1	LOC122579193	inactive LRR receptor-like serine/threonine-protein kinase BIR2	< 0.0001	down
XM_043761241.1	LOC122589012	uncharacterized LOC122589012	< 0.0001	down
XM_043751945.1	LOC122579721	probable polyol transporter 4	0.0002	down
XM_043782841.1	LOC122609887	beta-1,4-mannosyl-glycoprotein 4-beta-N-acetylglucosaminyltransferase-like	< 0.0001	down
XM_043773237.1	LOC122600509	UDP-glucuronate 4-epimerase 1	0.0002	down
XM_043779237.1	LOC122606274	probable cytokinin riboside 5'-monophosphate phosphoribohydrolase LOGL10	< 0.0001	down
XM_043767076.1	LOC122594641	scarecrow-like protein 18	< 0.0001	down
XM_043777820.1	LOC122604964	glycine cleavage system H protein 2, mitochondrial	0.0001	down
XM_043780037.1	LOC122607110	catalase, transcript variant X1	0.0037	down
XM_043772909.1	LOC122600223	zinc finger A20 and AN1 domain-containing stress-associated protein 5-like	0.0042	down
XM_043771056.1	LOC122598460	lysine histidine transporter-like 8	0.0011	down
XM_043781270.1	LOC122608180	V-type proton ATPase subunit a3-like	0.0001	down
XM_043763055.1	LOC122590875	polyphenol oxidase latent form, chloroplastic-like	0.0006	down
XM_043775537.1	LOC122602925	serine/threonine-protein phosphatase BSL3-like, transcript variant X2	0.0047	down
XM_043772507.1	LOC122599900	MAPK kinase substrate protein At1g80180	0.0031	down
XM_043778950.1	LOC122605985	uncharacterized LOC122605985, transcript variant X3	0.0054	down
XM_043770551.1	LOC122597955	uncharacterized LOC122597955	< 0.0001	down
XM_043755467.1	LOC122583030	fanconi-associated nuclease 1 homolog	0.0010	down
XM_043756200.1	LOC122583820	calcium uniporter protein 6, mitochondrial-like	< 0.0001	down
XM_043773021.1	LOC122600312	two-pore potassium channel 1-like	0.0002	down
XM_043769391.1	LOC122596763	pectinesterase inhibitor 7-like	< 0.0001	down
XM_043778010.1	LOC122605130	uncharacterized LOC122605130	< 0.0001	down
XM_043768796.1	LOC122596247	VIN3-like protein 1, transcript variant X2	< 0.0001	down
XM_043772191.1	LOC122599650	heat shock factor protein HSF30, transcript variant X2	0.0002	down
XM_043759236.1	LOC122587147	heat shock 70 kDa protein 18-like	< 0.0001	down
XM_043768032.1	LOC122595626	uncharacterized LOC122595626, transcript variant X1	0.0019	down
XM_043778444.1	LOC122605490	kunitz trypsin inhibitor 5-like	< 0.0001	down
XM_043775220.1	LOC122602625	17.8 kDa class I heat shock protein-like	< 0.0001	down
XM_043773039.1	LOC122600331	probable WRKY transcription factor 53, transcript variant X2	0.0004	down
XM_043762048.1	LOC122589722	protein indeterminate-domain 16	0.0006	down
XM_043760908.1	LOC122588735	homeobox-leucine zipper protein HAT5	0.0004	down
XM_043772054.1	LOC122599528	umecyanin-like	0.0024	down
XM_043752108.1	LOC122579856	ACT domain-containing protein ACR1	0.0009	down
XM_043776631.1	LOC122603812	probable amino-acid acetyltransferase NAGS2, chloroplastic	0.0018	down
XM_043776303.1	LOC122603568	lipid phosphate phosphatase delta-like	< 0.0001	down
XM_043760814.1	LOC122588658	F-box protein At5g39250, transcript variant X2	< 0.0001	down
XM_043777081.1	LOC122604184	disease resistance protein Roq1-like	< 0.0001	down
XM_043758165.1	LOC122586054	auxin transporter-like protein 2	0.0059	down
XM_043750514.1	LOC122578536	protein NRT1/ PTR FAMILY 7.3	0.0001	down
XM_043768917.1	LOC122596352	uncharacterized LOC122596352	< 0.0001	down
XM_043772383.1	LOC122599798	elongation factor 1-alpha-like	0.0003	down

Transcript id	GeneID	Gene name/function	*P* Value	result
XM_043773012.1	LOC122600306	zinc finger protein ZAT10-like	0.0021	down
XM_043757294.1	LOC122585185	ethylene-responsive transcription factor ERF025-like	< 0.0001	down
XM_043755151.1	LOC122582726	macro domain-containing protein VPA0103, transcript variant X2	< 0.0001	down
XM_043775465.1	LOC122602857	phospholipase A1-Ibeta2, chloroplastic-like	< 0.0001	down
XM_043753692.1	LOC122581459	receptor-like protein kinase HERK 1	0.0062	down
XM_043763570.1	LOC122591315	protein EXORDIUM-like 2	0.0020	down
XM_043761293.1	LOC122589051	phosphate transporter PHO1 homolog 9-like	< 0.0001	down
XM_043775671.1	LOC122603057	uncharacterized LOC122603057	< 0.0001	down
XM_043774828.1	LOC122602099	putative ripening-related protein 1	< 0.0001	down
XM_043757404.1	LOC122585282	transmembrane 9 superfamily member 3-like, transcript variant X1	< 0.0001	down
XM_043766923.1	LOC122594475	protein MIZU-KUSSEI 1-like	0.0002	down
XM_043751963.1	LOC122579738	probable galactinol--sucrose galactosyltransferase 5	< 0.0001	down
XM_043751149.1	LOC122579057	probable inactive receptor kinase At1g48480	0.0029	down
XM_043766523.1	LOC122594041	uncharacterized LOC122594041	0.0002	down
XM_043756111.1	LOC122583732	cytochrome P450 94C1	< 0.0001	down
XM_043755559.1	LOC122583126	non-specific lipid transfer protein GPI-anchored 16-like	0.0021	down
XM_043758336.1	LOC122586342	uncharacterized LOC122586342	0.0062	down
XR_006321751.1	LOC122585652	uncharacterized LOC122585652, transcript variant X2	0.0015	down
XM_043784286.1	LOC122611285	probable polyamine transporter At1g31830, transcript variant X1	< 0.0001	down
XM_043761043.1	LOC122588832	queuosine salvage protein-like	< 0.0001	down
XM_043767111.1	LOC122594688	protein LURP-one-related 5-like	< 0.0001	down
XR_006323117.1	LOC122594836	uncharacterized LOC122594836, transcript variant X2	0.0029	down
XM_043753070.1	LOC122580927	glutathione S-transferase APIC-like	0.0002	down
XM_043773716.1	LOC122600937	uncharacterized LOC122600937	< 0.0001	down
XM_043759360.1	LOC122587247	F-box protein At1g78280	< 0.0001	down
XM_043783181.1	LOC122610188	23 kDa jasmonate-induced protein-like	< 0.0001	down
XM_043755582.1	LOC122583151	uncharacterized LOC122583151	< 0.0001	down
XM_043763249.1	LOC122591056	vesicle-associated protein 4-1-like	< 0.0001	down
XM_043770684.1	LOC122598073	oxysterol-binding protein-related protein 4C	< 0.0001	down
XM_043784223.1	LOC122611233	F-box protein At2g26850-like	0.0004	down
XM_043781125.1	LOC122608043	probable WRKY transcription factor 70	< 0.0001	down
XM_043761432.1	LOC122589182	zinc finger AN1 domain-containing stress-associated protein 12	0.0053	down
XM_043751231.1	LOC122579124	ADP-ribosylation factor 1-like	< 0.0001	down
XM_043765635.1	LOC122593249	phosphatidylinositol:ceramide inositolphosphotransferase 1-like	< 0.0001	down
XM_043755055.1	LOC122582636	uncharacterized LOC122582636	< 0.0001	down
XM_043762920.1	LOC122590744	leucine-rich repeat receptor-like serine/threonine-protein kinase BAM3	0.0002	down
XM_043758027.1	LOC122585909	protein HAIKU1-like	< 0.0001	down
XM_043761397.1	LOC122589151	probable galacturonosyltransferase-like 1	0.0007	down
XM_043750546.1	LOC122578561	UDP-glucuronate 4-epimerase 6-like	< 0.0001	down
XM_043767353.1	LOC122595063	FT-interacting protein 7-like	< 0.0001	down
XM_043759017.1	LOC122586966	uncharacterized acetyltransferase At3g50280-like, transcript variant X3	0.0004	down
XM_043775898.1	LOC122603238	protein NLP4-like	0.0048	down
XM_043766494.1	LOC122594018	short-chain dehydrogenase reductase 3b-like	0.0002	down
XM_043776147.1	LOC122603444	transcription factor TGA2.3-like, transcript variant X2	0.0005	down
XM_043755455.1	LOC122583017	probable serine/threonine-protein kinase PBL7	0.0004	down
XM_043750429.1	LOC122578458	cytochrome P450 78A5-like	< 0.0001	down
XM_043772985.1	LOC122600282	probable carboxylesterase 5	0.0005	down
XM_043770698.1	LOC122598091	probable LRR receptor-like serine/threonine-protein kinase At1g67720	< 0.0001	down
XM_043752827.1	LOC122580556	cytosolic sulfotransferase 12-like	< 0.0001	down
XM_043765686.1	LOC122593294	U-box domain-containing protein 17	< 0.0001	down
XM_043751777.1	LOC122579590	uncharacterized LOC122579590, transcript variant X2	< 0.0001	down
XM_043765860.1	LOC122593448	probable indole-3-pyruvate monooxygenase YUCCA5	< 0.0001	down
XM_043756017.1	LOC122583633	uncharacterized LOC122583633	0.0002	down
XM_043771888.1	LOC122599383	pollen receptor-like kinase 3	< 0.0001	down
XM_043762042.1	LOC122589717	TOM1-like protein 4	< 0.0001	down
XM_043783721.1	LOC122610762	uncharacterized LOC122610762	0.0001	down
XM_043751947.1	LOC122579723	aldehyde oxidase GLOX-like	0.0003	down
XM_043770562.1	LOC122597967	MLO-like protein 14	< 0.0001	down
XM_043762653.1	LOC122590450	probable aspartic proteinase GIP2	0.0001	down
XR_006322901.1	LOC122593943	uncharacterized LOC122593943	0.0029	down
XM_043776052.1	LOC122603372	probable aspartyl protease At4g16563	< 0.0001	down
XM_043778328.1	LOC122605387	ubiquitin-conjugating enzyme E2 20	< 0.0001	down

Transcript id	GeneID	Gene name/function	*P* Value	result
XM_043757778.1	LOC122585648	60S ribosomal protein L10-like	< 0.0001	down
XM_043755248.1	LOC122582815	transcription repressor OFP16-like	0.0014	down
XM_043751772.1	LOC122579583	polyamine oxidase 2-like	0.0003	down
XM_043761970.1	LOC122589651	eukaryotic translation initiation factor 3 subunit G	0.0024	down
XM_043771050.1	LOC122598454	nudix hydrolase 25	0.0026	down
XM_043764715.1	LOC122592476	uncharacterized LOC122592476	0.0026	down
XM_043753192.1	LOC122581054	leucine-rich repeat receptor-like protein kinase PXC1	0.0010	down
XM_043776715.1	LOC122603876	transcription factor bHLH18-like	0.0038	down
XM_043775343.1	LOC122602752	2-methylpropanoate--CoA ligase CCL4-like	0.0060	down
XM_043762588.1	LOC122590257	mini zinc finger protein 3-like	0.0004	down
XM_043751367.1	LOC122579246	phytohormone-binding protein CSBP-like	0.0023	down
XM_043770876.1	LOC122598280	protein DMR6-LIKE OXYGENASE 2-like	0.0063	down
XM_043782560.1	LOC122609524	probable LRR receptor-like serine/threonine-protein kinase IRK	< 0.0001	down
XM_043757259.1	LOC122585149	glycine-rich protein 5-like	< 0.0001	down
XM_043765734.1	LOC122593340	uncharacterized LOC122593340	0.0004	down
XM_043777875.1	LOC122605011	serine carboxypeptidase-like 51	< 0.0001	down
XM_043779611.1	LOC122606750	pathogen-associated molecular patterns-induced protein A70-like	0.0018	down
XM_043765855.1	LOC122593444	cysteine-rich and transmembrane domain-containing protein WIH2-like	0.0007	down
XM_043767878.1	LOC122595510	protein BIG GRAIN 1-like B	< 0.0001	down
XM_043784379.1	LOC122611359	protein DA1, transcript variant X1	< 0.0001	down
XM_043776659.1	LOC122603836	uncharacterized LOC122603836	0.0008	down
XM_043765640.1	LOC122593255	nuclear pore complex protein NUP98A-like	< 0.0001	down
XM_043776578.1	LOC122603777	probable glutamate carboxypeptidase AMP1	0.0003	down
XM_043763406.1	LOC122591186	vacuolar cation/proton exchanger 3-like	0.0052	down
XM_043780797.1	LOC122607763	peptidyl-prolyl cis-trans isomerase CYP18-4, transcript variant X3	0.0020	down
XM_043750702.1	LOC122578683	probable galacturonosyltransferase-like 3	< 0.0001	down
XM_043757361.1	LOC122585243	heat stress transcription factor A-3-like	< 0.0001	down
XM_043753563.1	LOC122581346	COBRA-like protein 7	< 0.0001	down
XM_043773480.1	LOC122600727	U-box domain-containing protein 6-like	0.0042	down
XM_043774230.1	LOC122601472	EG45-like domain containing protein	< 0.0001	down
XM_043778647.1	LOC122605692	protein DETOXIFICATION 49-like	< 0.0001	down
XM_043752243.1	LOC122579978	heavy metal-associated isoprenylated plant protein 21-like	0.0007	down
XM_043773250.1	LOC122600519	probable WRKY transcription factor 11	< 0.0001	down
XM_043769383.1	LOC122596753	U-box domain-containing protein 7	0.0006	down
XM_043758624.1	LOC122586641	phosphate transporter PHO1 homolog 3-like	0.0044	down
XM_043752032.1	LOC122579798	putative clathrin assembly protein At4g40080	< 0.0001	down
XM_043763504.1	LOC122591266	serine/threonine-protein kinase RIPK-like	< 0.0001	down
XM_043776237.1	LOC122603513	1-aminocyclopropane-1-carboxylate synthase 3-like	< 0.0001	down
XM_043758926.1	LOC122586904	methionine aminopeptidase 1D, chloroplastic/mitochondrial, transcript variant X1	< 0.0001	down
XM_043759210.1	LOC122587124	uncharacterized LOC122587124, transcript variant X3	< 0.0001	down
XM_043775914.1	LOC122603253	probable serine/threonine-protein kinase CST, transcript variant X2	< 0.0001	down
XM_043773654.1	LOC122600876	berberine bridge enzyme-like 8	0.0032	down
XM_043774814.1	LOC122602081	glutamate receptor 3.4-like	0.0017	down
XM_043770540.1	LOC122597946	ethylene-responsive transcription factor 12-like	< 0.0001	down
XM_043750319.1	LOC122578371	uncharacterized LOC122578371	< 0.0001	down
XM_043771468.1	LOC122599021	auxin response factor 7-like	< 0.0001	down
XR_006324462.1	LOC122603470	mitochondrial fission protein ELM1, transcript variant X2	< 0.0001	down
XM_043752282.1	LOC122580013	uncharacterized LOC122580013	0.0031	down
XM_043767305.1	LOC122595026	vacuolar iron transporter homolog 4-like	0.0001	down
XM_043772933.1	LOC122600246	BES1/BZR1 homolog protein 4-like	0.0059	down
XM_043772602.1	LOC122599968	nuclear pore complex protein NUP98A, transcript variant X2	< 0.0001	down
XM_043774532.1	LOC122601797	nuclear transcription factor Y subunit C-3-like	0.0019	down
XM_043768155.1	LOC122595726	UPF0496 protein 4-like	< 0.0001	down
XM_043756216.1	LOC122583835	NAC domain-containing protein 90-like	0.0004	down
XM_043781487.1	LOC122608388	E-beta-caryophyllene synthase-like	0.0005	down
XM_043754585.1	LOC122582224	alpha carbonic anhydrase 7-like	< 0.0001	down
XM_043761819.1	LOC122589520	uncharacterized protein At5g65660-like	< 0.0001	down
XM_043760474.1	LOC122588369	IQ domain-containing protein IQM1-like	< 0.0001	down
XM_043767306.1	LOC122595027	vacuolar iron transporter homolog 4-like	0.0044	down
XM_043776217.1	LOC122603501	UDP-glucose 6-dehydrogenase 3-like	< 0.0001	down
XM_043778920.1	LOC122605955	probable WRKY transcription factor 27	0.0014	down
XM_043754195.1	LOC122581881	indole-3-pyruvate monooxygenase YUCCA6	< 0.0001	down
XM_043773658.1	LOC122600878	omega-3 fatty acid desaturase, chloroplastic-like	0.0004	down

Transcript id	GeneID	Gene name/function	P Value	result
XM_043779016.1	LOC122606051	uncharacterized LOC122606051, transcript variant X2	< 0.0001	down
XM_043768579.1	LOC122596066	AP2/ERF and B3 domain-containing transcription factor At1g50680-like, transcript variant X1	0.0011	down
XM_043757051.1	LOC122584958	uncharacterized LOC122584958	< 0.0001	down
XM_043753872.1	LOC122581630	hsp70 nucleotide exchange factor fes1-like, transcript variant X2	< 0.0001	down
XM_043778959.1	LOC122605993	uncharacterized LOC122605993, transcript variant X4	< 0.0001	down
XM_043776373.1	LOC122603620	uncharacterized LOC122603620	< 0.0001	down
XM_043770911.1	LOC122598318	putative phospholipid-transporting ATPase 9	0.0009	down
XM_043759156.1	LOC122587081	actin-depolymerizing factor 2	< 0.0001	down
XM_043766338.1	LOC122593884	uncharacterized LOC122593884	< 0.0001	down
XR_006320747.1	LOC122580127	uncharacterized LOC122580127	0.0006	down
XM_043776252.1	LOC122603526	heavy metal-associated isoprenylated plant protein 34-like	0.0002	down
XM_043760074.1	LOC122587917	ethylene-responsive transcription factor CRF1-like	0.0056	down
XM_043780170.1	LOC122607234	septin and tuftelin-interacting protein 1 homolog 1-like, transcript variant X2	< 0.0001	down
XM_043780888.1	LOC122607833	dof zinc finger protein DOF2.4-like	< 0.0001	down
XM_043764064.1	LOC122591830	CASP-like protein 4D1	0.0012	down
XM_043751882.1	LOC122579667	protein ZW2-like	< 0.0001	down
XM_043764767.1	LOC122592519	protein NRT1/ PTR FAMILY 5.6-like	< 0.0001	down
XM_043779160.1	LOC122606198	two-on-two hemoglobin-3	< 0.0001	down
XM_043765593.1	LOC122593214	transcription factor TCP9-like	< 0.0001	down
XM_043762364.1	LOC122590027	transcription factor bHLH18-like	< 0.0001	down
XM_043763020.1	LOC122590844	mitogen-activated protein kinase kinase kinase 18-like	< 0.0001	down
XM_043776646.1	LOC122603825	uncharacterized LOC122603825	0.0011	down
XM_043763018.1	LOC122590842	tubulin alpha chain	< 0.0001	down
XM_043784364.1	LOC122611343	S-adenosylmethionine decarboxylase proenzyme-like	< 0.0001	down
XM_043765119.1	LOC122592804	RHOMBOID-like protein 1	< 0.0001	down
XM_043757729.1	LOC122585607	protein HIRA	0.0044	down
XM_043766001.1	LOC122593574	indole-3-acetic acid-amido synthetase GH3.5-like	< 0.0001	down
XM_043756326.1	LOC122583958	lachrymatory-factor synthase-like	0.0008	down
XM_043778664.1	LOC122605706	type I inositol polyphosphate 5-phosphatase 8	< 0.0001	down
XM_043767475.1	LOC122595153	carbonic anhydrase 2, transcript variant X1	0.0058	down
XM_043780578.1	LOC122607572	eugenol synthase 1-like, transcript variant X1	< 0.0001	down
XM_043759600.1	LOC122587434	probable plastid-lipid-associated protein 14, chloroplastic, transcript variant X2	0.0040	down
XM_043754114.1	LOC122581821	uncharacterized LOC122581821	< 0.0001	down
XM_043780392.1	LOC122607421	protein TIFY 10B-like	< 0.0001	down
XM_043769108.1	LOC122596521	DEAD-box ATP-dependent RNA helicase 52-like	0.0022	down
XM_043775923.1	LOC122603260	probable magnesium transporter NIPA8	< 0.0001	down
XR_006322836.1	LOC122593459	uncharacterized LOC122593459	0.0007	down
XM_043758384.1	LOC122586390	dehydration-responsive element-binding protein 1E-like	< 0.0001	down
XM_043775539.1	LOC122602926	putative disease resistance protein At1g59780, transcript variant X1	< 0.0001	down
XM_043758728.1	LOC122586739	galactoside 2-alpha-L-fucosyltransferase-like	0.0003	down
XM_043757554.1	LOC122585421	uncharacterized LOC122585421	< 0.0001	down
XM_043773826.1	LOC122601049	putative leucine-rich repeat receptor-like serine/threonine-protein kinase At2g24130	< 0.0001	down
XM_043755238.1	LOC122582801	E3 ubiquitin-protein ligase PUB23-like	0.0001	down
XM_043752419.1	LOC122580144	IQ domain-containing protein IQM2-like	< 0.0001	down
XM_043769296.1	LOC122596677	cyclin-U4-1-like	0.0006	down
XM_043773452.1	LOC122600698	ethylene-responsive transcription factor RAP2-3-like	< 0.0001	down
XM_043782098.1	LOC122609032	5-oxoprolinase, transcript variant X1	< 0.0001	down
XM_043756005.1	LOC122583621	protein ACCELERATED CELL DEATH 6-like	< 0.0001	down
XM_043757118.1	LOC122585018	probable carboxylesterase 15	0.0024	down
XM_043759346.1	LOC122587235	TORTIFOLIA1-like protein 3	0.0051	down
XM_043759566.1	LOC122587405	exportin-T, transcript variant X1	< 0.0001	down
XM_043776142.1	LOC122603443	serine/threonine-protein kinase/endoribonuclease IRE1b-like	< 0.0001	down
XM_043752319.1	LOC122580047	uncharacterized LOC122580047, transcript variant X1	< 0.0001	down
XM_043772394.1	LOC122599808	probable sphingolipid transporter spinster homolog 2	0.0042	down
XM_043771306.1	LOC122598819	protein LATE ELONGATED HYPOCOTYL-like, transcript variant X1	0.0023	down
XM_043767638.1	LOC122595296	mitogen-activated protein kinase kinase 5	< 0.0001	down
XM_043753209.1	LOC122581067	3-ketoacyl CoA thiolase 1, peroxisomal-like	< 0.0001	down
XM_043778764.1	LOC122605805	glucan endo-1,3-beta-glucosidase-like, transcript variant X2	0.0052	down
XM_043784234.1	LOC122611246	delta(12) fatty acid desaturase DES8.11-like	0.0023	down
XM_043782561.1	LOC122609525	O-fucosyltransferase 19-like	0.0018	down

Transcript id	GeneID	Gene name/function	P Value	result
XM_043770978.1	LOC122598383	putative pentatricopeptide repeat-containing protein At3g15930, transcript variant X2	0.0004	down
XM_043770791.1	LOC122598188	putative F-box protein At1g32420	0.0002	down
XR_006323282.1	LOC122595972	uncharacterized LOC122595972, transcript variant X7	< 0.0001	down
XM_043775301.1	LOC122602710	MLO protein homolog 1-like	< 0.0001	down
XR_006321656.1	LOC122585084	uncharacterized LOC122585084, transcript variant X4	0.0002	down
XM_043752469.1	LOC122580194	uncharacterized LOC122580194	< 0.0001	down
XM_043752528.1	LOC122580251	protein DA1-related 3-like, transcript variant X2	< 0.0001	down
XM_043763394.1	LOC122591173	putative F-box protein At1g32420	0.0011	down
XM_043760799.1	LOC122588643	dehydration-responsive element-binding protein 1E-like	< 0.0001	down
XM_043761442.1	LOC122589191	short-chain dehydrogenase TIC 32 B, chloroplastic	< 0.0001	down
XM_043776525.1	LOC122603737	uncharacterized LOC122603737	0.0036	down
XM_043780024.1	LOC122607106	trans-resveratrol di-O-methyltransferase-like	< 0.0001	down
XM_043776539.1	LOC122603748	DEAD-box ATP-dependent RNA helicase 37-like	< 0.0001	down
XM_043770117.1	LOC122597533	aspartic proteinase Asp1-like	0.0056	down
XM_043778953.1	LOC122605988	pentatricopeptide repeat-containing protein At5g03800, transcript variant X3	0.0009	down
XM_043781701.1	LOC122608605	uncharacterized LOC122608605	< 0.0001	down
XM_043765626.1	LOC122593241	BTB/POZ domain-containing protein At4g30940	0.0001	down
XM_043768391.1	LOC122595913	probable peroxygenase 4	0.0003	down
XM_043784210.1	LOC122611225	cytochrome P450 CYP72A219-like	0.0014	down
XM_043756162.1	LOC122583790	non-specific lipid transfer protein GPI-anchored 5-like	< 0.0001	down
XM_043768715.1	LOC122596184	uncharacterized LOC122596184, transcript variant X1	0.0014	down
XM_043750390.1	LOC122578429	U-box domain-containing protein 45-like	< 0.0001	down
XM_043767212.1	LOC122594927	uncharacterized LOC122594927	< 0.0001	down
XM_043754920.1	LOC122582515	alpha carbonic anhydrase 7-like	0.0021	down
XM_043751078.1	LOC122579000	probable receptor-like protein kinase At1g80640, transcript variant X1	0.0008	down
XM_043766437.1	LOC122593961	mitogen-activated protein kinase kinase kinase 18-like	< 0.0001	down
XM_043763322.1	LOC122591112	delta(12)-fatty-acid desaturase FAD2-like	< 0.0001	down
XM_043776825.1	LOC122603957	E3 ubiquitin-protein ligase WAV3	< 0.0001	down
XM_043772413.1	LOC122599826	thioredoxin 1-like	< 0.0001	down
XM_043767304.1	LOC122595024	vacuolar iron transporter homolog 4-like	0.0001	down
XM_043781121.1	LOC122608039	UDP-glycosyltransferase 85C1-like	0.0033	down
XM_043757919.1	LOC122585794	protein EARLY-RESPONSIVE TO DEHYDRATION 7, chloroplastic-like	< 0.0001	down
XM_043764030.1	LOC122591793	disease resistance protein RUN1-like	0.0028	down
XM_043756299.1	LOC122583924	carotenoid cleavage dioxygenase 8 homolog B, chloroplastic-like	< 0.0001	down
XM_043751027.1	LOC122578961	proline dehydrogenase 2, mitochondrial-like	< 0.0001	down
XM_043770306.1	LOC122597738	protein trichome birefringence-like 11, transcript variant X2	< 0.0001	down
XM_043754490.1	LOC122582132	epoxide hydrolase A-like	< 0.0001	down
XM_043774699.1	LOC122601966	auxin-responsive protein IAA16-like	0.0011	down
XM_043780691.1	LOC122607672	uncharacterized LOC122607672	0.0013	down
XM_043782633.1	LOC122609685	uncharacterized LOC122609685	< 0.0001	down
XM_043763518.1	LOC122591278	uncharacterized LOC122591278	< 0.0001	down
XM_043776215.1	LOC122603499	UDP-glycosyltransferase 88B1-like	< 0.0001	down
XM_043753039.1	LOC122580894	WAT1-related protein At2g37460-like	< 0.0001	down
XM_043753134.1	LOC122580990	zinc finger protein 10	0.0031	down
XM_043751795.1	LOC122579598	endoglucanase 6-like	0.0030	down
XM_043757308.1	LOC122585197	delta(7)-sterol-C5(6)-desaturase-like	0.0002	down
XM_043756713.1	LOC122584668	WAT1-related protein At1g70260	< 0.0001	down
XM_043766551.1	LOC122594078	uncharacterized protein C24B11.05-like	0.0001	down
XM_043776436.1	LOC122603668	ubiquitin-conjugating enzyme E2 22-like	0.0018	down
XM_043769040.1	LOC122596459	uncharacterized LOC122596459	< 0.0001	down
XM_043751674.1	LOC122579495	uncharacterized LOC122579495	< 0.0001	down
XM_043756666.1	LOC122584610	uncharacterized mitochondrial protein AtMg01010-like	0.0045	down
XM_043761872.1	LOC122589569	reticulocyte-binding protein 2 homolog a	0.0027	down
XM_043779633.1	LOC122606776	CDT1-like protein a, chloroplastic	< 0.0001	down
XM_043751745.1	LOC122579556	phosphatidylinositol 4-kinase gamma 5-like	< 0.0001	down
XM_043760790.1	LOC122588634	bidirectional sugar transporter N3-like	< 0.0001	down
XM_043779023.1	LOC122606058	inverted formin-2-like	< 0.0001	down
XM_043751970.1	LOC122579743	uncharacterized LOC122579743	< 0.0001	down
XM_043773423.1	LOC122600670	myb family transcription factor EFM	< 0.0001	down
XM_043750665.1	LOC122578659	B3 domain-containing protein Os03g0120900-like, transcript variant X2	0.0005	down
XM_043764912.1	LOC122592629	uncharacterized LOC122592629	< 0.0001	down

Transcript id	GeneID	Gene name/function	P Value	result
XM_043751644.1	LOC122579460	probable receptor-like protein kinase At1g11050	< 0.0001	down
XM_043766736.1	LOC122594262	protein NRT1/ PTR FAMILY 4.5-like	0.0027	down
XM_043779991.1	LOC122607070	dirigent protein 22-like	< 0.0001	down
XM_043773744.1	LOC122600966	dual specificity protein kinase zak2-like	0.0044	down
XM_043752891.1	LOC122580625	flavonoid 3'-monooxygenase-like	< 0.0001	down
XM_043765072.1	LOC122592766	non-lysosomal glucosylceramidase-like, transcript variant X7	0.0024	down
XM_043777556.1	LOC122604686	L-type lectin-domain containing receptor kinase IX.1-like	0.0029	down
XM_043776138.1	LOC122603438	alcohol acyltransferase 9-like	0.0016	down
XM_043764845.1	LOC122592583	helicase-like transcription factor CHR28	< 0.0001	down
XM_043775146.1	LOC122602540	wall-associated receptor kinase-like 2	< 0.0001	down
XM_043771277.1	LOC122598702	probable carboxylesterase 6	0.0033	down
XM_043760893.1	LOC122588721	protein RADIALIS-like 3	< 0.0001	down
XM_043761649.1	LOC122589362	uncharacterized LOC122589362	0.0002	down
XM_043768553.1	LOC122596049	probable mannitol dehydrogenase	0.0038	down
XM_043754409.1	LOC122582061	dirigent protein 21-like	< 0.0001	down
XM_043777782.1	LOC122604923	protein FAR1-RELATED SEQUENCE 11-like	0.0020	down
XM_043765697.1	LOC122593305	monothiol glutaredoxin-S1-like	< 0.0001	down
XM_043783240.1	LOC122610245	uncharacterized LOC122610245	< 0.0001	down
XM_043776319.1	LOC122603581	patatin-like protein 7	< 0.0001	down
XM_043757474.1	LOC122585345	ethylene-responsive transcription factor ERF061-like	0.0019	down
XR_006323566.1	LOC122598260	uncharacterized LOC122598260	< 0.0001	down
XM_043771713.1	LOC122599234	palmitoyl-acyl carrier protein thioesterase, chloroplastic, transcript variant X2	< 0.0001	down
XR_006323514.1	LOC122598017	uncharacterized LOC122598017	0.0018	down
XM_043749920.1	LOC122578052	uncharacterized LOC122578052	0.0007	down
XM_043763336.1	LOC122591121	fimbrin-1-like	< 0.0001	down
XM_043751954.1	LOC122579729	dof zinc finger protein DOF5.6	< 0.0001	down
XM_043781527.1	LOC122608421	uncharacterized LOC122608421, transcript variant X1	0.0024	down
XM_043778758.1	LOC122605802	protein SINE1-like	0.0006	down
XM_043759179.1	LOC122587101	metacaspase-1-like	< 0.0001	down
XM_043781262.1	LOC122608172	dof zinc finger protein DOF3.1-like	0.0004	down
XM_043761853.1	LOC122589555	anoctamin-like protein At1g73020, transcript variant X2	< 0.0001	down
XM_043754323.1	LOC122581987	phosphomethylpyrimidine synthase, chloroplastic	< 0.0001	down
XM_043776275.1	LOC122603549	protein DA1-related 2-like	< 0.0001	down
XM_043778189.1	LOC122605280	phosphoethanolamine N-methyltransferase 3-like	0.0033	down
XM_043780098.1	LOC122607169	xylan glycosyltransferase MUCI21-like	0.0036	down
XM_043765742.1	LOC122593349	protein CYPRO4-like	0.0029	down
XM_043751929.1	LOC122579706	zinc finger CCCH domain-containing protein 34-like	< 0.0001	down
XM_043768177.1	LOC122595747	YTH domain-containing protein ECT1-like, transcript variant X2	< 0.0001	down
XR_006321641.1	LOC122585027	uncharacterized LOC122585027	< 0.0001	down
XM_043750216.1	LOC122578289	E3 ubiquitin-protein ligase ATL6-like	0.0039	down
XM_043766890.1	LOC122594437	early nodulin-like protein 1	0.0010	down
XM_043775696.1	LOC122603083	probable protein phosphatase 2C 33, transcript variant X2	0.0048	down
XM_043752995.1	LOC122580839	serine/threonine-protein phosphatase BSL1-like, transcript variant X1	< 0.0001	down
XM_043753677.1	LOC122581445	uridine 5'-monophosphate synthase-like	< 0.0001	down
XM_043776446.1	LOC122603676	S-type anion channel SLAH2-like, transcript variant X1	< 0.0001	down
XM_043772717.1	LOC122600063	F-box protein At2g02240-like, transcript variant X1	0.0022	down
XM_043768003.1	LOC122595606	U-box domain-containing protein 4	< 0.0001	down
XM_043763835.1	LOC122591573	putative late blight resistance protein homolog R1A-10	0.0007	down
XM_043778193.1	LOC122605286	uncharacterized LOC122605286, transcript variant X1	0.0017	down
XM_043777660.1	LOC122604794	adhesive plaque matrix protein-like	< 0.0001	down
XM_043758903.1	LOC122586886	calcium-transporting ATPase 2, plasma membrane-type-like	< 0.0001	down
XM_043761609.1	LOC122589324	RING-H2 finger protein ATL74-like	< 0.0001	down
XM_043784082.1	LOC122611114	uncharacterized LOC122611114	< 0.0001	down
XM_043781803.1	LOC122608706	uncharacterized LOC122608706, transcript variant X2	0.0015	down
XM_043770549.1	LOC122597954	ACD11 homolog protein	0.0002	down
XM_043772296.1	LOC122599737	glutaredoxin-C6-like	0.0028	down
XM_043756410.1	LOC122584166	classical arabinogalactan protein 1-like	< 0.0001	down
XM_043780638.1	LOC122607624	uncharacterized LOC122607624	< 0.0001	down
XM_043768284.1	LOC122595832	60S ribosomal protein L18a-like protein, transcript variant X1	0.0002	down
XM_043762346.1	LOC122590008	putative UPF0481 protein At3g02645	< 0.0001	down
XM_043757338.1	LOC122585223	uncharacterized LOC122585223	0.0004	down
XM_043757176.1	LOC122585080	monothiol glutaredoxin-S2-like	< 0.0001	down
XM_043778589.1	LOC122605632	leucine-rich repeat receptor-like serine/threonine-protein kinase BAM1	0.0043	down

Transcript id	GeneID	Gene name/function	*P* Value	result
XM_043766031.1	LOC122593606	protein FANTASTIC FOUR 1-like	0.0048	down
XR_006321691.1	LOC122585333	uncharacterized LOC122585333	< 0.0001	down
XM_043765228.1	LOC122592902	E3 ubiquitin-protein ligase MBR2, transcript variant X3	< 0.0001	down
XM_043756644.1	LOC122584545	mitogen-activated protein kinase 9-like	< 0.0001	down
XR_006324830.1	LOC122606191	uncharacterized LOC122606191	< 0.0001	down
XM_043771731.1	LOC122599251	protein ANTAGONIST OF LIKE HETEROCHROMATIN PROTEIN 1-like	< 0.0001	down
XM_043778630.1	LOC122605677	probable prolyl 4-hydroxylase 10	0.0003	down
XM_043771970.1	LOC122599454	uncharacterized LOC122599454, transcript variant X1	< 0.0001	down
XM_043753046.1	LOC122580901	myb family transcription factor PHL7-like	0.0013	down
XM_043781398.1	LOC122608307	ABC transporter A family member 2-like, transcript variant X1	0.0012	down
XM_043757124.1	LOC122585025	lipid phosphate phosphatase 2-like	0.0002	down
XM_043766162.1	LOC122593715	mitogen-activated protein kinase kinase kinase 18-like	0.0001	down
XM_043756748.1	LOC122584700	dehydration-responsive element-binding protein 1D-like	< 0.0001	down
XM_043776105.1	LOC122603413	jasmonate-induced oxygenase 2-like	< 0.0001	down
XM_043778376.1	LOC122605425	uncharacterized LOC122605425	< 0.0001	down
XM_043784191.1	LOC122611210	protein NRT1/ PTR FAMILY 4.6-like, transcript variant X1	< 0.0001	down
XR_006322240.1	LOC122589248	uncharacterized LOC122589248	< 0.0001	down
XM_043772523.1	LOC122599913	3-ketoacyl-CoA synthase 9-like	< 0.0001	down
XR_006320761.1	LOC122580241	uncharacterized LOC122580241	< 0.0001	down
XM_043767838.1	LOC122595471	RNA demethylase ALKBH9B-like	< 0.0001	down
XM_043752308.1	LOC122580038	probable aspartyl protease At4g16563	< 0.0001	down
XR_006322255.1	LOC122589377	uncharacterized LOC122589377	0.0063	down
XM_043752332.1	LOC122580061	class V chitinase CHIT5-like	< 0.0001	down
XM_043776827.1	LOC122603960	dehydration-responsive element-binding protein 1D-like	0.0001	down
XM_043764914.1	LOC122592632	trifunctional UDP-glucose 4,6-dehydratase/UDP-4-keto-6-deoxy-D-glucose 3,5-epimerase/UDP-4-keto-L-rhamnose-reductase RHM1-like	< 0.0001	down
XM_043760402.1	LOC122588288	protein JINGUBANG-like	0.0041	down
XM_043752978.1	LOC122580818	thaumatin-like protein	< 0.0001	down
XM_043764032.1	LOC122591794	disease resistance protein RUN1-like	0.0003	down
XM_043773206.1	LOC122600486	G2/mitotic-specific cyclin S13-7-like, transcript variant X2	0.0046	down
XM_043761414.1	LOC122589165	uncharacterized LOC122589165	< 0.0001	down
XM_043765478.1	LOC122593121	uncharacterized LOC122593121	< 0.0001	down
XM_043770424.1	LOC122597849	probable LRR receptor-like serine/threonine-protein kinase At1g14390, transcript variant X4	0.0002	down
XM_043756735.1	LOC122584688	nudix hydrolase 17, mitochondrial-like	0.0015	down
XM_043777080.1	LOC122604183	disease resistance protein Roq1-like	< 0.0001	down
XM_043751797.1	LOC122579600	geraniol 8-hydroxylase-like	< 0.0001	down
XM_043765657.1	LOC122593270	purple acid phosphatase 2-like	< 0.0001	down
XM_043765544.1	LOC122593174	9-cis-epoxycarotenoid dioxygenase NCED1, chloroplastic-like	0.0044	down
XM_043782699.1	LOC122609756	cyanidin-3-O-glucoside 2-O-glucuronosyltransferase-like	0.0041	down
XM_043773892.1	LOC122601121	sodium transporter HKT1-like	0.0011	down
XM_043751895.1	LOC122579673	uncharacterized LOC122579673, transcript variant X1	< 0.0001	down
XM_043784048.1	LOC122611086	probable 2-oxoglutarate-dependent dioxygenase AOP1	0.0048	down
XM_043779370.1	LOC122606501	TMV resistance protein N-like, transcript variant X2	0.0050	down
XM_043767906.1	LOC122595534	probable histone H2A variant 3	0.0006	down
XM_043774829.1	LOC122602100	putative ripening-related protein 2	0.0003	down
XM_043781150.1	LOC122608064	ER lumen protein-retaining receptor erd-2.2-like	0.0017	down
XM_043757437.1	LOC122585309	sulfite exporter TauE/SafE family protein 4-like	< 0.0001	down
XM_043769436.1	LOC122596792	trimethyltridecatetraene synthase-like	< 0.0001	down
XM_043750728.1	LOC122578709	protein FREE1-like, transcript variant X1	0.0001	down
XM_043773348.1	LOC122600599	heavy metal-associated isoprenylated plant protein 36-like	0.0028	down
XM_043760674.1	LOC122588540	cytochrome P450 83B1-like	0.0019	down
XM_043752187.1	LOC122579924	ethylene-responsive transcription factor 4-like	< 0.0001	down
XM_043778543.1	LOC122605580	uncharacterized LOC122605580	0.0002	down
XM_043761079.1	LOC122588863	nitrate reductase [NADH]	< 0.0001	down
XM_043750968.1	**LOC122578915**	5'-nucleotidase domain-containing protein 4-like, transcript variant X1	0.0025	down
XM_043750967.1	**LOC122578915**	5'-nucleotidase domain-containing protein 4-like, transcript variant X1	< 0.0001	down
XM_043751331.1	**LOC122579206**	probable serine/threonine-protein kinase PBL28, transcript variant X1	< 0.0001	down
XM_043751325.1	**LOC122579206**	probable serine/threonine-protein kinase PBL28, transcript variant X1	0.0015	down
XM_043752460.1	**LOC122580176**	uncharacterized protein At1g76660-like, transcript variant X1	< 0.0001	down

Transcript id	GeneID	Gene name/function	*P* Value	result
XM_043752452.1	**LOC122580176**	uncharacterized protein At1g76660-like, transcript variant X1	< 0.0001	down
XM_043752896.1	**LOC122580601**	nuclear transcription factor Y subunit B-1-like, transcript variant X6	< 0.0001	down
XM_043752874.1	**LOC122580601**	nuclear transcription factor Y subunit B-1-like, transcript variant X6	0.0015	down
XM_043752921.1	**LOC122580655**	scarecrow-like protein 21, transcript variant X3	0.0019	down
XM_043752922.1	**LOC122580655**	scarecrow-like protein 21, transcript variant X3	< 0.0001	down
XR_006320985.1	**LOC122581230**	micronuclear linker histone polyprotein, transcript variant X3	0.0040	down
XR_006320989.1	**LOC122581230**	micronuclear linker histone polyprotein, transcript variant X3	0.0005	down
XM_043755615.1	**LOC122583188**	synaptotagmin-2-like, transcript variant X1	< 0.0001	down
XM_043755623.1	**LOC122583188**	synaptotagmin-2-like, transcript variant X1	< 0.0001	down
XM_043755627.1	**LOC122583188**	synaptotagmin-2-like, transcript variant X1	< 0.0001	down
XM_043757770.1	**LOC122585644**	uncharacterized LOC122585644, transcript variant X1	< 0.0001	down
XM_043757775.1	**LOC122585644**	uncharacterized LOC122585644, transcript variant X1	< 0.0001	down
XM_043758497.1	**LOC122586514**	NAC domain-containing protein 91-like, transcript variant X1	< 0.0001	down
XM_043758498.1	**LOC122586514**	NAC domain-containing protein 91-like, transcript variant X1	0.0004	down
XM_043758733.1	**LOC122586743**	malate dehydrogenase, chloroplastic, transcript variant X1	0.0003	down
XM_043758732.1	**LOC122586743**	malate dehydrogenase, chloroplastic, transcript variant X1	< 0.0001	down
XM_043758813.1	**LOC122586820**	synaptotagmin-2-like, transcript variant X2	< 0.0001	down
XM_043758814.1	**LOC122586820**	synaptotagmin-2-like, transcript variant X2	0.0020	down
XM_043759171.1	**LOC122587096**	probable WRKY transcription factor 40, transcript variant X2	< 0.0001	down
XM_043759170.1	**LOC122587096**	probable WRKY transcription factor 40, transcript variant X2	< 0.0001	down
XM_043760602.1	**LOC122588476**	GATA transcription factor 8-like, transcript variant X1	< 0.0001	down
XM_043760603.1	**LOC122588476**	GATA transcription factor 8-like, transcript variant X1	< 0.0001	down
XM_043761082.1	**LOC122588866**	thioredoxin H2-like, transcript variant X2	0.0008	down
XM_043761081.1	**LOC122588866**	thioredoxin H2-like, transcript variant X2	< 0.0001	down
XM_043762831.1	**LOC122590652**	calmodulin-binding protein 60 B-like, transcript variant X2	< 0.0001	down
XM_043762830.1	**LOC122590652**	calmodulin-binding protein 60 B-like, transcript variant X2	< 0.0001	down
XM_043762950.1	**LOC122590778**	glycine-rich protein A3-like, transcript variant X2	< 0.0001	down
XM_043762949.1	**LOC122590778**	glycine-rich protein A3-like, transcript variant X2	< 0.0001	down
XM_043763012.1	**LOC122590838**	receptor-like protein EIX2, transcript variant X1	0.0046	down
XM_043763013.1	**LOC122590838**	receptor-like protein EIX2, transcript variant X1	0.0026	down
XM_043763153.1	**LOC122590976**	F-box/FBD/LRR-repeat protein At1g13570-like, transcript variant X3	< 0.0001	down
XM_043763154.1	**LOC122590976**	F-box/FBD/LRR-repeat protein At1g13570-like, transcript variant X3	< 0.0001	down
XM_043763152.1	**LOC122590976**	F-box/FBD/LRR-repeat protein At1g13570-like, transcript variant X3	< 0.0001	down
XM_043763151.1	**LOC122590976**	F-box/FBD/LRR-repeat protein At1g13570-like, transcript variant X3	0.0002	down
XM_043766154.1	**LOC122593706**	metacaspase-1-like, transcript variant X1	< 0.0001	down
XM_043766153.1	**LOC122593706**	metacaspase-1-like, transcript variant X1	< 0.0001	down
XM_043766369.1	**LOC122593911**	common plant regulatory factor 1-like, transcript variant X1	0.0040	down
XM_043766368.1	**LOC122593911**	common plant regulatory factor 1-like, transcript variant X1	< 0.0001	down
XM_043766431.1	**LOC122593957**	probable F-box protein At2g36090, transcript variant X1	0.0007	down
XM_043766433.1	**LOC122593957**	probable F-box protein At2g36090, transcript variant X1	0.0015	down
XM_043766432.1	**LOC122593957**	probable F-box protein At2g36090, transcript variant X1	< 0.0001	down
XM_043768030.1	**LOC122595624**	probable protein phosphatase 2C 15, transcript variant X2	0.0019	down
XM_043768028.1	**LOC122595624**	probable protein phosphatase 2C 15, transcript variant X2	0.0007	down
XM_043768148.1	**LOC122595720**	BES1/BZR1 homolog protein 4-like, transcript variant X2	< 0.0001	down
XM_043768149.1	**LOC122595720**	BES1/BZR1 homolog protein 4-like, transcript variant X2	0.0004	down
XM_043768365.1	**LOC122595892**	heptahelical transmembrane protein 1-like, transcript variant X1	< 0.0001	down
XM_043768364.1	**LOC122595892**	heptahelical transmembrane protein 1-like, transcript variant X1	< 0.0001	down
XM_043769177.1	**LOC122596568**	anaphase-promoting complex subunit 4, transcript variant X1	0.0016	down
XM_043769176.1	**LOC122596568**	anaphase-promoting complex subunit 4, transcript variant X1	0.0005	down
XM_043769416.1	**LOC122596784**	protein LSD1-like, transcript variant X1	0.0001	down
XM_043769418.1	**LOC122596784**	protein LSD1-like, transcript variant X1	< 0.0001	down
XR_006323401.1	**LOC122596995**	cyclin-dependent kinase G-2-like, transcript variant X6	0.0018	down
XM_043769653.1	**LOC122596995**	cyclin-dependent kinase G-2-like, transcript variant X6	< 0.0001	down
XM_043769746.1	**LOC122597113**	phosphoinositide phospholipase C 2-like, transcript variant X1	< 0.0001	down
XM_043769751.1	**LOC122597113**	phosphoinositide phospholipase C 2-like, transcript variant X1	< 0.0001	down
XM_043770819.1	**LOC122598216**	probable serine/threonine-protein kinase PBL26, transcript variant X1	0.0024	down
XM_043770820.1	**LOC122598216**	probable serine/threonine-protein kinase PBL26, transcript variant X1	< 0.0001	down
XR_006323908.1	**LOC122599355**	disease resistance protein RPV1-like, transcript variant X9	0.0021	down
XR_006323912.1	**LOC122599355**	disease resistance protein RPV1-like, transcript variant X9	< 0.0001	down

Transcript id	GeneID	Gene name/function	*P* Value	result
XM_043772810.1	**LOC122600143**	alpha,alpha-trehalose-phosphate synthase [UDP-forming] 1, transcript variant X1	< 0.0001	down
XM_043772811.1	**LOC122600143**	alpha,alpha-trehalose-phosphate synthase [UDP-forming] 1, transcript variant X1	< 0.0001	down
XM_043772947.1	**LOC122600254**	transcription factor GTE3, chloroplastic-like, transcript variant X3	< 0.0001	down
XM_043772944.1	**LOC122600254**	transcription factor GTE3, chloroplastic-like, transcript variant X3	< 0.0001	down
XM_043773427.1	**LOC122600674**	metacaspase-1-like, transcript variant X1	< 0.0001	down
XM_043773426.1	**LOC122600674**	metacaspase-1-like, transcript variant X1	< 0.0001	down
XM_043775118.1	**LOC122602434**	two-component response regulator ARR17, transcript variant X2	< 0.0001	down
XM_043775121.1	**LOC122602434**	two-component response regulator ARR17, transcript variant X2	< 0.0001	down
XM_043775312.1	**LOC122602723**	wall-associated receptor kinase-like 14, transcript variant X1	0.0003	down
XM_043775313.1	**LOC122602723**	wall-associated receptor kinase-like 14, transcript variant X1	0.0003	down
XM_043775798.1	**LOC122603165**	exocyst complex component SEC8, transcript variant X1	< 0.0001	down
XM_043775796.1	**LOC122603165**	exocyst complex component SEC8, transcript variant X1	0.0026	down
XM_043776222.1	**LOC122603503**	uncharacterized LOC122603503, transcript variant X2	0.0005	down
XM_043776221.1	**LOC122603503**	uncharacterized LOC122603503, transcript variant X2	< 0.0001	down
XM_043776481.1	**LOC122603705**	patatin-like protein 6, transcript variant X3	< 0.0001	down
XM_043776480.1	**LOC122603705**	patatin-like protein 6, transcript variant X3	< 0.0001	down
XM_043776700.1	**LOC122603868**	protein REVEILLE 8, transcript variant X3	< 0.0001	down
XM_043776702.1	**LOC122603868**	protein REVEILLE 8, transcript variant X3	0.0007	down
XM_043776701.1	**LOC122603868**	protein REVEILLE 8, transcript variant X3	0.0015	down
XM_043778053.1	**LOC122605161**	thiosulfate sulfurtransferase 18-like, transcript variant X2	< 0.0001	down
XM_043778052.1	**LOC122605161**	thiosulfate sulfurtransferase 18-like, transcript variant X2	0.0030	down
XM_043778508.1	**LOC122605549**	uncharacterized LOC122605549, transcript variant X1	< 0.0001	down
XM_043778507.1	**LOC122605549**	uncharacterized LOC122605549, transcript variant X1	0.0059	down
XM_043778688.1	**LOC122605728**	pleiotropic drug resistance protein 1-like, transcript variant X1	< 0.0001	down
XM_043778687.1	**LOC122605728**	pleiotropic drug resistance protein 1-like, transcript variant X1	0.0020	down
XM_043779878.1	**LOC122606972**	protein MICRORCHIDIA 6-like, transcript variant X3	< 0.0001	down
XM_043779882.1	**LOC122606972**	protein MICRORCHIDIA 6-like, transcript variant X3	0.0019	down
XM_043780046.1	**LOC122607126**	extensin-like, transcript variant X1	0.0001	down
XM_043780045.1	**LOC122607126**	extensin-like, transcript variant X1	< 0.0001	down
XM_043781003.1	**LOC122607929**	receptor-like protein kinase, transcript variant X2	0.0004	down
XM_043781002.1	**LOC122607929**	receptor-like protein kinase, transcript variant X2	< 0.0001	down
XR_006325364.1	**LOC122609669**	probable carboxylesterase 11, transcript variant X2	< 0.0001	down
XM_043782623.1	**LOC122609669**	probable carboxylesterase 11, transcript variant X2	0.0003	down
XM_043782939.1	**LOC122609977**	uncharacterized WD repeat-containing protein C2A9.03, transcript variant X2	< 0.0001	down
XM_043782940.1	**LOC122609977**	uncharacterized WD repeat-containing protein C2A9.03, transcript variant X2	0.0048	down
XR_006325439.1	**LOC122610000**	uncharacterized LOC122610000, transcript variant X1	< 0.0001	down
XR_006325440.1	**LOC122610000**	uncharacterized LOC122610000, transcript variant X1	0.0002	down
XM_043784247.1	**LOC122611240**	protein TIFY 6B-like, transcript variant X5	0.0016	down
XM_043784232.1	**LOC122611240**	protein TIFY 6B-like, transcript variant X5	0.0001	down
XM_043784240.1	**LOC122611240**	protein TIFY 6B-like, transcript variant X5	0.0016	down
XM_043776610.1	**LOC122603802**	protein PSK SIMULATOR 1-like, transcript variant X2	0.0007	up
XM_043776612.1	**LOC122603802**	protein PSK SIMULATOR 1-like, transcript variant X2	0.0052	down
XM_043758915.1	**LOC122586895**	putative GPI-anchored protein pfl2, transcript variant X2	0.0032	up
XM_043758916.1	**LOC122586895**	putative GPI-anchored protein pfl2, transcript variant X2	0.0047	down
XM_043751188.1	**LOC122579090**	protein IQ-DOMAIN 13-like, transcript variant X2	0.0003	up
XM_043751190.1	**LOC122579090**	protein IQ-DOMAIN 13-like, transcript variant X2	0.0001	down
XR_006324593.1	**LOC122605332**	uncharacterized LOC122605332, transcript variant X1	0.0048	up
XR_006324589.1	**LOC122605332**	uncharacterized LOC122605332, transcript variant X1	0.0012	down
XM_043771925.1	**LOC122599417**	FGGY carbohydrate kinase domain-containing protein, transcript variant X2	< 0.0001	up
XM_043771922.1	**LOC122599417**	FGGY carbohydrate kinase domain-containing protein, transcript variant X2	< 0.0001	down
XM_043771077.1	**LOC122598485**	uncharacterized LOC122598485, transcript variant X1	0.0007	up
XM_043771079.1	**LOC122598485**	uncharacterized LOC122598485, transcript variant X1	0.0037	down
XM_043782823.1	**LOC122609878**	probable protein phosphatase 2C 60, transcript variant X1	< 0.0001	up
XM_043782822.1	**LOC122609878**	probable protein phosphatase 2C 60, transcript variant X1	0.0058	down
XM_043783022.1	**LOC122610044**	uncharacterized LOC122610044, transcript variant X1	0.0012	up
XM_043783023.1	**LOC122610044**	uncharacterized LOC122610044, transcript variant X1	< 0.0001	down
XM_043762692.1	**LOC122590488**	ribonuclease 3-like protein 3, transcript variant X1	< 0.0001	up
XM_043762691.1	**LOC122590488**	ribonuclease 3-like protein 3, transcript variant X1	0.0005	down
XM_043776500.1	**LOC122603715**	DNA-binding protein BIN4, transcript variant X1	0.0006	up
XM_043776498.1	**LOC122603715**	DNA-binding protein BIN4, transcript variant X1	< 0.0001	down

Transcript id	GeneID	Gene name/function	*P* Value	result
XM_043767859.1	**LOC122595398**	G-type lectin S-receptor-like serine/threonine-protein kinase At4g27290, transcript variant X2	< 0.0001	up
XM_043767785.1	**LOC122595398**	G-type lectin S-receptor-like serine/threonine-protein kinase At4g27290, transcript variant X2	0.0001	down
XM_043766259.1	**LOC122593806**	metacaspase-1-like, transcript variant X2	< 0.0001	up
XM_043766258.1	**LOC122593806**	metacaspase-1-like, transcript variant X2	< 0.0001	down
XM_043783075.1	**LOC122610086**	paired amphipathic helix protein Sin3-like 3, transcript variant X2	0.0007	up
XM_043783076.1	**LOC122610086**	paired amphipathic helix protein Sin3-like 3, transcript variant X2	0.0022	down
XM_043754171.1	**LOC122581860**	ATP-dependent zinc metalloprotease FTSH 8, mitochondrial-like, transcript variant X3	< 0.0001	up
XM_043754169.1	**LOC122581860**	ATP-dependent zinc metalloprotease FTSH 8, mitochondrial-like, transcript variant X3	< 0.0001	down
XM_043780959.1	**LOC122607889**	serine/threonine protein phosphatase 2A 55 kDa regulatory subunit B beta isoform-like, transcript variant X3	0.0044	up
XM_043780960.1	**LOC122607889**	serine/threonine protein phosphatase 2A 55 kDa regulatory subunit B beta isoform-like, transcript variant X3	0.0007	down

HC represents the high concentration treatment (4 mg/kg Cd and 360 mg/kg Pb). Locus IDs shown in bold indicate that more than one transcript is associated with the same gene.

Table S6 Expression profiles related to transporters in *Erigeron canadensis* roots treated with heavy metals

Transcript ID	Locus ID	MC versus CK		HC versus CK		HC versus MC		Gene description
		log_2[Fold change (treatment/control)]	*q* -value	log_2[Fold change (treatment/control)]	*q* -value	log_2[Fold change (treatment$_{HC}$/treatment$_{MC}$)]	*q* -value	
XM 043776917.1	LOC122604029	2.18	0.0409	3.27	0.1630	-0.49	0.5310	metal transporter Nramp3-like
XM 043750791.1	LOC122578759	-1.27	0.0303	-0.34	0.6510	0.92	0.3850	metal transporter Nramp5-like, transcript variant X1
XM 043780961.1	LOC122607887	1.71	0.0457	1.80	0.0255	0.09	0.6520	zinc transporter 29
XM 043768424.1	LOC122595941	2.22	0.1510	4.84	0.0033	2.62	0.0137	zinc transport protein ZntB-like, transcript variant X2
XM 043773734.1	LOC122600957	-1.75	0.0016	-0.17	0.0830	-2.37	< 0.0001	zinc transporter 1
XM 043768355.1	LOC122595883	-4.86	< 0.0001	-3.27	0.1050	-5.71	< 0.0001	zinc transporter 5-like
XM 043775551.1	LOC122602937	-1.76	0.0258	-1.34	0.1020	-1.16	0.2210	copper transport protein CCH-like
XM 043767376.1	LOC122594706	-1.13	0.2250	-2.62	0.0880	-1.47	0.1170	copper-transporting ATPase HMA4-like
XM 043784236.1	LOC122611248	-6.27	< 0.0001	0.77	0.0285	7.04	< 0.0001	metal-nicotianamine transporter YSL1-like, transcript variant X2
XM 043784302.1	LOC122611298	-4.41	0.0053	-2.83	0.1370	-3.59	0.2970	metal tolerance protein 1-like, transcript variant X1
XM 043773667.1	LOC122600889	2.34	0.0327	2.37	0.2810	-1.56	0.1000	metal tolerance protein 1-like
XM 043758903.1	LOC122586886	-1.91	0.0159	-2.54	0.0002	-0.62	0.3290	calcium-transporting ATPase 2, plasma membrane-type-like
XM_043769052.1	LOC122596473	-3.81	0.0125	-2.38	0.0022	1.43	0.1950	calcium-transporting ATPase 10, plasma membrane-type-like, transcript variant X2
XM 043781215.1	LOC122608122	-3.15	0.0001	-5.03	< 0.0001	-1.88	0.0196	calcium-transporting ATPase 12, plasma membrane-type-like
XM 043755300.1	LOC122582866	0.63	0.1310	3.35	0.0048	2.72	0.0034	calcium-transporting ATPase 12, plasma membrane-type-like
XM 043753094.1	LOC122580945	-0.96	0.4730	-3.59	< 0.0001	-2.63	< 0.0001	putative calcium-transporting ATPase 13, plasma membrane-type
XM 043781398.1	LOC122608307	-4.11	0.0266	-5.25	0.0138	-1.14	0.5240	ABC transporter A family member 2-like, transcript variant X1
XM 043757544.1	LOC122585410	-3.67	0.0152	-2.84	0.0010	0.82	0.3260	ABC transporter G family member 6-like
XM 043766757.1	LOC122594285	-11.17	0.0110	-11.17	0.0091	/	/	ABC transporter B family member 6-like
XM 043755209.1	LOC122582772	0.92	0.1830	2.33	0.0014	1.41	0.1230	ABC transporter B family member 26, chloroplastic
XM 043762063.1	LOC122589737	-1.11	0.0950	-2.71	0.0206	-1.60	0.0510	ABC transporter B family member 19
XM 043753138.1	LOC122580994	-2.86	0.1170	-3.87	0.0004	-1.00	0.6580	ABC transporter B family member 21-like, transcript variant X2
XM 043771317.1	LOC122598832	-1.18	0.3580	-3.96	< 0.0001	-2.78	< 0.0001	putative ABC transporter B family member 8
XM 043753421.1	LOC122581240	-2.10	< 0.0001	-1.92	0.0082	0.18	0.7470	ABC transporter C family member 4-like
XM 043771806.1	LOC122599310	-1.11	0.3320	-1.66	0.0311	-0.55	0.0530	ABC transporter C family member 3-like
XM 043762857.1	LOC122590676	-11.45	< 0.0001	-3.81	0.4070	/	/	ABC transporter C family member 3-like, transcript variant X7
XM 043758977.1	LOC122586944	1.12	0.1360	1.90	0.0428	0.78	0.1620	ABC transporter C family member 8-like
XM 043779517.1	LOC122606676	0.31	0.5060	2.40	0.1880	0.50	0.3090	ABC transporter C family member 13, transcript variant X1
XM 043770004.1	LOC122597404	-1.49	0.0327	-0.61	0.2330	0.89	0.3380	ABC transporter E family member 2-like
XR 006323437.1	LOC122597473	-11.84	< 0.0001	-5.21	0.3740	/	/	ABC transporter F family member 4-like, transcript variant X7
XM 043760963.1	LOC122588769	0.57	0.0540	2.18	< 0.0001	1.62	< 0.0001	ABC transporter F family member 5-like, transcript variant X2
XM 043752390.1	LOC122580118	-0.15	0.8110	-1.44	0.0040	-1.28	0.0372	ABC transporter G family member 14-like
XM 043757146.1	LOC122585048	-0.08	0.9160	-1.97	0.0003	-1.89	0.0146	ABC transporter G family member 14-like
XM 043767720.1	LOC122595368	0.97	0.0860	2.35	0.0122	1.38	0.1240	ABC transporter G family member 7, transcript variant X2
XM 043761157.1	LOC122588937	-0.95	0.1740	-1.63	0.0194	-0.67	0.5170	ABC transporter G family member 25
XM 043755674.1	LOC122583250	-5.12	0.0125	-3.03	0.3500	2.09	0.2590	ABC transporter G family member 29-like
XM 043753221.1	LOC122581077	-4.11	0.0266	-0.78	0.1990	2.03	0.1270	ABC transporter G family member 32-like, transcript variant X1
XM 043760607.1	LOC122588480	-1.75	0.0003	-1.40	0.0022	0.35	0.0140	amino acid transporter AVT6A-like
XM 043783377.1	LOC122610382	1.98	0.0183	2.31	0.0050	0.33	0.1920	amino acid transporter AVT6A-like
XM 043760142.1	LOC122587996	-2.10	0.0029	-2.27	0.0014	-0.17	0.6210	amino acid transporter AVT6C-like
XM 043762338.1	LOC122590002	3.26	0.0478	3.37	0.0336	0.11	0.8980	amino acid transporter AVT1I-like
XM 043779589.1	LOC122606731	3.72	0.0017	3.72	< 0.0001	0.00	0.9960	amino acid transporter AVT1A-like
XM 043750676.1	LOC122578662	2.13	0.0266	3.35	0.1040	-0.36	0.4990	amino acid transporter AVT1I-like, transcript variant X1
XM 043770981.1	LOC122598386	-1.91	0.0422	-3.69	0.0013	-1.77	0.1760	lysine histidine transporter-like 8
XM 043753312.1	LOC122581145	-2.76	< 0.0001	-2.53	0.0127	0.23	0.7160	lysine histidine transporter-like 8

XM 043771056.1	LOC122598460	-2.28	0.0272	-1.60	0.0127	0.67	0.3690	lysine histidine transporter-like 8
XM 043770644.1	LOC122598040	-2.26	< 0.0001	-1.56	0.0007	0.70	0.3260	lysine histidine transporter-like 6
XM 043759054.1	LOC122586998	-2.90	0.0010	0.91	0.0590	2.23	0.0530	lysine histidine transporter 1-like
XM 043770293.1	LOC122597728	1.97	0.0239	2.51	0.0002	0.54	0.0970	sugar transport protein 14
XM 043753290.1	LOC122581134	4.38	0.0087	4.23	0.0166	-0.15	0.7850	sugar transporter ERD6-like 7, transcript variant X2
XM 043782628.1	LOC122609676	2.60	< 0.0001	3.21	0.0530	-0.97	0.0520	sugar transporter ERD6-like 16
XM 043752083.1	LOC122579836	1.72	0.0091	3.44	0.1040	0.13	0.8160	sugar transporter ERD6-like 5, transcript variant X1
XM 043780285.1	LOC122607330	-3.72	0.0276	-4.77	0.0069	-1.05	0.3210	bidirectional sugar transporter SWEET9-like
XM 043754600.1	LOC122582231	-1.71	0.3500	-3.21	0.0368	-1.50	0.0700	bidirectional sugar transporter SWEET10-like
XM 043760790.1	LOC122588634	-3.07	0.1560	-5.12	0.0010	-2.05	0.3120	bidirectional sugar transporter N3-like
XM 043767305.1	LOC122595026	-6.19	< 0.0001	-4.24	0.0024	1.95	0.0690	vacuolar iron transporter homolog 4-like
XM 043767306.1	LOC122595027	-4.82	< 0.0001	-2.45	0.0379	2.37	0.0028	vacuolar iron transporter homolog 4-like
XM 043766967.1	LOC122594521	-5.24	0.0002	-4.18	0.0026	2.86	0.0001	vacuolar iron transporter homolog 4-like
XM 043767304.1	LOC122595024	-4.74	0.0019	-4.19	0.0019	0.56	0.5720	vacuolar iron transporter homolog 4-like
XM 043767301.1	LOC122595021	-2.92	0.0069	-0.06	0.9430	0.21	0.0540	vacuolar iron transporter homolog 4-like
XM 043768903.1	LOC122596340	-1.38	0.0009	-1.17	0.0710	1.14	0.6030	vacuolar iron transporter 1
XM 043773062.1	LOC122600353	-4.08	< 0.0001	-2.94	0.0074	0.08	0.3670	phospholipid-transporting ATPase 3-like, transcript variant X1
XM 043773947.1	LOC122601180	-1.99	0.0013	-0.33	0.0530	0.08	0.8880	putative phospholipid-transporting ATPase 9
XM 043770911.1	LOC122598318	-1.79	0.1940	-2.22	0.0110	-0.43	0.8660	putative phospholipid-transporting ATPase 9
XM 043763541.1	LOC122591300	-0.96	0.0570	-1.61	< 0.0001	-0.65	0.2950	plasma membrane ATPase 4
XM 043779499.1	LOC122606658	0.42	0.1400	1.83	0.0489	1.42	0.0079	plasma membrane ATPase 1-like, transcript variant X2
XM 043771846.1	LOC122599343	0.95	0.0170	2.03	0.0454	1.08	0.1590	putative inactive cadmium/zinc-transporting ATPase HMA3
XM 043753163.1	LOC122581022	2.77	0.0424	3.03	0.0840	-1.32	0.3730	auxin transporter-like protein 2
XM 043758165.1	LOC122586054	-0.73	0.3870	-1.47	0.0474	-0.74	0.1330	auxin transporter-like protein 2
XM 043768448.1	LOC122595962	1.52	0.0195	1.13	0.0820	-0.39	0.3170	IAA-alanine resistance protein 1
XM 043771780.1	LOC122599290	4.32	0.0430	3.59	0.0325	2.08	0.0170	cyclic nucleotide-gated ion channel 2
XM 043772185.1	LOC122599643	1.76	0.6680	0.28	0.0560	1.48	0.0076	probable cyclic nucleotide-gated ion channel 14

CK, MC, and HC represent the control (no Cd or Pb addition), moderate concentration (2 mg/kg Cd and 180 mg/kg Pb), and high concentration (4 mg/kg Cd and 360 mg/kg Pb) treatments, respectively. Fold change was calculated using the mean of three biological replicates. The symbol "/" indicates that the denominator is 0 or the numerator is 0, in which case the calculation of the log function cannot be performed.

Table S7 Expression profiles of signaling genes in *Erigeron canadensis* roots treated with heavy metals

Transcript id	Locus ID	MC versus CK		HC versus CK		HC versus MC		Gene description
		log_2[Fold change (treatment/control)] [a]	q-value	log_2[Fold change (treatment/control)] [a]	q-value	log_2[Fold change ($treatment_{HC}$/$treatment_{MC}$)] [a]	q-value	
XM_043755346.1	LOC122582916	-4.17	0.0006	-5.85	< 0.0001	-1.68	0.3490	serine/threonine-protein kinase PCRK1-like
XM_043758823.1	LOC122586828	-7.34	< 0.0001	-2.81	0.0010	4.53	< 0.0001	serine/threonine-protein kinase AFC2, transcript variant X1
XM_043780871.1	LOC122607819	-2.31	0.0285	-2.09	0.0272	0.22	0.5210	serine/threonine-protein kinase-like protein CCR4
XM_043778816.1	LOC122605859	-2.59	0.0004	-2.32	< 0.0001	0.26	0.7420	serine/threonine-protein kinase UCN-like
XM_043770961.1	LOC122598368	-2.11	0.0393	-2.54	0.0004	-0.43	0.6590	probable serine/threonine-protein kinase PBL1
XM_043778609.1	LOC122605651	2.91	0.0001	4.70	< 0.0001	1.79	< 0.0001	probable serine/threonine-protein kinase PBL3
XM_043757371.1	LOC122585251	1.74	0.1120	2.80	0.0335	1.05	0.0520	probable serine/threonine-protein kinase PBL3
XM_043755455.1	LOC122583017	-2.17	0.0029	-2.01	0.0051	0.16	0.5670	probable serine/threonine-protein kinase PBL7
XM_043753184.1	LOC122581046	2.38	0.0122	1.81	0.0740	-0.57	0.4500	probable serine/threonine-protein kinase PBL9, transcript variant X1
XM_043781008.1	LOC122607933	1.95	0.0640	2.35	0.0032	0.40	0.3330	probable serine/threonine-protein kinase PBL10, transcript variant X4
XM_043781010.1	LOC122607933	-14.39	< 0.0001	2.09	0.6640	14.89	0.0038	probable serine/threonine-protein kinase PBL10, transcript variant X4
XM_043767339.1	LOC122595054	-14.03	0.0086	-1.28	0.4140	/	/	probable serine/threonine-protein kinase PBL19, transcript variant X1
XM_043770819.1	LOC122598216	-2.99	0.0067	-1.73	0.0239	1.26	0.0930	probable serine/threonine-protein kinase PBL26, transcript variant X1
XM_043770820.1	LOC122598216	-0.81	0.4110	-12.58	0.0003	/	/	probable serine/threonine-protein kinase PBL26, transcript variant X1
XM_043751331.1	LOC122579206	-17.63	< 0.0001	-17.63	< 0.0001	/	/	probable serine/threonine-protein kinase PBL28, transcript variant X1
XM_043751325.1	LOC122579206	-1.89	0.0006	-1.34	0.0167	0.54	0.4030	probable serine/threonine-protein kinase PBL28, transcript variant X1
XM_043771382.1	LOC122598902	-4.56	0.1330	-11.94	0.0449	/	/	probable serine/threonine-protein kinase PBL28
XM_043767803.1	LOC122595443	0.00	0.6710	17.18	< 0.0001	17.18	< 0.0001	serine/threonine-protein kinase PBL34-like, transcript variant X2
XM_043763109.1	LOC122590934	0.33	0.3450	-2.25	0.0002	-2.58	0.0004	serine/threonine-protein kinase PBL35-like
XM_043775912.1	LOC122603252	-2.80	< 0.0001	-1.02	0.0068	1.78	0.0002	probable serine/threonine-protein kinase PIX13
XM_043779572.1	LOC122606714	-2.64	0.0006	-1.32	0.0492	1.31	0.0034	probable serine/threonine-protein kinase PIX13, transcript variant X1
XM_043760243.1	LOC122588103	-2.06	0.0107	-6.36	< 0.0001	-4.30	0.1060	serine/threonine-protein kinase STY46-like
XM_043760680.1	LOC122588547	-4.18	0.0212	-4.34	0.0108	-0.16	0.8890	serine/threonine-protein kinase STY13-like
XM_043759624.1	LOC122587450	-1.01	< 0.0001	-0.33	0.0580	0.68	0.0560	probable serine/threonine-protein kinase DDB_G0281745
XM_043779695.1	LOC122606825	-0.55	0.6240	-11.52	0.0003	/	/	probable serine/threonine-protein kinase DDB_G0271402, transcript variant X6
XM_043771518.1	LOC122599068	-14.89	0.0015	-1.41	0.4100	/	/	probable serine/threonine-protein kinase At1g54610, transcript variant X1
XM_043774848.1	LOC122602121	-0.46	0.1840	-4.19	0.0075	-3.73	0.0540	serine/threonine-protein kinase-like protein ACR4
XM_043766805.1	LOC122594326	-0.95	0.0590	-1.41	< 0.0001	-0.46	0.1960	serine/threonine-protein kinase-like protein ACR4
XM_043765561.1	LOC122593189	0.18	0.8530	-2.35	0.0051	-2.52	0.0081	serine/threonine-protein kinase ATG1c-like, transcript variant X2
XM_043752754.1	LOC122580480	-0.90	0.3120	-1.77	0.0254	-0.87	0.0510	probable serine/threonine-protein kinase At1g54610
XM_043753205.1	LOC122581064	0.42	0.8380	-13.17	0.0011	/	/	probable serine/threonine-protein kinase WNK4, transcript variant X1
XM_043776877.1	LOC122603995	-1.83	0.2030	-4.17	< 0.0001	-2.34	0.2560	serine/threonine-protein kinase PCRK1-like
XM_043756767.1	LOC122584718	-1.38	0.0830	-1.36	0.0235	0.02	0.9560	probable serine/threonine-protein kinase PIX13
XM_043750052.1	LOC122578154	-0.49	0.5240	-1.57	< 0.0001	-1.09	0.3800	probable serine/threonine-protein kinase PIX13, transcript variant X1
XM_043776424.1	LOC122603657	-2.59	0.0620	-1.49	0.0048	1.10	0.1610	probable serine/threonine-protein kinase PIX13, transcript variant X1
XM_043776423.1	LOC122603657	-1.48	< 0.0001	0.83	0.0530	0.72	0.0510	probable serine/threonine-protein kinase PIX13, transcript variant X1
XM_043771354.1	LOC122598859	0.15	0.8920	-11.58	0.0001	/	/	serine/threonine-protein kinase prpf4B-like, transcript variant X8
XM_043771347.1	LOC122598859	-1.21	0.0183	1.74	0.7280	1.36	0.0003	serine/threonine-protein kinase prpf4B-like, transcript variant X8
XM_043780313.1	LOC122607356	-0.05	0.9100	-1.04	0.0096	-1.00	0.0590	serine/threonine-protein kinase CTR1
XM_043763504.1	LOC122591266	-1.89	< 0.0001	-1.48	< 0.0001	0.41	0.3880	serine/threonine-protein kinase RIPK-like
XM_043775914.1	LOC122603253	-14.22	0.0014	-14.23	0.0012	/	/	probable serine/threonine-protein kinase CST, transcript variant X2
XM_043776142.1	LOC122603443	-1.13	0.0004	-1.30	0.0004	-0.18	0.6020	serine/threonine-protein kinase/endoribonuclease IRE1b-like
XM_043772261.1	LOC122599710	2.94	< 0.0001	3.90	0.0001	0.95	0.2700	serine/threonine-protein kinase D6PKL2
XM_043782701.1	LOC122609759	2.48	0.0025	3.72	0.0610	-0.34	0.4370	serine/threonine-protein kinase SAPK3-like
XM_043784322.1	LOC122611313	1.16	0.0580	2.13	0.0005	0.97	0.0530	serine/threonine-protein kinase SAPK2
XM_043758258.1	LOC122586152	1.23	0.0610	1.79	0.0020	0.55	0.0520	serine/threonine-protein kinase SRPK-like, transcript variant X1
XM_043780302.1	LOC122607346	1.72	0.0253	2.39	0.0610	-0.92	0.0550	serine/threonine-protein kinase 16-like

XM_043754581.1	LOC122582220	2.59	0.0018	3.32	0.1620	-0.96	0.0720	serine/threonine-protein kinase 19, transcript variant X3
XM_043765060.1	LOC122592762	2.07	0.0680	3.00	0.0007	0.93	0.1090	serine/threonine-protein kinase 24-like
XM_043780537.1	LOC122607544	2.19	0.0258	3.56	0.1210	-0.21	0.0740	serine/threonine-protein kinase BSK5-like, transcript variant X1
XM_043761583.1	LOC122589308	1.73	0.0040	2.51	0.0530	-0.81	0.0550	serine-threonine kinase receptor-associated protein-like
XM_043778155.1	LOC122605245	2.26	0.0550	3.47	0.0075	1.21	0.0530	probable inactive serine/threonine-protein kinase bub1
XM_043750064.1	LOC122578164	0.89	0.0530	1.68	0.0115	0.80	0.0620	serine/threonine-protein kinase BLUS1-like, transcript variant X3
XM_043754233.1	LOC122581915	0.88	0.1360	2.18	< 0.0001	1.30	0.0248	serine/threonine-protein kinase rio2
XM_043775711.1	LOC122603097	0.58	0.3970	1.78	0.0370	1.20	0.1030	probable serine/threonine-protein kinase SIS8, transcript variant X1
XM_043754663.1	LOC122582292	-3.95	0.0277	-2.49	0.0298	1.46	0.0510	LRR receptor-like serine/threonine-protein kinase ERL1
XM_043751308.1	LOC122579193	-1.75	< 0.0001	-1.77	< 0.0001	-0.02	0.8590	inactive LRR receptor-like serine/threonine-protein kinase BIR2
XM_043760878.1	LOC122588708	-1.81	0.0001	-0.85	0.1330	0.96	0.2340	LRR receptor-like serine/threonine-protein kinase ERL1
XM_043756331.1	LOC122583962	-13.20	0.0087	-13.20	0.0082	/	/	probable LRR receptor-like serine/threonine-protein kinase RFK1, transcript variant X2
XM_043756332.1	LOC122583962	-11.55	0.0149	-3.32	0.1440	/	/	probable LRR receptor-like serine/threonine-protein kinase RFK1, transcript variant X2
XM_043759243.1	LOC122587149	-4.88	0.0024	-5.88	0.5450	/	/	probable LRR receptor-like serine/threonine-protein kinase At3g47570
XM_043775154.1	LOC122602542	-16.38	< 0.0001	-0.01	0.1820	/	/	probable LRR receptor-like serine/threonine-protein kinase At5g63710, transcript variant X1
XM_043756145.1	LOC122583773	-1.38	0.0148	2.13	0.0570	1.92	0.0267	probable LRR receptor-like serine/threonine-protein kinase At3g47570
XM_043758107.1	LOC122585991	-1.44	0.0283	2.06	0.5330	1.92	0.0471	probable LRR receptor-like serine/threonine-protein kinase At1g56130
XM_043778000.1	LOC122605119	-3.59	0.0024	-0.46	0.2090	1.54	0.5020	LRR receptor-like serine/threonine-protein kinase FLS2
XM_043755879.1	LOC122583480	11.27	0.0102	2.81	0.0570	-1.82	0.0600	LRR receptor-like serine/threonine-protein kinase RGI1
XM_043762990.1	LOC122590821	-5.08	0.1250	-15.84	0.0006	/	/	LRR receptor-like serine/threonine-protein kinase EFR
XM_043772339.1	LOC122599771	1.40	0.3330	-14.65	0.0018	-16.06	< 0.0001	LRR receptor-like serine/threonine-protein kinase FEI 2, transcript variant X2
XM_043752482.1	LOC122580202	-1.42	0.2100	-1.61	0.0347	-0.19	0.6780	probable LRR receptor-like serine/threonine-protein kinase RPK1
XM_043770698.1	LOC122598091	-2.07	0.2350	-2.92	0.0010	-1.21	0.0580	probable LRR receptor-like serine/threonine-protein kinase At1g67720
XM_043782560.1	LOC122609524	-1.82	0.0590	-3.32	< 0.0001	-1.49	< 0.0001	probable LRR receptor-like serine/threonine-protein kinase IRK
XM_043770424.1	LOC122597849	0.98	0.1980	-5.67	0.0029	-6.65	0.0002	probable LRR receptor-like serine/threonine-protein kinase At1g14390, transcript variant X4
XM_043762920.1	LOC122590744	-0.34	0.5990	-1.71	0.0026	-1.37	0.0366	leucine-rich repeat receptor-like serine/threonine-protein kinase BAM3
XM_043773826.1	LOC122601049	-1.16	0.0540	-3.13	< 0.0001	-1.96	< 0.0001	putative leucine-rich repeat receptor-like serine/threonine-protein kinase At2g24130
XM_043778589.1	LOC122605632	-1.23	0.1950	-1.26	0.0371	-0.02	0.9430	leucine-rich repeat receptor-like serine/threonine-protein kinase BAM1
XM_043761748.1	LOC122589453	1.77	0.0510	2.10	0.0148	0.33	0.4640	LRR receptor-like serine/threonine-protein kinase FEI 2
XM_043763344.1	LOC122591129	4.24	0.3340	6.63	0.0361	2.39	0.1990	probable LRR receptor-like serine/threonine-protein kinase RFK1, transcript variant X1
XM_043772036.1	LOC122599508	2.92	0.0530	3.85	0.0298	0.93	0.3230	LRR receptor-like serine/threonine-protein kinase GSO2
XM_043758531.1	LOC122586542	0.00	0.9990	3.09	0.0201	3.09	0.0090	probable LRR receptor-like serine/threonine-protein kinase At3g47570
XM_043754185.1	LOC122581870	2.97	0.0840	2.91	0.0002	-0.07	0.9180	leucine-rich repeat receptor-like serine/threonine-protein kinase SKM1
XM_043755438.1	LOC122583001	-2.93	< 0.0001	-2.26	< 0.0001	0.66	0.2890	CBL-interacting serine/threonine-protein kinase 7-like
XM_043753608.1	LOC122581384	-1.45	< 0.0001	-1.63	< 0.0001	-0.18	0.4200	CBL-interacting serine/threonine-protein kinase 11
XM_043771674.1	LOC122599210	-1.37	0.0102	0.04	0.8330	1.41	0.0700	CBL-interacting serine/threonine-protein kinase 6
XM_043763195.1	LOC122591011	1.89	0.0008	1.23	0.0600	-0.65	0.0840	CBL-interacting serine/threonine-protein kinase 23
XM_043754864.1	LOC122582470	-2.16	0.0008	-2.15	< 0.0001	0.01	0.9890	CBL-interacting protein kinase 2-like
XM_043753207.1	LOC122581066	-1.21	0.0229	0.30	0.6610	1.51	0.1670	CBL-interacting protein kinase 18-like, transcript variant X1
XM_043774799.1	LOC122602068	-1.75	0.0011	-1.01	0.0112	0.74	0.1250	G-type lectin S-receptor-like serine/threonine-protein kinase At1g34300
XM_043764155.1	LOC122591928	-1.87	< 0.0001	0.77	0.0540	1.06	0.0510	G-type lectin S-receptor-like serine/threonine-protein kinase LECRK1
XM_043777679.1	LOC122604812	-15.85	< 0.0001	-0.69	0.3930	13.57	0.0148	G-type lectin S-receptor-like serine/threonine-protein kinase SD2-5, transcript variant X2
XM_043764519.1	LOC122592316	-8.34	0.0030	-3.52	0.0610	3.24	0.0720	G-type lectin S-receptor-like serine/threonine-protein kinase SD1-1, transcript variant X1
XM_043764520.1	LOC122592316	-4.27	0.0407	-1.61	0.0710	1.07	0.6610	G-type lectin S-receptor-like serine/threonine-protein kinase SD1-1, transcript variant X1

XM_043767785.1	LOC122595398	-1.64	0.2620	-14.14	0.0023	-12.49	0.0005	G-type lectin S-receptor-like serine/threonine-protein kinase At4g27290, transcript variant X2
XM_043767859.1	LOC122595398	-0.62	0.6240	5.03	< 0.0001	5.66	0.0024	G-type lectin S-receptor-like serine/threonine-protein kinase At4g27290, transcript variant X2
XM_043764511.1	LOC122592308	2.02	0.0012	3.12	< 0.0001	1.10	0.0347	G-type lectin S-receptor-like serine/threonine-protein kinase At4g27290
XM_043779465.1	LOC122606626	0.74	0.4270	2.67	0.0198	1.92	0.0192	G-type lectin S-receptor-like serine/threonine-protein kinase At1g67520
XM_043772034.1	LOC122599505	1.81	0.4320	3.44	0.0075	1.64	0.1300	G-type lectin S-receptor-like serine/threonine-protein kinase At4g27290
XM_043771997.1	LOC122599477	0.37	0.7730	4.98	0.0036	4.60	< 0.0001	G-type lectin S-receptor-like serine/threonine-protein kinase At4g27290
XM_043769544.1	LOC122596900	-0.25	0.6880	2.65	0.0183	2.90	< 0.0001	G-type lectin S-receptor-like serine/threonine-protein kinase At4g27290
XM_043772220.1	LOC122599675	-0.02	0.9680	1.83	0.0263	1.85	0.0037	G-type lectin S-receptor-like serine/threonine-protein kinase At1g67520
XM_043781050.1	LOC122607966	-1.06	0.5800	1.68	0.0269	2.74	0.0008	G-type lectin S-receptor-like serine/threonine-protein kinase SD1-1, transcript variant X1
XM_043766140.1	LOC122593695	2.18	0.0610	3.22	0.0352	1.04	0.0520	G-type lectin S-receptor-like serine/threonine-protein kinase At1g34300
XM_043784374.1	LOC122611354	3.51	0.0203	4.68	0.0540	-0.42	0.3550	CDPK-related protein kinase-like
XM_043766748.1	LOC122594275	1.01	0.0520	2.33	0.0043	1.32	0.0228	leucine-rich repeat receptor protein kinase HPCA1-like
XM_043768615.1	LOC122596097	-11.36	0.0069	-0.55	0.0660	/	/	probable leucine-rich repeat receptor-like protein kinase At1g68400
XM_043783462.1	LOC122610474	-1.67	0.1280	-1.26	0.0482	0.41	0.4660	putative leucine-rich repeat receptor-like protein kinase At2g19210
XM_043753192.1	LOC122581054	-1.35	0.0510	-1.38	0.0116	-0.04	0.9540	leucine-rich repeat receptor-like protein kinase PXC1
XM_043773258.1	LOC122600530	-4.85	0.0117	-2.55	0.0256	2.30	0.0740	L-type lectin-domain containing receptor kinase IX.1-like
XM_043767650.1	LOC122595305	-3.15	0.0195	-3.36	0.0309	-0.21	0.8140	L-type lectin-domain containing receptor kinase VIII.2-like
XM_043777556.1	LOC122604686	-1.35	0.2930	-2.24	0.0272	-0.89	0.4460	L-type lectin-domain containing receptor kinase IX.1-like
XM_043773180.1	LOC122600463	-2.84	0.0326	-0.26	0.7560	2.59	0.0382	L-type lectin-domain containing receptor kinase IX.1-like
XM_043773668.1	LOC122600890	-1.56	0.0022	0.20	0.1630	1.77	0.0096	L-type lectin-domain containing receptor kinase IX.1-like
XM_043751635.1	LOC122579451	-1.27	< 0.0001	0.59	0.0570	0.27	0.2510	probable L-type lectin-domain containing receptor kinase S.5
XM_043779202.1	LOC122606241	2.28	0.0067	2.66	0.0510	-1.21	0.0710	L-type lectin-domain containing receptor kinase IX.1-like
XM_043770719.1	LOC122598114	-2.17	0.0031	-2.18	0.0001	-0.01	0.9910	calcium-dependent protein kinase 26-like
XM_043777868.1	LOC122605006	-1.62	0.0027	-1.29	0.0053	0.33	0.4020	calcium-dependent protein kinase 14
XM_043755128.1	LOC122582702	1.52	0.0044	2.19	0.0021	0.68	0.2180	calcium-dependent protein kinase SK5-like
XM_043775665.1	LOC122603052	-1.77	0.0259	0.38	0.3150	0.57	0.0970	calcium-dependent protein kinase 18-like, transcript variant X2
XM_043765743.1	LOC122593351	-2.40	< 0.0001	-1.59	< 0.0001	0.80	0.1220	calcium-dependent protein kinase 19-like
XM_043769857.1	LOC122597239	-1.02	0.0048	-1.25	< 0.0001	-0.23	0.5090	calmodulin-binding receptor-like cytoplasmic kinase 2
XM_043771686.1	LOC122599220	1.63	0.0468	1.03	0.0560	-0.60	0.2060	calmodulin-binding receptor-like cytoplasmic kinase 3, transcript variant X1
XM_043758570.1	LOC122586585	-3.17	0.0003	-3.05	< 0.0001	0.12	0.7690	mitogen-activated protein kinase kinase kinase 20-like
XM_043778711.1	LOC122605756	-2.77	0.0345	-3.96	0.0014	-1.20	0.2390	mitogen-activated protein kinase kinase kinase 20-like
XM_043779661.1	LOC122606806	-1.49	< 0.0001	0.00	0.0530	0.78	0.0520	mitogen-activated protein kinase homolog NTF6-like
XM_043763025.1	LOC122590849	-2.51	< 0.0001	-4.18	< 0.0001	-1.67	0.0510	mitogen-activated protein kinase kinase kinase 18-like
XM_043763020.1	LOC122590844	-2.74	0.0540	-3.80	< 0.0001	-1.06	0.0650	mitogen-activated protein kinase kinase kinase 18-like
XM_043756644.1	LOC122584545	-1.08	< 0.0001	-1.46	< 0.0001	-0.38	0.0820	mitogen-activated protein kinase 9-like
XM_043766162.1	LOC122593715	-1.48	0.1240	-2.23	0.0023	-0.75	0.3530	mitogen-activated protein kinase kinase kinase 18-like
XM_043767638.1	LOC122595296	-1.16	0.0820	-1.26	< 0.0001	-0.10	0.7290	mitogen-activated protein kinase kinase 5
XM_043766437.1	LOC122593961	-1.63	0.1700	-2.25	< 0.0001	-0.62	0.3110	mitogen-activated protein kinase kinase kinase 18-like
XM_043761875.1	LOC122589572	-3.15	0.0028	-2.86	0.0036	0.29	0.6150	mitogen-activated protein kinase kinase kinase 20-like
XM_043763752.1	LOC122591485	-3.80	0.1190	-4.54	0.0076	-0.74	0.6870	wall-associated receptor kinase 2-like
XM_043763768.1	LOC122591500	0.45	0.8010	-14.12	< 0.0001	/	/	wall-associated receptor kinase 2-like
XM_043763933.1	LOC122591681	1.76	0.0960	3.00	0.0070	1.24	0.1830	wall-associated receptor kinase 2-like
XM_043775146.1	LOC122602540	-0.93	0.3410	-12.87	0.0002	/	/	wall-associated receptor kinase-like 2
XM_043763775.1	LOC122591506	-1.18	0.3560	-4.08	< 0.0001	-2.90	0.3930	wall-associated receptor kinase 2-like
XM_043777177.1	LOC122604290	-1.62	0.0195	-1.39	0.1110	0.22	0.7480	wall-associated receptor kinase 5-like
XM_043782418.1	LOC122609369	-5.25	0.0297	-2.08	0.3090	1.58	0.5610	wall-associated receptor kinase-like 9
XM_043775312.1	LOC122602723	-2.29	0.1620	-2.74	0.0050	-0.45	0.6140	wall-associated receptor kinase-like 14, transcript variant X1
XM_043775313.1	LOC122602723	-2.63	0.1510	-2.81	0.0041	-0.18	0.5210	wall-associated receptor kinase-like 14, transcript variant X1
XM_043752257.1	LOC122579990	0.33	0.7040	-1.74	0.0174	-2.07	0.0092	wall-associated receptor kinase-like 15
XM_043775221.1	LOC122602626	-5.11	0.1730	-12.49	0.0440	/	/	putative wall-associated receptor kinase-like 16

XM_043778915.1	LOC122605947	-1.12	0.0283	1.18	0.4140	0.71	0.1050	wall-associated receptor kinase-like 20

CK, MC, and HC represent the control (no Cd or Pb addition), moderate concentration (2 mg/kg Cd and 180 mg/kg Pb), and high concentration (4 mg/kg Cd and 360 mg/kg Pb) treatments, respectively. Fold change was calculated using the mean of three biological replicates. The symbol "/" indicates that the denominator is 0 or the numerator is 0, in which case the calculation of the log function cannot be performed.

Table S8 Expression profiles of ROS genes in *Erigeron canadensis* roots treated with heavy metals

Transcript id	Locus ID	MC versus CK		HC versus CK		HC versus MC		Gene description
		log_2[Fold change (treatment/control)] [a]	*q* -value	log_2[Fold change (treatment/control)] [a]	*q* -value	log_2[Fold change ($treatment_{HC}$/$treatment_{MC}$)] [a]	*q* -value	
XM_043754988.1	LOC122582577	1.86	0.0126	2.94	0.0530	-0.50	0.1970	2-alkenal reductase (NADP(+)-dependent)-like
XM_043753541.1	LOC122581328	1.74	0.0830	3.58	< 0.0001	1.85	0.0067	2-alkenal reductase (NADP(+)-dependent)-like
XM_043782951.1	LOC122609986	-12.16	0.0442	-12.16	0.0405	/	/	2-alkenal reductase (NADP(+)-dependent)-like
XM_043773515.1	LOC122600758	2.09	0.0011	2.01	< 0.0001	-0.08	0.8130	4-hydroxy-3-methylbut-2-enyl diphosphate reductase, chloroplastic-like
XM_043778019.1	LOC122605139	1.80	0.0318	1.94	0.0309	0.14	0.5450	monodehydroascorbate reductase 4, peroxisomal
XM_043767853.1	LOC122595487	3.30	0.0326	3.34	0.0546	0.03	0.8400	tropinone reductase homolog At5g06060-like
XM_043759153.1	LOC122587078	-0.73	0.5800	-2.35	0.0263	-1.62	0.0537	tropinone reductase homolog At5g06060-like
XM_043776617.1	LOC122603804	1.87	0.0181	3.13	0.0790	-0.32	0.3560	5'-adenylylsulfate reductase-like 5
XM_043753557.1	LOC122581340	1.70	0.0049	3.03	< 0.0001	-0.25	0.0660	glyoxylate/succinic semialdehyde reductase 1
XM_043779901.1	LOC122606987	1.97	0.0467	1.40	0.1030	-0.57	0.1560	tropinone reductase homolog At2g29260, chloroplastic-like, transcript variant X2
XM_043760683.1	LOC122588549	1.44	0.0131	1.10	0.1360	-0.34	0.4600	pyrroline-5-carboxylate reductase
XM_043768166.1	LOC122595738	1.82	0.0001	1.03	< 0.0001	-0.79	0.0510	very-long-chain 3-oxoacyl-CoA reductase 1-like
XM_043754577.1	LOC122582216	1.72	0.0323	1.54	0.0060	-0.18	0.3760	NADPH-dependent pterin aldehyde reductase, transcript variant X1
XM_043765442.1	LOC122593091	2.69	0.0230	2.39	0.1430	-0.30	0.6810	glyoxylate/hydroxypyruvate reductase HPR3-like
XM_043752001.1	LOC122579772	1.68	0.2460	4.44	0.0032	2.76	0.0560	tetraketide alpha-pyrone reductase 1, transcript variant X2
XM_043752854.1	LOC122580586	3.87	0.1560	7.01	< 0.0001	3.14	0.0800	ferredoxin--NADP reductase, leaf-type isozyme, chloroplastic
XM_043756310.1	LOC122583934	1.13	0.0580	1.57	0.0014	0.44	0.0690	delta(14)-sterol reductase
XM_043752401.1	LOC122580131	0.72	0.3200	2.47	0.0051	1.74	0.0201	3-hydroxy-3-methylglutaryl coenzyme A reductase 2-B-like
XM_043768521.1	LOC122596024	0.88	0.0570	1.96	0.0161	1.08	0.0520	glyoxylate/hydroxypyruvate/pyruvate reductase 2KGR-like
XM_043780546.1	LOC122607552	2.32	0.4770	6.61	0.0137	4.29	0.0550	dehydrogenase/reductase SDR family member 12, transcript variant X1
XM_043770566.1	LOC122597971	6.92	0.1200	14.73	< 0.0001	1.16	0.0820	enoyl-[acyl-carrier-protein] reductase [NADH] 1, chloroplastic-like
XM_043784046.1	LOC122611084	0.96	0.3040	3.27	0.0288	2.31	0.0079	glutamyl-tRNA reductase 1, chloroplastic-like
XM_043765021.1	LOC122592726	0.92	0.0520	1.87	0.0243	0.94	0.1030	enoyl-[acyl-carrier-protein] reductase, mitochondrial
XM_043784091.1	LOC122611122	2.39	0.2190	5.49	< 0.0001	3.10	0.0011	protochlorophyllide reductase, chloroplastic-like
XM_043751220.1	LOC122579113	1.57	0.0780	2.26	0.0230	0.68	0.2720	polyprenol reductase 2-like
XM_043752285.1	LOC122580017	0.10	0.8970	-1.54	0.0390	-1.64	0.2270	putative 12-oxophytodienoate reductase 11, transcript variant X2
XM_043781837.1	LOC122608744	-3.53	0.1510	-14.29	0.0007	/	/	short-chain dehydrogenase reductase 2a
XM_043753243.1	LOC122581094	0.43	0.6060	-3.35	< 0.0001	-3.78	0.0002	3-dehydrosphinganine reductase TSC10A-like
XM_043764914.1	LOC122592632	-3.44	0.0990	-6.41	< 0.0001	-2.97	0.0136	trifunctional UDP-glucose 4,6-dehydratase/UDP-4-keto-6-deoxy-D-glucose 3,5-epimerase/UDP-4-keto-L-rhamnose-reductase RHM1-like
XM_043761079.1	LOC122588863	-0.02	0.9520	-2.88	< 0.0001	-2.86	< 0.0001	nitrate reductase [NADH]
XM_043768831.1	LOC122596282	5.89	0.3440	6.73	0.0134	0.84	0.5630	ferredoxin-thioredoxin reductase, variable chain-like
XM_043774887.1	LOC122602162	-1.40	0.0088	-1.06	0.0128	0.34	0.3710	3-hydroxy-3-methylglutaryl coenzyme A reductase 1-like
XM_043765796.1	LOC122593394	-4.51	< 0.0001	-3.44	< 0.0001	1.07	0.0570	3-oxoacyl-[acyl-carrier-protein] reductase FabG-like
XM_043774658.1	LOC122601928	-1.40	0.0020	-1.40	0.0002	0.00	0.9920	glutamyl-tRNA reductase 2-like
XM_043766494.1	LOC122594018	-3.41	0.0031	-3.10	0.0032	0.31	0.7000	short-chain dehydrogenase reductase 3b-like
XM_043768124.1	LOC122595699	-1.96	0.0300	0.03	0.3330	0.41	0.3460	artemisinic aldehyde Delta(11(13)) reductase
XM_043753573.1	LOC122581354	-2.01	0.0021	-0.78	0.2910	1.23	0.0030	cinnamoyl-CoA reductase 1
XM_043767829.1	LOC122595464	-1.36	0.0414	-1.03	0.2880	0.33	0.1070	7-dehydrocholesterol reductase
XM_043768997.1	LOC122596422	12.81	0.0030	12.28	0.0179	-0.54	0.2090	2-hydroxy-palmitic acid dioxygenase MPO1-like, transcript variant X2
XM_043767378.1	LOC122595081	3.19	0.0258	3.07	0.0528	-0.12	0.8120	2-oxoglutarate-dependent dioxygenase 19-like
XM_043767377.1	LOC122595080	2.70	0.0570	3.13	< 0.0001	0.43	0.3610	2-oxoglutarate-dependent dioxygenase 19-like
XM_043774939.1	LOC122602226	-1.61	0.0205	1.46	0.8640	1.49	0.2520	carotenoid cleavage dioxygenase 7, chloroplastic
XM_043779421.1	LOC122606581	-6.27	0.0119	-14.13	0.0018	/	/	hyoscyamine 6-dioxygenase-like
XM_043779334.1	LOC122606485	-0.61	0.1770	-1.61	0.0262	-1.00	0.1120	hyoscyamine 6-dioxygenase-like
XM_043771908.1	LOC122599400	-3.22	< 0.0001	-3.94	< 0.0001	-0.72	0.0570	probable carotenoid cleavage dioxygenase 4, chloroplastic
XM_043769233.1	LOC122596621	-3.89	< 0.0001	-6.22	0.0011	-2.33	0.0560	probable 2-oxoglutarate-dependent dioxygenase AOP1

XM_043784048.1	LOC122611086	-2.80	0.0351	-2.25	0.0406	0.56	0.5280	probable 2-oxoglutarate-dependent dioxygenase AOP1
XM_043762570.1	LOC122590240	-2.27	0.0530	-12.61	0.0022	/	/	probable 2-oxoglutarate-dependent dioxygenase AOP1
XM_043769376.1	LOC122596747	-12.94	0.0047	-12.94	0.0036	/	/	probable inactive 2-oxoglutarate-dependent dioxygenase AOP2
XM_043756299.1	LOC122583924	-3.35	0.0253	-4.18	< 0.0001	-0.83	0.6360	carotenoid cleavage dioxygenase 8 homolog B, chloroplastic-like
XM_043765544.1	LOC122593174	-1.66	0.0244	-1.94	0.0377	-0.28	0.6740	9-cis-epoxycarotenoid dioxygenase NCED1, chloroplastic-like
XM_043763425.1	LOC122591201	-1.41	0.1970	-2.06	0.0048	-0.65	0.0610	gibberellin 2-beta-dioxygenase 1-like
XM_043756800.1	LOC122584747	-1.05	0.4220	-2.10	0.0086	-1.05	0.0276	extradiol ring-cleavage dioxygenase
XM_043757948.1	LOC122585824	0.35	0.7250	-2.98	0.0286	-3.33	0.0014	gibberellin 2-beta-dioxygenase 8-like
XM_043772206.1	LOC122599661	0.53	0.1490	1.42	0.0497	0.90	0.0560	homogentisate 1,2-dioxygenase
XM_043784131.1	LOC122611155	4.66	0.1110	11.69	0.0345	0.38	0.5800	carotenoid 9,10(9',10')-cleavage dioxygenase 1-like
XM_043778867.1	LOC122605908	1.05	0.3200	1.92	0.0494	0.87	0.1940	2-oxoglutarate-dependent dioxygenase DAO-like
XM_043756116.1	LOC122583740	-1.00	0.3670	-2.28	0.0019	-1.28	0.1220	gibberellin 3-beta-dioxygenase 1-like
XM_043776121.1	LOC122603424	1.51	0.0448	3.02	0.0510	-0.07	0.6600	thiol-disulfide oxidoreductase LTO1
XM_043756605.1	LOC122584442	5.81	0.0237	2.03	0.3550	-5.37	0.0143	NADH-ubiquinone oxidoreductase chain 3
XM_043762229.1	LOC122589900	2.47	0.0154	2.10	0.0610	-0.36	0.3260	NADPH:quinone oxidoreductase-like
XM_043755225.1	LOC122582787	1.75	0.0018	2.55	0.0534	0.80	0.0540	electron transfer flavoprotein-ubiquinone oxidoreductase, mitochondrial
XM_043763244.1	LOC122591054	-2.39	0.4270	5.42	0.0025	7.81	< 0.0001	2-oxoglutarate-Fe(II) type oxidoreductase hxnY-like, transcript variant X2
XM_043777604.1	LOC122604735	3.27	0.2330	12.50	0.0354	2.58	0.1260	probable mitochondrial saccharopine dehydrogenase-like oxidoreductase At5g39410
XM_043752361.1	LOC122580090	5.49	0.0351	5.65	0.0129	0.16	0.8400	thioredoxin-like 1-2, chloroplastic
XM_043751982.1	LOC122579753	-2.55	0.0040	-2.83	< 0.0001	-0.27	0.7080	thioredoxin H-type-like
XM_043753099.1	LOC122580951	-1.43	0.0240	-1.89	0.0111	-0.47	0.2790	thioredoxin H-type-like
XM_043761081.1	LOC122588866	-3.85	< 0.0001	-3.94	0.0005	-0.09	0.9280	thioredoxin H2-like, transcript variant X2
XM_043761082.1	LOC122588866	-4.62	0.0090	-6.33	0.0095	-1.72	0.4290	thioredoxin H2-like, transcript variant X2
XM_043767493.1	LOC122595167	-4.45	0.0290	-6.99	< 0.0001	-2.55	0.4600	thioredoxin H2-like, transcript variant X1
XM_043772413.1	LOC122599826	0.13	0.8990	-3.04	0.0015	-3.17	0.0048	thioredoxin 1-like
XM_043751205.1	LOC122579105	1.76	0.0334	2.47	0.0003	0.70	0.1610	protein overexpressor of cationic peroxidase 3, transcript variant X2
XM_043752653.1	LOC122580383	3.48	0.0031	3.35	< 0.0001	-0.12	0.8750	peroxidase 10-like
XM_043754739.1	LOC122582361	3.46	0.0168	1.74	0.0840	-1.71	< 0.0001	cationic peroxidase 1-like
XM_043757643.1	LOC122585517	-1.07	0.2690	-2.12	0.0053	-1.05	0.0600	cationic peroxidase 1-like
XM_043757555.1	LOC122585422	-0.39	0.6570	-1.85	0.0189	-1.46	0.0493	cationic peroxidase 1-like
XM_043761820.1	LOC122589521	-5.49	0.0069	-1.01	0.0590	2.89	0.1920	peroxidase 9-like
XM_043752352.1	LOC122580082	-5.14	< 0.0001	-1.15	0.0730	2.41	0.1940	peroxidase 9-like
XM_043768000.1	LOC122595601	-5.04	< 0.0001	-4.88	< 0.0001	0.16	0.8770	peroxidase 60-like
XM_043770383.1	LOC122597808	-2.55	< 0.0001	-4.68	< 0.0001	-2.13	0.0001	peroxidase N1-like
XM_043770305.1	LOC122597737	-2.20	0.0006	-4.35	< 0.0001	-2.15	0.0012	peroxidase N1-like
XM_043770036.1	LOC122597443	-0.98	0.0810	-4.06	0.0474	-3.08	0.0120	peroxidase N1-like
XM_043777458.1	LOC122604575	-1.74	0.0040	-0.86	0.0920	0.88	0.1220	peroxidase 17
XM_043770500.1	LOC122597911	-0.78	0.2310	-2.99	0.0002	-2.21	0.0202	lignin-forming anionic peroxidase-like
XM_043752054.1	LOC122579819	-0.20	0.8000	-2.57	0.0107	-2.37	0.0082	peroxidase 12-like
XM_043775158.1	LOC122602556	-0.33	0.5910	-5.12	< 0.0001	-4.80	< 0.0001	peroxidase N-like
XM_043751000.1	LOC122578941	-0.55	0.3780	-2.13	0.0002	-1.57	0.0007	peroxidase 63-like
XM_043772234.1	LOC122599686	1.81	0.0016	2.70	0.0530	-0.69	0.0610	glutathione peroxidase 1
XM_043773124.1	LOC122600412	1.92	0.0444	2.91	0.2680	-0.59	0.2950	probable phospholipid hydroperoxide glutathione peroxidase
XM_043784106.1	LOC122611133	-0.88	0.1130	-1.94	< 0.0001	-1.06	0.0220	probable phospholipid hydroperoxide glutathione peroxidase
XM_043770718.1	LOC122598117	1.61	0.0372	2.55	< 0.0001	0.94	0.0570	glutathione hydrolase 3-like
XM_043764659.1	LOC122592435	3.08	0.0031	2.29	0.0039	-0.79	0.3670	glutathione S-transferase U19-like
XM_043776450.1	LOC122603675	-0.49	0.4300	-2.32	< 0.0001	-1.83	0.0150	glutathione S-transferase DHAR2-like
XM_043783796.1	LOC122610840	-1.81	0.3120	-16.34	< 0.0001	-14.54	0.0201	probable glutathione S-transferase
XM_043774866.1	LOC122602139	-0.38	0.7640	-4.90	0.0019	-4.52	0.0004	probable glutathione S-transferase
XM_043754436.1	LOC122582083	3.43	0.0042	3.59	0.0790	-1.43	0.0164	probable glutathione S-transferase
XM_043765536.1	LOC122593165	-1.32	0.1280	-1.76	< 0.0001	-0.44	0.5690	glutathione S-transferase APIC-like
XM_043753070.1	LOC122580927	-0.49	0.3830	-1.61	0.0032	-1.12	0.2670	glutathione S-transferase APIC-like
XM_043762657.1	LOC122590455	3.71	0.0002	0.58	0.3680	-3.13	< 0.0001	glutathione S-transferase U8-like

XM 043773753.1	LOC122600972	3.14	0.0002	2.03	0.0580	-1.11	0.0106	probable glutathione S-transferase parC
XM 043780037.1	LOC122607110	-2.75	0.0395	-2.55	0.0330	0.20	0.8330	catalase, transcript variant X1
XM 043763233.1	LOC122591043	1.36	0.1750	2.21	< 0.0001	0.85	0.1020	ferritin, chloroplastic-like
XM 043752981.1	LOC122580820	-2.80	0.0004	1.34	0.7040	2.55	< 0.0001	ferritin-3, chloroplastic-like
XM 043779495.1	LOC122606653	-0.76	0.0710	-2.04	0.0130	-1.28	0.0550	caffeic acid 3-O-methyltransferase-like
XM 043779008.1	LOC122606044	-0.44	0.0900	-1.09	< 0.0001	-0.65	0.0530	caffeoyl-CoA O-methyltransferase-like
XM 043767446.1	LOC122595132	-1.64	0.0560	-1.60	0.0330	0.04	0.0960	caffeoyl-CoA O-methyltransferase-like
XM 043768517.1	LOC122596020	-3.15	< 0.0001	-2.43	< 0.0001	0.72	0.3480	caffeoyl-CoA O-methyltransferase 1

CK, MC, and HC represent the control (no Cd or Pb addition), moderate concentration (2 mg/kg Cd and 180 mg/kg Pb), and high concentration (4 mg/kg Cd and 360 mg/kg Pb) treatments, respectively. Fold change was calculated using the mean of three biological replicates. The symbol "/" indicates that the denominator is 0 or the numerator is 0, in which case the calculation of the log function cannot be performed.

Table S9 Expression profiles of transcription factors genes in *Erigeron canadensis* roots treated with heavy metals

Transcript id	Locus ID	MC versus CK		HC versus CK		HC versus MC		Gene description
		$\log_2$[Fold change (treatment/control)] [a]	*q* -value	$\log_2$[Fold change (treatment/control)] [a]	*q* -value	$\log_2$[Fold change (treatment$_{HC}$/treatment$_{MC}$)] [a]	*q* -value	
XM 043752202.1	LOC122579935	-1.75	0.0098	-3.44	< 0.0001	-3.33	< 0.0001	FCS-Like Zinc finger 2-like
XM 043765551.1	LOC122593182	3.36	< 0.0001	4.10	0.0004	0.74	0.3590	FCS-Like Zinc finger 8-like
XM 043773257.1	LOC122600529	-2.23	< 0.0001	-1.55	0.0291	0.68	0.4070	FCS-Like Zinc finger 1-like
XR 006324821.1	LOC122606139	-12.89	0.0002	0.93	0.5430	/	/	B-box zinc finger protein 19-like, transcript variant X3
XM 043769283.1	LOC122596664	-1.68	0.0010	-2.20	< 0.0001	-0.52	0.3270	B-box zinc finger protein 22-like
XM 043754048.1	LOC122581772	-2.14	< 0.0001	-1.94	< 0.0001	-1.44	0.0206	B-box zinc finger protein 24-like
XM 043766638.1	LOC122594167	2.62	0.0219	4.21	0.1090	0.01	0.9920	B-box zinc finger protein 20-like
XM 043757222.1	LOC122585123	-2.06	< 0.0001	-2.06	< 0.0001	0.00	0.9960	dof zinc finger protein DOF2.1
XM 043766024.1	LOC122593594	-2.17	0.0088	-2.26	0.0013	-0.09	0.8370	dof zinc finger protein DOF3.2-like
XM 043781262.1	LOC122608172	-1.89	0.0418	-3.15	0.0057	-1.26	0.1680	dof zinc finger protein DOF3.1-like
XM 043761576.1	LOC122589299	-0.93	0.2980	-2.17	0.0007	-1.23	0.1050	dof zinc finger protein DOF1.7-like
XM 043766016.1	LOC122593587	-0.73	0.0590	-1.77	< 0.0001	-1.90	0.0126	dof zinc finger protein DOF1.4-like
XM 043765655.1	LOC122593267	-0.26	0.0510	-2.19	< 0.0001	-1.93	0.0760	dof zinc finger protein DOF2.4-like
XM 043780888.1	LOC122607833	1.31	0.6190	-2.37	< 0.0001	-2.74	0.0290	dof zinc finger protein DOF2.4-like
XM 043784213.1	LOC122611226	-1.66	0.1570	-1.85	0.0244	-0.19	0.4610	dof zinc finger protein DOF2.5-like, transcript variant X2
XM 043751954.1	LOC122579729	0.76	0.4110	-5.79	< 0.0001	-6.70	0.0003	dof zinc finger protein DOF5.6
XM 043762332.1	LOC122589999	-3.41	0.0134	-3.78	0.0032	-0.37	0.2630	RING-H2 finger protein ATL8-like
XM 043778375.1	LOC122605424	2.19	0.0062	3.59	0.0800	-0.19	0.7300	RING-H2 finger protein ATL8-like
XM 043781587.1	LOC122608483	-1.75	0.0447	0.23	0.1950	0.39	0.5990	RING-H2 finger protein ATL3-like, transcript variant X2
XM 043761609.1	LOC122589324	0.60	0.2010	-2.18	< 0.0001	-1.20	0.0650	RING-H2 finger protein ATL74-like
XM 043764624.1	LOC122592406	-0.60	0.1730	-1.17	< 0.0001	-0.57	0.2120	RING-H2 finger protein ATL74-like
XM 043778648.1	LOC122605693	0.74	0.2130	3.22	0.0509	2.48	0.0326	RING-H2 finger protein ATL46-like
XR 006324602.1	LOC122605362	-3.27	0.0196	-3.06	0.0870	-1.41	0.5050	zinc finger CCCH domain-containing protein 41, transcript variant X3
XM 043776822.1	LOC122603954	-1.25	0.0242	0.71	0.2380	0.38	0.1550	zinc finger CCCH domain-containing protein 29-like
XM 043759164.1	LOC122587089	-1.82	0.1950	-2.11	0.0300	-0.29	0.3620	zinc finger CCCH domain-containing protein 29-like
XM 043769042.1	LOC122596462	-2.59	< 0.0001	-3.38	< 0.0001	-4.08	0.0006	zinc finger protein CONSTANS-LIKE 5-like
XM 043765007.1	LOC122592717	-1.70	0.0019	-2.65	< 0.0001	-4.75	0.0002	zinc finger protein CONSTANS-LIKE 5-like
XM 043765514.1	LOC122593149	15.49	< 0.0001	0.00	0.6790	/	/	zinc finger protein CONSTANS-LIKE 9-like, transcript variant X1
XM 043765513.1	LOC122593149	2.60	0.0003	2.85	< 0.0001	0.25	0.6020	zinc finger protein CONSTANS-LIKE 9-like, transcript variant X1
XM 043780362.1	LOC122607396	2.05	0.0317	2.50	0.0099	0.46	0.2900	zinc finger protein CONSTANS-LIKE 13
XM 043771328.1	LOC122598843	-1.58	0.0091	-1.71	0.0030	-1.18	0.0321	NF-X1-type zinc finger protein NFXL1, transcript variant X2
XM 043774918.1	LOC122602205	-1.35	0.0078	2.71	0.0530	2.47	0.0630	GATA zinc finger domain-containing protein 4-like
XM 043750696.1	LOC122578677	-4.41	0.0471	2.96	0.2160	5.79	0.0640	zinc finger CCCH domain-containing protein 18-like, transcript variant X1
XM 043756616.1	LOC122584454	-3.03	0.0040	-3.43	0.0007	-0.40	0.6380	zinc finger protein CONSTANS-like
XM 043760553.1	LOC122588438	2.77	0.0050	2.94	< 0.0001	0.17	0.7170	zinc finger CCCH domain-containing protein 11-like
XM 043778742.1	LOC122605787	1.65	0.0243	1.17	0.0580	-0.48	0.0610	zinc finger CCCH domain-containing protein 3
XM 043766853.1	LOC122594388	3.21	0.0228	2.75	0.1430	-0.46	0.6160	zinc finger SWIM domain-containing protein 7
XM_043765007.1	LOC122606810	-1.47	0.0002	-0.78	0.0550	-0.95	0.0600	cysteine-tryptophan domain-containing zinc finger protein 5-like, transcript variant X1
XM_043779669.1	LOC122606810	-12.65	0.0082	-0.34	0.7230	5.25	0.0273	cysteine-tryptophan domain-containing zinc finger protein 5-like, transcript variant X1
XM 043763250.1	LOC122591057	-2.48	0.0007	-1.53	0.0153	0.95	0.1140	zinc finger CCCH domain-containing protein 20
XM 043775787.1	LOC122603158	-4.34	0.0177	-1.04	0.4620	3.30	0.3440	zinc finger protein CONSTANS-LIKE 2
XM 043776226.1	LOC122603492	-13.16	< 0.0001	0.07	0.8010	/	/	zinc finger protein CONSTANS-LIKE 15-like, transcript variant X1
XM 043778071.1	LOC122605176	-1.63	< 0.0001	-2.50	< 0.0001	-0.87	0.0510	zinc finger A20 and AN1 domain-containing stress-associated protein 6-like
XM 043770315.1	LOC122597746	-2.13	0.0044	-2.83	< 0.0001	-0.70	0.0580	zinc finger A20 and AN1 domain-containing stress-associated protein 5-like
XM 043772909.1	LOC122600223	-1.52	0.2020	-1.94	0.0367	-0.43	0.1610	zinc finger A20 and AN1 domain-containing stress-associated protein 5-like
XM 043754039.1	LOC122581764	-1.34	0.2250	-1.58	0.0327	-0.24	0.6260	zinc finger A20 and AN1 domain-containing stress-associated protein 5
XM 043754386.1	LOC122582034	-1.44	0.0048	-0.09	0.7600	1.34	0.0590	ranBP2-type zinc finger protein At1g67325, transcript variant X3

XM_043752543.1	LOC122580265	-1.11	0.0410	-0.38	0.5240	0.72	0.1010	zinc finger CCCH domain-containing protein 44-like
XM_043772422.1	LOC122599835	-2.89	0.0048	-1.28	0.2630	1.62	0.0540	zinc finger CCCH domain-containing protein 18
XM_043753134.1	LOC122580990	-2.07	0.0011	-3.04	0.0290	-0.97	0.1860	zinc finger protein 10
XM_043751929.1	LOC122579706	0.07	0.0550	-1.45	< 0.0001	0.06	0.8480	zinc finger CCCH domain-containing protein 34-like
XM_043765701.1	LOC122593308	-1.50	0.0028	-1.55	0.0008	-0.06	0.8680	zinc finger protein ZAT10-like
XM_043761559.1	LOC122589285	-1.54	0.1750	-2.00	0.0048	1.50	0.0201	zinc finger protein ZAT10-like
XM_043773012.1	LOC122600306	-1.84	0.1790	-2.01	0.0219	1.60	0.0006	zinc finger protein ZAT10-like
XM_043757278.1	LOC122585171	-0.21	0.0570	-1.57	0.0095	-1.36	0.1230	zinc finger protein ZAT11-like
XM_043783874.1	LOC122610920	-2.19	0.0540	-5.51	0.0089	-3.32	0.1700	zinc finger protein ZAT11-like
XM_043766557.1	LOC122594081	0.12	0.9020	-1.13	0.0250	-1.25	0.3040	zinc finger protein ZAT11-like
XM_043761696.1	LOC122589403	1.65	0.0590	3.09	0.0035	1.44	0.0760	zinc finger protein ZAT9-like
XM_043780361.1	LOC122607395	-1.40	0.2060	-1.99	0.0082	-0.59	0.0640	zinc finger protein ZAT10
XM_043751675.1	LOC122579496	1.56	0.0520	2.74	0.0009	1.18	0.0530	zinc finger protein VAR3, chloroplastic-like
XM_043775134.1	LOC122602529	-1.46	0.1740	-1.80	0.0217	-0.34	0.2840	zinc finger protein BALDIBIS-like
XM_043752567.1	LOC122580290	4.39	0.1380	7.12	< 0.0001	2.73	0.0630	zinc finger protein CONSTANS-LIKE 6
XM_043762588.1	LOC122590257	-2.39	0.1720	-4.19	0.0062	-1.80	0.3870	mini zinc finger protein 3-like
XM_043773608.1	LOC122600833	0.79	0.1070	2.13	0.0015	1.34	0.0790	zinc finger CCCH domain-containing protein 22
XM_043751247.1	LOC122579137	0.38	0.8550	-12.67	0.0353	/	/	zinc finger CCCH domain-containing protein 38-like, transcript variant X1
XM_043763199.1	LOC122591014	0.54	0.5150	-1.99	0.0471	-1.44	0.0710	zinc finger BED domain-containing protein DAYSLEEPER-like, transcript variant X1
XM_043760992.1	LOC122588792	-1.22	0.2920	-1.64	0.0253	-0.42	0.6810	zinc finger protein BRUTUS-like At1g74770, transcript variant X2
XM_043761359.1	LOC122589116	1.57	0.0760	1.71	0.0430	0.14	0.7040	zinc finger CCCH domain-containing protein 15 homolog
XM_043761432.1	LOC122589182	-1.15	0.2300	-1.56	0.0434	-0.41	0.5230	zinc finger AN1 domain-containing stress-associated protein 12
XM_043753233.1	LOC122581087	0.65	0.5330	1.68	0.0454	1.03	0.1660	zinc finger CCCH domain-containing protein 53, transcript variant X2
XM_043774775.1	LOC122602043	3.76	0.1100	2.94	0.0130	0.77	0.1880	zinc finger protein CONSTANS-LIKE 9-like, transcript variant X1
XM_043760835.1	LOC122588671	-13.99	0.0067	0.01	0.1760	/	/	AP2-like ethylene-responsive transcription factor ANT, transcript variant X3
XM_043778498.1	LOC122605542	4.47	0.1070	4.23	0.0479	1.35	0.3030	AP2-like ethylene-responsive transcription factor AIL7
XM_043768579.1	LOC122596066	-13.81	0.0132	-13.81	0.0126	/	/	AP2/ERF and B3 domain-containing transcription factor At1g50680-like, transcript variant X1
XM_043769740.1	LOC122597104	-1.52	0.0280	-1.27	0.0368	0.26	0.1910	AP2/ERF domain-containing protein PFD0985w-like
XM_043776781.1	LOC122603925	-3.66	0.0002	0.85	0.0660	2.92	0.1050	ethylene-responsive transcription factor RAP2-7-like
XM_043756802.1	LOC122584749	-4.13	0.0009	-5.26	< 0.0001	-1.13	0.0580	ethylene-responsive transcription factor TINY-like
XM_043784095.1	LOC122611124	2.16	0.0155	3.58	0.0660	-0.16	0.7000	ethylene-responsive transcription factor RAP2-3, transcript variant X1
XM_043784094.1	LOC122611124	3.99	0.0267	3.23	0.0546	-0.76	0.6080	ethylene-responsive transcription factor RAP2-3, transcript variant X1
XM_043779026.1	LOC122606063	-2.62	0.0077	0.51	0.0780	1.55	0.0950	ethylene-responsive transcription factor 1B-like
XM_043778935.1	LOC122605971	-4.04	< 0.0001	-5.01	< 0.0001	-1.95	0.0066	ethylene-responsive transcription factor ERF027-like
XM_043752625.1	LOC122580354	-3.26	< 0.0001	-5.42	< 0.0001	-1.31	0.0378	ethylene-responsive transcription factor ERF027-like
XM_043776759.1	LOC122603906	2.47	0.0128	3.79	< 0.0001	1.88	0.0273	ethylene-responsive transcription factor ERF104-like
XM_043769282.1	LOC122596663	-1.46	0.0382	-1.92	0.0020	-0.46	0.1600	ethylene-responsive transcription factor 3-like
XM_043780919.1	LOC122607853	-2.07	0.0044	-2.11	0.0022	-0.04	0.9310	ethylene-responsive transcription factor CRF1-like
XM_043760074.1	LOC122587917	1.36	0.7720	-2.49	0.0452	-4.12	0.0012	ethylene-responsive transcription factor CRF1-like
XM_043762587.1	LOC122590256	-1.79	0.0093	-4.62	0.0032	-5.17	0.0035	ethylene-responsive transcription factor ERF019
XM_043765192.1	LOC122592873	-2.04	0.0001	-1.70	0.0010	0.35	0.4970	ethylene-responsive transcription factor 4-like
XM_043752187.1	LOC122579924	1.47	0.7830	-1.18	< 0.0001	-1.07	0.0510	ethylene-responsive transcription factor 4-like
XM_043776110.1	LOC122603417	2.72	< 0.0001	3.76	< 0.0001	1.05	0.0590	ethylene-responsive transcription factor 5-like
XM_043758511.1	LOC122586524	-1.19	0.0276	0.85	0.1070	0.46	0.5100	ethylene-responsive transcription factor 2-like
XM_043771469.1	LOC122599022	-4.83	< 0.0001	-5.91	< 0.0001	-1.08	0.1140	ethylene-responsive transcription factor ERF017-like
XM_043764258.1	LOC122592046	-4.05	0.0025	-3.96	0.0022	0.08	0.9040	ethylene-responsive transcription factor ERF017-like
XM_043774710.1	LOC122601978	-3.30	0.0006	-3.41	< 0.0001	-0.12	0.8220	ethylene-responsive transcription factor ERF109-like
XM_043783811.1	LOC122610852	-2.11	< 0.0001	-2.47	< 0.0001	-0.37	0.2630	ethylene-responsive transcription factor CRF2-like
XM_043778046.1	LOC122605156	-4.83	< 0.0001	-5.74	< 0.0001	-1.49	0.0002	ethylene-responsive transcription factor ERF023-like
XM_043778203.1	LOC122605294	-1.62	0.0001	-2.59	< 0.0001	-1.15	0.0066	ethylene-responsive transcription factor CRF4-like
XM_043764259.1	LOC122592047	-3.95	0.0013	-1.64	0.1320	2.30	0.4720	ethylene-responsive transcription factor ERF018-like
XM_043770901.1	LOC122598309	-2.91	0.0001	-3.83	< 0.0001	-0.93	0.2140	ethylene-responsive transcription factor ERF109
XM_043757294.1	LOC122585185	-5.83	< 0.0001	-8.25	< 0.0001	-2.42	0.1440	ethylene-responsive transcription factor ERF025-like

XM 043784094.1	LOC122594455	-0.57	0.2600	-2.00	< 0.0001	-0.76	0.6080	ethylene-responsive transcription factor 12-like
XM 043770540.1	LOC122597946	-2.79	< 0.0001	-2.69	< 0.0001	0.09	0.7690	ethylene-responsive transcription factor 12-like
XM 043773452.1	LOC122600698	-1.74	< 0.0001	-1.58	< 0.0001	0.16	0.5980	ethylene-responsive transcription factor RAP2-3-like
XM 043772479.1	LOC122599877	-0.84	0.0630	-1.21	< 0.0001	-0.37	0.4020	ethylene-responsive transcription factor 1A-like
XM 043784376.1	LOC122611356	-1.21	0.2410	-1.50	0.0434	-0.30	0.2030	ethylene-responsive transcription factor RAP2-4-like
XM 043766942.1	LOC122594493	3.07	0.0540	5.54	< 0.0001	2.46	0.0910	ethylene-responsive transcription factor 13-like
XM 043760042.1	LOC122587882	-0.63	0.2980	-1.53	0.0038	-0.89	0.2370	ethylene-responsive transcription factor ERF014-like
XM 043757474.1	LOC122585345	-3.20	0.0155	-3.53	0.0197	-0.33	0.7540	ethylene-responsive transcription factor ERF061-like
XM 043773557.1	LOC122600794	-1.98	0.0261	0.05	0.0730	0.45	0.6280	transcription factor MYB17-like
XM 043766196.1	LOC122593751	-2.17	< 0.0001	-0.58	0.0770	-1.00	0.3120	transcription factor MYB17-like
XM 043777786.1	LOC122604927	2.77	0.0288	3.42	0.0013	1.93	0.0036	transcription factor MYB73-like
XM 043776783.1	LOC122603926	-1.38	< 0.0001	0.21	0.0590	-0.57	0.1350	transcription factor MYB73-like
XM 043770410.1	LOC122597839	-1.36	0.0004	-1.61	< 0.0001	-0.24	0.5130	transcription factor MYB1R1
XM 043773383.1	LOC122600636	2.23	< 0.0001	2.06	< 0.0001	-0.18	0.1880	transcription factor MYB14-like
XM 043758000.1	LOC122585885	2.74	0.0001	2.79	< 0.0001	0.04	0.8820	transcription factor MYB62-like
XM 043750439.1	LOC122578468	1.92	0.0113	1.92	0.0620	1.07	0.0540	transcription factor MYB62-like
XM 043750490.1	LOC122578515	-1.77	0.0183	-3.26	< 0.0001	-3.91	0.0028	transcription factor MYB10-like
XM 043775867.1	LOC122603219	-1.49	0.0179	-0.63	0.1630	0.86	0.0880	myb-related protein 308-like, transcript variant X1
XM 043764882.1	LOC122592607	-2.85	< 0.0001	-1.79	0.0710	1.06	0.4050	transcription factor MYB102
XM 043773423.1	LOC122600670	-1.63	0.0307	-1.86	< 0.0001	-0.23	0.7390	myb family transcription factor EFM
XM 043753046.1	LOC122580901	-13.10	0.0169	-13.10	0.0144	/	/	myb family transcription factor PHL7-like
XM 043772094.1	LOC122599572	-1.24	0.0002	0.34	0.0630	-0.58	0.4460	myb family transcription factor APL
XM 043761379.1	LOC122589137	-2.51	0.0126	-0.93	0.1440	-0.69	0.4710	transcription factor MYB25-like
XM 043769977.1	LOC122597377	5.25	0.0205	6.83	< 0.0001	1.57	0.0540	MYB-like transcription factor EOBII
XM 043772722.1	LOC122600066	-2.01	0.0223	-0.43	0.1500	-1.05	0.0810	MYB-like transcription factor ODO1
XM 043756641.1	LOC122584497	2.00	0.0365	2.91	0.0560	-0.68	0.0260	myb-like protein P
XM 043771630.1	LOC122599172	1.73	0.0082	1.73	0.0890	1.49	0.0290	myb-like protein X
XM 043765728.1	LOC122593333	-1.11	0.0050	-1.14	0.0003	-0.04	0.8290	WRKY transcription factor WRKY51-like
XM 043770459.1	LOC122597881	-2.61	< 0.0001	-2.53	< 0.0001	-2.89	0.0015	WRKY transcription factor WRKY51-like
XM 043771305.1	LOC122598818	-2.72	0.0002	-1.61	0.0001	1.11	0.0540	probable WRKY transcription factor 53
XM 043755042.1	LOC122582625	-1.41	0.0422	0.93	0.0590	0.75	0.2900	probable WRKY transcription factor 26
XM 043765690.1	LOC122593298	-2.59	< 0.0001	-3.25	< 0.0001	-2.62	0.0011	WRKY transcription factor 22-like
XM 043757564.1	LOC122585431	-2.80	0.0004	-3.60	0.0002	-3.74	0.0384	WRKY transcription factor 22-like
XM 043770321.1	LOC122597752	-1.37	0.0313	-1.68	0.0231	-2.27	0.0333	WRKY transcription factor 22-like
XM 043752016.1	LOC122579781	-2.07	< 0.0001	-1.54	< 0.0001	0.53	0.0560	probable WRKY transcription factor 27
XM 043778920.1	LOC122605955	-12.05	0.0193	-12.05	0.0156	/	/	probable WRKY transcription factor 27
XM 043781775.1	LOC122608684	-2.14	0.0430	-1.29	0.2860	0.85	0.4210	probable WRKY transcription factor 70
XM 043758312.1	LOC122586310	-3.65	0.0033	-2.29	0.1780	1.36	0.0990	probable WRKY transcription factor 70
XM 043781125.1	LOC122608043	-2.20	< 0.0001	-2.27	< 0.0001	-3.77	0.0002	probable WRKY transcription factor 70
XM 043755716.1	LOC122583290	-1.53	0.0035	-2.94	< 0.0001	-3.10	< 0.0001	probable WRKY transcription factor 57, transcript variant X2
XM 043757564.1	LOC122587096	-1.46	< 0.0001	-1.05	< 0.0001	-0.80	0.1490	probable WRKY transcription factor 40, transcript variant X2
XM 043759171.1	LOC122587096	-1.40	0.0048	-1.29	< 0.0001	0.11	0.8070	probable WRKY transcription factor 40, transcript variant X2
XM 043781243.1	LOC122608151	-13.57	0.0199	-13.57	0.0194	-13.30	0.0294	probable WRKY transcription factor 70, transcript variant X1
XM 043773250.1	LOC122600519	-2.43	0.0017	-2.27	0.0004	0.16	0.6860	probable WRKY transcription factor 11
XM 043756758.1	LOC122584711	0.86	0.2130	1.74	0.0010	0.88	0.0550	probable WRKY transcription factor 56
XM 043752532.1	LOC122580253	2.89	0.3100	3.47	0.0181	0.58	0.5610	probable WRKY transcription factor 75
XM 043763360.1	LOC122591144	-1.52	0.0520	-1.59	< 0.0001	-0.08	0.9280	auxin response factor 3, transcript variant X1
XM 043780995.1	LOC122607920	3.15	0.0520	3.88	0.0043	0.73	0.6370	auxin-induced in root cultures protein 12-like
XM 043757855.1	LOC122585730	-1.15	0.3110	-2.46	0.0110	-1.32	0.1370	auxin-responsive protein SAUR15
XM 043758165.1	LOC122586054	-0.73	0.3870	-1.47	0.0474	-0.74	0.1330	auxin transporter-like protein 2
XM 043775529.1	LOC122602916	-13.76	< 0.0001	-13.76	< 0.0001	/	/	auxin-induced protein PCNT115-like, transcript variant X2
XM 043762651.1	LOC122590447	-2.63	0.0027	0.24	0.0670	-2.13	0.0059	auxin-induced protein 15A-like
XM 043783284.1	LOC122610290	-1.67	0.0085	0.81	0.3080	0.89	0.0990	auxin response factor 18-like, transcript variant X2
XM 043770729.1	LOC122598126	-2.20	< 0.0001	0.88	0.0640	1.50	0.0870	auxin response factor 18-like
XM 043765822.1	LOC122593413	-2.04	0.0062	-2.43	0.0008	-0.39	0.1640	auxin-responsive protein IAA11-like

XM 043771792.1	LOC122599298	-15.89	< 0.0001	-15.89	< 0.0001	/	/	auxin response factor 9, transcript variant X1
XM 043761958.1	LOC122589641	4.51	0.0036	4.39	0.1070	-1.71	0.1260	auxin response factor 5-like
XM 043783006.1	LOC122610033	-8.39	0.0044	-15.45	0.0410	/	/	auxin response factor 8-like, transcript variant X2
XM 043783096.1	LOC122610099	2.39	0.0001	1.28	< 0.0001	-1.10	0.0450	auxin response factor 4-like
XM 043765747.1	LOC122593354	-13.88	0.0092	-0.01	0.9940	/	/	auxin response factor 1-like, transcript variant X2
XM 043749909.1	LOC122578045	-12.86	0.0076	-12.86	0.0060	/	/	auxin-responsive protein IAA29, transcript variant X1
XM 043771468.1	LOC122599021	-2.95	< 0.0001	-3.54	< 0.0001	-0.58	0.3830	auxin response factor 7-like
XM 043752849.1	LOC122580579	-1.79	0.0006	-0.92	0.1790	0.86	0.0530	auxin response factor 2A-like
XM 043774699.1	LOC122601966	-3.56	< 0.0001	-1.79	0.0128	1.77	0.0840	auxin-responsive protein IAA16-like
XM 043772809.1	LOC122600142	-1.35	0.0004	-1.02	0.0195	0.32	0.5620	bHLH transcription factor RHL1-like
XM 043767610.1	LOC122595271	-5.15	< 0.0001	-4.28	< 0.0001	0.87	0.1890	transcription factor bHLH35-like
XM 043750328.1	LOC122578380	-2.07	0.0004	-1.96	0.0011	0.10	0.0850	transcription factor bHLH162-like
XM 043777195.1	LOC122604304	-1.35	0.0069	-0.67	0.4040	0.68	0.5230	transcription factor bHLH62-like, transcript variant X1
XM 043751479.1	LOC122579325	-1.02	0.0323	0.16	0.6710	1.18	0.0570	transcription factor bHLH123-like
XM 043751175.1	LOC122579082	-1.73	0.1290	-7.44	< 0.0001	-9.12	0.0014	transcription factor bHLH47, transcript variant X1
XM 043764565.1	LOC122592357	4.64	0.3740	13.71	< 0.0001	13.71	< 0.0001	transcription factor bHLH112-like, transcript variant X2
XM 043759555.1	LOC122587397	1.60	0.2810	-14.64	0.0046	/	/	transcription factor bHLH110-like, transcript variant X1
XM 043762364.1	LOC122590027	-3.26	0.0002	-2.92	0.0001	-0.58	0.3770	transcription factor bHLH18-like
XM 043776715.1	LOC122603876	-0.75	0.3590	-1.39	0.0336	-0.64	0.5620	transcription factor bHLH18-like
XM 043780165.1	LOC122607227	-4.39	< 0.0001	-5.72	< 0.0001	-6.63	< 0.0001	NAC transcription factor 32-like
XM 043757551.1	LOC122585417	-1.18	0.0105	1.47	0.4970	1.06	0.0580	NAC domain-containing protein 87-like
XM 043750614.1	LOC122578618	-2.34	< 0.0001	2.21	0.2750	2.96	0.0780	NAC domain-containing protein 83-like
XM 043781265.1	LOC122608175	-2.71	0.0004	-4.33	< 0.0001	-1.61	0.1340	NAC domain-containing protein 83-like
XM 043751327.1	LOC122579210	-0.35	0.5060	-1.20	< 0.0001	-2.56	0.0476	NAC domain-containing protein 83-like
XM 043758497.1	LOC122586514	-1.02	0.0209	-1.33	< 0.0001	-0.30	0.4230	NAC domain-containing protein 91-like, transcript variant X1
XM 043758498.1	LOC122586514	-1.41	0.0187	-1.44	0.0055	-0.02	0.9630	NAC domain-containing protein 91-like, transcript variant X1
XM 043756216.1	LOC122583835	0.40	0.1010	-12.18	0.0056	/	/	NAC domain-containing protein 90-like
XM 043757379.1	LOC122585261	-5.04	< 0.0001	-2.47	0.1690	3.61	0.0087	NAC domain-containing protein 90-like
XM 043752094.1	LOC122579844	2.32	0.0329	2.70	0.0001	0.38	0.3980	NAC domain-containing protein 21/22-like
XM 043761570.1	LOC122589293	-2.06	0.0181	1.26	0.0670	1.74	0.1020	bZIP transcription factor 27-like, transcript variant X2
XM 043755206.1	LOC122582773	3.66	< 0.0001	3.87	< 0.0001	0.22	0.5650	bZIP transcription factor 53-like
XM 043779628.1	LOC122606771	1.28	0.0690	2.43	0.0149	1.15	0.0740	bZIP transcription factor 23-like, transcript variant X1
XM 043784335.1	LOC122611322	-0.62	0.4320	-1.02	0.0309	-0.40	0.7200	bZIP transcription factor 68-like, transcript variant X1
XM 043770641.1	LOC122598035	2.70	0.1900	2.37	0.0015	1.26	0.0560	bZIP transcription factor 11-like
XM 043780395.1	LOC122607423	2.01	0.0001	1.32	0.0510	-0.69	0.0960	bZIP transcription factor 11-like
XM 043778374.1	LOC122605423	2.35	0.0234	2.43	0.0065	0.07	0.8320	transcription factor TCP20-like
XM 043751476.1	LOC122579323	-1.24	< 0.0001	0.86	0.0870	0.51	0.0880	transcription factor TCP7-like
XM 043750722.1	LOC122578704	-3.29	0.0007	-3.82	< 0.0001	-0.53	0.2430	transcription factor TCP15-like
XM 043776823.1	LOC122603955	-0.34	0.0890	-1.31	0.0012	-2.34	0.0017	transcription factor TCP19
XM 043765838.1	LOC122593428	1.72	0.0960	2.24	0.0041	0.52	0.3370	transcription factor TCP4-like, transcript variant X1
XM 043765593.1	LOC122593214	1.84	0.3120	-2.56	< 0.0001	-2.73	0.0083	transcription factor TCP9-like
XR 006320710.1	LOC122579897	-0.80	0.2780	-1.82	0.0460	-1.02	0.0820	trihelix transcription factor GT-4, transcript variant X4
XM 043773442.1	LOC122600688	0.33	0.7700	-2.81	0.0175	-3.14	0.0168	trihelix transcription factor ASIL1, transcript variant X3
XM 043773599.1	LOC122600828	1.54	0.3270	2.67	0.0381	1.12	0.1250	trihelix transcription factor ASR3-like, transcript variant X2
XM 043754157.1	LOC122581850	1.47	0.0530	2.51	0.0047	1.04	0.0800	trihelix transcription factor ASIL2-like
XM 043784002.1	LOC122611049	1.02	0.0910	2.44	< 0.0001	1.42	0.0570	trihelix transcription factor DF1
XM 043783735.1	LOC122610777	-3.04	0.0050	-14.71	< 0.0001	-13.44	0.0468	basic leucine zipper 43-like
XM 043772166.1	LOC122599628	-1.44	< 0.0001	-0.68	0.0760	2.01	0.0197	homeobox-leucine zipper protein HAT4
XM 043773163.1	LOC122600451	2.00	0.0154	0.44	0.1460	-1.57	0.0690	basic leucine zipper 9
XM 043759605.1	LOC122587437	-1.54	0.0127	-0.56	0.1020	0.98	0.1370	homeobox-leucine zipper protein ATHB-15, transcript variant X1
XM 043774356.1	LOC122601604	-4.46	0.0176	-0.69	0.0530	3.77	0.0510	homeobox-leucine zipper protein ANTHOCYANINLESS 2
XM 043768528.1	LOC122596028	1.74	0.0002	1.53	0.0690	-0.21	0.4130	homeobox-leucine zipper protein ATHB-15-like
XM 043760908.1	LOC122596028	3.32	0.1290	1.53	0.0041	0.15	0.8250	homeobox-leucine zipper protein ATHB-15-like
XM 043752901.1	LOC122580633	-2.46	0.1470	-11.69	0.0440	/	/	homeobox-leucine zipper protein HDG11-like
XM 043772166.1	LOC122588735	-2.82	0.0122	-2.66	0.0640	-0.83	0.1070	homeobox-leucine zipper protein HAT5

XM 043760908.1	LOC122588735	-2.82	0.1290	-2.66	0.0061	0.15	0.8250	homeobox-leucine zipper protein HAT5
XM 043754521.1	LOC122582163	2.36	0.2330	2.02	0.0003	1.25	0.0720	homeobox-leucine zipper protein HOX11-like
XM 043768528.1	LOC122588476	-1.20	0.0510	-2.95	< 0.0001	-0.21	0.4130	GATA transcription factor 8-like, transcript variant X1
XM 043760603.1	LOC122588476	-15.43	< 0.0001	-15.43	< 0.0001	-14.82	< 0.0001	GATA transcription factor 8-like, transcript variant X1
XM 043768098.1	LOC122595678	2.38	0.0037	2.30	0.3040	-1.98	0.0426	GATA transcription factor 24-like, transcript variant X1
XM 043783306.1	LOC122610310	-2.84	< 0.0001	-3.59	< 0.0001	-0.76	0.0590	GATA transcription factor 8-like
XM 043781275.1	LOC122608185	-0.36	0.6120	-3.55	0.0013	-4.29	0.0187	nuclear transcription factor Y subunit B-5-like
XM 043774532.1	LOC122601797	1.20	0.5280	-1.57	0.0201	-1.18	0.2740	nuclear transcription factor Y subunit C-3-like
XM 043752874.1	LOC122580601	-2.48	0.0007	-2.03	0.0168	0.45	0.7050	nuclear transcription factor Y subunit B-1-like, transcript variant X6
XM 043752881.1	LOC122580601	-2.33	0.0136	-2.58	0.0009	-0.81	0.7290	nuclear transcription factor Y subunit B-1-like, transcript variant X6
XM 043768092.1	LOC122595672	-1.85	0.0456	1.68	0.8750	1.95	0.0650	nuclear transcription factor Y subunit B-3-like
XM 043780778.1	LOC122607746	-16.37	0.0436	1.18	0.7950	/	/	nuclear transcription factor Y subunit A-3-like, transcript variant X2
XM 043751483.1	LOC122579330	-2.53	0.0010	-13.84	< 0.0001	/	/	transcription factor TGA3-like
XM 043781204.1	LOC122608115	-1.74	< 0.0001	-1.72	0.0060	0.02	0.9740	transcription factor TGA4-like, transcript variant X1
XM 043776147.1	LOC122603444	-14.18	0.0071	-14.18	0.0066	/	/	transcription factor TGA2.3-like, transcript variant X2
XM 043751916.1	LOC122579695	1.38	0.2450	2.99	0.0139	1.62	0.0540	transcription factor EGL1, transcript variant X1
XM 043749982.1	LOC122578102	2.11	0.1150	3.52	0.0277	1.41	0.1970	transcription factor HY5-like
XM 043762475.1	LOC122590138	-2.97	0.0530	-13.12	0.0150	/	/	transcription factor ILI6-like
XM 043772947.1	LOC122600254	-0.98	0.0580	-1.31	0.0002	-0.32	0.0900	transcription factor GTE3, chloroplastic-like, transcript variant X3
XM 043749941.1	LOC122578071	-1.20	0.0182	1.93	0.5650	1.55	0.1220	transcription factor BIM1
XM 043780802.1	LOC122607766	-1.36	0.0151	0.89	0.2100	0.67	0.0780	transcription factor BIM1-like, transcript variant X1
XM 043780803.1	LOC122607766	-14.20	0.0027	-14.20	0.0024	/	/	transcription factor BIM1-like, transcript variant X1
XM 043773415.1	LOC122600663	2.09	0.0027	2.33	0.1220	0.24	0.7340	transcription factor LHW-like
XM 043778926.1	LOC122605962	-2.63	0.0119	1.20	0.1960	3.83	0.0550	transcription factor LHW, transcript variant X2
XM 043773150.1	LOC122600439	-11.27	0.0166	0.31	0.1790	/	/	transcription factor MYC2-like, transcript variant X2
XM 043756307.1	LOC122583931	1.81	< 0.0001	1.01	0.0770	-0.80	0.0960	basic transcription factor 3-like
XM 043759570.1	LOC122587408	-2.35	< 0.0001	-1.73	0.0002	0.62	0.3790	transcription factor AS1-like
XM 043764845.1	LOC122592583	-1.08	< 0.0001	-1.52	0.0690	-0.44	0.1130	helicase-like transcription factor CHR28
XM 043783841.1	LOC122610885	-2.43	0.0100	-3.06	0.0015	-0.63	0.3290	transcription factor WER-like
XM 043764599.1	LOC122592386	-1.92	0.0357	-0.38	0.1580	-0.05	0.6480	scarecrow-like transcription factor PAT1
XM 043781714.1	LOC122608620	-2.40	< 0.0001	-1.56	< 0.0001	0.84	0.2300	transcription factor GTE10-like
XM 043769148.1	LOC122596546	-13.71	0.0081	1.10	0.7610	/	/	transcription factor VOZ1, transcript variant X1
XM 043781567.1	LOC122608460	-2.16	0.0474	0.41	0.3120	0.98	0.3280	transcription factor DIVARICATA-like
XM 043756891.1	LOC122584827	-1.67	< 0.0001	-1.11	0.0002	0.57	0.0990	transcription factor ILR3-like
XM 043768138.1	LOC122595712	-1.48	0.0040	-0.63	0.2420	0.85	0.0720	transcription factor MTB1-like
XM 043750778.1	LOC122578752	13.37	0.0152	6.61	0.3160	8.19	0.0437	agamous-like MADS-box protein AGL65, transcript variant X2
XM 043750779.1	LOC122578752	-1.25	0.0077	0.61	0.0550	-1.71	0.0194	agamous-like MADS-box protein AGL65, transcript variant X2
XM 043761370.1	LOC122589125	-1.80	0.0082	-0.80	0.0660	-0.58	0.4180	agamous-like MADS-box protein AGL12
XM 043779190.1	LOC122606227	-1.87	0.1820	-14.16	0.0001	/	/	agamous-like MADS-box protein AGL62
XM 043764850.1	LOC122592584	7.30	0.3730	15.62	< 0.0001	1.68	0.2120	MADS-box protein SOC1, transcript variant X4

CK, MC, and HC represent the control (no Cd or Pb addition), moderate concentration (2 mg/kg Cd and 180 mg/kg Pb), and high concentration (4 mg/kg Cd and 360 mg/kg Pb) treatments, respectively. Fold change was calculated using the mean of three biological replicates. The symbol "/" indicates that the denominator is 0 or the numerator is 0, in which case the calculation of the log function cannot be performed.

Table S10 Expression profiles of genes related to ribosome in *Erigeron canadensis* roots treated with heavy metals

Transcript id	Locus ID	MC versus CK		HC versus CK		HC versus MC		Gene description
		$\log_2$[Fold change (treatment/control)] [a]	*q* -value	$\log_2$[Fold change (treatment/control)] [a]	*q* -value	$\log_2$[Fold change (treatment$_{HC}$/treatment$_{MC}$)] [a]	*q* -value	
XM_043778104.1	LOC122605203	1.87	<0.0001	1.33	0.0010	-0.54	0.0050	30S ribosomal protein S17-like, transcript variant X2
XM_043756731.1	LOC122584684	1.95	0.0094	1.50	0.0190	-0.45	0.2600	40S ribosomal protein S10-1-like
XM_043766799.1	LOC122594321	1.44	0.0257	0.78	0.1280	0.67	0.0501	40S ribosomal protein S15a-like
XM_043776887.1	LOC122604007	1.51	0.0002	1.54	0.0520	0.03	0.9420	40S ribosomal protein S23
XM_043781558.1	LOC122608449	2.04	<0.0001	1.97	0.0032	-0.07	0.8500	40S ribosomal protein S24-1-like
XM_043750042.1	LOC122578147	1.25	0.0298	0.77	0.0770	-0.48	0.0520	40S ribosomal protein S12-like
XM_043771944.1	LOC122599433	1.46	0.0316	0.98	0.0690	-0.48	0.0880	40S ribosomal protein S8
XM_043779828.1	LOC122606933	1.49	0.0190	1.39	0.0050	-0.01	0.6520	40S ribosomal protein S26-1-like
XM_043765289.1	LOC122592954	2.27	<0.0001	0.54	0.0010	-1.08	< 0.0001	40S ribosomal protein S24-1
XM_043754076.1	LOC122581796	2.00	<0.0001	1.48	0.0072	-0.52	0.0560	40S ribosomal protein S14-3
XM_043751500.1	LOC122579342	1.55	0.0012	0.59	0.1230	-0.97	0.0590	40S ribosomal protein S28-2-like
XM_043749991.1	LOC122578109	1.59	0.0154	1.16	0.0010	-0.43	0.1000	40S ribosomal protein S3a
XM_043778079.1	LOC122605182	1.49	0.0208	1.16	0.0340	-0.33	0.3050	40S ribosomal protein S6-like
XM_043779790.1	LOC122606903	1.67	0.0008	1.04	0.0010	-0.64	0.1000	40S ribosomal protein S18
XM_043770654.1	LOC122598047	2.08	0.0253	1.74	0.0190	-0.35	0.3010	ubiquitin-40S ribosomal protein S27a-like
XM_043759427.1	LOC122587301	1.39	0.0471	0.88	0.0560	0.51	0.0550	40S ribosomal protein S19-3
XM_043765672.1	LOC122593282	2.03	0.0228	0.78	0.1340	-1.25	0.0590	40S ribosomal protein S15a-5-like
XM_043753019.1	LOC122580871	2.20	0.0004	1.58	0.0120	-0.62	0.0310	50S ribosomal protein L15
XM_043765785.1	LOC122593386	1.71	0.0173	1.05	0.0040	-0.66	0.0940	50S ribosomal protein L9-like
XM_043751536.1	LOC122579370	1.52	0.0034	0.93	0.0180	-0.59	0.0140	50S ribosomal protein L20
XM_043777537.1	LOC122604662	2.48	0.0004	2.63	0.0005	0.14	0.7700	50S ribosomal protein L9, chloroplastic
XM_043750998.1	LOC122578939	1.45	0.0060	1.17	0.0040	-0.28	0.1110	50S ribosomal protein L17-like
XM_043768198.1	LOC122595762	2.03	0.0001	2.13	< 0.0001	0.11	0.6500	50S ribosomal protein L18, chloroplastic
XM_043776745.1	LOC122603900	3.71	0.0148	2.35	0.0090	-1.36	0.0143	50S ribosomal protein L7/L12, transcript variant X1
XM_043776746.1	LOC122603900	2.31	0.0041	1.12	0.0810	-1.19	0.0157	50S ribosomal protein L7/L12, transcript variant X1
XM_043769130.1	LOC122596532	2.06	0.0076	0.63	0.0040	-1.43	0.0004	50S ribosomal protein L21, mitochondrial
XM_043778679.1	LOC122605721	1.50	0.0157	0.98	0.0200	-0.52	0.0460	50S ribosomal protein HLP, mitochondrial
XM_043768827.1	LOC122596276	1.73	0.0102	1.63	0.0364	-0.10	0.7080	50S ribosomal protein L3, chloroplastic
XM_043751704.1	LOC122579518	1.51	0.0024	0.89	< 0.0001	-0.62	0.0240	50S ribosomal protein L27-like
XM_043762283.1	LOC122589952	2.42	0.0352	3.57	0.0006	1.15	0.0580	50S ribosomal protein L29, chloroplastic
XM_043756277.1	LOC122583905	2.91	0.0233	3.87	< 0.0001	0.96	0.1740	50S ribosomal protein L18-like
XM_043759382.1	LOC122587267	3.21	<0.0001	1.54	0.0266	-1.68	< 0.0001	60S ribosomal protein L35a-1-like, transcript variant X2
XM_043751582.1	LOC122579409	1.95	<0.0001	1.34	0.0020	-0.61	0.0180	60S ribosomal protein L38-like
XM_043773846.1	LOC122601068	1.38	0.0338	0.61	< 0.0001	-0.77	0.0210	60S ribosomal protein L35a-1-like
XM_043762917.1	LOC122590742	1.85	0.0063	0.51	0.0840	-1.35	0.0003	ubiquitin-60S ribosomal protein L40, transcript variant X2
XM_043780487.1	LOC122607501	1.55	0.0236	1.13	< 0.0001	-0.42	0.1380	60S ribosomal protein L23
XM_043755153.1	LOC122582731	1.35	0.0202	0.86	0.0030	-0.49	0.0050	60S ribosomal protein L23
XM_043753486.1	LOC122581285	1.45	0.0471	1.06	0.0020	-0.38	0.1670	60S ribosomal protein L10a-1
XM_043779606.1	LOC122606747	1.94	<0.0001	1.38	0.0400	-0.55	0.0890	60S ribosomal protein L32-1-like
XM_043773000.1	LOC122600294	2.05	<0.0001	0.94	0.0170	-1.11	0.0137	60S ribosomal protein L37a
XM_043775319.1	LOC122602731	2.05	0.0067	1.19	0.0140	-0.86	0.0880	60S ribosomal protein L35a-1
XM_043770909.1	LOC122598316	1.99	0.0044	0.80	0.1300	-1.19	0.0370	60S ribosomal protein L11
XM_043781796.1	LOC122608702	2.49	<0.0001	0.81	0.0770	-1.68	0.0011	ubiquitin-60S ribosomal protein L40
XM_043769034.1	LOC122596454	2.12	0.0003	1.79	0.0040	-0.32	0.0140	60S ribosomal protein L37-2-like
XM_043752604.1	LOC122580332	2.38	0.0265	1.77	0.0050	-0.61	0.0280	60S ribosomal protein L34
XM_043779999.1	LOC122607079	1.41	0.0095	0.74	< 0.0001	-0.67	0.0020	60S ribosomal protein L21-1

XM_043774450.1	LOC122601707	2.39	<0.0001	1.09	0.0010	-1.31	0.0003	60S ribosomal protein L30-like
XM_043768966.1	LOC122596397	2.13	0.0077	0.92	0.0780	-1.21	< 0.0001	60S ribosomal protein L39-1
XM_043765150.1	LOC122592832	-1.67	0.0078	-1.23	0.0218	0.43	0.2510	60S ribosomal protein L3-like
XM_043754669.1	LOC122582298	2.08	0.0069	1.69	0.0157	-0.39	0.3820	60S ribosomal protein L14-2-like
XM_043780602.1	LOC122607594	2.19	0.0199	1.16	0.0190	-1.03	0.2210	60S ribosomal protein L14-2-like
XM_043770558.1	LOC122597964	2.47	<0.0001	1.05	0.0560	-1.41	0.0162	60S ribosomal protein L37a
XM_043751104.1	LOC122579024	2.11	0.0043	1.45	0.0390	-0.66	0.2580	60S ribosomal protein L9-like
XM_043764349.1	LOC122592142	2.51	0.0347	1.04	0.1350	-1.48	0.0225	60S ribosomal protein L31-like
XM_043757912.1	LOC122585787	1.82	0.0423	1.10	0.0030	-0.73	0.0210	60S ribosomal protein L6, mitochondrial-like
XM_043766018.1	LOC122593589	1.83	0.0001	0.63	0.0030	-1.20	< 0.0001	60S ribosomal protein L39-1
XM_043769277.1	LOC122596659	1.97	0.0004	1.03	0.0100	-0.94	0.0270	60S ribosomal protein L7-2
XM_043753714.1	LOC122581483	2.09	0.0005	0.99	0.0040	-1.10	0.0004	60S ribosomal protein L19-1-like
XM_043760538.1	LOC122588428	2.35	<0.0001	1.18	0.0020	-1.17	0.0001	60S ribosomal protein L24-like
XM_043762982.1	LOC122590813	1.27	0.0107	0.67	0.0590	-0.60	0.0650	60S ribosomal protein L11
XM_043783626.1	LOC122610653	3.31	<0.0001	1.49	0.0070	-1.82	0.0003	60S ribosomal protein L29-1-like, transcript variant X1
XM_043783634.1	LOC122610653	2.93	0.0168	1.10	0.0580	-1.83	0.0013	60S ribosomal protein L29-1-like, transcript variant X1
XM_043772178.1	LOC122599640	1.65	0.0124	0.50	0.0380	-1.14	0.0097	60S ribosomal protein L32-1-like
XM_043780100.1	LOC122607173	3.24	<0.0001	1.30	0.0070	-1.94	< 0.0001	60S ribosomal protein L22-3-like
XM_043754513.1	LOC122582157	3.05	<0.0001	1.84	< 0.0001	-1.20	0.0009	60S ribosomal protein L29-2-like
XM_043760672.1	LOC122588538	2.18	0.0001	0.84	0.0020	-1.34	0.0006	60S ribosomal protein L36-3-like
XM_043759577.1	LOC122587415	1.39	0.0017	1.56	0.0030	0.16	0.3580	ribosomal protein S7, mitochondrial
XM_043765842.1	LOC122593431	1.89	0.0110	2.06	0.0005	0.17	0.5520	mediator of RNA polymerase II transcription subunit 28
XM_043763568.1	LOC122591314	1.62	0.0004	1.32	0.0001	0.30	0.3150	RNA polymerase II C-terminal domain phosphatase-like 1
XM_043776690.1	LOC122603860	16.20	0.0085	/	/	-0.21	0.8260	mediator of RNA polymerase II transcription subunit 11, transcript variant X2
XM_043778254.1	LOC122605327	2.00	0.0222	1.33	0.0370	-0.67	0.0880	mediator of RNA polymerase II transcription subunit 22a-like
XM_043780095.1	LOC122607166	14.79	0.0240	/	/	-0.62	0.7150	mediator of RNA polymerase II transcription subunit 19a-like, transcript variant X1
XM_043781405.1	LOC122608312	2.14	0.0034	1.23	0.0010	-0.91	< 0.0001	DNA-directed RNA polymerase III subunit RPC8
XM_043770289.1	LOC122597724	1.91	0.0142	3.00	0.0017	1.09	0.1810	DNA-directed RNA polymerases II and IV subunit 5A-like
XM_043754227.1	LOC122581911	5.27	0.0007	-1.67	0.2180	-4.60	0.0373	mediator of RNA polymerase II transcription subunit 7a-like, transcript variant X1

CK, MC, and HC represent the control (no Cd or Pb addition), moderate concentration (2 mg/kg Cd and 180 mg/kg Pb), and high concentration (4 mg/kg Cd and 360 mg/kg Pb) treatments, respectively. Fold change was calculated using the mean of three biological replicates. The symbol "/" indicates that the denominator is 0 or the numerator is 0, in which case the calculation of the log function cannot be performed.

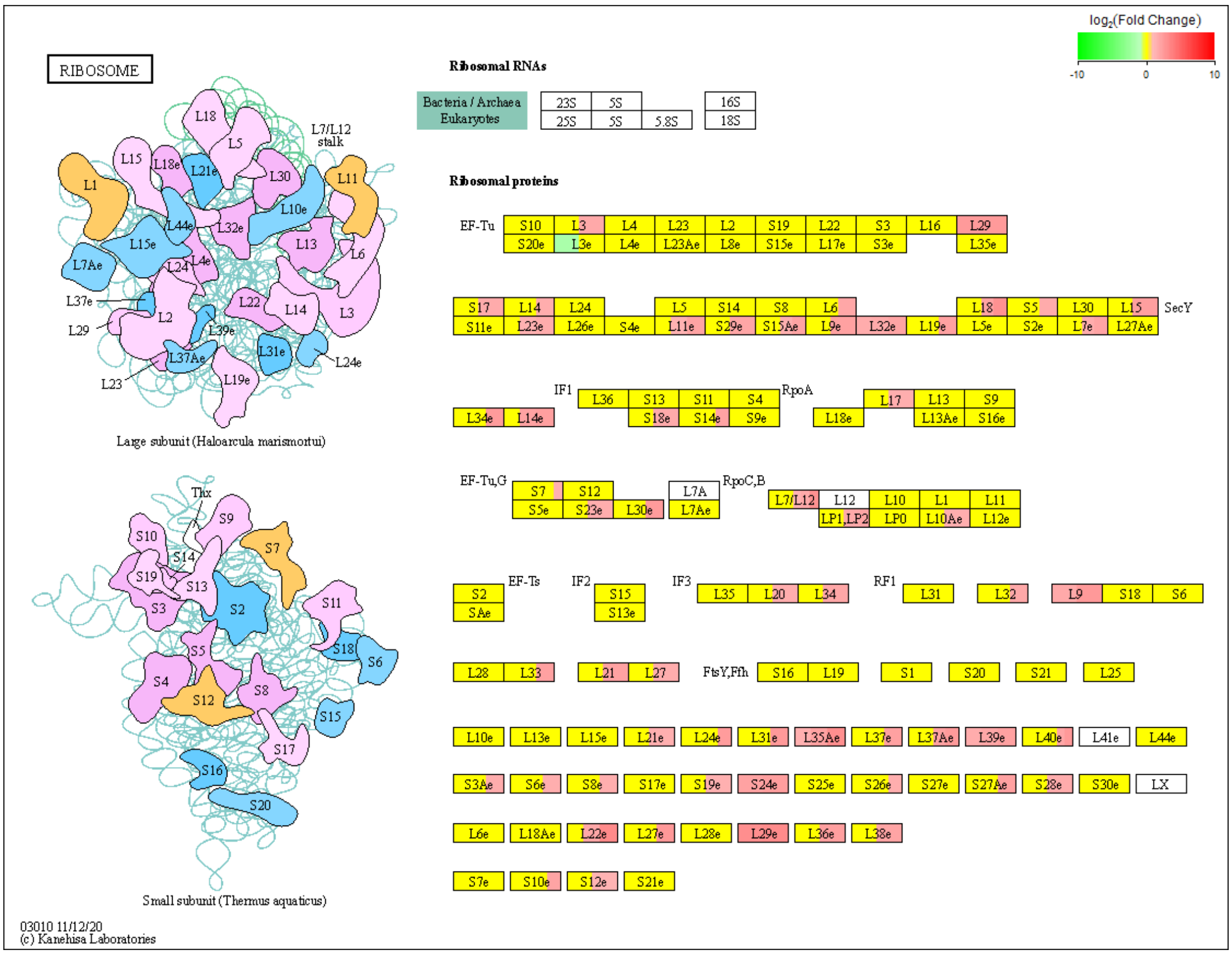

Figure S1. KEGG network of the ribosome pathway in *Erigeron canadensis* roots. The yellow color indicates no difference in gene expression, the green color indicates down-regulated genes, and red color indicates the up-regulated genes in *E. canadensis* roots. The white color in sharp-cornered rectangles indicates unannotated gene products in this pathway, and that in rounded rectangles indicates other associated KEGG pathways.

ALT TEXT: Graph depicting ribosome pathway network in *Erigeron canadensis* roots, with red, green, and yellow nodes representing up-regulated, down-regulated, and unchanged genes, respectively.

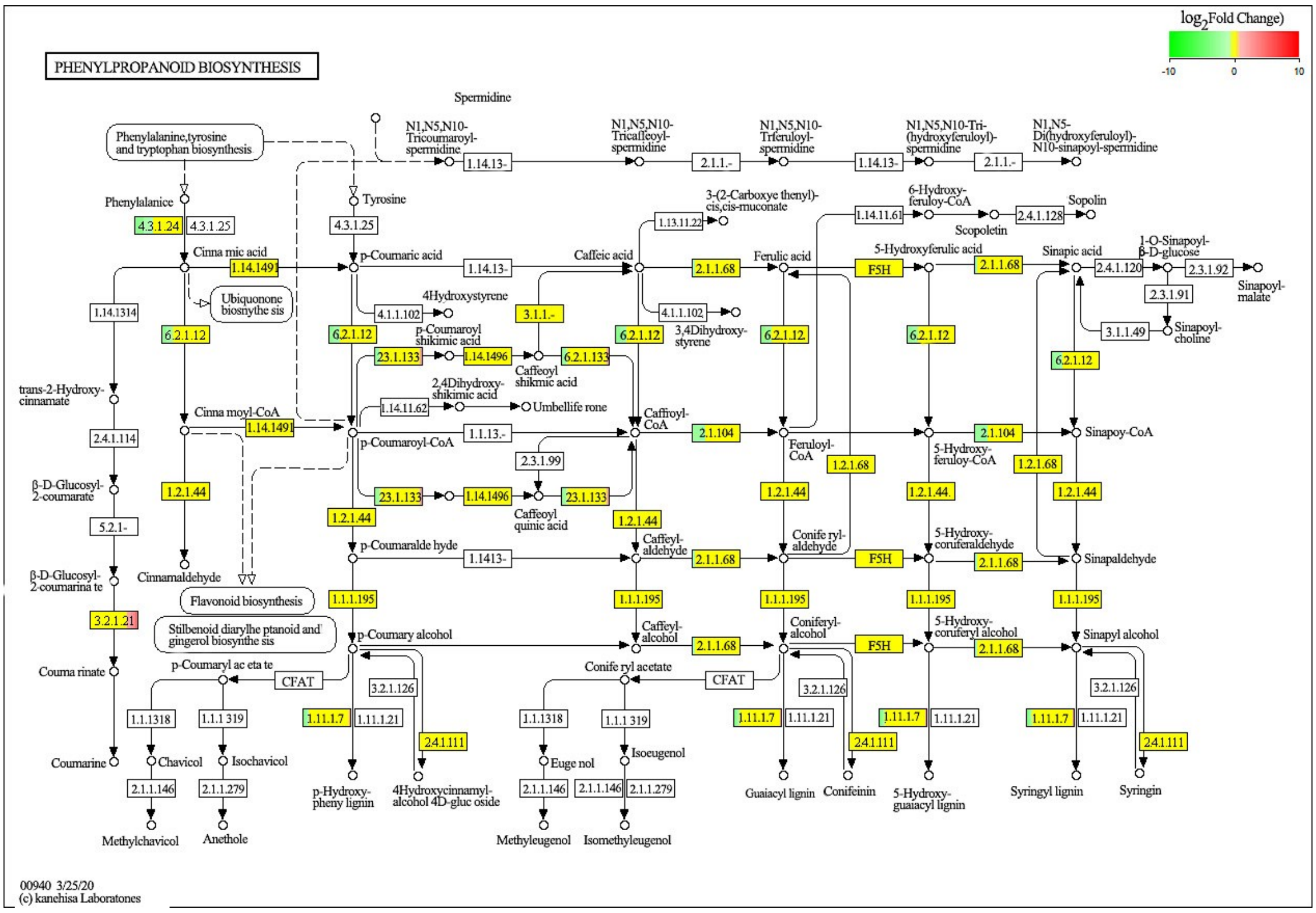

Figure S2. KEGG network of the phenylpropanoid biosynthesis in *Erigeron canadensis* roots. The yellow color indicates no difference in gene expression, the green color indicates down-regulated genes, and red color indicates the up-regulated genes in *E. canadensis* roots. The number in the box represents the EC number of enzymes. The white color in sharp-cornered rectangles indicates unannotated gene products in this pathway, and that in rounded rectangles indicates other associated KEGG pathways.

ALT TEXT: Graph depicting phenylpropanoid biosynthesis network in *Erigeron canadensis* roots, with boxed numbers representing EC enzyme identifiers.
